# Mechanistic Basis of the Cu(OAc)_2_ Catalyzed Azide-Ynamine (3 + 2) Cycloaddition Reaction

**DOI:** 10.1021/jacs.4c03348

**Published:** 2024-05-07

**Authors:** Roderick P. Bunschoten, Frederik Peschke, Andrea Taladriz-Sender, Emma Alexander, Matthew J. Andrews, Alan R. Kennedy, Neal J. Fazakerley, Guy C. Lloyd Jones, Allan J. B. Watson, Glenn A. Burley

**Affiliations:** † Department of Pure and Applied Chemistry, University of Strathclyde, Thomas Graham Building, 295 Cathedral Street, Glasgow G1 1XL, U.K.; ‡ EaStCHEM, Purdie Building, School of Chemistry, University of St Andrews, North Haugh, St Andrews, FifeKY16 9ST, U.K.; § GlaxoSmithKline, Medicines Research Centre, Gunnels Wood Road, Stevenage, Hertfordshire SG1 2NY, U.K.; ∥ EaStCHEM. School of Chemistry, University of Edinburgh, Edinburgh EH9 3FJ, U.K.

## Abstract

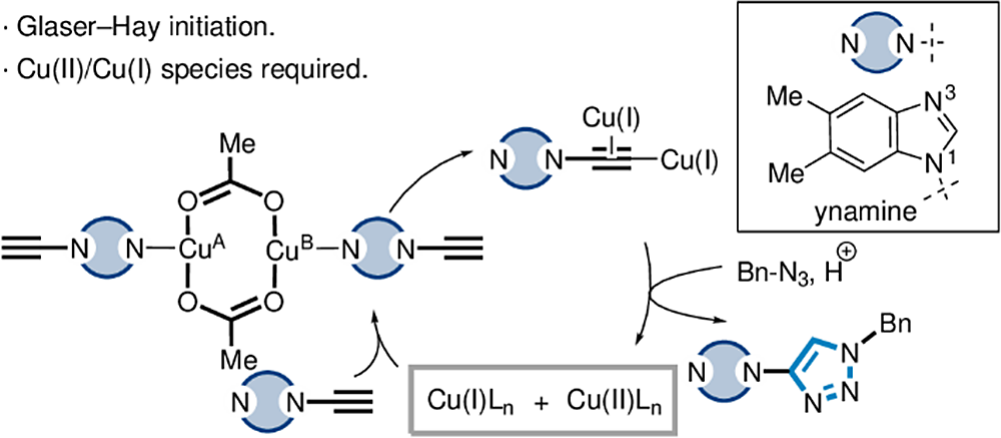

The Cu-catalyzed azide–alkyne cycloaddition (CuAAC) reaction is used as a ligation tool throughout chemical and biological sciences. Despite the pervasiveness of CuAAC, there is a need to develop more efficient methods to form 1,4-triazole ligated products with low loadings of Cu. In this paper, we disclose a mechanistic model for the ynamine-azide (3 + 2) cycloadditions catalyzed by copper(II) acetate. Using multinuclear nuclear magnetic resonance spectroscopy, electron paramagnetic resonance spectroscopy, and high-performance liquid chromatography analyses, a dual catalytic cycle is identified. First, the formation of a diyne species via Glaser–Hay coupling of a terminal ynamine forms a Cu(I) species competent to catalyze an ynamine-azide (3 + 2) cycloaddition. Second, the benzimidazole unit of the ynamine structure has multiple roles: assisting C–H activation, Cu coordination, and the formation of a postreaction resting state Cu complex after completion of the (3 + 2) cycloaddition. Finally, reactivation of the Cu resting state complex is shown by the addition of isotopically labeled ynamine and azide substrates to form a labeled 1,4-triazole product. This work provides a mechanistic basis for the use of mixed valency binuclear catalytic Cu species in conjunction with Cu-coordinating alkynes to afford superior reactivity in CuAAC reactions. Additionally, these data show how the CuAAC reaction kinetics can be modulated by changes to the alkyne substrate, which then has a predictable effect on the reaction mechanism.

## 1 Introduction

**Figure 1:**
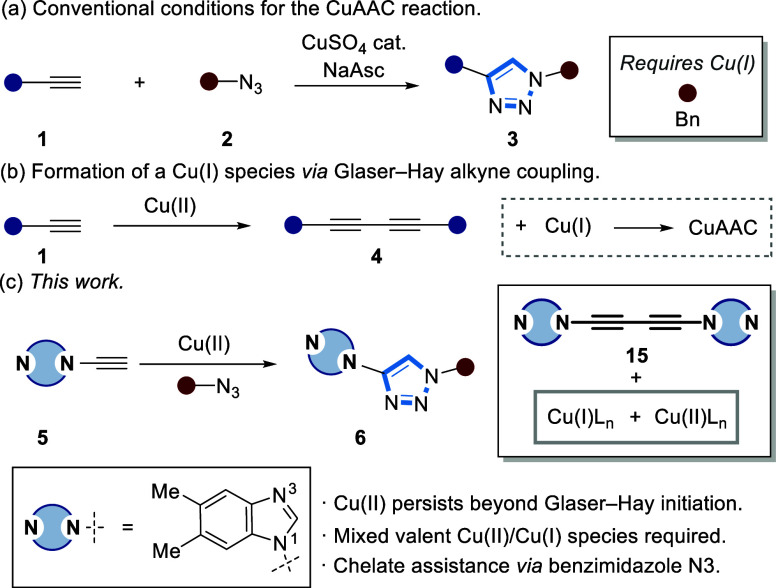
(a) Overview of the CuAAC reaction. (b) Accessing Cu(I) via Glaser–Hay coupling. (c) *This work:* Dynamic behavior of Cu in the ynamine-azide (3 + 2) cycloaddition through initiation and reaction promotion via coordination through interaction of the N3 benzimidazole position.

The Cu-catalyzed azide–alkyne (3 + 2) cycloaddition (CuAAC) is the most prominent class of “click” reactions.^1,2^ By virtue of fast reaction rates,^3^ the synthetic tractability of installing alkyne (**1**) and azide (**2**) groups in small molecules and biomolecules,^4^ and the regioselective formation of a 1,4-triazole product (**3**), the CuAAC is used across all facets of medicinal chemistry,^5^ chemical biology,^6−8^ and in the material sciences (Figure 1a).^9,10^

The CuAAC reaction typically proceeds with a Cu(II) source, which is subsequently reduced by the addition of a reductant (e.g., sodium ascorbate).^2^ Alternatively, the use of Cu(I) salts and stabilizing ligands obviates the addition of a reductant, thereby simplifying the operation of this reaction.^1^ A major limitation in the wider application of the CuAAC reaction in chemical biology workflows and the preparation of bioconjugates is the need for superstoichiometric quantities of Cu(I) for the reaction to proceed in aqueous buffers because the Cu typically coordinates to a variety of Lewis basic sites present in the solvent and in biomolecules.^11,12^ This invariably results in the onset of oxidative damage to proteins and nucleic acids due to the formation of Cu(I)-mediated reactive oxygen species^13−15^ and reduces the yield of bioconjugate products.^16^ Therefore, despite the broad uptake and development of the CuAAC platform, there is a distinct need to develop alternative CuAAC reactions, which produce a single ligated product, with low Cu loadings and without significantly compromising the reaction kinetics of the process.^17^

Previous mechanistic analyses of the catalytic cycle of the CuAAC reaction using conventional alkynes and a Cu(I) source have identified the formation of a Cu-acetylide species being rate-determining.^18−23^ A proposed mechanistic feature of the CuAAC is the formation of a binuclear Cu species, which is held together within a ligand architecture.^18,19,22,24,25^ These binuclear Cu complexes can potentially exist in a mixed valence state [i.e., Cu(I) and Cu(II)], possibly working in concert to form the Cu-acetylide species, facilitate azide ligation, and finally to initiate the (3 + 2) cycloaddition.^19,25−27^

While the in situ reduction of a Cu(II) precatalyst by sodium ascorbate (NaAsc) is the most widely used strategy to effect CuAAC reactions, an alternative approach is to access catalytically competent Cu(I) via a Cu(II)-catalyzed Glaser–Hay coupling of an alkyne (Figure 1b). In this case, a diyne product (e.g., **4**) is formed along with a Cu(I) species, which catalyzes the (3 + 2) cycloaddition reaction in the presence of an azide (e.g., **2**).^28−31^

The implication of diyne formation as a means to access a Cu(I) species suitable to catalyze the CuAAC reaction is that this requires coordination of Cu(II) with an alkyne substrate.^12,25,27,32^ At present, the molecular characteristics which influence alkyne reactivity as the sacrificial reductant have not been exploited in the context of CuAAC-mediated reaction chemistry.^33^

One approach to enhance the reactivity of an alkyne toward Cu is to conjugate a Lewis basic site with the triple bond. A pertinent example is the use of aromatic ynamines (e.g., **5**) in CuAAC reactions.^34−37^ These alkyne surrogates exhibit superior reactivity relative to alkyl and aromatic alkynes with azides to form 1,4-triazoles (**6**) using catalytic Cu(OAc)_2_ (Figure 1c).^36,37^

In earlier work, we highlighted a mechanistic divergence of the azide-ynamine (3 + 2) cycloaddition reaction in which the rate-determining step (RDS) shifts from Cu-acetylide formation to azide ligation of the Cu catalyst.^35^ However, these studies did not identify the role of Cu(OAc)_2_; whether the Cu(II) was a precatalyst that could form Cu(I) in situ via a Glaser coupling to form a diyne (e.g., **4**) or had other mechanistic significance.^25,33^

In this work, we present a mechanistic framework that demonstrates how the Cu catalyst influences ynamine reactivity in CuAAC reactions. A combination of heteronuclear nuclear magnetic resonance (NMR) and electron paramagnetic resonance (EPR) spectroscopy is used to elucidate an array of catalytic speciation derived from Cu(OAc)_2_, in which the oxidation states and interactions with the substrates, product, and solvent evolve over time. These data show that the Cu catalyst in the (3 + 2) cycloaddition reaction displays dynamic behavior^23^ through the stages of initiation and reaction promotion. Finally, we show that the triazole product (e.g., **6**) forms a complex with Cu, resulting in a catalytic resting state that can be reactivated by further addition of ynamine and azide substrates.

## 2 Results and Discussion

The objective of this work was to determine how the nature of the Cu catalyst influences the ynamine reactivity relative to those of cognate alkyne substrates. Our initial hypothesis was Cu(OAc)_2_ played a dual catalytic role: (i) the paddlewheel architecture assists in the formation of a diyne species (e.g., **4**) via a Glaser–Hay coupling of an ynamine (e.g., **5**); and (ii) the resultant Cu(I) species catalyzes the formation of a triazole product in the presence of a corresponding azide (e.g., **2**, Figure 1c).

### 2.1 Divergent Ynamine Reactivity Is Promoted by a Combination of Base and Cu Catalyst

**Figure 2:**
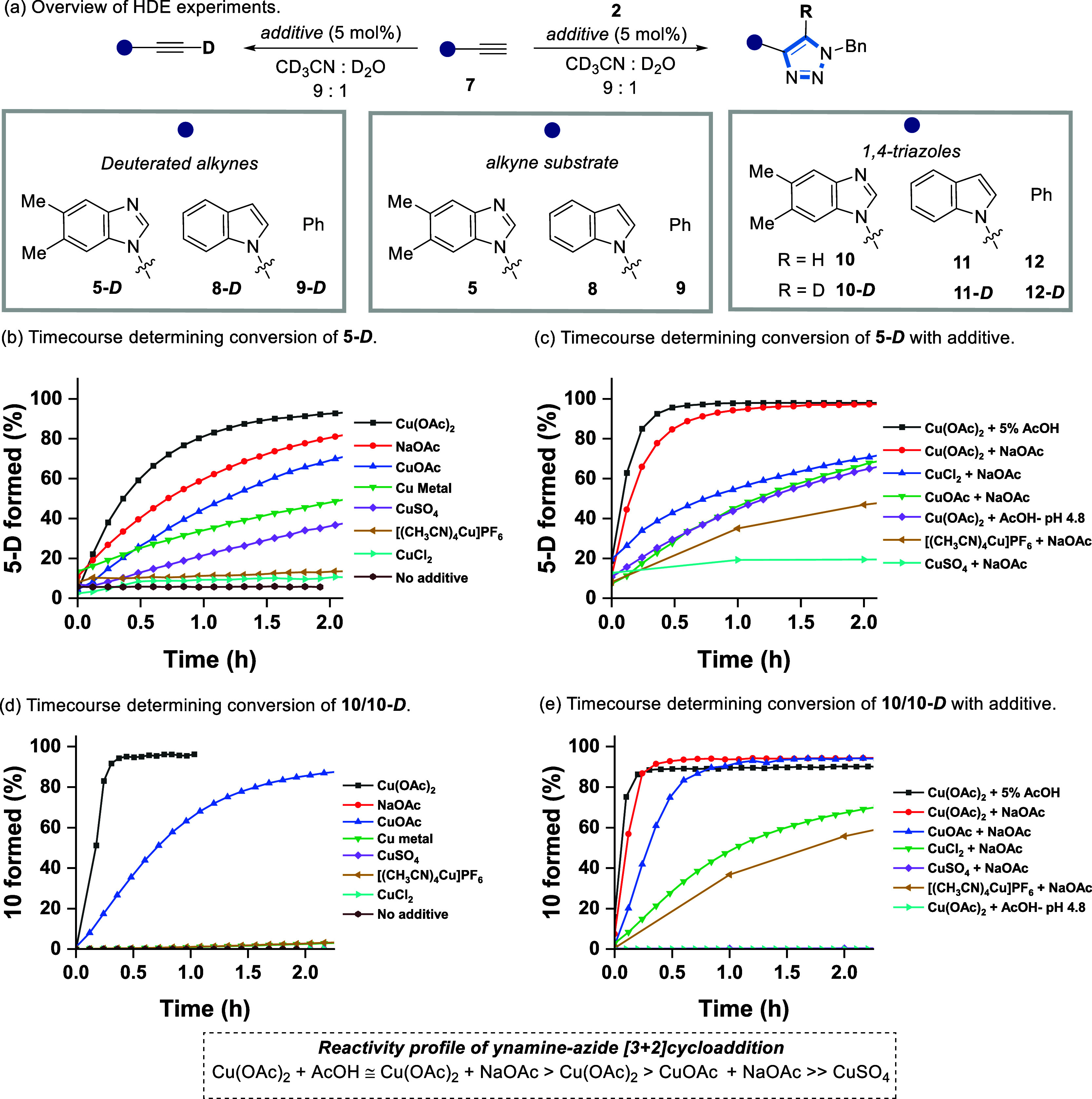
(a) Reaction scheme of ynamine HDE experiments as a function of additive in 9:1 CD_3_CN:D_2_O (for full scope, see Figures S18–S21). (b) Time course of HDE as a function of the Cu source to form **5-***D*. (c) Time course of HDE as a function of additive and Cu source to form **5-***D*. (d) Time course of triazole formation (**10/10-***D*) as a function of Cu source. (e) Time course of triazole formation (**10/10-***D*) as a function of additive and Cu source. [**5**] = 62 mM. Additional added NaOAc was 5 mol %. Lines through data are drawn solely as guides to the eye.

Hydrogen–deuterium exchange (HDE) of a terminal alkyne (**7**) was investigated in either CD_3_CN or a 9:1 mixture of CD_3_CN: D_2_O as a function of a 5 mol % additive (Figure 2a). All of the additives were soluble under experimental conditions, apart from CuOAc that was used as a suspension. Only 6% HDE was observed in a 9:1 CD_3_CN:D_2_O mixture when ynamine **5** was used (Figure 2c). The fastest HDE was observed using 5 mol % Cu(OAc)_2_ and either 5 mol % AcOH or NaOAc, forming 93% **5-***D* within 30 min (Figure 2c). Interestingly, HDE was slower when CuOAc was used (70% after 2 h), with a further reduction in the formation of **5-***D* observed in the presence of either CuSO_4_ (37%) or CuPF_6_ (13%). However, the rate of HDE increased when CuPF_6_ + NaOAc (5 mol %) was used (47%), suggestive of a cooperative effect between a Cu source and acetate. When only 5 mol % NaOAc was used, the rate of HDE of **5** to **5-***D* (81% after 2 h) was almost as fast as when Cu(OAc)_2_ was added. Finally, no enhancement in the rate of HDE was observed using **8** or **9** in the presence of any of these additives (Figures S13–S15), with the reaction mixture ≥pH 6 when 5 mol % NaOAc was added, regardless of the counterion (Figure S121).

The time course of the (3 + 2) cycloaddition to form 1,4-triazole **10/10-***D* as a function of the Cu catalyst (5 mol %) and salt additive was then monitored by ^1^H NMR spectroscopy in CD_3_CN:D_2_O 9:1 (Figure 2d). We identified that the use of deuterated solvents resulted in the formation of **10/10-***D* in a ratio of 1:9 using 5 mol % Cu(OAc)_2_ (Section S3.7) Without the addition of a catalyst, no formation of **10/10-***D* was detected over 2 h. Cu(OAc)_2_ was identified as the superior catalyst, with the formation of **10/10-***D* being complete (96%) after 26 min.

In comparison, the use of CuOAc resulted in slower formation of **10/10-***D* (complete ∼2 h) and neither CuSO_4_ or CuPF_6_ induced any detectable CuAAC (Figure 2e). However, the addition of NaOAc (5 mol %) to CuPF_6_ (5 mol %) did result in the formation of **10/10-***D*, albeit at a rate that was slower than that effected by direct use of Cu(OAc)_2_. This could suggest a potential inhibitory effect of the PF_6_ counterion. No conversion to **10/10-***D* was observed using either a mixture of 5 mol % of CuSO_4_ and NaOAc (Figure 2e), or NaOAc. However, the addition of 5 mol % of AcOH or NaOAc to 5 mol % Cu(OAc)_2_ or CuOAc enabled the CuAAC reaction to proceed (Figure 2e). Since CuOAc can form binuclear species and higher order aggregates,^38^ this suggests that a preformed Cu(OAc)_2_/CuOAc species is optimal to catalyze (3 + 2) cycloadditions.^39^

Alkynes **8** and **9** did not undergo the (3 + 2) cycloaddition with **2** using any of the Cu catalysts used with **5** as the alkyne surrogate when CD_3_CN:D_2_O (9:1) was used. Furthermore, triazole products **11/11-***D* and **12/12-***D* only formed when the reaction was conducted in pure CD_3_CN (Figures S19–S21). These results emphasize the divergent reactivity of **5** when the Cu-catalyzed (3 + 2) cycloaddition is performed in aqueous conditions.

Taken together, these results show that the HDE of **5** is predominantly promoted by the acetate ligand. Furthermore, the presence of the benzimidazole N3 in **5** is essential for HDE and the initiation of the Cu-catalyzed (3 + 2) cycloaddition reaction with **2**.

### 2.2 Diyne Formation Is a Source of Cu(I), Promoted by Benzimidazole N3 Coordination

**Figure 3:**
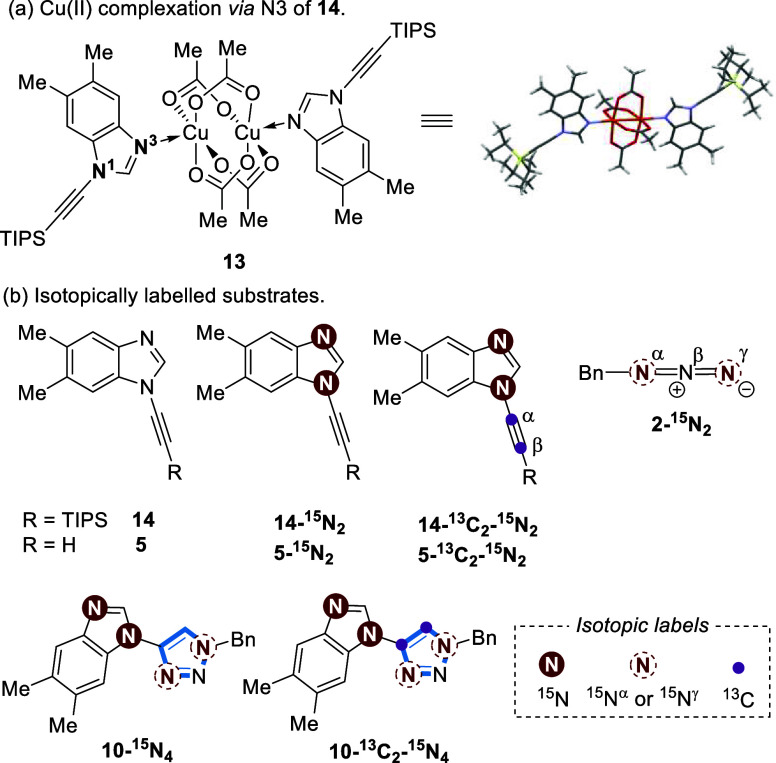
(a) Asymmetric unit cell of ynamine (**14**) with Cu(OAc)_2_. (b) Isotopically labeled compounds used in this study. Compound **2-**^**15**^**N** labeled on either N^α^ or N^γ^ positions.

We sought to further understand how Cu(OAc)_2_ could promote the Cu(I) catalyzed (3 + 2) cycloaddition between **5** and **2** to form **10**. Kuang et al. have proposed that Cu(I) could be formed either during a Cu(OAc)_2_ catalyzed Glaser–Hay reaction of an alkyne in MeCN or via a Cu-mediated oxidation of MeOH when used as a solvent,^25,40^ and that the Cu(I) species formed in situ could then catalyze the (3 + 2) cycloaddition reaction with a Cu-coordinating azide. In the case of ynamine **5**, benzimidazole N3 coordination by Cu(OAc)_2_ enhances the reactivity. Formation of complex (**13**) on mixing TIPS-protected ynamine (**14**) with Cu(OAc)_2_ confirmed coordination at the N3 position at the apical site of the Cu(OAc)_2_ paddlewheel structure (Figures 3a and S120). However, EPR measurements under a catalytic regime (10 mol % of Cu(OAc)_2_) showed that **14** can desymmetrize the paddlewheel complex in solution, resulting in a strong signal indicative of a Cu(II) species (Section S4.1). This suggests that the N3 position of **14** can denucleate the Cu(OAc)_2_ paddlewheel (present in 10 mol %), to form a mononuclear complex in MeCN, to deliver the catalytically active species.^41^

To further test the influence of N3 coordination in the (3 + 2) cycloaddition, a range of isotopically labeled ynamine (**5**) and benzyl azide (**2**) substrates and their corresponding triazole products were prepared, and then used to monitor the reaction by NMR spectroscopy (Figure 3b).

**Figure 4:**
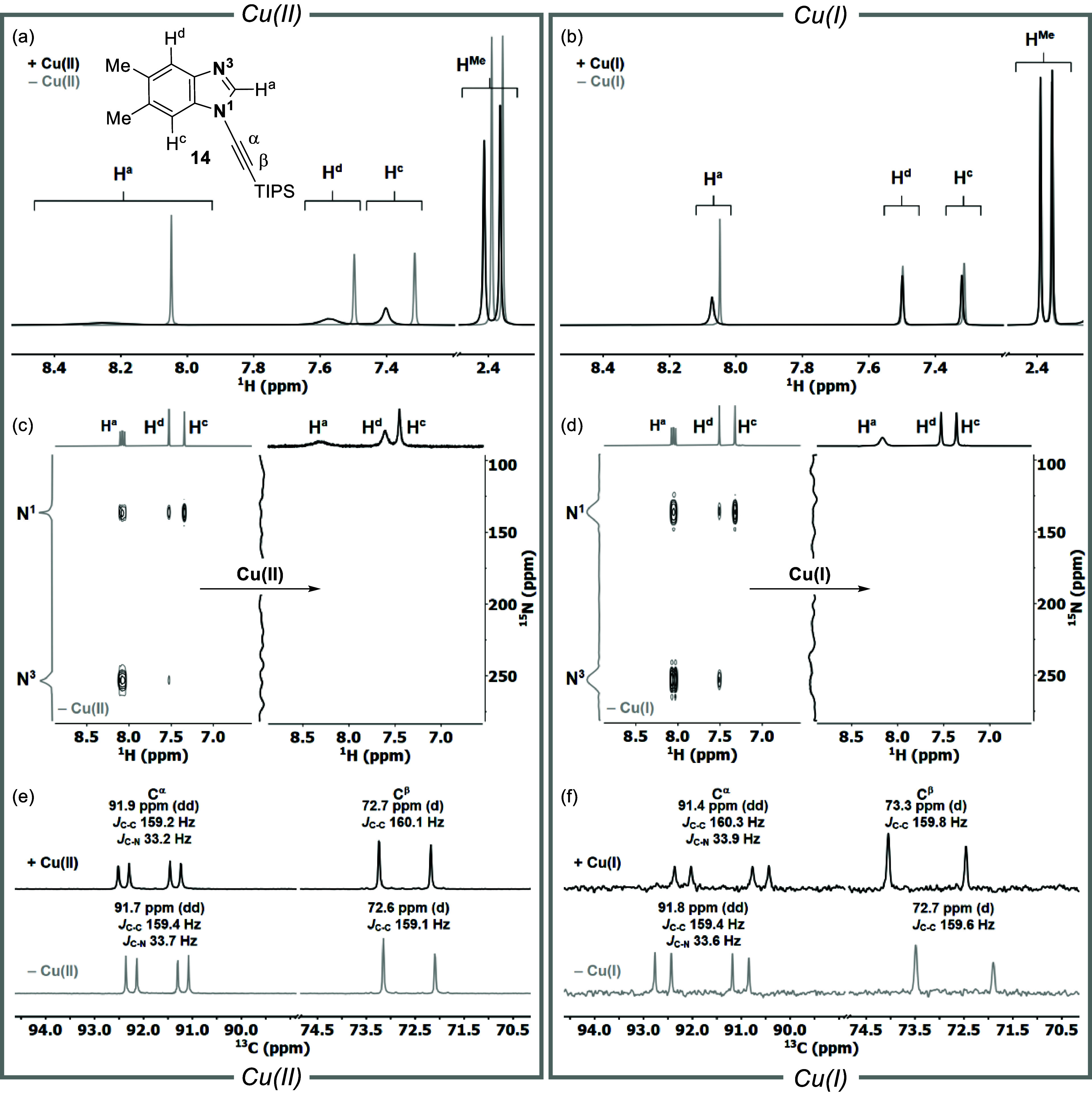
(a) ^1^H NMR spectra of **14** (gray) and upon the addition of 5 mol % Cu(OAc)_2_ (black). (b) ^1^H NMR spectra of **14** (gray) and upon addition of 5 mol % [(MeCN)_4_Cu]PF_6_ (black). (c) ^1^H–^15^N HMBC analysis of **14-**^**15**^**N**_**2**_ after the addition of 5 mol % Cu(OAc)_2_. (d) ^1^H–^15^N HMBC analysis of **14-**^**15**^**N**_**2**_ after the addition of 5 mol % [(MeCN)_4_Cu]PF_6_. (e) ^13^C NMR spectra of **14-**^**13**^**C**_**2**_**-**^**15**^**N**_**2**_ (gray) and after the addition of 5 mol % Cu(OAc)_2_ (black). (f) ^13^C NMR spectra of **14-**^**13**^**C**_**2**_**-**^**15**^**N**_**2**_ (gray) and after the addition of 1.0 equiv of [(MeCN)_4_Cu]PF_6_ to **14-**^**13**^**C**_**2**_**-**^**15**^**N**_**2**_ (black). All spectra using isotopically labeled compounds were acquired in CD_3_CN at an ynamine concentration of 15.5 mM (c–f), and 62 mM for natural abundant **5** (e.g., b, c).

We first explored the influence of the N3 position in a TIPS protected ynamine with Cu(OAc)_2_ and [(MeCN)_4_Cu]PF_6_ using **14**, **14-**^**15**^**N**_**2**_ and **14-**^**13**^**C**_**2**_**-**^**15**^**N**_**2**_. The addition of 5 mol % Cu(OAc)_2_ resulted in a broadening of ^1^H signals, with the extent of broadening increasing in the order, H^Me^, H^c^, H^d^, H^a^ (Figure 4a). This order reflects the increasing spatial and electronic proximity to the site of Cu(II) coordination, i.e., at N3.^42,43^ The impact of the addition of 5 mol % [(MeCN)_4_Cu]PF_6_ on the ^1^H NMR spectrum of **14** was far less pronounced than with 5 mol % Cu(OAc)_2_, with only significant signal broadening detected at H^a^, Figure 4b.

^1^H–^15^N HMBC experiments were then acquired with 5 mol % of both Cu catalysts using **14-**^**15**^**N**_**2**_ (Figure 4c,d). In neither case were the N1 or N3 resonances detected. Finally, negligible changes in the chemical shifts of C^α^ and C^β^ in **14-**^**13**^**C**_**2**_**-**^**15**^**N**_**2**_ were detected after the addition of 5 mol % Cu(OAc)_2_ (Figure 4e); this was also the case for [(MeCN)_4_Cu]PF_6_ (Figure S29). While the addition of one equivalent Cu(OAc)_2_ resulted in precipitation, the addition of one equivalent of [(MeCN)_4_Cu]PF_6_ resulted in an upfield shift of C^α^ and a concomitant downfield shift of the C^β^ resonance, suggesting a weak, long-range interaction between **14** and a Cu(I) source, likely via N3 coordination (Figure 4f).

**Figure 5:**
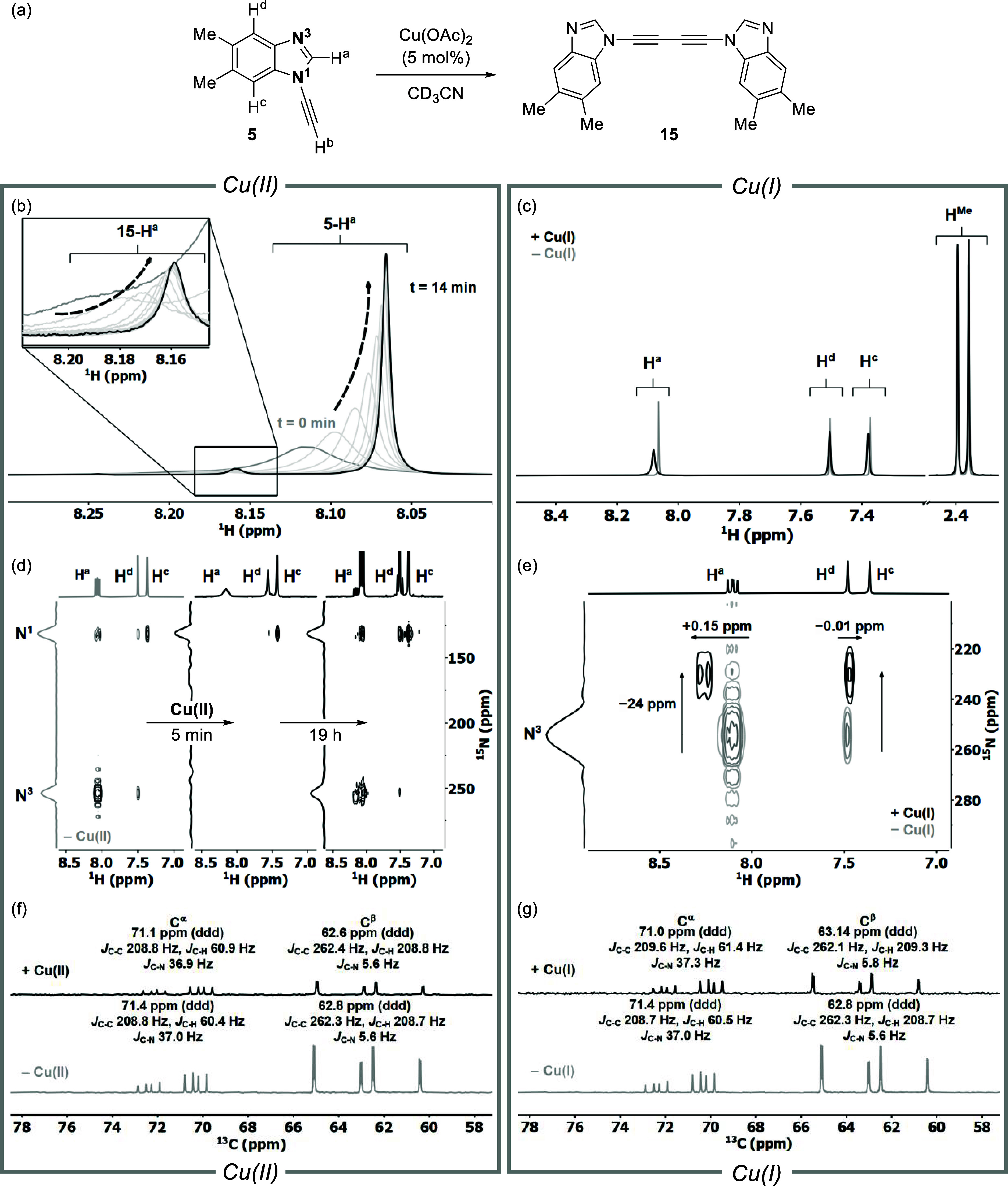
(a) Glaser–Hay coupling of **5** to form diyne **15** catalyzed by Cu(OAc)_2_. (b) Chemical shift change of H^a^ in **5** as a function of time in the presence of 5 mol % Cu(OAc)_2_ at 300 K. (c) Expansion plot of **5** in the presence of 5 mol % [(MeCN)_4_Cu]PF_6_. (d) ^1^H–^15^N HMBC analyses of the Glaser–Hay reaction of **5-**^**15**^**N**_**2**_ catalyzed by 5 mol % Cu(OAc)_2_ after 5 min and 19 h at 300 K. (e) ^1^H–^15^N HMBC analysis of **5-**^**15**^**N**_**2**_ upon addition of 1 equiv of [(MeCN)_4_Cu]PF_6_ at 233 K. (f) ^13^C NMR spectra of **5-**^**13**^**C**_**2**_**-**^**15**^**N**_**2**_ in CD_3_CN (gray) and in the presence of 5 mol % Cu(OAc)_2_ at 300 K (black). (g) ^13^C NMR spectra of **5-**^**13**^**C**_**2**_**-**^**15**^**N**_**2**_ in CD_3_CN at 300 K (gray) and in the presence of 1 equiv. [(MeCN)_4_Cu]PF_6_ (black).

Having established that Cu coordinates at the N3 position of **14**, the potential for a Cu catalyst to form diyne **15** via a Glaser–Hay coupling with **5** was explored (Figure 5a). The time course for the reaction of **5** in the presence of 5 mol % Cu(OAc)_2_ revealed immediate broadening of the ^1^H NMR signals of **5**, and an associated downfield shift of H^a^ (Figure 5b). Sharpening of the H^a^ resonance accompanied by an upfield shift then occurred over 14 min. Accompanying the sharpening of this resonance was the presence of a new set of peaks associated with the formation of **15**. This effect was not observed when 5 mol % [(MeCN)_4_Cu]PF_6_ was used (Figure 5c).

^1^H–^15^N HMBC spectra were acquired to monitor the Glaser–Hay coupling using **5-**^**15**^**N**_**2**_ in the presence of 5 mol % Cu(OAc)_2_. Spectra showed the characteristic disappearance of the ^15^N resonance for N3 after 5 min. In contrast to the resulting ^1^H–^15^N HMBC spectrum of **14-**^**15**^**N**_**2**_ on the addition of 5 mol % Cu(OAc)_2_ (Figure 4c), only the N3 resonance disappeared when 5 mol % Cu(OAc)_2_ was added to **5-**^**15**^**N**_**2**_ (Figure 5d). After 19 h, both ^15^N signals were detected alongside a set of new signals associated with diyne formation (**15-**^**15**^**N**_**2**_, Figure 5d). In stark contrast, the addition of 5 mol % [(MeCN)_4_Cu]PF_6_ to **5-**^**15**^**N**_**2**_, only changed the chemical shift of N3, detected at 233 K (Figure 5e). This data show that although both Cu catalysts coordinate to the N3 position of **5**, only the Cu(II) species induces Glaser–Hay coupling of **5** to form **15** prior to entering the CuAAC catalytic cycle. In contrast to the intense Cu(II) signals detected by EPR spectroscopy when **14** was mixed with Cu(OAc)_2_, analysis of a mixture of **5** with 10 mol % Cu(OAc)_2_ showed a weak signal, which decreased over time (Section S4.2). This is indicative of Cu(II) being reduced to a Cu(I) species.

**Figure 6:**
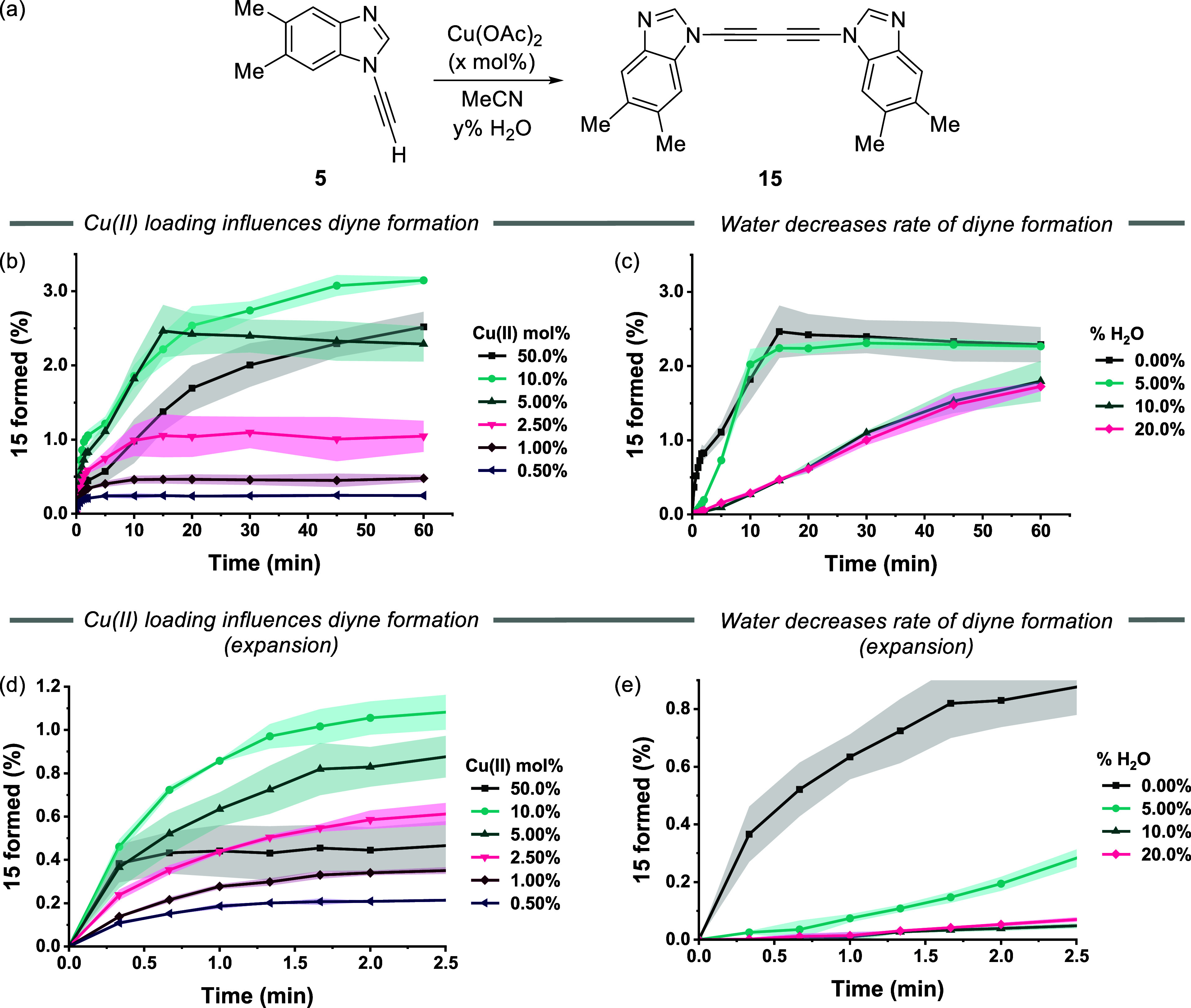
Catalyst loading and water effect on (a) formation of **15** from **5** as determined by RP-HPLC. Monitoring the conversion to **15** as a function of Cu(OAc)_2_ catalytic loading over 60 min as a function of (b) catalytic loading of Cu(OAc)_2_ and (c) water content (0–20% water). *Reaction conditions*: [**5**] 62 mM in MeCN. Initial rate of reaction over a 2.5 min time frame exploring the rate of formation of **15** as a function of (d) catalytic loading of Cu(OAc)_2_ and (e) water content (0–20% water). Lines through the data are solely a guide to the eye.

Previous studies on the Glaser–Hay coupling of diynes have revealed the type of N-ligand used influences the amount of diyne formed,^28,44^ with the benzimidazole N3 position known to be a Lewis basic site for both Cu(II) and Cu(I).^45,46^ We then explored if Cu loading influenced diyne (**15**) formation using **5** and catalytic Cu(OAc)_2_ by monitoring the reaction by high-performance liquid chromatography (HPLC; 0.5–50 mol %, Figure 6a).

There was a distinct correlation between Cu(OAc)_2_ loading and the formation of **15** when the catalyst loading range is 0.5–5 mol %, i.e., formation of 0.25% **15** using 0.5 mol % Cu(OAc)_2_, i.e., resulting in complete reduction of Cu(II) to a Cu(I) species (Section S6.4). Deviation from this correlation occurred when catalyst loading was in the 10–50 mol % range. These results demonstrate that Cu(OAc)_2_ forms **15**, thus producing a Cu(I) species, as supported by the reappearance of ^15^N resonances and the concomitant sharpening of ^1^H resonances after ∼15 min.

Using the HPLC assay, the influence of the water content on the rate of formation of **15** was explored (Figure 6c). This was an important factor to understand as water could compete with the benzimidazole N3 at the apical coordination sites of Cu(OAc)_2_,^28^ thus slowing down the Glaser–Hay coupling, and possibly leading to the slow accumulation of Cu(I) to catalyze the CuAAC reaction.^47^ In MeCN containing up to 5% water, approximately 2.5% conversion of **15** proceeded in 15 min when using 5 mol % Cu(OAc)_2_. However, when water was present at higher concentrations, e.g., 10–20% H_2_O, the rate of Glaser–Hay coupling was substantially reduced (Figure 6c).

### 2.3 Ynamine-Azide (3 + 2) Cycloaddition Is Catalyzed by a Cu(I) Species Formed In Situ

**Figure 7:**
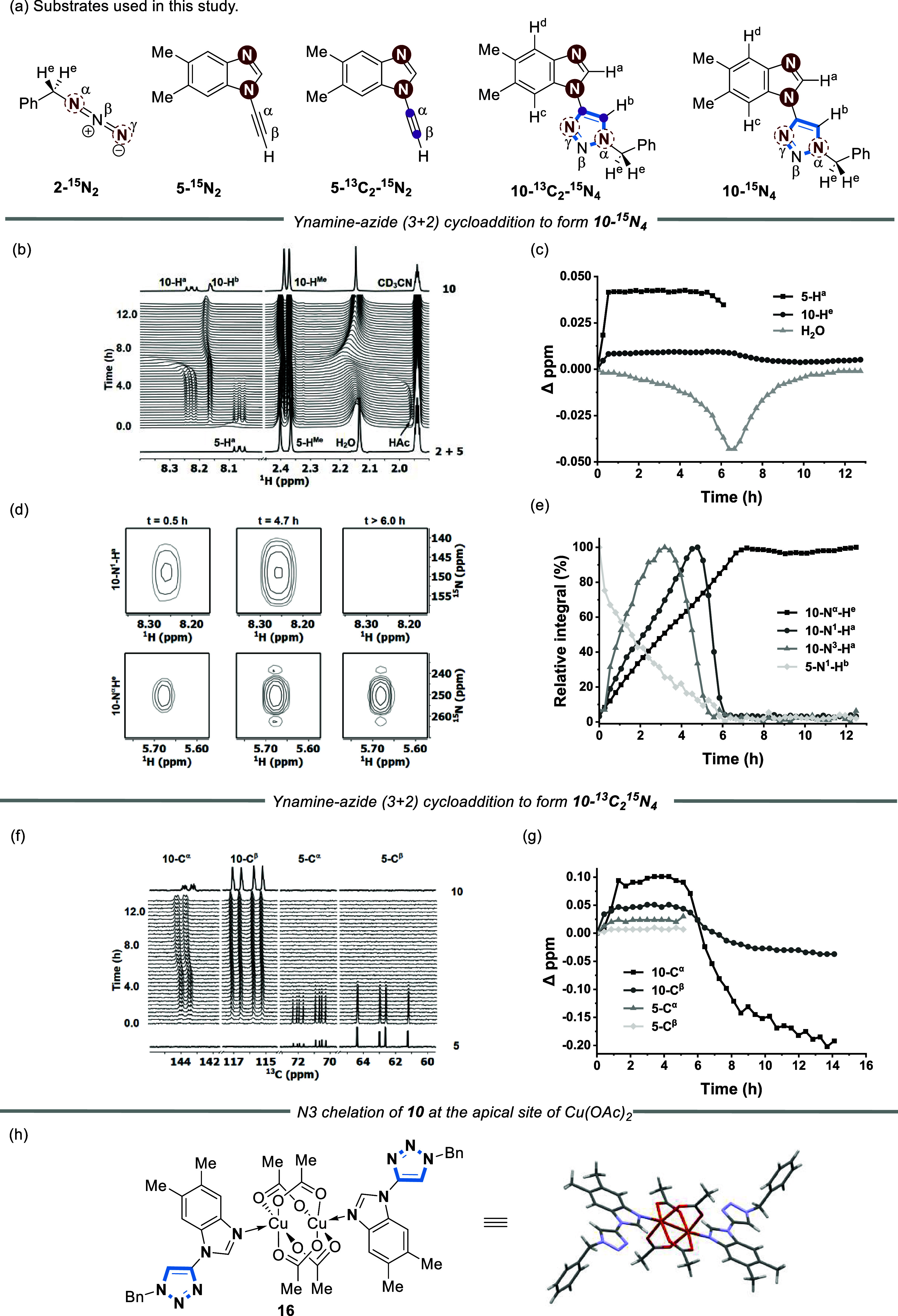
(a) Isotopically labeled substrates used in the reaction time course NMR experiments. Ynamine-azide (3 + 2) cycloaddition reaction conditions: 5 mol % Cu(OAc)_2_ in CD_3_CN at 300 K. [**5-**^**15**^**N**_**2**_] and [**2-**^**15**^**N**_**2**_] were 15.5 mM. (b) Time course of the formation of **10-**^**15**^**N**_**4**_ using **5-**^**15**^**N**_**2**_ and **2-**^**15**^**N**_**2**_ monitored over 13 h by ^1^H NMR. (c) Chemical shift changes of the (3 + 2) cycloaddition using **5-**^**15**^**N**_**2**_ and **2-**^**15**^**N**_**2**_ extracted from stacked 1D ^1^H data. (d) 2D ^1^H–^15^N HMBC expansion plots of N^1^–H^a^ and N^α^-H^e^ arising from the reaction forming **10-**^**15**^**N**_**4**_. (e) Relative intensities of ^1^H–^15^N correlations of the reaction extracted from stacked 2D ^1^H–^15^N HMBC spectra. (f) (3 + 2) cycloaddition reaction with Cu(OAc)_2_ in CD_3_CN. [**5-**^**13**^**C**_**2**_**-**^**15**^**N**_**2**_] and [**2-**^**15**^**N**_**2**_] = 15.5 mM. (g) Time course monitored over 13 h by ^13^C NMR. (h) Chemical shift changes of the reaction extracted from stacked 1D ^13^C data. (i) Asymmetric unit cell of complex **16** formed when **10** is mixed with Cu(OAc)_2_ in a 2:1 stoichiometry. Lines through data are solely a guide to the eye.

Having confirmed that Cu(I) is generated in situ via a Glaser–Hay coupling, we sought to further understand how the Cu catalyst evolves during the CuAAC reaction. The Cu-catalyzed reaction of labeled ynamines (**5-**^**15**^**N**_**2**_, and **5-**^**13**^**C**_**2**_**-**^**15**^**N**_**2**_, 15.5 mM) with azide (**2-**^**15**^**N**_**2**_, 15.5 mM) in CD_3_CN (Figure 7a) was monitored by various NMR techniques. First, we explored the formation of **10-**^**15**^**N**_**4**_ using **5-**^**15**^**N**_**2**_ and **2-**^**15**^**N**_**2**_ (Figure 7b–e). Under these conditions, the formation of triazole **10-**^**15**^**N**_**4**_ was complete within 6 h. However, a series of spectral changes was observed during the course of this reaction to form **10-**^**15**^**N**_**4**_ (Figure 7b,c). These included a broadening and downfield shift of both the H^a/b 1^H NMR signal in **10-**^**15**^**N**_**4**_ and the H_2_O resonance. Between 6 and 10 h, the water resonance returned to its original chemical shift position (Figure 7c). We surmise that the apical site on the Cu(OAc)_2_ paddlewheel structure is dynamic, with water competing with the N3 of **10-**^**15**^**N**_**4**_ toward the end of the reaction as **5-**^**15**^**N**_**4**_ is consumed. Further analysis of this phenomenon by ^1^H–^15^N HMBC also showed spectral changes in the intensity of the N^1^–H^a^ and N^α^-H^e^ cross peaks over time (Figure 7d,e). For example, the formation of **10-**^**15**^**N**_**4**_ was observed after 30 min, with the intensity of the N^α^-H^e^ cross peak remaining constant after 7 h.

A second set of experiments were conducted to provide further insight into changes in the alkyne by the reaction of **5-**^**13**^**C**_**2**_**-**^**15**^**N**_**2**_ with **2-**^**15**^**N**_**2**_ to form **10-**^**13**^**C**_**2**_**-**^**15**^**N**_**4**_ (Figure 7f,g). A distinct downfield chemical shift of the C^β^ resonance was observed before returning to its original position after 12 h. This suggests a structural change occurring with the Cu catalyst after 6 h involving coordination at the N3 position of the triazole product (**10-**^**13**^**C**_**2**_**-**^**15**^**N**_**4**_). For example, the formation of **10-**^**15**^**N**_**4**_ was observed after 30 min, with the intensity of the N^α^-H^e^ cross peak remaining constant after 7 h. In contrast, the corresponding N1/N3–H^a^ cross peak rapidly started to lose intensity after 4.7 h and completely disappeared after 12 h (Figure 7e).

EPR measurements under these reaction conditions show no signal for Cu(II) over the reaction time frame until 6 h. After 6 h a Cu(II) signal emerges, which suggests either reoxidation^48,49^ of a Cu(I) species to Cu(II) or a reorganization of an EPR active Cu species (Section S4.4).

### 2.4 Cu-Catalyst is in a Postreaction Resting State after Completion of the Ynamine-Azide (3 + 2) Cycloaddition

**Figure 8:**
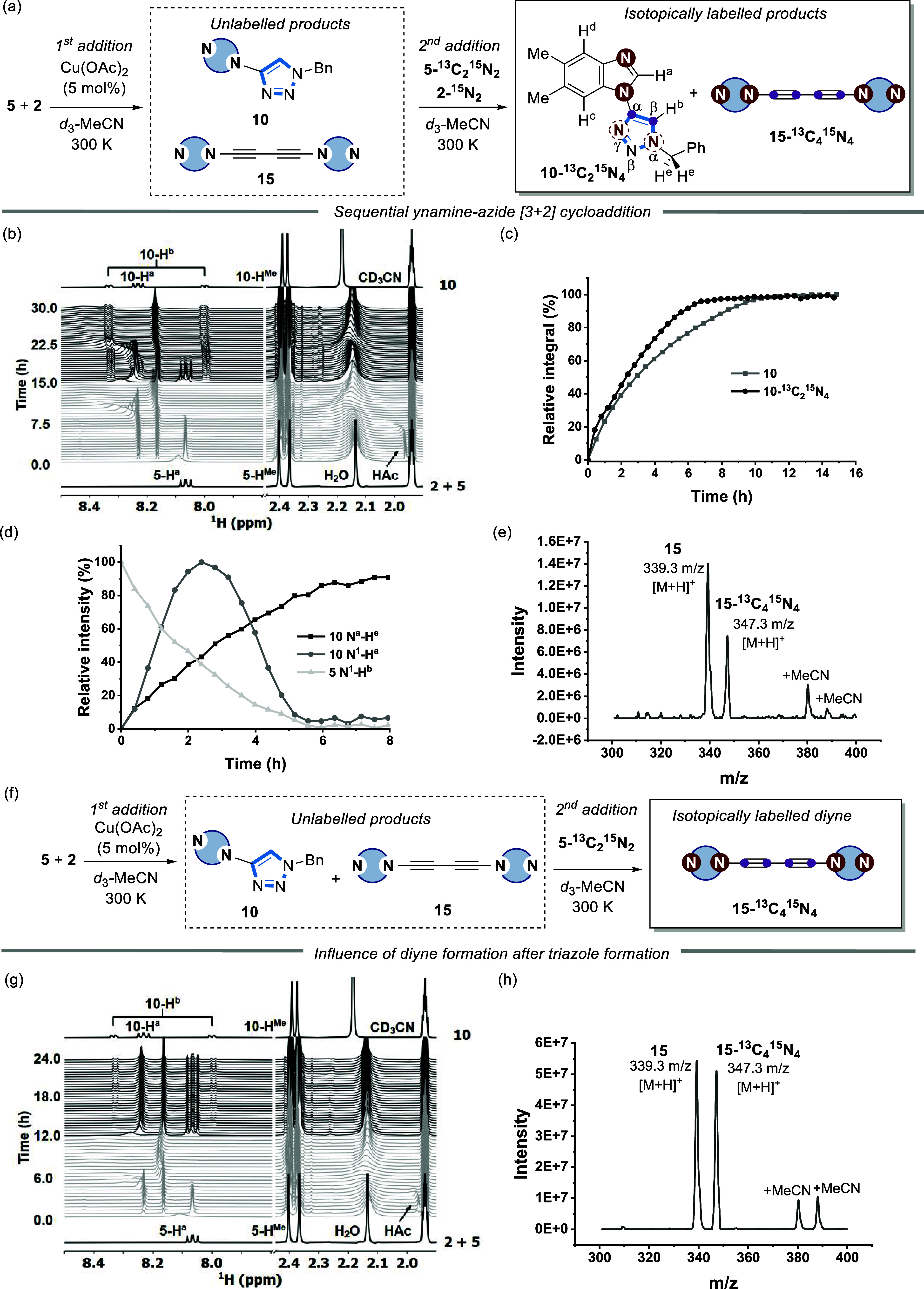
(a) (3 + 2) cycloaddition upon sequential addition of isotopically labeled **5-**^**15**^**N**_**2**_ and **5-**^**13**^**C**_**2**_**-**^**15**^**N**_**2**_; [**5-**^**15**^**N**_**2**_] and [**5-**^**13**^**C**_**2**_**-**^**15**^**N**_**2**_] = 15.5 mM; 5 mol % Cu(OAc)_2_. (b) Time course of the first (gray) and second (black) (3 + 2) cycloaddition monitored by ^1^H NMR, and (c) plotted as conversion to **10** (gray square) and **10-**^**13**^**C**_**2**_**-**^**15**^**N**_**4**_ (black circle). (d) Relative intensities of ^1^H–^15^N correlations of the second (3 + 2) cycloaddition extracted from stacked 2D ^1^H–^15^N HMBC spectra. Blacksquare = N^α^-H^e^ cross peak of **10-**^**13**^**C**_**2**_**-**^**15**^**N**_**4**_; dark graycircle = N^1^–H^a^ cross peak of **5-**^**13**^**C**_**2**_**-**^**15**^**N**_**2**_; light gray triangle = N^1^–H^b^ cross peak of **10-**^**13**^**C**_**2**_**-**^**15**^**N**_**4**_. (e) Mass spectrometric (ESI-MS) analysis of the reaction mixture after the addition of **5-**^**15**^**N**_**2**_ and **5-**^**13**^**C**_**2**_**-**^**15**^**N**_**2**_ (1 h). (f) Diyne formation upon the addition of **5-**^**13**^**C**_**2**_**-**^**15**^**N**_**2**_ after completion of the first (3 + 2) cycloaddition. (g) Time course of the first (3 + 2) cycloaddition (gray) and after the addition of **5-**^**13**^**C**_**2**_**-**^**15**^**N**_**2**_ (black) by ^1^H NMR. (h) Mass spectrometry (ESI-MS) analysis of the reaction mixture after the addition of **5-**^**13**^**C**_**2**_**-**^**15**^**N**_**2**_ (1 h). Lines through data are solely a guide to the eye.

The observation that triazole **10** forms a Cu complex by coordination at the apical site of Cu(OAc)_2_ led us to determine whether this species is catalytically competent after completion of the (3 + 2) cycloaddition. This was interrogated by first running the (3 + 2) cycloaddition with unlabeled substrates, followed by the addition of a second equivalent of isotopically labeled ynamine (**5-**^**13**^**C**_**2**_**-**^**15**^**N**_**2**_) and azide (**2-**^**15**^**N**_**2**_) after 15 h (Figure 8a). The time course of the (3 + 2) cycloaddition showed that the presence of **5-**^**13**^**C**_**2**_**-**^**15**^**N**_**2**_ resulted in a direct displacement of **10** (Figures 8b and S76). In addition, the second (3 + 2) cycloaddition resulted in a faster rate of formation of **10-**^**13**^**C**_**2**_**-**^**15**^**N**_**4**_ relative to the first, i.e., the formation of **10** (Figure 8c), which is likely due to a Cu(I) species present in a resting state with the apical face occupied by N3 coordination to **10**. Using ^1^H–^15^N HMBC, the spectral characteristics of the second (3 + 2) cycloaddition reaction were similar to the first. For example, the disappearance of the N^1^–H^b^ cross peak of **5-**^**13**^**C**_**2**_**-**^**15**^**N**_**2**_ correlated with the appearance of the N^α^-H^e^ cross peak corresponding to the formation of **10-**^**13**^**C**_**2**_**-**^**15**^**N**_**4**_ (Figure 8d). The dynamic behavior of the triazole product (**10-**^**13**^**C**_**2**_**-**^**15**^**N**_**4**_) was observed by the initial appearance of the N^1^–H^a^ which then gradually disappeared between 2 and 6 h after the addition of the isotopically labeled substrates.

A key spectroscopic difference between the first and second (3 + 2) cycloaddition was the H_2_O resonance (Figure 8b). A larger downfield shift of the H_2_O resonance (Δδ – 0.031) was detected for the first (3 + 2) cycloaddition, compared to Δδ – 0.015 for the second (Figure S79). Furthermore, the intensity of the H_2_O signal was significantly lower for the second (3 + 2) cycloaddition than the first. This could be due to H_2_O acting as a proton shuttle with acetate and the N3 benzimidazole position as **10** is formed (p*K*_a_H of *N*-methylbenzimidazole ∼4.6).^50^ We surmise that the addition of **5-**^**13**^**C**_**2**_**-**^**15**^**N**_**2**_ and **2-**^**15**^**N**_**2**_ in the second (3 + 2) cycloaddition results in a further bias in the equilibrium, reducing the magnitude of the chemical shift change of the H_2_O resonance.

We then explored the catalytic potential of the Cu species present at the end of the first (3 + 2) cycloaddition to undergo a second Glaser–Hay reaction (i.e., formation of **15-**^**13**^**C**_**4**_**-**^**15**^**N**_**4**_) after the addition of **2-**^**15**^**N**_**2**_ and **5-**^**13**^**C**_**2**_**-**^**15**^**N**_**2**_. Unexpectedly, a 1:0.5 ratio of **15** to **15-**^**13**^**C**_**4**_**-**^**15**^**N**_**4**_ (Figure 8e, further confirmed by HPLC analysis, Table S27) was present in the reaction mixture after 1 h. A 50% reduction in the amount of diyne formed.

Finally, we explored whether the amount of diyne formed (i.e., **15-**^**13**^**C**_**4**_**-**^**15**^**N**_**4**_) after completion of the first (3 + 2) cycloaddition was influenced by the presence of **2-**^**15**^**N**_**2**_ in the reaction mixture. This was interrogated by only adding **5-**^**13**^**C**_**2**_**-**^**15**^**N**_**2**_ after the formation of **10** (Figure 8f). Monitoring the reaction by ^1^H NMR showed a direct displacement of **10** from the Cu complex as observed previously (Figure 8a). Mass spectrometric analysis of the reaction mixture 1 h after addition of **5-**^**13**^**C**_**2**_**-**^**15**^**N**_**2**_ revealed a 1:1 ratio of **15**: **15-**^**13**^**C**_**4**_**-**^**15**^**N**_**4**_ was formed (Figure 8g,h). This is in contrast to the ratio of 1:0.5 formed in the presence of **2-**^**15**^**N**_**2**_ (Figure 8e, further confirmed by HPLC analysis, Table S27).

This suggests that there is ∼2.5 mol % of Cu(II) and Cu(I) present after the first (3 + 2) cycloaddition, and this dynamic mixed valent system is then influenced by the presence of both ynamine and azide substrates.

### 2.5 Proposed Reaction Mechanism of the Cu-Catalyzed Ynamine-Azide (3 + 2) Cycloaddition

Our study has highlighted how the interplay of two reactions, the Glaser–Hay alkyne coupling and CuAAC, is mediated by the choice of Cu catalyst and the alkyne substrate. Previous mechanistic studies of the Glaser–Hay reaction with conventional alkynes have shown that diyne formation results in concomitant reduction of a binuclear Cu(II) catalyst to form a mixed valent Cu(II)/Cu(I) species.^51,52^ In this study, the formation of 2.5% diyne **15** when 5 mol % Cu(OAc)_2_ is used suggests a Cu(II)-mediated C–H activation of **5**(53) followed by a single turnover of the Cu catalyst (i.e., a 2 × 1 electron reduction). This is sufficient to produce a Cu(I) species which then catalyzes the ynamine-azide (3 + 2) cycloaddition reaction to form **10**.

Zhu and co-workers identified the importance of the initial binuclear paddlewheel structure of Cu(OAc)_2_, and indeed the role played by the acetate ligands, for alkyne–azide (3 + 2) cycloadditions when a coordinating azide is used.^25,27^

Consistent with this work, we also show that the N3 position of ynamine **5** is situated in the apical position of Cu(OAc)_2_, displacing H_2_O, i.e., **17**. After complexation, a Glaser–Hay reaction produces **15** and the concomitant reduction of 50% of the available Cu catalyst. A mixed valent Cu(I)/(II) species could enter the CuAAC catalytic cycle via the N3 coordination. Disproportionation of CuOAc to a mixed valent state has also been reported, which could suggest a mixture of Cu(I)–Cu(I) and Cu(II)–Cu(I) is present^19^ and that either of these could catalyze the (3 + 2) cycloaddition.^39^

**Figure 9:**
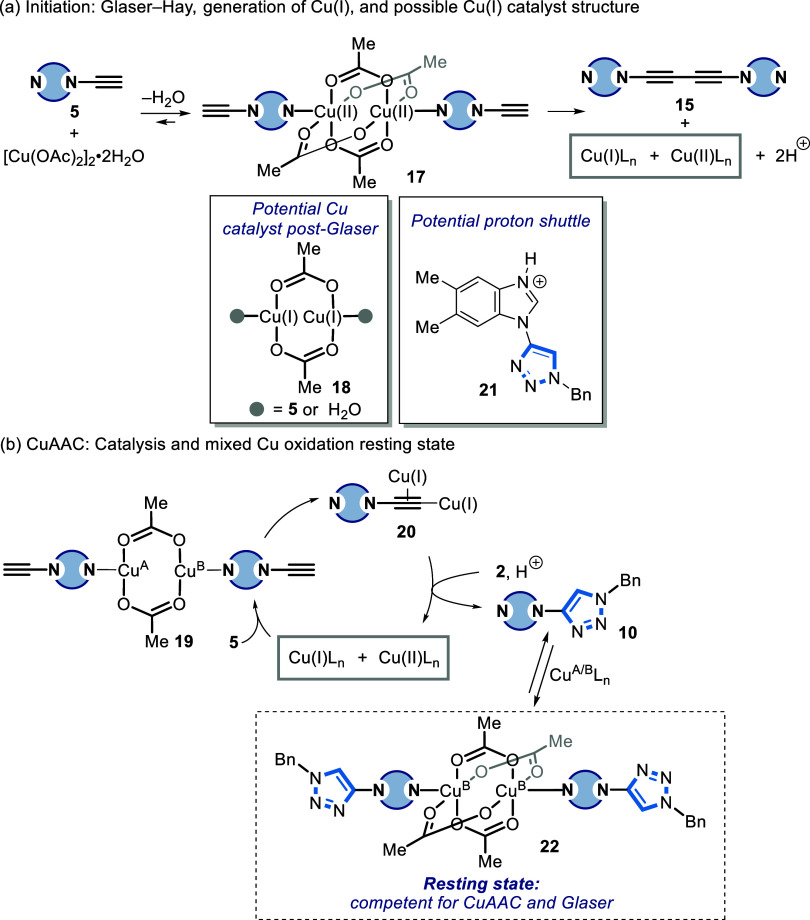
Proposed mechanism for the Cu-catalyzed ynamine-azide (3 + 2) cycloaddition. A = Cu(I), B = Cu(II) oxidation state. The net conversion of **17** to **15** plus Cu(I)/Cu(II) involves a sequence of steps not shown.

Taken collectively, we propose the following mechanism for the ynamine-azide (3 + 2) cycloaddition reaction, which deviates from a conventional CuAAC (Figure 9).^18^ N3 coordination at the apical face of Cu(OAc)_2_ by **5** forms Cu complex **17**. Homocoupling of **5** produces diyne **15** and a Cu(I) complex such as **18**(25,38,52) could enter the CuAAC catalytic cycle. N3 coordination of **5** produces complex **19** in which the Cu atoms could be mixed valent^19,25^ and rapidly forms a binuclear Cu-acetylide species **20**.^35^ Similar mixed-valence Cu(I)/Cu(II) complexes have previously been implicated as catalytically active species for CuAAC.^19,25^ Finally, azide ligation of **2** followed by protodemetalation of the Cu-triazolide forms **10**. The final protonation of the Cu-triazolide could occur via dissociated acetic acid or by a buildup of **21** as the triazole species **10** forms. When performed in MeCN, protonation of the benzimidazole N3 position of **10** (e.g., **21**; p*K*_a_H of *N*-methylbenzimidazole is 4.6)^50^ could provide a potential proton shuttle, which would likely be in equilibrium with the acetate ligands associated with Cu(OAc)_2_. These species could then facilitate protodemetalation of the Cu-triazolide.^22^

Previous, speciation studies have highlighted the potential for Cu(OAc)_2_ to act as a precatalyst and can form higher order and catalytically competent aggregates,^48^ which are highly dependent on water content and Lewis basic sites. Therefore, the proposed mechanism based on binuclear Cu complexes may be an oversimplification, with the system evolving into a series of multinuclear species as the reaction progresses.^25^ As highlighted by the changes in the chemical shift of the H_2_O (Figures 7b and 8b), H_2_O competes with the benzimidazole N3 of **5** as a ligand for the apical position of the Cu species. Upon completion of the reaction, **10** coordinates to the apical face of a Cu species post CuAAC, which could result in the formation of a postreaction resting complex such as **22**, which is catalytically competent for both a Glaser–Hay coupling and CuAAC.

## 3 Conclusions

In summary, a comprehensive investigation of the ynamine-azide (3 + 2) cycloaddition reaction has provided insight into how two Cu-catalyzed reactions work in concert to produce 1,4-triazole products. A combination of HPLC, ^1^H/^13^C/^15^N NMR, EPR and MS analyses has identified how the molecular features of the ynamine substrate manifest in its unique and divergent reactivity relative to conventional alkyne–azide (3 + 2) cycloaddition reactions. The data set emphasizes the susceptibility of the CuAAC reaction to kinetic modification based on simple changes to substrates, i.e., by the inclusion of simple coordinating groups to the alkyne substrate. These changes have clear outcomes: (i) the onset of Glaser–Hay Cu(II) reduction pathway, (ii) increases in the complexity of speciation, and, despite this, (iii) improved CuAAC kinetics. These findings will enhance our understanding of the CuAAC reaction in general and extend the application of ynamine substrates as kinetically advantageous CuAAC partners.

## Supporting Information Available

Synthetic experimental protocols, compound characterization, NMR, EPR, and HPLC assay protocols, and MS and pH experimental data and protocols (PDF)
