# Supplementary material for: Mechanistic Basis of the Cu(OAc)2 Catalyzed Azide-Ynamine (3 + 2) Cycloaddition Reaction
Source: J Am Chem Soc. 2024 May 7;146(19):13558–70. doi: 10.1021/jacs.4c03348 (PMC11099971; doi:10.1021/jacs.4c03348)
Supplement: Supplementary file 1 — ja4c03348_si_001.pdf [file ja4c03348_si_001.pdf]

Mechanistic Basis of the Cu(OAc)$_2$ Catalyzed Azide-Ynamine (3+2) Cycloaddition Reaction

Roderick P. Bunschoten,[1§] Frederik Peschke,[1§] Andrea Taladriz-Sender,[1§] Emma Alexander,[1] Matthew J. Andrews,[2] Alan R. Kennedy,[1] Neal Fazakerley,[3] Guy Lloyd Jones,[4] Allan J.B. Watson,[2*] Glenn A. Burley[1*]

[1] Department of Pure and Applied Chemistry, University of Strathclyde. Thomas Graham Building, 295 Cathedral Street, Glasgow, G1 1XL, U.K. Email: glenn.burley@strath.ac.uk

[2] EaStCHEM, School of Chemistry, University of St Andrews, Purdie Building North Haugh, St Andrews, Fife, KY16 9ST, U.K. Email: aw260@st-andrews.ac.uk

[3] GlaxoSmithKline, Medicines Research Centre, Gunnels Wood Road, Stevenage, Hertfordshire, SG1 2NY U.K.

[4] EaStCHEM. School of Chemistry, University of Edinburgh, Edinburgh EH9 3FJ, U.K.

[§] These authors contributed equally.

1 Contents

2 General Information **5**

2.1 *General* 5

2.2 *Purification of solvents* 5

2.3 *Experimental details* 5

2.4 *Purification of products* 5

2.5 *Spectroscopic analysis of products* 6

2.6 *pH measurements* 7

3 NMR experimentation and reaction monitoring **8**

3.1 *Example protocol of sample preparation for Glaser-Hay reaction monitoring* 8

3.2 *Example sample preparation for (3+2) cycloaddition reaction monitoring* 9

3.3 *Data processing protocol* 10

3.4 *Compound NMR references in CD_3CN acquired at 300 K* 11

3.5 *Hydrogen-deuterium exchange (HDE)* 16

3.6 *Intermolecular hydrogen-deuterium exchange (HDE) of compound 5* 21

3.7 *(3+2) Cycloaddition reaction screening in 9:1 CD_3CN / D_2O* 23

3.8 *^{1}H analysis of compound 14 in presence of $Cu(OAc)_2{\cdot}H_2O$* 27

3.9 *^{1}H-^{15}N HMBC analysis of compound 14-$^{15}N_2$ in presence of $Cu(OAc)_2{\cdot}H_2O$* 28

3.10 *^{13}C Signal to noise ratio for compound 14-$^{13}C_2$-$^{15}N_2$* 31

3.11 *^{13}C NMR analysis of compound 14-$^{13}C_2$-$^{15}N_2$ in presence of $Cu(OAc)_2{\cdot}H_2O$* 31

3.12 *^{1}H analysis of compound 14 in presence of $[(CH_3CN)_4Cu]PF_6$* 33

3.13 *^{1}H-^{15}N HMBC and ^{13}C Analysis of compound 14-$^{15}N_2$ in presence of 5 mol% $[(CH_3CN)_4Cu]PF_6$* 34

3.14 *VT-NMR analysis of compound 14-$^{13}C_2$-$^{15}N_2$ in presence of 1.0 equiv $[(CH_3CN)_4Cu]PF_6$* 36

3.15 *^{1}H-NMR analysis of compound 5 in presence of $Cu(OAc)_2{\cdot}H_2O$* 40

3.16 *^{1}H-^{15}N HMBC NMR analysis of compound 5-$^{15}N_2$ in presence of 5 mol% $Cu(OAc)_2$* 44

3.17 *^{13}C and ^{1}H VT-NMR analysis of compound 5-$^{13}C_2$-$^{15}N_2$ in presence of 5 mol% $Cu(OAc)_2{\cdot}H_2O$* 48

3.18 *^{1}H-NMR Analysis of compound 5 in presence of 5 mol% $[(CH_3CN)_4Cu]PF_6$* 51

3.19 VT-NMR analysis of compound 5-$^{13}C_2$-$^{15}N_2$ in presence of[$(CH_3CN)_4Cu$]PF_6 53

3.20 ^{1}H and ^{13}C NMR analysis of compound S10 in presence of 1.00 equiv [$(CH_3CN)_4Cu$]PF_6 61

3.21 ^{1}H-^{15}N HMBC VT-NMR analysis of compound 8 in presence of 1.00 equiv [$(CH_3CN)_4Cu$]PF_6 63

3.22 Preliminary ^{1}H NMR monitoring of the ynamine-azide (3+2) cycloaddition reaction in CD_3CN 64

3.23 ^{1}H and ^{1}H-^{15}N HMBC monitoring of the ynamine-azide (3+2) cycloaddition reaction in CD_3CN 66

3.24 ^{13}C NMR monitoring of the ynamine-azide (3+2) cycloaddition reaction in CD_3CN 74

3.25 Monitoring the sequential addition of substrates post completed (3+2) cycloaddition reaction in CD_3CN 77

3.26 Monitoring the sequential addition of compound 5-$^{13}C_2$-$^{15}N_2$ post completed (3+2) cycloaddition reaction in CD_3CN 82

3.27 Monitoring the sequential addition of compound 2 post completed (3+2) cycloaddition in CD_3CN 86

4 EPR 91

4.1 EPR spectrum of compound 14 in presence of $Cu(OAc)_2 \cdot H_2O$ 91

4.2 EPR monitoring compound 5 in presence of $Cu(OAc)_2 \cdot H_2O$ 92

4.3 EPR monitoring Ynamine-Azide (3+2) Cycloaddition in CH_3CN 93

4.4 EPR monitoring Ynamine-Azide (3+2) Cycloaddition in CH_3CN in the same conditions as the NMR experiments 94

4.5 EPR control experiments 95

4.5.1 EPR spectra of reagents 95

4.5.2 EPR experiment monitoring the effect of triazole 10 and Cu(I) in the EPR signal during the time course experiment over 6 h 96

4.5.3 EPR experiment monitoring the effect of the triazole 10 and Cu(II) in the S/N during the time course experiment over 6 h 97

5 Single Crystal X-ray Diffraction 99

6 Analytical RP-HPLC 101

6.1 Calibration 101

6.2 Glaser-Hay reaction monitoring procedure A 103

6.3 (3+2) Cycloaddition reaction monitoring procedure B 104

6.4 Stoichiometry and the formation of compound 15 104

6.5 Copper loading and compound 15 formation in MeCN 105

6.6 Effect of water on compound 15 formation 106

6.7 Monitoring the ynamine-azide (2+3) cycloaddition and the formation of compound 10 and 15 in MeCN 107

6.8 Monitoring the sequential addition of substrates post completed (3+2) cycloaddition reaction in CD_3CN 109

7 MS investigation of potential copper Ynamine-TIPS complexes 113

8 pH measurements of HDE experiments 115

9 CuAAC in THF 116

10 Synthetic procedures 117

11 References 136

12 NMR, FT-IR, and MS spectra 137

2 General Information

2.1 General

All reagents and solvents were obtained from commercial suppliers and were used without further purification unless otherwise stated. Purification was carried out according to standard laboratory methods. Starting materials were purchase from commercial suppliers and used without further purification unless otherwise stated. Isotopically labelled (Triisopropylsilyl) [1,2-^{13}C] acetylene (912441), Sodium azide [1-^{15}N] (609374), [^{15}N] Ammonium Hydroxide (488011) and Sodium [^{15}N] Nitrate (364606) were purchased from Merck, Sigma-Aldrich.

Naturally abundant 1-ethynyl-5,6-dimethyl-1*H*-benzo[*d*]imidazole was prepared according to literature procedures.[1]

2.2 Purification of solvents

Dry solvents for reactions were either obtained from a PureSolv SPS-400-5 solvent purification System or were purchased from Sigma-Aldrich and stored under nitrogen. Dichloromethane, chloroform, methanol, ethyl acetate, and petroleum ether (40 – 60 °C) for purification purposes were used as obtained from suppliers, without further purification.

2.3 Experimental details

Reactions were carried out using conventional glassware for the preparation of starting materials. Microwave reactions were carried out in capped 2.00 – 5.00 mL microwave vials purchased from Biotage®. Microwave reactions were carried out at elevated temperatures using a Biotage® Initiator + equipped with a Robot Eight microwave system.

2.4 Purification of products

Thin layer chromatography was carried out using Merck silica plates coated with fluorescent indicator UV254 and were analyzed under both 254 nm and 375 nm UV light or developed using potassium permanganate solution. Normal phase flash chromatography was carried out using 60Å 40-63 µm silica gel from Fluorochem. Automatic purification was carried out on an Interchim PuriFLASH XS52Plus system, using Silicycle 230-400 mesh 40-63 µm silica columns of various sizes. Semi-preparative reversed-phase HPLC purification was carried out on a Kinetex 5u.C18 100A, 150 × 21.2 mm column using a DIONEX 3000 series HPLC system equipped with a VWD3400 variable wavelength detector. Preparative purifications of small molecules were performed using a gradient B (Table S1, solvent A: water, solvent B: acetonitrile), with a flow rate of 12.0 mL/min. The absorbance UV-active material was detected at 254 nm. Analytical reversed-phase HPLC (RP-HPLC) was carried out on a Shimadzu

Prominence instrument equipped with a PDA Detector scanning from 190 to 600 nm using a using a Kinetex® C18 100 Å, 50 x 4.6 mm, 2.6 µm at a column oven temperature of 40 °C, using a gradient B as seen in the table below (solvent A: 0.1% v/v TFA in water, solvent B: 0.1% v/v TFA in acetonitrile), with a flow rate of 1.5 mL/min. The absorbance UV-active material was detected at 254 nm.

Table S1. Gradient profile table

Analytical/ Semi-preparative RP-HPLC		
Time (min)	%A	%B
0.0	95.0	5.00
5.8	40.0	60.0
6.0	5.00	95.0
8.0	5.00	95.0
8.2	95.0	5.00
10.0	95.0	5.00

2.5 Spectroscopic analysis of products

NMR spectroscopy was carried out using a AVIIIHD Nanobay400 NMR, Bruker AVIII HD 500 MHz, AVIII400 NMR Spectrometer with Cryoprobe, or an AVII+ 600 MHz spectrometer. All chemical shifts (δ) are reported in parts per million (ppm) and were referenced to the solvents used $SO(CD_3)_2$ 2.50 ppm (^1H) and 39.52 ppm (^{13}C), CD_3CN 1.94 ppm (^1H), 118.26 ppm (^{13}C), 245.00 ppm (^{15}N), CD_3OD referenced at 3.31 ppm (^1H) and 49.0 ppm (^{13}C), $CDCl_3$ 7.26 (^1H) and 77.2 ppm (^{13}C) (as reported in https://pubs.acs.org/doi/10.1021/jo971176v). Coupling constants are quoted in hertz (Hz). Abbreviations for splitting patterns are s (singlet), d (doublet), t (triplet), q (quartet) and m (multiplet). All NMR data was processed using Mestrelab Research S.L. Mnova 14.2.1-27684. 1,3,5-trimethoxy benzene was used as an internal standard where annotated. Proton, carbon, and nitrogen chemical shifts were assigned using proton (^1H), carbon (^{13}C) or nitrogen (^{15}N), Heteronuclear Single Quantum Coherence (HSQC), Heteronuclear Multiple-Bond Correlation Spectroscopy (HMBC) and Correlation Spectroscopy (COSY). T_1 determination experiments were performed on a Bruker/OXFORD Avance 400 MHz spectrometer. ^1H T_1 relaxation times, the time when 63% magnetization along the z-axis is restored of all reagents were determined using standard saturation recovery experiments at a concentration of 62.0 mM. The recovery time in the experiment was varied

from 1 to 15 seconds. The optimal recycle delay was determined by a Three Parameter Exponential Fit.

High-resolution mass spectra were recorded on a Bruker microTOF II mass spectrometer at the SIRCAMs facility at the University of Edinburgh or on a ThermoScientific Orbitrap Fusion Lumos with Ultimate 3000 Nano LC spectrometer at the University of St Andrews or a ThermoScientific Exactive™ Plus Orbitrap Mass Spectrometer.

Fourier Transformed Infra-Red (FTIR) spectra were obtained on a Shimadzu IR Affinity-1 instrument. Only major absorbance bands are reported.

Gas chromatography-mass spectrometry was performed on an Agilent Technologies 7890A GC-system fitted with an Agilent Technologies 7693 Autosampler and an Agilent Technologies 5975C inert XL/EI/CI MSD with a triple-axis detector.

2.6 pH measurements

pH measurements were performed using a calibrated Orion Star™ A211 Benchtop pH Meter equipped with a Hanna® Instruments HI-1093B pH electrode.

3 NMR experimentation and reaction monitoring

3.1 Example protocol of sample preparation for Glaser-Hay reaction monitoring

The following protocol describes a representative example of a time course experiment that was used to monitor Glaser-Hay reactions. Stock solutions were prepared in the acetonitrile (Table S2):

Table S2. Stock solution preparation

Stock	Analyte	Catalyst
Species	Ynamine **5**	$Cu(OAc)_2{\cdot}H_2O$
MW ($g{\cdot}mol^{-1}$)	170.22	199.65
Concentration (mM)	77.5	15.5
Volume (mL)	1.00	2.00
Mass (mg)	13.2	6.20

Reference/shimming sample: 400 µL of analyte stock were transferred to a clean and dry NMR tube. An additional 100 µL of CD_3CN were added, reaching a final concentration of 62.0 mM. The sample was placed in the magnet, locked, tuned, shimmed, and a proton spectrum was acquired to study the quality of shims. When VT was required, the probe was chilled or heated to the desired temperature whilst the reference sample was in the probe.

Reaction sample: after the probe was locked and shimmed onto the reference sample, 400 µL of analyte stock was transferred to a clean and dry NMR tube and 100 µL of catalyst stock was added, reaching a final concentration of 62.0 mM analyte. The sample was shaken vigorously and immediately lowered into the magnet to start the time course experiment. For cold VT experiments, the analyte stock and catalyst stock were separately chilled to the desired temperature prior to mixing. Time course experiments were recorded using 1D spectra and zg30 pulse or 2D experiment programs that were automated utilising the *multi_zgvd2* command with a fixed delay to allow for a *topshim_1dfast* shim after each acquisition. This line of code was added to the standard *multi_zgvd2* AU program.

When an internal standard was required, stock solutions were prepared with 1,3,5-trimetoxybenzene (Table S3).

Table S3. Stock solution preparation with an internal standard.

Stock	Analyte/IS		Catalyst
Species	Ynamine **5**	1,3,5-TMB	$Cu(OAc)_2 \cdot H_2O$
MW ($g \cdot mol^{-1}$)	170.22	168.19	199.65
Concentration (mM)	77.5	7.75	15.5
Volume (mL)	6.60		2.00
Mass (mg)	87.0	8.60	6.20

When required, catalyst solutions were diluted to reach representative concentrations (Table S4).

Table S4. Catalyst stock solution dilution table.

Rep.% cat.	V solvent (µL)	V cat. Stock (µL)	Cat. (mM)
5.00	0.00	2000	15.50
1.00	400	100	3.10
0.50	250	0.00	1.55
0.25	250	0.00	0.78
0.125	250	0.00	0.39

3.2 Example sample preparation for (3+2) cycloaddition reaction monitoring

The following protocol describes an example of a time course experiment that was used to monitor (3+2) cycloaddition reactions. Stock solutions were prepared in the solvent required for the experiment (Table S5).

Table S5. Stock solution preparation.

Stock	Analyte		Catalyst
Species	Ynamine **5**	Azide **2**	$Cu(OAc)_2 \cdot H_2O$
MW ($g \cdot mol^{-1}$)	170.22	130.15	199.65
Concentration (mM)	77.5	77.5	15.5
Stock volume (mL)	1.00		2.00
Mass (mg)	13.2	10.3	6.20
Density ($g \cdot mL^{-1}$)		1.07	
Volume (µL)		9.63	

Reference/shimming sample: 400 µL of analyte stock was transferred to a clean and dry NMR tube, to this was added an additional 100 µL deuterated solvent, reaching a concentration of 62.0 mM. The sample was placed in the magnet, tuned, shimmed, locked and a proton spectrum was acquired to study the quality of shims. When VT was required, the probe was chilled or heated to the desired temperature whilst the reference sample was in the probe.

Reaction sample: after the probe was locked and shimmed onto the reference sample, 400 µL of analyte stock was transferred to a clean and dry NMR tube, to this was added 100 µL catalyst stock, reaching a concentration of 62.0 mM analyte. All copper sources were soluble in the reaction conditions except for CuOAc, which was used as a suspension. The reactions were performed under atmospheric conditions. The sample was shaken vigorously and immediately lowered into the magnet to start the time course experiment. For cold VT experiments, the analyte stock and catalyst stock were separately chilled to the desired temperature prior to mixing. Time course experiments were recorded using 1D spectra and zg30 pulse or 2D experiment programs that were automated utilising the *multi_zgvd2* command with a fixed delay to allow for a *topshim_1dfast* shim after each acquisition. This line of code was added to the standard *multi_zgvd2* AU program.

3.3 Data processing protocol

Stacking 1D spectra: spectra were individually referenced to their respective solvent. Phase correction was performed automatically and adjusted manually where appropriate. The baseline was automatically adjusted using the Whittaker Smoother. Spectra of interest were then selected, stacked, and analysed.

Integrating stacked 1D spectra: graphs were generated with MNova using the *Advanced > Data Analysis > Create > Integrals Graph* tool. The integrated area was adjusted for drifting. The generated report table was then copied to Microsoft Excel for further processing. For Δppm graphs, the *Advanced > Data Analysis > Create > Alignment Shifts Graph* tool was used. The acquired data was then further processed in Microsoft Excel or Origin.

Processing 2D spectra: Corresponding 1D spectra were processed as mentioned above and loaded into the 2D experiment using MNova – 2D correlations were then graphically referenced to 1D signals. Where appropriate, 2D data was stacked and integrated. The acquired data was then further processed in Microsoft Excel or Origin.

3.4 Compound NMR references in CD_3CN acquired at 300 K

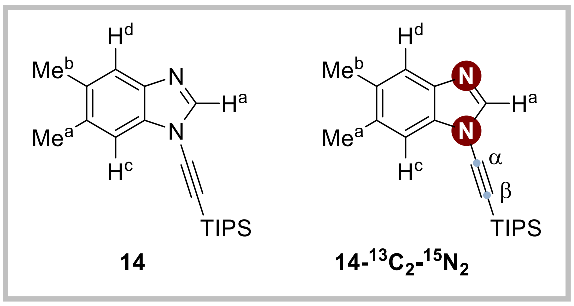

Scheme S1. Two isotopic derivatives of the aromatic ynamine-TIPS. Annotated for NMR.

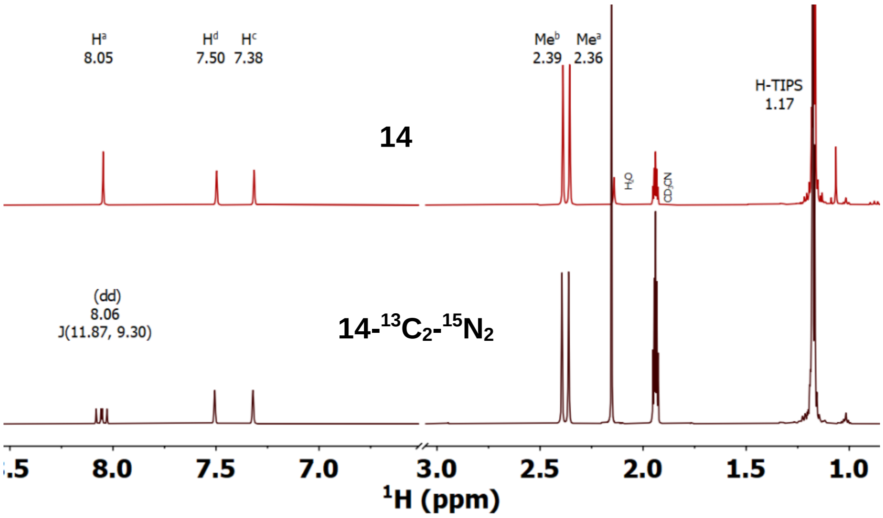

Figure S1. ^{1}H NMR analysis of the two isotopic derivatives **14** and **14-$^{13}C_2$-$^{15}N_2$** of the aromatic ynamine-TIPS. Acquired on a AVIIIHD Nanobay400 NMR spectrometer. Referenced to CD_3CN, ^{1}H = 1.94 ppm.

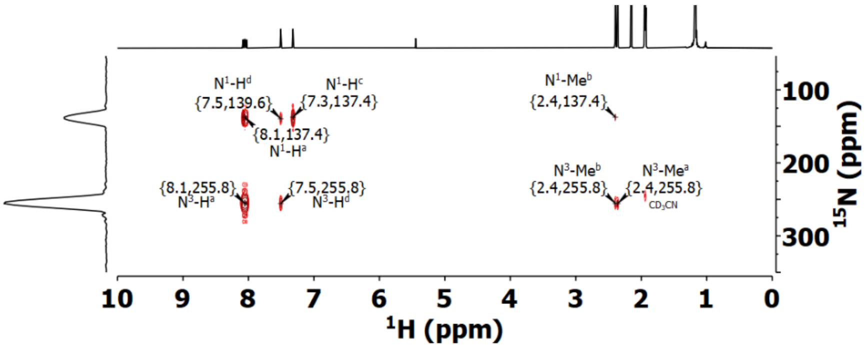

Figure S2. ^{1}H-^{15}N HMBC NMR analysis of isotopic derivative **14-$^{13}C_2$-$^{15}N_2$** of the aromatic ynamine-TIPS. Acquired on a AVIIIHD Nanobay400 NMR spectrometer. Referenced to CD_3CN, ^{1}H = 1.94 ppm, ^{15}N = 245 ppm.

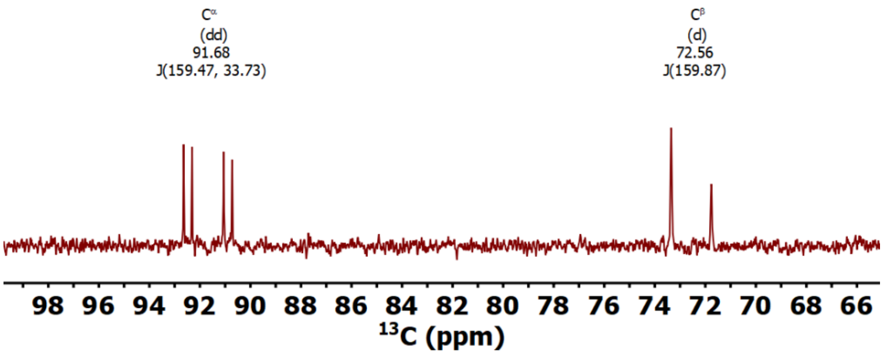

Figure S3. ^{13}C NMR analysis of isotopic derivative **14-$^{13}C_2{}^{15}N_2$** of the aromatic ynamine-TIPS. Acquired on a AVIIIHD Nanobay400 NMR spectrometer. Referenced to CD_3CN, ^{13}C = 118.26 ppm.

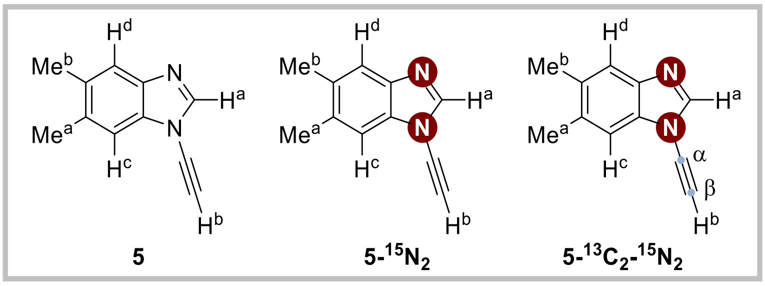

Scheme S2. Three isotopic derivatives of the aromatic ynamine. Annotated for NMR**.**

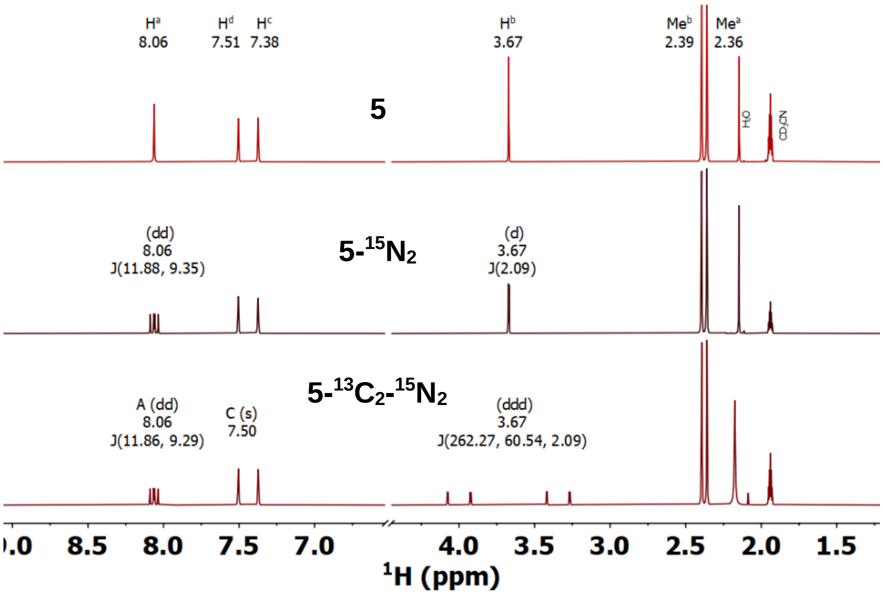

Figure S4. ^{1}H NMR analysis of the three isotopic derivatives **5**, **5-$^{15}N_2$** and **5-$^{13}C_2$-$^{15}N_2$** of the aromatic ynamine. Acquired on a AVIIIHD Nanobay400 NMR spectrometer. Referenced to CD_3CN, ^{1}H = 1.94 ppm.

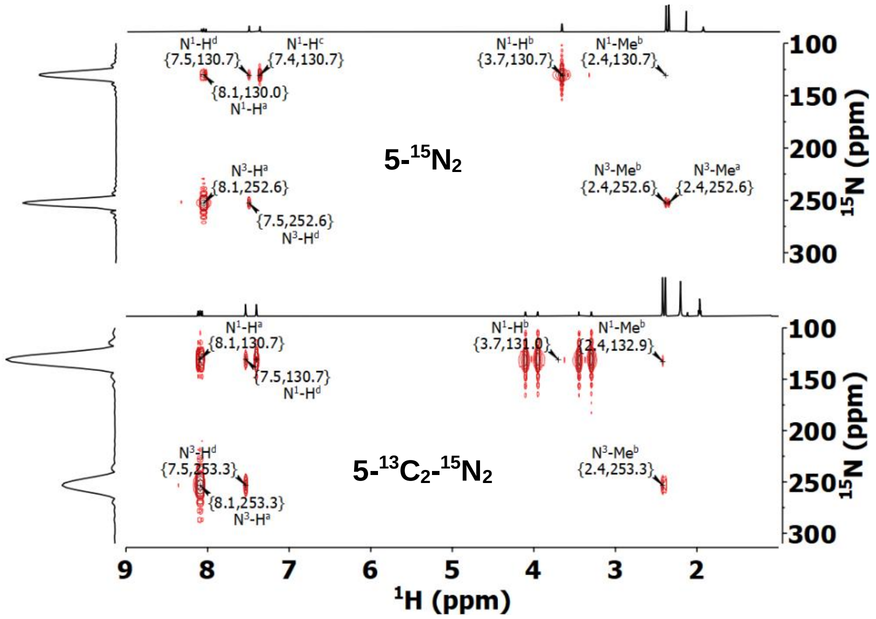

Figure S5. ^{1}H-^{15}N HMBC NMR analysis of the two isotopic derivatives **5-$^{15}N_2$** and **5-$^{13}C_2$-$^{15}N_2$** of the aromatic ynamine. Acquired on a AVIIIHD Nanobay400 NMR spectrometer. Referenced to CD_3CN, ^{1}H = 1.94 ppm, ^{15}N = 245 ppm.

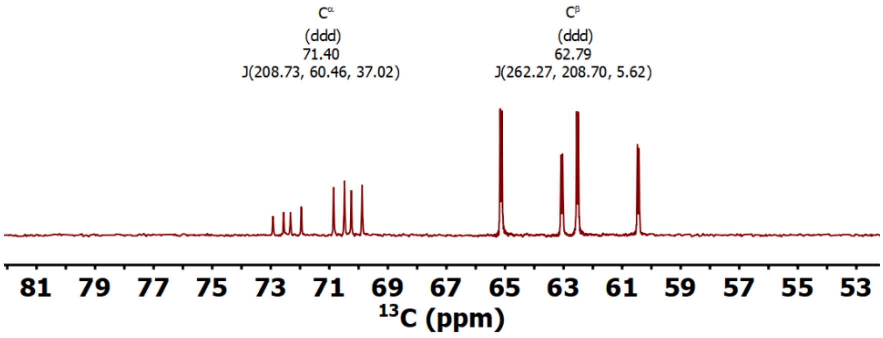

Figure S6. ^{13}C NMR analysis of isotopic derivative **5-$^{13}C_2$-$^{15}N_2$** of the aromatic ynamine. Acquired on a AVIIIHD Nanobay400 NMR spectrometer. Referenced to CD_3CN, ^{13}C = 118.26 ppm.

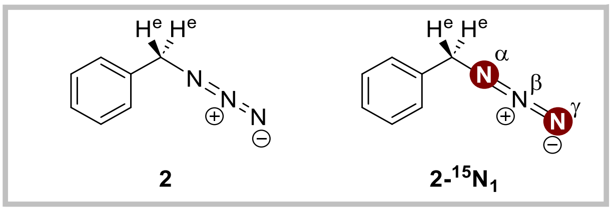

Scheme S3. Two isotopic derivatives of benzyl azide. Annotated for NMR.

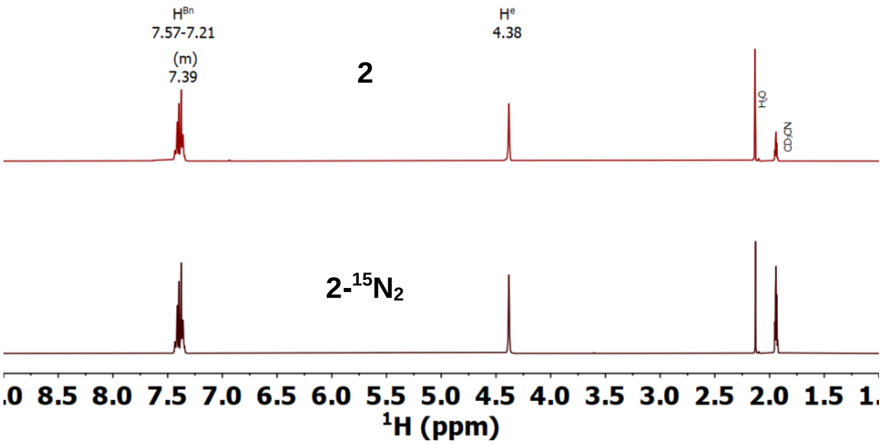

Figure S7. ^{1}H NMR analysis of the two isotopic derivatives **2**, and **2-^{15}N$_1$** of benzyl azide. Acquired on a AVIIIHD Nanobay400 NMR spectrometer. Referenced to CD_3CN, ^{1}H = 1.94 ppm.

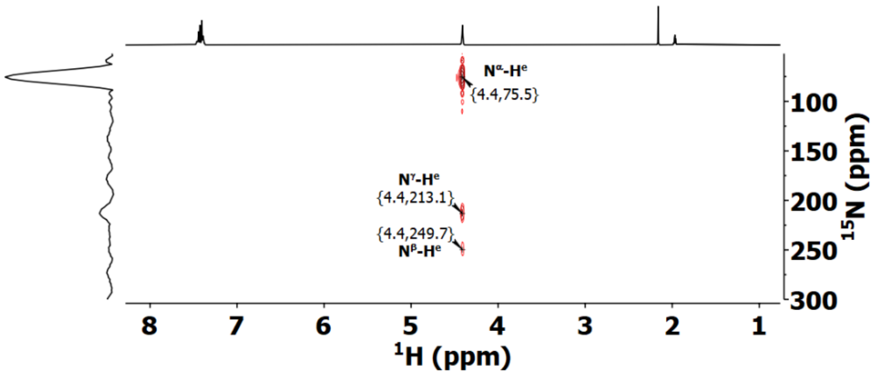

Figure S8. ^{1}H-^{15}N HMBC NMR analysis of the isotopic derivative **2-^{15}N$_1$** of benzyl azide. Acquired on a AVIIIHD Nanobay400 NMR spectrometer. Referenced to CD_3CN, ^{1}H = 1.94 ppm, ^{15}N = 245 ppm.

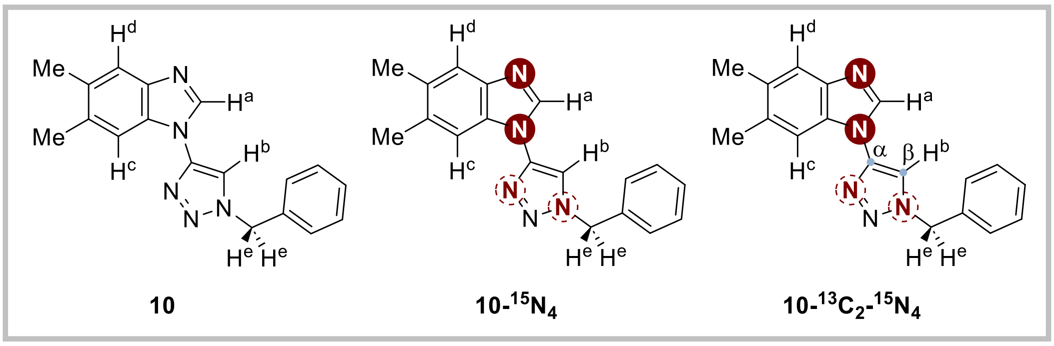

Scheme S4. Three isotopic derivatives of the aromatic ynamine triazole (3+2) cycloaddition reaction product. Annotated for NMR.

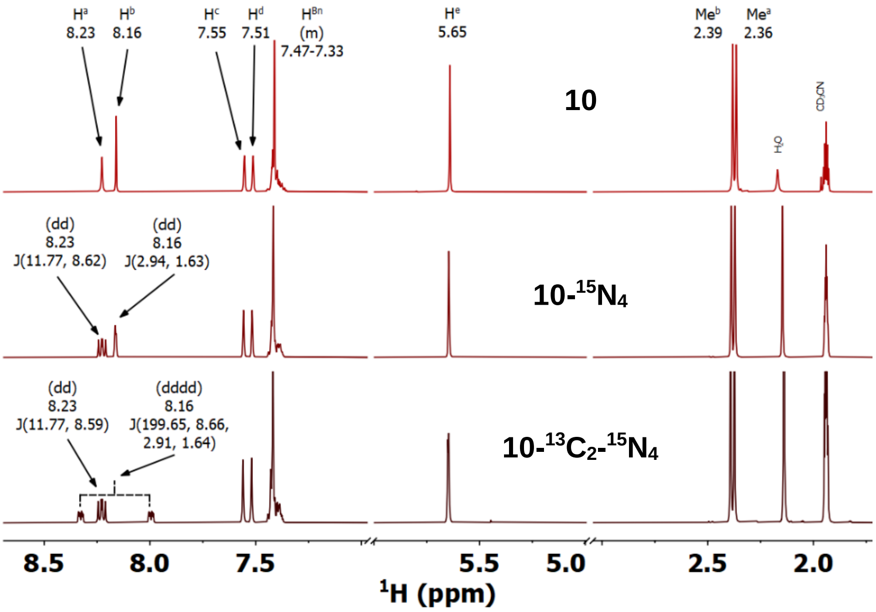

Figure S9. ^{1}H NMR analysis of the three isotopic triazole derivatives **10**, **10-^{15}N$_4$** and **10-^{13}C$_2$-^{15}N$_4$**. The ^{1}H spectrum for **10** was acquired on a AVIIIHD Nanobay400 NMR spectrometer. The ^{1}H spectra for **10-^{15}N$_4$** and **10-^{13}C$_2$-^{15}N$_4$** were acquired on an AVII+ 600MHz spectrometer. Referenced to CD$_3$CN, ^{1}H = 1.94 ppm.

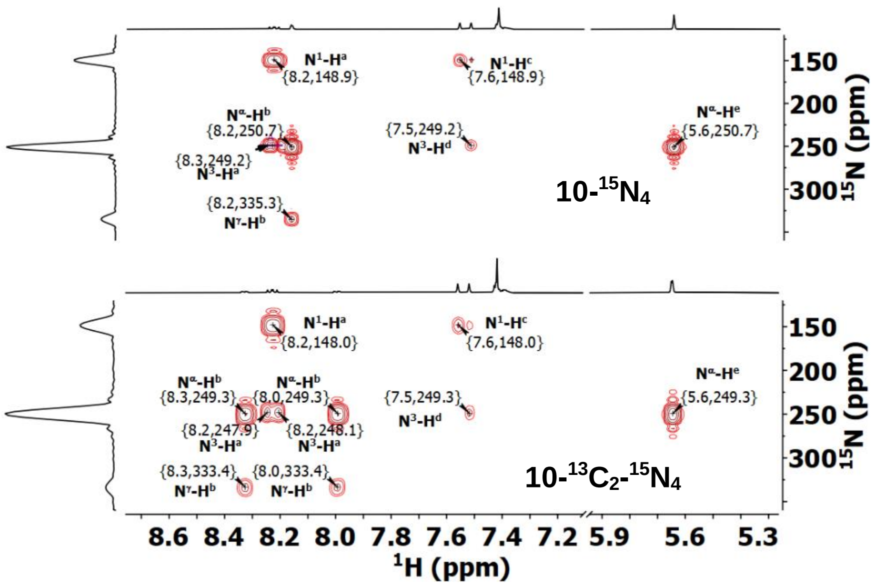

Figure S10. ^{1}H-^{15}N HMBC NMR analysis of isotopic derivatives **10-^{15}N$_3$** and **10-^{13}C$_2$-^{15}N$_3$** of the triazole. Acquired on an AVII+ 600MHz spectrometer. Referenced to CD$_3$CN, ^{1}H = 1.94 ppm, ^{15}N = 245 ppm.

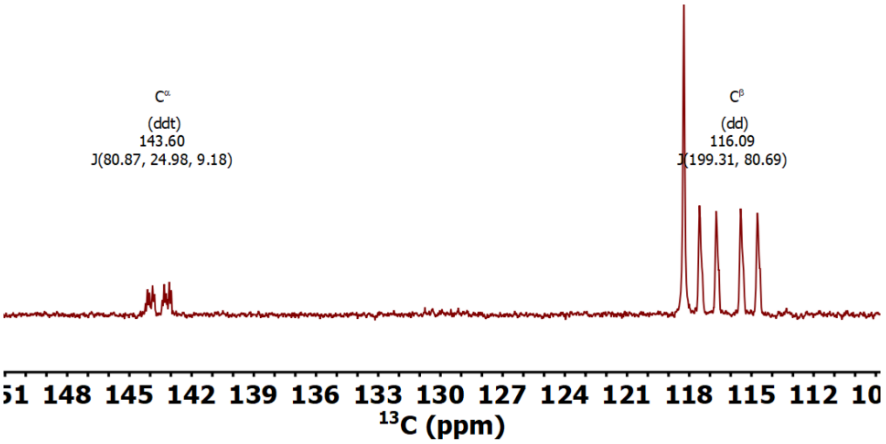

Figure S11. ^{13}C NMR analysis of isotopic derivative **10-$^{13}C_2$-$^{15}N_4$** of the triazole. Acquired on a AVIIIHD Nanobay400 NMR spectrometer. Referenced to CD_3CN, ^{13}C = 118.26 ppm.

3.5 Hydrogen-deuterium exchange (HDE)

The HDE capacity for various alkynes was investigated in a mixture of 9:1 CD_3CN / D_2O at a concentration of 62.0 mM. 69 mM alkyne stock solutions were prepared in CD_3CN, and 31 mM "catalyst'' stock solutions were prepared in D_2O. The ''catalyst'' stock solution was prepared next to the NMR magnet and used immediately to prevent potential oxidation. NMR samples were prepared by transferring 450 µL alkyne stock to a clean NMR tube, after which 50 µL of the ''catalyst'' stock was added, reacting an alkyne concentration of 62.0 mM and 5.00 mol% "catalyst". The addition of NaOAc is 5.00 mol% or 10.0 mol% (i.e., 2 NaOAc). After the addition of the ''catalyst'' solution, ^{1}H-spectra were acquired immediately (acquired on a AVIIIHD Nanobay400 NMR spectrometer. Parameters: TE = 300 K, NS = 16, D1 = 5 sec, RG = 211.5). When the reaction rate was fast, samples were measured manually and individually, using the *multi_zgvd2* command that included a *topshim_1dfast* shim after each acquisition. Experiments had a fixed delay of 300 seconds, n = 20 to 35 to monitor every 5 minutes over 3 hours. Slower experiments were combined on a sample carousel and measured automatically using IconNMR Automation Software at a regular interval. Deuteration was calculated by monitoring and integrating the alkyne proton, normalised against non-exchangeable protons (CH_3 protons).

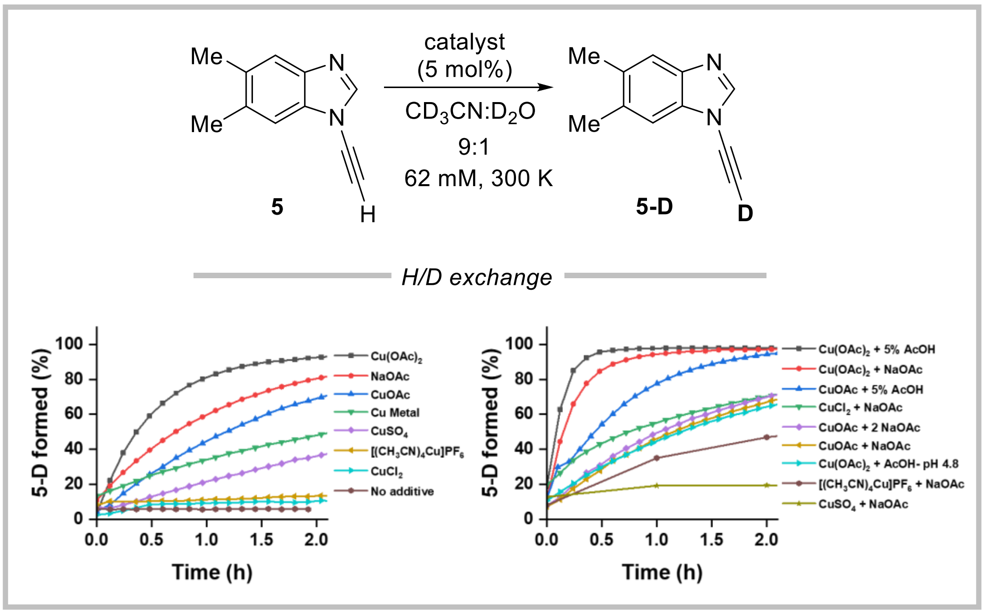

Figure S12. HDE in 9:1 CD_3CN / D_2O forming deuterated **5-D**. HDE time course of **5** to form **5-D** as monitored by ^1H NMR spectroscopy. The addition of NaOAc is 5.00 mol% or 10.0 mol% (i.e. 2 NaOAc). Deuteration was calculated by monitoring and integrating the alkyne proton, normalised against non-exchangeable protons (CH_3).

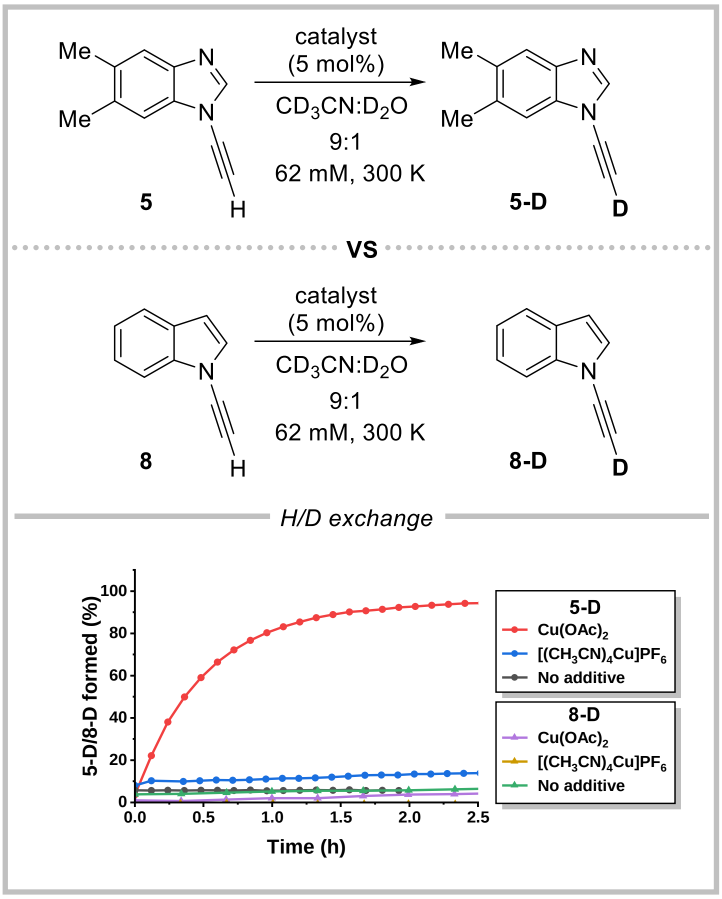

Figure S13. HDE in 9:1 CD_3CN / D_2O forming deuterated indole **8-D**, compared to ynamine **5-D**. HDE time course as monitored by ^1H NMR spectroscopy. Deuteration was calculated by monitoring and integrating the alkyne proton, normalised against non-exchangeable protons.

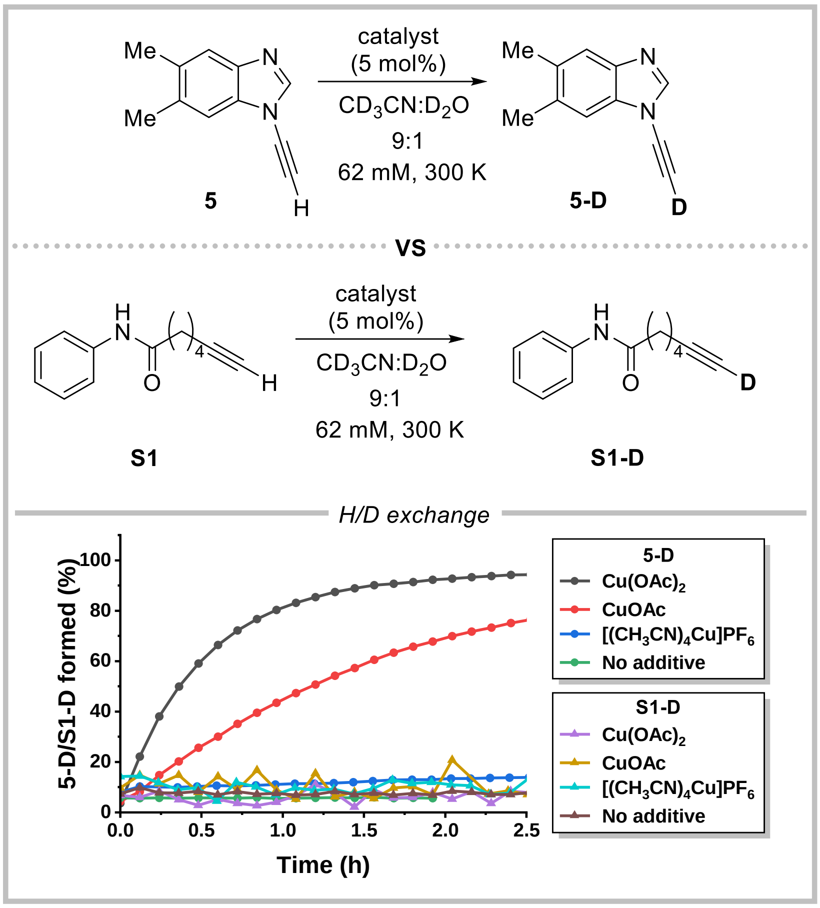

Figure S14. HDE in 9:1 CD_3CN / D_2O forming deuterated alkyne **S1-D**, compared to ynamine **5-D**. HDE time course as monitored by ^{1}H NMR spectroscopy. Deuteration was calculated by monitoring and integrating the alkyne proton, normalised against non-exchangeable protons.

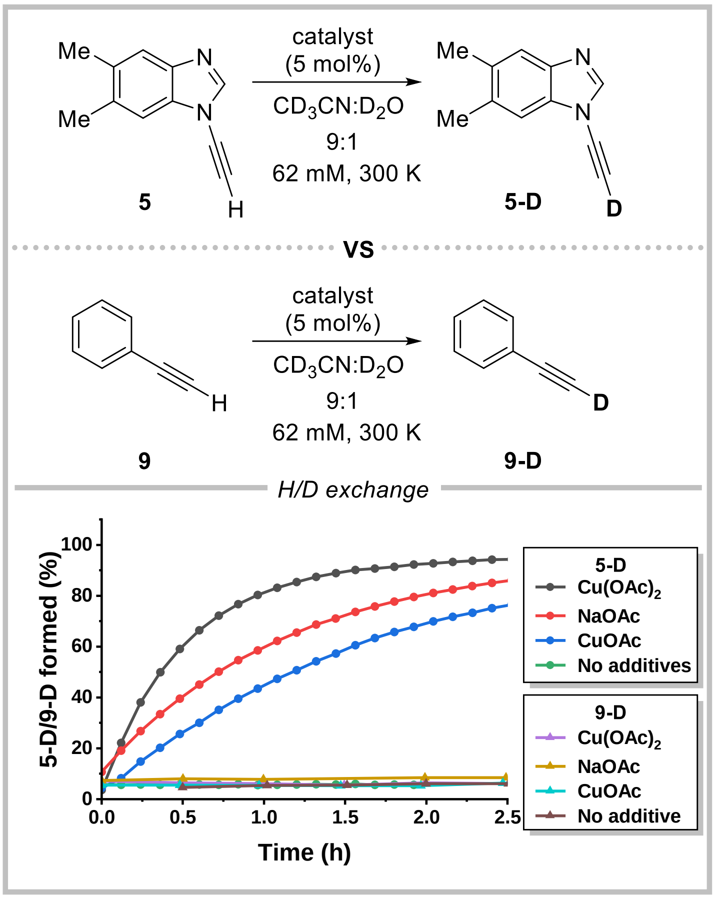

Figure S15. HDE in 9:1 CD_3CN / D_2O forming deuterated phenyl-acetylene **9-D**, compared to ynamine **5-D**. HDE time course as monitored by ^1H NMR spectroscopy. Deuteration was calculated by monitoring and integrating the alkyne proton, normalised against non-exchangeable protons.

3.6 Intermolecular hydrogen-deuterium exchange (HDE) of compound 5

The intramolecular HDE capacity of the aromatic ynamine was investigated in CD_3CN by NMR. Labelled ynamine **5-$^{13}C_2$-$^{15}N_2$** has a terminal alkyne-proton H^b with a distinct *ddd* splitting pattern of $J_{H\text{-}C\beta}$ = 262.3 Hz, $J_{H\text{-}C\alpha}$ = 60.5 Hz and $J_{H\text{-}N1}$ = 2.1 Hz at δ_{1H} = 3.67 ppm. This property is useful when compared to the H^b singlet resonance of **5** at δ_{1H} = 3.67 ppm that would form with the protonation of deuterated ynamine **5-D**. Thus when **5-$^{13}C_2$-$^{15}N_2$** and **5-D** would be mixed, a H^b integral decrease for **5-$^{13}C_2$-$^{15}N_2$**, and an increase in the integral for (protonated) H^b **5-D** would be the result of an intramolecular HDE. 250 µL 31 mM **5-$^{13}C_2$-$^{15}N_2$** (1.30 mg, 7.50 µmol, 174.18 g·mol^{-1} in 250 µL CD_3CN) was mixed with 250 µL 31 mM **5-D** (1.30 mg, 7.50 µmol, 171.22 g·mol^{-1} in 250 µL CD_3CN) in a clean and dry NMR tube, reaching a final concentration of 15.5 mM. The tube was immediately capped, shaken vigorously, and lowered down the probe. ^1H spectra (acquired on a AVIIIHD Nanobay400 NMR spectrometer. Parameters: TE = 300 K, NS = 32, D1 = 1 sec, RG = 211.5) were acquired at 300 K over 1.5 hours in which no exchange was detected (Figure S16).

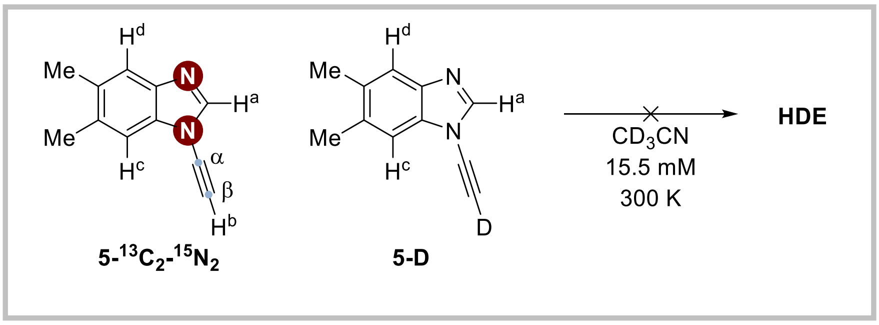

Scheme S5. Monitoring the HDE between two ynamine molecules **5-$^{13}C_2$-$^{15}N_2$** and **5-D**.

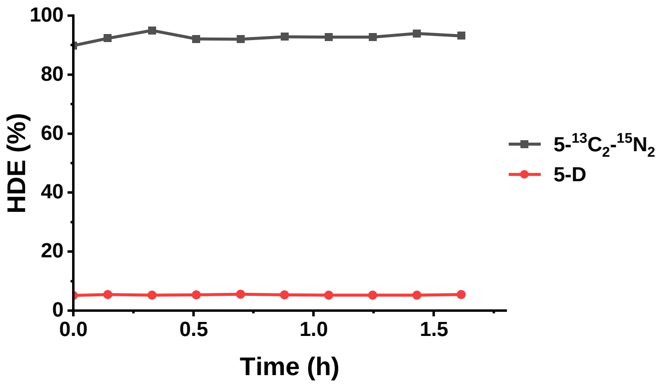

Figure S16. Monitoring the intramolecular HDE (H^b) between ynamine **5-$^{13}C_2$-$^{15}N_2$** and **5-D** in CD_3CN at 15.5 mM and 300 K.

It was postulated that $Cu(OAc)_2{\cdot}H_2O$ would catalyse the intermolecular HDE between **5-$^{13}C_2$-$^{15}N_2$** and **5-D**. 225 µL 31 mM **5-$^{13}C_2$-$^{15}N_2$** (1.30 mg, 7.50 µmol, 174.18 g·mol^{-1} in 225 µL CD_3CN) was mixed with 225 µL 31 mM **5-D** (1.30 mg, 7.50 µmol, 171.22 g·mol^{-1} in 225 µL CD_3CN) in a clean and dry NMR tube. To this tube was added 50 µL 15.5 mM $Cu(OAc)_2{\cdot}H_2O$ (3.10 mg, 15.5 µmol, 199.65 g·mol^{-1} in 2.00 mL CD_3CN) reaching a final concentration of 15.5 mM analyte and 5.00 mol% catalyst. The tube was immediately capped, shaken vigorously, and lowered down the probe. ^{1}H spectra (acquired on a AVIIIHD Nanobay400 NMR spectrometer. Parameters: TE = 300 K, NS = 32, D1 = 1 sec, RG = 211.5) were acquired at 300 K over 2.7 hours. The rate of deuteration of **5-$^{13}C_2$-$^{15}N_2$** is slower than the protonation of **5-D**, which reached a steady state after 2 hours. As a control the experiment was repeated without the addition of **5-$^{13}C_2$-$^{15}N_2$**, giving a similar rate of protonation of **5-D**, indicating that there is no specific intramolecular HDE between the two ynamine molecules. This was further confirmed by monitoring the residual water resonance which integral decreases by 20% over the monitored time, which acts as a proton source (Figure S17).

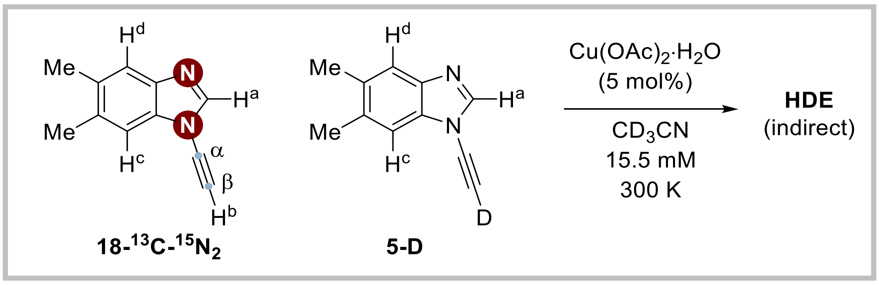

Scheme S6. Monitoring the HDE between two ynamine molecules **5-$^{13}C_2$-$^{15}N_2$** and **5-D** with 5.00 mol% $Cu(OAc)_2{\cdot}H_2O$.

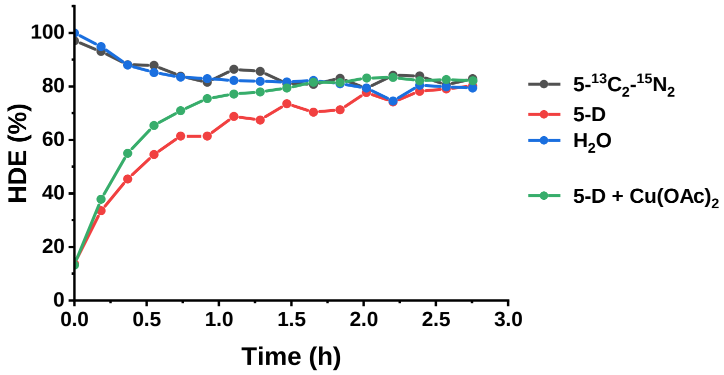

Figure S17. Monitoring the intramolecular HDE between ynamine **5-$^{13}C_2$-$^{15}N_2$** and **20** in CD_3CN at 15.5 mM and 300 K with 5.00 mol% $Cu(OAc)_2{\cdot}H_2O$, monitoring integral reduction of **5-$^{13}C_2$-$^{15}N_2$** H^b and **5-D** H^b integral increase.

3.7 (3+2) Cycloaddition reaction screening in 9:1 CD_3CN / D_2O

The HDE reactivity of **5** was correlated with the reaction rate of the (3+2) cycloaddition reaction in the presence of benzyl azide **2** in 9:1 CD_3CN / D_2O at a concentration of 62.0 mM. 69 mM alkyne/azide stock solutions were prepared in CD_3CN, and 31 mM “catalyst’’ stock solutions were prepared in D_2O. The ‘’catalyst’’ stock solution was prepared next to the NMR magnet and used immediately to prevent potential oxidation. NMR samples were prepared by transferring 450 µL alkyne/azide stock to a clean NMR tube, after which 50 µL of the ’’catalyst’’ stock was added, reaching a final alkyne concentration of 62.0 mM and 5.00 mol% “catalyst”. The addition of NaOAc is 5.00 mol% or 10.0 mol% (i.e., 2 NaOAc). After the addition of the ‘’catalyst’’ solution, ^{1}H-spectra were acquired immediately (Acquired on a AVIIIHD Nanobay400 NMR spectrometer. Parameters: TE = 300 K, NS = 16, D1 = 5 sec, RG = 211.5). When the reaction rate was fast, samples were measured manually and individually, using the *multi_zgvd2* command that included a *topshim_1dfast* shim after each acquisition. Experiments had a fixed delay of 300 seconds, n = 35 to monitor every 5 minutes over 3 hours. Slower experiments were combined on a sample carousel and measured automatically using IconNMR Automation Software at a regular intervals. Conversions were calculated by integration of the benzylic protons (CH_2) of triazole product, normalised against the integral of methyl protons (2 × CH_3) for ynamine **5** and click product **10**, and pyrrolic protons for indole **8** and click product **11**, and aryl protons for alkyne **9** and click product **12**. Since the reaction

was monitored in 10% D_2O, deuterium was incorporated into the triazole during the demetallation step (Table S6).

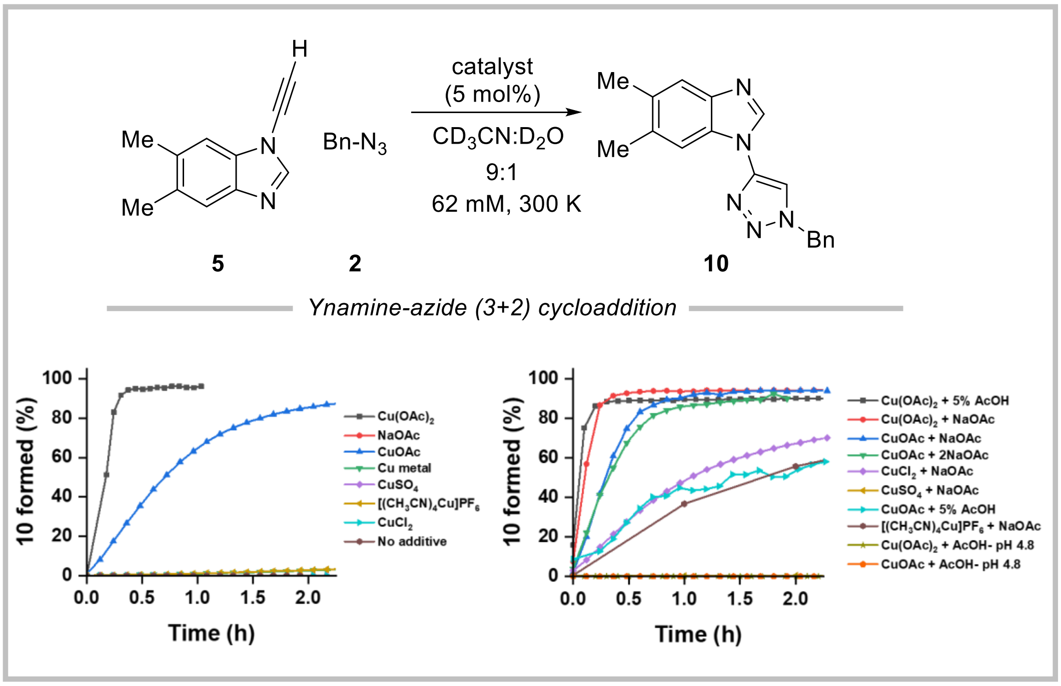

Figure S18. Ynamine-azide (3+2) cycloaddition in 9:1 CD_3CN / D_2O forming **10**. Time course of **5** to form **10** as monitored by ^1H NMR spectroscopy. The addition of NaOAc is 5.00 mol% or 10.0 mol% (i.e. 2 NaOAc). Conversions were calculated by integration of the benzylic protons of triazole product, normalised against the integral of methyl protons.

Table S6. Deuteration of **10** after the (3+2) cycloaddition reaction of **5** and **2**. Deuterium incorporation as detected by ^1H NMR, integrated against benzylic triazole protons in final acquired time point. ^{A} 100% CD_3CN.

Catalyst	**10** deuterium incorporation	Comment
$Cu(OAc)_2{\cdot}H_2O$	96 - 86%	n = 2
$Cu(OAc)_2{\cdot}H_2O^A$	0%	
$CuSO_4$	ND	No product
$CuCl_2$	ND	No product
CuOAc	96%	
$CuPF_6$	ND	Peak overlap
NaOAc	ND	No product
$Cu(OAc)_2{\cdot}H_2O$ + NaOAc	93%	

$Cu(OAc)_2{\cdot}H_2O$ + 5% AcOH	ND	Peak overlap
$CuSO_4$ + NaOAc	ND	Trace product
$CuCl_2$ + NaOAc	97%	
CuOAc + NaOAc	93%	
CuOAc + 2 NaOAc	92%	
$CuPF_6$ + NaOAc	94%	

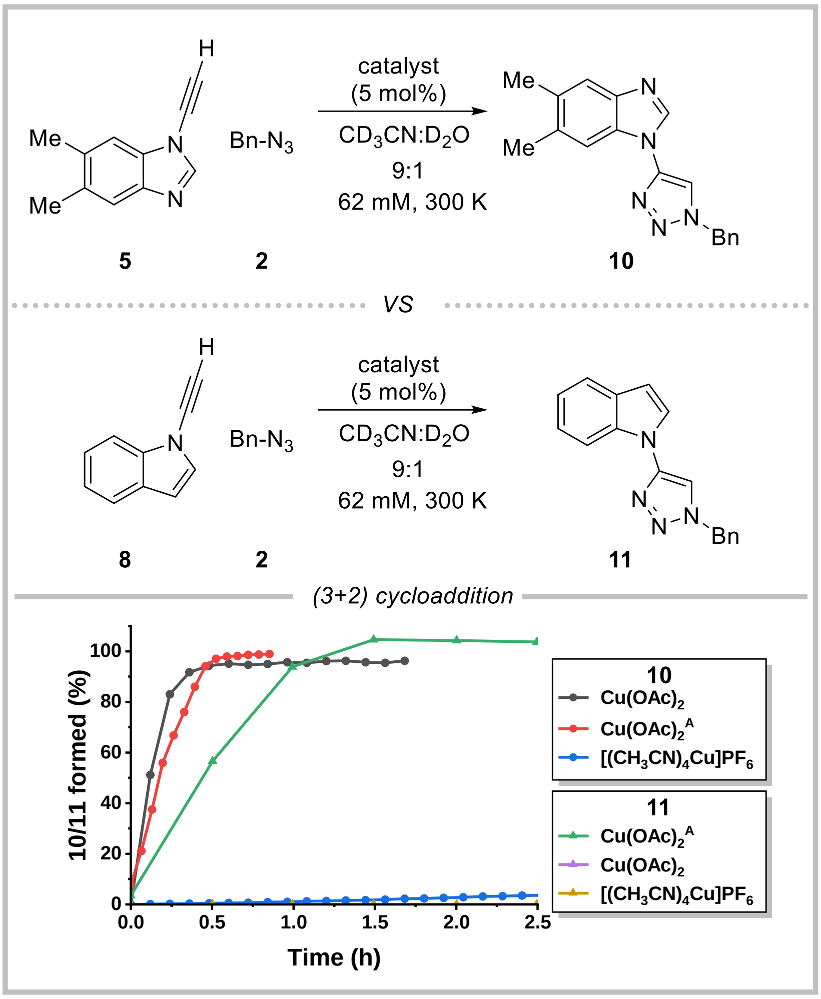

Figure S19. (3+2) Cyclo-addition in 9:1 CD_3CN / D_2O with indole **8** forming triazole **11**, compared to ynamine **5** forming triazole **10**. Time course as monitored by ^1H NMR spectroscopy. [A]100% CD_3CN. Conversions were calculated by integration of the benzylic protons of triazole product, normalised against the integral of methyl protons for **10**, and pyrrolic protons for indole **8** and indole-triazole **11**.

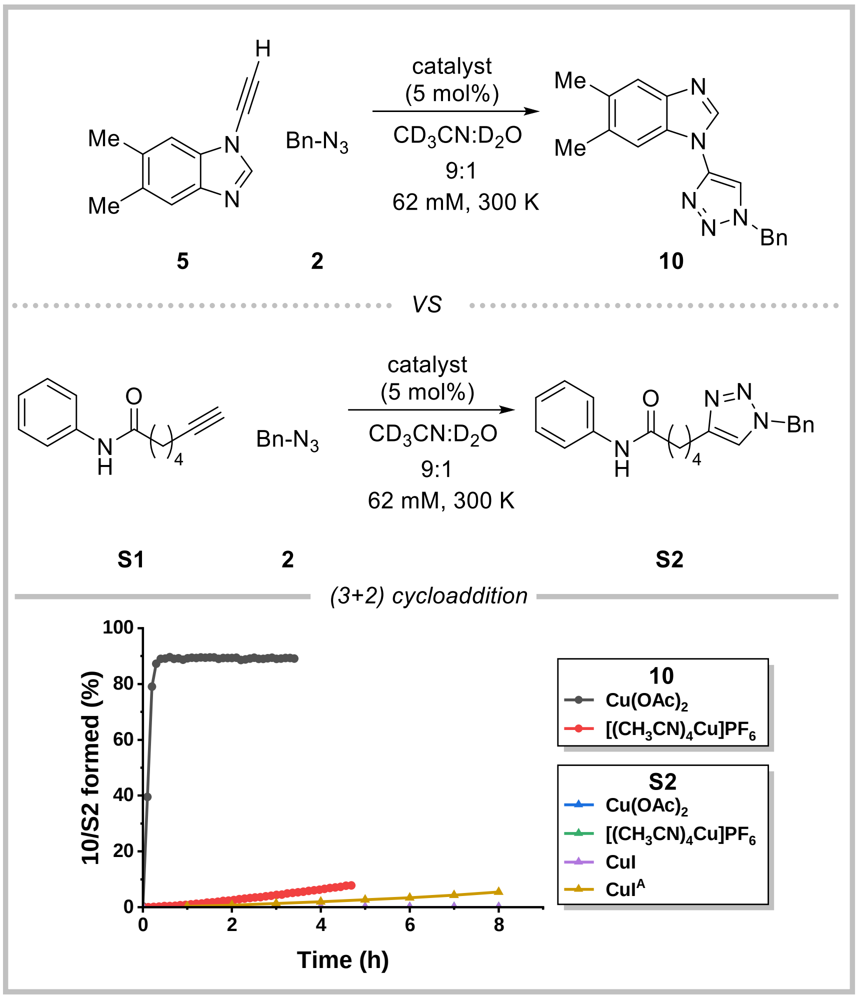

Figure S20. (3+2) Cyclo-addition in 9:1 CD_3CN / D_2O with alkyne **S1** forming triazole **S2**, compared to ynamine **5** forming triazole **10**. Time course as monitored by ^1H NMR spectroscopy. [A]100% CD_3CN. Conversions were calculated by integration of the benzylic protons of triazole product, normalised against the integral of methyl protons for **10**, and aryl protons for alkyne **S1** and triazole **S2**.

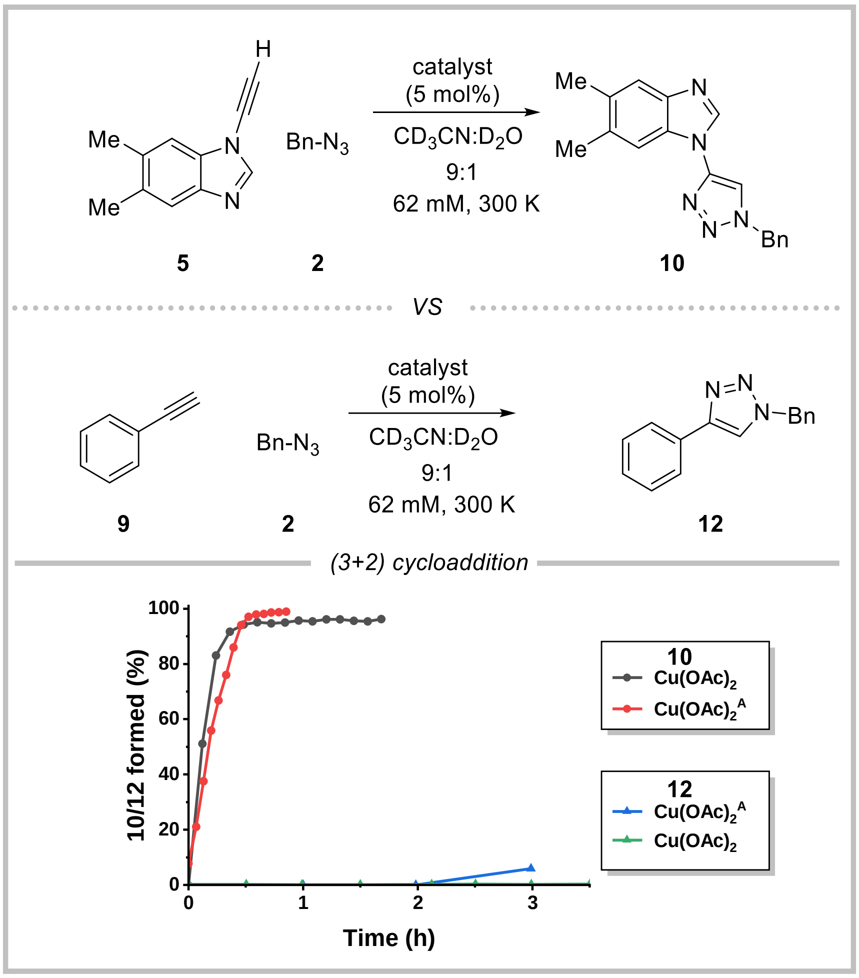

Figure S21. (3+2) Cycloaddition in 9:1 CD_3CN/D_2O with phenylacetylene **9** forming triazole **12**, compared to ynamine **5** forming triazole **10**. Time course as monitored by ^1H NMR spectroscopy. [A]100% CD_3CN. Conversions were calculated by integration of the benzylic protons of triazole product, normalised against the integral of methyl protons for **10**, and benzylic protons for alkyne **9** and triazole **12**.

3.8 ^1H analysis of compound 14 in presence of $Cu(OAc)_2{\cdot}H_2O$

The effect of paramagnetic Cu(II) on **14** was studied. To a glass vial was added **14** (10.1 mg, 0.031 mmol, 326.56 g·mol^{-1}, 1.00 equiv) and 400 µL CD_3CN, reaching a concentration of 77.5 mM. The mixture was transferred to an NMR tube, which was capped and lowered into the magnet, locked, tuned and matched, and shimmed. ^1H and ^{15}N were acquired. The sample was ejected from the magnet and freshly prepared 100 µL 15.0 mM $Cu(OAc)_2{\cdot}H_2O$ stock solution was added (6.20 mg $Cu(OAc)_2{\cdot}H_2O$, 199.65 g·mol^{-1}, 31.0 µmol in 2.00 mL CD_3CN), reaching a final reagent concentration of 62.0 mM and 3.10 mM (5.00 mol%) catalyst. The sample was shaken vigorously and immediately lowered into the magnet to start the measurement.

Prior to the addition of Cu(II), both aromatic resonances for **14** $H^{a\text{-}d}$ were sharp and intense. Thereafter, all aromatic ^1H resonances displayed line-shape broadening and experienced a downfield, isotropic shift, in reference to the solvent. Line-shape broadening affects H^a & H^d more than H^c. This effect was stable with time.

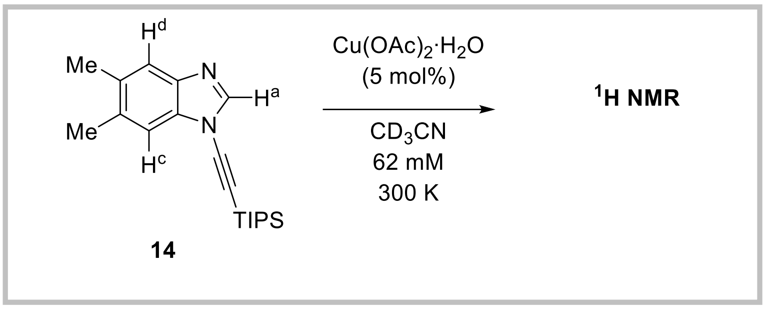

Scheme S7. Addition of Cu(II) to **14** (annotated for NMR) to study its effect on the ^1H resonances.

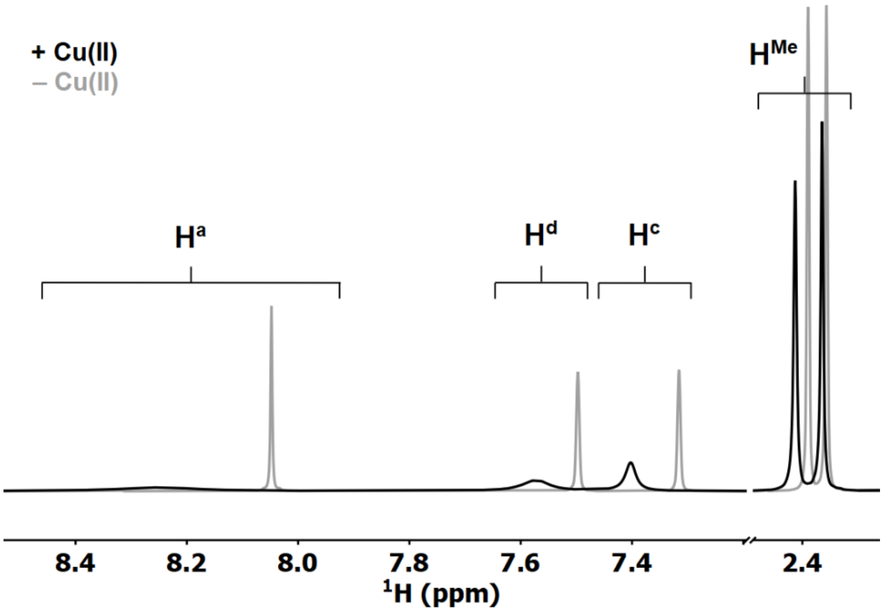

Figure S22. Superimposed 1D ^1H spectra of 62.0 mM **14**, without $Cu(OAc)_2{\cdot}H_2O$ (grey), and with 5.00 mol% $Cu(OAc)_2{\cdot}H_2O$ added (black), in CD_3CN, with annotations. Referenced to CD_3CN, ^1H = 1.94 ppm. Acquired on a AVIIIHD Nanobay400 NMR spectrometer, NS = 16, RG = 189.8, D1 = 3.

3.9 ^1H-^{15}N HMBC analysis of compound 14-$^{15}N_2$ in presence of $Cu(OAc)_2{\cdot}H_2O$

The effect of paramagnetic Cu(II) on **14-$^{15}N_2$** was studied. To a glass vial was added **14-$^{15}N_2$** (10.2 mg, 0.031 mmol, 328.55 g·mol^{-1}, 1.00 equiv) and 400 µL CD_3CN, reaching a concentration of 77.5 mM. The mixture was transferred to an NMR tube, which was capped

and lowered into the magnet, locked, tuned and matched, and shimmed. ^{1}H and ^{15}N were acquired. The sample was ejected from the magnet and freshly prepared 100 µL 15.0 mM $Cu(OAc)_2{\cdot}H_2O$ stock solution was added (6.20 mg $Cu(OAc)_2{\cdot}H_2O$, 199.65 g·mol^{-1}, 31.0 µmol in 2.00 mL CD_3CN), reaching a final reagent concentration of 62.0 mM and 3.10 mM (5.00 mol%) catalyst. The sample was shaken vigorously and immediately lowered into the magnet to start the measurement.

Prior to the addition of Cu(II), both nitrogen signals for N^1 and N^3 were observed, thereafter, all ^{1}H-^{15}N correlations were lost. ^{1}H shifts experienced a downfield, isotropic shift, in reference to the solvent. Line-shape broadening affects H^a & H^d more than H^c.

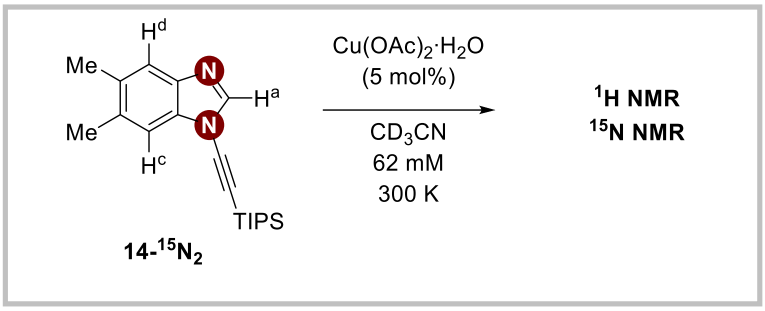

Scheme S8. Addition of Cu(II) to **14-$^{15}N_2$** (annotated for NMR) to study its effect on N^3 and surrounding protons.

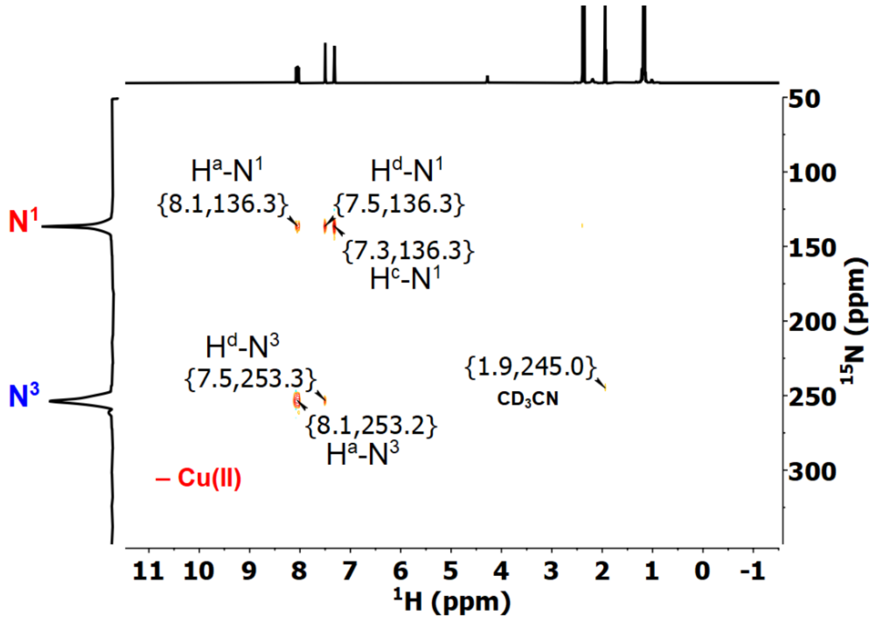

Figure S23. 2D ^{1}H-^{15}N HMBC spectrum of 62.0 mM **14-$^{15}N_2$** in CD_3CN, with correlations annotated. Referenced to CD_3CN, ^{1}H = 1.94 ppm, 77.5 mM, ^{15}N = 245 ppm. Acquired on a AVIIIHD Nanobay400 NMR spectrometer, NS = 2, RG = 211.5, D1 = 1.8.

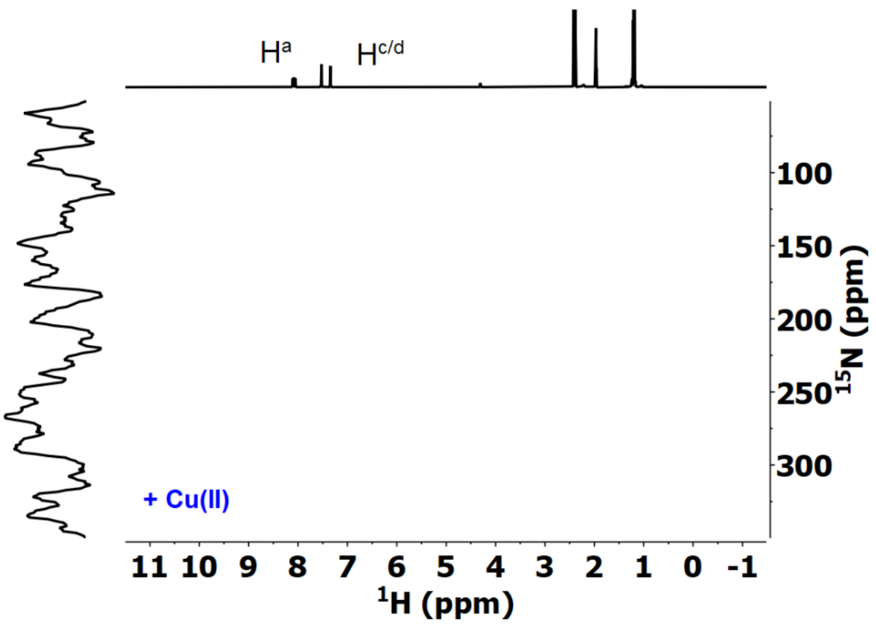

Figure S24. 2D ^{1}H ^{15}N HMBC spectrum of 62.0 mM **14-$^{15}N_2$**, with 5.00 mol% $Cu(OAc)_2{\cdot}H_2O$ added, in CD_3CN, with correlations annotated. Referenced to CD_3CN, ^{1}H = 1.94 ppm, ^{15}N = 245 ppm. Acquired on a AVIIIHD Nanobay400 NMR spectrometer, NS = 16, RG = 211.5, D1 = 1.8.

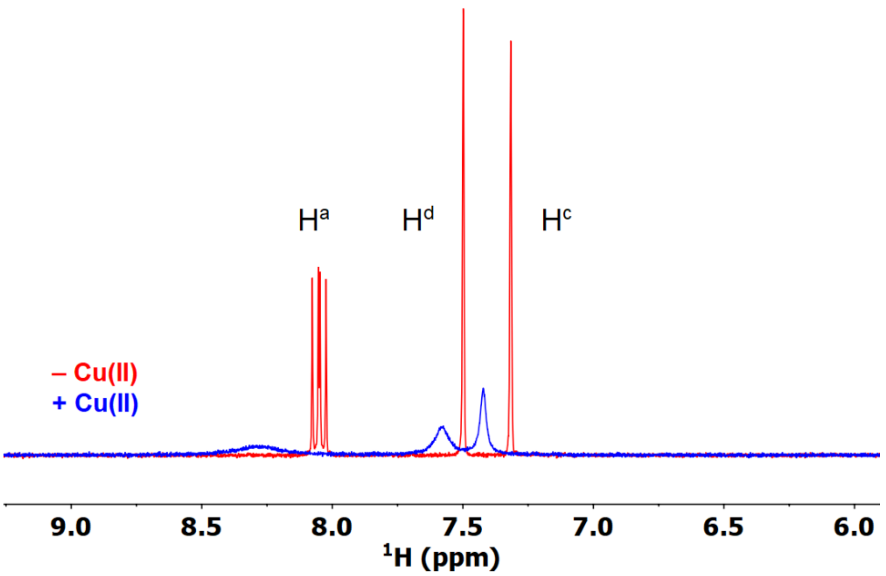

Figure S25. Superimposed 1D ^{1}H spectra of 62.0 mM **14-$^{15}N_2$**, without $Cu(OAc)_2{\cdot}H_2O$ (red), and with 5.00 mol% $Cu(OAc)_2{\cdot}H_2O$ added (blue), in CD_3CN, with annotations. Referenced to CD_3CN, ^{1}H = 1.94 ppm. Acquired on a AVIIIHD Nanobay400 NMR spectrometer, NS = 1, RG = 189.8, D1 = 3.

3.10 ^{13}C Signal to noise ratio for compound 14-$^{13}C_2$-$^{15}N_2$

14-$^{15}N_2$ was used to determine the minimal required scans to give a signal to noise ratio of > 10 at various concentrations in CD_3CN for zgpg30 $^{13}C\{^1H\}$. For this, the resonances for **14-$^{15}N_2$** C^{α} and C^{β} were compared to the noise using the TopSpin AU-program *sinocal*. Signal left limit = 100 ppm, right limit = 60 ppm. Noise left limit = 60 ppm, right limit = 20 ppm, noise width = 40 ppm. RG = 912, D1 = 0.7, TE = 300.

Table S 7. ^{13}C signal to noise ratios for **14-$^{15}N_2$** in CD_3CN at various concentrations and scans.

NS	EXPT (s)	SINO		
		62.0 mM	14.5 mM	7.25 mM
1024	1037	181.3	--	--
512	520	153.7	--	--
256	262	124.6	--	--
128	133	93.7	--	--
64	68	69.6	16.7	10.6
32	36	50.9	12.6	6.9
16	20	37.5	9.7	5.9

3.11 ^{13}C NMR analysis of compound 14-$^{13}C_2$-$^{15}N_2$ in presence of $Cu(OAc)_2{\cdot}H_2O$

The effect of paramagnetic Cu(II) on **14-$^{13}C_2$-$^{15}N_2$** was studied. To a glass vial was added **14-$^{13}C_2$-$^{15}N_2$** (23.0 mg, 69.6 µmol, 330.52 g·mol^{-1}), and 900 µL CD_3CN, reaching a concentration of 77.5 mM. 400 µL of this mixture was transferred to an NMR tube and 100 µL CD_3CN was added, reaching a concentration of 62.0 mM. This reference sample was capped and lowered into the magnet. The reference sample was used to locked, tune, match, and shim the magnet in preparation of the reaction sample. A ^{1}H and $^{13}C\{^1H\}$ was acquired at 298 K and the sample was ejected. Thereafter, 400 µL 77.5 mM **14-$^{13}C_2$-$^{15}N_2$** in CD_3CN, and 100 µL of a 15.5 mM $Cu(OAc)_2{\cdot}H_2O$ solution (15.5 µmol, 199.65 g·mol^{-1}, 3.10 mg in 1.00 mL CD_3CN) was added, reaching a final concentration of 62.0 mM **14-$^{13}C_2$-$^{15}N_2$** and 5.0 mol% $Cu(OAc)_2{\cdot}H_2O$.

Prior to the addition of copper, both resonances for C^{α} and C^{β} were observed, thereafter, a small isotropic shift in C^{α} was detected. It is postulated that N^3 is chelating Cu(II), which generated anisotropy is translated to C^{α}. When the experiment was repeated with 1.00 equiv Cu(II), it produced only noise.

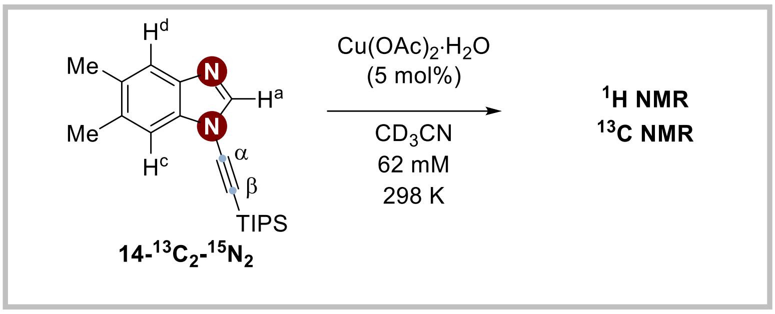

Scheme S9. Addition of 5 mol% $Cu(OAc)_2·H_2O$ to **14-$^{13}C_2$-$^{15}N_2$** (annotated for NMR) to study its effect on alkyne carbons $^{13}C^{\alpha}$, $^{13}C^{\beta}$ at 298 K and 62.0 mM.

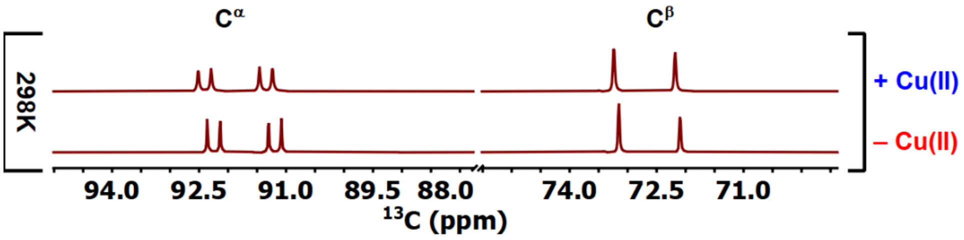

Figure S26. Stacked 1D $^{13}C\{^{1}H\}$ spectra of 62 mM **14-$^{13}C_2$-$^{15}N_2$**, without $Cu(OAc)_2·H_2O$ (red), and with 5 mol% $Cu(OAc)_2·H_2O$ added (blue) at 298 K, in CD_3CN, with annotations. Referenced to CD_3CN, δ ^{13}C = 180.26 ppm. Acquired on a AVIIIHD Nanobay400 NMR spectrometer, NS = 1024, RG = 406, D1 = 0.7.

Table S8. Effect on carbon shifts after the addition of 5 mol% $Cu(OAc)_2·H_2O$ to **14-$^{13}C_2$-$^{15}N_2$** to study its effect on alkyne carbons $^{13}C^{\alpha}$ and $^{13}C^{\beta}$.

K	Cu(II)	δ $^{13}C^{\alpha}$ (ppm)	δ $^{13}C^{\beta}$ (ppm)	Δ ppm
	+	91.87	72.71	19.16
298	–	91.72	72.62	19.10
	Δ ppm	0.26	0.09	/

Table S9. Effect on J_{CC} and J_{CN} of Cα and Cβ after the addition of 5 mol% $Cu(OAc)_2·H_2O$ to **14-$^{13}C_2$-$^{15}N_2$**.

K	Cu(I)	Alkyne-carbon	Δ (ppm)	J_{CC} (Hz)	J_{CN} (Hz)
298	+	α	91.87	159.56	33.56
		β	72.71	160.09	/
	-	α	91.72	159.47	33.59
		β	72.62	159.47	/

3.12 ^{1}H analysis of compound 14 in presence of $[(CH_3CN)_4Cu]PF_6$

The effect of diamagnetic Cu(II) on **14** was studied. To a glass vial was added **14** (33.8 mg, 0.104 mmol, 326.56 g·mol^{-1}, 1.00 equiv) and 1.50 mL CD_3CN, reaching a concentration of 69 mM. 450 µL of the mixture was transferred to an NMR tube, and µL CD_3CN was added. The tube was capped and lowered into the magnet, locked, tuned and matched, and shimmed. ^{1}H was acquired. To another NMR tube was added 450 µL **14** solution (69 mM in CD_3CN), to this was added freshly prepared 50 µL 31.0 mM $[(CH_3CN)_4Cu]PF_6$ stock solution was added (5.80 mg $[(CH_3CN)_4Cu]PF_6$, 372.72 g·mol^{-1}, 15.5 µmol in 500 µL CD_3CN), reaching a final reagent concentration of 62.0 mM and 3.10 mM (5.00 mol%) catalyst. The sample was shaken vigorously and immediately lowered into the magnet to start the measurement.

Prior to the addition of Cu(I), both aromatic resonances for **14** H^{a-d} were sharp and intense. Thereafter, all aromatic ^{1}H resonances displayed some line-shape broadening and experienced a minor downfield, isotropic shift, in reference to the solvent.

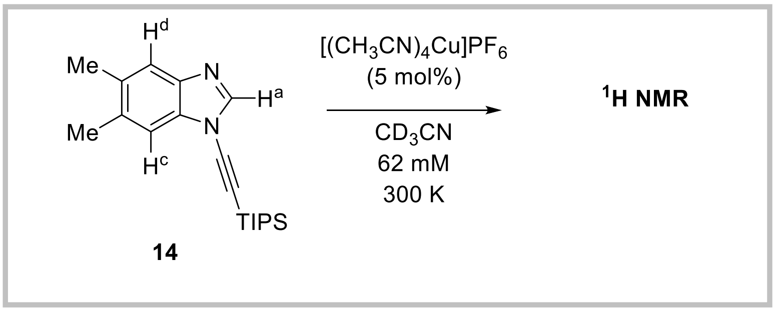

Scheme S10. Addition of Cu(I) to **14** (annotated for NMR) to study its effect on the ^{1}H resonances.

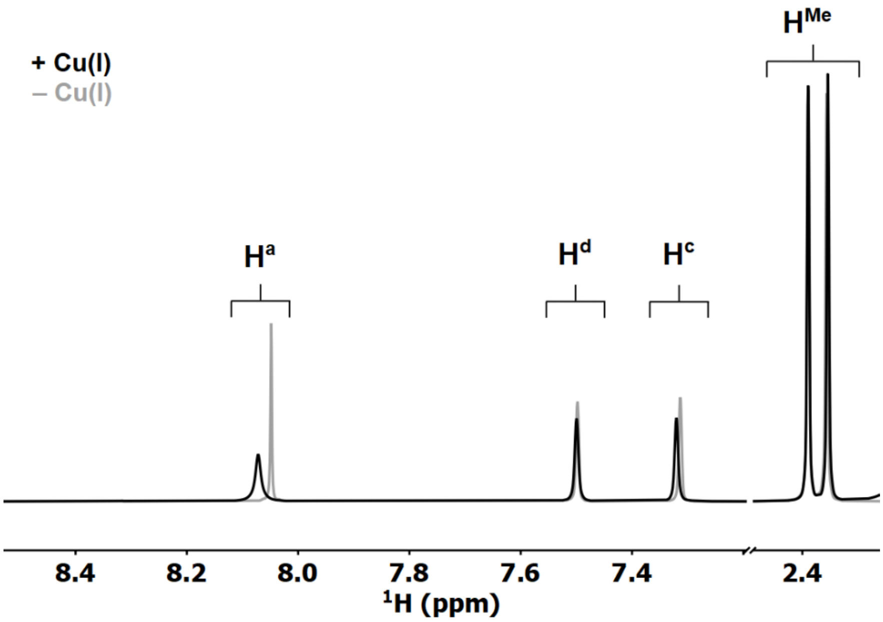

Figure S27. Superimposed 1D ^{1}H spectra of 62.0 mM **14**, without [($CH_3CN)_4$Cu]PF_6 (grey), and with 5.00 mol% [($CH_3CN)_4$Cu]PF_6 added (black), in CD_3CN, with annotations. Referenced to CD_3CN, ^{1}H = 1.94 ppm. Acquired on a AVIII400 NMR Spectrometer with Cryoprobe, NS = 16, RG = 57, D1 = 2.

3.13 ^{1}H-^{15}N HMBC and ^{13}C Analysis of compound 14-$^{15}N_2$ in presence of 5 mol% [($CH_3CN)_4$Cu]PF_6

The effect of diamagnetic [($CH_3CN)_4$Cu]PF_6 on **14-$^{15}N_2$** was studied. To a glass vial was added **14-$^{15}N_2$** (10.2 mg, 0.031 mmol, 328.55 g·mol^{-1}, 1.00 equiv) and 400 µL CD_3CN, reaching a concentration of 77.5 mM. The mixture was transferred to an NMR tube, which was capped and lowered into the magnet, locked, tuned and matched, and shimmed. ^{1}H and ^{15}N were acquired, and since the paramagnetic effect on **14** in general is stable over time, an extended number of scans allowed for the interrogation of ^{13}C. The sample was ejected from the magnet and freshly prepared 100 µL 15.0 mM Cu(OAc)$_2$·H_2O stock solution was added (11.6 mg [($CH_3CN)_4$Cu]PF_6, 372.72 g·mol^{-1}, 31.0 µmol in 2.00 mL CD_3CN), reaching a final reagent concentration of 62.0 mM and 3.10 mM (5.00 mol%) catalyst. The sample was shaken vigorously and immediately lowered into the magnet to start the measurement.

Prior to the addition of Cu(I), both nitrogen signals for N^1 and N^3 were observed, thereafter, all ^{1}H-^{15}N correlations were lost. ^{1}H shifts experienced a downfield, isotropic shift, in reference to the solvent. Line-shape broadening affects H^a & H^d more than H^c.

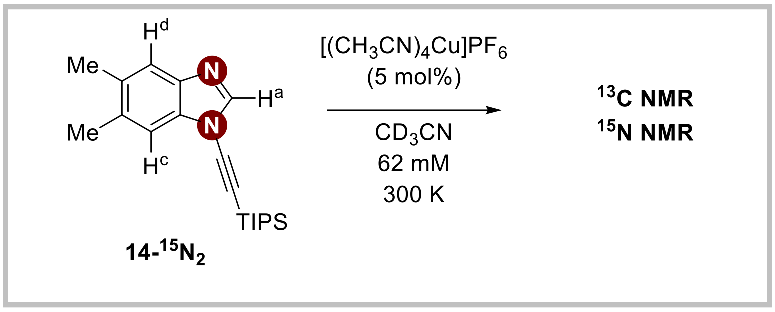

Scheme S11. Addition of Cu(I) to **14-$^{15}N_2$** (annotated for NMR) to study its effect on N^3 and surrounding protons.

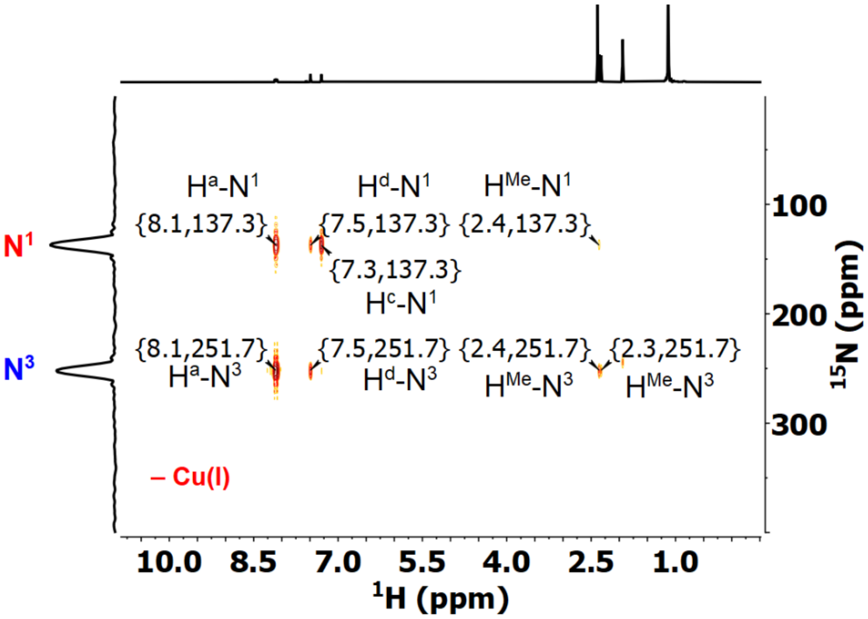

Figure S28. 2D ^1H-^{15}N HMBC spectrum of 62.0 mM **14-$^{15}N_2$** in CD_3CN, with correlations annotated. Referenced to CD_3CN, ^1H = 1.94 ppm, 77.5 mM, ^{15}N = 245 ppm. Acquired on a AVIIIHD Nanobay400 NMR spectrometer, NS = 2, RG = 211.5, D1 = 1.8.

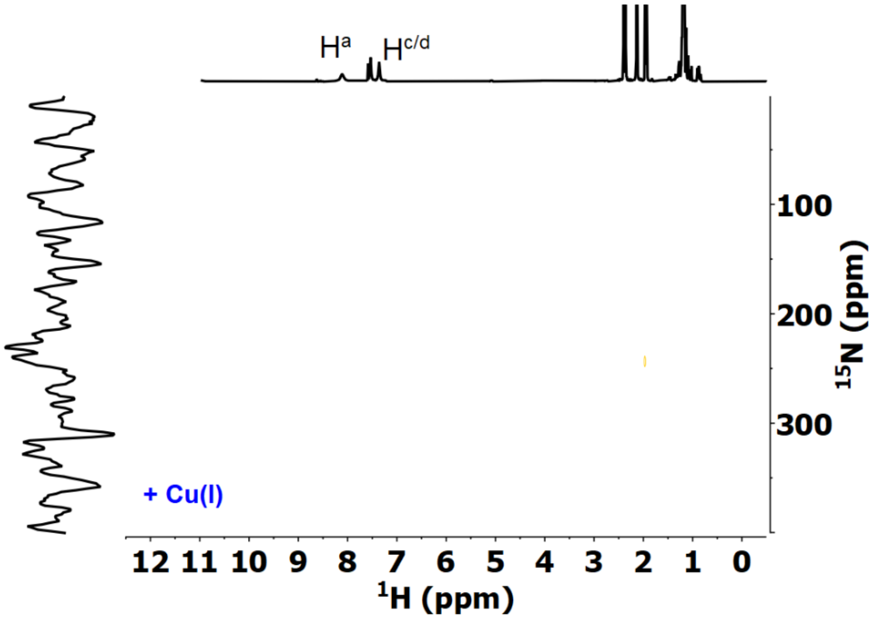

Figure S29. 2D ^{1}H ^{15}N HMBC spectrum of 62.0 mM **14-$^{15}N_2$**, with 5.00 mol% $[(CH_3CN)_4Cu]PF_6$ added, in CD_3CN, with correlations annotated. Referenced to CD_3CN, ^{1}H = 1.94 ppm, ^{15}N = 245 ppm. Acquired on a AVIIIHD Nanobay400 NMR spectrometer, NS = 16, RG = 211.5, D1 = 1.8.

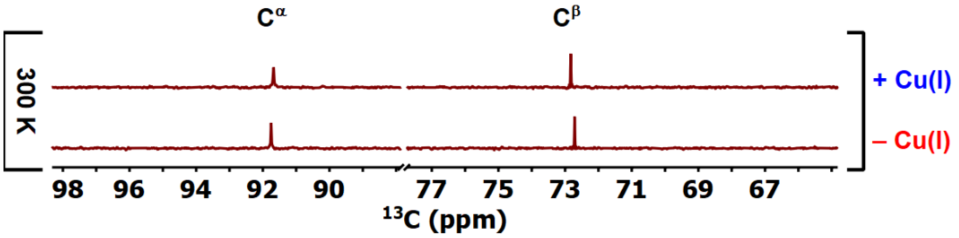

Figure S30. Stacked 1D $^{13}C\{^{1}H\}$ spectra of 62 mM **14-$^{13}C_2$-$^{15}N_2$**, without $[(CH_3CN)_4Cu]PF_6$ (red), and with 5 mol% $[(CH_3CN)_4Cu]PF_6$ added (blue) at 300 K, in CD_3CN, with annotations. Referenced to CD_3CN, δ ^{13}C = 180.26 ppm. Acquired on a AVIIIHD Nanobay400 NMR spectrometer, NS = 2048, RG = 2050, D1 = 0.7.

3.14 VT-NMR analysis of compound 14-$^{13}C_2$-$^{15}N_2$ in presence of 1.0 equiv $[(CH_3CN)_4Cu]PF_6$

The effect of diamagnetic Cu(I) on **14-$^{13}C_2$-$^{15}N_2$** was studied. To a glass vial was added **14-$^{13}C_2$-$^{15}N_2$** (6.15 mg, 18.6 µmol, 330.52 g·mol^{-1}, 1.00 equiv) and 300 µL CD_3CN, reaching a concentration of 62.0 mM. 125 µL of this mixture was transferred to an NMR tube and 375 µL CD_3CN was added, reaching a concentration of 15.5 mM. This reference sample was capped, inserted into a ceramic standardbore spinner, and lowered into the magnet. The probe was then cooled to 233 K with liquid nitrogen, maintaining a shim coil temperature of >278 K. When

the desired temperature was reached and stable, the reference sample was locked, tuned and matched, and shimmed. ^{1}H, $^{13}C\{^{1}H\}$, ^{13}C and ^{1}H-^{15}N HMBC were acquired at 233, 253, 263, 273, and 300 K, shimming at each temperature interval. Thereafter, the probe was cooled back down to 233 K with the reference sample in place. 125 µL 62.0 mM **14-$^{13}C_2$-$^{15}N_2$** in CD_3CN, and 125 µL CD_3CN was added to an NMR tube, reaching a concentration of 31 mM. The NMR tube was chilled in a $CO_{2(s)}$ / CH_3CN cooling bath. To the chilled NMR tube was then added freshly prepared and pre-pipetted and chilled 250 µL 31 mM $[(CH_3CN)_4Cu]PF_6$ (5.8 mg, 372.72 g·mol^{-1} in 500 µL CD_3CN), reaching a final reagent concentration of 15.5 mM and 15.5 mM of catalyst. The sample was shaken vigorously and immediately lowered into the magnet to start the measurement. The sample was locked, tuned and matched, and shimmed. ^{1}H, $^{13}C\{^{1}H\}$, ^{13}C and ^{1}H-^{15}N HMBC were acquired at 233, 253, 263, 273, and 300 K, shimming at each temperature interval.

Prior to the addition of copper, both nitrogen signals for N1 and N3 were observed at all temperatures, thereafter, all ^{1}H-^{15}N correlations were lost. ^{1}H shifts experienced a downfield, isotropic shift. Line-shape broadening affects H^a & H^d more than H^c. In addition, acquired $^{13}C\{^{1}H\}$ and ^{13}C spectra found an upfield shift for C^α, and a downfield shift was observed for C^β.

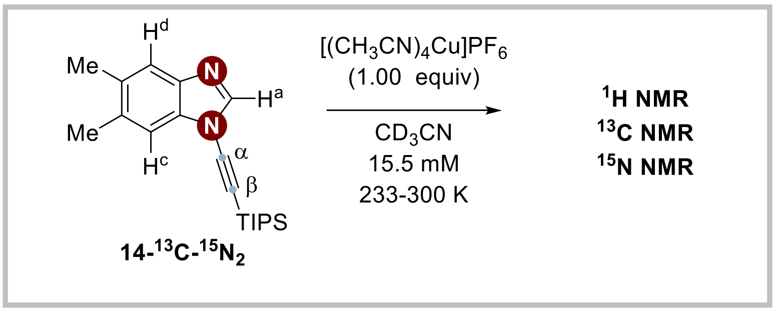

Scheme S12. Addition of 1.00 equiv $[(CH_3CN)_4Cu]PF_6$ to 15.5 mM $^{13}C_2$-$^{15}N_2$ ynamine-TIPS **17** (annotated for NMR) to study its effect on alkyne carbons $^{13}C^\alpha$ and $^{13}C^\beta$, and surrounding protons at various temperatures.

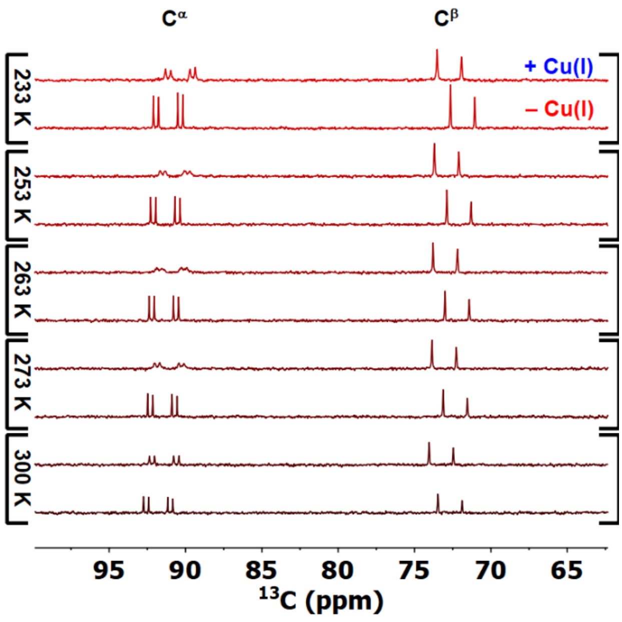

Figure S31. Stacked 1D ^{13}C spectra of 15.5 mM **14-$^{13}C_2$-$^{15}N_2$**, without $[(CH_3CN)_4Cu]PF_6$ (red), and with 1.00 equiv $[(CH_3CN)_4Cu]PF_6$ added (blue) at various temperatures, in CD_3CN, with annotations. Referenced to CD_3CN, δ ^{13}C = 180.26 ppm. Acquired on a AVIIIHD Nanobay400 NMR spectrometer, NS = 64, RG = 211.5, D1 = 0.7 ^{1}H-decoupler off.

Table S10. Effect on carbon shifts after the addition of 1.00 equiv $[(CH_3CN)_4Cu]PF_6$ to **14-$^{13}C_2$-$^{15}N_2$** to study its effect on alkyne carbons $^{13}C^{\alpha}$ and $^{13}C^{\beta}$.

K	Cu(I)	δ $^{13}C^{\alpha}$ (ppm)	δ $^{13}C^{\beta}$ (ppm)	Δ ppm
	+	90.34	72.72	17.62
233	–	91.14	71.85	19.29
	Δ ppm	0.80	-0.87	/
	+	90.69	72.90	17.79
253	–	91.32	72.09	19.23
	Δ ppm	0.63	-0.81	/
	+	90.90	72.99	17.91
263	–	91.42	72.21	19.21
	Δ ppm	0.52	-0.78	/
	+	91.06	73.06	18
273	–	91.52	72.33	19.19
	Δ ppm	0.46	-0.73	/
	+	91.40	73.25	18.15
300	–	91.79	72.68	19.11
	Δ ppm	0.39	-0.57	/

Table S11. Effect on J_{CC} and J_{CN} of Cα and Cβ after the addition of 1.00 equiv. $[(CH_3CN)_4Cu]PF_6$ to **14-$^{13}C_2$-$^{15}N_2$**

K	Cu(I)	Alkyne-carbon	Δ (ppm)	J_{CC} (Hz)	J_{CN} (Hz)
233	+	α	90.34	161.16	34.41
		β	72.72	160.87	/
	-	α	91.14	160.41	33.85
		β	71.85	160.53	/
253	+	α	90.69	162.15	33.84
		β	72.90	160.57	/
	-	α	91.32	160.16	33.87
		β	72.09	160.11	/
263	+	α	90.90	163.22	33.73
		β	72.99	160.70	/
	-	α	91.42	159.96	33.85
		β	72.21	159.86	/
273	+	α	91.06	160.08	32.16
		β	73.06	160.17	/
	-	α	91.52	159.94	33.68
		β	72.33	159.91	/
300	+	α	91.40	160.29	33.91
		β	73.25	159.82	/
	-	α	91.79	159.39	33.59
		β	72.68	159.58	/

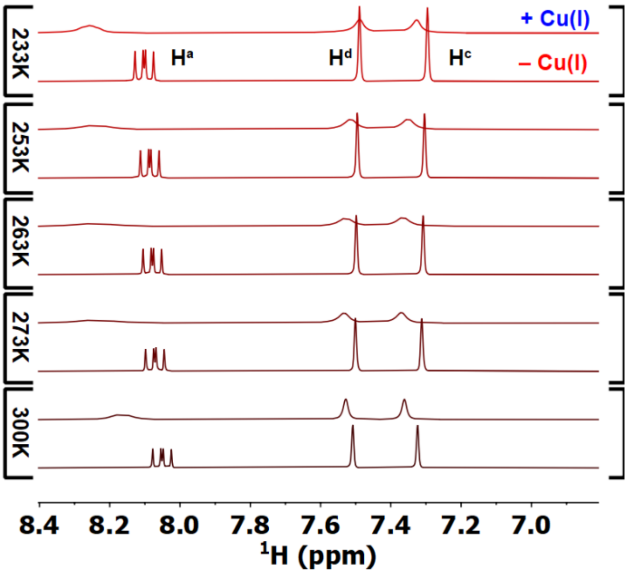

Figure S32. Stacked 1D ^{1}H spectra of 15.5 mM **14-$^{13}C_2$-$^{15}N_2$**, without $[(CH_3CN)_4Cu]PF_6$ (red), and with 1.00 equiv $[(CH_3CN)_4Cu]PF_6$ added (blue) at various temperatures, in CD_3CN, with annotations. Referenced to CD_3CN, ^{1}H = 1.94 ppm. Acquired on a AVIIIHD Nanobay400 NMR spectrometer, NS = 16, RG = 211.5, D1 = 1.0

3.15 ^{1}H-NMR analysis of compound 5 in presence of $Cu(OAc)_2 \cdot H_2O$

The effect of paramagnetic Cu(II) on ynamine **5** was studied by means of ^{1}H NMR, varying the copper concentration from 0.125 – 5.00 mol% $Cu(OAc)_2 \cdot H_2O$. To a glass vial was added ynamine **5** (87.0 mg, 170.22 g·mol^{-1}, 510 µmol), 1,3,5-trimethoxybenzene (86 mg, 510 µmol, 168.19 g·mol^{-1}) and 6.60 mL CD_3CN, reaching a concentration of 77.5 mM. 400 µL of this mixture was transferred to an NMR tube and 100 µL CD_3CN was added, reaching a concentration of 62.0 mM. This reference sample was capped and lowered into the magnet. The reference sample was used to locked, tune, match, and shim the magnet in preparation of the reaction sample. ^{1}H was acquired at 298 K and the sample was ejected. A 15.5 mM $Cu(OAc)_2 \cdot H_2O$ stock was prepared in CD_3CN and diluted (Table S12). Thereafter, 400 µL 77.5 mM ynamine **5** in CD_3CN, and 100 µL of the representative $Cu(OAc)_2 \cdot H_2O$ solution was added to an NMR tube, reaching a final concentration of 62.0 mM ynamine **5** and 1,3,5-trimethoxybenzene, and 0.125, 0.25, 0.50, 1.00 and 5.00 mol% $Cu(OAc)_2 \cdot H_2O$.

Table S12. Catalyst stock solution dilution table in CD_3CN.

Rep.% cat.	V solvent (µL)	V cat. Stock (µL)	Cat. (mM)
5.00	0	2000	15.50

2.50	250	250	7.75
1.00	400	100	3.10
0.50	250	0	1.55
0.25	250	0	0.78
0.125	250	0	0.39

The sample was shaken vigorously and immediately lowered into the magnet to start the measurement. ^{1}H-spectra were acquired immediately (parameters: TE = 298 K, NS = 4, D1 = 5 sec, RG = 128). using the *multi_zgvd2* command that included a *topshim_1dfast* shim after each acquisition. Experiments had a fixed delay of 60 seconds, n = 35.

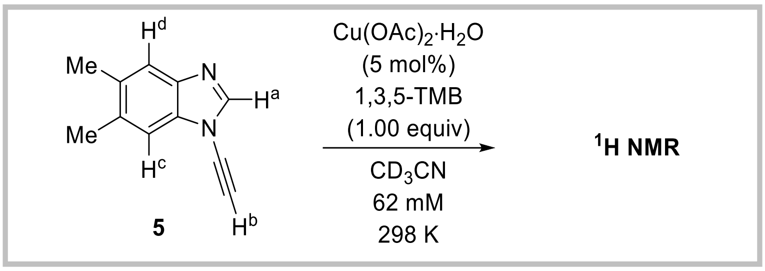

Scheme S13. Addition of 5.00 mol% $Cu(OAc)_2{\cdot}H_2O$ to 62.0 mM **5** (annotated for NMR) to study its effect on protons.

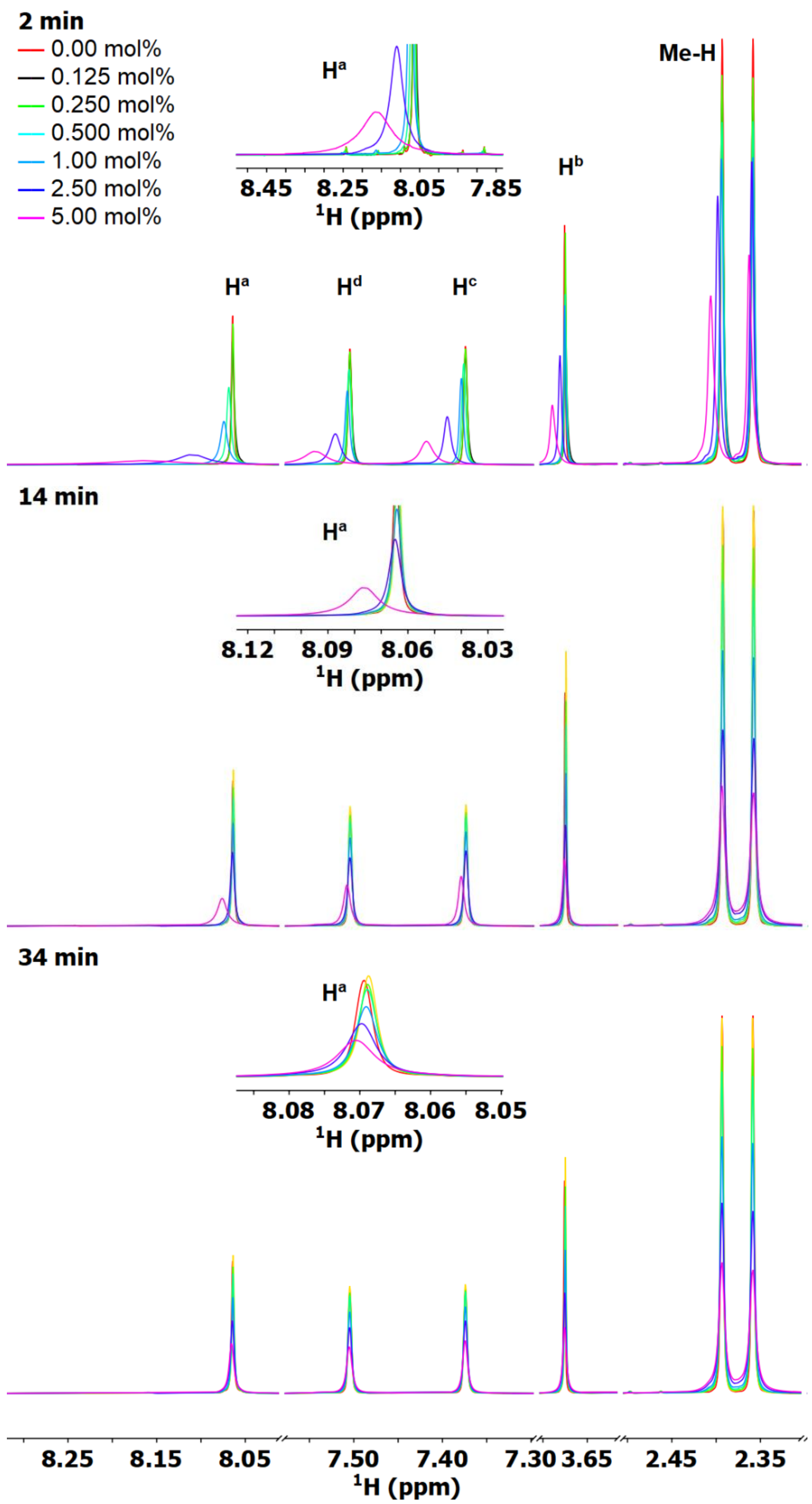

Figure S33. Superimposed 1D ^{1}H spectra of 62.0 mM **5** in CD_3CN, with 0.00 – 5.00 mol% $Cu(OAc)_2·H_2O$. As acquired at t = 2 min (top), 14 min (centre), and 34 min (bottom). Referenced to CD_3CN, ^{1}H = 1.94 ppm. Expansion plots for H^a for each representative

timepoint. Acquired on an AVII+ 600MHz spectrometer, NS = 4, RG = 128, D1 = 5, TE = 298 K, in CD_3CN, with annotations.

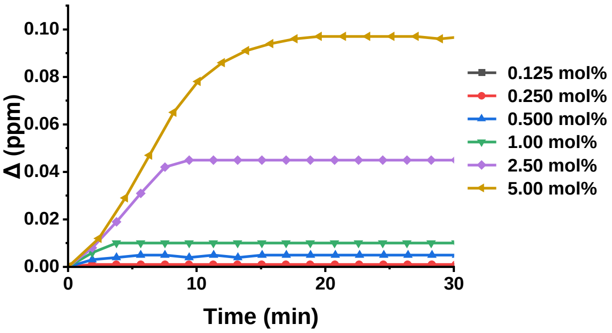

Figure S34. Isotropic shift change of H^a over time of ynamine **5** with 0.125 – 5.00 mol% $Cu(OAc)_2{\cdot}H_2O$ in CD_3CN. Referenced to CD_3CN, ^1H = 1.94 ppm.

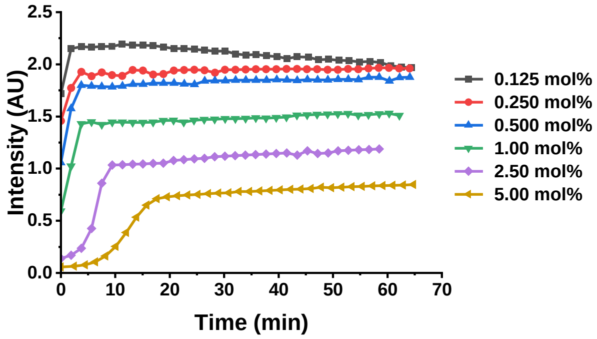

Figure S35. Intensity of H^a over time of ynamine **5** with 0.125 – 5.00 mol% $Cu(OAc)_2{\cdot}H_2O$ in CD_3CN. Referenced to CD_3CN, ^1H = 1.94 ppm.

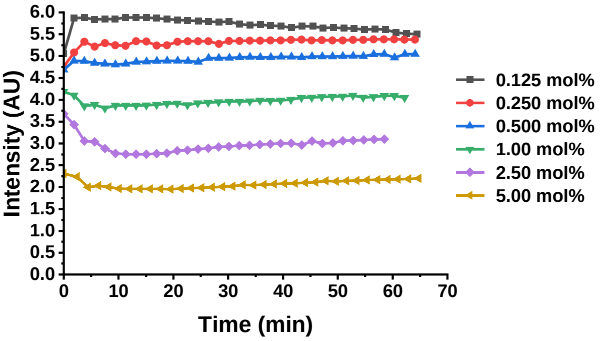

Figure S36. Intensity of H^{Me} (downfield) over time of ynamine **5** with 0.125 – 5.00 mol% $Cu(OAc)_2{\cdot}H_2O$ in CD_3CN. Referenced to CD_3CN, ^1H = 1.94 ppm.

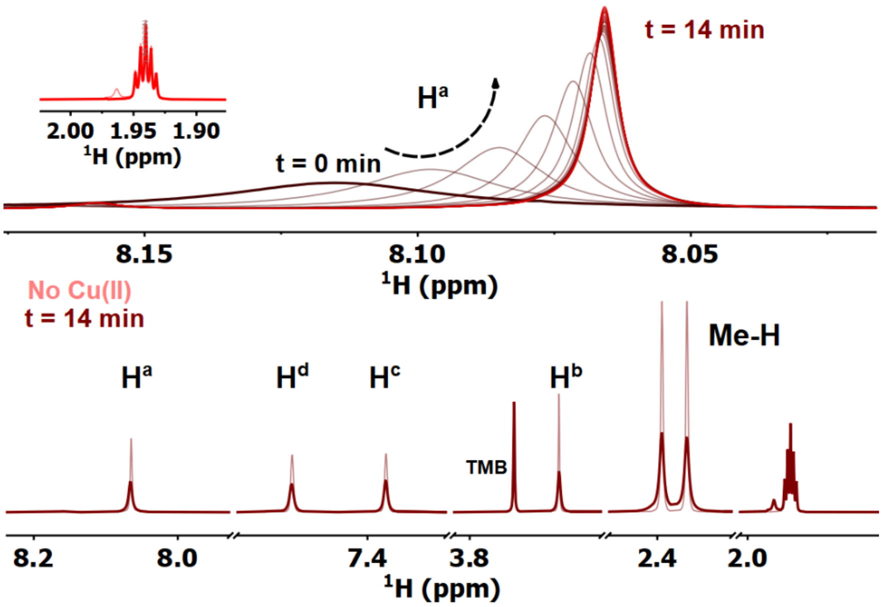

Figure S37. (top) Superimposed 1D ^{1}H spectra of 62.0 mM ynamine **5**, with 5.00 mol% $Cu(OAc)_2{\cdot}H_2O$. Monitored for one hour. (bottom) Superimposed 1D ^{1}H spectra of a reference sample without catalyst, and t = 14 min trace. Referenced to CD_3CN, ^{1}H = 1.94 ppm. Acquired on an AVII+ 600MHz spectrometer, NS = 4, RG = 128, D1 = 5, TE = 298 K, in CD_3CN, with annotations.

3.16 ^{1}H-^{15}N HMBC NMR analysis of compound 5-$^{15}N_2$ in presence of 5 mol% $Cu(OAc)_2$

The effect of paramagnetic Cu(II) on **5-$^{15}N_2$** was studied by means of ^{1}H and ^{1}H-^{15}N HMBC NMR analysis. To a glass vial was added **5-$^{15}N_2$** (11.34 mg, 172.20 g·mol^{-1}, 66.0 µmol,), and 850 µL CD_3CN, reaching a concentration of 77.5 mM. 400 µL of this mixture was transferred to an NMR tube and 100 µL CD_3CN was added, reaching a concentration of 62.0 mM. This reference sample was capped and lowered into the magnet. The reference sample was used to locked, tuned and matched, and shimmed the magnet in preparation of the reaction sample. ^{1}H ^{1}H-^{15}N HMBC and was acquired at 300 K and ejected. Thereafter, 400 µL 77.5 mM **5-$^{15}N_2$** in CD_3CN, and 100 µL 15.5 mM $Cu(OAc)_2H_2O$ (3.10 mg, 199.65 g·mol^{-1} in 1.00 mL CD_3CN) was added to an NMR tube, reaching a concentration of 62.0 mM **5-$^{15}N_2$** and 5.00 mol% $Cu(OAc)_2{\cdot}H_2O$. The sample was shaken vigorously and immediately lowered into the magnet to start the measurement. ^{1}H (TE 300, NS = 4, D1 = 1, RG = 211.5) and ^{1}H-^{15}N HMBC (TE 300 K, NS = 2, D1 = 1.8, RG = 211.5) spectra were acquired immediately using the

multi_zgvd2 command that included a *topshim_1dfast* shim after each acquisition. Experiments had a fixed delay of 200 seconds.

Upon the addition of $Cu(OAc)_2{\cdot}H_2O$ and the first acquisition of ^{1}H-^{15}N HMBC correlations were found for N^1, N^3 was not detected. Only after 20 minutes, correlations for both N1 and N3 were detected. Additionally, resonances for diyne **15-$^{15}N_4$** were detected in ^{1}H, although no ^{15}N resonances for **15-$^{15}N_4$** were detected in the ^{1}H-^{15}N HMBC. Only after the sample was left overnight the signal to noise ratio was sufficient enough to resolve the resonances for diyne **15-$^{15}N_4$** from remaining ynamine **5-$^{15}N_2$**. LC-MS analysis confirmed the presence of diyne **15-$^{15}N_4$** in the monitored reaction mixture.

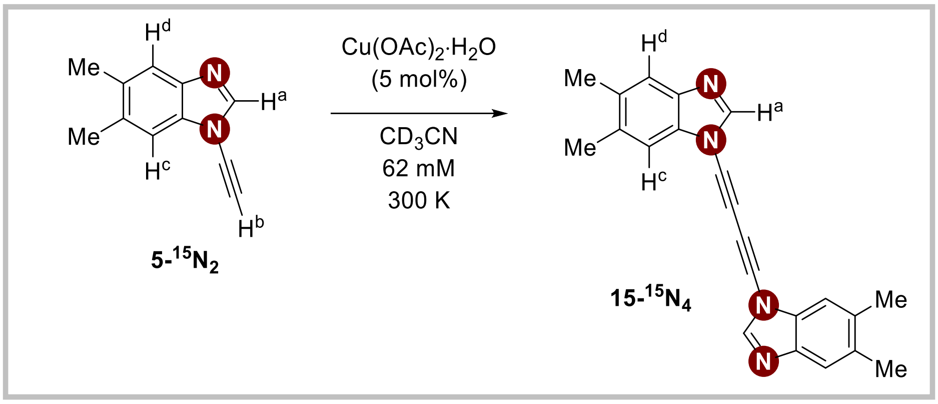

Scheme S14. Addition of 5.00 mol% $Cu(OAc)_2{\cdot}H_2O$ to 15.5 mM **5-$^{15}N_2$** (annotated for NMR) to study its effect on N^1 and N^3, and surrounding protons.

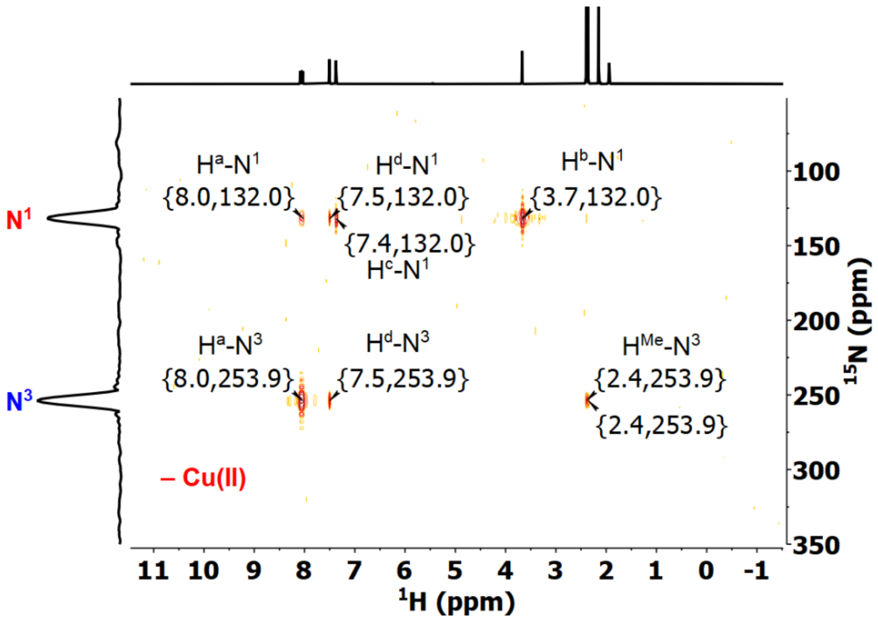

FigureS 38. 2D ^{1}H-^{15}N HMBC spectrum of 62.0 mM **5-$^{15}N_2$** in CD_3CN. Correlations annotated. Referenced to CD_3CN, ^{1}H = 1.94 ppm, ^{15}N = 245 ppm. Acquired on a AVIIIHD Nanobay400 NMR spectrometer, NS = 2, RG = 211.5, D1 = 1.8.

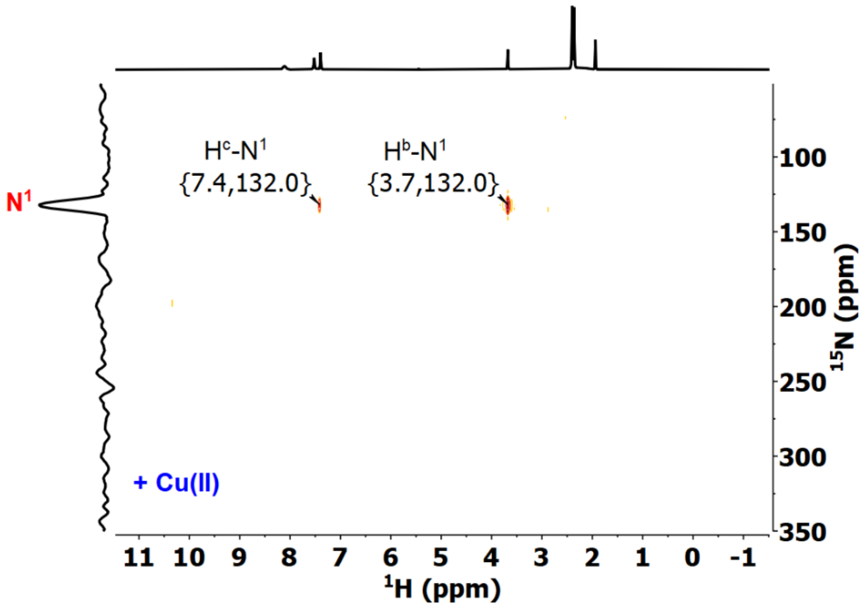

Figure S39. 2D ^{1}H-^{15}N HMBC spectrum of 62.0 mM **5-$^{15}N_2$** in CD_3CN with 5.00 mol% $Cu(OAc)_2 \cdot H_2O$. Spectra acquired 5 minutes after the addition of the catalyst. Correlations annotated. Referenced to CD_3CN, ^{1}H = 1.94 ppm, ^{15}N = 245 ppm. Acquired on a AVIIIHD Nanobay400 NMR spectrometer, NS = 2, RG = 211.5, D1 = 1.8.

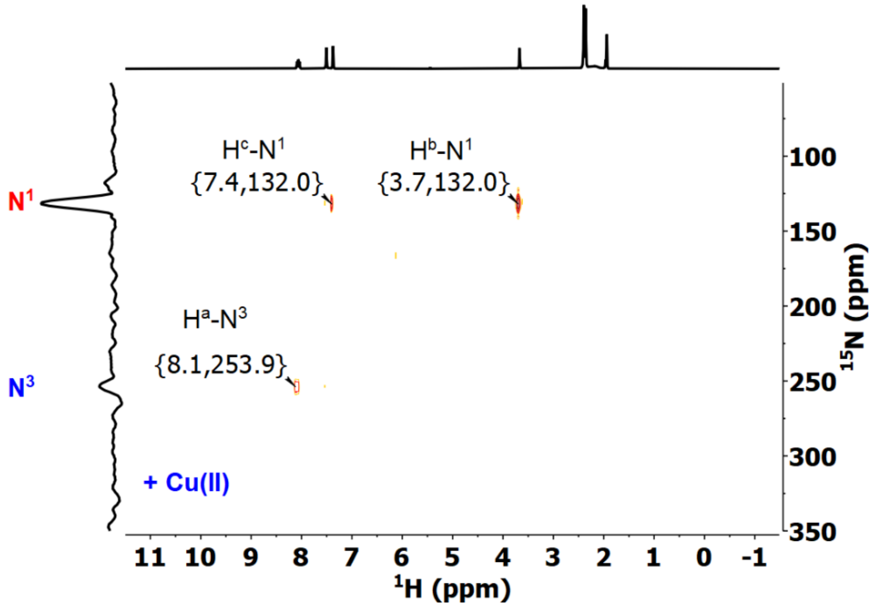

Figure S40. 2D ^{1}H-^{15}N HMBC spectrum of 62.0 mM **5-$^{15}N_2$** in CD_3CN with 5.00 mol% $Cu(OAc)_2 \cdot H_2O$, showing the reappearance of N^3. Spectra acquired 20 minutes after the

addition of the catalyst. Correlations annotated. Referenced to CD_3CN, ^1H = 1.94 ppm, ^{15}N = 245 ppm. Acquired on a AVIIIHD Nanobay 400 NMR spectrometer, NS = 2, RG = 211.5, D1 = 1.8.

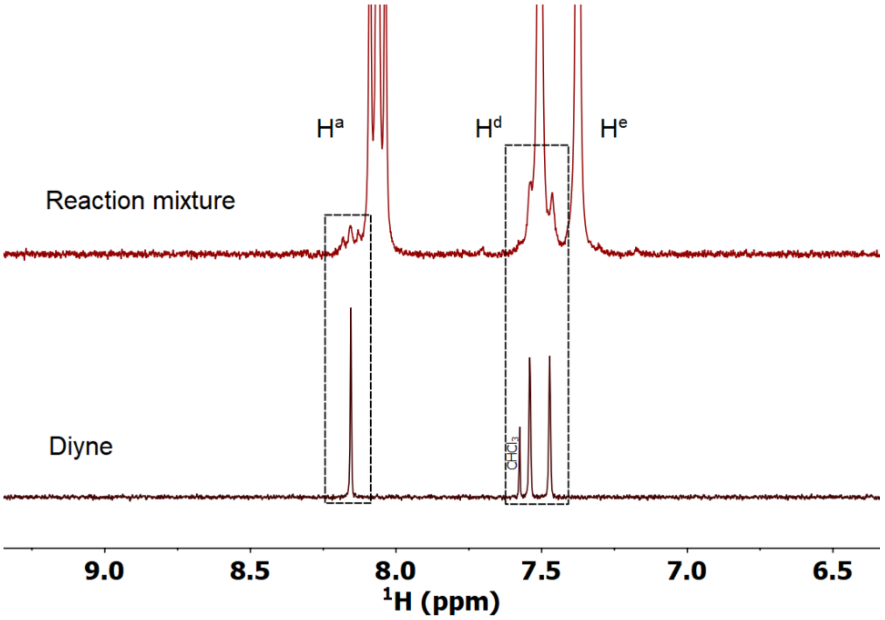

Figure S41. Stacked ^1H spectrum. (Top) 62.0 mM **5-^{15}N$_2$** in CD_3CN with 5.00 mol% $Cu(OAc)_2{\cdot}H_2O$ showing the formation of diyne **15-^{15}N$_4$**. Spectra acquired 20 minutes after the addition of the catalyst. Resonances annotated. Referenced to CD_3CN, ^1H = 1.94 ppm. Acquired on a AVIIIHD Nanobay400 NMR spectrometer, NS = 4, RG = 211.5, D1 = 1. (Bottom) Diyne **15** in CD_3CN. Referenced to CD_3CN, ^1H = 1.94 ppm. Acquired on a AVIIIHD Nanobay400 NMR spectrometer, NS = 32, RG = 211.5, D1 = 2.

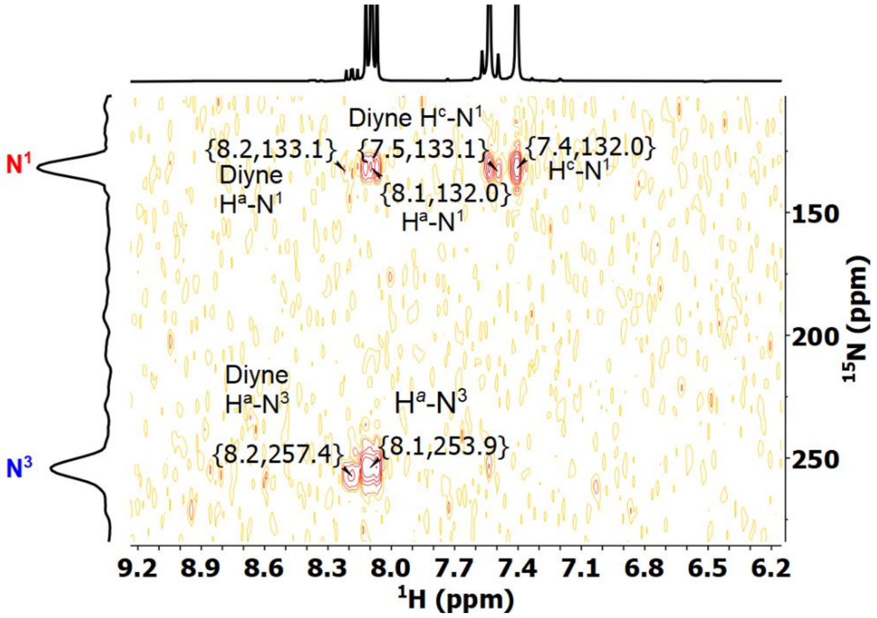

Figure S42. 2D ^{1}H ^{15}N HMBC spectrum of 62.0 mM **5-^{15}N$_2$** in CD_3CN with 5.00 mol% $Cu(OAc)_2 \cdot H_2O$, showing the correlations of $^{15}N_4$ diyne **15-^{15}N$_4$**. Spectra acquired 19 hours after the addition of the catalyst. Correlations annotated. Referenced to CD_3CN, ^{1}H = 1.94 ppm, ^{15}N = 245 ppm. Acquired on a AVIIIHD Nanobay400 NMR spectrometer, NS = 2, RG = 211.5, D1 = 1.8.

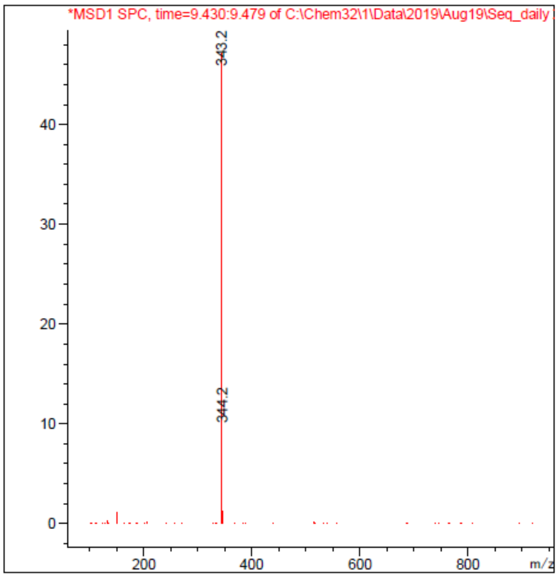

Figure S43. LC-MS spectrum of the reaction mixture, showing the presence of **15-^{15}N$_4$** that is formed in the reaction between **5-^{15}N$_2$** $Cu(OAc)_2 \cdot H_2O$. $C_{22}H_{19}{}^{15}N_4{}^+$ $[M+H]^+$ calculated 343.15.

3.17 ^{13}C and ^{1}H VT-NMR analysis of compound 5-$^{13}C_2$-$^{15}N_2$ in presence of 5 mol% $Cu(OAc)_2 \cdot H_2O$

The effect of paramagnetic Cu(II) on the alkyne of **5-^{13}C$_2$-^{15}N$_2$** was studied. To a glass vial was added **5-^{13}C$_2$-^{15}N$_2$** (3.24 mg, 174.18 g·mol^{-1}, 18.6 µmol, 1.00 equiv) and 300 µL CD_3CN, reaching a concentration of 62.0 mM. 125 µL of this mixture was transferred to an NMR tube and 375 µL CD_3CN was added, reaching a concentration of 15.5 mM. This reference sample was capped, inserted into a spinner, and lowered into the magnet. The probe was then cooled to 233 K with liquid nitrogen, maintaining a shim coil temperature of >278 K. When the desired temperature was reached and stable, the reference sample was locked, tuned and matched, and shimmed. ^{1}H and ^{13}C were acquired at 233, 253, 263, 273, and 300 K, shimming at each temperature interval. Thereafter, the probe was cooled back down to 233 K with the reference sample in place. 125 µL 62.0 mM **5-^{13}C$_2$-^{15}N$_2$** in CD_3CN, and 125 µL CD_3CN was added to an NMR tube, reaching a concentration of 31 mM. The NMR tube was chilled to –40 °C in a $CO_{2(s)}/CH_3CN$ cooling bath. To the chilled NMR tube was then added freshly prepared and pre-pipetted and chilled 250 µL 1.55 mM $Cu(OAc)_2 \cdot H_2O$ (3.10 mg, 199.65 g·mol^{-1} 15.5 µmol

in 1.00 mL CD_3CN), reaching a final reagent concentration of 15.5 mM and 775 µM of catalyst. The sample was shaken vigorously and immediately lowered into the magnet to start the measurement. The sample was locked, tuned and matched, and shimmed. ^1H, and ^{13}C and were acquired at 233, 253, 263, 273, and 300 K, shimming at each temperature interval.

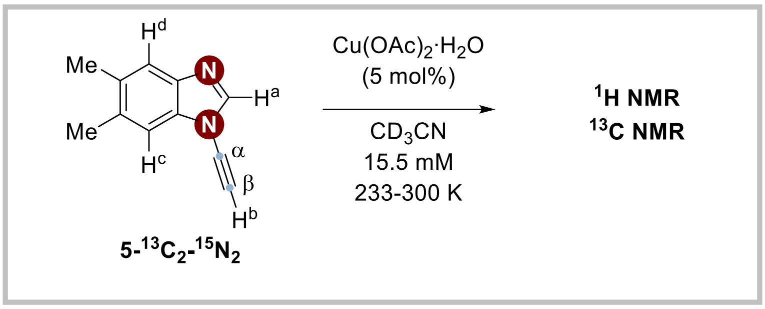

Scheme S15. Addition of 5.00 mol% $Cu(OAc)_2 \cdot H_2O$ to 15.5 mM **5-$^{13}C_2$-$^{15}N_2$** (annotated for NMR) to study its effect on alkyne carbons $^{13}C^{\alpha}$ and $^{13}C^{\beta}$, and surrounding protons at various temperatures.

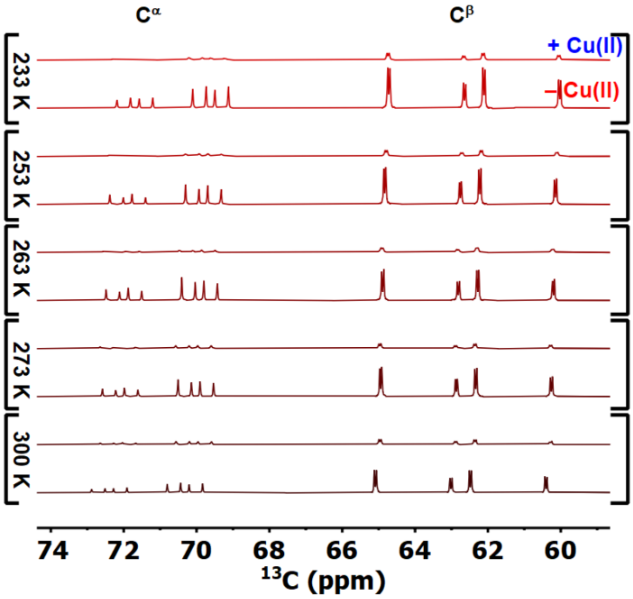

Figure S44. Stacked 1D ^{13}C spectra of 15.5 mM **5-$^{13}C_2$-$^{15}N_2$**, without $Cu(OAc)_2 \cdot H_2O$ (red), and with 5.00 mol% $Cu(OAc)_2 \cdot H_2O$ added (blue) at various temperatures, in CD_3CN, with annotations. Referenced to CD_3CN, δ ^{13}C = 180.26 ppm. Acquired on a AVIIIHD Nanobay400 NMR spectrometer, NS = 64, RG = 211.5, D1 = 0.7, ^1H-decoupler off.

Table S13. Effect on carbon shifts after the addition of 5.00 mol% $Cu(OAc)_2 \cdot H_2O$ to **5-$^{13}C_2$-$^{15}N_2$** to study its effect on alkyne carbons $^{13}C^{\alpha}$ and $^{13}C^{\beta}$.

K	Cu(II)	δ $^{13}C^{\alpha}$ (ppm)	δ $^{13}C^{\beta}$ (ppm)	Δ ppm
	+	70.76	62.40	8.36
233	–	70.66	62.38	8.28
	Δ ppm	0.10	0.02	
	+	70.85	62.45	8.40
253	–	70.85	62.49	8.36
	Δ ppm	0.00	- 0.04	
	+	71.05	62.57	8.48
263	–	70.96	62.55	8.41
	Δ ppm	0.09	0.02	
	+	71.12	62.62	8.50
273	–	71.06	62.60	8.46
	Δ ppm	0.06	0.02	
	+	71.12	62.62	8.50
300	–	71.36	62.75	8.61
	Δ ppm	- 0.024	- 0.13	

Table S14. Effect on J_{CC}, J_{CN} and J_{CH} of Cα and Cβ after the addition of 5.00 mol% $Cu(OAc)_2 \cdot H_2O$ to **5-$^{13}C_2$-$^{15}N_2$**.

K	Cu(II)	Alkyne-carbon	δ (ppm)	J_{CC} (Hz)	J_{CN} (Hz)	J_{CH} (Hz)
233	+	α	70.76	209.22	37.10	61.87
		β	62.4	208.97	5.63	262.64
	–	α	70.66	209.02	37.18	61.41
		β	62.38	208.98	5.76	262.58
253	+	α	70.85	209.05	37.75	61.81
		β	62.45	208.89	5.63	262.41
	–	α	70.85	208.95	37.18	61.19
		β	62.49	208.95	5.69	262.60
263	+	α	71.05	208.84	37.15	60.83
		β	62.57	209.03	5.63	262.61
	–	α	70.96	209.05	37.11	61.02
		β	62.55	209.06	5.81	262.49

273	+	α	71.12	208.82	36.99	60.81
		β	62.62	208.75	5.66	262.40
	−	α	71.06	208.85	37.02	60.86
		β	62.6	208.88	5.65	262.39
300	+	α	71.12	208.85	36.86	60.95
		β	62.62	208.85	5.62	262.40
	−	α	71.36	208.75	37.01	60.43
		β	62.75	208.69	5.61	262.28

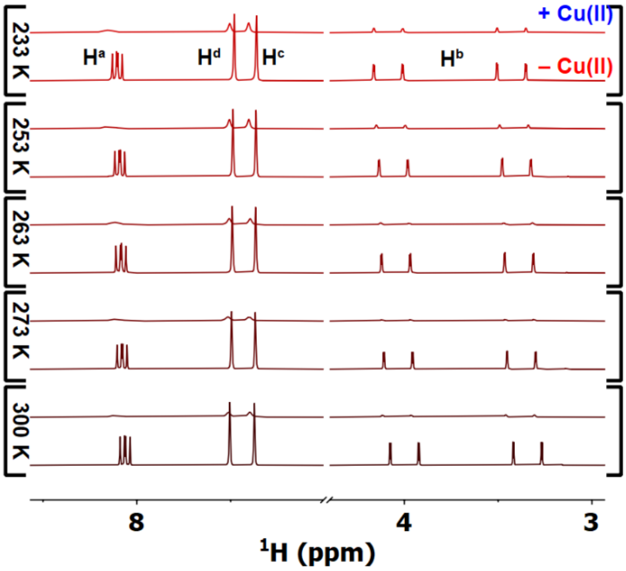

Figure S45. Stacked 1D ^{1}H spectra of 15.5 mM **5-$^{13}C_2$-$^{15}N_2$**, without $Cu(OAc)_2{\cdot}H_2O$ (red), and with 5 mol% $Cu(OAc)_2{\cdot}H_2O$ added (blue) at various temperatures, in CD_3CN, with annotations. Referenced to CD_3CN, ^{1}H = 1.94 ppm. Acquired on a AVIIIHD Nanobay400 NMR spectrometer, NS = 16, RG = 211.5, D1 = 1.0.

3.18 ^{1}H-NMR Analysis of compound 5 in presence of 5 mol% $[(CH_3CN)_4Cu]PF_6$

The effect of diamagnetic Cu(I) on **5** was studied. To a glass vial was added **5** (21.1 mg, 170.22 g·mol^{-1}, 124 µmol) and 1.00 mL CD_3CN, reaching a concentration of 124 mM. 250 µL of this mixture was transferred to an NMR tube and 250 µL CD_3CN was added, reaching a concentration of 62 mM. This reference sample was capped, inserted into a standardbore spinner, and lowered into the magnet at 300 K. The reference sample was locked, tuned and matched, and shimmed. ^{1}H was acquired at 300 K. Thereafter, the reference sample was

removed. 250 µL 124 mM **5** in CD_3CN, and 225 µL CD_3CN was added to an NMR tube, to this was then added freshly prepared and pre-pipetted 25 µL 62 mM $[(CH_3CN)_4Cu]PF_6$ (23.1 mg, 372.72 g·mol^{-1} in 1.00 mL CD_3CN, requiring sonication), reaching a final reagent concentration of 62 mM and 5.00 mol% of catalyst. The sample was shaken vigorously and immediately lowered into the magnet to start the measurement. The sample was locked, tuned and matched, and shimmed. ^1H spectra were acquired immediately using the *multi_zgvd2* command that included a *topshim_1dfast* shim after each acquisition. Experiments had a fixed delay of 60 seconds.

.

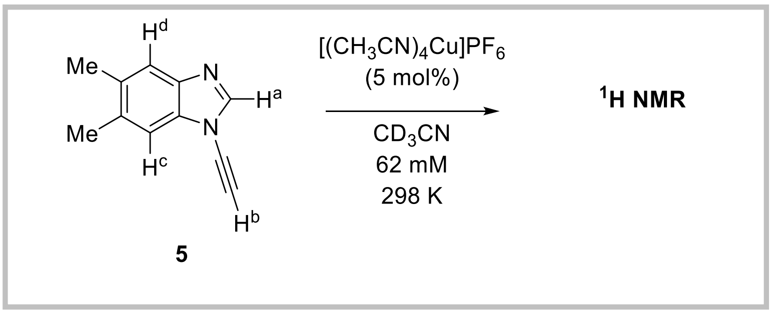

Scheme S16. Addition of 5.00 mol% $[(CH_3CN)_4Cu]PF_6$ to 62 mM **5** (annotated for NMR) to study its effect on ^1H resonances.

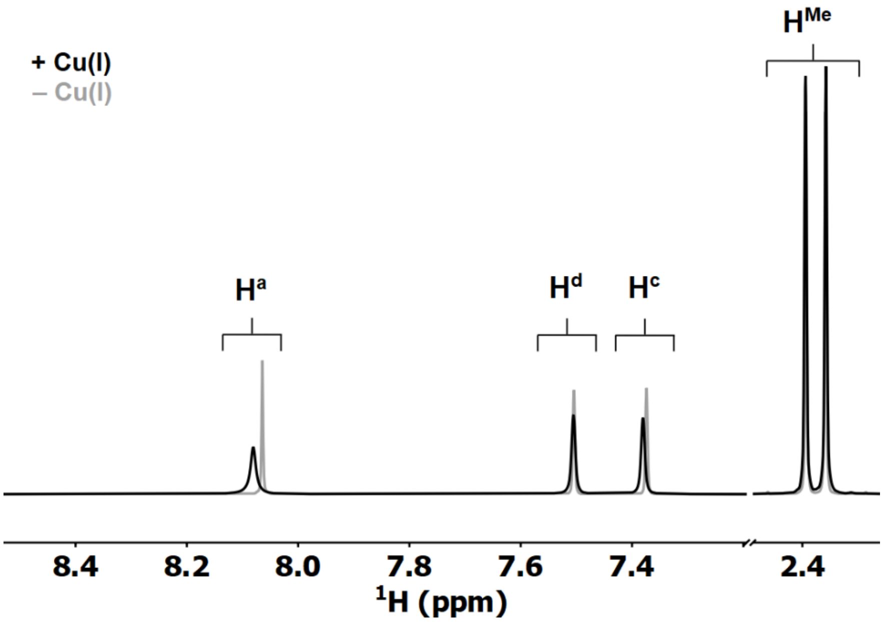

Figure S46. Superimposed 1D ^1H spectra of 62.0 mM ynamine **5**, with 5.00 mol% $[(CH_3CN)_4Cu]PF_6$. This effect was stable over time (recorded for 1 h). Referenced to CD_3CN,

^{1}H = 1.94 ppm. Acquired on an AVII+ 600MHz spectrometer, NS = 4, RG = 128, D1 = 5, TE = 298 K, in CD_3CN, with annotations.

3.19 VT-NMR analysis of compound 5-$^{13}C_2$-$^{15}N_2$ in presence of[$(CH_3CN)_4Cu$]PF_6

The effect of diamagnetic Cu(I) on **5-$^{13}C_2$-$^{15}N_2$** was studied. To a glass vial was added **5-$^{13}C_2$-$^{15}N_2$** (3.24 mg, 174.18 g·mol^{-1}, 18.6 µmol, 1.00 equiv) and 300 µL CD_3CN, reaching a concentration of 62.0 mM. 125 µL of this mixture was transferred to an NMR tube and 375 µL CD_3CN was added, reaching a concentration of 15.5 mM. This reference sample was capped, inserted into a ceramic standardbore spinner, and lowered into the magnet. The probe was then cooled to 233 K with liquid nitrogen, maintaining a shim coil temperature of >278 K. When the desired temperature was reached and stable, the reference sample was locked, tuned and matched, and shimmed. ^{1}H, $^{13}C\{^{1}H\}$, ^{13}C and ^{1}H-^{15}N HMBC were acquired at 233, 253, 263, 273, and 300 K, shimming at each temperature interval. Thereafter, the probe was cooled back down to 233 K with the reference sample in place. 125 µL 62.0 mM **5-$^{13}C_2$-$^{15}N_2$** in CD_3CN, and 125 µL CD_3CN was added to an NMR tube, reaching a concentration of 31 mM. The NMR tube was chilled to –40 °C in a $CO_{2(s)}$/CH_3CN cooling bath. To the chilled NMR tube was then added freshly prepared and pre-pipetted and chilled 250 µL 31 mM [$(CH_3CN)_4Cu$]PF_6 (5.8 mg, 372.72 g·mol^{-1} in 500 µL CD_3CN), reaching a final reagent concentration of 15.5 mM and 15.5 mM of catalyst. The sample was shaken vigorously and immediately lowered into the magnet to start the measurement. The sample was locked, tuned and matched, and shimmed. ^{1}H, $^{13}C\{^{1}H\}$, ^{13}C and ^{1}H-^{15}N HMBC were acquired at 233, 253, 263, 273, and 300 K, shimming at each temperature interval.

Prior to the addition of Cu(I) at 233 K, both nitrogen signals for N^1 and N^3 were observed at all temperatures. Thereafter, different ^{1}H-^{15}N correlations were found at 233 K. ^{1}H shifts experienced a downfield, isotropic shift. Line-shape broadening affects H^a & H^d more than H^c. In addition, acquired $^{13}C\{^{1}H\}$ and ^{13}C spectra found an up field shift for C^{α}, and a downfield shift was observed for C^{β}.

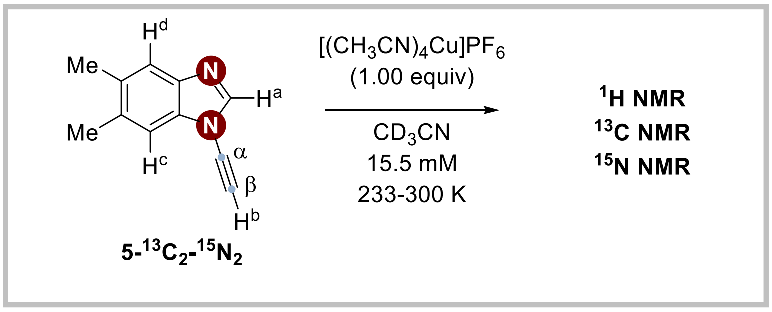

Scheme S17. Addition of 1.00 equiv $[(CH_3CN)_4Cu]PF_6$ to 15.5 mM **5-$^{13}C_2$-$^{15}N_2$** (annotated for NMR) to study its effect on alkyne carbons $^{13}C^{\alpha}$ and $^{13}C^{\beta}$, and surrounding protons at various temperatures.

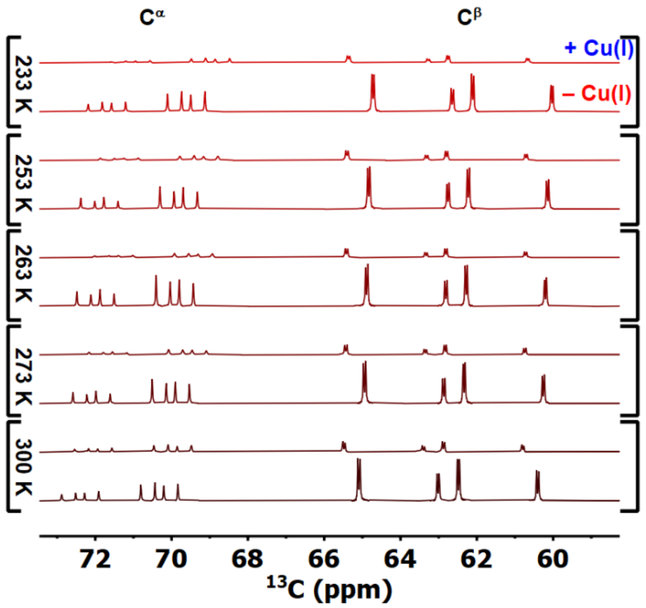

Figure S47. Stacked 1D ^{13}C spectra of 15.5 mM **5-$^{13}C_2$-$^{15}N_2$**, without $[(CH_3CN)_4Cu]PF_6$ (red), and with 1.00 equiv $[(CH_3CN)_4Cu]PF_6$ added (blue) at various temperatures, in CD_3CN, with annotations. Referenced to CD_3CN, δ ^{13}C = 180.26 ppm. Acquired on a AVIIIHD Nanobay400 NMR spectrometer, NS = 64, RG = 211.5, D1 = 0.7, ^{1}H-decoupler off.

Table S15. Effect on carbon shifts after the addition of 1.00 equiv $[(CH_3CN)_4Cu]PF_6$ to **5-$^{13}C_2$-$^{15}N_2$** to study its effect on alkyne carbons $^{13}C^{\alpha}$ and $^{13}C^{\beta}$.

K	Cu(I)	δ $^{13}C^{\alpha}$ (ppm)	δ $^{13}C^{\beta}$ (ppm)	Δ ppm
	+	69.74	62.73	7.01
233	–	70.92	62.61	8.31
	Δ ppm	1.18	-0.12	/
	+	69.84	62.57	7.27
253	–	71.05	62.65	8.4
	Δ ppm	1.21	0.08	/
	+	69.89	62.49	7.4
263	–	70.33	61.96	8.37
	Δ ppm	0.44	-0.53	/
273	+	69.94	62.4	7.54
	–	71.19	62.73	8.46

	Δ ppm	1.25	0.33	/
	+	70.06	62.18	7.88
300	–	71.4	62.79	8.61
	Δ ppm	1.34	0.61	/

Table S16. Effect on J_{CC}, J_{CN} and J_{CH} of Cα and Cβ after the addition of 1.00 equiv $[(CH_3CN)_4Cu]PF_6$ to **5-$^{13}C_2$-$^{15}N_2$**.

K	Cu(I)	Alkyne-carbon	δ (ppm)	J_{CC} (Hz)	J_{CN} (Hz)	J_{CH} (Hz)
233	+	α	69.74	210.09	37.59	63.13
		β	62.73	210.09	6.13	261.95
	–	α	70.92	209.23	37.28	61.51
		β	62.64	209.00	5.76	262.58
253	+	α	69.84	210.00	37.56	62.23
		β	62.57	209.83	6.06	261.97
	–	α	71.05	208.95	37.18	61.20
		β	62.68	208.94	5.69	262.60
263	+	α	69.89	209.89	37.36	62.21
		β	62.49	209.89	5.97	262.12
	–	α	70.38	209.50	23.42	98.28
		β	61.96	209.04	5.81	262.49
273	+	α	69.94	209.62	37.42	61.91
		β	62.40	209.74	5.93	262.14
	–	α	71.19	208.86	37.03	60.86
		β	62.73	208.88	5.66	262.37
300	+	α	70.06	209.62	37.28	61.38
		β	62.18	209.26	5.85	262.10
	–	α	71.40	208.73	37.02	60.46
		β	62.79	208.70	5.62	262.27

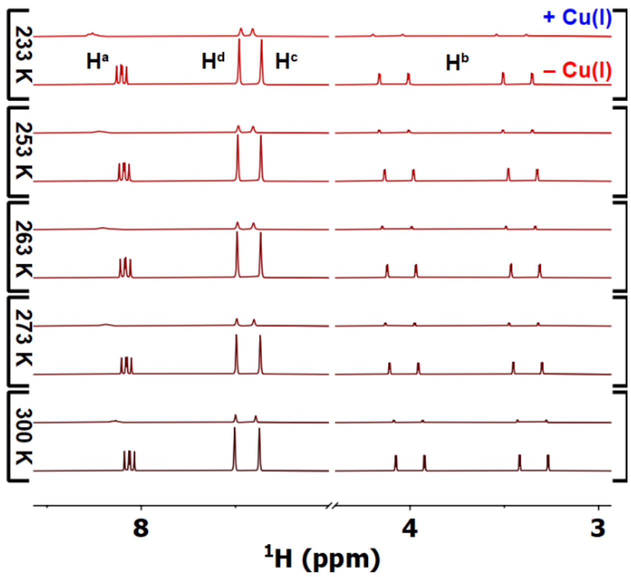

Figure S48. Stacked 1D ^{1}H spectra of 15.5 mM **5-[13]C$_2$-[15]N$_2$**, without [(CH$_3$CN)$_4$Cu]PF$_6$ (red), and with 1.00 equiv. [(CH$_3$CN)$_4$Cu]PF$_6$ added (blue) at various temperatures, in CD$_3$CN, with annotations. Referenced to CD$_3$CN, ^{1}H = 1.94 ppm. Acquired on a AVIIIHD Nanobay400 NMR spectrometer, NS = 16, RG = 211.5, D1 = 1.0.

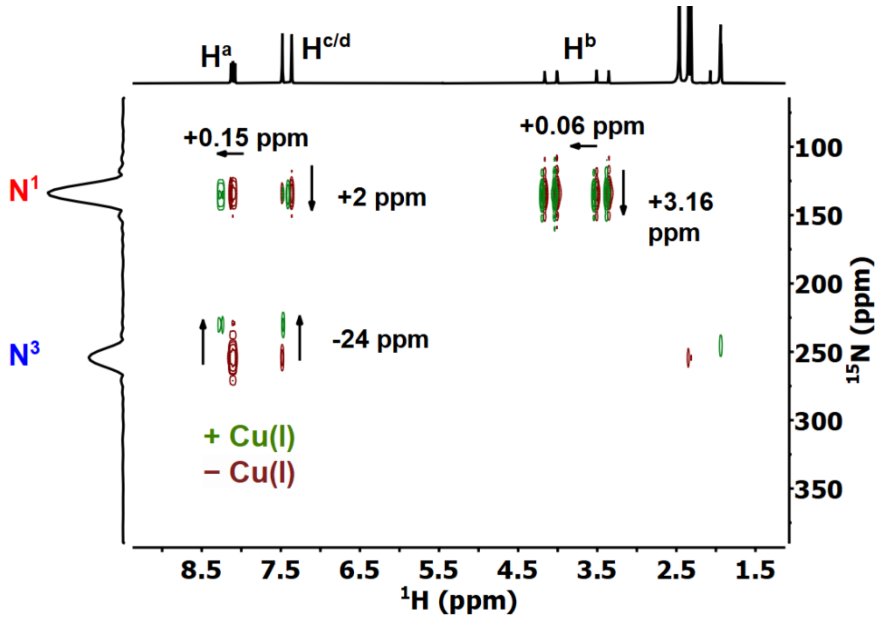

Figure S49. 2D ^{1}H ^{15}N HMBC spectrum of 15.5 mM **5-[13]C$_2$-[15]N$_2$** in CD$_3$CN at 233 K. Without (red), and with 1.00 equiv [(CH$_3$CN)$_4$Cu]PF$_6$ added (green). Correlations annotated. Referenced to CD$_3$CN, ^{1}H = 1.94 ppm, ^{15}N = 245 ppm. Acquired on a AVIIIHD Nanobay400 NMR spectrometer, NS = 2, RG = 211.5, D1 = 1.8.

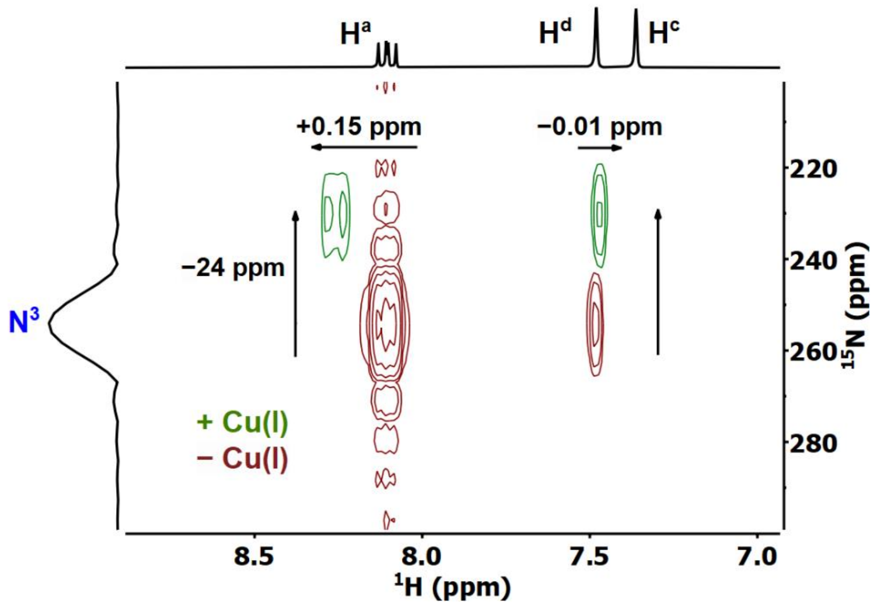

Figure S50. 2D ^{1}H ^{15}N HMBC spectrum of 15.5 mM **5-$^{13}C_2$-$^{15}N_2$** in CD_3CN at 233 K. Expansion plot, focussed on aromatic proton correlations. Without (red), and with 1.00 equiv $[(CH_3CN)_4Cu]PF_6$ added (green). Correlations annotated. Referenced to CD_3CN, ^{1}H = 1.94 ppm, ^{15}N = 245 ppm. Acquired on a AVIIIHD Nanobay400 NMR spectrometer, NS = 2, RG = 211.5, D1 = 1.8.

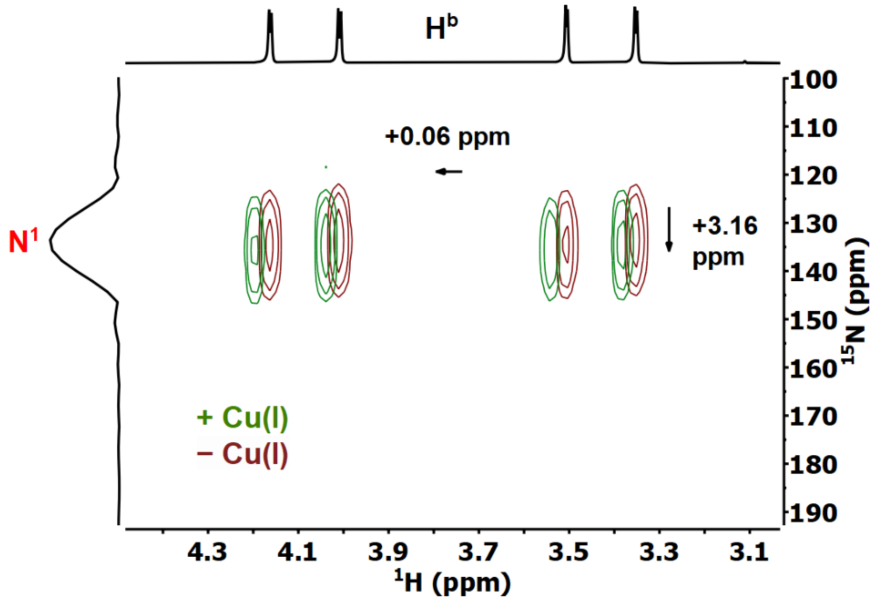

Figure S51. 2D ^{1}H ^{15}N HMBC spectrum of 15.5 mM **5-$^{13}C_2$-$^{15}N_2$** in CD_3CN at 233 K. Expansion plot, focussed on alkyne proton correlations. Without (red), and with 1.00 equiv $[(CH_3CN)_4Cu]PF_6$ added (green). Correlations annotated. Referenced to CD_3CN, ^{1}H = 1.94 ppm, ^{15}N = 245 ppm. Acquired on a AVIIIHD Nanobay400 NMR spectrometer, NS = 2, RG = 211.5, D1 = 1.8.

Table S17. Chemical shift data comparing ^{1}H and ^{15}N shifts with, and without the addition of $[(CH_3CN)_4Cu]PF_6$.

Cu(I)	^{15}N	δ ^{15}N	δ ^{1}H (ppm)			
			H^a	H^c	H^d	H^b
+	**N1**	135.30	8.24	7.48	7.41	3.81
	N3	229.94	8.24	7.48	/	/
–	**N1**	133.14	8.11	7.48	7.37	3.76
	N3	253.60	8.11	7.48	7.37	/

Initially, the experiment was performed with 5.00 mol% $[(CH_3CN)_4Cu]PF_6$. To a glass vial was added **5-$^{13}C_2$-$^{15}N_2$** (3.24 mg, 174.18 g·mol^{-1}, 18.6 µmol, 1.00 equiv) and 300 µL CD_3CN, reaching a concentration of 62.0 mM. 125 µL of this mixture was transferred to an NMR tube and 375 µL CD_3CN was added, reaching a concentration of 15.5 mM. This reference sample was capped, inserted into a ceramic standardbore spinner, and lowered into the magnet. The probe was then cooled to 233 K with liquid nitrogen, maintaining a shim coil temperature of >278 K. When the desired temperature was reached and stable, the reference sample was locked, tuned and matched, and shimmed. ^{1}H, $^{13}C\{^{1}H\}$, ^{13}C and ^{1}H-^{15}N HMBC were acquired at 233, 253, 263, 273, and 300 K, shimming at each temperature interval. Thereafter, the probe was cooled back down to 233 K with the reference sample in place. 125 µL 62.0 mM **5-$^{13}C_2$-$^{15}N_2$** in CD_3CN, and 125 µL CD_3CN was added to an NMR tube, reaching a concentration of 31 mM. The NMR tube was chilled to –40 °C in a $CO_{2(s)}$/CH_3CN cooling bath. To the chilled NMR tube was then added freshly prepared and pre-pipetted and chilled 250 µL 1.55 mM $[(CH_3CN)_4Cu]PF_6$ (2.90 mg, 372.72 g·mol^{-1} in 5.00 mL CD_3CN), reaching a final reagent concentration of 15.5 mM and 775 µM of catalyst. The sample was shaken vigorously and immediately lowered into the magnet to start the measurement. The sample was locked, tuned and matched, and shimmed. ^{1}H, $^{13}C\{^{1}H\}$, ^{13}C and ^{1}H-^{15}N HMBC were acquired at 233, 253, 263, 273, and 300 K, shimming at each temperature interval.

Acquired ^{13}C spectra found a minor upfield shift for C^{α}, and a downfield shift was observed for C^{β}.

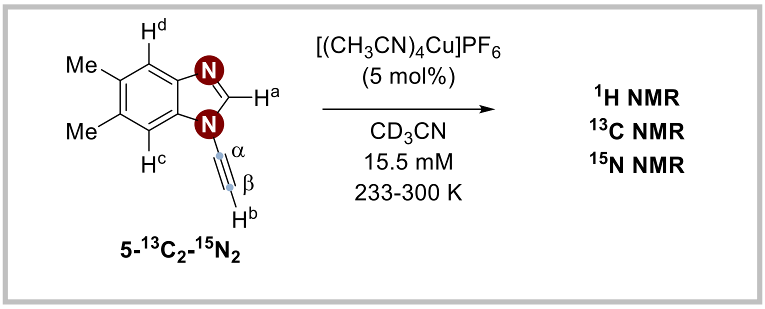

Scheme S18. Addition of 5.00 mol% $[(CH_3CN)_4Cu]PF_6$ to 15.5 mM **5-$^{13}C_2$-$^{15}N_2$** (annotated for NMR) to study its effect on alkyne carbons $^{13}C^{\alpha}$ and $^{13}C^{\beta}$, and surrounding protons at various temperatures.

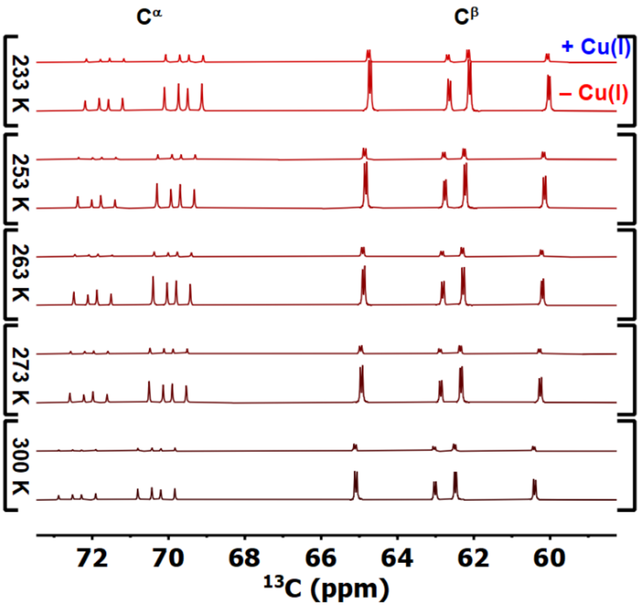

Figure S52. Stacked 1D ^{13}C spectra of 15.5 mM **5-$^{13}C_2$-$^{15}N_2$**, without $[(CH_3CN)_4Cu]PF_6$ (red), and with 5.00 mol% $[(CH_3CN)_4Cu]PF_6$ added (blue) at various temperatures, in CD_3CN, with annotations. Referenced to CD_3CN, δ ^{13}C = 180.26 ppm. Acquired on a AVIIIHD Nanobay400 NMR spectrometer, NS = 64, RG = 211.5, D1 = 0.7, ^{1}H-decoupler off.

Table S18. Effect on carbon shifts after the addition of 5.00 mol% $[(CH_3CN)_4Cu]PF_6$ to **5-$^{13}C_2$-$^{15}N_2$** to study its effect on alkyne carbons $^{13}C^{\alpha}$ and $^{13}C^{\beta}$.

K	Cu(I)	δ $^{13}C^{\alpha}$ (ppm)	δ $^{13}C^{\beta}$ (ppm)	Δ ppm
	+	70.62	62.41	8.20
233	–	70.92	62.61	8.31
	Δ ppm	0.30	0.20	/
253	+	70.62	62.31	8.31
	–	71.05	62.65	8.40

	Δ ppm	0.43	0.34	/
	+	70.63	62.27	8.36
263	–	70.96	62.55	8.37
	Δ ppm	0.33	0.28	/
	+	70.64	62.22	8.09
273	–	71.19	62.73	8.46
	Δ ppm	0.55	0.51	/
	+	70.67	62.09	8.58
300	–	71.4	62.79	8.61
	Δ ppm	0.73	0.70	/

Table S19. Effect on J_{CC}, J_{CN} and J_{CH} of Cα and Cβ after the addition of 5.00 mol% $[(CH_3CN)_4Cu]PF_6$ to **5-$^{13}C_2$-$^{15}N_2$**.

K	Cu(I)	Alkyne-carbon	δ (ppm)	J_{CC} (Hz)	J_{CN} (Hz)	J_{CH} (Hz)
233	+	α	70.62	209.62	37.28	61.46
		β	62.41	209.01	5.71	262.43
	–	α	70.92	209.23	37.28	61.51
		β	62.64	209.00	5.76	262.58
253	+	α	70.62	209.04	37.08	61.17
		β	62.31	208.99	5.72	262.47
	–	α	71.05	208.95	37.18	61.20
		β	62.68	208.94	5.69	262.60
263	+	α	70.63	208.85	37.16	61.14
		β	62.27	208.92	5.67	262.41
	–	α	70.96	209.05	37.11	61.02
		β	62.55	209.06	5.81	262.49
273	+	α	70.64	209.10	37.07	60.92
		β	62.37	208.93	5.69	262.37
	–	α	71.19	208.86	37.03	60.86
		β	62.73	208.88	5.66	262.37
300	+	α	70.67	208.70	36.96	60.51
		β	62.09	208.76	5.63	262.19
	–	α	71.40	208.73	37.02	60.46
		β	62.79	208.70	5.62	262.27

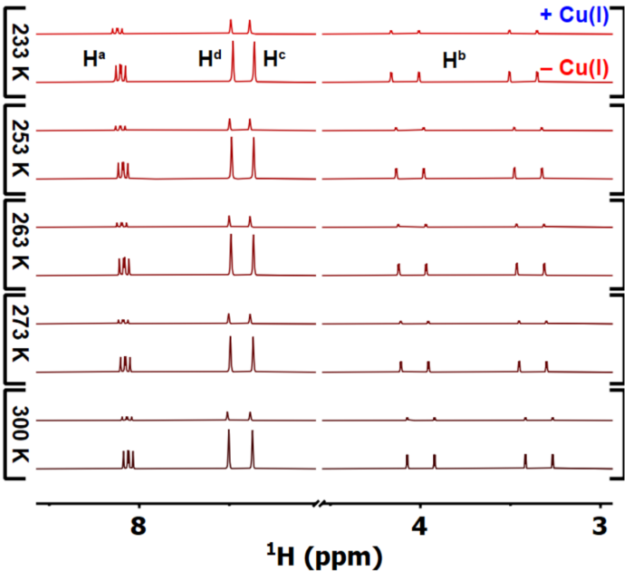

Figure S53. Stacked 1D ^{1}H spectra of 15.5 mM **5-$^{13}C_2$-$^{15}N_2$**, without $[(CH_3CN)_4Cu]PF_6$ (red), and with 5.00 mol% $[(CH_3CN)_4Cu]PF_6$ added (blue) at various temperatures, in CD_3CN, with annotations. Referenced to CD_3CN, ^{1}H = 1.94 ppm. Acquired on a AVIIIHD Nanobay400 NMR spectrometer, NS = 16, RG = 211.5, D1 = 1.0.

3.20 <u>^{1}H and ^{13}C NMR analysis of compound S10 in presence of 1.00 equiv $[(CH_3CN)_4Cu]PF_6$</u>

The effect of diamagnetic Cu(I) on indole ynamine-TIPS **S10** was studied. To a glass vial was added on **S10** (5.53 mg, 18.6 µmol, 297.51 g·mol^{-1}, 1.00 equiv) and 300 µL CD_3CN, reaching a concentration of 62.0 mM. 125 µL of this mixture was transferred to an NMR tube and 375 µL CD_3CN was added, reaching a concentration of 15.5 mM. This reference sample was capped and submitted for ^{1}H and $^{13}C\{^{1}H\}$ analysis at 300 K. 125 µL 62.0 mM **S10** in CD_3CN, and 125 µL CD_3CN was added to an NMR tube, reaching a concentration of 31 mM. To this NMR tube was then added freshly prepared 250 µL 31 mM $[(CH_3CN)_4Cu]PF_6$ (5.8 mg, 372.72 g·mol^{-1} in 500 µL CD_3CN), reaching a final reagent concentration of 15.5 mM and 15.5 mM of catalyst. The sample was shaken vigorously and submitted for ^{1}H and $^{13}C\{^{1}H\}$ at 300 K.

Upon the addition of $[(CH_3CN)_4Cu]PF_6$, ^{1}H signals did not experience a downfield, isotropic shift. Line-shape broadening did affect $H^{a\text{-}g}$. $^{13}C\{^{1}H\}$ shifts remained unchanged. Additionally, the signal intensity for C^{α}, and C^{β} remained unaffected also.

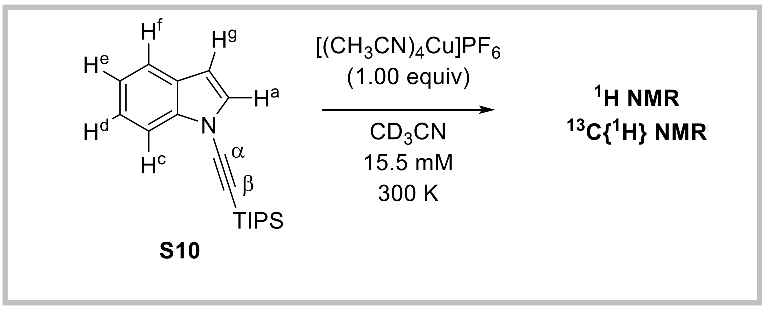

Scheme S19. Addition of 1.00 equiv $[(CH_3CN)_4Cu]PF_6$ to 15.5 mM **S10** (annotated for NMR) to study its effect on alkyne carbons C^α and C^β, and surrounding protons at various temperatures.

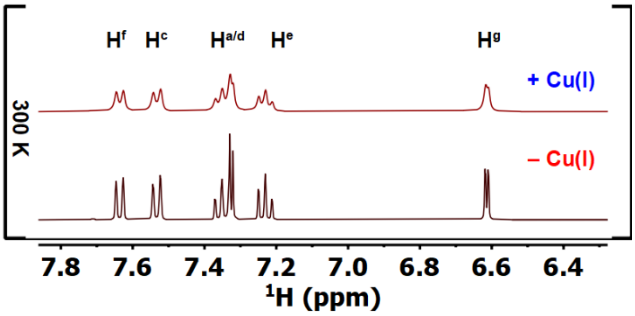

Figure S54. Stacked 1D ^{1}H spectra of 15.5 mM **S10**, without $[(CH_3CN)_4Cu]PF_6$ (red), and with 1.00 equiv $[(CH_3CN)_4Cu]PF_6$ added (blue). Acquired at 300 K, in CD_3CN, with annotations. Referenced to CD_3CN, ^{1}H = 1.94 ppm. Acquired on a AVIIIHD Nanobay400 NMR spectrometer, NS = 16, RG = 211.5, D1 = 1.0.

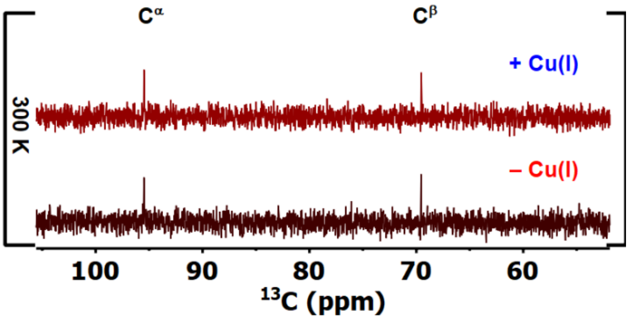

Figure S55. Stacked 1D ^{13}C{^{1}H} spectra of 15.5 mM **S10**, without $[(CH_3CN)_4Cu]PF_6$ (red), and with 1.00 equiv $[(CH_3CN)_4Cu]PF_6$ added (blue). Acquired at 300 K, in CD_3CN, with annotations. Referenced to CD_3CN, δ ^{13}C = 180.26 ppm. Acquired on a AVIIIHD Nanobay400 NMR spectrometer, NS = 8192, RG = 211.5, D1 = 0.7, ^{1}H-decoupler on.

Table S20. There is no significant effect on carbon shifts of alkyne carbons $^{13}C^{\alpha}$ and $^{13}C^{\beta}$ after the addition of 1.00 equiv $[(CH_3CN)_4Cu]PF_6$ to 15.5 mM **S10**.

K	Cu(I)	δ $^{13}C^{\alpha}$ (ppm)	δ $^{13}C^{\beta}$ (ppm)
300	+	95.47	69.54
	–	95.48	69.54

3.21 ^{1}H-^{15}N HMBC VT-NMR analysis of compound 8 in presence of 1.00 equiv $[(CH_3CN)_4Cu]PF_6$

The effect of diamagnetic Cu(I) on indole **8** was studied at 233 K. To a glass vial was added on indole **8** (2.60 mg, 18.6 µmol, 141.17 g·mol^{-1}, 1.00 equiv) and 300 µL CD_3CN, reaching a concentration of 62.0 mM. 125 µL of this mixture was transferred to an NMR tube and 375 µL CD_3CN was added, reaching a final concentration of 15.5 mM. This reference sample was capped and submitted for ^{1}H and ^{1}H-^{15}N HMBC analysis at 233 K. 125 µL 62.0 mM indole **8** in CD_3CN, and 125 µL CD_3CN was added to an NMR tube, reaching a concentration of 31 mM. To this NMR tube was then added freshly prepared 250 µL 31 mM $[(CH_3CN)_4Cu]PF_6$ (5.80 mg, 372.72 g·mol^{-1} in 500 µL CD_3CN), reaching a final reagent concentration of 15.5 mM and 15.5 mM of catalyst. The sample was shaken vigorously and submitted for ^{1}H and ^{1}H-^{15}N HMBC analysis at 233 K. Upon the addition of $[(CH_3CN)_4Cu]PF_6$, ^{1}H signals did not experience a downfield, isotropic shift. Line-shape broadening did affect $H^{a\text{-}g}$. ^{1}H-^{15}N correlations shifted downfield by $\delta_{^{15}N}$ = 0.8 ppm for N^1-H^a and N^1-H^g. The correlation for N^1-H^b was lost.

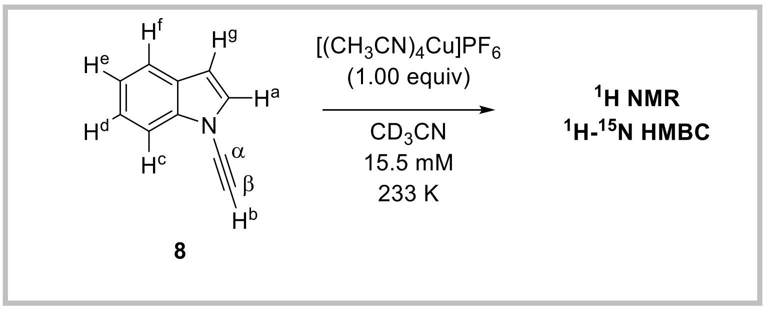

Scheme S20. Addition of 1.00 equiv $[(CH_3CN)_4Cu]PF_6$ to 15.5 mM **8** (annotated for NMR) to study its effect on alkyne carbons C^{α} and C^{β}, and surrounding protons at various temperatures.

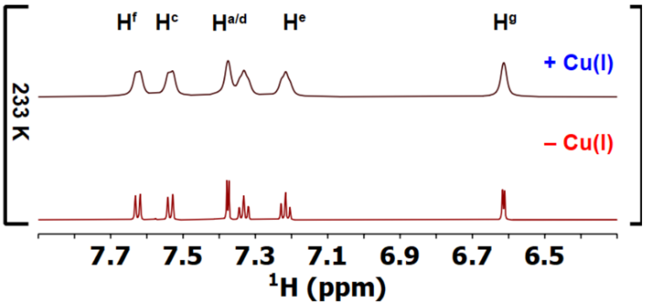

Figure S56. Stacked 1D ^{1}H spectra of 15.5 mM **8**, without $[(CH_3CN)_4Cu]PF_6$ (red), and with 1.00 equiv $[(CH_3CN)_4Cu]PF_6$ added (blue). Acquired at 233 K, in CD_3CN, with annotations. Referenced to CD_3CN, ^{1}H = 1.94 ppm. Acquired on an AVII+ 600MHz spectrometer, NS = 16, RG = 211.5, D1 = 1.0.

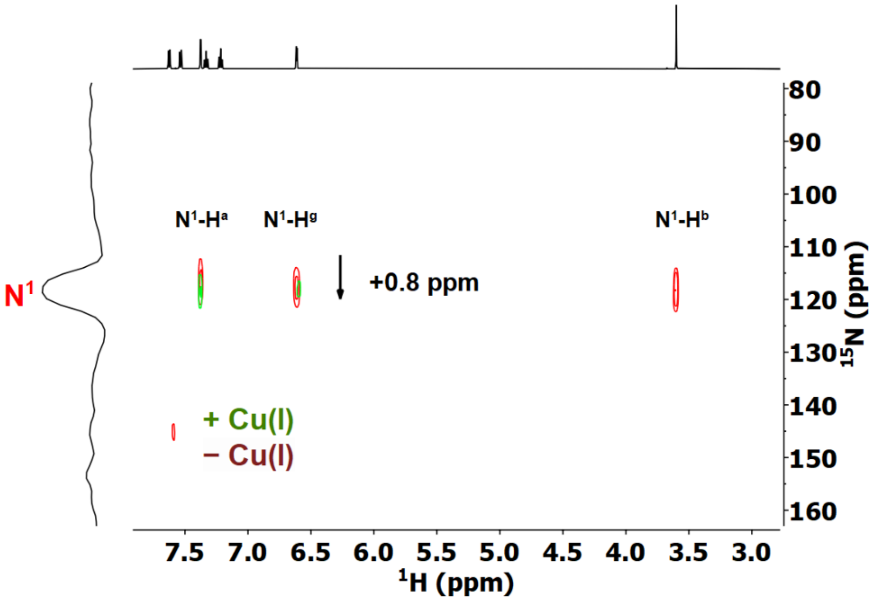

Figure S57. Stacked 2D ^{1}H-^{15}N HMBC spectrum of 15.5 mM indole ynamine **8** in CD_3CN at 233 K. Without (red), and with 1.00 equiv $[(CH_3CN)_4Cu]PF_6$ added (green). 1D corresponds to sample without catalyst. Correlations annotated. Referenced to CD_3CN, ^{1}H = 1.94 ppm, ^{15}N = 245 ppm. Acquired on an AVII+ 600MHz spectrometer, NS = 32, RG = 2050, D1 = 1.8.

3.22 Preliminary ^{1}H NMR monitoring of the ynamine-azide (3+2) cycloaddition reaction in CD_3CN

The effect of paramagnetic Cu(II) on the ynamine-azide (3+2) cycloaddition reaction by means of ^{1}H NMR, using 5.00 mol% $Cu(OAc)_2{\cdot}H_2O$. To a glass vial was added **5** (11.90 mg, 170.22 g·mol^{-1}, 69.8 µmol, 1.00 equiv), and **2** (18.7 mg, 133.15 g·mol^{-1}, 69.8 µmol, 1.00 equiv) and 900 µL CD_3CN, reaching a concentration of 77.5 mM. 400 µL of the 77.5 mM mixture and 100 µL CD_3CN was transferred to a clean and dry NMR tube, reaching a final concentration of 62.0

mM. This reference sample was capped and lowered into the magnet. The reference sample was used to locked, tune, match, and shim the magnet in preparation of the reaction sample. ^{1}H was acquired at 300 K and the sample was ejected. Thereafter, 400 µL of the 77.5 mM **5**/**2** mixture, and 100 µL 15.5 mM $Cu(OAc)_2 \cdot H_2O$ (63.10 mg, 199.65 g·mol^{-1}, 316 µmol, in 1.00 mL CD_3CN) solution was added to an NMR tube, reaching a final concentration of 62.0 mM **5** and **2**, and 5.0 mol% $Cu(OAc)_2 \cdot H_2O$. The sample was shaken vigorously and immediately lowered into the magnet to start the measurement. ^{1}H (Acquired on a AVIIIHD Nanobay400 NMR spectrometer, TE = 300, NS = 8, D1 = 15, RG = 189.8) spectra were acquired immediately using the *multi_zgvd2* command that included a *topshim_1dfast* shim after each acquisition. Experiments had a fixed delay of 60 seconds.

The consumption of **5**-H^b and **2**-H^e, and the formation of **10**-H^e are monitorable, as these resonances are not aromatic (Figure S59). However, the aromatic resonances for **5**, and **10** drift significantly. When the reaction is complete at t = 30 min (**5** is consumed), there is an almost instant downfield ~0.1 ppm drift for **10**-H^a and **10**-H$^{c/d}$ (Figure S58, top left - middle). Water initially displayed a very broad resonance, but when the formation of **10** was complete, line-shape broadening decreased and an upfield shift is seen (Figure S58, top-right).

It was found that at these concentrations it was not feasible to monitor the reaction since the frequency of acquisition was too slow, and information was potentially lost. When using precious labelled materials, and monitoring multiple nuclei, it would be better to reduce the concentration to slow the reaction and create more time for data acquisition.

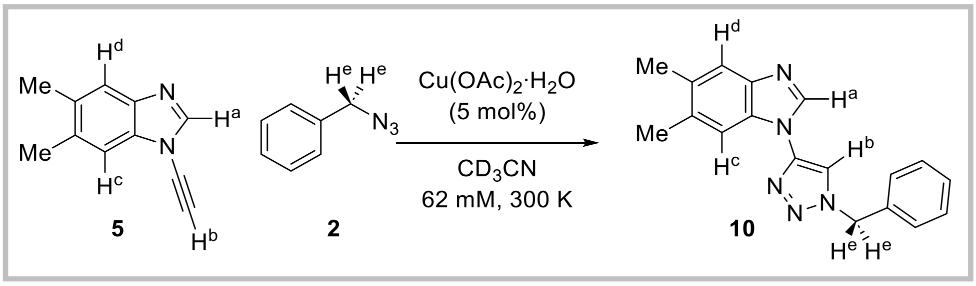

Scheme S21. Monitoring the reaction of 62.0 mM **5** and **2** with 5.00 mol% $Cu(OAc)_2 \cdot H_2O$ (annotated for NMR) in CD_3CN.

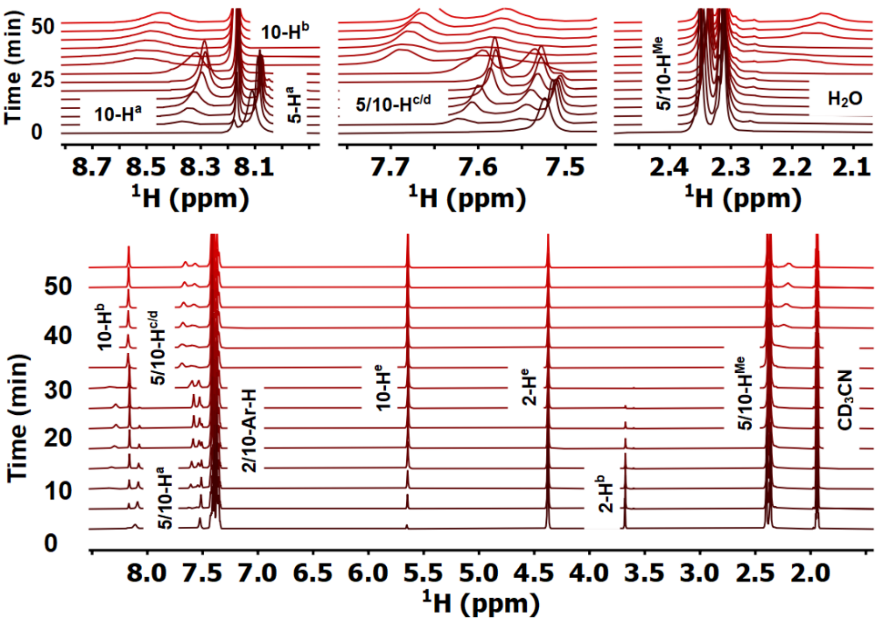

Figure S58. (bottom) Stacked 1D ^{1}H NMR spectra, monitoring the reaction of 62.0 mM **5** and **2** with 5.00 mol% $Cu(OAc)_2{\cdot}H_2O$ in CD_3CN at 300 K. (top) Three expansion plots focussing on the resonances of interest. Referenced to CD_3CN, ^{1}H = 1.94 ppm. Acquired on a AVIIIHD Nanobay400 NMR spectrometer at 300 K, NS = 8, RG = 189.8, D1 = 15. With annotations.

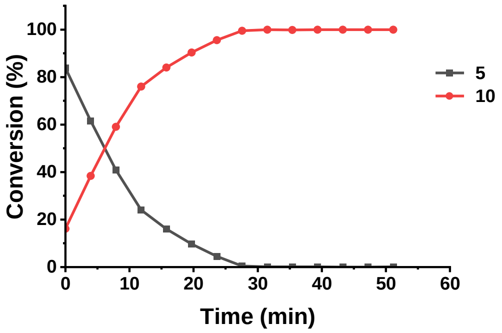

Figure S59. Conversion of **5** and **10** for the ynamine-azide (3+2) cycloaddition reaction as monitored by ^{1}H NMR. Conversion calculated by total sum of peak area for **5**-H^{b} and **10**-H^{e}. n = 1.

3.23 ^{1}H and ^{1}H-^{15}N HMBC monitoring of the ynamine-azide (3+2) cycloaddition reaction in CD_3CN

The (3+2) cycloaddition was monitored with **5-$^{15}N_2$**, **2-$^{15}N_2$** and 5.00 mol% $Cu(OAc)_2{\cdot}H_2O$ using ^{1}H-^{15}N HMBC. To a glass vial was added **5-$^{15}N_2$** (1.35 mg, 172.19 g·mol^{-1}, 7.75 µmol, 1.00 equiv), 400 µL CD_3CN, and **2-$^{15}N_2$** (1.04 mg, 134.14 g mol^{-1}, 7.75 µmol, 1.00 equiv), reaching a concentration of 19.4 mM. This sample was capped and used to tune, match, and shim the

magnet, after which the sample was ejected. To the same sample was added 100 µL 3.88 mM $Cu(OAc)_2{\cdot}H_2O$ (3.86 mg, 199.65 g·mol^{-1}, 19.3 µmol in 5.00 mL CD_3CN) to reach a final concentration of 15.5 mM and 5.00 mol% catalyst. The sample was shaken vigorously and immediately lowered into the magnet to start the measurement. ^{1}H and ^{1}H ^{15}N HMBC (Acquired on an AVII+ 600MHz spectrometer, TE = 300, NS = 4, D1 = 1.8, RG = 2050) spectra were acquired immediately using the *multi_zgvd2* command that included a *topshim_1dfast* shim after each acquisition. Experiments had a fixed delay of 120 seconds. To increase the clarity of 2D ^{1}H ^{15}N HMBC spectra, composite images were generated in which each compound of interest was assigned a colour. A representative spectrum containing more than one species was cloned and only the ^{1}H resonances (and thus the ^{15}N cross peaks) for the species of interest were kept and assigned a specific colour, while other resonances were obscured using signal suppression. This processed spectrum was then used as a composite layer. After all spectra were processed for each species, the final layers were overlapped to produce a composite image of the original spectra, in which all species coloured individually (Figure S63).

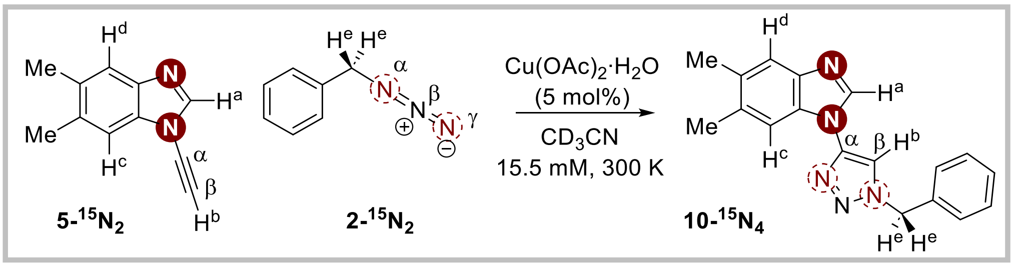

Scheme S22. Addition of 5.00 mol% $Cu(OAc)_2{\cdot}H_2O$ to 15.5 mM **5-^{15}N$_2$**, **2-^{15}N$_2$** (annotated for NMR) to study its effect on ^{1}H and ^{15}N resonances.

The reaction was monitored for 12 hours. The reaction is completed after 6.9 hours (Figure S60). Postulating from our observations, the triazole product **10-^{15}N$_4$** is (potentially) chelating with the remaining Cu(II) that is in solution, forming a triazole-$Cu(OAc)_2$-$Cu(OAc)_2$-triazole complex on the N_3 of **10-^{15}N$_4$**. It can clearly be seen that at the 8.07 ppm area **5-^{15}N$_4$** H^{a} starts broad and downfield as the ynamine initially chelates to Cu(II), the Cu(II) paddlewheel dissociates and is reduced to Cu(I) so the signals resolve, after which **5-^{15}N$_2$** is consumed as the reaction progresses. It was then observed how **10-^{15}N$_4$** H^{a} forms at 8.23 ppm, which starts to shift downfield after 6.9h, indicating that when the reaction is done, and suggesting that monomeric Cu(II) is not participating in the CuAAC anymore, and associates with the triazole product (Figure S62). A similar effect was observed for the **5-^{15}N$_2$-10-^{15}N$_4$** CH_3 moieties in closest vicinity of N^{3} at 2.4 ppm. Another interesting effect was observed when monitoring water at 2.15 ppm: its resonance starts sharp, but broadens over time, including a downfield

shift, suggesting an association with Cu(II). When the reaction is finished at 6.5h, water dissociates and becomes sharper again, presumably because the triazole product now chelates with the catalyst: water becomes available again (Figure S61). Additionally, a small resonance for acetate was observed downfield from CD_3CN at 1.96 ppm that is formed upon the addition of Cu(II). It displays a drastic downfield shift as the CuAAC progresses. It is postulated that acetate initially dissociates from Cu(II) and forms a strong resonance but is later reabsorbed by the Cu(II)/triazole complex as the resonance moved downfield and broadens.

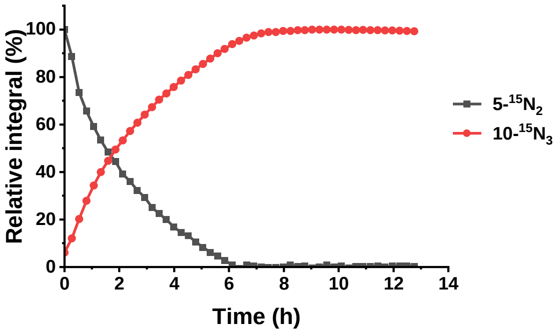

Figure S60. Reaction profile of the aromatic ynamine CuAAC reaction as monitored by ^{1}H NMR. 5-$^{15}N_2$-H^b and 10-$^{15}N_3$-H^e were monitored showing a plateau between 6-7 hours.

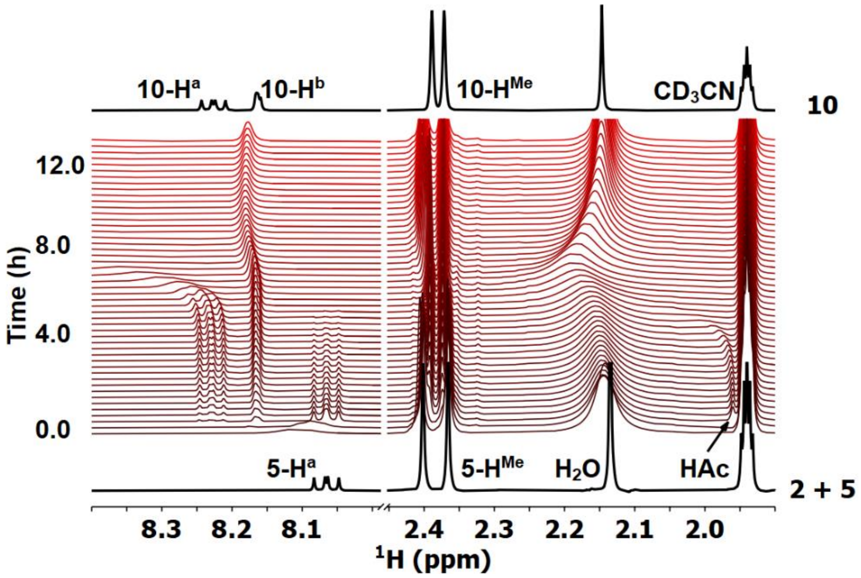

Figure S61. Stacked ^{1}H spectra of the ynamine CuAAC reaction as monitored over 12 hours. Top spectrum is isolated **10-$^{15}N_4$** in CD_3CN. Bottom spectrum is isolated **5-$^{15}N_2$** in CD_3CN. Acquired on an AVII+ 600MHz spectrometer, TE = 300, NS = 4, D1 = 1.8, RG = 2050. Referenced to CD_3CN, ^{1}H = 1.94 ppm.

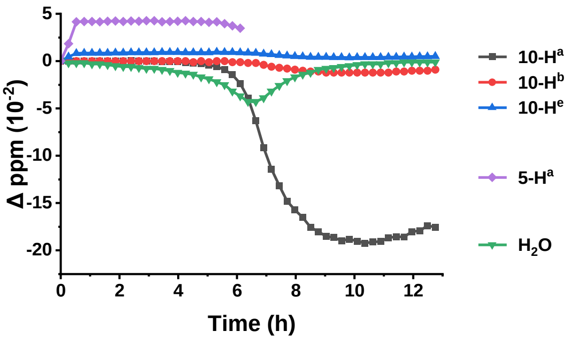

Figure S62. ^{1}H isotropic shift changes of **10-^{15}N$_4$** and **5-^{15}N$_2$** over time.

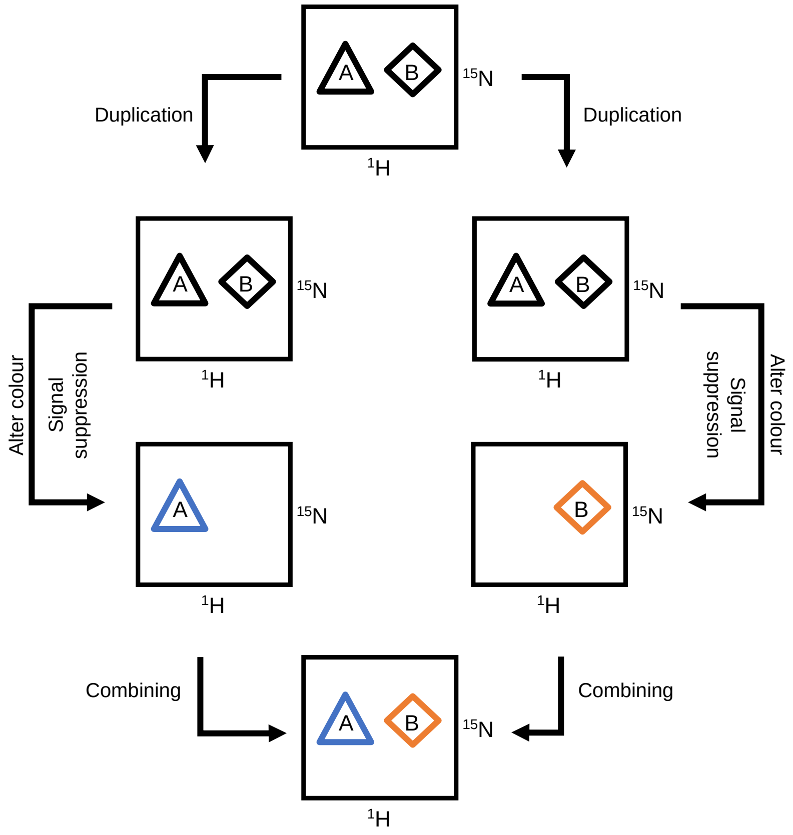

Figure S63. Flow chart used for the processing of ^{1}H ^{15}N HMBC when the production of composite images was required to give species a specific colour. The original ^{1}H ^{15}N HMBC spectrum was first duplicated, processed by signal suppression, and finally recomposited.

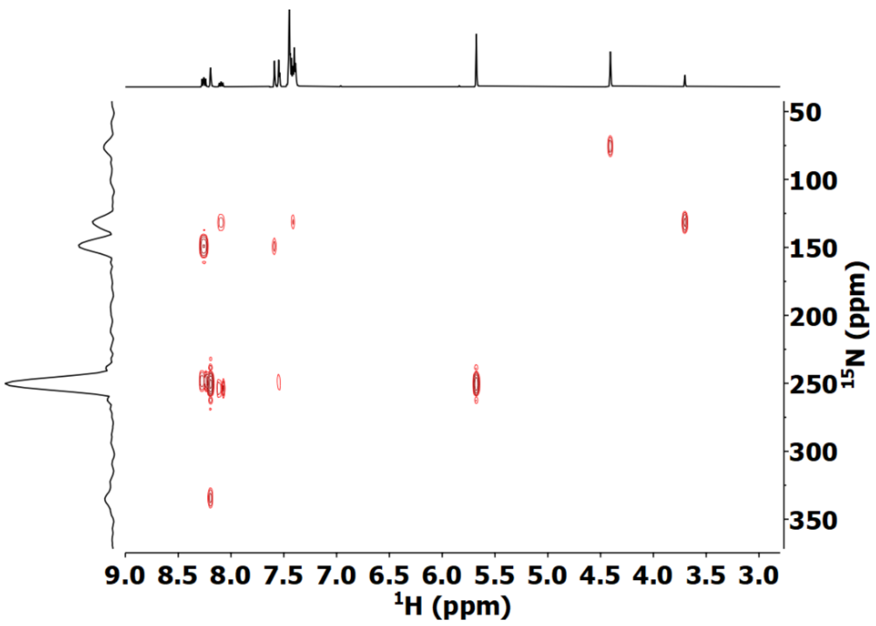

Figure S64. Original unprocessed ^{1}H ^{15}N HMBC of the reaction between **5-$^{15}N_2$** and **2-$^{15}N_2$** at 15.5 mM in CD_3CN, forming **10-$^{15}N_4$**, as acquired at t = 3 h. Referenced to CD_3CN, ^{1}H = 1.94 ppm, ^{15}N = 245 ppm. NS = 4, RG = 2050, D1 = 1.8.

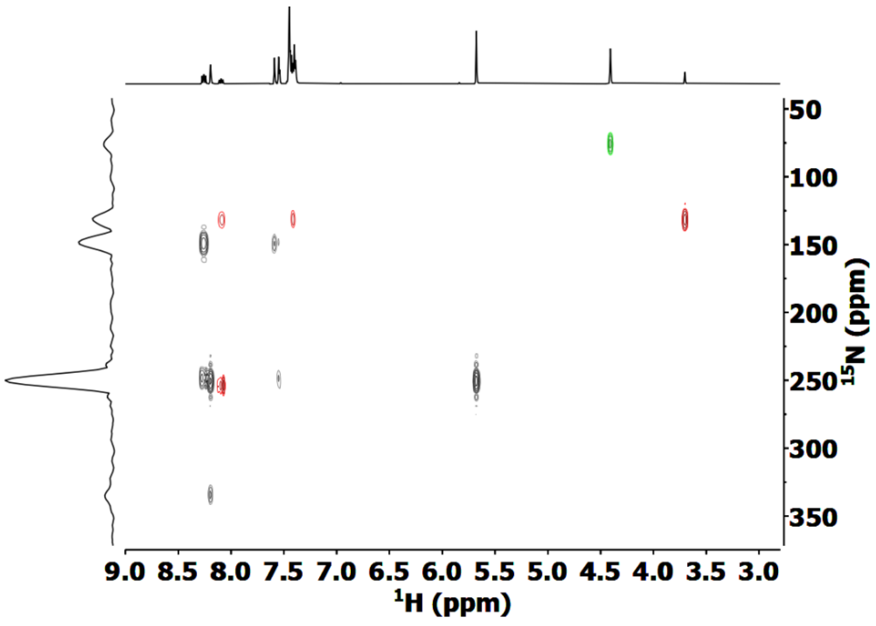

Figure S65. Composited ^{1}H ^{15}N HMBC of the reaction between **5-$^{15}N_2$** (red) and **2-$^{15}N_2$** (green), at 15.5 mM in CD_3CN, forming **10-$^{15}N_3$** (grey), as acquired at t = 3 h. Composite consists of 3 layers. Referenced to CD_3CN, ^{1}H = 1.94 ppm, ^{15}N = 245 ppm. NS = 4, RG = 2050, D1 = 1.8.

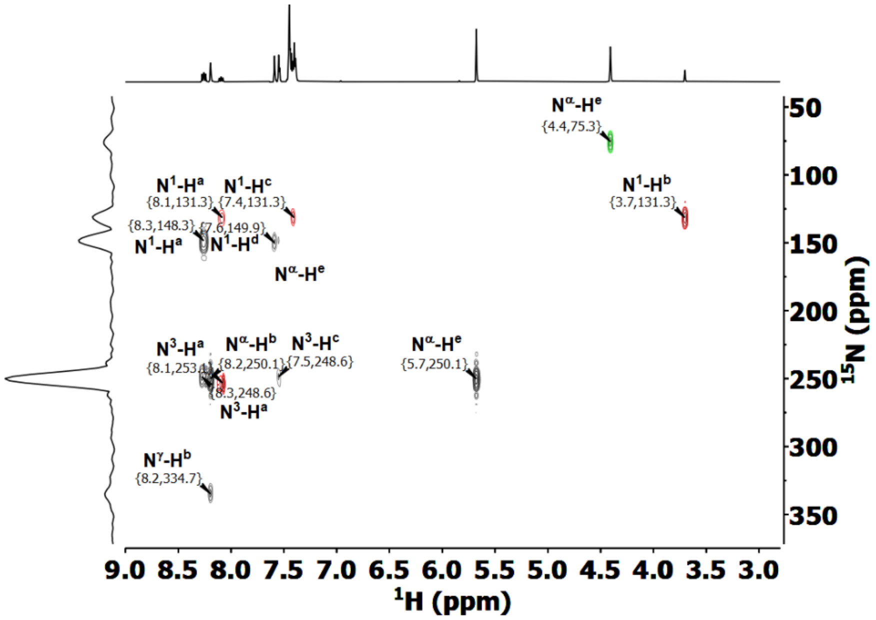

Figure S66. Composited ^{1}H ^{15}N HMBC of the reaction between **5-$^{15}N_2$** (red) and **2-$^{15}N_2$** (green), at 15.5 mM in CD_3CN, forming **10-$^{15}N_3$** (grey), as acquired at t = 3 h. Composite consists of 3 layers. Peaks picked and annotated. Referenced to CD_3CN, ^{1}H = 1.94 ppm, ^{15}N = 245 ppm. NS = 4, RG = 2050, D1 = 1.8.

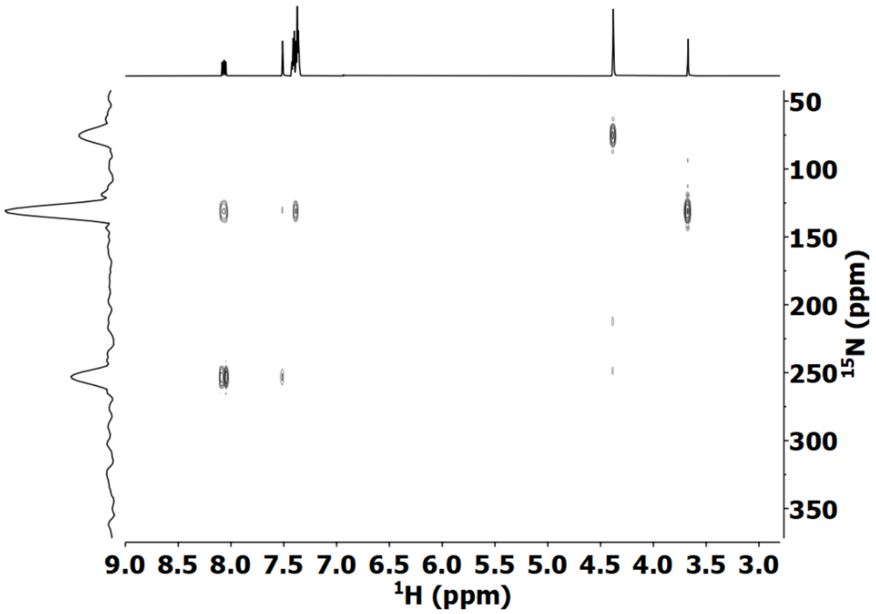

Figure S67. Original ^{1}H ^{15}N HMBC of a mixture of **5-$^{15}N_2$** and **2-$^{15}N_2$** at 15.5 mM in CD_3CN. Referenced to CD_3CN, ^{1}H = 1.94 ppm, ^{15}N = 245 ppm. NS = 4, RG = 2050, D1 = 1.8.

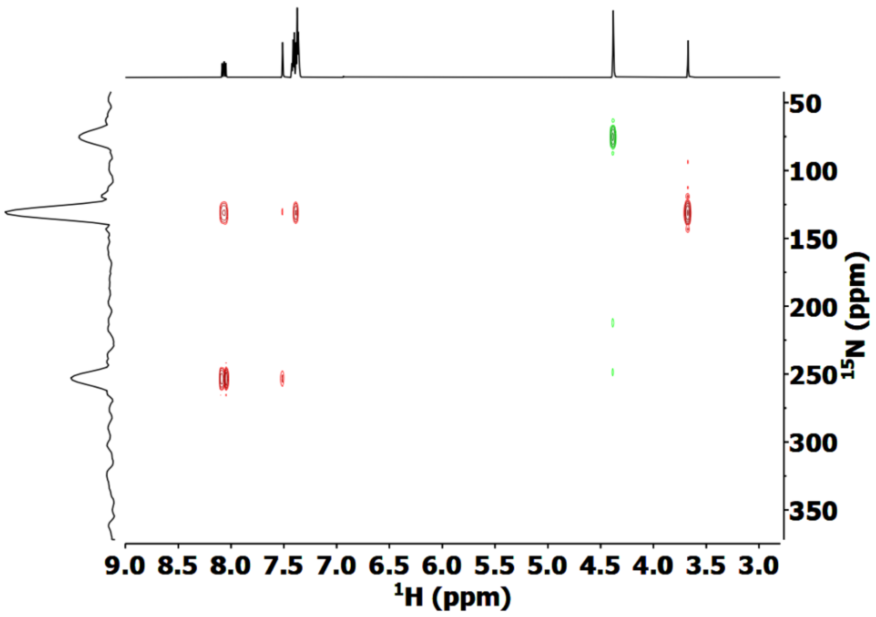

Figure S68. Composited ^{1}H ^{15}N HMBC of a mixture of **5-$^{15}N_2$** (red) and **2-$^{15}N_2$** (green) at 15.5 mM in CD_3CN. Composite consists of 2 layers. Referenced to CD_3CN, ^{1}H = 1.94 ppm, ^{15}N = 245 ppm. NS = 4, RG = 2050, D1 = 1.8.

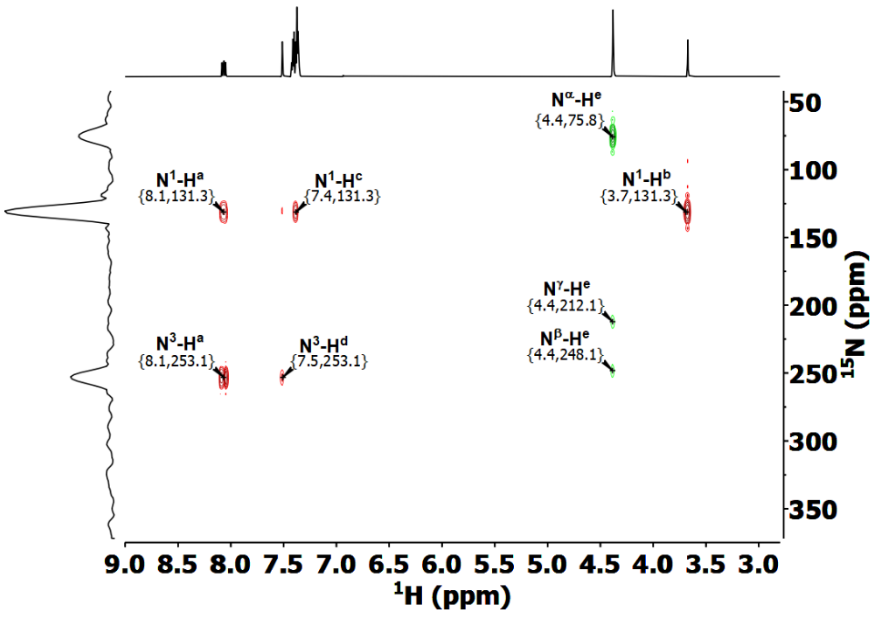

Figure S69. Composited ^{1}H ^{15}N HMBC of a mixture of **5-$^{15}N_2$** (red) and **2-$^{15}N_2$** (green) at 15.5 mM in CD_3CN. Composite consists of 2 layers. Peaks picked and annotated. Referenced to CD_3CN, ^{1}H = 1.94 ppm, ^{15}N = 245 ppm. NS = 4, RG = 2050, D1 = 1.8.

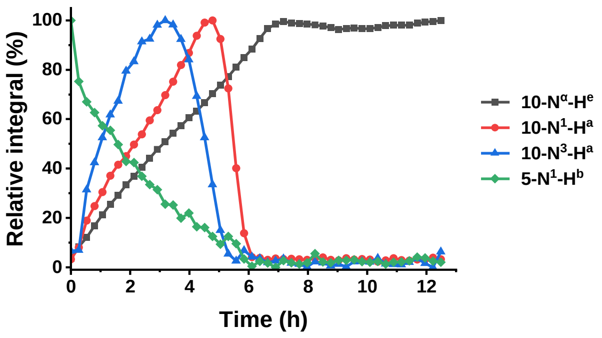

Figure S70. ^{1}H-^{15}N HMBC relative intensities of various nuclei in **5-$^{15}N_2$** and **10-$^{15}N_4$** as the CuAAC reaction progressed.

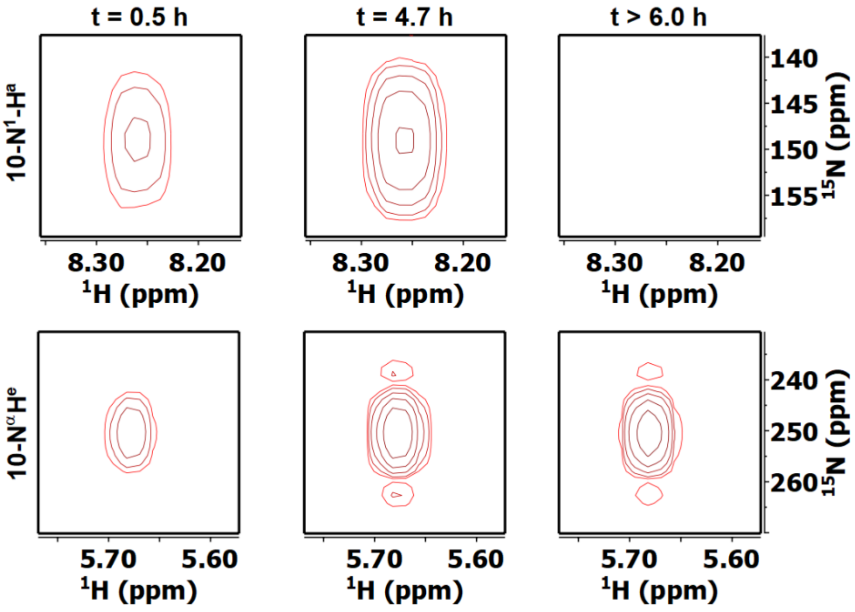

Figure S71. Progression of the CuAAC reaction as monitored by ^{1}H-^{15}N HMBC. Expansion plots of **10-$^{15}N_4$** N^1-H^a and **10-$^{15}N_4$** N^{α}-H^e at various crucial time-points. Intensities normalised.

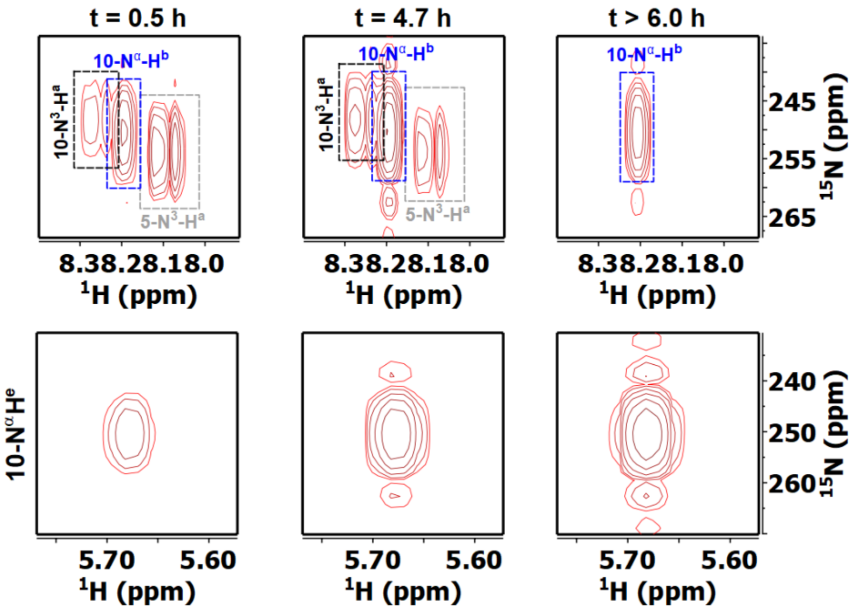

Figure S72. Progression of the CuAAC reaction as monitored by ^{1}H-^{15}N HMBC. Expansion plots of **10-^{15}N$_4$** N^3-H^a and **10-^{15}N$_4$** N$^\alpha$-H^e at various crucial time-points. Intensities normalised.

3.24 ^{13}C NMR monitoring of the ynamine-azide (3+2) cycloaddition reaction in CD_3CN

The (3+2) cycloaddition was monitored with **5-^{13}C$_2$-^{15}N$_2$**, **2-^{15}N$_2$** and 5.00 mol% $Cu(OAc)_2{\cdot}H_2O$ using ^{13}C NMR. To a glass vial was added **5-^{13}C$_2$-^{15}N$_2$** (3.24 mg, 174.18 g·mol^{-1}, 18.6 µmol, 1.00 equiv), **2-^{15}N$_2$** (2.50 mg, 134.14 g·mol^{-1}, 18.6 µmol, 1.00 equiv) and 300 µL CD_3CN, reaching a concentration of 62.0 mM. 125 µL of this mixture was transferred to an NMR tube and 375 µL CD_3CN was added, reaching a concentration of 15.5 mM. This reference sample was capped and lowered into the magnet. The reference sample was used to locked, tune, match, and shim the magnet in preparation of the reaction sample. ^{1}H and ^{13}C was acquired at 300 K to check the sample quality and the sample was ejected. A 15.5 mM $Cu(OAc)_2{\cdot}H_2O$ stock was prepared in CD_3CN (3.10 mg, 199.65 g·mol^{-1}, 15.5 µmol in 1.00 mL CD_3CN). 125 µL of the 62.0 mM **5-^{13}C$_2$-^{15}N$_2$** and **2-^{15}N$_2$** mixture, and 125 µL CD_3CN was added to an NMR tube, reaching a concentration of 31 mM. To this was added 250 µL 15.5 mM $Cu(OAc)_2{\cdot}H_2O$ to reach a final reagent concentration of 15.5 mM and 5.00 mol% catalyst. The sample was shaken vigorously and immediately lowered into the magnet to start the measurement. ^{13}C (Acquired on an AVII+ 600MHz spectrometer, TE = 300, NS = 64, D1 = 0.7, RG = 2050) spectra were acquired immediately using the *multi_zgvd2* command that included a *topshim_1dfast* shim after each acquisition. Experiments had a fixed delay of 60 seconds. The reaction was monitored over 4.4 hours, n = 125 spectra.

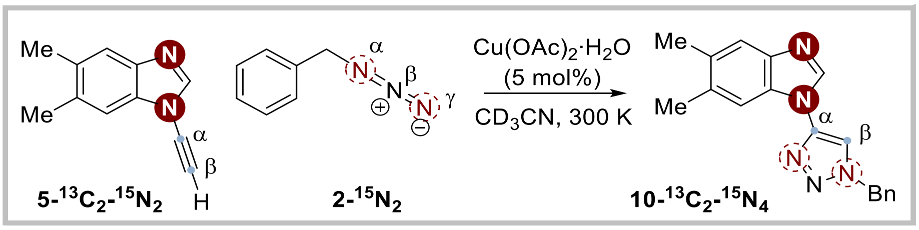

Scheme S23. Addition of 5.00 mol% $Cu(OAc)_2{\cdot}H_2O$ to 15.5 mM **5-$^{13}C_2$-$^{15}N_2$**, **2-$^{15}N_2$** (annotated for NMR) to study its effect on $^{13}C^{\alpha/\beta}$.

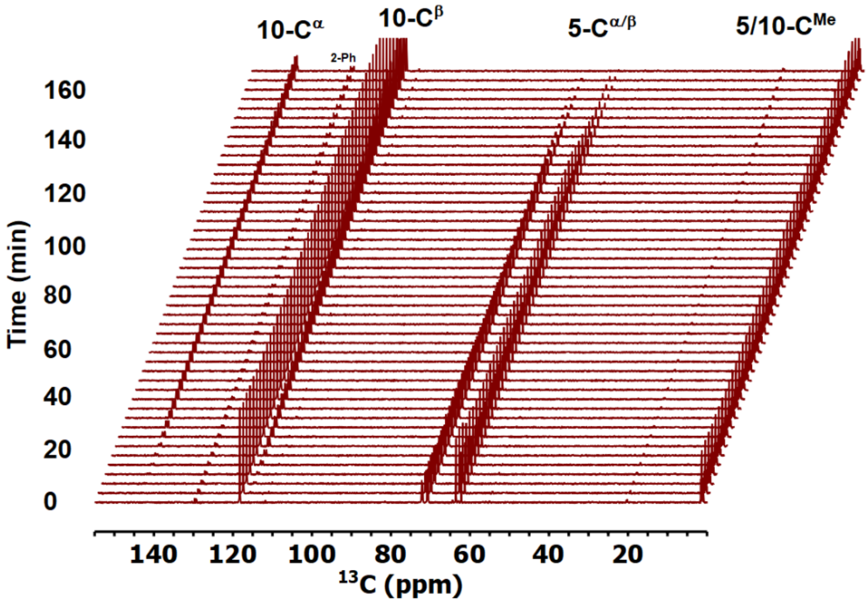

Figure S73. Stacked 1D ^{13}C spectra of 15.5 mM **5-$^{13}C_2$-$^{15}N_2$**, **2-$^{15}N_2$** and 5.00 mol% $Cu(OAc)_2{\cdot}H_2O$ in CD_3CN. Referenced to CD_3CN, ^{13}C = 118.26 ppm. NS = 64, RG = 2050, D1 = 0.7, TE = 300, with annotations.

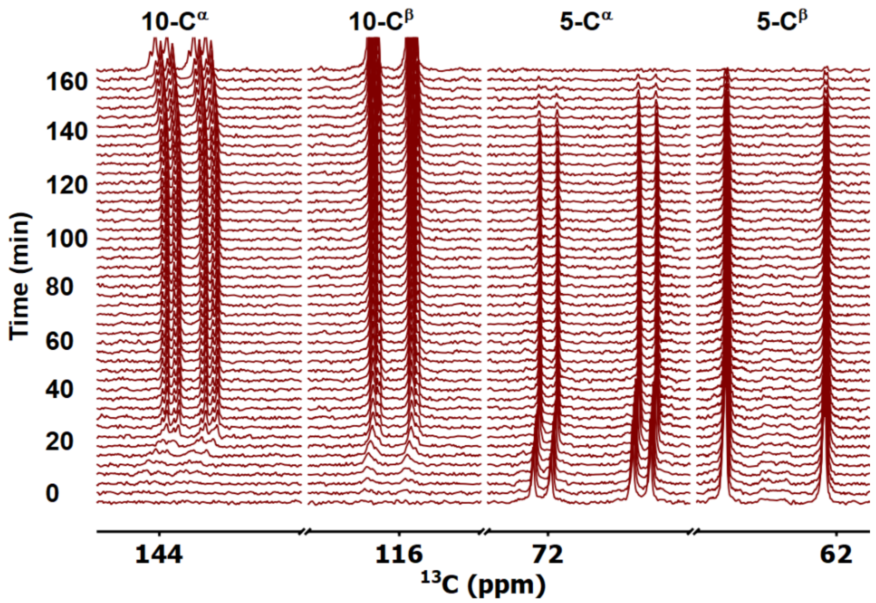

Figure S74. Expansion of stacked 1D ^{13}C spectra of 15.5 mM **5-$^{13}C_2$-$^{15}N_2$**, **2-$^{15}N_2$** and 5.00 mol% $Cu(OAc)_2{\cdot}H_2O$ in CD_3CN. Referenced to CD_3CN, ^{13}C = 118.26 ppm. NS = 64, RG = 2050, D1 = 0.7, TE = 300, with annotations.

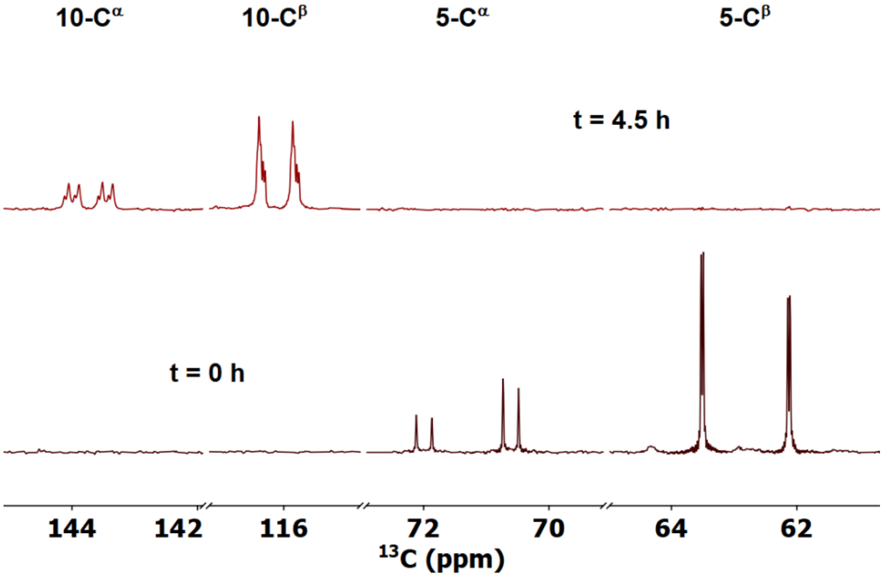

Figure S75. (bottom) 1D ^{13}C spectrum of 15.5 mM **5-$^{13}C_2$-$^{15}N_2$**, **2-$^{15}N_2$** prior to the addition of the catalyst, zoomed on alkyne and triazole resonance region. (top) Final acquired 1D ^{13}C spectrum of the reaction of 15.5 mM **5-$^{13}C_2$-$^{15}N_2$**, **2-$^{15}N_2$** and 5.00 mol% $Cu(OAc)_2{\cdot}H_2O$ in CD_3CN, showing the formation of (3+2) cycloaddition product **10-$^{13}C_2$-$^{15}N_4$**. Referenced to CD_3CN, ^{13}C = 118.26 ppm. NS = 64, RG = 2050, D1 = 0.7, TE = 300, with annotations.

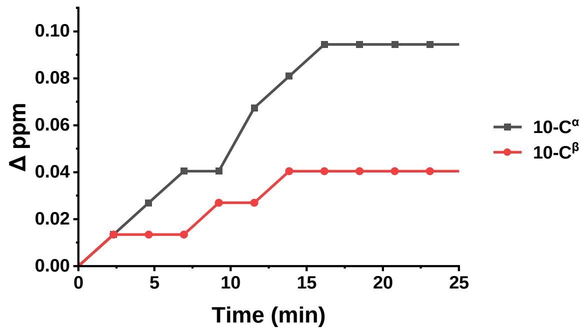

Figure S76. Isotropic shift change of **10-$^{13}C_2$-$^{15}N_4$**-C^{α} and **10-$^{13}C_2$-$^{15}N_4$**-C^{β} over time of 15.5 mM **5-$^{13}C_2$-$^{15}N_2$**, **2-$^{15}N_2$** and 5.00 mol% $Cu(OAc)_2{\cdot}H_2O$ in CD_3CN.

3.25 Monitoring the sequential addition of substrates post completed (3+2) cycloaddition reaction in CD_3CN

The unexpected observation that **10** formed a Cu complex by chelation at the apical site of $Cu(OAc)_2{\cdot}H_2O$ led us to explore the catalytic competency of this species after the (3+2) cycloaddition is complete. This was interrogated by first running the (3+2) cycloaddition with unlabelled substrates, followed by the addition of a second equivalent of isotopically labelled ynamine (**5-$^{13}C_2$-$^{15}N_2$**) and azide (**2-$^{15}N_2$**) was added to the reaction when the initial cycloaddition was complete.

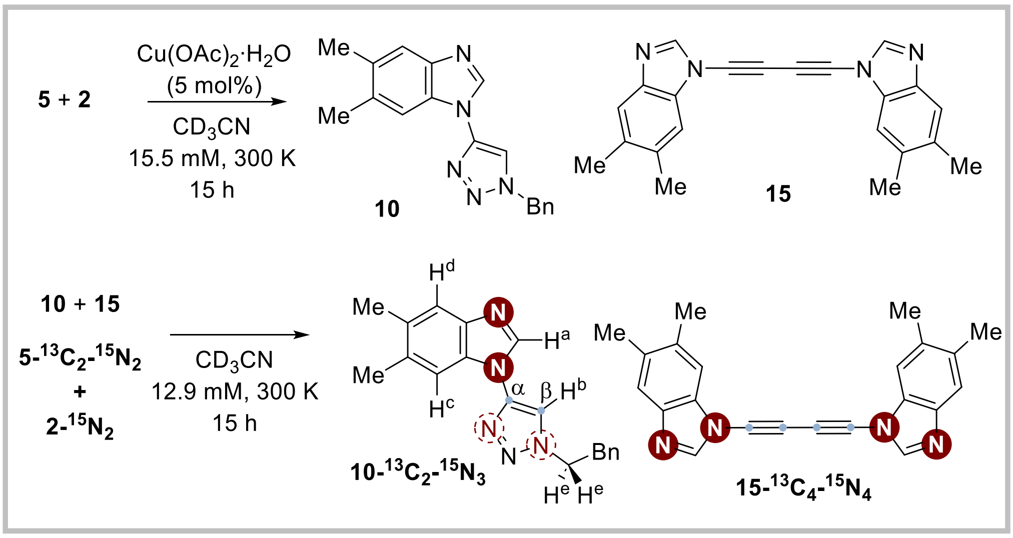

Scheme S24. Sequential addition of **5-$^{15}N_2$**, **2-$^{15}N_2$** (annotated for NMR) to an already finished (3+2) cycloaddition reaction to study its effect on ^{1}H and ^{15}N resonances.

To a glass vial was added **5** (1.32 mg, 170.21 g·mol^{-1}, 7.75 µmol, 1.00 equiv), 400 µL CD_3CN, and **2** (1.03 mg, 133.15 g·mol^{-1}, 7.75 µmol, 1.00 equiv), reaching a concentration of 19.4 mM.

This sample was capped and used to tune, match, and shim the magnet, after which the sample was ejected. To the same sample was added 100 µL 3.88 mM $Cu(OAc)_2 \cdot H_2O$ (3.86 mg, 199.65 g·mol^{-1}, 19.3 µmol in 5.00 mL CD_3CN) to reach a final concentration of 15.5 mM and 5.00 mol% catalyst. The sample was shaken vigorously and immediately lowered into the magnet to start the measurement. ^1H (Acquired on an AVII+ 600MHz spectrometer, TE = 300, NS = 4, D1 = 1.8, RG = 2050) spectra were acquired immediately using the *multi_zgvd2* command that included a *topshim* after each acquisition. Experiments had a fixed delay of 200 seconds. When the reaction was completed, a mixture of **5-$^{13}C_2$-$^{15}N_2$** (1.35 mg, 174.2 g·mol^{-1}, 7.75 µmol, 1.00 equiv) and **2-$^{15}N_2$** (1.04 mg, 1.065 g·mL^{-1}, 0.98 µL, 134.14 g·mol^{-1}, 7.75 µmol, 1.00 equiv) in 100 µL CD_3CN was added to reach a final concentration of 12.9 mM and 5.00 mol% catalyst. The sample was shaken vigorously and immediately lowered into the magnet to start the measurement. Using the Topspin job spooler, ^1H, ^{13}C and ^1H-^{15}N HMBC spectra were queued and acquired, allowing for tuning and matching, and shimming between experiments.

The time course of the reaction showed that the addition of **5-$^{13}C_2$-$^{15}N_2$** to the reaction mixture resulted in direct displacement of **10** as observed by ^1H-NMR (Figure 8b). Furthermore, there was a lack of N3 cross-peaks associated with **10-$^{13}C_2{}^{15}N_4$** suggestive that the N3 position is a more effective chelation site for the Cu catalyst than **10**. In addition, the second addition of isotopically labelled substrates **5-$^{13}C_2$-$^{15}N_2$** and **2-$^{15}N_2$** resulted in a faster rate of formation of **10-$^{13}C_2{}^{15}N_4$** relative the formation of **10** (Figure 8c). This is likely due to the Cu species (*e.g.,* **16**) is in a resting state with the apical face occupied by N3 chelation of **10**.

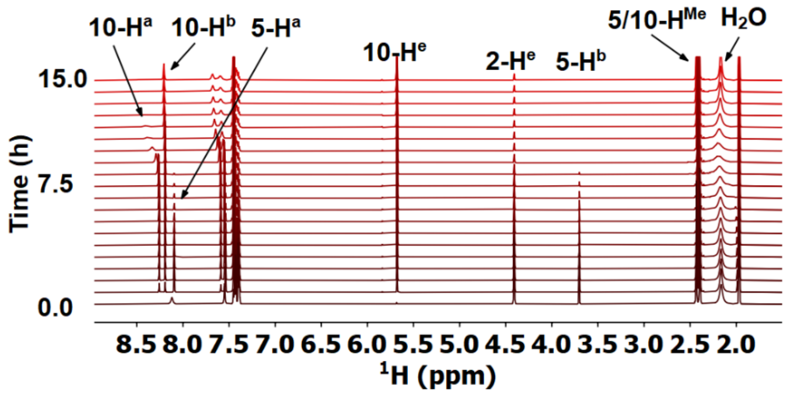

Figure S77. Full stacked ^1H spectra of the ynamine CuAAC reaction between **5** and **2** as monitored over 15 hours, before the addition of additional substrates. Acquired on an AVII+ 600MHz spectrometer, TE = 300, NS = 32, D1 = 2, RG = 256. Referenced to CD_3CN, ^1H = 1.94 ppm.

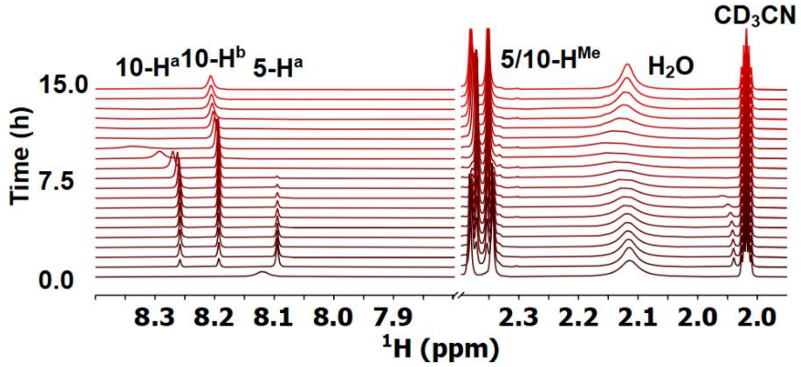

Figure S78. Zoomed stacked ^{1}H spectra of the ynamine CuAAC reaction **5** and **2** as monitored over 15 hours, before the addition of additional substrates. Acquired on an AVII+ 600MHz spectrometer, TE = 300, NS = 32, D1 = 2, RG = 256. Referenced to CD_3CN, ^{1}H = 1.94 ppm.

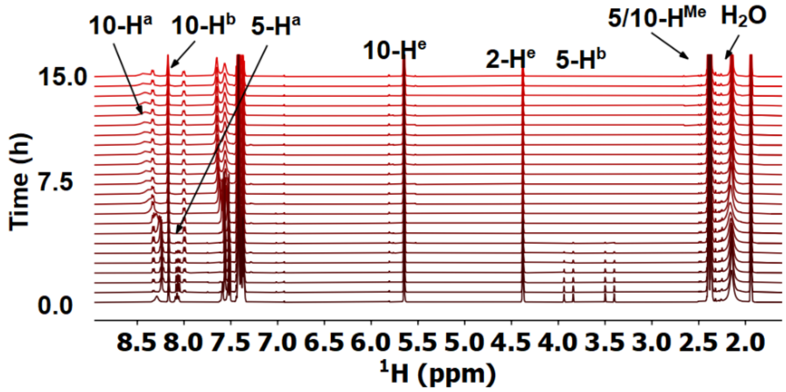

Figure S79. Full stacked ^{1}H spectra of the ynamine CuAAC reaction between **5-$^{13}C_2$-$^{15}N_2$** and **2-$^{15}N_2$** as monitored over 15 hours, after the initial reaction between **5** and **2**. Acquired on an AVII+ 600MHz spectrometer, TE = 300, NS = 32, D1 = 2, RG = 256. Referenced to CD_3CN, ^{1}H = 1.94 ppm.

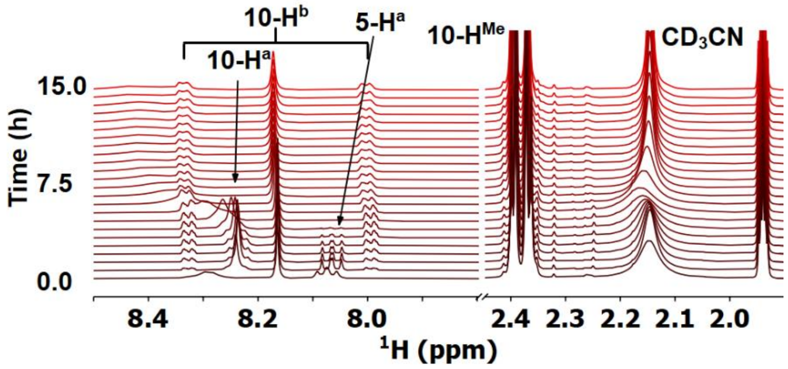

Figure S80. Zoomed stacked ^{1}H spectra of the ynamine CuAAC reaction between **5-$^{13}C_2$-$^{15}N_2$** and **2-$^{15}N_2$** as monitored over 15 hours, after the initial reaction between **5** and **2**. Acquired on

an AVII+ 600MHz spectrometer, TE = 300, NS = 32, D1 = 2, RG = 256. Referenced to CD_3CN, ^1H = 1.94 ppm.

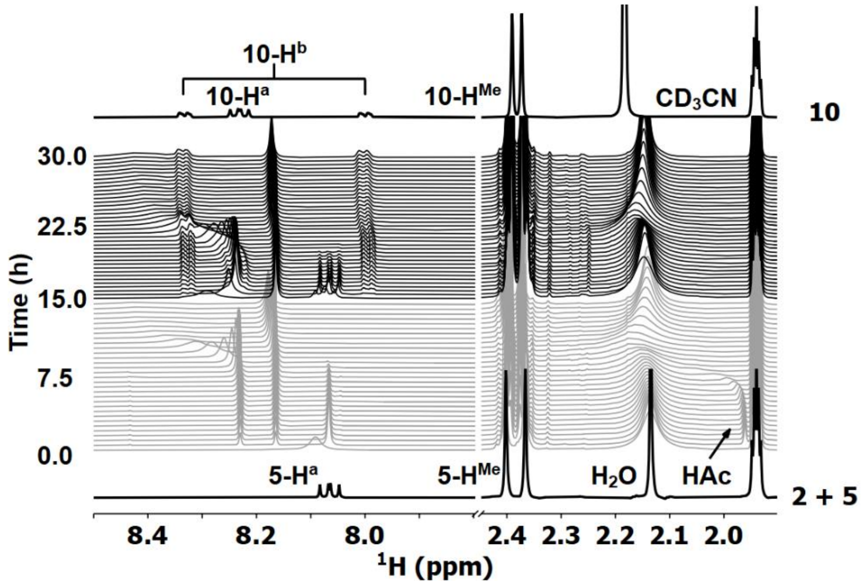

Figure S81. Combined stacked ^1H spectra of the ynamine CuAAC reaction between **5-$^{13}C_2$-$^{15}N_2$** and **2-$^{15}N_2$** as monitored over 15 hours, before (grey) and after (black) the initial reaction between **5** and **2**. Acquired on an AVII+ 600MHz spectrometer, TE = 300, NS = 32, D1 = 2, RG = 256. Referenced to CD_3CN, ^1H = 1.94 ppm.

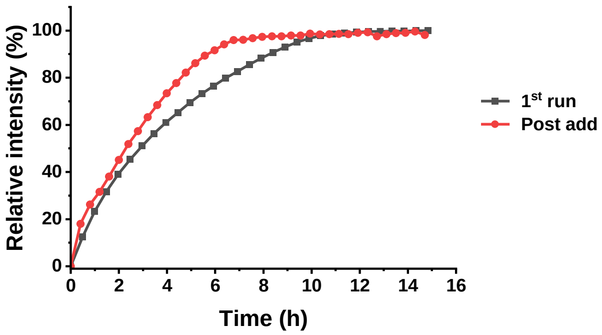

Figure S82. Reaction profile of the first and second aromatic ynamine CuAAC reaction as monitored by ^1H NMR, integrating **10-H^e**.

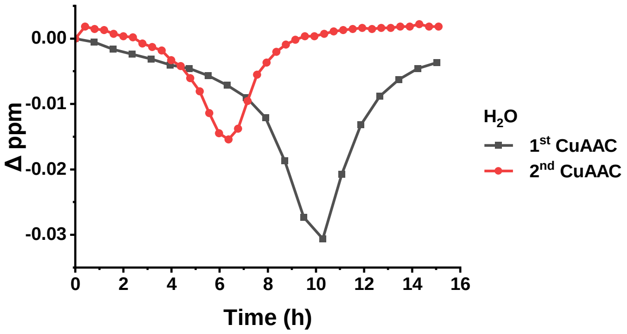

Figure S83. ^{1}H isotropic shift changes of water over time during the first and second (3+2) cycloaddition reaction.

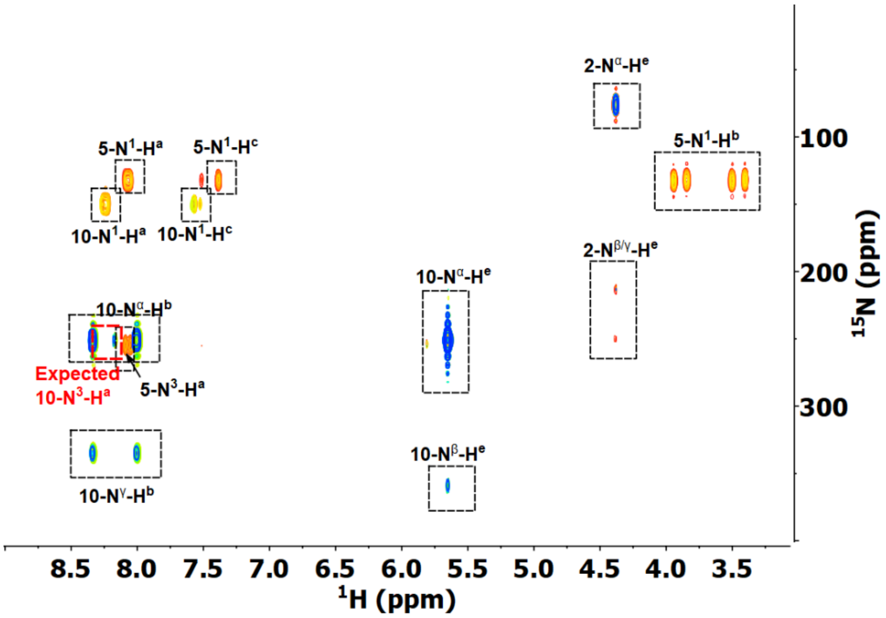

Figure S84. Stacked ^{1}H ^{15}N HMBC spectra of the ynamine CuAAC reaction between **5-$^{13}C_2$-$^{15}N_2$** and **2-$^{15}N_2$** as monitored over 15 hours, after the initial reaction between **5** and **2**. Correlations annotated, including expected position of **10-N^{3}-H^{a}**. Referenced to CD_3CN, ^{1}H = 1.94 ppm, ^{15}N = 245 ppm. NS = 4, RG = 2050, D1 = 1.8.

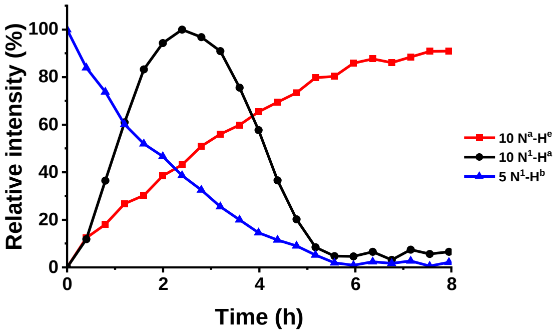

Figure S85. ^{1}H-^{15}N HMBC relative intensities of various nuclei in **5** and **10** as the CuAAC reaction progressed. Note how N^3 is not detected.

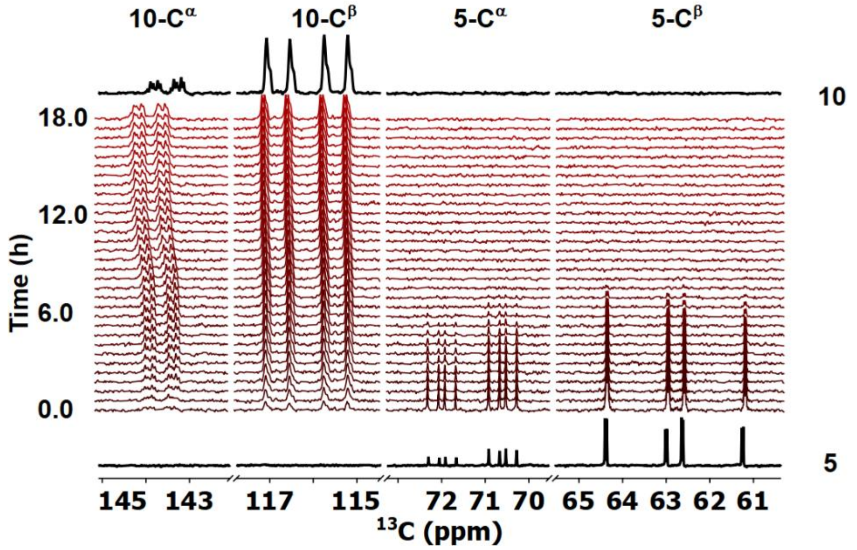

Figure S86. Expansion of stacked 1D ^{13}C spectra of the ynamine CuAAC reaction between **5-$^{13}C_2$-$^{15}N_2$** and **2-$^{15}N_2$** as monitored over 15 hours, after the initial reaction between **5** and **2**. Referenced to CD_3CN, ^{13}C = 118.26 ppm. NS = 64, RG = 2050, D1 = 0.7, TE = 300, with annotations. Top and bottom are isolated starting material and product.

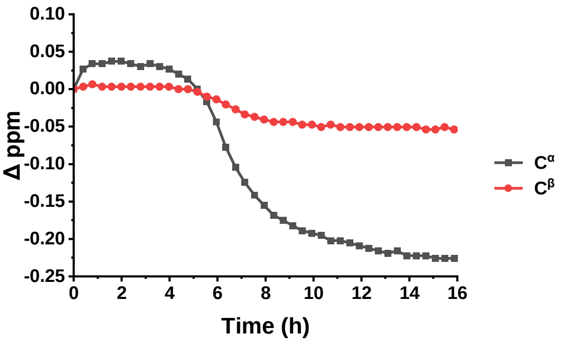

Figure S87. Isotropic shift change of **10-$^{13}C_2$-$^{15}N_4$**-C^{α} and **10-$^{13}C_2$-$^{15}N_4$**-C^{β} extracted from spectra of the ynamine CuAAC reaction between **5-$^{13}C_2$-$^{15}N_2$** and **2-$^{15}N_2$** as monitored over 15 hours, after the initial reaction between **5** and **2**.

3.26 Monitoring the sequential addition of compound 5-$^{13}C_2$-$^{15}N_2$ post completed (3+2) cycloaddition reaction in CD_3CN

We explored whether the amount of diyne formed (*i.e.*, **15-$^{13}C_4$$^{15}N_4$**) after completion of the first (3+2) cycloaddition was influenced by the presence of an azide in the reaction mixture. This was interrogated by just adding **5-$^{13}C_2$-$^{15}N_2$** after formation of **10**. Monitoring the reaction by ^{1}H NMR showed direct displacement of **10** from the Cu complex as observed previously.

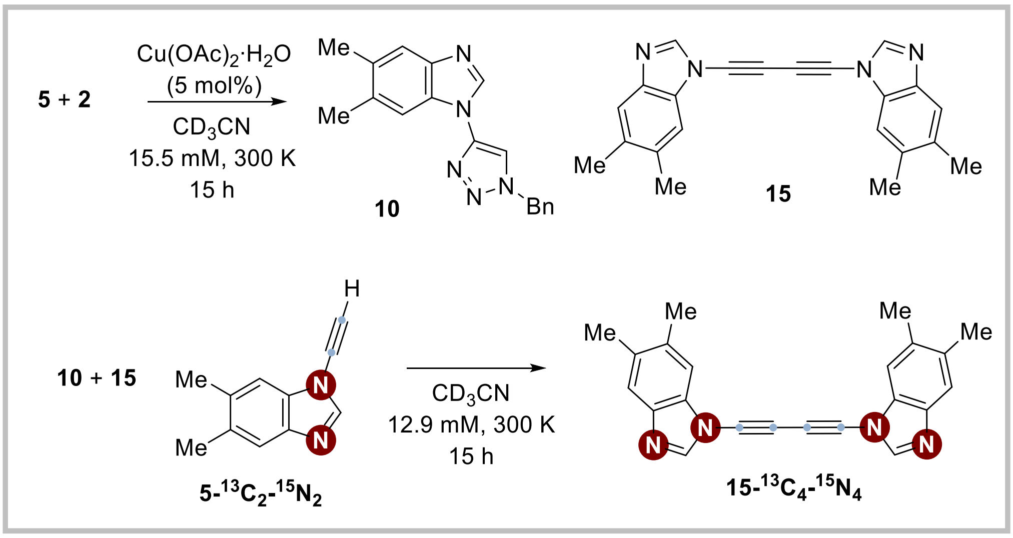

Scheme S25. Sequential addition of **5-^{15}N$_2$** (annotated for NMR) to an already finished (3+2) cycloaddition reaction to study its effect on ^{1}H and ^{15}N resonances.

To a glass vial was added **5** (1.32 mg, 170.21 g·mol^{-1}, 7.75 µmol, 1.00 equiv), 400 µL CD_3CN, and **2** (1.03 mg, 133.15 g·mol^{-1}, 7.75 µmol, 1.00 equiv), reaching a concentration of 19.4 mM. This sample was capped and used to tune, match, and shim the magnet, after which the sample was ejected. To the same sample was added 100 µL 3.88 mM $Cu(OAc)_2{\cdot}H_2O$ (3.86 mg, 199.65 g·mol^{-1}, 19.3 µmol in 5.00 mL CD_3CN) to reach a final concentration of 15.5 mM and 5.00 mol% catalyst. The sample was shaken vigorously and immediately lowered into the magnet to start the measurement. ^{1}H (Acquired on an AVII+ 600MHz spectrometer, TE = 300, NS = 4, D1 = 1.8, RG = 2050) spectra were acquired immediately using the *multi_zgvd2* command that included a *topshim* after each acquisition. Experiments had a fixed delay of 200 seconds. When the reaction was completed, a mixture of **5-^{13}C$_2$-^{15}N$_2$** (1.35 mg, 174.2 g·mol^{-1}, 7.75 µmol, 1.00 equiv) in 100 µL CD_3CN was added to reach a final concentration of 12.9 mM and 5.00 mol% catalyst. The sample was shaken vigorously and immediately lowered into the magnet to start the measurement. Using the Topspin job spooler, ^{1}H, ^{13}C and ^{1}H-^{15}N HMBC spectra were queued and acquired, allowing for tuning and matching, and shimming between experiments. Even though care has been taken to use exactly one equivalent of all substrates at all times, it proved to be challenging. Because of this, a small excess of **2** was left before the addition of **5-^{13}C$_2$-^{12}N$_2$**, and thus a minor amount of **10-^{13}C$_2$-^{15}N$_3$** formed, but still leaving a major excess of **5-^{13}C$_2$-^{12}N$_2$**.

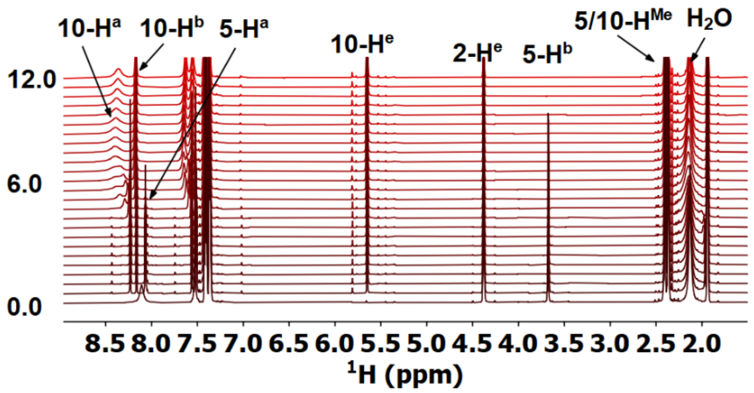

Figure S88. Full stacked ^{1}H spectra of the ynamine CuAAC reaction between **5** and **2** as monitored over 12 hours, before the addition of additional substrates. Acquired on an AVII+ 600MHz spectrometer, TE = 300, NS = 32, D1 = 2, RG = 256. Referenced to CD_3CN, ^{1}H = 1.94 ppm.

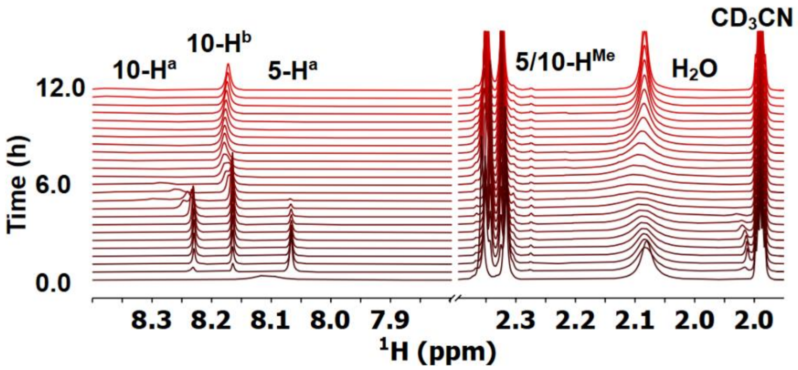

Figure S89. Zoomed stacked ^{1}H spectra of the ynamine CuAAC reaction **5** and **2** as monitored over 12 hours, before the addition of additional substrates. Acquired on an AVII+ 600MHz spectrometer, TE = 300, NS = 32, D1 = 2, RG = 256. Referenced to CD_3CN, ^{1}H = 1.94 ppm.

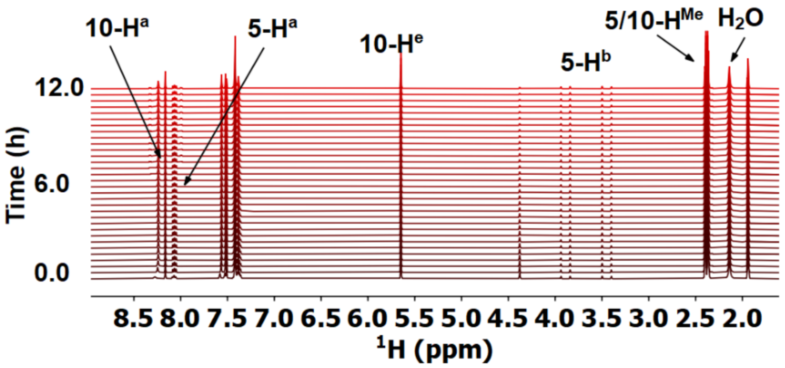

Figure S90. Full stacked ^{1}H spectra after the addition of **5-$^{13}C_2$-$^{15}N_2$** as monitored over 12 hours, after the initial reaction between **5** and **2**. Acquired on an AVII+ 600MHz spectrometer, TE = 300, NS = 32, D1 = 2, RG = 256. Referenced to CD_3CN, ^{1}H = 1.94 ppm.

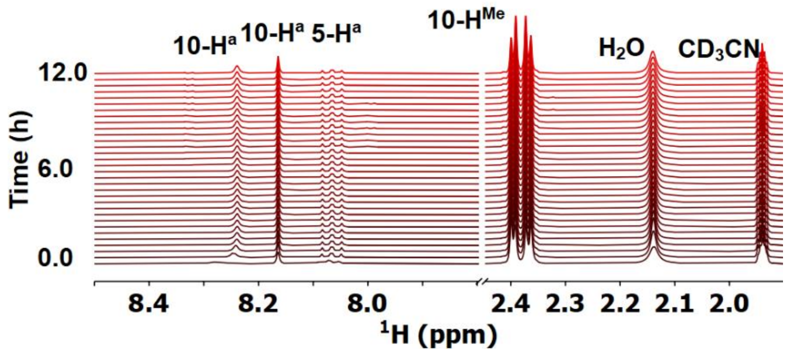

Figure S91. Zoomed stacked ^{1}H spectra after the addition of **5-^{13}C$_2$-^{15}N$_2$** as monitored over 12 hours, after the initial reaction between **5** and **2**. Acquired on an AVII+ 600MHz spectrometer, TE = 300, NS = 32, D1 = 2, RG = 256. Referenced to CD$_3$CN, ^{1}H = 1.94 ppm.

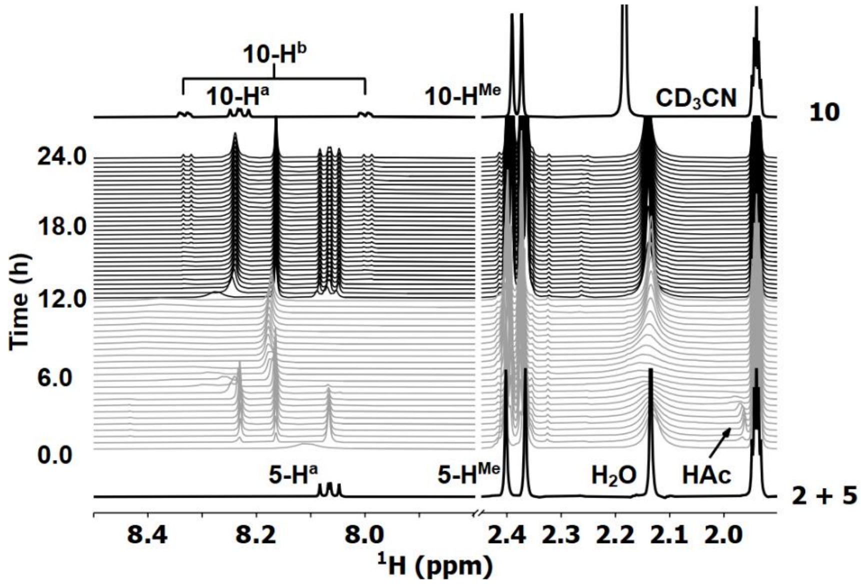

Figure S92. Combined stacked ^{1}H spectra after the addition of **5-^{13}C$_2$-^{15}N$_2$** as monitored over 12 hours, before (grey) and after (black) the initial reaction between **5** and **2**. Acquired on an AVII+ 600MHz spectrometer, TE = 300, NS = 32, D1 = 2, RG = 256. Referenced to CD$_3$CN, ^{1}H = 1.94 ppm.

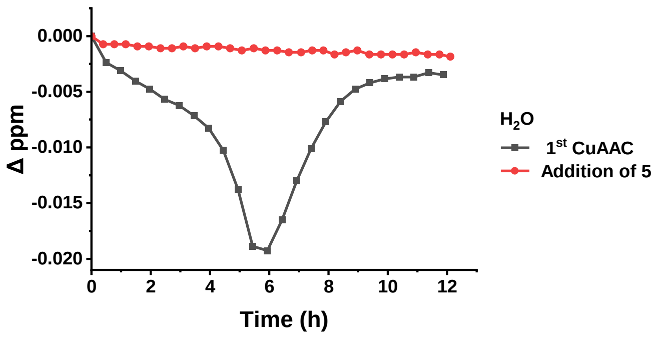

Figure S93. ^{1}H isotropic shift changes of water over time during the (3+2) cycloaddition reaction and after the addition of **5-$^{13}C_2$-$^{15}N_2$**.

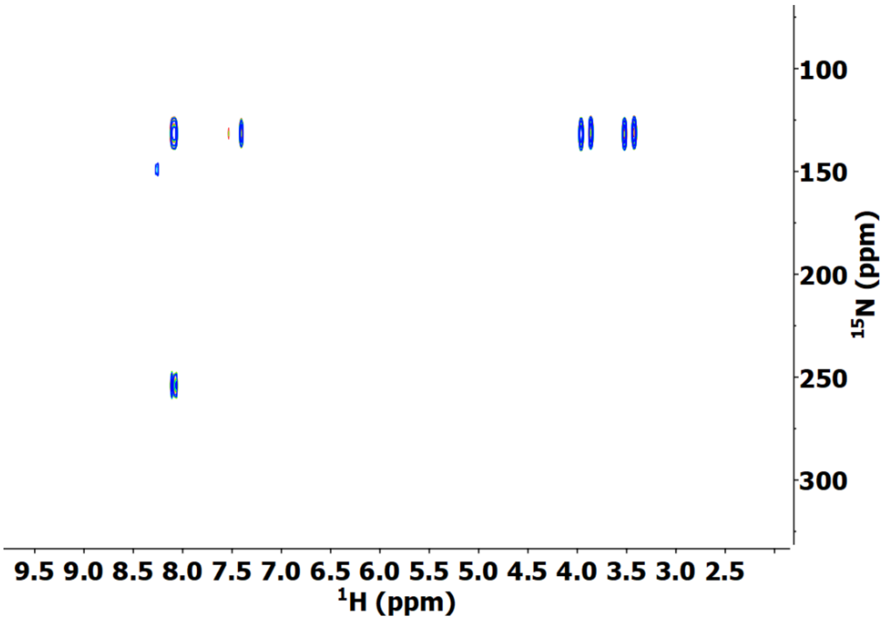

Figure S94. Stacked ^{1}H ^{15}N HMBC spectra of spectra after the addition of **5-$^{13}C_2$-$^{15}N_2$** as monitored over **12** hours, after the initial reaction between **5** and **2**. Referenced to CD_3CN, ^{1}H = 1.94 ppm, ^{15}N = 245 ppm. NS = 4, RG = 2050, D1 = 1.8.

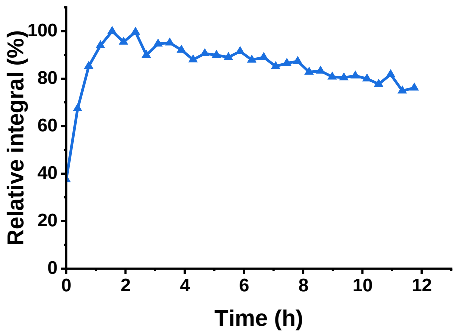

Figure S95. ^{1}H-^{15}N HMBC relative intensities of **5-$^{13}C_2$-$^{15}N_2$**.

3.27 Monitoring the sequential addition of compound 2 post completed (3+2) cycloaddition in CD_3CN

As a control, **2-$^{15}N_2$** was added to a finished (3+2) cycloaddition reaction between **5** and **2**.

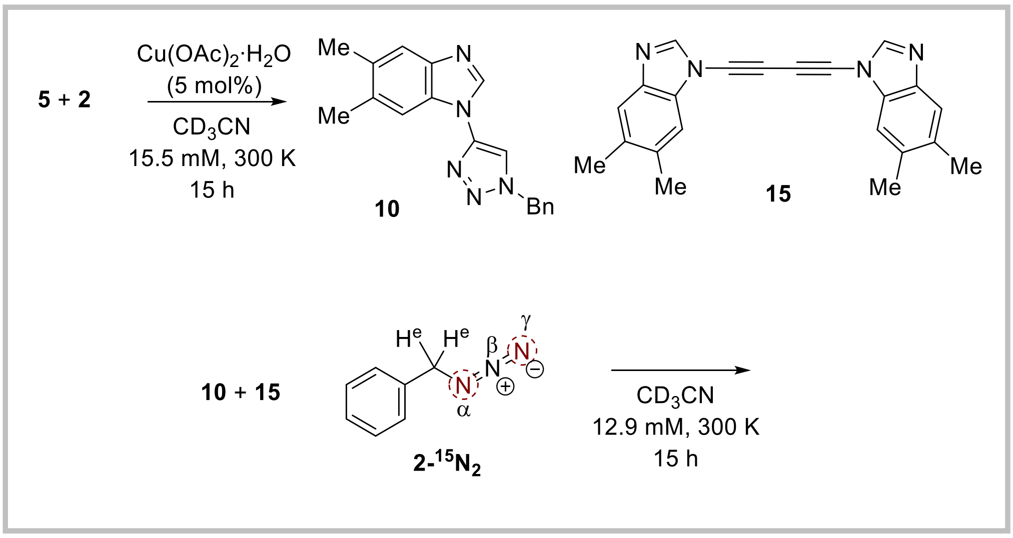

Scheme S26. Sequential addition of **5-$^{15}N_2$** (annotated for NMR) to an already finished (3+2) cycloaddition reaction to study its effect on ^{1}H and ^{15}N resonances.

To a glass vial was added **5** (1.32 mg, 170.21 g·mol^{-1}, 7.75 µmol, 1.00 equiv), 400 µL CD_3CN, and **2** (1.03 mg, 133.15 g·mol^{-1}, 7.75 µmol, 1.00 equiv), reaching a concentration of 19.4 mM. This sample was capped and used to tune, match, and shim the magnet, after which the sample was ejected. To the same sample was added 100 µL 3.88 mM $Cu(OAc)_2·H_2O$ (3.86 mg, 199.65 g·mol^{-1}, 19.3 µmol in 5.00 mL CD_3CN) to reach a final concentration of 15.5 mM and 5.00 mol% catalyst. The sample was shaken vigorously and immediately lowered into the magnet to start the measurement. ^{1}H (Acquired on an AVII+ 600MHz spectrometer, TE = 300, NS = 4, D1 = 1.8, RG = 2050) spectra were acquired immediately using the *multi_zgvd2* command that included a *topshim* after each acquisition. Experiments had a fixed delay of 200 seconds. When the reaction was completed, a mixture **2-$^{15}N_2$** (1.04 mg, 1.065 g·mL^{-1}, 0.98 µL, 134.14 g·mol^{-1}, 7.75 µmol, 1.00 equiv) in 100 µL CD_3CN was added to reach a final concentration of 12.9 mM and 5.00 mol% catalyst. The sample was shaken vigorously and immediately lowered into the magnet to start the measurement. Using the Topspin job spooler, ^{1}H, ^{13}C and ^{1}H-^{15}N HMBC spectra were queued and acquired, allowing for tuning and matching, and shimming between experiments.

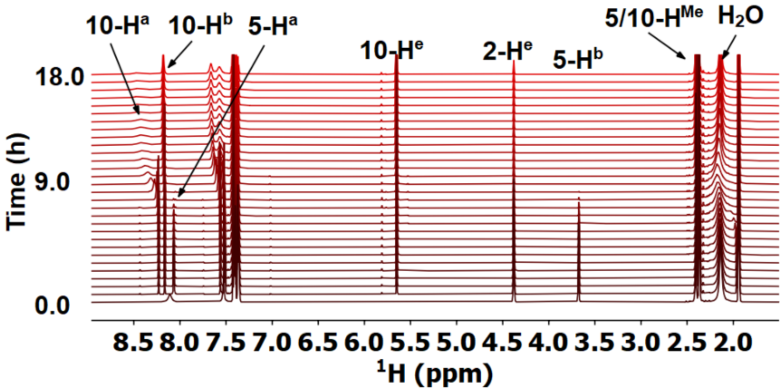

Figure S96. Full stacked ^{1}H spectra of the ynamine CuAAC reaction between **5** and **2** as monitored over 18 hours, before the addition of additional substrates. Acquired on an AVII+ 600MHz spectrometer, TE = 300, NS = 32, D1 = 2, RG = 256. Referenced to CD_3CN, ^{1}H = 1.94 ppm.

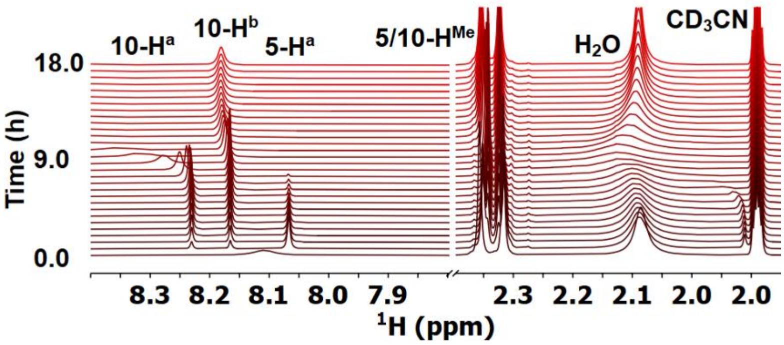

Figure S97. Zoomed stacked ^{1}H spectra of the ynamine CuAAC reaction **5** and **2** as monitored over 18 hours, before the addition of additional substrates. Acquired on an AVII+ 600MHz spectrometer, TE = 300, NS = 32, D1 = 2, RG = 256. Referenced to CD_3CN, ^{1}H = 1.94 ppm.

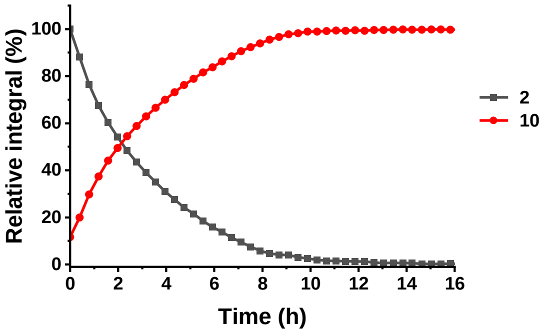

Figure S98. Reaction profile of the first (3+2) cycloaddition reaction between **5** and **2**, forming **10** as monitored by ^{1}H NMR, integrating **2-H^{e}** and **10-H^{e}**.

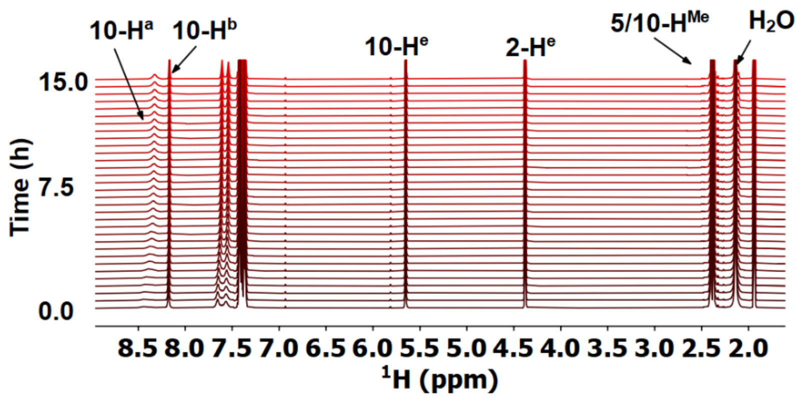

Figure S99. Full stacked ^{1}H spectra after the addition of **2-$^{15}N_2$** as monitored over 15 hours, after the initial reaction between **5** and **2**. Acquired on an AVII+ 600MHz spectrometer, TE = 300, NS = 32, D1 = 2, RG = 256. Referenced to CD_3CN, ^{1}H = 1.94 ppm.

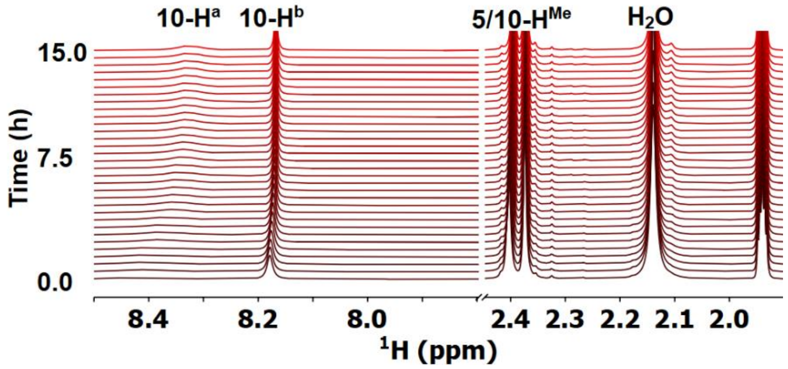

Figure S100. Zoomed stacked ^{1}H spectra after the addition of **2-$^{15}N_2$** as monitored over 15 hours, after the initial reaction between **5** and **2**. Acquired on an AVII+ 600MHz spectrometer, TE = 300, NS = 32, D1 = 2, RG = 256. Referenced to CD_3CN, ^{1}H = 1.94 ppm.

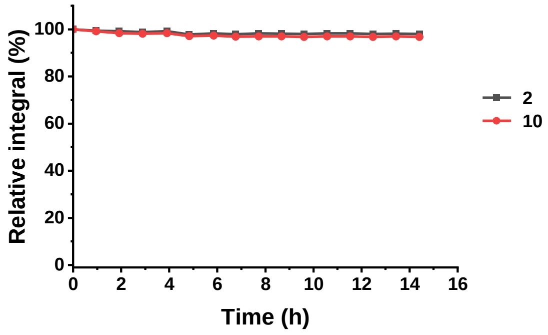

Figure S101. Monitoring the integrals **2-H^{e}** and **10-H^{e}** after the addition of **2-$^{15}N_2$** to the initial reaction between **5** and **2** forming **10**.

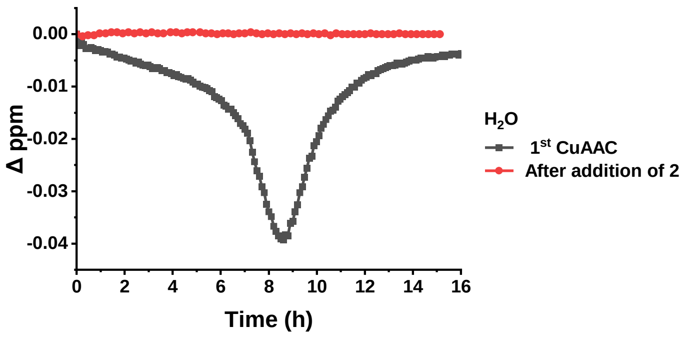

Figure S102. ^{1}H isotropic shift changes of water over time during the (3+2) cycloaddition reaction and after the addition of **2-^{15}N$_2$**.

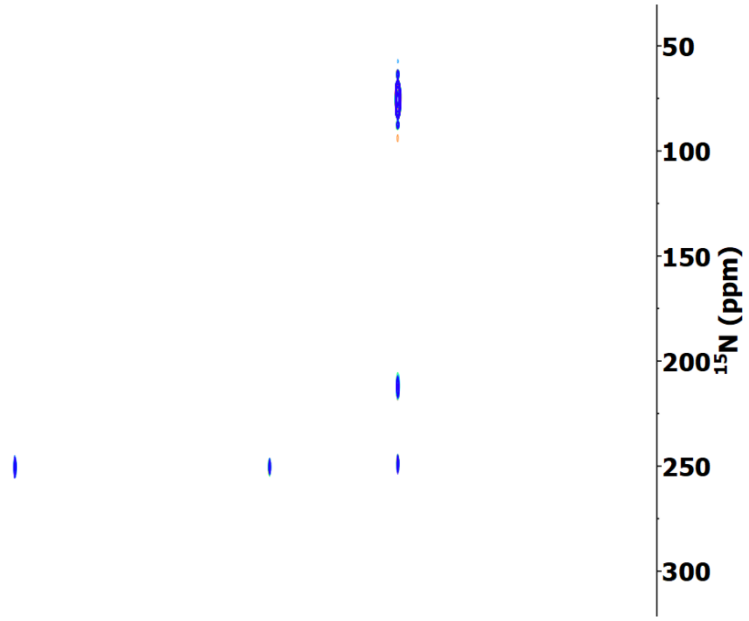

Figure S103. Stacked ^{1}H ^{15}N HMBC spectra of spectra after the addition of **2-^{15}N$_2$** as monitored over **12** hours, after the initial reaction between **5** and **2**. Referenced to CD_3CN, ^{1}H = 1.94 ppm, ^{15}N = 245 ppm. NS = 4, RG = 2050, D1 = 1.8. Note that there are cross-peaks for the correlations of **10** N$^{\alpha}$-H^{b} and **10** N$^{\alpha}$-H^{e} which are there due to the sensitivity of these resonances for natural abundant ^{15}N in **10**.

4 EPR

EPR experiments conducted on a Bruker EMX plus spectrometer controlled by a Bruker ER 083 CS microwave bridge operating at X-Band, microwave frequency of ≈9.9 GHz at microwave power of 20.70 mW, modulation amplitude of 10.0 G and a time constant of 40.96 s. The sweep centre was at 3425 G and a sweep width of 2000 G with 2000 points on X-axis. All spectra were corrected against a solvent baseline.

4.1 EPR spectrum of compound 14 in presence of $Cu(OAc)_2 \cdot H_2O$

TIPS-protected ynamine **14** (16.3 mg, 50 µmol, 10 equiv, 50 mM) and $Cu(OAc)_2 \cdot H_2O$ (0.9 mg, 5 µmol, 0.10 equiv, 5 mM) were dissolved in CH_3CN (1 mL) and mixed before a 20 µL aliquot was taken and EPR spectrum was acquired.

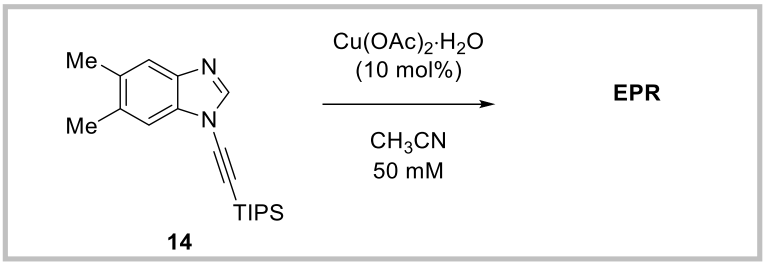

Scheme S27. Addition of $Cu(OAc)_2 \cdot H_2O$ to ynamine **14**.

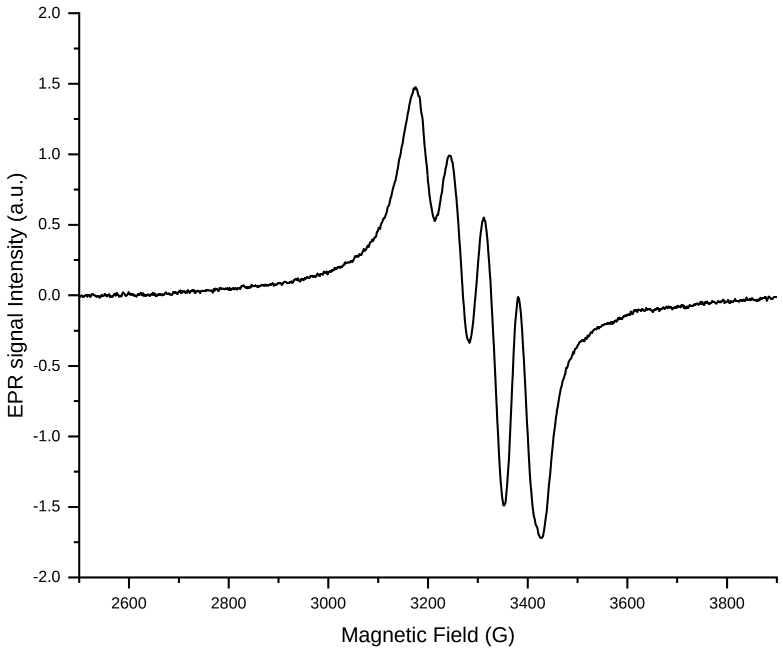

Figure S104. EPR spectrum of TIPS-protected ynamine **14** in presence of $Cu(OAc)_2 \cdot H_2O$.

4.2 EPR monitoring compound 5 in presence of $Cu(OAc)_2{\cdot}H_2O$

Ynamine **5** (8.5 mg, 50 µmol, 1 equiv, 50 mM) and $Cu(OAc)_2{\cdot}H_2O$ (0.9 mg, 5 µmol, 0.1 equiv, 5 mM) were dissolved in CH_3CN (1 mL) in a 2 mL glass vial and mixed. 20 µL aliquots were taken over the course of the reaction and EPR spectra were acquired.

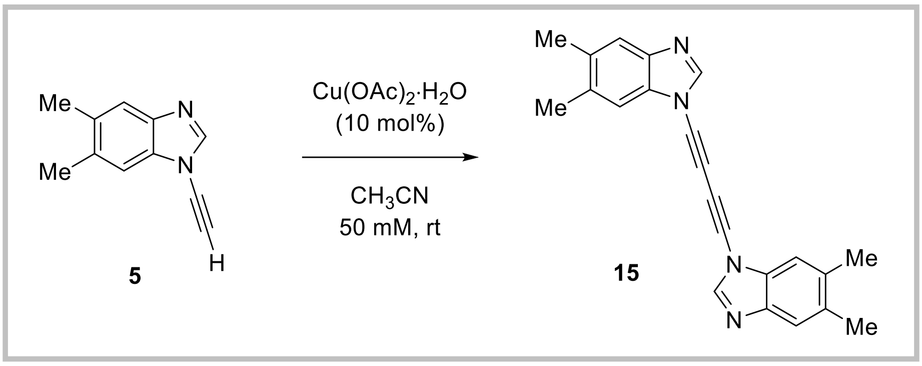

Scheme S28. Addition of $Cu(OAc)_2{\cdot}H_2O$ to Ynamine **5**.

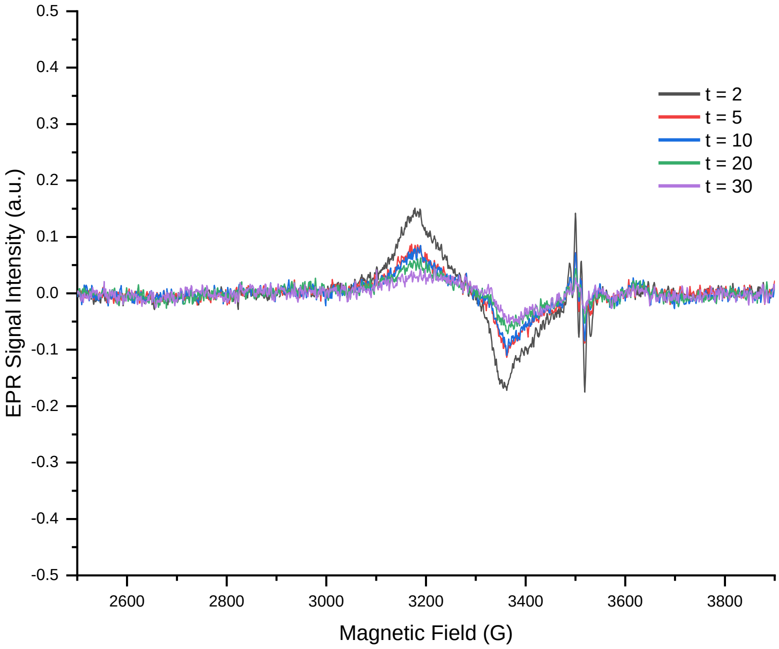

Figure S105. Stacked EPR spectra of Ynamine **5** in presence of $Cu(OAc)_2{\cdot}H_2O$ at different time points over the course of 30 min (2 min (black line), 5 min (red line), 10 min (blue line), 20 min (green line) and 30 min (purple line)).

4.3 EPR monitoring Ynamine-Azide (3+2) Cycloaddition in CH_3CN

Ynamine **5** (17.0 mg, 0.1 mmol, 1 equiv, 100 mM), azide **2** (13.3 mg, 0.1 mmol, 1 equiv, 100 mM) and $Cu(OAc)_2{\cdot}H_2O$ (0.9 mg, 5 µmol, 0.05 equiv, 5 mM) were dissolved in CH_3CN (1 mL) in a 2 mL glass vial and mixed. 20 µL aliquots were taken over the course of the reaction and EPR spectra were acquired.

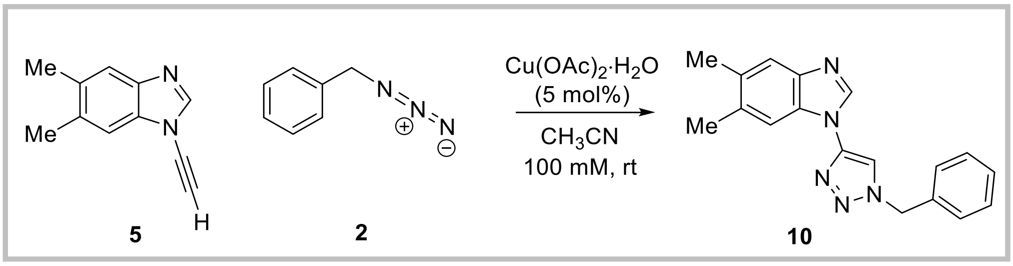

Scheme S29. Ynamine-Azide (3+2) Cycloaddition of ynamine **5** with azide **2**.

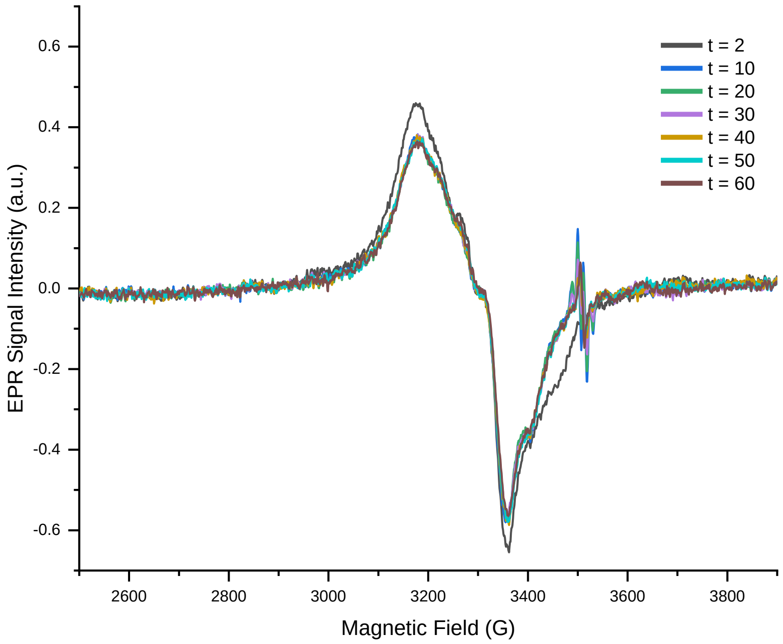

Figure S106. Stacked EPR spectra of Ynamine-Azide (3+2) Cycloaddition of ynamine **5** with azide **2** in presence of 5 mol % $Cu(OAc)_2{\cdot}H_2O$ at different time points over the course of 1 h (2 min (black line), 10 min (blue line), 20 min (green line), 30 min (purple line), 40 min (yellow line), 50 min (light blue), 60 min (brown)).

4.4 EPR monitoring Ynamine-Azide (3+2) Cycloaddition in CH_3CN in the same conditions as the NMR experiments

An EPR experiment was carried out under the exact same conditions as the NMR monitoring experiments (15 mM Ynamine solution and 5 mol % $Cu(OAc)_2{\cdot}H_2O$. Ynamine **5** (1.4 mg, 7.75 µmol, 1 equiv, 15.5 mM) and azide **2** (1.0 mg, 7.75 µmol, 1 equiv, 15.5 mM) were dissolved in CH_3CN (0.4 mL). Next, 0.1 mL of a $Cu(OAc)_2{\cdot}H_2O$ stock solution in CH_3CN was added (3.8 mg, 20 µmol, 5 mL CH_3CN) to the reaction mixture in a 2 mL glass vial. 20 µL aliquots were taken over the course of the reaction and EPR spectra were acquired. The reaction was monitored over 28 h.

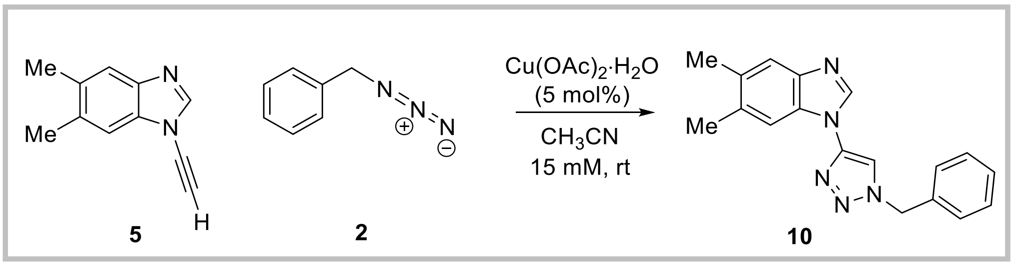

Scheme S30. Ynamine-Azide (3+2) Cycloaddition of ynamine **5** with azide **2** using NMR experiment conditions.

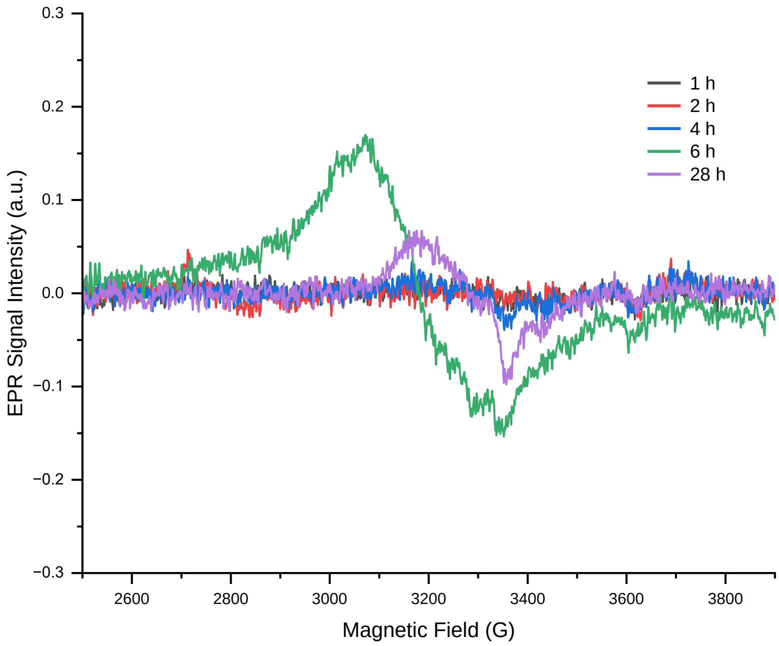

Figure S107. Stacked EPR spectra of Ynamine-Azide (3+2) Cycloaddition of ynamine **5** with azide **2** in presence of 5 mol % $Cu(OAc)_2{\cdot}H_2O$ at different time points over the course of 28 h (1 h (black line), 2 h (red line), 4 h (blue line), 6 h (green line), 28 h (purple line)).

4.5 EPR control experiments

4.5.1 EPR spectra of reagents

Baseline monitoring of $Cu(OAc)_2{\cdot}H_2O$ (0.9 mg, 5 µmol, 5 mM) dissolved in CH_3CN and starting materials (Ynamine **5** (17.0 mg, 0.1 mmol, 1 equiv, 100 mM) and compound **2** (13.3 mg, 0.1 mmol, 1 equiv, 100 mM) dissolved in CH_3CN (1 mL). $Cu(OAc)_2{\cdot}H_2O$ denucleation was not observed and both starting material are EPR silent.

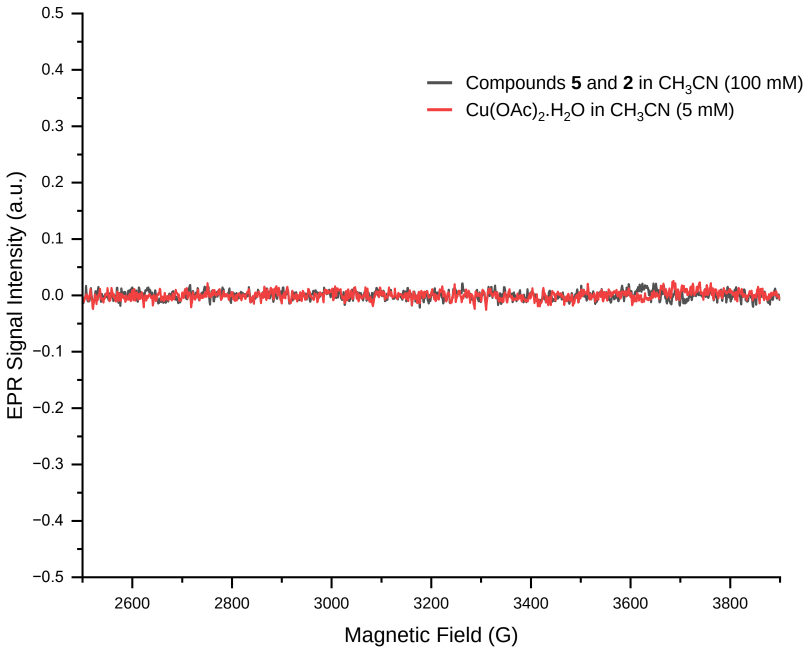

Figure S108. EPR spectra of Ynamine **5** and azide **2** 100 mM in CH_3CN (grey line) and $Cu(OAc)_2 \cdot H_2O$ 5 mM in CH_3CN.

4.5.2 EPR experiment monitoring the effect of triazole 10 and Cu(I) in the EPR signal during the time course experiment over 6 h

To study the cause of the increase in signal to noise ratio in Figure S107, Cu(OAc) (0.2 mg, 1.5 µmol, 0.05 equiv, 0.8 mM) was dissolved in CH_3CN (2 mL) and compound **10** (9.0 mg, 30 µmol, 1 equiv, 15 mM) was added. 20 µL aliquots were taken over the course of the reaction and EPR spectra were acquired. The mixture was monitored over 6 h, however no signal was observed.

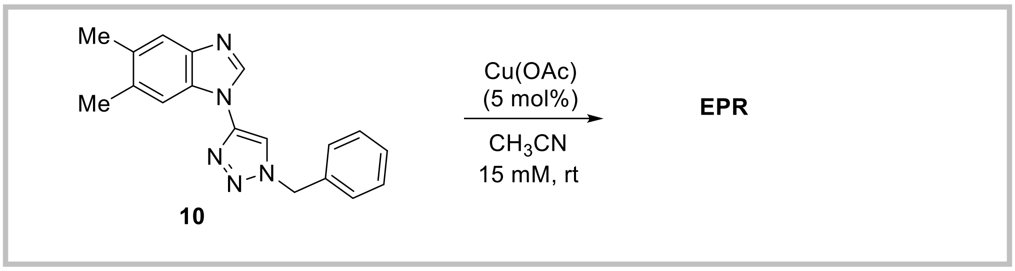

Scheme S31. Addition of Cu(OAc) to triazole **10**.

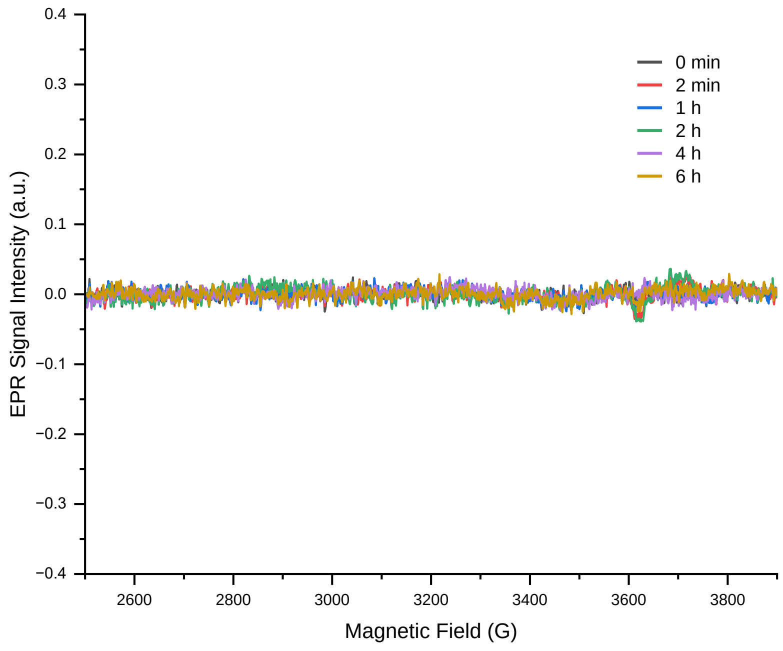

Figure S109. Stacked EPR spectra of a mixture of triazole **10** (15 mM) and Cu(OAc) in CH_3CN over the course of 6 h (time 0 (black line), 2 min (red line), 1 h (blue line), 2 h (green line), 4 h (purple line), 6 h (yellow line)).

4.5.3 EPR experiment monitoring the effect of the triazole 10 and Cu(II) in the S/N during the time course experiment over 6 h

A solution of $Cu(OAc)_2{\cdot}H_2O$ (0.3 mg, 1.5 µmol, 0.05 equiv, 1.5 mM) was prepared in 1 mL MeCN in a 2 mL sample vial. Triazole **10** (9.1 mg, 30 µmol, 1 equiv, 30 mM) was then added, the solution mixed, and further 20 µL aliquots were taken over a period of 6 h. A signal appeared over time corresponding with desymmetrization of the paddlewheel.

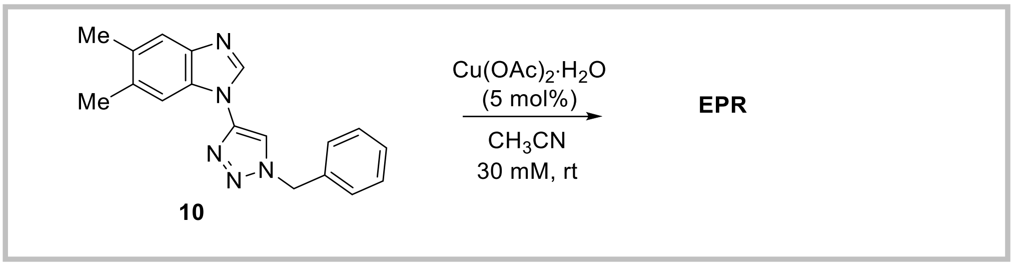

Scheme S32. Addition of $Cu(OAc)_2{\cdot}H_2O$ to triazole **10**.

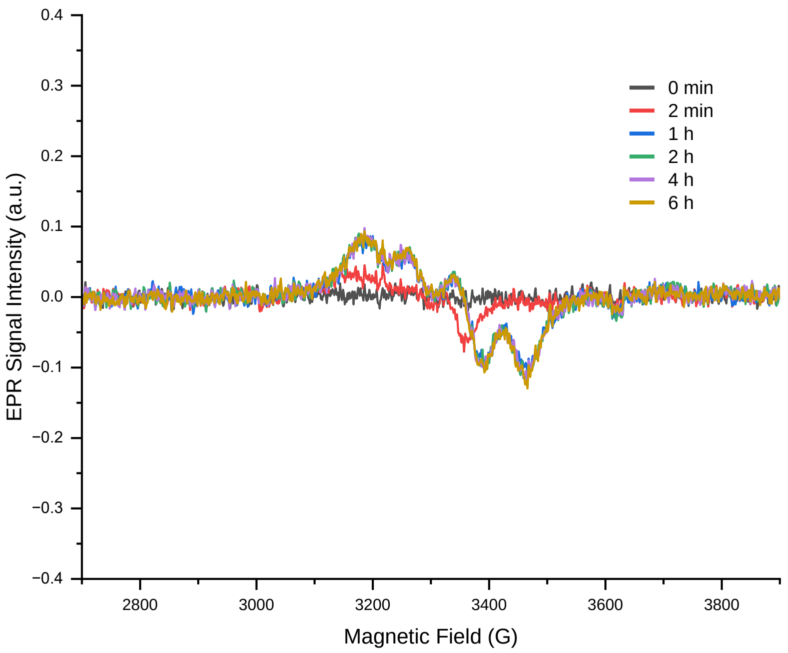

Figure S110. Stacked EPR spectra of a mixture of triazole **10** (30 mM) and $Cu(OAc)_2{\cdot}H_2O$ (1.5 mM) in CH_3CN over the course of 6 h (time 0 (black line), 2 min (red line), 1 h (blue line), 2 h (green line), 4 h (purple line), 6 h (yellow line)).

5 Single Crystal X-ray Diffraction

Data were measured with Oxford Diffraction Gemini S and with Rigaku Synergy-i instruments for compounds **16** and **13** respectively. Both used Cu Kα(λ = 1.54184 Å) radiation. In all cases, data collection and processing used CrysalisPro software.[2] The structures were refined to convergence on F^2 using all independent reflections and SHELXL as implemented within WinGX.[3, 4] In all structures the non-hydrogen atoms were refined anisotropically and hydrogen atoms were placed in idealised positions and refined in riding modes. Samples of **16** were found to be twinned by a 180° rotation about the 100° direction. This structure was thus refined against a hklf 5 formatted reflection file, and the twin ratio refined to 0.4882(14):0.5118(14). Selected crystallographic data and refinement parameters are presented in Table S21. Deposition numbers 2274319 and 2274320 contain the full supplementary crystallographic data for this paper in cif format. These data are provided free of charge by the joint Cambridge Crystallographic Data Centre and Fachinformationszentrum Karlsruhe Access Structures service www.ccdc.cam.ac.uk/structures.

Table S21. Crystallographic data and refinement parameters.

**Compound**	**16**	**13**
**CCDC**	2274319	2274320
**Formula**	$C_{44}H_{46}Cu_2N_{10}O_8$	$C_{48}H_{72}Cu_2N_4O_8Si_2$
**Form. Wt.**	969.99	1016.35
**Space Group**	$P2_1/c$	P-1
**Crystal system**	Monoclinic	Triclinic
**Temp. (K)**	150(2)	100(2)
**a (Å)**	11.9530(15)	8.5416(1)
**b (Å)**	23.633(2)	11.7320(1)
**c (Å)**	8.1920(8)	13.2393(1)
**α (°)**	90	96.411(1)
**β (°)**	108.157(12)	98.152(1)
**γ (°)**	90	99.188(1)
**Volume (Å^3)**	2198.9(4)	1284.153(2)
**Z**	2	1
**Z'**	0.5	0.5
**Measured Reflections**	15931*	45754
**Unique Reflections**	7942	4961
**2θmax (°)**	137.98	142.666
**R_{int}**	0.1434*	0.0493
**Observed Reflections [I>2σI]**	3451	4730
**No. Parameters**	294	299
**S**	0.793	1.089
**R [on *F*, obs refs only]**	0.0532	0.0403
**ωR [on F^2, all data]**	0.1623	0.1190

**Largest diff. peak /hole (eÅ^{-3})**	0.456/-0.331	0.911/-0.850

*Value prior to twin treatment.

6 Analytical RP-HPLC

6.1 Calibration

Stock solutions were prepared in MeCN and according to Table S22.

Table S22. Calibration stock solutions in MeCN

Type	Analyte stock				IS Stock
Species	Ynamine **5**	Triazole **10**	Diyne **15**	BnN_3 **2**	1,3,5-TMB
MW ($g \cdot mol^{-1}$)	170.22	303.37	338.41	133.15	168.19
Volume (mL)	50.0	50.0	50.0	50.0	50.0
Concentration (mM)	1.50	1.50	0.0625	1.50	5.00
Mass (mg)	12.8	22.8	1.10	10.0	42.0

The premade stock solutions were used to prepare calibration curves in MeCN and according to Table S23.

Table S23. Calibration stock solution dilution table

#	Total volume (µL)	Volume stock (µL)	ynamine (mM)	Triazole (mM)	Diyne (µM)	BnN_3 (mM)	V IS Stock (µL)	IS (mM)	V Solvent (µL)
1	500	400	1.20	1.20	50.0	1.20	100	1.00	0.00
2	500	360	1.08	1.08	45.0	1.08	100	1.00	40.0
3	500	280	0.84	0.84	35.0	0.84	100	1.00	120
4	500	200	0.60	0.60	25.0	0.60	100	1.00	200
5	500	120	0.36	0.36	15.0	0.36	100	1.00	280
6	500	40.0	0.12	0.12	5.00	0.12	100	1.00	360
7	500	0.00	0.00	0.00	0.00	0.00	100	1.00	400

The prepared samples were injected undiluted. Results were plotted in ratios, in which:

C_A = concentration of the analyte

C_{IS} = concentration of the internal standard

R_A = response of the analyte

R_{IS} = response of the internal standard

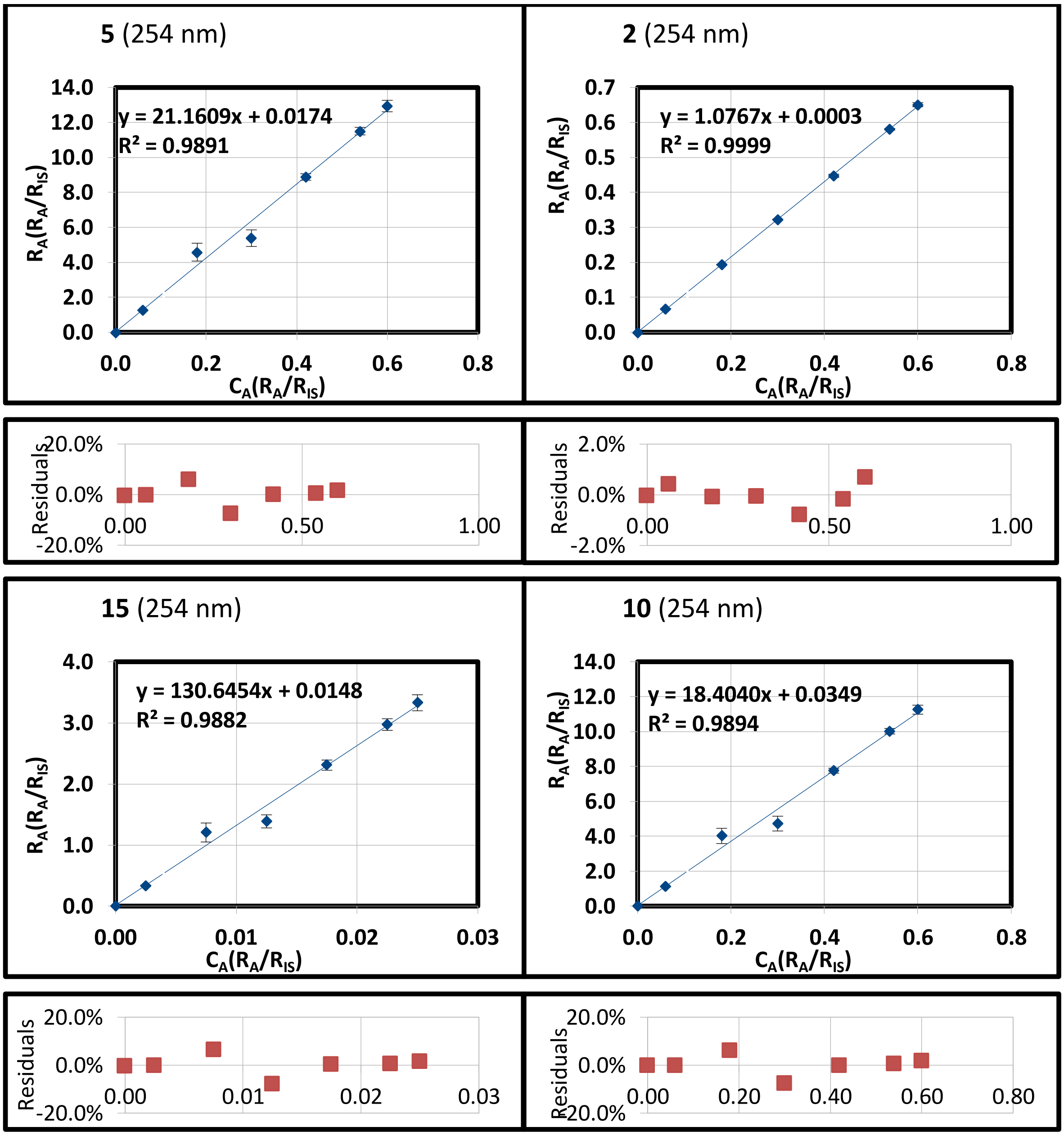

Figure S111. HPLC calibration curves and residuals.

6.2 Glaser-Hay reaction monitoring procedure A

The following protocol describes an example of a time course experiment that was used to monitor Glaser-Hay reactions. Stock solutions were prepared in MeCN according to Table S24.

Table S24. Exemplary time course stock solution preparation.

Stock	Analyte		Catalyst
Species	Ynamine **5**	1,3,5-TMB	$Cu(OAc)_2 \cdot H_2O$
MW ($g \cdot mol^{-1}$)	170.22	168.19	199.65
Concentration (mM)	77.5	77.5	15.5
Volume (mL)	1.00		2.00
Mass (mg)	13.2	13.0	6.20

To a stirring solution of analyte stock (400 µL) in a small vial containing a magnetic stir bar was added catalyst stock (100 µL) to reach an end concentration of 62.0 mM alkyne, 62.0 mM 1,3,5-TMB and catalyst solution. After the addition of catalyst, 10.0 µL aliquots were taken at regular intervals, and diluted to 1.00 mM with a quench solution (610 µL, 49/33/18 v/v% MeCN, water, EDTA solution (10% in water pH ≈ 10.0, NaOH)). To a second small vial was added analyte stock (400 µL) and solvent (100 µL), a 10.0 µL aliquot was taken and diluted in to 1mM with a quench solution (610 µL, 49/33/18 v/v% MeCN, water, EDTA solution (10% in water pH ≈ 10.0)) that was used as a t = 0 measurement.

6.3 (3+2) Cycloaddition reaction monitoring procedure B

The following protocol describes an example of a time course experiment that was used to monitor (3+2) cycloaddition reactions. Stock solutions were prepared in MeCN, according to Table S25.

Table S25. Exemplary time course stock solutions preparation.

Stock		Analyte		Catalyst
Species	Ynamine **5**	Azide **2**	1,3,5-TMB	Cu(OAc)$_2$·H$_2$O
MW (g·mol^{-1})	170.22	130.15	168.19	199.65
Concentration (mM)	77.5	77.5	77.5	15.5
Stock volume (mL)		1.00		2.00
Mass (mg)	13.2	10.3	13.0	6.2
Density (g·mL^{-1})		1.07		
Volume (µL)		9.63		

To a stirring solution of analyte stock (400 µL) in a small vial containing a magnetic stir bar was added catalyst stock (100 µL) to reach an end concentration of 62.0 mM alkyne, 62.0 mM azide, 62.0 mM 1,3,5-TMB and catalyst. After the addition of catalyst, 10.0 µL aliquots were taken at regular intervals, and diluted to 1.00 mM with a quench solution (610 µL, 49/33/18 v/v% MeCN, water, EDTA solution (10% in water pH ≈ 10, NaOH)). To a second small vial was added analyte stock (400 µL) and solvent (100 µL), a 10.0 µL aliquot was taken and diluted in to 1mM with a quench solution (610 µL, 49/33/18 v/v% MeCN, water, EDTA solution (10% in water pH ≈ 10)) that was used as a t = 0 measurement.

6.4 Stoichiometry and the formation of compound 15

In order to understand the stoichiometry of diyne **15** formation of **5** using Cu(OAc)$_2$·H$_2$O through the Glaser-Hay reaction, and the formation of Cu(I), we describe the maximum theoretical conversion to **15** possible. Throughout this project, data was usually benchmarked against 5 mol% Cu(OAc)$_2$·H$_2$O. The Glaser-Hay reaction in general is described as:

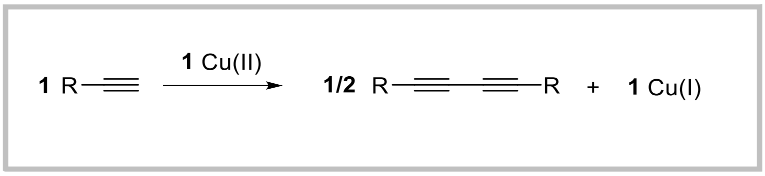

For ynamine **5** and diyne **15**:

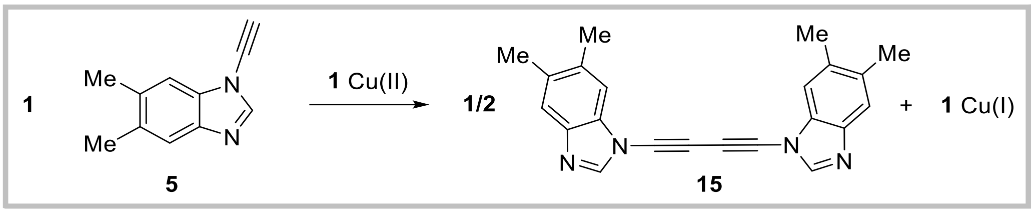

Thus:

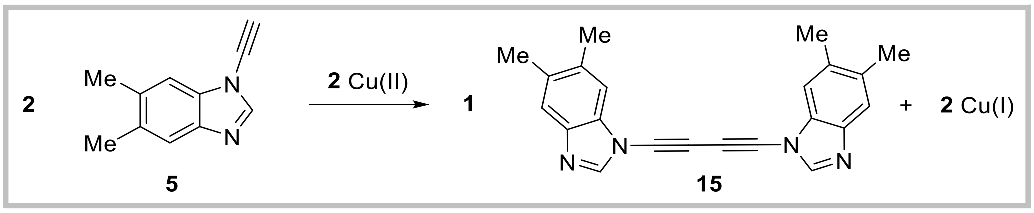

And therefore:

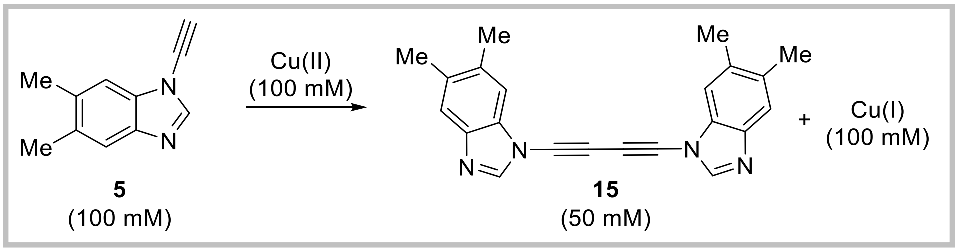

And finally percentagewise for 5 mol%:

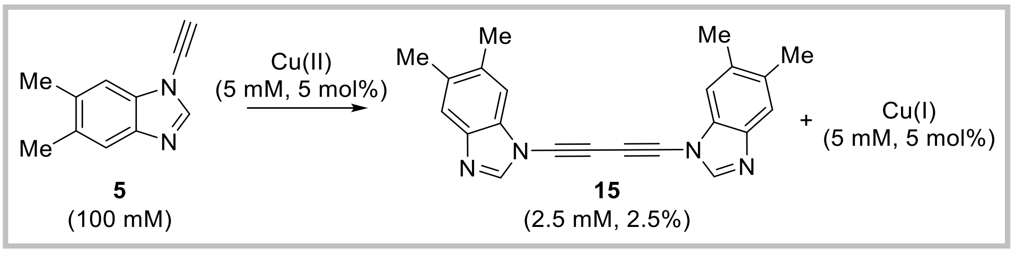

Meaning that if 5 mol% Cu(II) is used, a 2.5% formation of **15** corresponds to a full conversion of Cu(II) to Cu(I).

6.5 Copper loading and compound 15 formation in MeCN

The aromatic ynamine was reacted with various loadings of $Cu(OAc)_2{\cdot}H_2O$ to monitor the formation of diyne **15** at a concentration of 62 mM in MeCN according to protocol **A**. Catalyst stock solutions were diluted to reach the desired catalyst loading before addition. The loading of catalyst was ranged from 0.50 – 50.0 mol%.

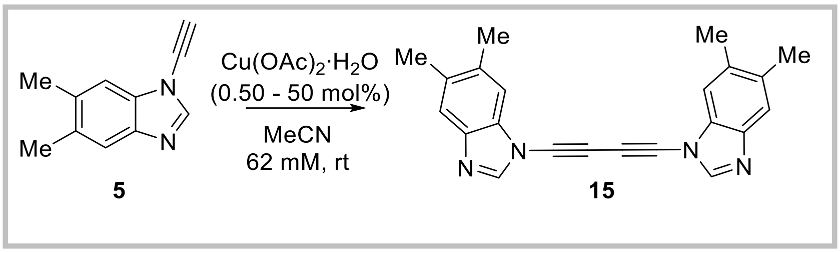

Scheme S33. Screening diyne **15** formation in various loadings of $Cu(OAc)_2{\cdot}H_2O$.

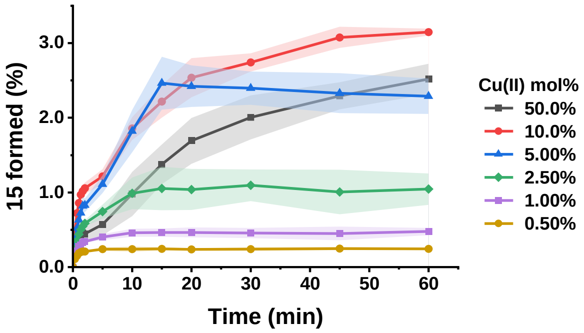

Figure S112. Screening formation of diyne **15** varying $Cu(OAc)_2{\cdot}H_2O$ loading from 0.50 – 50.0 mol% in MeCN. Monitored by sampling aliquots from the reaction mixture that were quenched in an EDTA solution that were subsequently analysed by RP-HPLC. Reaction conditions: **5** (62 mM), $Cu(OAc)_2{\cdot}H_2O$ (0.50 – 50.0 mol%) in MeCN, final volume 500 µL. 1,3,5-TMB (62 mM) was used as an internal standard. Shaded regions correspond to experimental error.

6.6 Effect of water on compound 15 formation

The aromatic ynamine was reacted with 2.5 and 5 mol% $Cu(OAc)_2{\cdot}H_2O$ in various amounts of water to monitor the formation of diyne **15** at a concentration of 62 mM in MeCN according to protocol **A**. The reagent stock solutions were adjusted accordingly to reach the desired concentration of water prior to adding the catalyst solution.

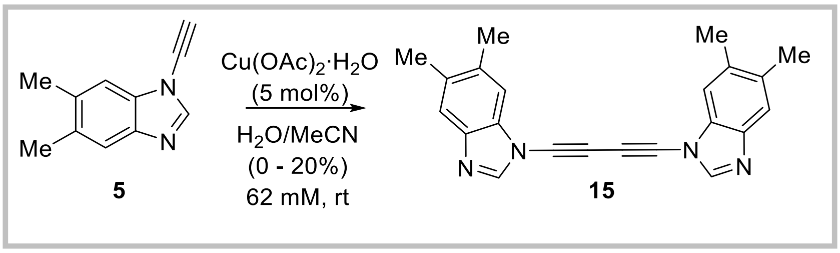

Scheme S34. Screening diyne **15** formation in various concentrations of water using 2.5 or 5 mol% $Cu(OAc)_2{\cdot}H_2O$.

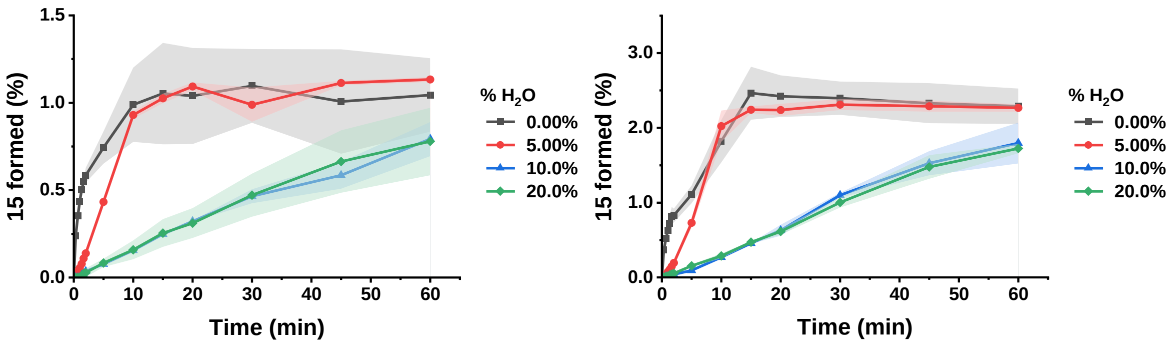

Figure S113. Screening formation of diyne **15** using 2.5 (left) and 5 mol% (right) $Cu(OAc)_2{\cdot}H_2O$ in MeCN with varying amounts of water. Monitored by sampling aliquots from the reaction mixture that were quenched in an EDTA solution that were subsequently analysed by RP-HPLC. Reaction conditions: **5** (62 mM), $Cu(OAc)_2{\cdot}H_2O$ (2.5 or 5 mol%) and water (0 – 20%) in MeCN, final volume 500 µL. 1,3,5-TMB (62 mM) was used as an internal standard. Shaded regions correspond to experimental error.

6.7 Monitoring the ynamine-azide (2+3) cycloaddition and the formation of compound 10 and 15 in MeCN

The aromatic ynamine **5** was reacted with **2** and 2.5 or 5 mol% $Cu(OAc)_2{\cdot}H_2O$ in various amounts of water to monitor the formation of triazole **10** and diyne **15** at a concentration of 62 mM in MeCN according to protocol **B**. The reagent stock solutions were adjusted accordingly to reach the desired concentration of water prior to adding the catalyst solution.

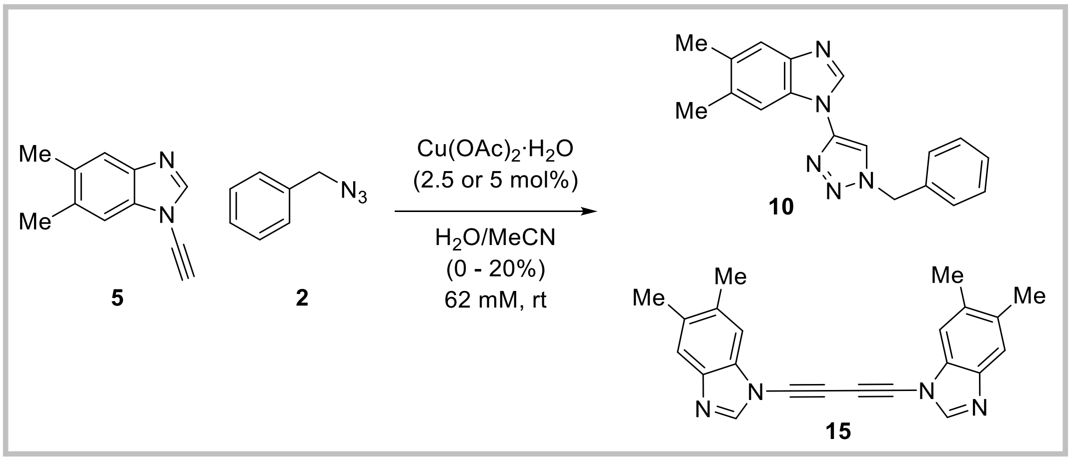

Scheme S35. Screening triazole **10** and diyne **15** formation in various concentrations of water in MeCN using 2.5 or 5 mol% $Cu(OAc)_2{\cdot}H_2O$.

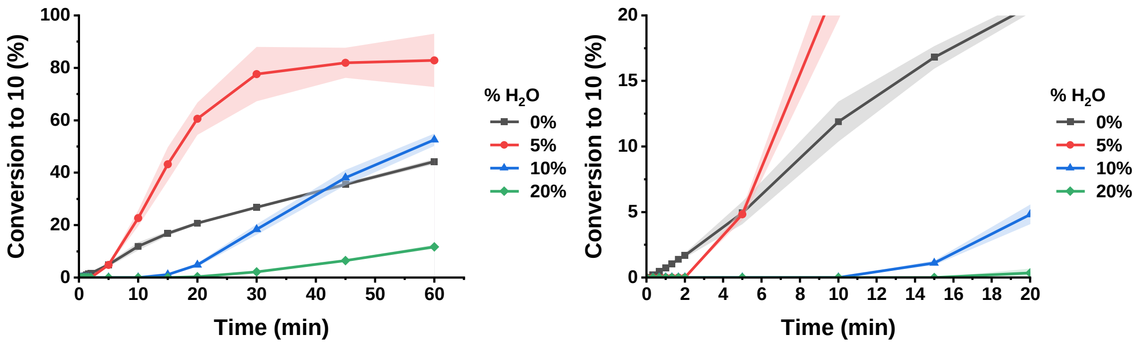

Figure S114. Monitoring the (3+2) cycloaddition between **5** and **2** and the formation of triazole **10** using 2.5 mol% $Cu(OAc)_2 \cdot H_2O$ in MeCN with varying amounts of water. Left, full graph. Right, zoom. Monitored by sampling aliquots from the reaction mixture that were quenched in an EDTA solution that were subsequently analysed by RP-HPLC. Reaction conditions: **5** (62 mM), **2** (62 mM), $Cu(OAc)_2 \cdot H_2O$ (2.5 mol%) and water (0 – 20%) in MeCN, final volume 500 µL. 1,3,5-TMB (62 mM) was used as an internal standard. Shaded regions correspond to experimental error.

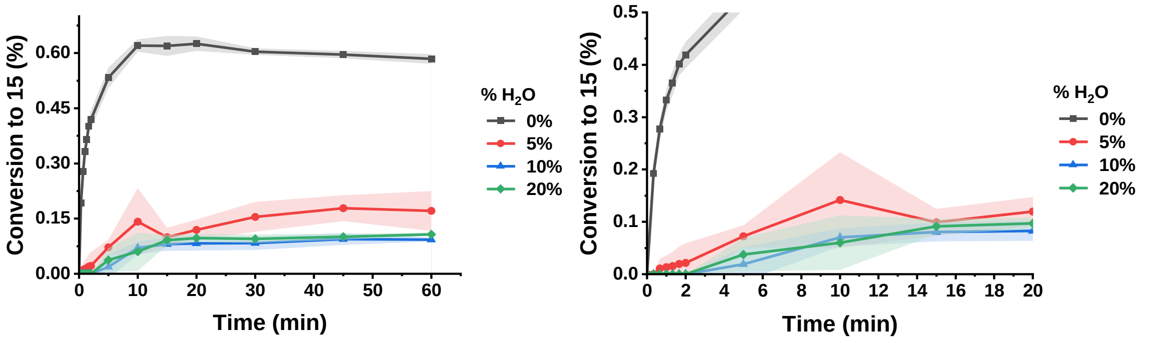

Figure S115. Monitoring the (3+2) cycloaddition between **5** and **2** and the formation of diyne **15** using 2.5 mol% $Cu(OAc)_2 \cdot H_2O$ in MeCN with varying amounts of water. Left, full graph. Right, zoom. Monitored by sampling aliquots from the reaction mixture that were quenched in an EDTA solution that were subsequently analysed by RP-HPLC. Reaction conditions: **5** (62 mM), **2** (62 mM), $Cu(OAc)_2 \cdot H_2O$ (2.5 mol%) and water (0 – 20%) in MeCN, final volume 500 µL. 1,3,5-TMB (62 mM) was used as an internal standard. Shaded regions correspond to experimental error.

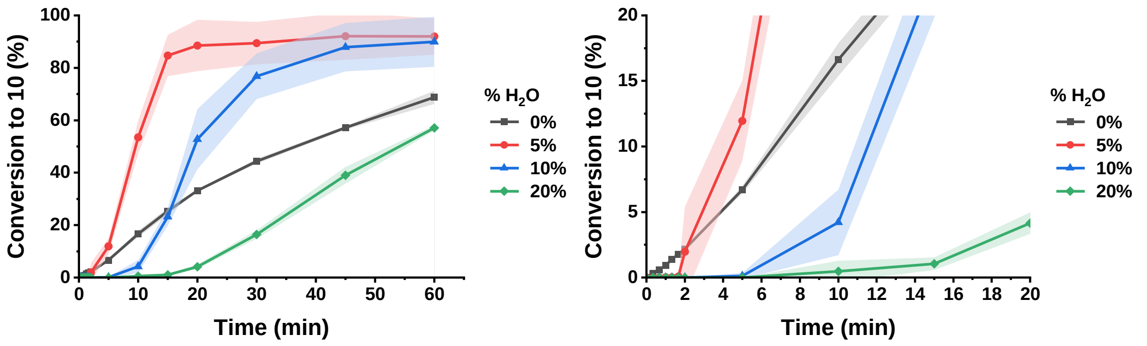

Figure S116. Monitoring the (3+2) cycloaddition between **5** and **2** and the formation of triazole **10** using 5 mol% $Cu(OAc)_2{\cdot}H_2O$ in MeCN with varying amounts of water. Left, full graph. Right, zoom. Monitored by sampling aliquots from the reaction mixture that were quenched in an EDTA solution that were subsequently analysed by RP-HPLC. Reaction conditions: **5** (62 mM), **2** (62 mM), $Cu(OAc)_2{\cdot}H_2O$ (5 mol%) and water (0 – 20%) in MeCN, final volume 500 µL. 1,3,5-TMB (62 mM) was used as an internal standard. Shaded regions correspond to experimental error.

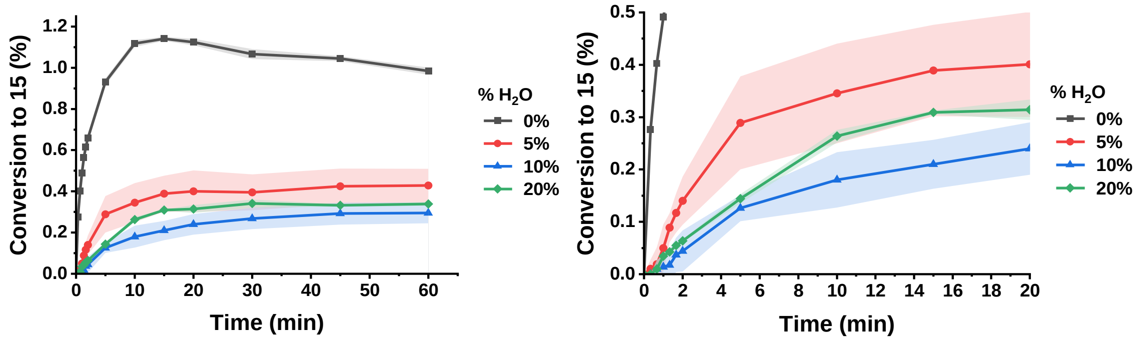

Figure S117. Monitoring the (3+2) cycloaddition between **5** and **2** and the formation of diyne **15** using 5 mol% $Cu(OAc)_2{\cdot}H_2O$ in MeCN with varying amounts of water. Left, full graph. Right, zoom. Monitored by sampling aliquots from the reaction mixture that were quenched in an EDTA solution that were subsequently analysed by RP-HPLC. Reaction conditions: **5** (62 mM), **2** (62 mM), $Cu(OAc)_2{\cdot}H_2O$ (5 mol%) and water (0 – 20%) in MeCN, final volume 500 µL. 1,3,5-TMB (62 mM) was used as an internal standard. Shaded regions correspond to experimental error.

6.8 Monitoring the sequential addition of substrates post completed (3+2) cycloaddition reaction in CD_3CN

The unexpected observation that **10** formed a Cu complex by chelation at the apical site of $Cu(OAc)_2{\cdot}H_2O$ led us to explore the catalytic competency of this species after the (3+2) cycloaddition is complete. Besides monitoring the formation of **10**, we were also interested in

the formation of diyne **15**. This was interrogated by first running the (3+2) cycloaddition with unlabelled substrates, followed by the addition of a second equivalent of isotopically labelled ynamine (**5-$^{13}C_2$-$^{15}N_2$**) and **2** was added to the reaction when the initial cycloaddition was complete. Thereafter, aliquots would be analysed by HPLC and LC-MS. For constancy and to make the data comparable to NMR conditions, CD_3CN was used.

Stock solutions of reagents were prepared according to Table S26 in CD_3CN:

Table S26. Stock solutions prepared in CD_3CN.

Entry	Species	Concentration (mM)
1	Ynamine **5**	77.5
2	Benzyl azide **2**	77.5
3	$Cu(OAc)_2$	3.875
4	4,4'-sulfinylbis(methylbenzene)	10
5	Ynamine **5-$^{13}C_2$-$^{15}N_2$**	250
6	Benzyl azide **2** 2nd addition	250

Ynamine **5** (77.5 mM, 100 µL), benzyl azide **2** (77.5 mM, 100 µL) and 4,4'-sulfinylbis(methylbenzene) (10 mM, 100 µL) and CD3CN (100 µL) were added to a 1.5 mL glass vial. The reaction was initiated by the addition of $Cu(OAc)_2{\cdot}H_2O$ (3.875 mM, 100 µL). The vial was then capped with a lid and stirred at 300 K for 17 h. Then labelled ynamine **5-$^{13}C_2$-$^{15}N_2$** (250 mM, 31 µL) and/or benzyl azide **2** (250 mM, 31 µL) were added and the reaction was stirred at 300K for 7 h. The reaction was sampled and analysed by HPLC and LC-MS after 1 h, 3 h and 24 h. Samples were prepared for analysis by taking a 10 µL aliquot and then quenched into 190 uL (0.1% TFA, H_2O/MeCN = 1:1) and analysed on a Shimadzu Prominence HPLC (2.5 µL flow cell) and Advion LC-MS.

Shimadzu HPLC method:

Column: Phenomenex Kinetex® 50 x 3 mm, 2.6 µm.

Column Temperature: 50 °C

Flow rate: 1 mL·min^{-1}

Injection volume: 5 µL

Wavelength: 254 nm

Buffer A:	0.1% TFA in H_2O
Buffer B:	0.1% TFA in MeCN
Gradient:	5% B to 95% B in 1 min, 95% B for 0.7min, 95% B to 5% B in 0.2min then 5% B for 1.7min

<u>HPLC results:</u>

Table S27. HPLC results extracted from chromatogram integrals.

	Relative area (Diyne **15** / 4,4'-sulfinylbis(methylbenzene))		Post addition **15** increase (Δ%)
Sample time	17 h (**5** + **2** only)	1 h post addition of substrate(s)	
Additive			
**2**	0.276482	0.27489	- 0.60
**5-^{13}C$_2$-^{15}N$_2$** + **2**	0.274636	0.429773	56.5
**5-^{13}C$_2$-^{15}N$_2$**	0.279967	0.554307	98.0

<u>Advion LC-MS method:</u>

Column: Phenomenex Kinetex® 30 x 2.1 mm, 2.6 µm.

Column Temperature: 40 °C

Flow rate: 0.2 mL·min^{-1}

Injection volume: 5 µL

Wavelength: 254 nm

Buffer A: 0.1% formic acid in H_2O

Buffer B: 0.1% formic acid in MeCN

Gradient: 5% B for 0.3 min, 5% B to 95% B in 8.7 min, 95% B for 1.5 min, 95% B to 5% B in 0.2 min then 5% B for 1.3 min

<u>LC-MS results:</u>

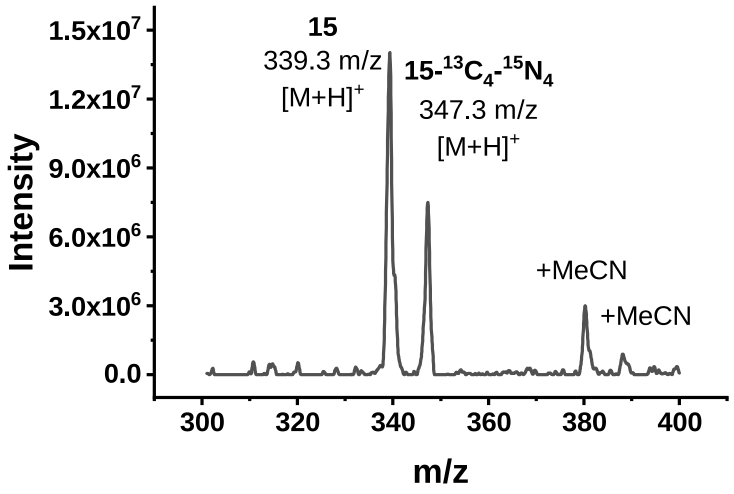

Figure S118. LC-MS results of the mass spectrum corresponding to UV detected peak of **15** 1 hour after the addition of **5-$^{13}C_2$-$^{15}N_2$** and **2** to a completed reaction of **5** and **2**.

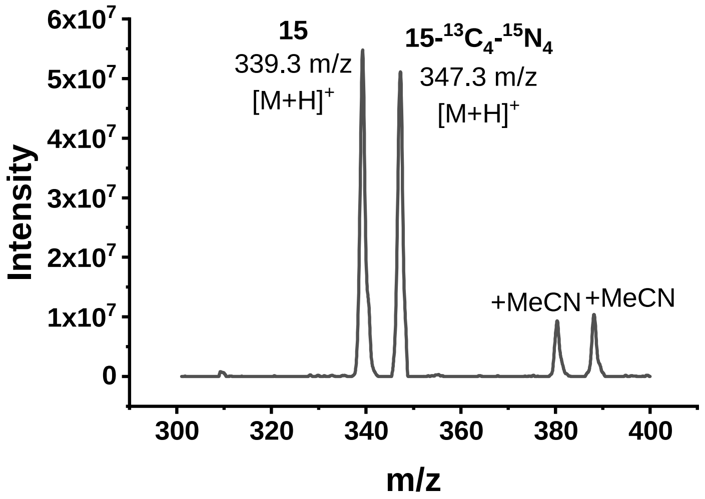

Figure S119. LC-MS results of the mass spectrum corresponding to UV detected peak of **15** 1 hour after the addition of **5-$^{13}C_2$-$^{15}N_2$** to a completed reaction of **5** and **2**.

7 MS investigation of potential copper Ynamine-TIPS complexes

Mass spectrometry was carried out on an Advion Expression L CMS instrument via direct infusion. Stock solutions of **14** (YnaTIPS) (68.8 mM), $Cu(OAc)_2{\bullet}H_2O$ (31 mM) and CuOAc (31 mM, suspension) in MeCN were prepared and diluted to the appropriate concentrations with MeCN (see table below). Aliquots were further diluted with MeCN before injection into the mass spectrometer.

Table S28. Table of stock solutions and concentrations.

Condition	[YnamineTIPS] (mM)	[Cu] (mM)	Dilution for MS
1	-	31	1 in 100
2	62	3.1	1 in 100
3	6.2	3.1	1 in 100

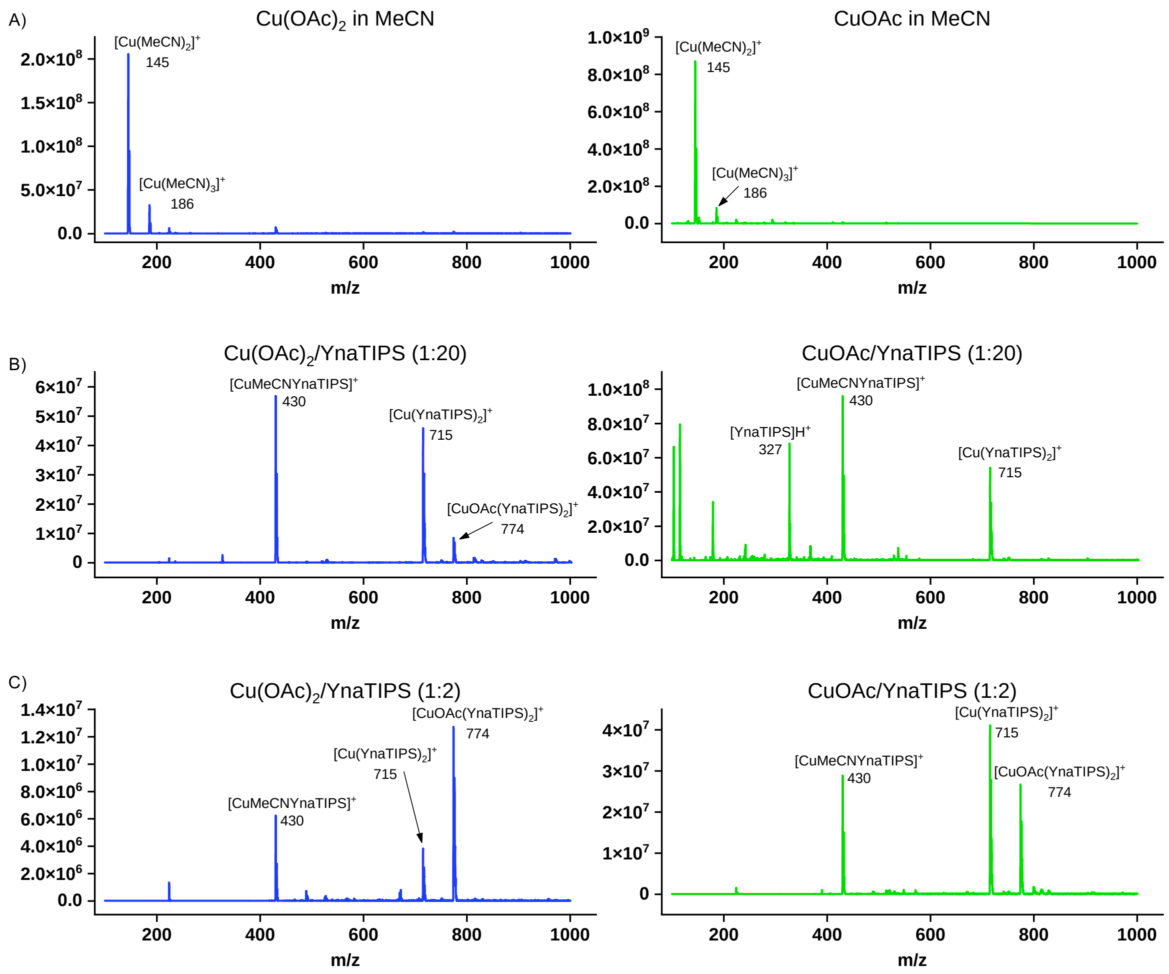

Figure S120. ESI mass spectrometry of $Cu(OAc)_2 \cdot H_2O$, CuOAc and their associated complexes with ynamine **14**. A) ESI-MS spectra of $Cu(OAc)_2 \cdot H_2O$ and CuOAc in MeCN. B) ESI-MS spectra of $Cu(OAc)_2 \cdot H_2O$ and CuOAc with ynamine **14** in a ratio of 1:20 in MeCN. C) ESI-MS spectra of $Cu(OAc)_2 \cdot H_2O$ and CuOAc with ynamine **14** in a ratio of 1:2 in MeCN.

8 pH measurements of HDE experiments

Stock solutions of ynamine **15** (100 mM) and copper salts (62 mM) were prepared in MeCN. Then 620 µL of ynamine **15** stock were mixed with 50 µL MiliQ H_2O and 50 µL copper solution to achieve final concentrations of ynamine **15** (62 mM) and copper salt (3.1 mM). In case were NaOAc was added, 50 µL of NaOAc (62 mM in H_2O) was added instead of H_2O to achieve a final concentration of 3.1 mM (5 mol%). In experiments where AcOH was used, 0.177 µL were added straight to the reaction to achieve a final concentration of 3.1mM (5 mol%).

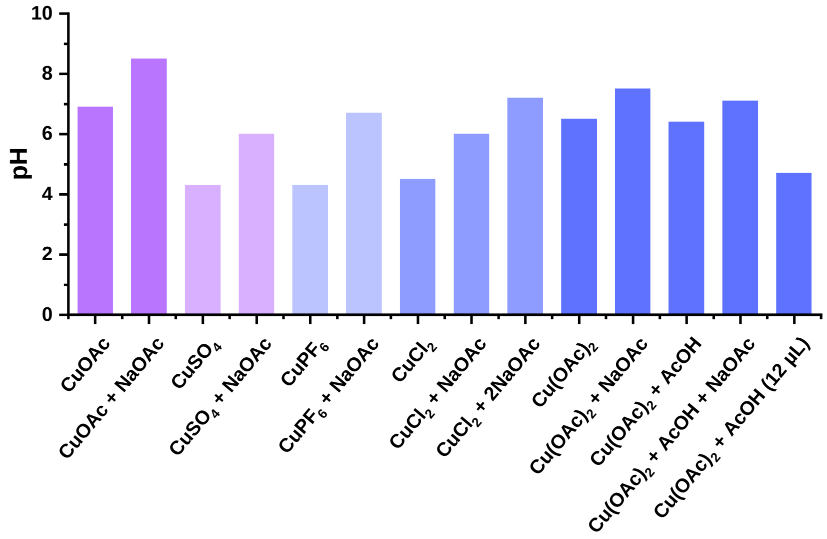

Figure S121. Influence of difference copper salts and additives on the pH of an ynamine **15** solution in MeCN/H_2O (9:1). $CuPF_6$ = $[Cu(MeCN)_4]PF_6$.

9 CuAAC in THF

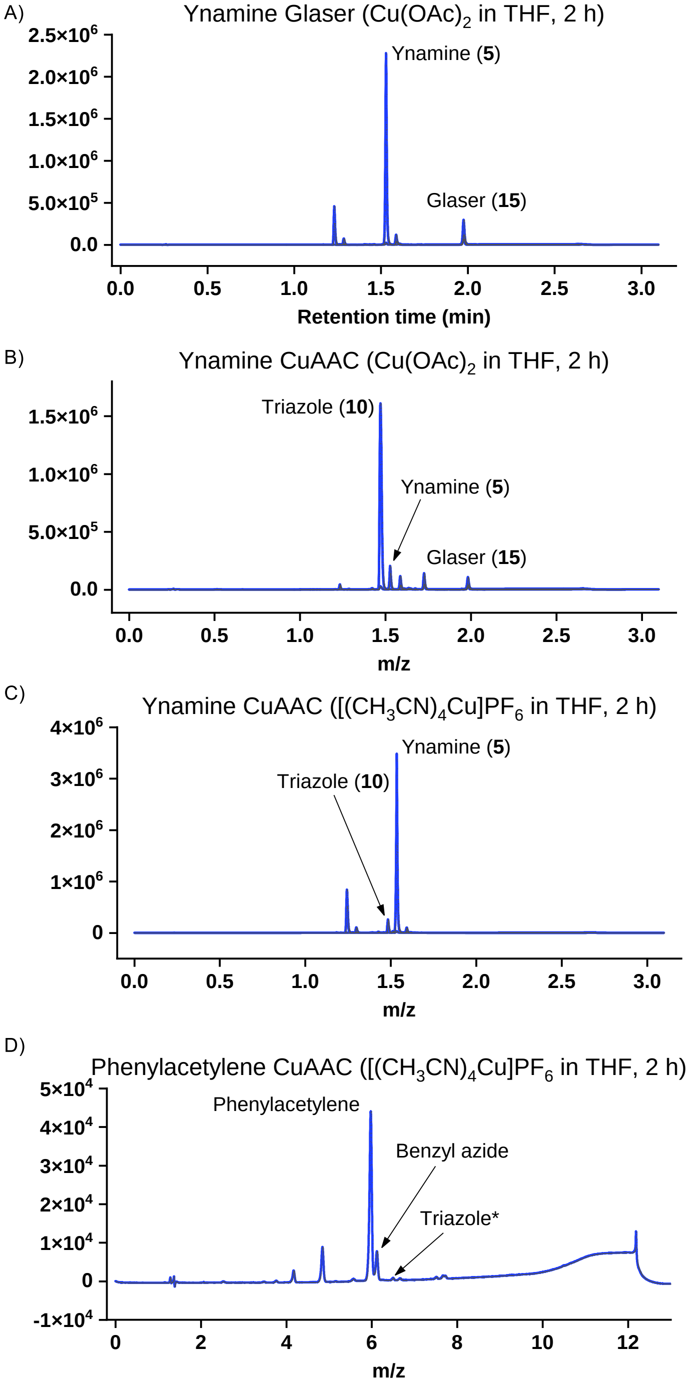

Figure S122. Ynamine CuAAC using THF as the solvent. A) Ynamine **5** (62 mM) and $Cu(OAc)_2{\bullet}H_2O$ (5 mol %) in THF. B) Ynamine **5** (62 mM), benzyl azide (62 mM) and $Cu(OAc)_2{\bullet}H_2O$ (5 mol %) in THF. C) Ynamine **5** (62 mM), benzyl azide (62 mM) and $[(CH_3CN)_4Cu]PF_6$ (5 mol %) in THF. D) Phenylacetylene (62 mM), benzyl azide (62 mM) and $[(CH_3CN)_4Cu]PF_6$ (5 mol%) in THF, *retention time confirmed by external standard.

10 Synthetic procedures

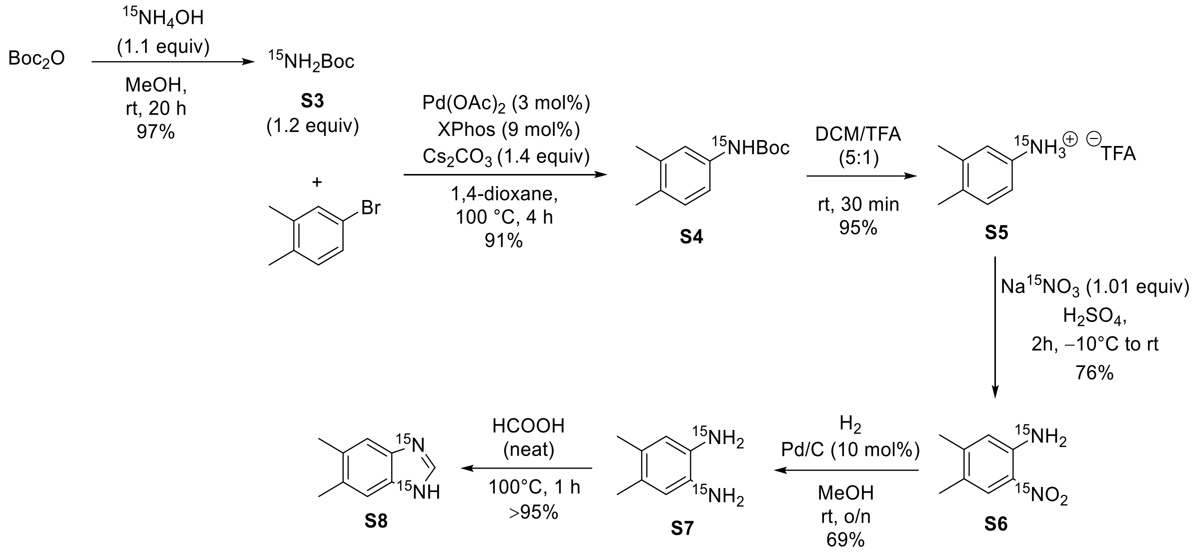

Scheme S36 Synthetic pathway for the synthesis of **S8**.

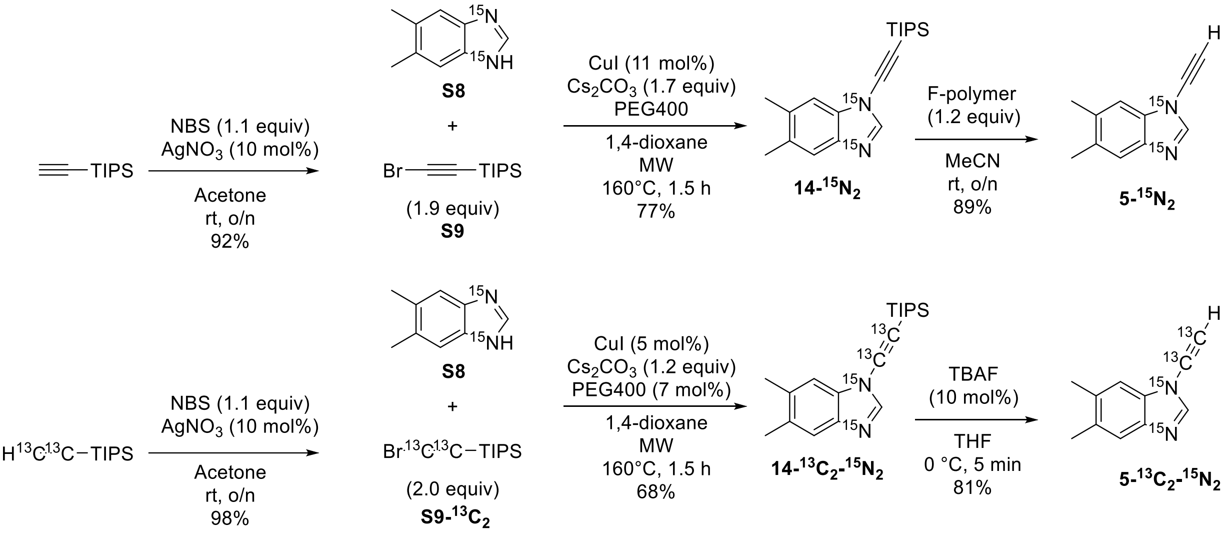

Scheme S37 Synthetic pathway for the synthesis of **5-$^{15}N_2$** and **5-$^{13}C_2$-$^{15}N_2$**

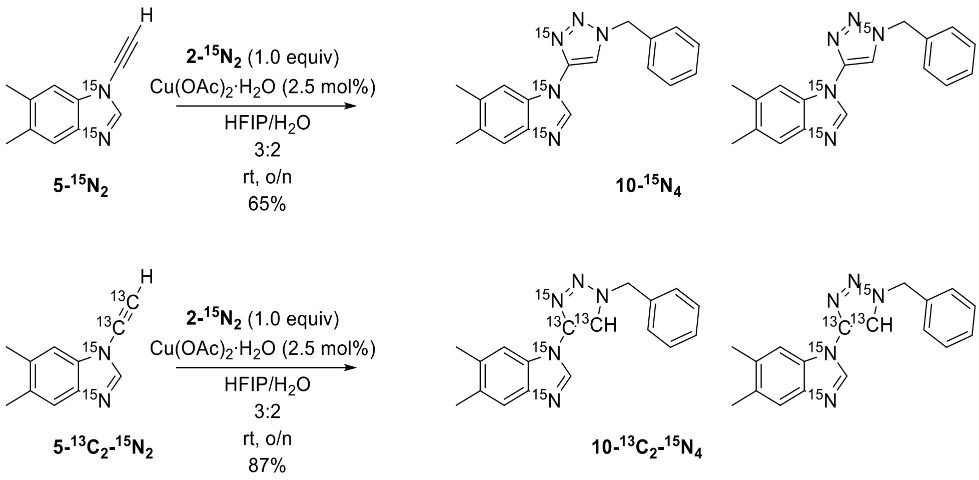

Scheme S38 Synthetic pathway for the synthesis of **10-^{15}N$_4$** and **10-^{13}C$_2$-^{15}N$_4$**

Benzyl azide **2**

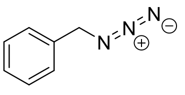

To round bottom flask charged with a magnetic stir bar was added benzyl bromide (206 mg, 1.44 g·mL^{-1}, 297 µL, 171.04 g·mol^{-1}, 2.50 mmol, 1.00 equiv), a mixture of water/acetone (1:4, 50.0 mL), and sodium azide (179 mg, 65.01 g·mol^{-1}, 2.75 mmol, 1.10 equiv). The resulting suspension was stirred at ambient temperature for 24 hours. Full starting material consumption was confirmed by TLC (PE (40 – 60), 254 nm, $Rf_{\text{benzyl bromide}}$ = 0.55). DCM (50.0 mL) was added to the mixture and the organic layer was separated. The aqueous layer was extracted with DCM (3 x 10.0 mL) and the combined organic layers were dried over Na_2SO_4. Solvent was removed in vacuo, and the azide was found sufficiently pure to use without further work up (clear oil, 287 mg, 133.15 g·mol^{-1}, 86 %)

^{1}H NMR: (400 MHz, CD_3CN) δ 7.78 – 7.06 (m, 5H, Ar*H*), 4.38 (s, 2H, C*H*$_2$).

^{13}C NMR: (101 MHz, CD_3CN) δ 136.87 (1C, Ar*C*), 129.77 (2C, Ar*C*H),129.40 (1C, Ar*C*H), 129.21 (2C, Ar*C*H), 55.18 (1C, *C*H$_2$).

TLC Rf: = 0.34 (PE (40 – 60)).

Experimental values in accordance with literature values.[7]

Benzyl azide-^{15}N **2-^{15}N$_2$**

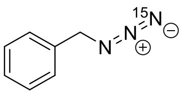

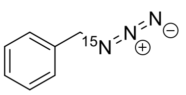

To a stirred solution of benzyl bromide (708 µL, 1.40 g·ml^{-1}, 171.04 g·mol^{-1}, 3.40 mmol, 1.00 equiv) in a 50.0 mL, H_2O/acetone mixture (1:4) was added sodium azide-$^{15}N_1$ (250 mg, 66.00 g·mol^{-1}, 3.80 mmol, 1.10 equiv). The resulting suspension was stirred at ambient temperature for 24 h. Full starting material consumption was confirmed by TLC (PE (40 – 60), 254 nm, $R_{\text{fbenzyl bromide}}$ = 0.55). 50.0 mL DCM was added to the mixture and the organic layer was separated. The aqueous layer was extracted with 3 x 10.0 mL aliquots of DCM and the combined organic layers were dried over Na_2SO_4. Solvent was removed *in vacuo* to yield the azide as a clear oil that was used without purification (378 mg, 134.15 g·mol^{-1}, 2.80 mmol, 82%).

^{1}H NMR: (500 MHz, $CDCl_3$) δ 7.43 – 7.29 (m, 5H, C*H*), 4.35 (s, 2H, C*H*$_2$).

^{13}C NMR: (101 MHz, $CDCl_3$) δ 135.52 (1C, Ar*C*), 128.98 (2C, Ar*C*H), 128.45 (1C, Ar*C*H), 128.35 (2C, Ar*C*H), 54.96 (1C, *C*H$_2$).

^{1}H-^{15}N HMBC: (400 MHz, $CDCl_3$) δ 75.77 (N^{α}), 214.3 (N^{γ}), 248.5 (N^{β}).

HRMS (ESI): $C_7H_7N_2{}^{15}N_1$ $[M]^+$ calculated 134.0605, found 134.0608.

IR (ν_{max}): 2073 (N=N=N) cm^{-1}.

TLC R_f: 0.34 (PE (40 – 60)).

Experimental values in accordance with literature values.[8]

1-Ethynyl-5,6-dimethyl-1H-benzo[d]imidazole **5**

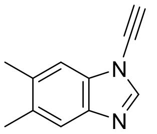

To a clean and dry 50 mL round bottom flask charged with a magnetic stir bar was added subsequently 5,6-dimethyl-1-((triisopropylsilyl)ethynyl)-1H-benzo[*d*]imidazole (1.44 g, 326.56 g·mol^{-1}, 4.40 mmol, 1.00 equiv), MeCN (25.0 mL) and fluorine on polymer (6.60 mmol, 3 mmol·g^{-1}, 2.20 g, 1.50 equiv). The mixture was stirred overnight at ambient temperature. After full conversion of the starting material determined by thin layer chromatography ($R_{f\ TIPS\text{-}ynamine}$ = 0.33 4:1 PE (40 – 60)/EtOAc, visualised at 254 nm and $KMnO_4$), the mixture was filtered, concentrated *in vacuo* and purified on 30.0 g silica and eluted with 4:1 PE (40 – 60)/EtOAc to yield the deprotected ynamine as a white powder (580 mg, 170.22 g·mol^{-1}, 3.40 mmol, 77%)

^{1}H NMR: (400 MHz, CD_3CN) δ 8.06 (s, 1H, ArC*H*), 7.51 (s, 1H, Ar-*H*), 7.37 (s, 1H, Ar-*H*), 3.67 (s, 1H, C≡C-*H*), 2.40 (s, 3H, CH_3), 2.36 (s, 3H, CH_3).

^{13}C NMR: (101 MHz, CD_3CN) δ 144.52 (1C, Ar*C*H), 141.36 (1C, Ar*C*), 135.37 (1C, Ar*C*), 134.06 (1C, Ar*C*), 133.85 (1C, Ar*C*), 121.39 (1C, Ar*C*H), 111.73 (1C, Ar*C*H), 71.28 (1C, N-*C*≡C-H), 62.91 (1C, N-C≡*C*-H), 20.39 (1C, *C*H_3), 20.19 (1C, *C*H_3).

LC-MS: (17-minute, pH = 10.5) t_R = 7.2 min, *m/z* 171.6 $[M+H]^+$.

IR (ν_{max}): 2148 (sharp, C≡C stretch) cm^{-1}.

TLC R_f : 0.3 (4:1 PE (40 – 60)/EtOAc, 254 nm + $KMnO_4$).

Experimental values in accordance with literature values.[6]

1-(ethynyl-d)-5,6-dimethyl-1H-benzo[d]imidazole **5-D**

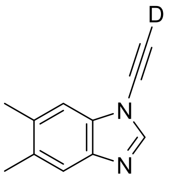

To a 7 mL glass vial charged with a stir bar was added 1-ethynyl-5,6-dimethyl-1H-benzo[*d*]imidazole (100 mg, 170.22 g·mol^{-1}, 588 µmol, 1.00 equiv) and 2.00 mL MeOD. The vial was capped, and the mixture was left to stir overnight at ambient temperature. The solvent was then removed *in vacuo* to give the

desired product as a white solid (101 mg, 171.22 g·mol^{-1}, 588 µmol, quantitative, 92% deuteration).

^{1}H NMR: (400 MHz, MeOD) δ 8.24 (s, 1H, C*H*), 7.43 (s, 1H, C*H*), 7.27 (s, 1H, C*H*), 3.98 (s, residual alkyne-H), 2.35 (s, 3H, CH_3), 2.33 (s, 3H, CH_3).

HRMS (ESI) m/z: $C_{11}H_{10}DN_2$ [M+H]$^+$ calculated 172.0979, found 172.0979.

IR (ν_{max}): 2540 (sharp, C≡C stretch) cm^{-1}.

1-ethynyl-5,6-dimethyl-1H-benzo[d]imidazole-1,3-$^{15}N_2$ **5-^{15}N$_2$**

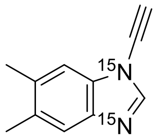

To a clean and dry 7 mL glass vial charged with a magnetic stir bar was added subsequently 5,6-dimethyl-1-((triisopropylsilyl)ethynyl)-1H-benzo[d]imidazole-$^{15}N_2$ (209 mg, 600 µmol, 328.55 g·mol^{-1}, 1.00 equiv), fluorine on polymer (233 mg, 700 µmol, 3 mmol·g^{-1}, 1.17 equiv) and MeCN (3.50 mL). The mixture was stirred overnight at ambient temperature. Thin layer chromatography confirmed depletion of starting material ($R_{f\ TIPS\text{-}ynamine\text{-}15N}$ = 0.4 (4:1 PE (40 – 60)/EtOAc, visualised with 254 nm and $KMnO_4$). The mixture was filtered, concentrated *in vacuo* and purified on 10.0 g silica and eluted with 4:1 PE (40 – 60)/EtOAc to yield de deprotected ynamine as a white powder (98.0 mg, 172.21 g·mol^{-1}, 600 µmol, 89%)

^{1}H NMR: (400 MHz, $CDCl_3$) δ 7.98 (dd, *J* = 11.6, 9.1 Hz, 1H, C*H*), 7.56 (s, 1H, C*H*), 7.35 (s, 1H, C*H*), 3.26 (d, *J* = 2.0 Hz, 1H, C≡C-*H*), 2.41 (s, 3H, CH_3), 2.38 (s, 3H, CH_3).

^{13}C NMR: (101 MHz, $CDCl_3$) δ 142.97 (d, *J* = 10.7 Hz, 1C, *C*-H), 140.39 (d, *J* = 8.5 Hz, 1C, Ar*C*), 134.52 (1C, Ar*C*), 133.30 (d, *J* = 1.5 Hz, 1C, Ar*C*), 120.98 (d, *J* = 5.8 Hz, 1C, Ar*C*-H), 111.18 (1C, Ar*C*-H), 101.42 (1C, Ar*C*), 70.81 (d, *J* = 37.9 Hz, 1C, N-*C*≡C), 61.76 (d, *J* = 5.3 Hz, 1C, N-C≡*C*), 20.62 (1C, *C*H$_3$), 20.38 (1C, *C*H$_3$).

^{15}N NMR: (41 MHz, $CDCl_3$) δ 132.00 (1N, Im*N*^{1}), 251.00 (1N, Im*N*^{3}).

IR (ν_{max}): 3196 (C≡C-H), 2148 (C≡C) cm^{-1}.

LC-MS: (17-minute, high pH) t_R = 7.2 min, *m/z* 173.2 [M+H]$^+$.

HRMS (ESI): $C_{11}H_{11}{}^{15}N_2$ [M+H]$^+$ calculated 173.0857, found 173.0866.

TLC R_f: 0.19 (4:1 PE (40 – 60)/EtOAc, 254 nm + $KMnO_4$).

1-(ethynyl-$^{13}C_2$)-5,6-dimethyl-1H-benzo[d]imidazole-1,3-$^{15}N_2$ **5-^{13}C$_2$-^{15}N$_2$**

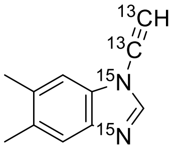

To a clean and dry 25 mL glass round bottom flask charged with a magnetic stir bar and suspended in an ice bath was added 5,6-dimethyl-1-((triisopropylsilyl)ethynyl-1,2-$^{13}C_2$)-*1H*-benzo[*d*]imidazole-1,3-$_{15}N_2$ (169 mg, 330.524 g·mol^{-1}, 511 µmol, 1.00 equiv). The flask was stoppered and purged with argon before the addition of THF (5.11 mL, anhydrous) and TBAF solution (51.1 µL, 51.1 µmol, 1.00 M in THF, 10.0 mol%). The reaction was kept at 0 °C and monitored by TLC (4:1 hexane/EtOAc). After 5 minutes, all starting material was consumed and the solvent was removed *in vacuo*. Water was added to the reaction vessel and the mixture was transferred to a 100 mL separatory funnel. The product was extracted with MTBE (3 × 25.0 mL). The organic layers were combined, and the solvent was removed *in vacuo*. The crude was purified by automatic normal phase chromatography, eluting with a 5% EtOAc/hexane to 95% EtOAc/hexane gradient over 20 CV on a 12.0 g column, yielding the desired product as a white powder (72.0 mg, 174.183 g·mol^{-1}, 413 µmol, 81%).

^{1}H NMR: (400 MHz, CD_3CN) δ 8.06 (dd, *J* = 11.8, 9.3 Hz, 1H, C*H*), 7.51 (s, 1H, C*H*), 7.37 (s, 1H, C*H*), 3.67 (ddd, *J* = 262.5, 62.8, 2.1 Hz, 1H, ArN-C≡C-*H*), 2.39 (s, 3H, CH_3), 2.36 (d, *J* = 0.8 Hz, 3H, CH_3).

^{13}C NMR: (101 MHz, CD_3CN) δ 144.49 (d, *J* = 10.2 Hz, Im*C*H), 141.33 (1C, Ar*C*), 135.37 (1C, Ar*C*), 134.06 (d, *J* = 2.1 Hz, 1C, Ar*C*), 133.82 (d, *J* = 18.5 Hz, 1C, Ar*C*), 121.38 (d, *J* = 6.1 Hz, 1C, Ar*C*H), 111.72 (1C, Ar*C*H), 71.36 (dd, *J* = 208.8, 37.0 Hz, 1C, N-*C*≡C-H), 62.75 (dd, J = 208.8, 5.7 Hz, 1C, N-C≡*C*-H), 20.37 (1C, *C*H_3), 20.17 (1C, *C*H_3).

HRMS (ESI): $C_9{}^{13}C_2H_{11}{}^{15}N_2$ [M+H]$^+$ calculated 175.0925, found 175.0925.

IR (ν_{max}): 3175 (C≡C-H), 2073 (C≡C) cm^{-1}.

TLC R$_f$: 0.19 (4:1 PE (40 – 60)/EtOAc, 254 nm + $KMnO_4$).

1-ethynyl-1H-indole **8**

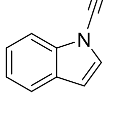

To a clean and dry 50 mL round bottom flask charged with a magnetic stir bar was subsequently added 1-((triisopropylsilyl)ethynyl)-1H-indole (500 mg, 297.51 g·mol^{-1}, 1.68 mmol, 1.00 equiv) and THF (16.8 mL). The mixture was chilled to –20 °C using a 1:3 NaCl/ice bath, and the flask was thoroughly purged with argon. To this was added dropwise tetrabutylammonium solution (168 µL, 1 M, 168 µmol, 0.10 equiv). The progress of the reaction was monitored by TLC (hexane, $Rf_{1\text{-((triisopropylsilyl)ethynyl)-1H-indole}}$ = 0.50) The mixture

was allowed to react at –20 °C until completion at 1 hour. The mixture was then transferred to a separatory funnel using Et_2O (25 mL) and water. The product was then extracted with Et_2O (2 × 25 mL), and the organic layers were subsequently combined and washed with brine. The organic layer was then dried using Na_2SO_4 and filtered into a 100 mL round bottom flask which was placed in a water bath at ambient temperature. A gentle flow of argon was applied to evaporate the solvent until dry. The crude was then purified by automatic normal phase chromatography (12.0 g silica gel, hexane, isocratic) to yield the desired product as a temperature sensitive off-white solid (237 mg, 141.17 g·mol^{-1}, 1.68 mmol, 57%).

^{1}H NMR: (400 MHz, $CDCl_3$) δ 7.64 (dt, J = 7.8, 1.0 Hz, 1H, Ar*H*), 7.61 (dq, J = 8.2, 0.9 Hz, 1H, Ar*H*), 7.37 (ddd, J = 8.3, 7.2, 1.2 Hz, 1H, Ar*H*), 7.32 – 7.19 (m, 2H, Ar*H*), 6.59 (dd, J = 3.4, 0.9 Hz, 1H, Ar*H)*, 3.17 (s, 1H, C≡C-*H*).

^{13}C NMR: (101 MHz, $CDCl_3$) δ 138.32 (1C, Ar*C*), 128.92 (1C, Ar*C*H), 128.88 (1C, Ar*C*), 127.83 (1C, Ar*C*H), 123.87 (1C, Ar*C*H), 123.83 (1C, Ar*C*H), 122.28 (1C, Ar*C*H), 122.24 (1C, Ar*C*H), 121.38 (1C, Ar*C*H), 121.34 (1C, Ar*C*H), 111.43 (1C, Ar*C*H), 111.39 (1C, Ar*C*H), 105.74 (1C, Ar*C*H), 105.70 (1C, Ar*C*H), 74.51 (1C, N-*C*≡C-H), 58.91 (1C, N-C≡*C*-H).

HRMS (ESI): $C_{10}H_8N$ $[M+H]^+$ calculated 142.0651, found 142.0652.

IR (ν_{max}): 3267 (C≡C-H), 2146 (C≡C) cm^{-1}.

TLC R_f: 0.45 (hexane, 254 nm).

Experimental values in accordance with literature values.[6]

1-(1-benzyl-1H-1,2,3-triazol-4-yl)-5,6-dimethyl-1H-benzo[d]imidazole **10**

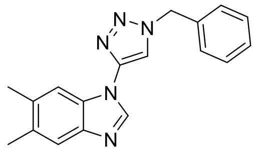

To a 7 mL glass vial charged with a magnetic stir bar was added subsequently 1-(ethynyl)-5,6-dimethyl-1H-benzo[d]imidazole (85.1 mg, 170.22 g·mol^{-1} 500 µmol, 1.00 equiv), MeCN (2.00 mL) benzyl azide (66.6 mg, 1.065 g·mol^{-1}, 133.15 g·mol^{-1}, 500 µmol, 1.00 equiv) and $Cu(OAc)_2 \cdot H_2O$ (5.00 mg, 199.65 g·mol^{-1}, 30.0 µmol, 5.00 mol%). The resulting mixture was allowed to stir overnight. The solvent was evaporated *in vacuo* and the resulting mint green solid purified over flash-silica (10.0 g, 4:1 EtOAc/PE (40 – 60)) to yield the triazole as an off white solid (26.0 mg, 303.37 g·mol^{-1}, 420 µmol, 83%).

^{1}H NMR: (400 MHz, CD_3CN) δ 8.23 (s, 1H, C*H*), 8.16 (s, 1H, C*H*), 7.55 (s, 1H, C*H*), 7.51 (s, 1H, C*H*), 7.47 – 7.33 (m, 5H, C*H*), 5.64 (s, 2H, C*H$_2$*), 2.38 (s, 3H, C*H$_3$*), 2.37 (s, 3H, C*H$_3$*).

^{13}C NMR: (101 MHz, CD_3CN) δ 143.60 (1C, N-*C*=CH-N), 143.37 (1C, Ar*C*), 141.84 (1C, Ar*C*H), 136.29 (1C, Ar*C*), 134.24 (1C, Ar*C*), 132.85 (1C, Ar*C*), 132.21 (1C, Ar*C*), 129.96 (2C, Ar*C*H), 129.55 (1C, Ar*C*H) , 129.12 (2C, Ar*C*H), 121.00 (1C, Ar*C*H), 116.10 (1C, N-C=*C*H-N), 112.51 (1C, Ar*C*H), 55.33 (1C, *C*H_2), 20.52 (1C, *C*H_3), 20.22 (1C, *C*H_3).

HRMS (ESI): $C_{18}H_{18}N_5$ $[M+H]^+$ calculated 304.1557, found 304.1555.

IR (ν_{max}): 3084 (C=C-H) 1581 (C=C) cm^{-1}.

LC-MS: (17-minute, high pH) t_R = 7.18 min, *m/z* 304.4 $[M+H]^+$

TLC R_f: 0.43 (4:1 EtOAc/PE (40 – 60), 254 nm).

Experimental values in accordance with literature values.[6]

1-(1-benzyl-1H-1,2,3-triazol-4-yl-1-^{15}N)-5,6-dimethyl-1H-benzo[d]imidazole-1,3-$^{15}N_2$ **10-$^{15}N_4$**

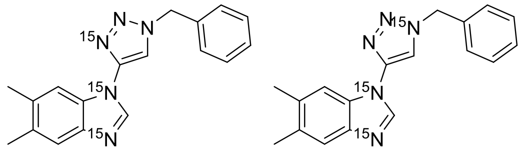

To a 7 mL glass vial charged with a magnetic stir bar was added subsequently 1-(ethynyl-*$^{13}C_2$*)-5,6-dimethyl-1H-benzo[d]imidazole-1,3-*$^{15}N_2$* (20.0 mg, 172.20 g·mol^{-1} 116 µmol, 1.00 equiv), HFIP (1.12 mL), benzyl azide-^{15}N (15.6 mg, 1.065 g·mL^{-1}, 14.6 µL 134.15 g·mol^{-1}, 116 µmol, 1.00 equiv) and a mixture of $Cu(OAc)_2{\cdot}H_2O$ in water (580 µg, 199.65 g·mol^{-1}, 2.90 µmol, 2.50 mol%, in 749 µL water). The resulting mixture was allowed to stir overnight. The solvent was evaporated *in vacuo* and the resulting mint green solid was washed with a sat. solution of EDTA (20.0 mL) in a 250 mL separatory funnel. The aqueous layer was extracted with DCM (3 × 10.0 mL). The combined organic layers were washed with brine, dried over Na_2SO_4 and filtered. The solvent was removed *in vacuo* and the remaining crude purified by automatic normal phase chromatography, eluting with a 20% EtOAc/hexane to 80% EtOAc/hexane gradient on a 4.00 g column to yield the product as a white solid (23.0 mg, 306.35 g·mol^{-1}, 217 µmol, 65%).

^{1}H NMR: (400 MHz, CD_3CN) δ 8.23 (dd, *J* = 11.8, 8.6 Hz, 1H, C*H*), 8.16 (dd, *J* = 2.9, 1.6 Hz, 1H, C*H*), 7.56 (s, 1H, C*H*), 7.52 (s, 1H, C*H*), 7.47 – 7.34 (m, 5H, C*H*), 5.64 (d, *J* = 1.2 Hz, 2H, C*H_2*), 2.39 (s, 3H, C*H_3*), 2.37 (s, 3H,C*H_3*).

^{13}C NMR: (101 MHz, CD_3CN) δ 143.66 (m, 1C, N-*C*=CH-N), 143.39, 141.76 (d, *J* = 10.63 Hz, 1C, Ar*C*), 136.30 (1C, Ar*C*), 134.26 (1C, Ar*C*), 132.86 (1C, Ar*C*), 129.97 (2C, Ar*C*), 129.56 (1C, Ar*C*), 129.13 (2C, Ar*C*), 121.00 (d, *J* = 6.32 Hz, Ar*C*-H, 1C, Ar*C*-H), 116.10 (m, 1C, N-C=*C*H-N), 112.51 (1C, Ar*C*-H), 55.32 (m, 2C, *C*H_2), 20.50 (1C, Ar*C*H_3), 20.21 (1C, Ar*C*H_3).

HRMS (ESI): $C_{18}H_{18}N_2{}^{15}N_3$ $[M+H]^+$ calculated 307.1468, found 307.1464.

IR (ν_{max}): 3078 (C=C-H) 1573 (C=C) cm^{-1}.

TLC R_f: 0.43 (4:1 EtOAc/PE (40 – 60), 254 nm).

1-(1-benzyl-1H-1,2,3-triazol-4-yl-4,5-$^{13}C_2$-1/3-^{15}N)-5,6-dimethyl-1H-benzo[d]imidazole-1,3-$^{15}N_2$ **10-$^{13}C_2$-$^{15}N_4$**

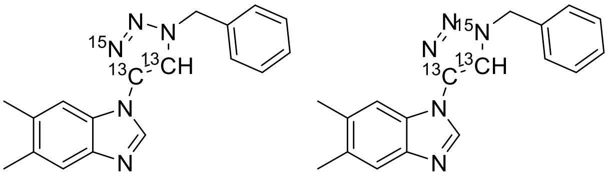

To a 7 mL glass vial charged with a magnetic stir bar was added subsequently 1-(ethynyl-$^{13}C_2$)-5,6-dimethyl-1H-benzo[d]imidazole-1,3-$^{15}N_2$ (43.5 mg, 174.183 g·mol^{-1} 250 µmol, 1.00 equiv), HFIP (2.40 mL), water (1.60 mL) benzyl azide-^{15}N (33.5 mg, 1.065 g·mL^{-1}, 31.5 µL, 134.15 g·mol^{-1}, 250 µmol, 1.00 equiv) and $Cu(OAc)_2 \cdot H_2O$ (1.25 mg, 199.65 g·mol^{-1}, 6.25 µmol, 2.50 mol%). The resulting mixture was allowed to stir at ambient temperature overnight. The solvent was removed *in vacuo* and the resulting mint green solid was washed with sat. EDTA (50.0 mL) in a 250 mL separatory funnel. The aqueous layer was extracted with DCM (3 × 20.0 mL). The combined organic layers were washed with brine, dried over Na_2SO_4 and filtered. The solvent was removed *in vacuo* and the remaining crude purified by automatic normal phase chromatography, eluting with a 20% EtOAc/hexane to 80% EtOAc/hexane gradient on a 4.00 g column to yield the product as a white solid (67.0 mg, 308.33 g mol^{-1}, 217 µmol, 87%).

^{1}H NMR: (400 MHz, CD_3CN) δ 8.23 (dd, J = 11.7, 8.6 Hz, 1H, C*H*), 8.16 (dddd, *J* = 199.6, 8.7, 2.9, 1.6 Hz, 1H, C*H*), 7.56 (s, 1H, C*H*), 7.51 (s, 1H, C*H*), 7.46 – 7.33 (m, 5H, C*H*), 5.64 (d, *J* = 3.0 Hz, 2H, CH_2), 2.38 (s, 3H, CH_3), 2.37 (s, 3H, CH_3).

^{13}C NMR: (101 MHz, CD_3CN) δ 143.56 (m, 1C, N-*C*=CH-N), 141.82 (d, *J* = 10.90 Hz, 1C, Ar*C*), 136.30 (1C, Ar*C*), 134.24 (1C, Ar*C*), 132.85 (1C, Ar*C*), 129.96 (2C, Ar*C*H), 129.55 (1C, Ar*C*H), 129.12 (2C, Ar*C*H), 121.00 (d, *J* = 6.14 Hz, 1C, Ar*C*H), 116.05 (ddt, *J* = 81.09, 9.52, 4.66 Hz, 1C, N-C=*C*H-N), 112.51 (1C, Ar*C*H), 55.32 (1C, *C*H_2), 20.50 (1C, *C*H_3), 20.22 (1C, *C*H_3).

HRMS (ESI): $C_{16}{}^{13}C_2H_{18}{}^{15}N_3$ $[M+H]^+$ calculated 309.1535, found 309.1526.

IR (ν_{max}): 3086 (C=C-H) 1531 (C=C) cm^{-1}.

TLC R_f: 0.43 (8:2 EtOAc/PE (40 – 60), 254 nm).

5,6-dimethyl-1-((triisopropylsilyl)ethynyl)-1H-benzo[d]imidazole **14**

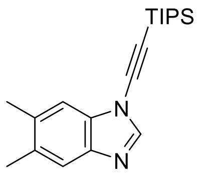

To a clean and dry 2-5 mL Biotage® microwave vial charged with a magnetic stir bar was subsequently added 5,6-dimethyl-1H-benzo[*d*]imidazole (146 mg, 146.19 g·mol^{-1}, 1.00 mmol, 1.00 equiv), Cs_2CO_3 (391 mg, 325.82 g·mol^{-1}, 1.20 mmol, 1.20 equiv) and CuI (19.0 mg, 190.45 g·mol^{-1}, 100 µmol, 10.0 mol%). After capping and an argon purge, 1,4-dioxane (5.00 mL, anhydrous) and PEG400 (36.0 µL, 10.0 µmol) were added. With stirring, the mixture was degassed under reduced pressure and purged with argon thrice over 45 minutes before the addition of (Bromoethynyl)triisopropylsilane (575 mg, 261.28 g·mol^{-1}, 2.20 mmol, 2.20 equiv). The reaction mixture was heated to 160 °C) in a Biotage® Initiator+ microwave for 60 minutes. The reaction was allowed to cool down to ambient temperature and the resulting suspension was diluted with diethyl ether (25.0 mL). The mixture was washed with saturated EDTA solution, extracted with Et_2O (3 × 15.0 mL), washed with brine, and dried over Na_2SO_4. The organic phase was concentrated *in vacuo* and purified by manual flash column chromatography (silica gel: 3 CV PE (40 – 60), further CV 10% EtOAc/PE (40 – 60)) to acquire the desired compound as a colourless oil (246 mg, 326.56 g mol^{-1}, 750 µmol, 75%).

^{1}H NMR: (400 MHz, CD_3CN) δ 8.05 (s, 1H, Ar*H*), 7.50 (s, 1H, Ar*H*), 7.31 (s, 1H, Ar*H*), 2.39 (s, 3H, C*H*$_3$), 2.36 (s, 3H, C*H*$_3$), 1.23 – 1.12 (m, 21H, SiC*H*C*H*$_3$).

^{13}C NMR: (101 MHz, CD_3CN) δ 144.47 (1C, Ar*C*H), 141.38 (1C, Ar*C*), 135.30 (1C, Ar*C*), 134.01 (1C, Ar*C*), 133.93 (1C, Ar*C*), 121.45 (1C, Ar*C*H), 111.74 (1C, Ar*C*H), 91.76 (1C, N-*C*≡C-), 72.72 (1C, N-C≡*C*-), 20.52 (1C, *C*H$_3$), 20.23 (1C, *C*H$_3$), 18.97 (6C, SiCH*C*H$_3$), 12.04 (3C, Si*C*HCH$_3$).

LC-MS: (17-minute, high pH) t_R = 11.7 min, *m/z* 327.4 $[M+H]^+$.

IR (νmax): 2177 (sharp, C≡C stretch) cm^{-1}.

TLC R_f: 0.56 (9:1 PE (40 – 60)/EtOAc, 254 nm + $KMnO_4$).

Experimental values in accordance with literature values.[6]

5,6-dimethyl-1-((triisopropylsilyl)ethynyl)-1H-benzo[d]imidazole-1,3-$^{15}N_2$ **14-^{15}N$_2$**

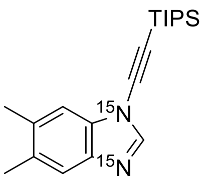

To a clean and dry 2-5 mL Biotage® microwave vial charged with a magnetic stir bar was subsequently added 5,6-dimethyl-1H-benzo[d]imidazole-$^{15}N_2$ (133 mg, 148.18 g·mol^{-1}, 900 µmol, 1.00 equiv), Cs_2CO_3 (502 mg, 325.82 g·mol^{-1}, 1.50 mmol, 1.66 equiv) and CuI (15.0 mg, 190.45 g·mol^{-1}, 100 µmol, 11 mol%). After capping and an argon purge, 1,4-dioxane (2.60 mL, anhydrous) and PEG400 (2 drops) were added. With stirring, the mixture was degassed and purged with argon thrice over 45 minutes before the addition of (bromoethynyl)triisopropylsilane (442 mg, 261.28 g·mol^{-1}, 1.70 mmol, 1.88 equiv). The reaction mixture was heated to 160 °C in a Biotage® Initiator+ microwave for 90 minutes. The reaction was allowed to cool down to ambient temperature and the resulting suspension was diluted with diethyl ether (25.0 mL). The mixture was washed with sat. EDTA solution, extracted with Et_2O (2 × 25.0 mL), washed with brine, and then dried over Na_2SO_4. The organic phase was concentrated *in vacuo* and purified by flash column chromatography (20.0 g silica gel: 3 CVs PE (40 – 60), further CVs 10% PE (40 – 60)/EtOAc) to acquire the desired compound as a colourless oil (226 mg, 328.55 g mol^{-1}, 820 µmol, 77%).

^{1}H NMR: (400 MHz, $CDCl_3$) δ 7.97 (dd, *J* = 11.6, 9.0 Hz, 1H, C*H*), 7.55 (s, 1H, C*H*), 7.29 (s, 1H, C*H*), 2.41 (s, 3H, CH_3), 2.38 (s, 3H, CH_3), 1.20 – 1.13 (m, 21H, Si(C*H*CH_3)$_3$).

^{13}C NMR: (101 MHz, $CDCl_3$) δ 143.10 (d, *J* = 10.9 Hz, 1C, Ar*C*), 140.41 (d, *J* = 8.4 Hz, 1C, Ar*C*), 134.27 (1C, Ar*C*), 133.21 (1C, Ar*C*), 133.08 (1C, Ar*C*), 120.92 (d, *J* = 5.8 Hz, 1C, Ar*C*), 111.25, 90.7 (d, *J* = 33.9 Hz, 1C, N-*C*≡C), 72.48(d, *J* = 2.5 Hz, 1C, N-C≡*C*), 20.75 (1C, *C*H_3), 20.40 (1C, *C*H_3), 18.79 (6C, Si(CH*C*H_3)$_3$), 11.39 (3C, Si(*C*HCH_3)$_3$).

LC-MS: (17-minute, high pH) t_R = 11.8 min, *m/z* 329.3 [M+H]$^+$.

HRMS (ESI): $C_{20}H_{31}{}^{15}N_2Si_1$ [M+H]$^+$ calculated 329.2192, found 329.2195.

IR (ν_{max}): 2185 (C≡C) cm^{-1}.

TLC R_f: 0.32 (9:1 PE (40 – 60)/EtOAc, 254 nm + $KMnO_4$).

5,6-dimethyl-1-((triisopropylsilyl)ethynyl-1,2-$^{13}C_2$)-1H-benzo[d]imidazole-1,3-$^{15}N_2$ **14-^{13}C$_2$-^{15}N$_2$**

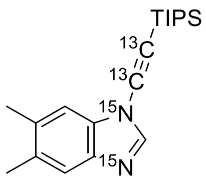

Over two clean and dry 2-5 mL Biotage® microwave vials charged with a magnetic stir bar was split 5,6-dimethyl-1H-benzo[d]imidazole-$^{15}N_2$ (222 mg, 148.18 g·mol^{-1}, 1.55 mmol, 1.00 equiv), Cs_2CO_3 (585 mg, 325.82 g·mol^{-1}, 1.80 mmol, 1.20 equiv), CuI (14.3 mg, 190.45 g·mol^{-1}, 74.9 µmol, 5.00

mol%) and PEG400 (34.4 µL, 97.3 µmol, 6.50 mol%). After capping and an argon purge, 1,4-dioxane (7.49 mL, anhydrous) was added. With stirring, the mixture was degassed *in vacuo* and purged with argon thrice over 15 minutes before the addition of (bromoethynyl-1,2-$^{13}C_2$)triisopropylsilane (778 mg, 263.26 g·mol^{-1}, 2.95 mmol, 1.97 equiv). Both reaction mixtures were heated to 160 °C in a Biotage® Initiator+ microwave for 90 minutes. The reaction was allowed to cool down to ambient temperature and the resulting suspension were combined and transferred to a 100 mL separatory funnel using both water and EtOAc. The mixture was extracted with EtOAc (3 × 25.0 mL). The combined organic layers were washed with sat. EDTA solution and brine, and then dried over Na_2SO_4. The organic phase was concentrated *in vacuo* and purified by flash column chromatography (30.0 g silica gel, 13 mL fractions. Fractions 1 to 12 were eluted with hexane. Fractions 13 to 25 were eluted with 20% EtOAc in hexane. Fractions 6 to 10 contained unreacted (bromoethynyl-1,2-$^{13}C_2$)triisopropylsilane. Fractions 21 to 25 contained the desired compound as a colourless oil (337 mg, 330.524 g mol^{-1}, 1.02 mmol, 68%).

^{1}H NMR: (400 MHz, $CDCl_3$) δ 7.96 (dd, *J* = 11.6, 9.0 Hz, 1H, C*H*), 7.55 (s, 1H, C*H*), 7.28 (s, 1H, C*H*), 2.41 (s, 3H, CH_3), 2.38 (s, 3H, CH_3), 1.17 (m, 21H, Si(C*H*CH_3)$_3$).

^{13}C NMR: (101 MHz, $CDCl_3$) δ 143.113 (d, *J* = 10.9 Hz, 1C, Ar*C*), 140.45 (dd, *J* = 8.0, 4.6 Hz, 1C, Ar*C*), 134.28 (1C, Ar*C*), 133.22 (1C, Ar*C*), 133.09 (1C, Ar*C*), 120.94 (d, *J* = 5.9 Hz, 1C, Ar*C*), 111.25 (1C, Ar*C*), 90.74 (dd, *J* = 159.9, 33.8 Hz, 1C, ArN-*C*≡C), 72.39 (dd, *J* = 159.9, 3.0 Hz, N-C≡*C*), 20.75 (1C, Ar-*C*H$_3$), 20.40 (1C, Ar-*C*H$_3$), 20.58 (d, *J* = 35.01 Hz, 6C, Si(CH*C*H$_3$)$_3$), 11.39 (d, *J* = 4.6 Hz, 3C, Si(*C*HCH$_3$)$_3$).

HRMS (ESI): $C_{18}{}^{13}C_2H_{31}{}^{15}N_2Si$ [M+H]$^+$ calculated 331.2259, found 331.2253.

IR (ν_{max}): 2098 (C≡C) cm^{-1}.

TLC R$_f$: 0.32 (9:1 PE (40 – 60)/EtOAc, 254 nm + $KMnO_4$).

1,4-bis(5,6-dimethyl-1H-benzo[d]imidazol-1-yl)buta-1,3-diyne **15**

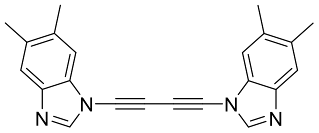

To a 50 mL round bottom flask charged with a magnetic stir bar was subsequently added 1-ethynyl-5,6-dimethyl-*1H*-benzo[*d*]imidazole-1,3 (340 mg, 170.22 g·mol^{-1}, 2.00 mmol, 1.00 equiv) and DCM (25.0 mL). Air was allowed to bubble through the solution for 15 minutes before the dropwise addition of a mixture of TMEDA (13.9 mg, 0.78 g·mL^{-1}, 18 µL, 116.24 g·mol^{-1}, 120 µmol, 6.00 mol%) and copper chloride (16.1 mg, 134.45 g·mol^{-1}, 120 µmol, 6.00 mol%) in a small amount of DCM. Again, air was bubbled through the solution for 10 minutes and the reaction mixture

was allowed to stir at ambient temperature for 24 h. The crude reaction mixture was diluted with DCM (50.0 mL) and transferred to a separatory funnel. The organic layer was washed with sat. EDTA (2 × 50.0 mL) which was extracted with more DCM (2 × 50.0 mL). The organic layers were combined, washed with brine, and dried over Na_2SO_4. The solvent was removed *in vacuo* to yield the crude as brown solid that was purified by flash chromatography (20.0 g silica gel: 97:3 DCM/MeOH) to give the desired 1,4-bis(5,6-dimethyl-*1H*-benzo[*d*]imidazol-1-yl)buta-1,3-diyne as an off white solid (136 mg, 338.41 g·mol^{-1}, 400 μmol, 40%)

^{1}H NMR: (500 MHz, $CDCl_3$) δ 8.05 (s, 2H, Ar*H*), 7.60 (s, 2H, Ar*H*), 7.43 (s, 2H, Ar*H*), 2.42 (s, 6H, C*H*3), 2.40 (s, 6H, C*H*3).

^{1}H NMR: (400 MHz, CD_3CN) δ 8.16 (s, 2H, Ar*H*), 7.55 (s, 2H, Ar*H*), 7.48 (s, 2H, Ar*H*), 2.42 (s, 6H, C*H*3), 2.38 (s, 6H, C*H*3).

^{13}C NMR: (126 MHz, $CDCl_3$) δ 143.29 (2C, Ar*C*H), 140.41 (2C, Ar*C*), 135.09 (2C, Ar*C*), 134.01 (2C, Ar*C*), 133.06 (2C, Ar*C*) 121.30 (2C, Ar*C*H) 111.46 (2C, Ar*C*H), 70.42 (2C, N-*C*≡C), 59.93 (2C, N-C≡*C*), 20.66 (2C, *C*H3), 20.42 (2C, *C*H3).

LC-MS: (17-minute, high pH) t_R = 9.5 min, *m/z* 339.4 [M+H]$^+$.

HRMS (ESI): $C_{22}H_{19}N_4$ [M+H]$^+$ calculated 339.1604, found 339.1598.

TLC R_f: 0.36 (1:1 PE (40 – 60)/EtOAc, $KMnO_4$).

N-phenylhept-6-ynamide **S1**

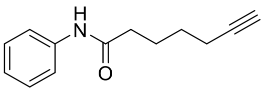

Hept-6-ynoic acid (200 mg, 126.15 g·mol^{-1}, 1.58 mmol, 1.00 equiv), aniline (162 mg, 93.13 g·mol^{-1}, 1.74 mmol, 1.10 equiv) and 4-dimethylaminopyridine (20 mg, 122.17 g·mol^{-1}, 0.16 mmol, 10 mol%) were dissolved in DCM (10 mL) and stirred at ambient temperature. N, N′-Dicyclohexylcarbodiimide (326 mg, 206.33 g·mol^{-1},1.58 mmol, 1.00 equiv) was dissolved in DCM (10 mL) and this solution was slowly added to the stirred solution. After 4 h the suspension was cooled in an ice bath and filtered. The filtrate was collected and washed subsequently with a 5 % wt aqueous solution of HCl (2 × 20 mL), a saturated solution of $NaHCO_3$ (20 mL), brine (20 mL) and dried over anhydrous Na_2SO_4. The solvent was removed *in vacuo* and the residue purified by column chromatography (0 – 20% EtOAc/PE (40 – 60) to give the desired compound as a white solid (292 mg, 201.27 g·mol^{-1}, 1.45 mmol, 92%).

^{1}H NMR (500 MHz, $CDCl_3$) δ 7.51 (d, *J* = 7.9 Hz, 2H), 7.36 – 7.27 (m, 3H), 7.10 (t, *J* = 7.4 Hz, 1H), 2.39 (t, *J* = 7.5 Hz, 2H), 2.28 – 2.20 (m, 2H), 1.97 (t, *J* = 2.6 Hz, 1H), 1.91 – 1.80 (m, 2H), 1.67 – 1.57 (m, 2H).

^{13}C NMR (126 MHz, $CDCl_3$) δ 170.9, 137.9, 129.0, 124.3, 119.8, 84.0, 68.7, 37.1, 27.8, 24.6, 18.2.

IR (ν_{max}): 3271 (sp C-H), 2951 (C-H), 2163 (C≡C), 1657 (C=O) cm^{-1}.

Experimental values in accordance with literature values.[6]

tert-Butyl [^{15}N] carbamate **S3**

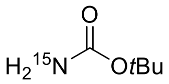

To a stirring solution of di-*tert*-butyl dicarbonate (923 mg, 218.25 g·mol^{-1}, 2.20 mmol, 1.00 equiv) in MeOH (15.0 mL) in a 50 mL round bottom flask was added [^{15}N] ammonium hydroxide solution (3.00 N, 1.60 mL, 4.70 mmol, 1.10 equiv). The reaction mixture was stirred at ambient temperature for 15 h. Upon completion of the reaction, the mixture was diluted with water and extracted with Et_2O (3 × 25.0 mL). The organic layers were combined, washed with brine, and dried over Na_2SO_4. The solvent was removed *in vacuo* yielding *tert*-butyl carbamate as a white solid (107.8 mg, 118.14 g mol^{-1}, 900 µmol, 98%).

^{1}H NMR: ($CDCl_3$, 400 MHz) δ 4.41 (d, 2H, $J_{H\text{-}N}$ = 88.6 Hz, NH_2), 1.45 (s, 9H, (CH_3)$_3$).

^{13}C{^{1}H} NMR: ($CDCl_3$,101 MHz) δ 156.47 (*C*=O), 79.83 (*C*(CH_3)$_3$), 28.38 (C(*C*H_3)$_3$).

^{15}N NMR: ($CDCl_3$,41 MHz) δ 72.0 (1N, *N*H_2).

HRMS (ESI) m/z: $C_5H_{12}O_2{}^{15}N$ $[M+H]^+$ calculated 119.0833, found 119.0832.

IR (ν_{max}): 3429 (N-H), 3117 (ar. C-H), 2923 (C-H), 1619 (C=O) cm^{-1}.

TLC R_f: 0.6 (9:1 DCM/MeOH).

tert-Butyl (3,4-dimethylphenyl) [^{15}N] carbamate **S4**

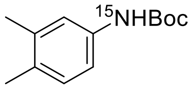

o a clean and dry microwave vial (5.00 mL) was added subsequently $Pd(OAc)_2$ (3.64 mg, 224.51 g·mol^{-1}, 16.2 µmol, 3.00 mol%), Xphos (23.2 mg, 476.72 g·mol^{-1}, 50.0 µmol, 9.00 mol%), Cs_2CO_3 (246 mg, 325.82 g·mol^{-1}, 800 µmol, 1.40 equiv), *tert*-butlylcarbamate-^{15}N (76.0 mg, 118.14 g·mol^{-1}, 650 µmol, 1.20 equiv), 4-bromo-1,2-dimethylbenzene (73.0 µL, 100 mg, 184.06 g·mol^{-1}, 540 µmol, 1.00 equiv)

and anhydrous 1,4-dioxane (4.00 mL). The flask was purged with argon thrice and allowed to reflux at 100 °C overnight. After the completion of the reaction, the mixture was allowed to cool down to room temperature and poured in H_2O. The H_2O was extracted with Et_2O (3 x 5.00 mL). The organic layers were combined, washed with brine, and dried over Na_2SO_4. The solvent was removed *in vacuo* and the remaining crude purified by flash chromatography (15.0 g silica gel, eluent 9:1 PE (40 – 60)/EtOAc) to yield *tert*-butyl (3,4-dimethylphenyl)[^{15}N]carbamate as a white solid (104 mg, 222.29 g·mol^{-1}, 0.50 mmol, 87%).

^{1}H NMR: ($CDCl_3$, 400 MHz) δ 7.19 (s, 1H, C*H*), 7.03 (m, 2H, C*H*), 6.34 (d, 1H, J_{H-N} = 89.8 Hz, N*H*), 2.23 (s, 3H, CH_3), 2.20 (s, 3H, CH_3), 1.51 (s, 9H, (CH_3)$_3$).

^{13}C{^{1}H} NMR: ($CDCl_3$,101 MHz) δ 137.34 (NH-*C*=O), 136.21 (1C, Ar*C*), 136.05 (1C, Ar*C*), 131.41 (1C, Ar*C*), 130.07 (1C, Ar*C*), 120.15 (1C, Ar*C*), 116.25 (1C, Ar*C*), 80.38 (1C, Ar*C*), 28.53 (3C, *C*H_3), 20.05 (*C*H_3), 19.19 (*C*H_3).

^{15}N NMR: ($CDCl_3$,41 MHz) δ 103.44 (1N, *N*H).

HRMS (ESI) m/z: $C_{13}H_{12}O_2{}^{15}N$ [M+H]$^+$ calculated 223.1459, found 223.1442.

IR (ν_{max}): 3360 (N-H), 1606 (C=O), 1550 (CH_3) cm^{-1}.

TLC R$_f$: 0.63 (PE (40 – 60)/EtOAc).

3,4-dimethylbenzenaminium-^{15}N 2,2,2-trifluoroacetate **S5**

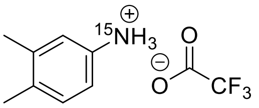

To a 50 mL round bottom flask charged with a magnetic stir bar was added subsequently *tert*-butyl (3,4-dimethylphenyl)carbamate-^{15}N (200 mg, 222.29 g·mol^{-1}, 900 µmol, 1.00 equiv), 10.0 mL DCM and 2.00 mL TFA. The full consumption of the starting material was confirmed by TLC after 30 minutes (4:1 PE (40 – 60)/EtOAc, 254 nm, $Rf_{carbamate}$ = 0.69). The solvent was evaporated *in vacuo*. The residual oil was co-evaporated with copious amounts of DCM until an off-white solid formed (trifluoroacetic acid salt, 202 mg, 236.20 g·mol^{-1}, 860 µmol, 95%).

^{1}H NMR: (400 MHz, DMSO-d_6) δ 9.13 (s, 3H, NH_3), 7.16 (d, *J* = 7.9 Hz, 1H, C*H*), 6.96 (d, *J* = 7.9 Hz, 1H, C*H*), 6.93 (d, *J* = 7.9 Hz, 1H, C*H*), 2.22 (s, 3H, CH_3), 2.19 (s, 3H, CH_3).

^{15}N NMR: (41 MHz, DMSO-d_6) δ 50.66.

TLC Rf : 0.25 (4:1 PE (40 – 60)/EtOAc).

4,5-dimethyl-2-(nitro)aniline-$^{15}N_2$ **S6**

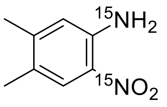

In a 25.0 mL round bottom flask charged with a magnetic stir bar was dissolved 3,4-dimethylbenzenaminium-^{15}N 2,2,2-trifluoroacetate (433 mg, 236.20 g·mol^{-1}, 1.80 mmol, 1.00 equiv) in H_2SO_4 (7.00 mL). The mixture was stirred, and the flask chilled to -10 °C using a 1:1 ice/acetone bath which was kept at a constant temperature. A mixture of sodium nitrate-^{15}N (159 mg, 85.99 g·mol^{-1}, 1.90 mmol, 1.00 equiv) dissolved in H_2SO_4 (4.00 mL) was added to the chilled flask dropwise over 15 minutes. The ice/acetone bath was kept in place and allowed to reach room temperature. Two hours after the addition of sodium nitrate-^{15}N, full consumption of the starting material was confirmed by TLC (7:3 PE (40 – 60)/EtOAc, $Rf_{4,5\text{-dimethyl-2-nitroaniline-15N}}$ = 0.34, 254 nm). The reaction mixture was transferred to a 500 mL separatory funnel that was charged with a saturated solution of $NaHCO_3$ and ice. The aqueous layer was extracted with EtOAc (4 × 50.0 mL). The organic layers were combined and washed with brine, dried over Na_2SO_4 and the solvent was evaporated *in vacuo*. The resulting crude product was purified using flash chromatography (30.0 g silica, 8:2 PE (40 – 60)/EtOAc), crude loaded in DCM, to yield the desired product as a bright orange solid (235 mg, 168.17 g mol^{-1}, 1.40 mmol, 76%).

^{1}H NMR: (400 MHz, DMSO-*d$_6$*) δ 7.72 (s, 1H, C*H*), 7.21 (d, *J* = 90.6 Hz, 2H, N*H$_2$*), 6.79 (s, 1H, C*H*), 2.17 (s, 3H, C*H$_3$*), 2.12 (s, 3H, C*H$_3$*).

^{13}C NMR: (101 MHz, DMSO-*d$_6$*) δ 146.37 (1C, Ar*C*), 144.63 (1C, Ar*C*), 124.45 (Ar*C*H), 118.98 (1C, Ar*C*H), 101.17 (1C, Ar*C*), 99.41 (1C, Ar*C*), 19.77 (1C, *C*H$_3$), 18.13 (1C, *C*H$_3$).

^{15}N NMR: (41 MHz, dimethyl sulfoxide-*d$_6$*) δ 76.13 (1N, *N*H).

HRMS (ESI): $C_8H_{11}O_2{}^{15}N_2$ $[M+H]^+$ calculated 169.0756, found 169.0754.

IR (ν_{max}): 3369 (N-H), 1606 (C=O), 1556 (N-O) cm^{-1}.

TLC Rf : 0.44 (7:3 PE (40 – 60)/EtOAc, bright yellow)

4,5-dimethylbenzene-1,2-diamine-$^{15}N_2$ **S7**

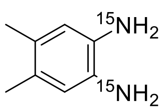

To a 100 mL round bottom flask charged with a magnetic stir bar was added 4,5-dimethyl-2-(nitro)aniline-$^{15}N_2$ (420 mg, 168.17 g·mol^{-1}, 2.50 mmol, 1.00 equiv), Pd/C (10.0% Pd loading, 265 mg, 106.42 g·mol^{-1}, 250 µmol, 10.0 mol%) and MeOH (50.0 mL). The mixture was degassed twice before the addition of two H_2-balloons. The mixture was stirred for 13 h at ambient temperature. TLC conformed full

consumption of the starting material (4:1 EtOAc/PE (40 – 60), $Rf_{4,5\text{-dimethyl-2-(nitro)aniline-15N2}}$ = 0.87, yellow). The Pd/C was filtered off over a nylon filter, and the filtrate was evaporated to yield 4,5-dimethylbenzene-1,2-diamine-$^{15}N_2$ as a brown solid (299 mg, 138.18 g mol^{-1}, 2.20 mmol, 87%).

^{1}H NMR: (400 MHz, DMSO-*d_6*) δ 6.29 (s, 2H, C*H*), 4.12 (s, 4H, N*H_2*), 1.97 (s, 6H, C*H_3*).

^{13}C NMR: (101 MHz, DMSO-*d_6*) δ 132.62 (2C, Ar*C*NH_2), 123.76 (2C, Ar*C*), 116.57 (2C, Ar*C*H), 18.59 (2C, Ar-*C*H_3).

^{15}N NMR: (41 MHz, DMSO-*d_6*) δ 48.88 (2N, *N*H_2).

LC-MS: (17-minute, low pH) t_R = 5.4 min, *m/z* 139.2 $[M+H]^+$.

HRMS (ESI): $C_8H_{13}{}^{15}N_2$ $[M+H]^+$ calculated 139.1014, found 139.1022.

IR (ν_{max}): 3350 (N-H) cm^{-1}.

TLC Rf : 0.37 (4:1 PE (40 – 60)/EtOAc, 254 nm)

5,6-dimethyl-1H-benzo[d]imidazole-1,3-$^{15}N_2$ **S8**

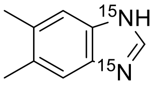

To a 100 mL round bottom flask charged with a magnetic stir bar was added 4,5-dimethylbenzene-1,2-diamine-$^{15}N_2$ (380 mg, 138.18 g·mol^{-1}, 2.80 mmol, 1.00 equiv) and formic acid (97%, 25.0 mL, neat) giving a red coloured mixture. An air-cooled reflux condenser was fitted, and the mixture was stirred at 500 RPM and heated to reflux temperature (100 °C) for one hour. TLC confirmed the full consumption of the starting material (EtOAc Et_3N neutralised sample, $Rf_{5,6\text{-dimethyl-1H-benzo[d]imidazole-15N}}$ = 0.1, 254 nm). The formic acid was evaporated (rotary evaporation: ~150 mbar, 60 °C). The remaining residue (red/brown, liquid at 60 °C, solid at ambient temperature) was dissolved in 50.0 mL 200 mM HCl solution that remained red/brown and was washed with $CHCl_3$ (4 × 20.0 mL). The aqueous solution was adjusted to pH 10.0 using NH_4OH solution, whereupon the aqueous layer cleared up and a light brown/yellow, gooey solid precipitated that was extracted with $CHCl_3$ (4 × 30.0 mL). The organic extract was dried over Na_2SO_4 and the solvent removed *in vacuo*. Drying under vacuum overnight yielded the desired pure 5,6-dimethyl-1H-benzo[d]imidazole-$^{15}N_2$ (407 mg, 148.18 g·mol^{-1}, 2.80 mmol, quantitative).

^{1}H NMR: (400 MHz, DMSO) δ 8.14 (s, 1H, N^1-*H*), 8.04 (t, *J* = 10.5 Hz, 1H, Im-*H*), 7.34 (s, 2H, C*H*), 2.30 (s, 6H, C*H_3*).

^{13}C NMR: (101 MHz, DMSO) δ 163.03 (2C, Ar*C*), 140.87 (t, *J* = 5.1 Hz, 1C, Im*C*-H), 130.00 (2C, Ar*C*), 115.30 (2C, Ar*C*-H), 19.94 (2C, *C*H$_3$).

LC-MS: (17-minute, low pH) t_R = 5.5 min, *m/z* 149.2 [M+H]$^+$, 147.2 [M-H]$^-$.

HRMS (ESI): $C_9H_{11}{}^{15}N_2$ [M+H]$^+$ calculated 149.0857, found 149.0857.

IR (ν_{max}): 2918 (N-H) cm^{-1}.

TLC Rf: 0.32 (9:1 DCM/MeOH, 254 nm).

(bromoethynyl)Triisopropylsilane **S9**

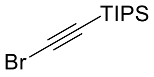

A mixture of N-bromosuccinimide (7.60 g, 177.99 g·mol^{-1}, 45.8 mmol, 1.10 equiv), ethynyltriisopropylsilane (7.56 g, 0.813 g·mL^{-1}, 9.30 mL, 182.38 g·mol^{-1}, 41.6 mmol, 1.00 equiv) and $AgNO_3$ (8.00 mg, 169.87 g·mol^{-1}, 4.20 mmol, 10.0 mol%) in acetone (37.0 mL) was covered in foil and stirred overnight at ambient temperature. The reaction mixture was filtered through a Celite© plug in a fritted funnel using PE (40 – 60) and concentrated in vacuum to give a clear oil with white solid precipitation. The residue was suspended in PE (40 – 60) and filtered through a silica plug in a fritted funnel and subsequently eluted with PE (40 – 60). The solvent was removed *in vacuo* and the desired product was obtained as a clear liquid (10.0 g, 261.28 g·mol^{-1}, 38.3 mmol, 92%, 1.04 g mL^{-1}).

^{1}H NMR: (500 MHz, $CDCl_3$): δ 1.43 – 0.83 (s, 21H, Si(C*H*C*H*$_3$)$_3$).

^{13}C NMR: (126 MHz, $CDCl_3$) δ 83.59 (1C, Si-*C*≡C-Br), 61.90 (1C, Si-C≡*C*-Br), 18.65 (6C, Si(CH*C*H$_3$)$_3$), 11.49 (3C, Si(*C*HCH$_3$)$_3$).

TLC R_f: 0.79 (PE (40 – 60), $KMnO_4$).

GC-MS: $C_{11}H_{21}{}^{79}BrSi$ [M]$^+$ calculated 260.1, found 260.1

$C_{11}H_{21}{}^{81}BrSi$ [M]$^+$ calculated 262.1, found 262.0

Experimental values in accordance with literature values.[5]

(bromoethynyl-1,2-$^{13}C_2$)triisopropylsilane **S9-^{13}C$_2$**

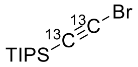

To a small vial charged with a stir bar was added N-bromosuccinimide (1.03 g, 177.98 g·mol^{-1}, 5.80 mmol, 1.10 equiv), (ethynyl-$^{13}C_2$)triisopropylsilane (1.00 g, 184.37 g·mol^{-1}, 5.42 mmol, 1.00 equiv) and silver nitrate (102 mg, 169.87 g·mol^{-1}, 600 µmol,

10.0 mol%) in acetone (2.70 ml). The vial was covered in foil and stirred overnight at ambient temperature. The reaction mixture was filtered through a celite plug in a fritted funnel using PE (40 – 60) and concentrated *in vacuo* to give a clear oil with white solid precipitation. The residue was suspended in PE (40 – 60) ether again and filtered through a silica plug in a fritted funnel and eluted with PE (40 – 60). The solvent was removed in vacuum and the desired product was obtained as a clear oil (1.40 g, 263.26 g·mol^{-1}, 5.32 mmol, 98%).

^{1}H NMR: (400 MHz, $CDCl_3$) δ 1.09 – 1.06 (m, 21H, (C*H*)$_3$, (C*H*$_3$)$_6$).

^{13}C NMR: (101 MHz, $CDCl_3$) δ 83.64 (d, *J* = 141.2 Hz, Br-*C*≡C-TIPS), 61.82 (d, *J* = 141.6 Hz, Br-C≡*C*-TIPS), 18.64 (Si(CH)$_3$(*C*H$_3$)$_6$), 11.44 (d, J = 4.6 Hz, (Si(*C*H)$_3$(CH$_3$)$_6$)).

IR (ν_{max}): 2036 (C≡C) cm^{-1}.

GC-MS: $C_9{}^{13}C_2H_{21}{}^{79}BrSi$ $[M]^+$ calculated 262.1, found 262.0

$C_9{}^{13}C_2H_{21}{}^{81}BrSi$ $[M]^+$ calculated 264.1, found 264.1

1-((triisopropylsilyl)ethynyl)-1H-indole **S10**

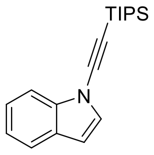

To a clean and dry 20 mL Biotage® microwave vial charged with a magnetic stir bar was subsequently added indole (468 mg, 117.15 g·mol^{-1}, 4.00 mmol, 1.00 equiv), caesium carbonate (1.56 g, 325.82 g·mol^{-1}, 4.80 mmol, 1.20 equiv), copper iodide (38.0 mg, 190.45 g·mol^{-1}, 200 µmol, 5.00 mol%), (bromoethynyl)triisopropylsilane (2.10 g, 267.28 g·mol^{-1}, 8.00 mmol, 2.00 equiv) and PEG400 (160 mg, 0.4 mmol, 10.0 mol%). The vial was capped and purged with argon thrice and 1,4-dioxane (11.5 mL, anhydrous) was added. The reaction mixture was heated to 160 °C for 16 hours. The reaction was allowed to cool down to ambient temperature and the resulting suspension was filtered over a Celite® plug. The Celite® was washed with EtOAc to give a yellow permeate. The solvent was removed *in vacuo* to give 2.90 g of crude product which was purified by flash column chromatography using 30 g silica and eluted with hexane, slowly, to yield the desired product as a colourless oil (690 mg, 297.52 g·mol^{-1}, 2.32 mmol, 58%).

^{1}H NMR: (400 MHz, CD_3CN) δ 7.64 (dt, J = 7.9, 1.0 Hz, 1H, Ar*H*), 7.53 (dq, J = 8.1, 0.9 Hz, 1H, Ar*H*), 7.39 – 7.30 (m, 2H, 1, Ar*H*), 7.23 (ddd, J = 8.1, 7.1, 1.0 Hz, 1H, Ar*H*), 6.61 (dd, J = 3.4, 0.9 Hz, 1H, Ar*H*), 1.17 (d, J = 2.7 Hz, 21H, Si((C*H*)$_3$(C*H*$_3$)$_6$).

^{13}C NMR: (101 MHz, CD_3CN) δ 139.04 (1C, Ar*C*), 130.12 (1C, Ar*C*H), 128.74 (1C, Ar*C*H), 124.81 (1C, Ar*C*H), 123.16 (1C, Ar*C*H), 122.27 (1C, Ar*C*H), 111.87 (1C, Ar*C*H), 106.42 (1C, Ar*C*H), 95.48 (1C, N-C≡*C*-Si), 69.54 (1C, N-*C*≡C-Si), 19.01 (6C, *C*H$_3$), 12.11 (3C, *C*H).

LC-MS: (17-minute, pH = 10.5) t_R = 7.2 min, *m/z* 298.2 $[M+H]^+$.

TLC R_f: 0.5 (hexane, 254 nm + $KMnO_4$).

Experimental values in accordance with literature values.[6]

11 References

1. Burley, G. A.; Davies, D. L.; Griffith, G. A.; Lee, M.; Singh, K., Cu-Catalyzed N-Alkynylation of Imidazoles, Benzimidazoles, Indazoles, and Pyrazoles Using PEG as Solvent Medium. *J. Org. Chem.* **2010,** *75* (3), 980-983.
2. *CrysalisPro*, Rigaku Oxford Diffraction: 2019.
3. Sheldrick, G., Crystal structure refinement with SHELXL. *Acta Crystallographica Section C* **2015,** *71* (1), 3-8.
4. Farrugia, L., WinGX and ORTEP for Windows: an update. *J. Appl. Crystallogr.* **2012,** *45* (4), 849-854.
5. Porey, S.; Zhang, X.; Bhowmick, S.; Kumar Singh, V.; Guin, S.; Paton, R. S.; Maiti, D., Alkyne Linchpin Strategy for Drug:Pharmacophore Conjugation: Experimental and Computational Realization of a Meta-Selective Inverse Sonogashira Coupling. *J. Am. Chem. Soc.* **2020,** *142* (8), 3762-3774.
6. Seath, C. P.; Burley, G. A.; Watson, A. J., Determining the Origin of Rate-Independent Chemoselectivity in CuAAC Reactions: An Alkyne-Specific Shift in Rate-Determining Step. *Angew. Chem. Int. Ed.* **2017,** *56* (12), 3314-3318.
7. Campbell-Verduyn, L.; Elsinga, P. H.; Mirfeizi, L.; Dierckx, R. A.; Feringa, B. L., Copper-free 'click': 1,3-dipolar cycloaddition of azides and arynes. *Org. Biomol. Chem.* **2008,** *6* (19), 3461-3.
8. Albertin, G.; Antoniutti, S.; Baldan, D.; Castro, J.; Garcia-Fontan, S., Preparation of benzyl azide complexes of iridium(III). *Inorganic chemistry* **2008,** *47* (2), 742-8.

12 NMR, FT-IR, and MS spectra

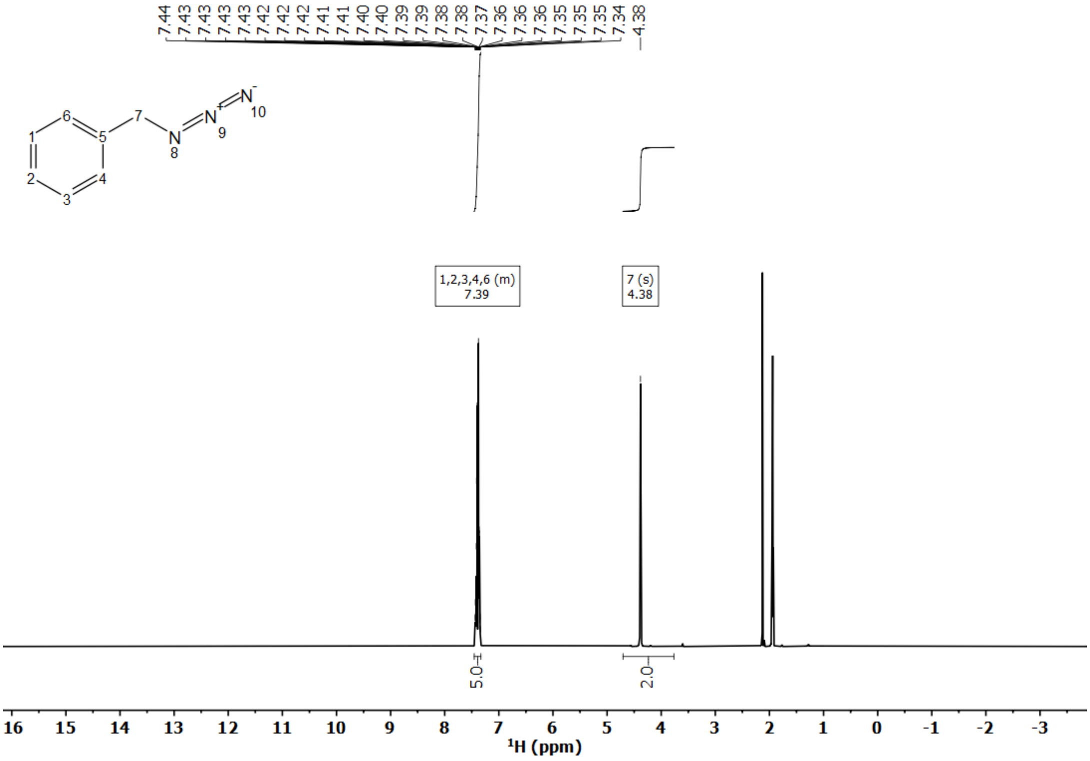

Figure S120. ^{1}H NMR spectrum of benzyl azide 2.

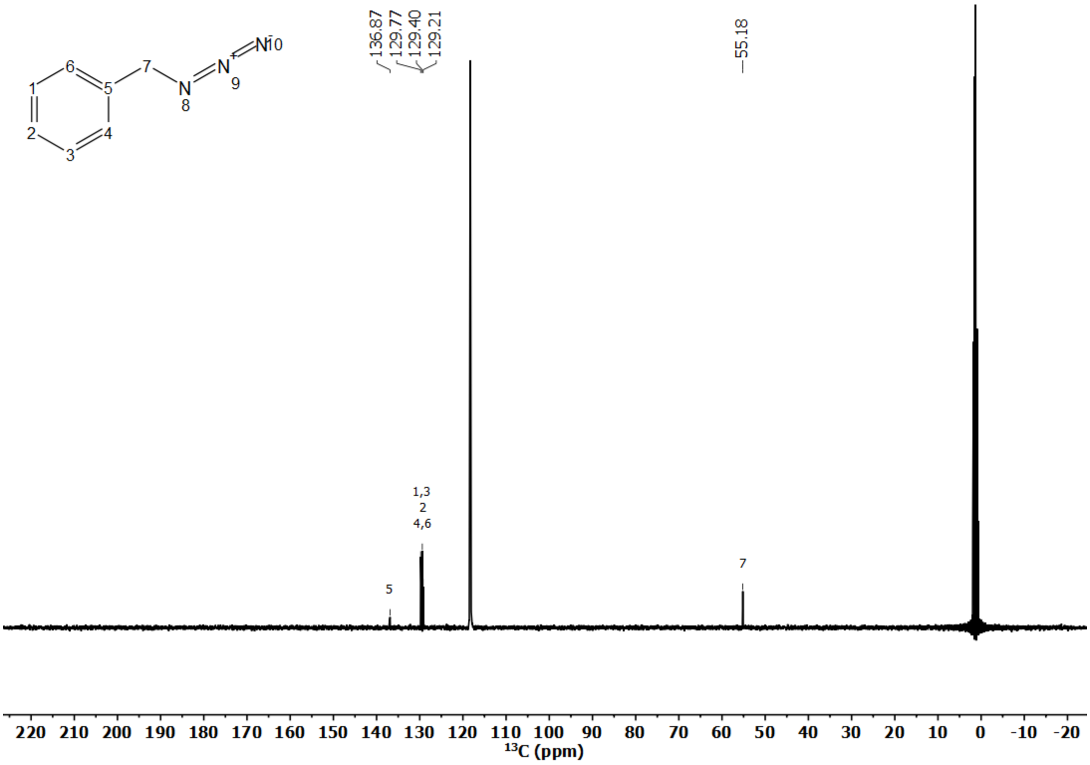

Figure S121. ^{13}C{^{1}H} NMR spectrum of benzyl azide **2**.

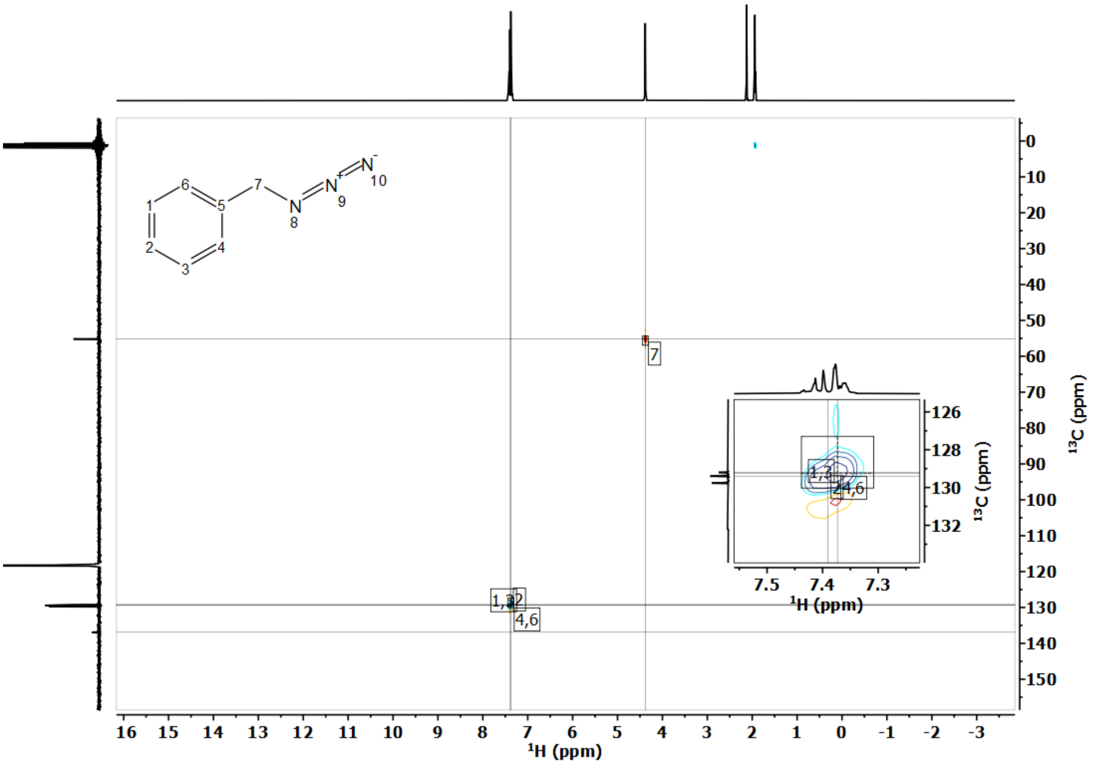

Figure S122. ^{1}H-^{13}C HSQC NMR spectrum of benzyl azide **2**.

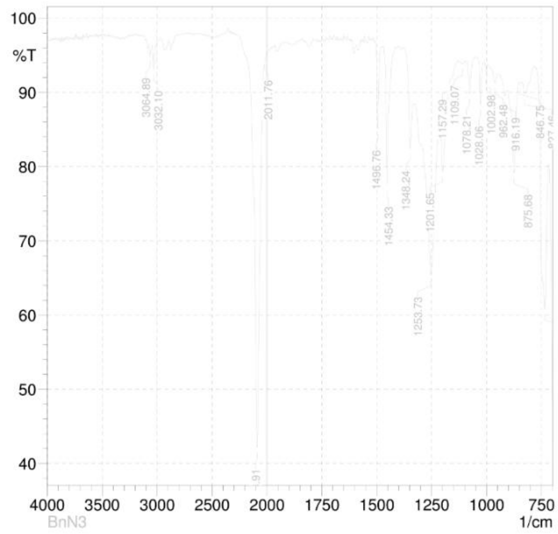

SHIMADZU

No.	Peak	Intensity	Corr. Inte	Base (H)	Base (L)	Area	Corr. Are
1	734.88	60.756	9.509	744.52	717.52	4.415	0.683
2	827.46	89.782	2.012	837.11	785.03	2.048	0.282
3	846.75	91.157	0.19	850.61	839.03	0.458	0.004
4	875.68	79.133	11.742	906.54	852.54	3.422	1.163
5	916.19	89.491	1.224	948.98	908.47	1.631	0.06
6	962.48	91.245	1.594	995.27	950.91	1.482	0.108
7	1002.98	92.535	1.323	1008.77	997.2	0.352	0.032
8	1028.06	88.343	6.039	1049.28	1010.7	1.201	0.235
9	1078.21	89.853	4.532	1093.64	1058.92	1.118	0.251
10	1109.07	93.808	0.422	1128.36	1095.57	0.878	0.032
11	1157.29	91.917	0.355	1161.15	1130.29	0.937	0.011
12	1201.65	79.357	7.575	1213.23	1161.15	3.361	0.691
13	1253.73	65.341	18.853	1294.24	1215.15	9.6	3.687
14	1348.24	82.335	9.235	1379.1	1327.03	2.448	0.627
15	1454.33	77.5	18.609	1469.76	1417.68	2.178	1.271
16	1496.76	85.094	11.423	1504.48	1479.4	0.85	0.451
17	2011.76	94.532	0.177	2013.68	2002.11	0.264	0.006
18	2088.91	42.162	51.886	2167.99	2013.68	19.888	15.769
19	3032.1	93.089	3.273	3047.53	3010.88	0.806	0.218
20	3064.89	94.852	1.554	3078.39	3049.46	0.551	0.09

Comment;
BnN3

Date/Time; 30/01/2023 14:08:31
No. of Scans; 16
Resolution; 4 [1/cm]
Apodization; Happ-Genzel

Figure S123. FT-IR spectrum of benzyl azide 2.

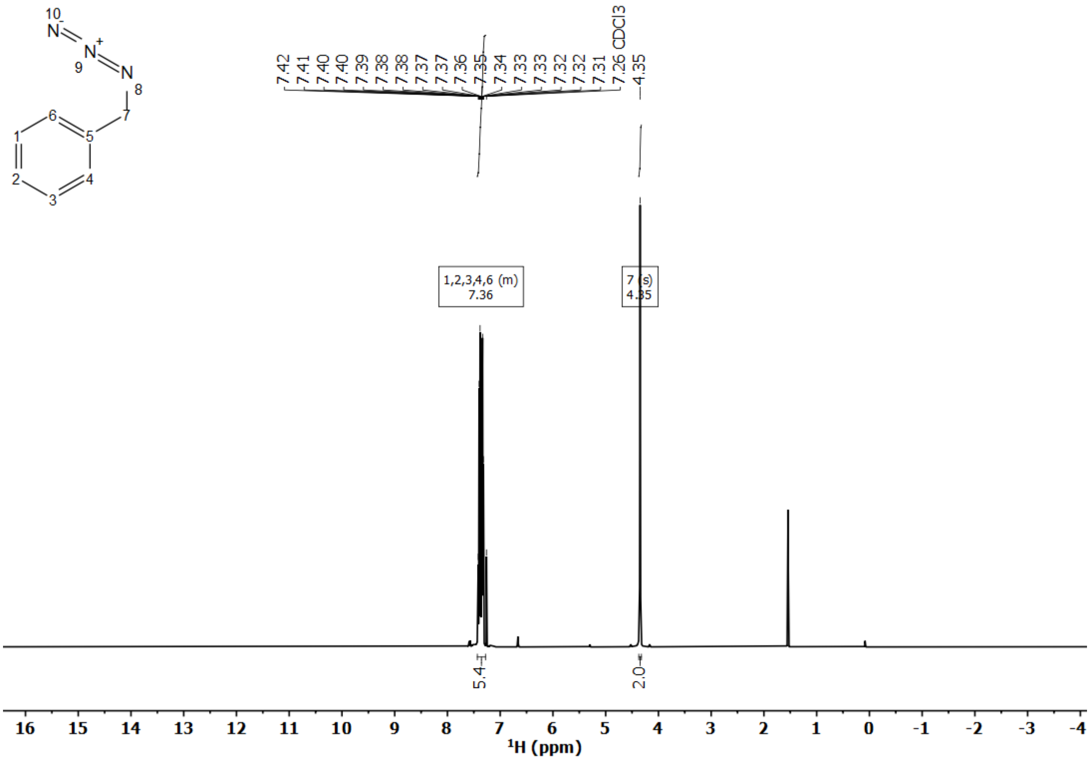

Figure S124. ^{1}H NMR spectrum of benzyl azide-^{15}N **2-^{15}N$_2$.**

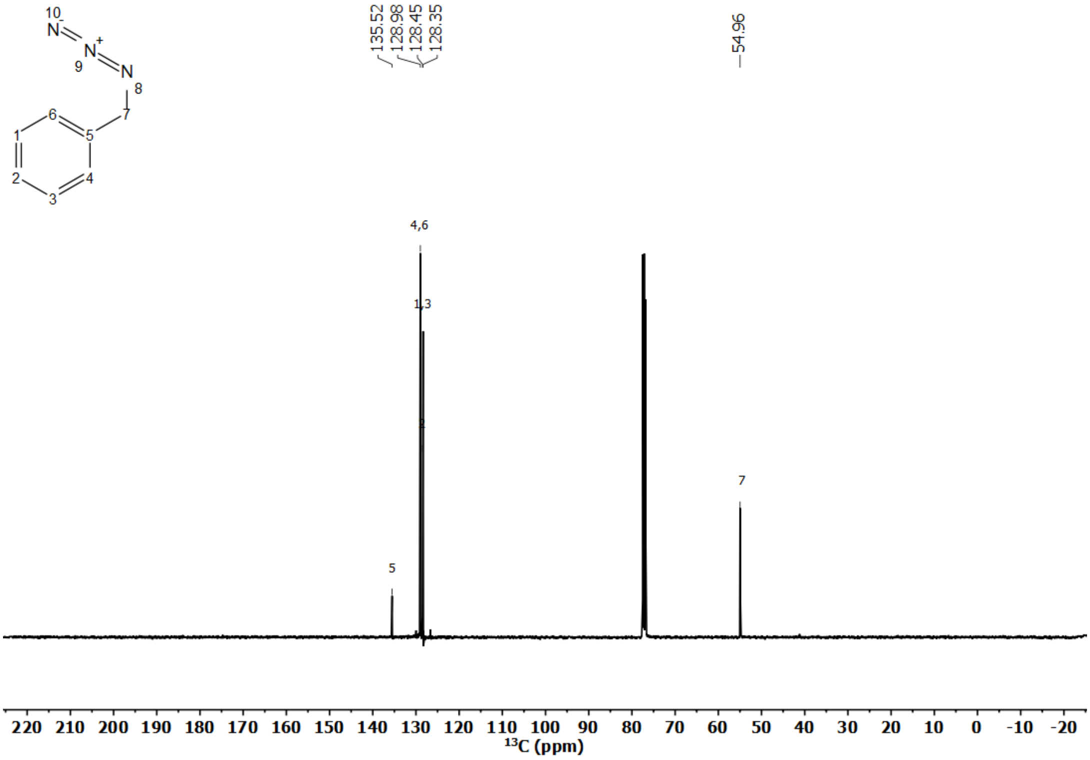

Figure S125. ^{13}C{^{1}H} NMR spectrum of benzyl azide-^{15}N **2-^{15}N$_2$.**

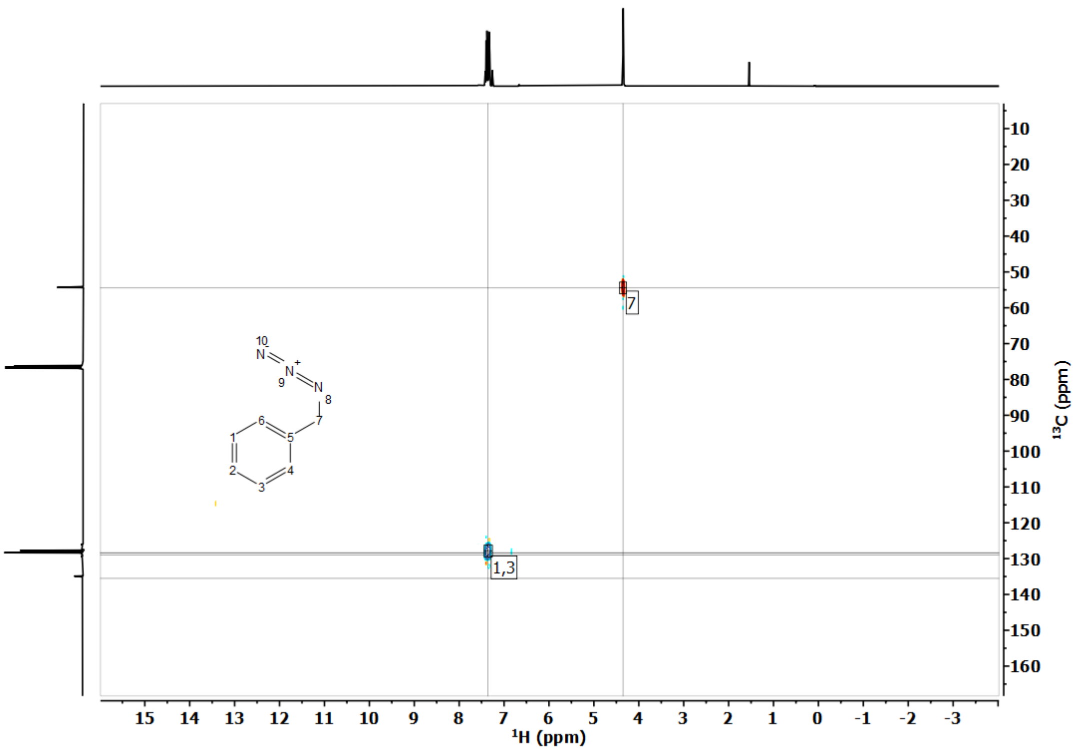

Figure S126. ^{1}H-^{13}C HSQC NMR spectrum of benzyl azide-^{15}N **2-^{15}N$_2$**.

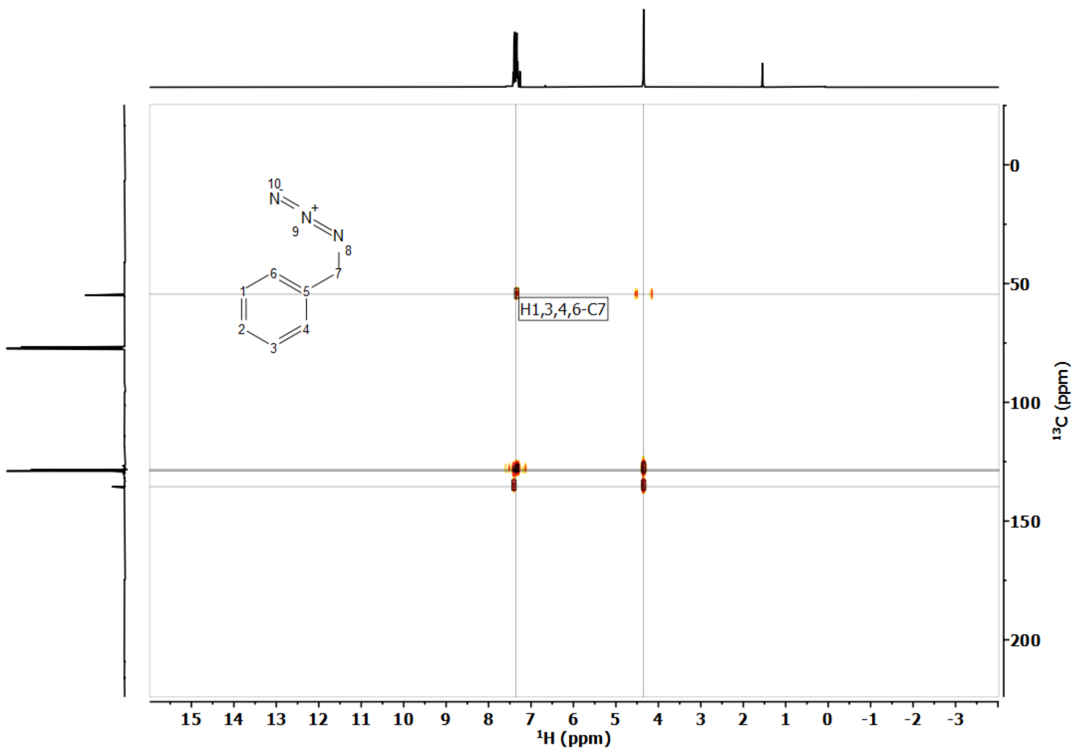

Figure S127. ^{1}H-^{13}C HMBC NMR spectrum of benzyl azide-^{15}N **2-^{15}N$_2$**.

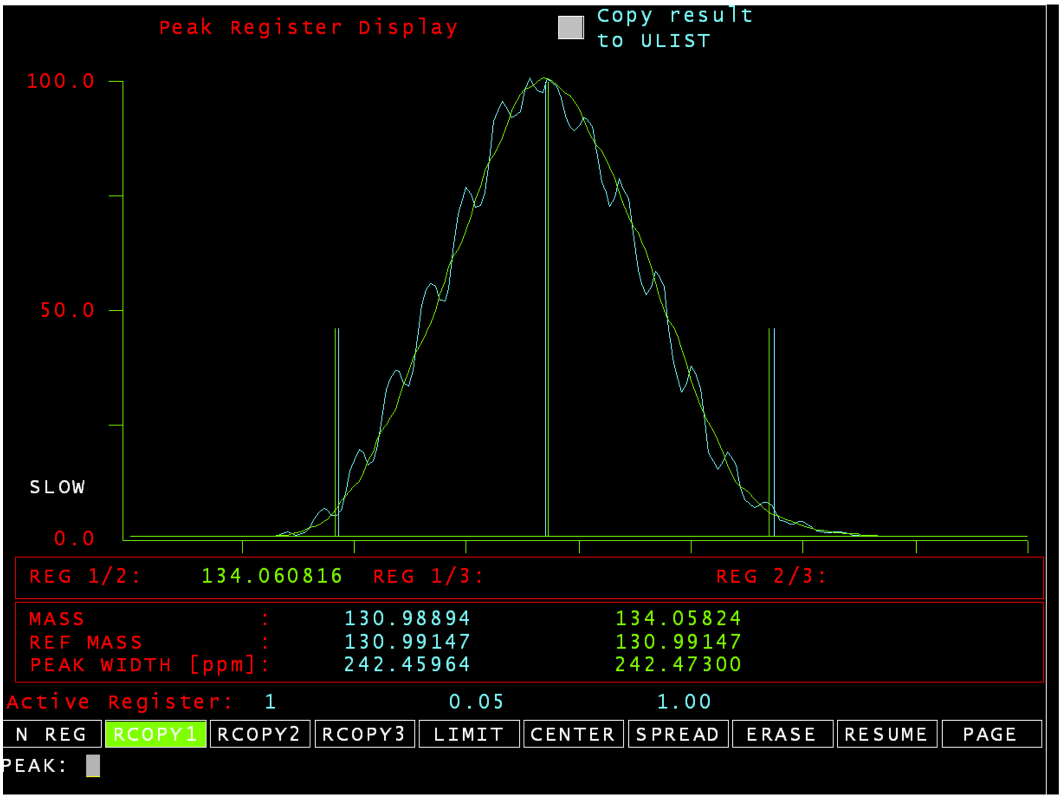

Figure S128. HRMS analysis of benzyl azide-^{15}N **2-$^{15}N_2$**.

SHIMADZU

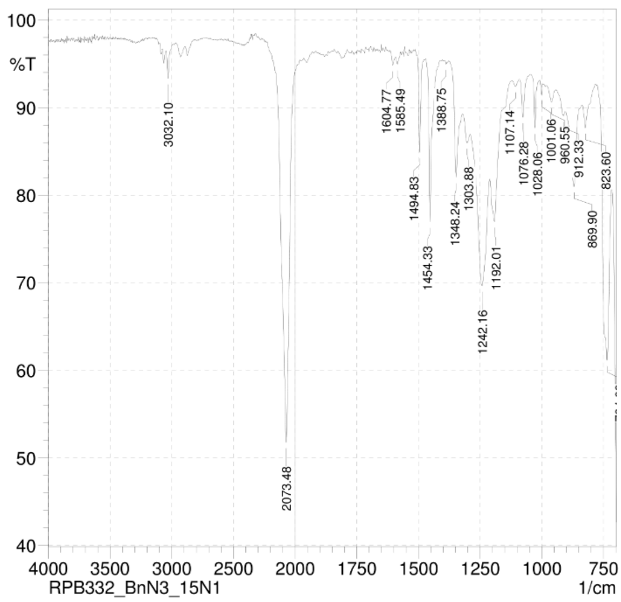

No.	Peak	Intensity	Corr. Inte	Base (H)	Base (L)	Area	Corr. Are
1	734.88	61.16	7.831	742.59	717.52	4.007	0.521
2	823.6	87.704	3.193	837.11	785.03	2.314	0.306
3	869.9	80.991	8.95	906.54	839.03	4.14	1.025
4	912.33	89.058	0.917	941.26	906.54	1.516	0.055
5	960.55	90.599	1.642	981.77	943.19	1.473	0.122
6	1001.06	91.469	1.35	1008.77	997.2	0.407	0.035
7	1028.06	87.665	5.784	1045.42	1010.7	1.26	0.236
8	1076.28	88.886	4.541	1091.71	1047.35	1.555	0.265
9	1107.14	92.422	0.715	1122.57	1093.64	0.941	0.047
10	1192.01	76.97	7.38	1211.3	1124.5	5.657	0.782
11	1242.16	69.644	14.048	1290.38	1213.23	8.541	2.794
12	1303.88	85.982	1.888	1321.24	1292.31	1.727	0.125
13	1348.24	82.029	10.048	1377.17	1323.17	2.661	0.767
14	1388.75	94.969	0.399	1392.61	1379.1	0.292	0.013
15	1454.33	76.944	18.475	1469.76	1411.89	2.478	1.295
16	1494.83	84.874	10.938	1504.48	1479.4	0.925	0.448
17	1585.49	94.897	1.019	1591.27	1573.91	0.345	0.042
18	1604.77	94.822	1.376	1614.42	1593.2	0.423	0.058
19	2073.48	51.779	42.986	2162.2	1982.82	19.989	15.808
20	3032.1	93.323	3.32	3045.6	2997.38	0.931	0.254

Comment;
RPB332_BnN3_15N1

Date/Time; 30/01/2023 14:11:53
No. of Scans; 16
Resolution; 4 [1/cm]
Apodization; Happ-Genzel

Figure S129. FT-IR spectrum of benzyl azide-^{15}N **2-$^{15}N_2$**.

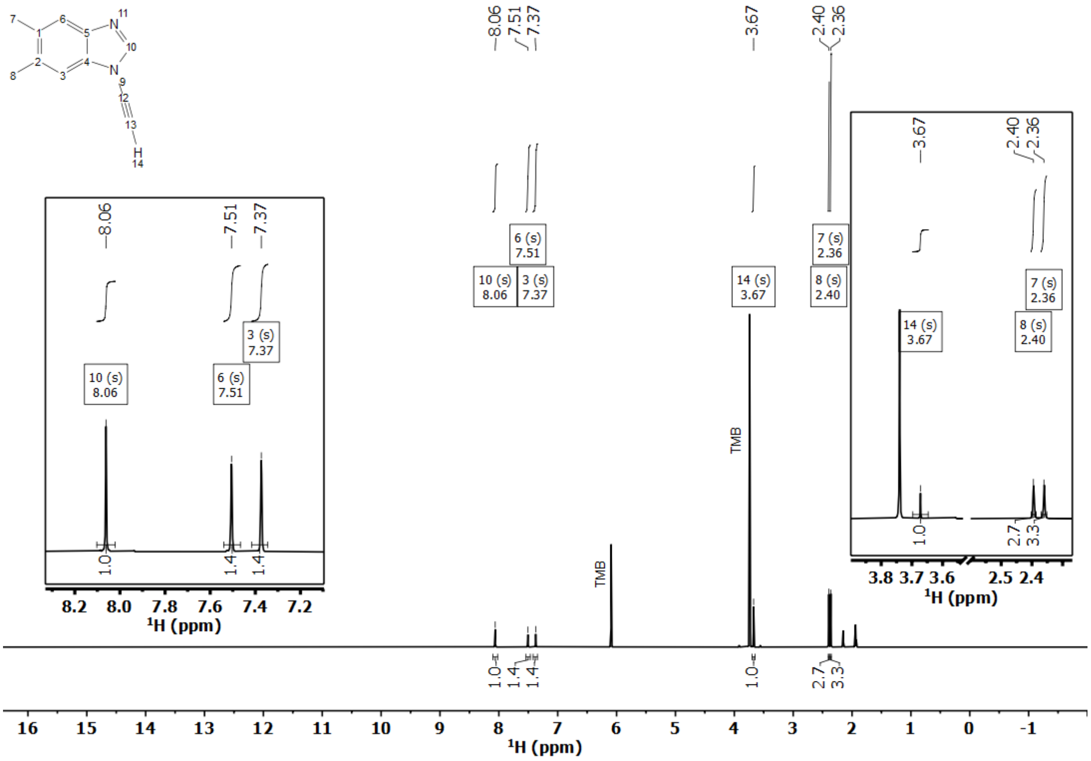

Figure S130. ^{1}H NMR spectrum of 1-ethynyl-5,6-dimethyl-1*H*-benzo[*d*]imidazole **5**.

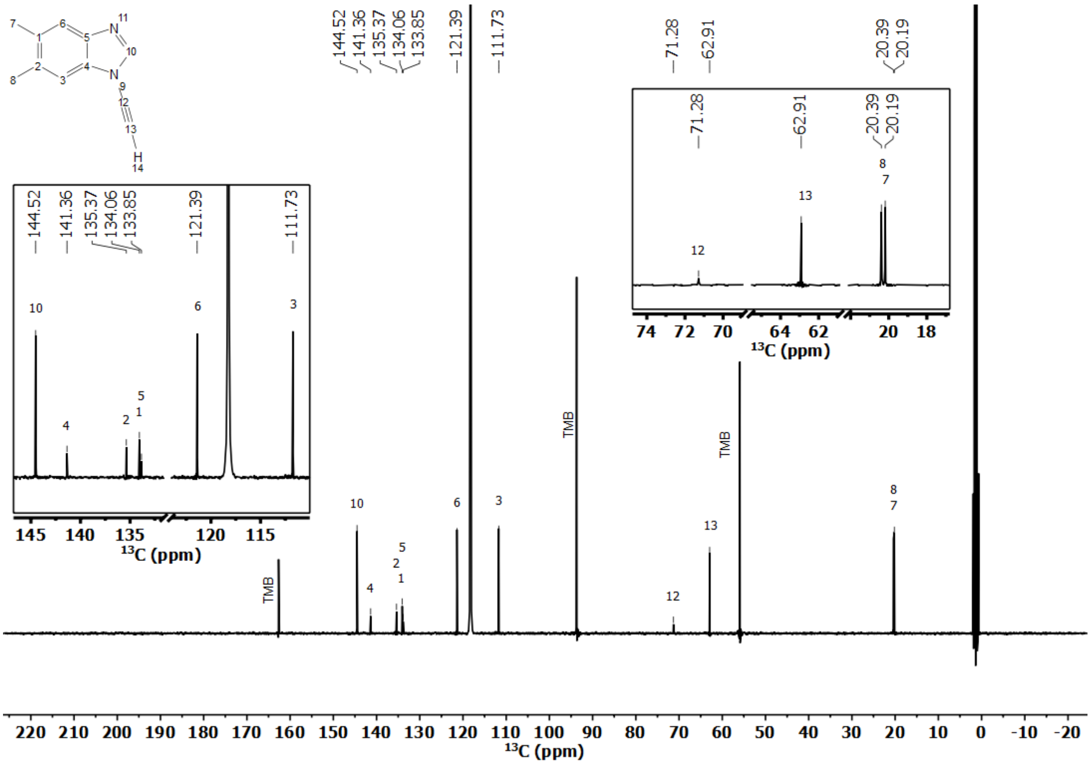

Figure S131. ^{13}C{^{1}H} NMR spectrum of 1-ethynyl-5,6-dimethyl-1*H*-benzo[*d*]imidazole **5**.

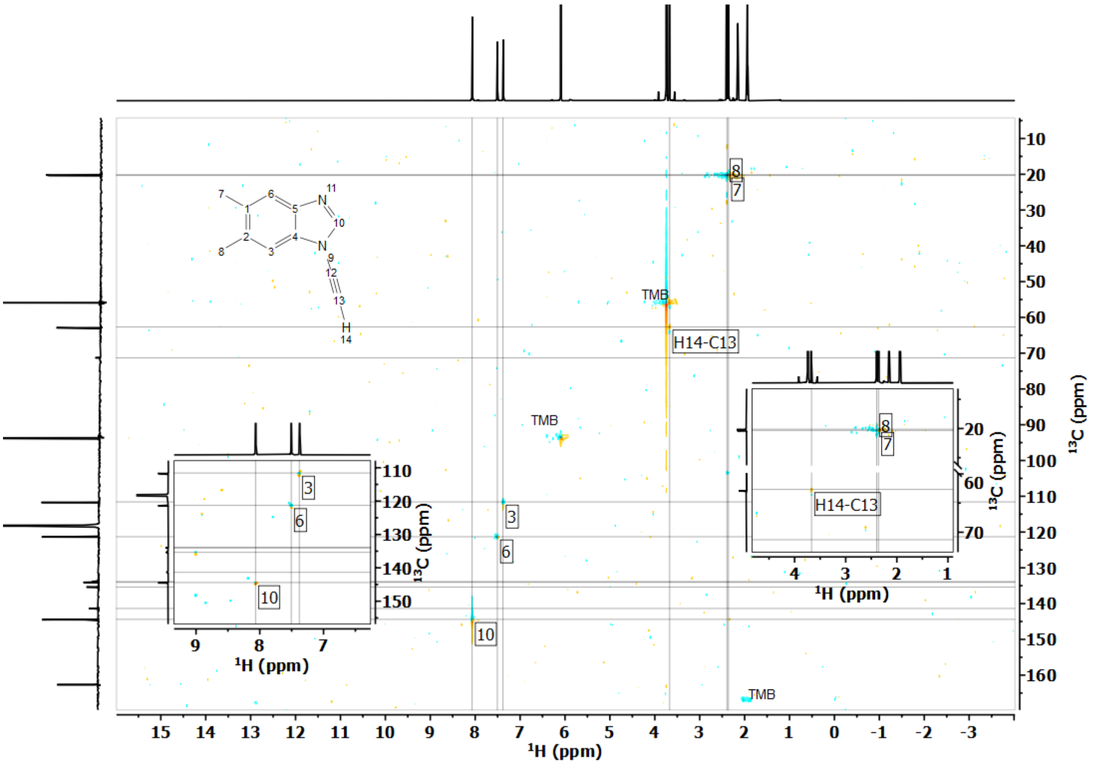

Figure S132. ^1H-^{13}C HSQC NMR spectrum of 1-ethynyl-5,6-dimethyl-1*H*-benzo[*d*]imidazole **5**.

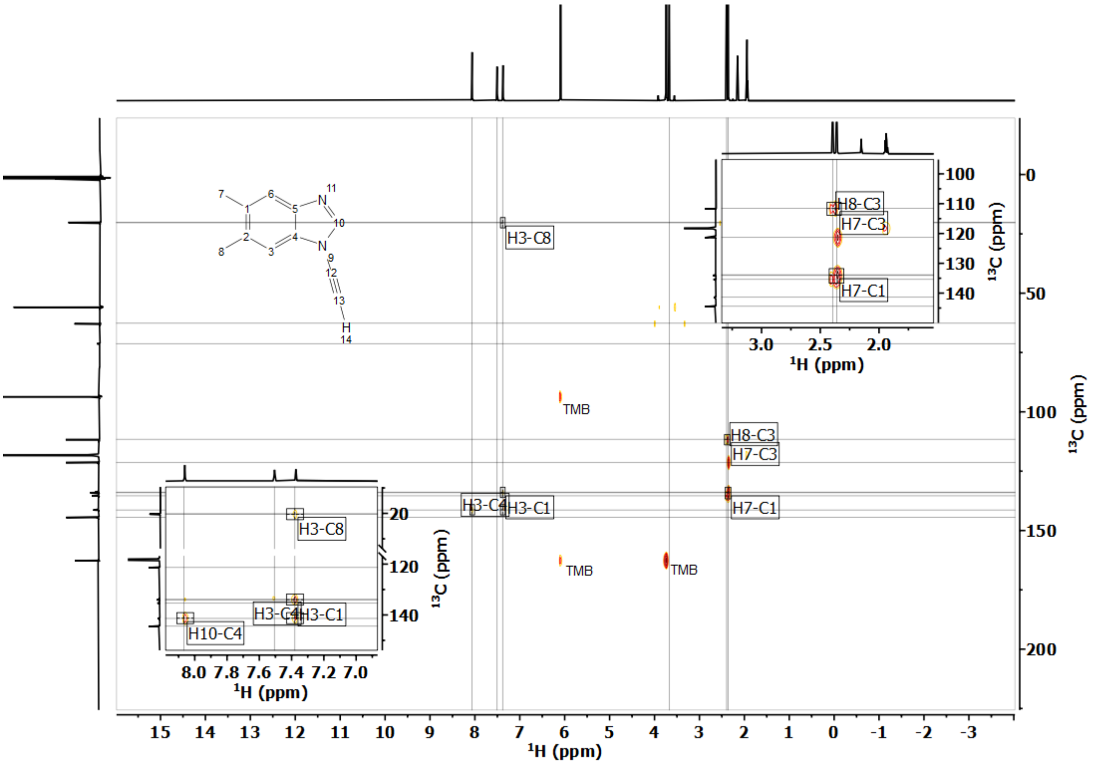

Figure S133. ^1H-^{13}C HMBC NMR spectrum of 1-ethynyl-5,6-dimethyl-1*H*-benzo[*d*]imidazole **5**.

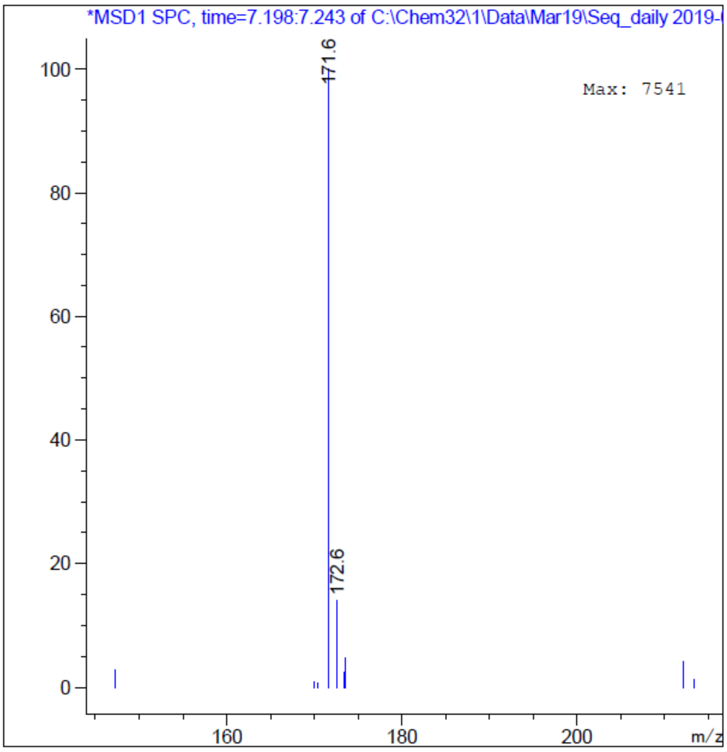

Figure S134. LC-MS analysis of 1-ethynyl-5,6-dimethyl-1*H*-benzo[*d*]imidazole **5**.

SHIMADZU

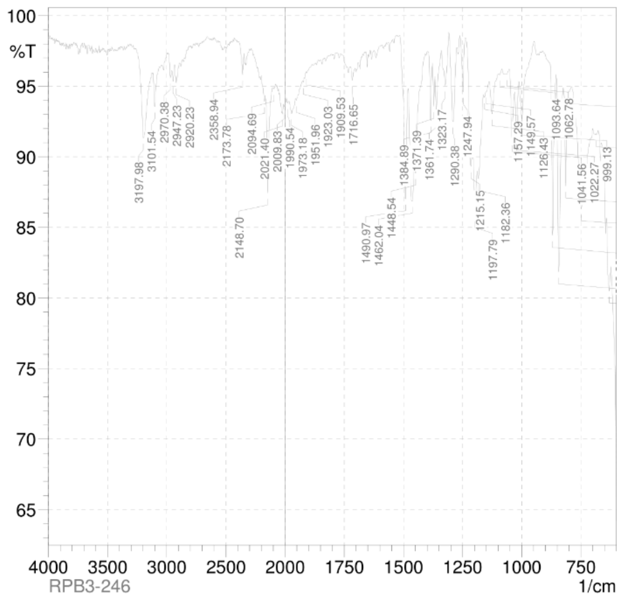

No.	Peak	Intensity	Corr. Inte	Base (H)	Base (L)	Area	Corr. Are
1	630.72	80.499	2.579	640.37	624.94	1.371	0.145
2	684.73	91.566	0.122	686.66	677.01	0.363	0.004
3	748.38	86.318	6.818	788.89	707.88	3.682	1.157
4	798.53	94.607	0.159	802.39	790.81	0.271	0.004
5	813.96	87.927	6.86	823.6	802.39	0.73	0.235
6	844.82	81.845	13.672	854.47	825.53	1.415	0.813
7	869.9	84.433	11.653	910.4	856.39	1.33	0.506
8	999.13	92.409	3.406	1010.7	948.98	1.167	0.278
9	1022.27	91.311	3.611	1029.99	1012.63	0.516	0.13
10	1041.56	91.222	4.058	1051.2	1031.92	0.578	0.165
11	1062.78	95.854	0.154	1068.56	1053.13	0.274	0.005
12	1093.64	95.806	0.168	1097.5	1083.99	0.241	0.006
13	1126.43	93.057	2.453	1138	1099.43	0.911	0.161
14	1149.57	94.613	0.116	1151.5	1139.93	0.263	0.006
15	1157.29	94.206	0.292	1161.15	1151.5	0.242	0.007
16	1182.36	88.455	1.264	1186.22	1163.08	0.945	0.08
17	1197.79	85.859	3.785	1207.44	1188.15	1.082	0.16
18	1215.15	89.326	2.512	1238.3	1209.37	0.831	0.11
19	1247.94	94.604	3.785	1259.52	1240.23	0.282	0.147
20	1290.38	92.494	5.683	1305.81	1274.95	0.673	0.422
21	1323.17	95.048	1.813	1328.95	1307.74	0.326	0.102
22	1361.74	92.947	2.801	1365.6	1340.53	0.493	0.112
23	1371.39	93.583	2.496	1377.17	1367.53	0.223	0.056
24	1384.89	92.549	4.175	1398.39	1379.1	0.379	0.118
25	1448.54	88.839	1.27	1452.4	1408.04	1.157	0.028
26	1462.04	86.869	4.138	1479.4	1454.33	1.287	0.357
27	1490.97	87.034	8.808	1514.12	1481.33	1.05	0.543
28	1716.65	95.421	0.908	1724.36	1705.07	0.346	0.031
29	1909.53	95.941	0.129	1911.46	1886.38	0.416	0.006
30	1923.03	95.274	0.257	1926.89	1913.39	0.266	0.008
31	1951.96	93.998	0.291	1955.82	1926.89	0.69	0.017
32	1973.18	93.066	0.909	1982.82	1957.75	0.734	0.06
33	1990.54	93.804	0.29	1994.4	1984.75	0.263	0.006
34	2009.83	93.549	0.169	2011.76	1996.32	0.436	0.013
35	2021.4	93.038	0.327	2025.26	2013.68	0.347	0.01
36	2094.69	94.799	0.124	2096.62	2081.19	0.347	0.006
37	2148.7	87.435	7.081	2167.99	2108.2	2.078	0.641
38	2173.78	93.937	0.551	2202.71	2169.92	0.775	0.044
39	2358.94	95.798	1.687	2389.8	2349.3	0.528	0.117
40	2920.23	95.254	1.29	2931.8	2893.22	0.691	0.107
41	2947.23	95.364	0.925	2956.87	2933.73	0.422	0.047

Comment;
RPB3-246

Date/Time; 12/03/2021 12:55:29
No. of Scans; 16
Resolution; 4 [1/cm]
Apodization; Happ-Genzel

Figure S135. FT-IR spectrum of 1-ethynyl-5,6-dimethyl-1*H*-benzo[*d*]imidazole **5**.

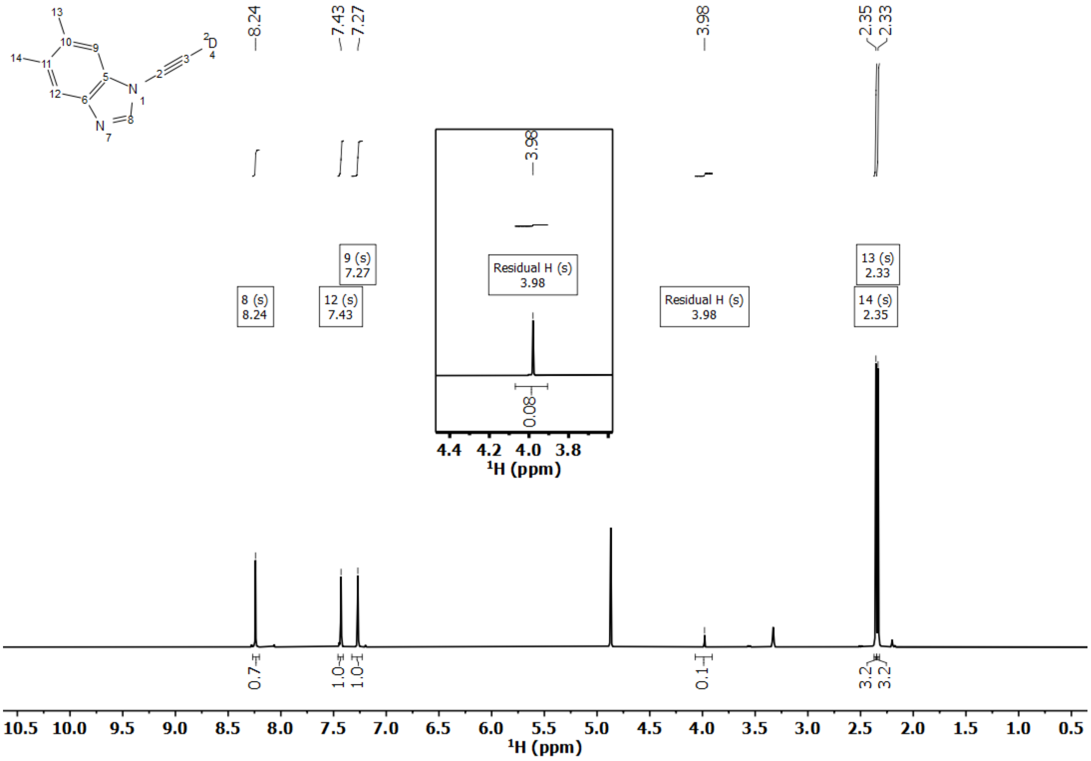

Figure S136. ^{1}H NMR spectrum of 1-(ethynyl-*d*)-5,6-dimethyl-1*H*-benzo[*d*]imidazole **5-D**.

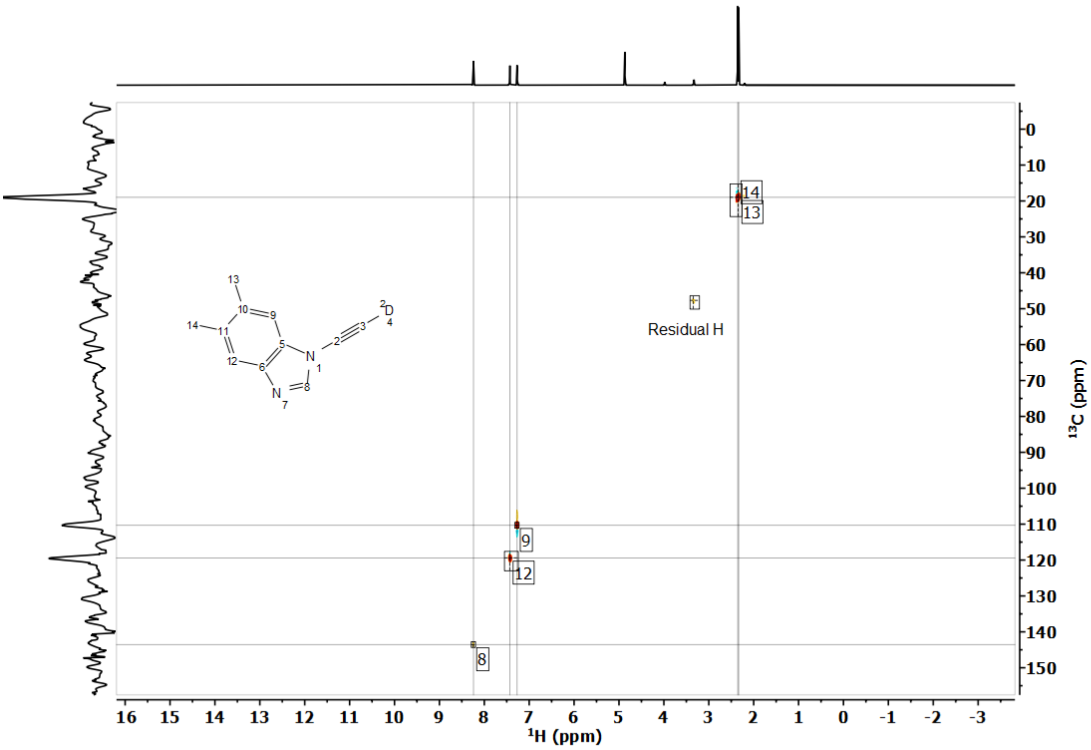

Figure S137. ^{1}H-^{13}C HSQC NMR spectrum of 1-(ethynyl-*d*)-5,6-dimethyl-1*H*-benzo[*d*]imidazole **5-D**.

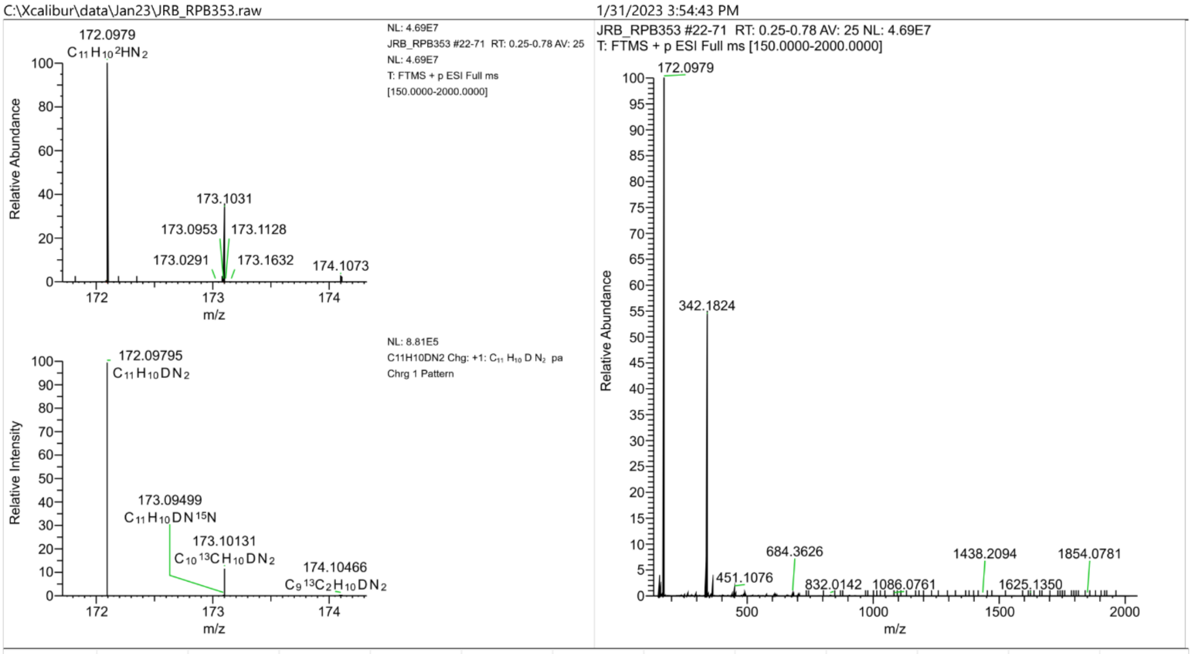

Peak Mass	Display For...	S Fit	RDB	Delta [ppm]	Theo. mass	Rank	Combined S...	# Matched I...	# Missed Iso.	MS Cov. [%]	Pattern Cov....	MSMS Matc...
172.0979	$C_2H_6{}^2H_4O_4N_5$	0	0.50	0.26	172.09784	1	0	1	3	0	0	(Collection)
172.0979	$C_{11}H_{10}{}^2HN_2$	0	7.50	-0.40	172.09795	2	0	1	3	0	0	(Collection)
172.0979	$H_6{}^2H_3O_3N_8$	0	0.50	-0.93	172.09804	3	0	2	3	0	0	(Collection)
172.0979	$C_4H_6{}^2H_5O_5N_2$	0	0.50	1.46	172.09763	4	0	0	4	0	0	(Collection)
172.0979	$C_9H_2{}^2H_7O_3$	0	5.50	-3.93	172.09856	5	0	0	4	0	0	(Collection)

Figure S138. HRMS analysis of 1-(ethynyl-*d*)-5,6-dimethyl-1*H*-benzo[*d*]imidazole **5-D**.

SHIMADZU

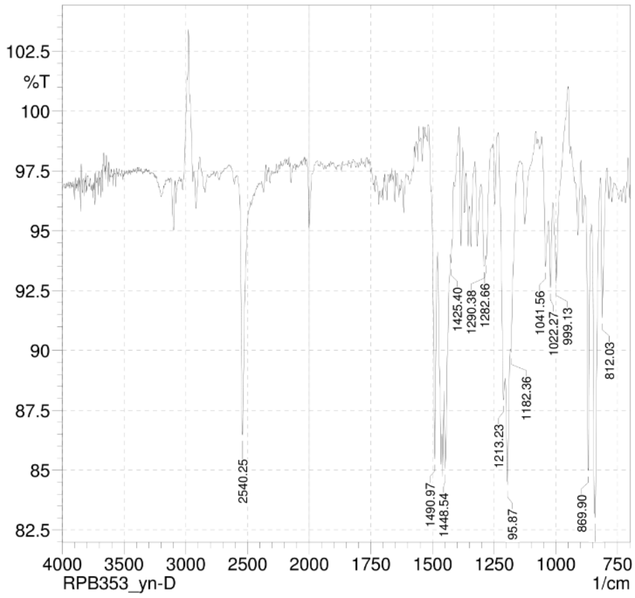

No.	Peak	Intensity	Corr. Inte	Base (H)	Base (L)	Area	Corr. Are
1	812.03	91.366	5.682	817.82	794.67	0.535	0.255
2	840.96	83.007	12.947	854.47	819.75	1.479	0.901
3	869.9	84.976	10.959	881.47	856.39	0.898	0.435
4	999.13	92.805	4.268	1010.7	950.91	0.742	0.383
5	1022.27	92.621	3.139	1029.99	1012.63	0.428	0.108
6	1041.56	93.499	3.234	1056.99	1031.92	0.495	0.162
7	1182.36	89.921	0.552	1184.29	1147.65	0.795	0.012
8	1195.87	84.377	5.04	1205.51	1186.22	1.149	0.217
9	1213.23	87.924	3.241	1232.51	1207.44	0.883	0.14
10	1282.66	93.807	0.568	1284.59	1263.37	0.329	0.016
11	1290.38	93.522	1.429	1301.95	1284.59	0.424	0.06
12	1425.4	93.643	0.886	1427.32	1415.75	0.258	0.041
13	1448.54	85.058	3.145	1452.4	1429.25	1.12	0.132
14	1490.97	85.47	9.843	1517.98	1481.33	1.078	0.509
15	2540.25	86.466	10.39	2584.61	2476.6	3.082	1.531

Comment;
RPB353_yn-D

Date/Time; 30/01/2023 13:02:00
No. of Scans; 16
Resolution; 4 [1/cm]
Apodization; Happ-Genzel

Figure S139. FT-IR spectrum of 1-(ethynyl-*d*)-5,6-dimethyl-1*H*-benzo[*d*]imidazole **5-D**.

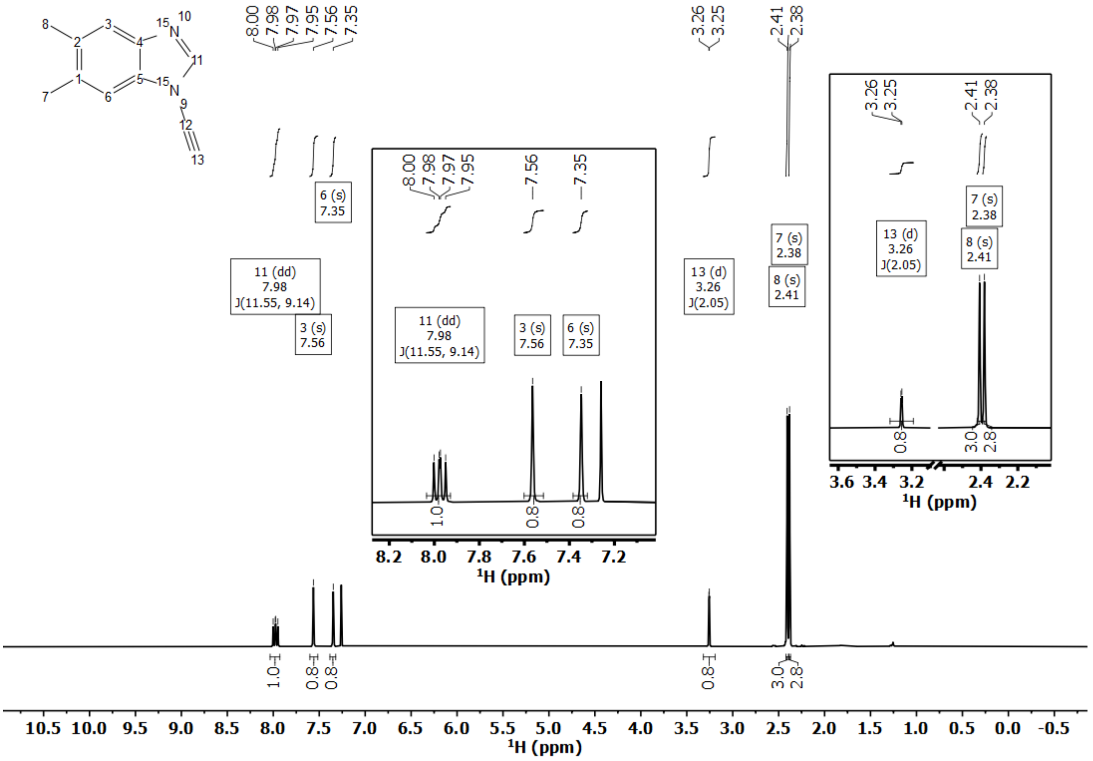

Figure S140. ^{1}H NMR spectrum of 1-ethynyl-5,6-dimethyl-1*H*-benzo[*d*]imidazole-1,3-$^{15}N_2$ **5-^{15}N$_2$**.

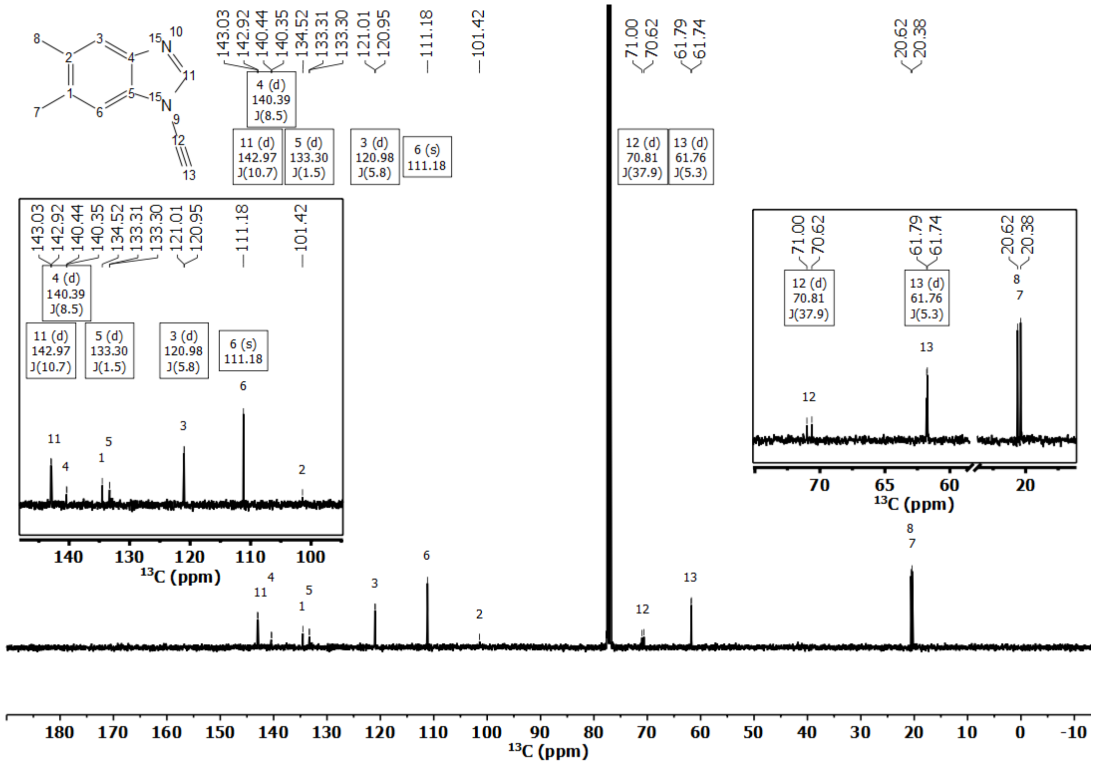

Figure S141. ^{13}C{^{1}H} NMR spectrum of 1-ethynyl-5,6-dimethyl-1*H*-benzo[*d*]imidazole-1,3-$^{15}N_2$ **5-^{15}N$_2$**.

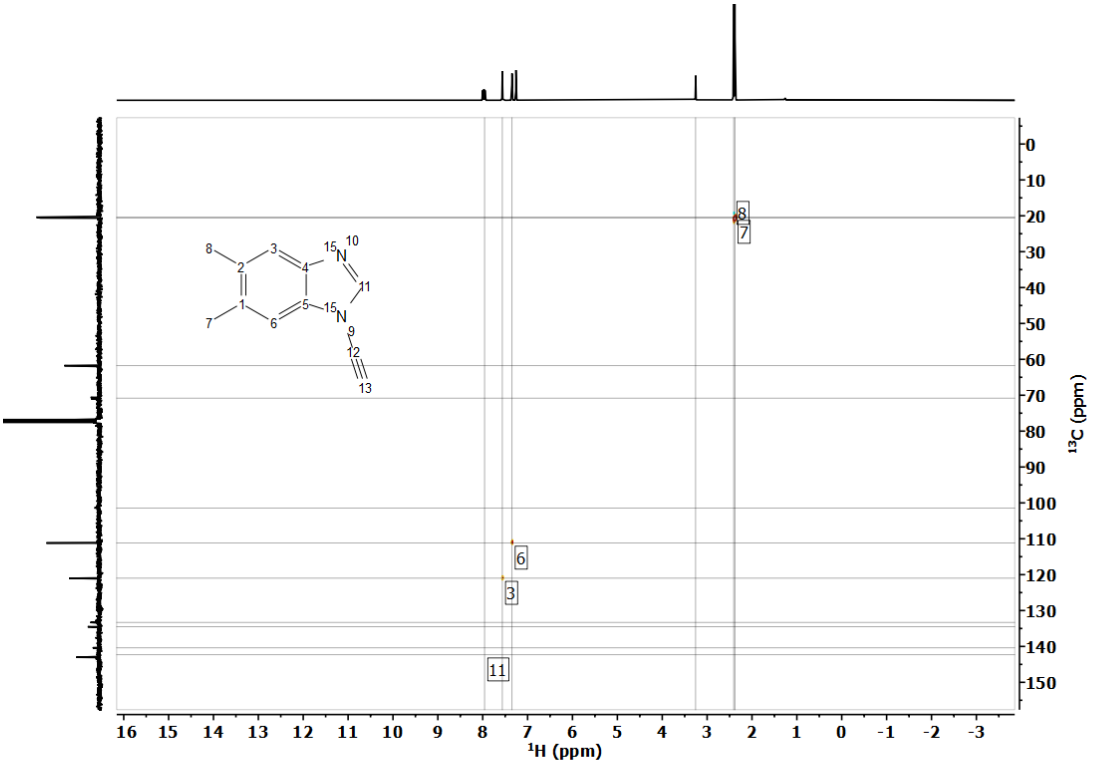

Figure S142. ^{1}H-^{13}C HSQC NMR spectrum of 1-ethynyl-5,6-dimethyl-1*H*-benzo[*d*]imidazole-1,3-$^{15}N_2$ **5-$^{15}N_2$.**

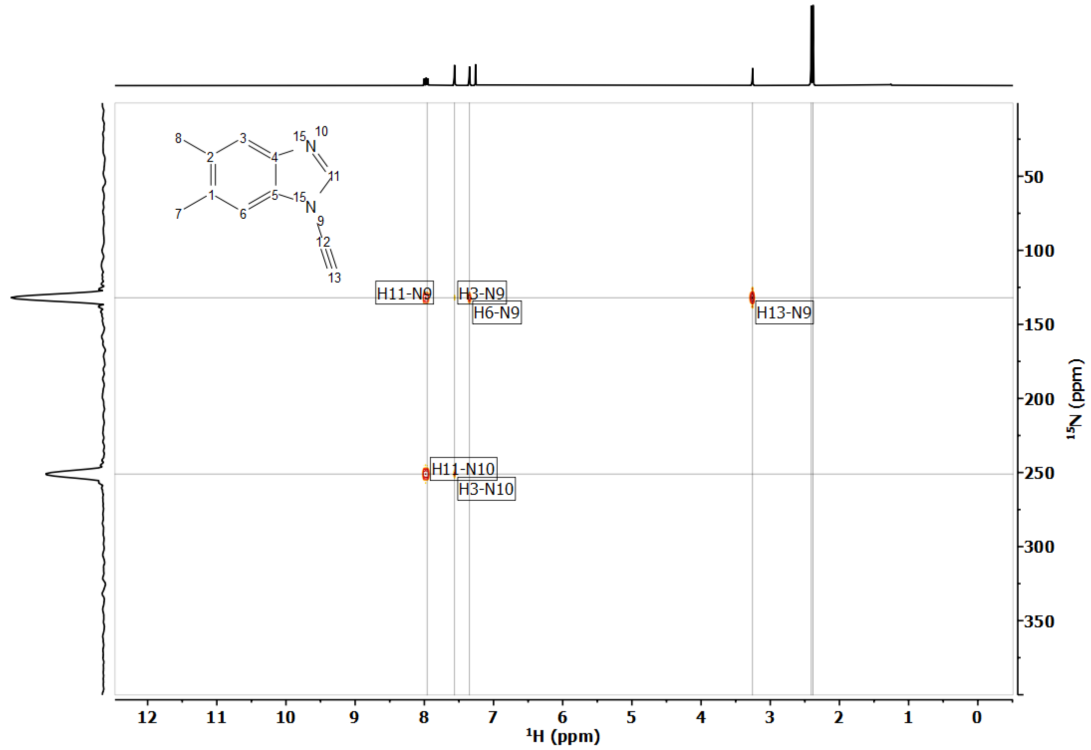

Figure S143. ^{1}H-^{15}N HMBC NMR spectrum of 1-ethynyl-5,6-dimethyl-1*H*-benzo[*d*]imidazole-1,3-$^{15}N_2$ **5-$^{15}N_2$.**

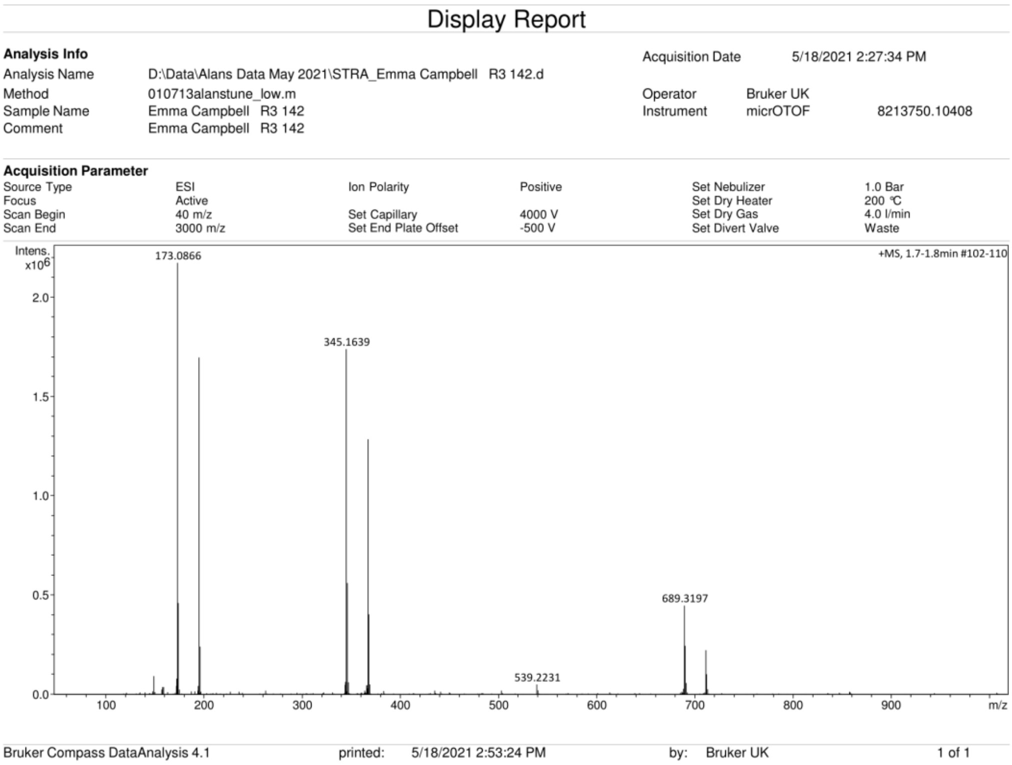

Figure S144. HRMS analysis of 1-ethynyl-5,6-dimethyl-1*H*-benzo[*d*]imidazole-1,3-$^{15}N_2$ **5-^{15}N$_2$**.

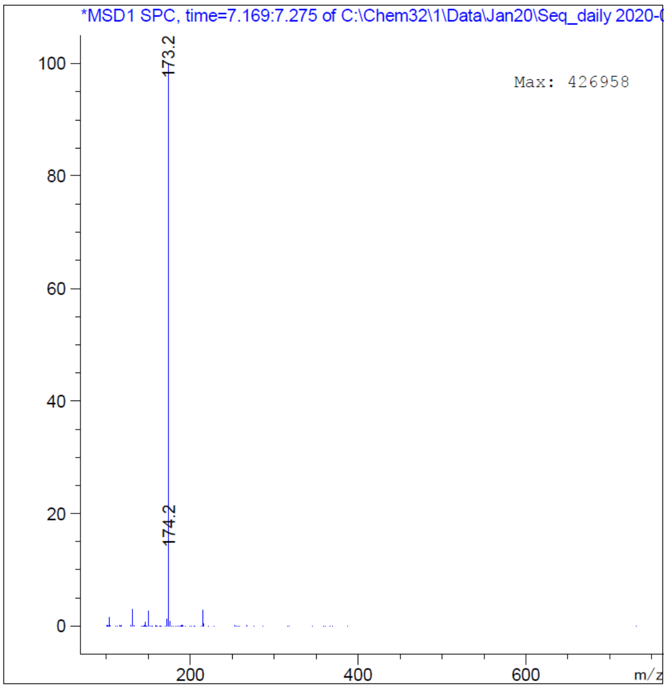

Figure S145. LC-MS analysis of 1-ethynyl-5,6-dimethyl-1*H*-benzo[*d*]imidazole-1,3-$^{15}N_2$ **5-^{15}N$_2$**.

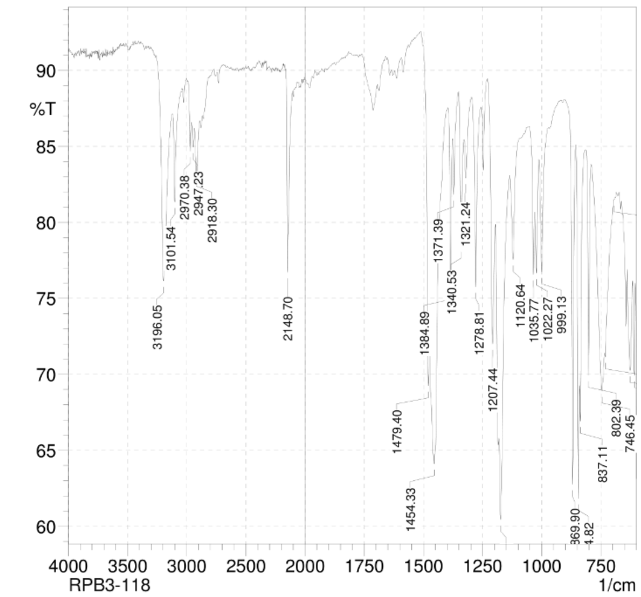

SHIMADZU

No.	Peak	Intensity	Corr. Inte	Base (H)	Base (L)	Area	Corr. Are
1	609.51	69.928	6.132	615.29	605.65	1.332	0.186
2	626.87	70.255	5.958	638.44	617.22	2.957	0.453
3	694.37	81.481	0.094	696.3	684.73	1.018	0.007
4	731.02	71.145	1.278	734.88	696.3	4.399	0.033
5	746.45	68.904	5.152	788.89	734.88	6.716	0.635
6	802.39	69.944	14.392	812.03	790.81	2.125	0.546
7	837.11	66.918	4.92	840.96	817.82	2.43	0.111
8	844.82	61.838	11.93	854.47	840.96	2.005	0.382
9	869.9	62.758	23.221	902.69	856.39	3.997	1.116
10	999.13	76.736	7.958	1010.7	972.12	3.187	0.503
11	1022.27	76.656	5.314	1028.06	1012.63	1.479	0.199
12	1035.77	76.573	5.609	1051.2	1029.99	1.85	0.176
13	1120.64	77.544	6.363	1134.14	1095.57	3.281	0.428
14	1172.72	60.434	8.307	1180.44	1136.07	6.05	0.572
15	1207.44	71.998	10.522	1228.66	1197.79	3.027	0.7
16	1278.81	75.756	12.209	1296.16	1255.66	2.971	0.702
17	1321.24	82.918	2.997	1327.03	1298.09	1.914	0.149
18	1340.53	78.442	8.68	1350.17	1328.95	1.683	0.382
19	1371.39	81.819	4.209	1375.25	1352.1	1.505	0.127
20	1384.89	75.727	10.501	1396.46	1377.17	1.616	0.385
21	1454.33	64.13	12.543	1473.62	1398.39	8.935	1.798
22	1479.4	69.246	6.725	1510.26	1475.54	2.597	0.206
23	2148.7	76.703	13.018	2164.13	2104.34	4.287	1.362
24	2918.3	83.17	3.214	2935.66	2889.37	3.269	0.34
25	2947.23	84.948	1.445	2958.8	2935.66	1.544	0.074
26	2970.38	84.675	2.874	2991.59	2958.8	1.971	0.14
27	3101.54	81.336	6.155	3118.9	3049.46	4.515	0.618
28	3196.05	76.14	13.114	3257.77	3120.82	10.365	3.445

Comment;
RPB3-118

Date/Time; 12/03/2021 13:26:09
No. of Scans; 16
Resolution; 4 [1/cm]
Apodization; Happ-Genzel

Figure S146. FT-IR spectrum of 1-ethynyl-5,6-dimethyl-1*H*-benzo[*d*]imidazole-1,3-$^{15}N_2$ **5-$^{15}N_2$**.

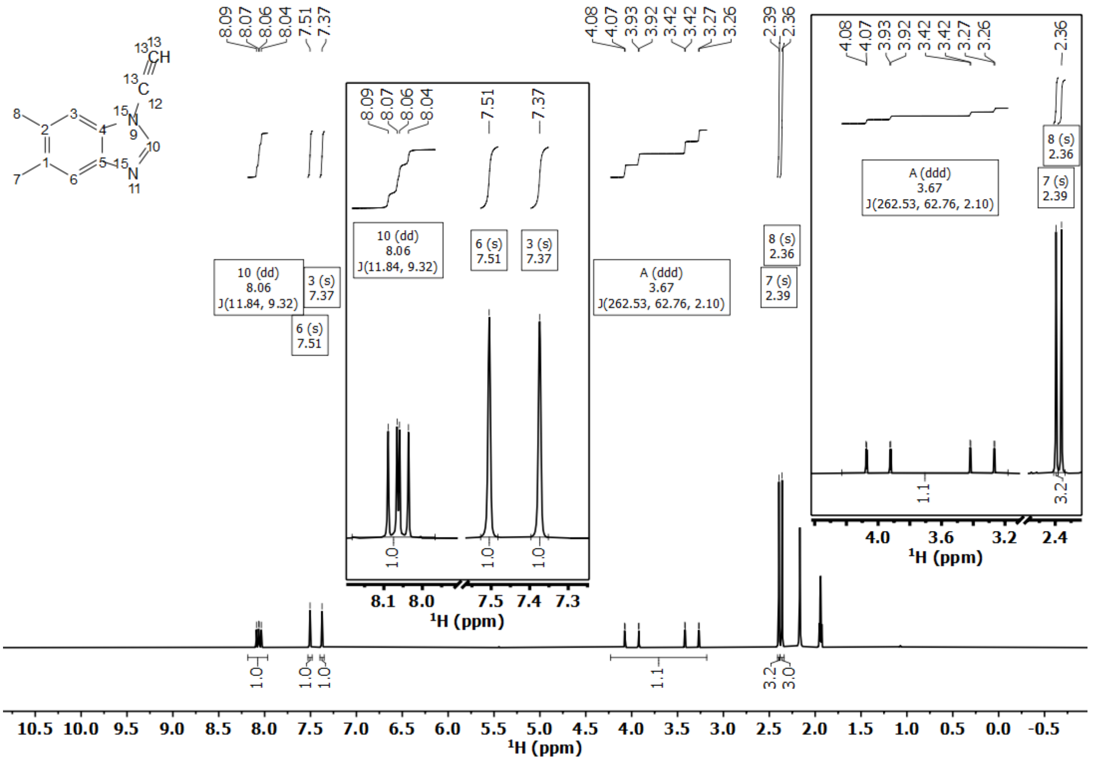

Figure S147. ^{1}H NMR spectrum of 1-(ethynyl-$^{13}C_2$)-5,6-dimethyl-1*H*-benzo[*d*]imidazole-1,3-$^{15}N_2$ **5-$^{13}C_2$-$^{15}N_2$**.

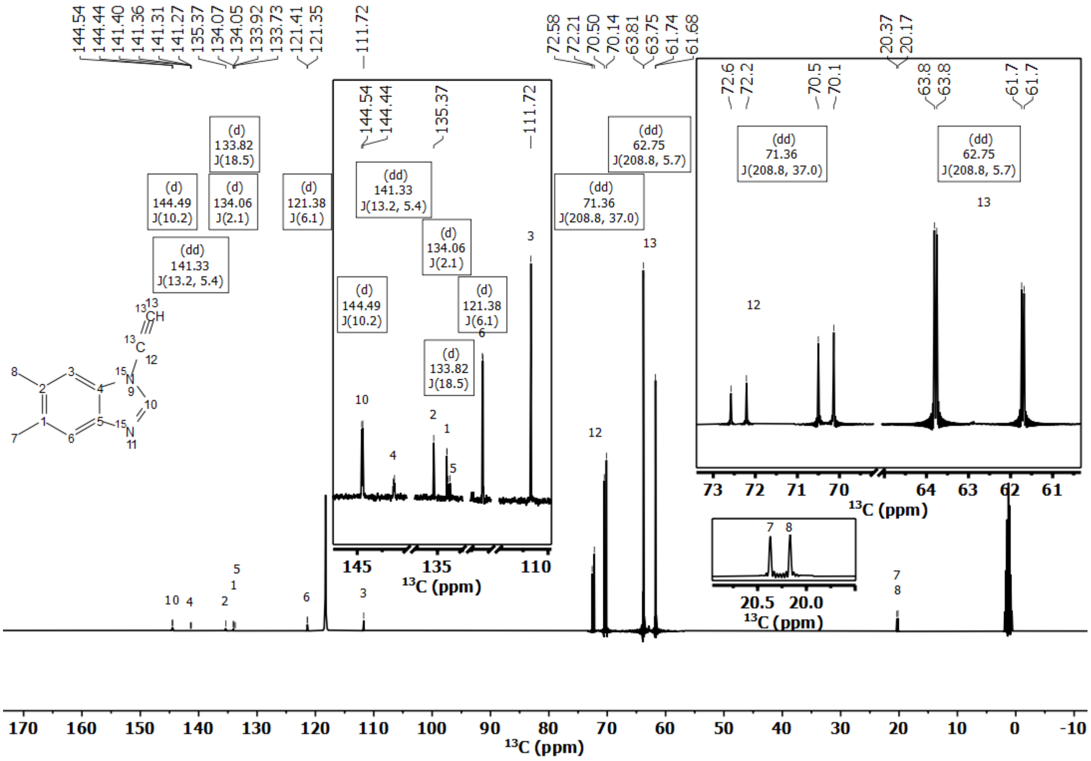

Figure S148. ^{13}C{^{1}H} NMR spectrum of 1-(ethynyl-$^{13}C_2$)-5,6-dimethyl-1*H*-benzo[*d*]imidazole-1,3-$^{15}N_2$ **5-^{13}C$_2$-^{15}N$_2$.**

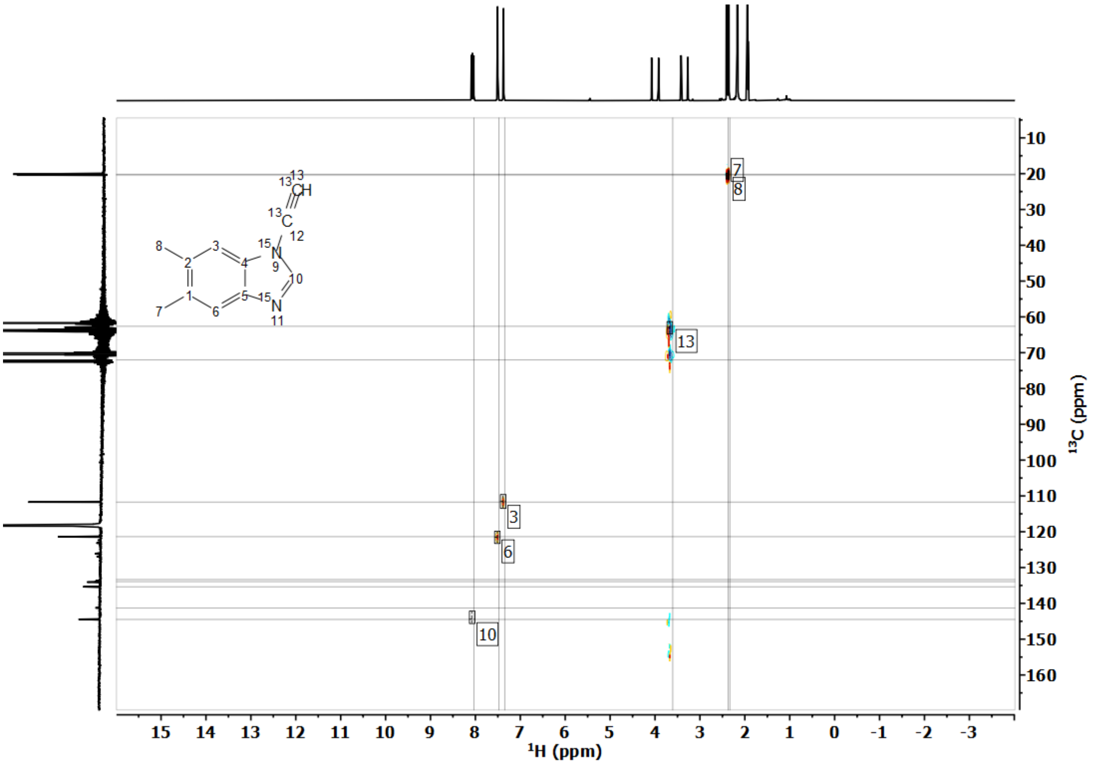

Figure S149. ^{1}H-^{13}C HSQC NMR spectrum of 1-(ethynyl-$^{13}C_2$)-5,6-dimethyl-1*H*-benzo[*d*]imidazole-1,3-$^{15}N_2$ **5-^{13}C$_2$-^{15}N$_2$.**

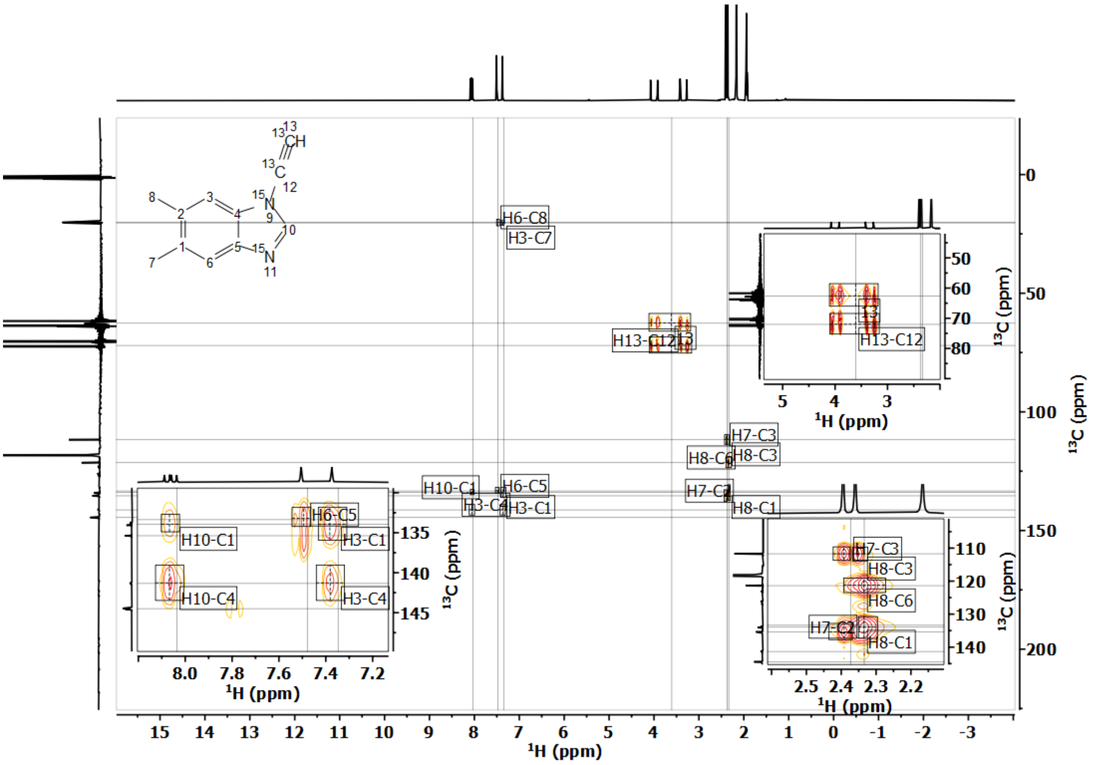

Figure S150. ^{1}H-^{13}C HMBC NMR spectrum of 1-(ethynyl-$^{13}C_2$)-5,6-dimethyl-1*H*-benzo[*d*]imidazole-1,3-$^{15}N_2$ **5-^{13}C$_2$-^{15}N$_2$.**

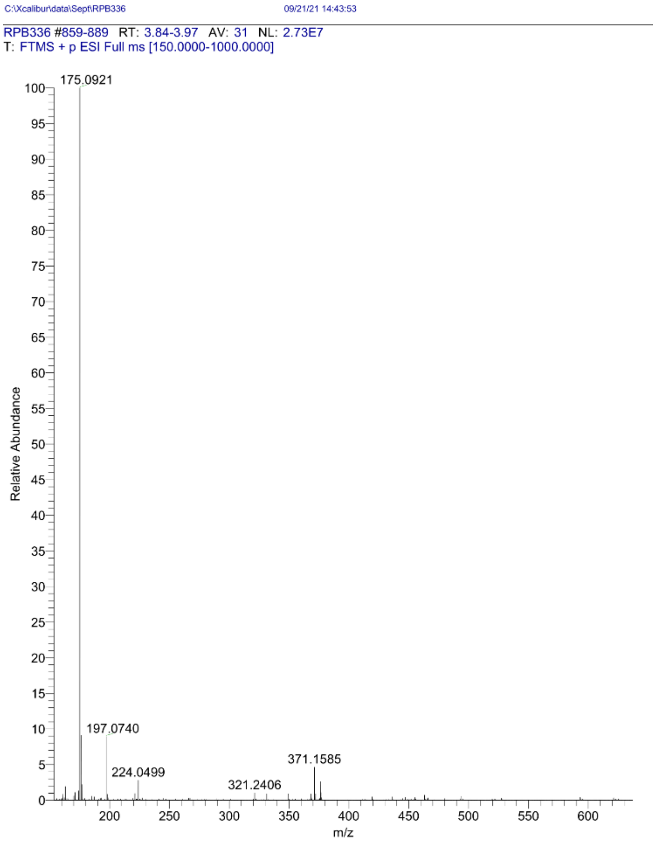

Figure S151. HRMS analysis of 1-(ethynyl-$^{13}C_2$)-5,6-dimethyl-1*H*-benzo[*d*]imidazole-1,3-$^{15}N_2$ **5-^{13}C$_2$-^{15}N$_2$.**

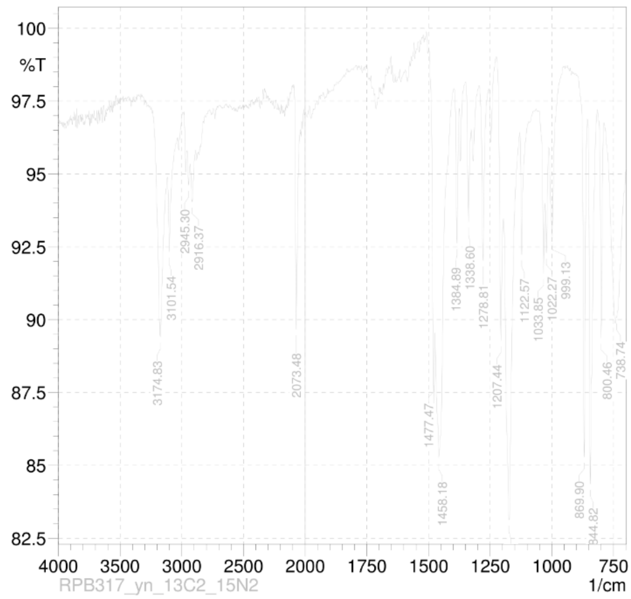

No.	Peak	Intensity	Corr. Inte	Base (H)	Base (L)	Area	Corr. Are
1	738.74	90.068	0.24	740.67	698.23	1.413	0.044
2	800.46	89.377	7.5	815.89	790.81	0.587	0.246
3	844.82	84.361	12.553	854.47	817.82	1.312	0.826
4	869.9	85.282	12.059	902.69	856.39	0.975	0.499
5	999.13	92.813	3.624	1010.7	956.69	0.834	0.2
6	1022.27	92.293	1.984	1026.13	1012.63	0.352	0.048
7	1033.85	91.585	3.189	1051.2	1028.06	0.542	0.082
8	1122.57	92.233	4.11	1128.36	1095.57	0.678	0.175
9	1174.65	83.131	11.298	1195.87	1130.29	2.836	1.37
10	1207.44	89.282	6.13	1224.8	1197.79	0.713	0.246
11	1278.81	92.025	5.958	1294.24	1255.66	0.639	0.296
12	1338.6	93.362	4.025	1348.24	1327.03	0.405	0.147
13	1384.89	92.623	4.799	1396.46	1377.17	0.367	0.152
14	1458.18	85.27	6.002	1473.62	1398.39	2.749	0.698
15	1477.47	87.955	2.308	1498.69	1475.54	0.657	0.107
16	2073.48	89.672	7.747	2092.77	2042.62	1.242	0.623
17	2916.37	94.04	1.463	2931.8	2889.37	0.963	0.129
18	2945.3	94.616	0.953	2956.87	2933.73	0.505	0.047
19	3101.54	92.327	2.802	3116.97	3061.03	1.408	0.25
20	3174.83	89.406	6.551	3234.62	3118.9	3.515	1.448

Comment;
RPB317_yn_13C2_15N2

Date/Time; 30/01/2023 13:59:07
No. of Scans; 16
Resolution; 4 [1/cm]
Apodization; Happ-Genzel

Figure S152. FT-IR spectrum of 1-(ethynyl-$^{13}C_2$)-5,6-dimethyl-1*H*-benzo[*d*]imidazole-1,3-$^{15}N_2$ **5-^{13}C$_2$-^{15}N$_2$.**

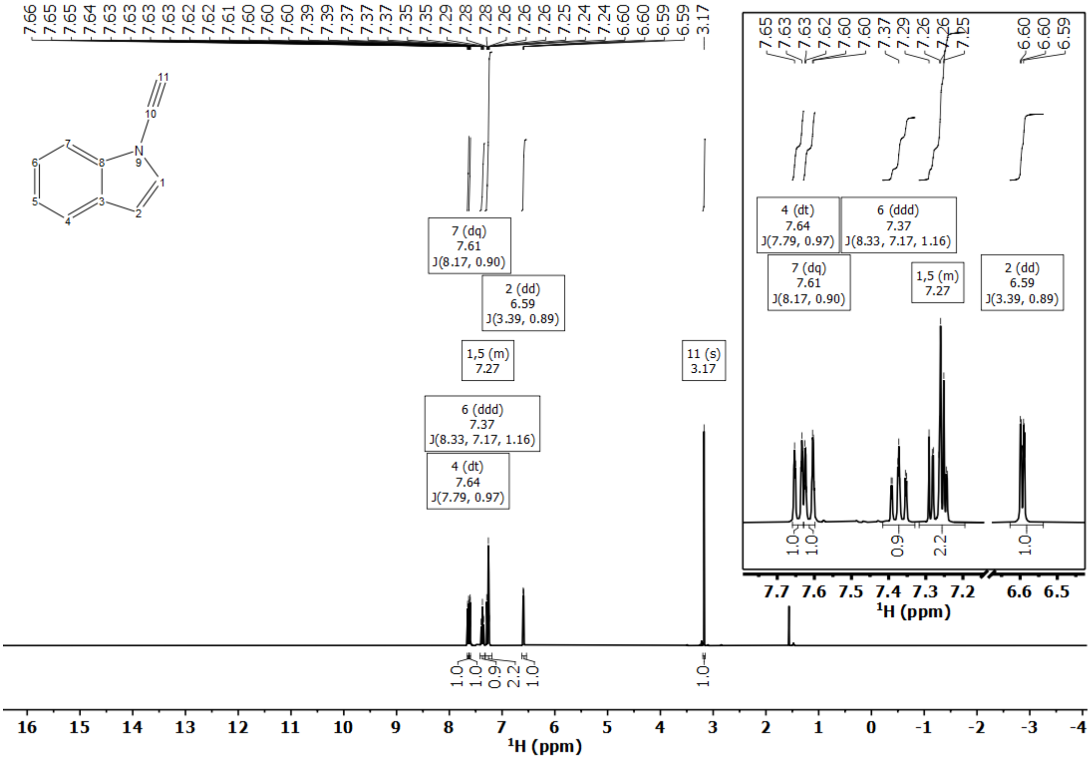

Figure S153. ^{1}H NMR spectrum of 1-ethynyl-1*H*-indole **8**.

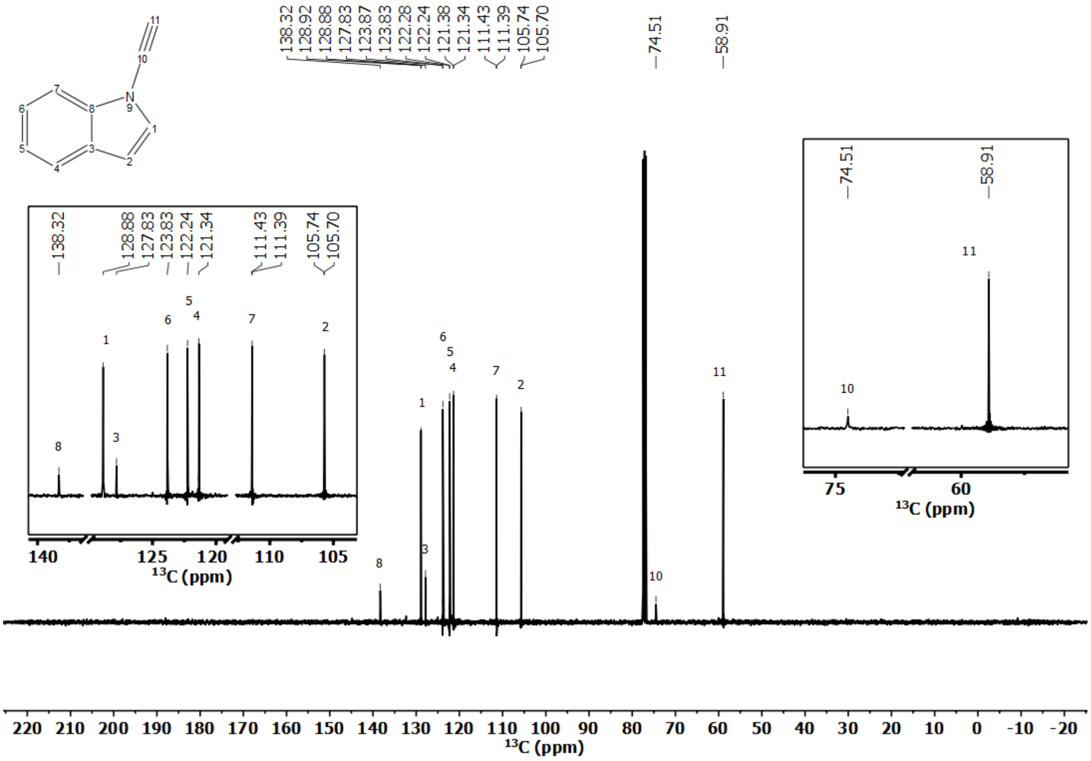

Figure S154. $^{13}C\{^{1}H\}$ NMR spectrum of 1-ethynyl-1*H*-indole **8**.

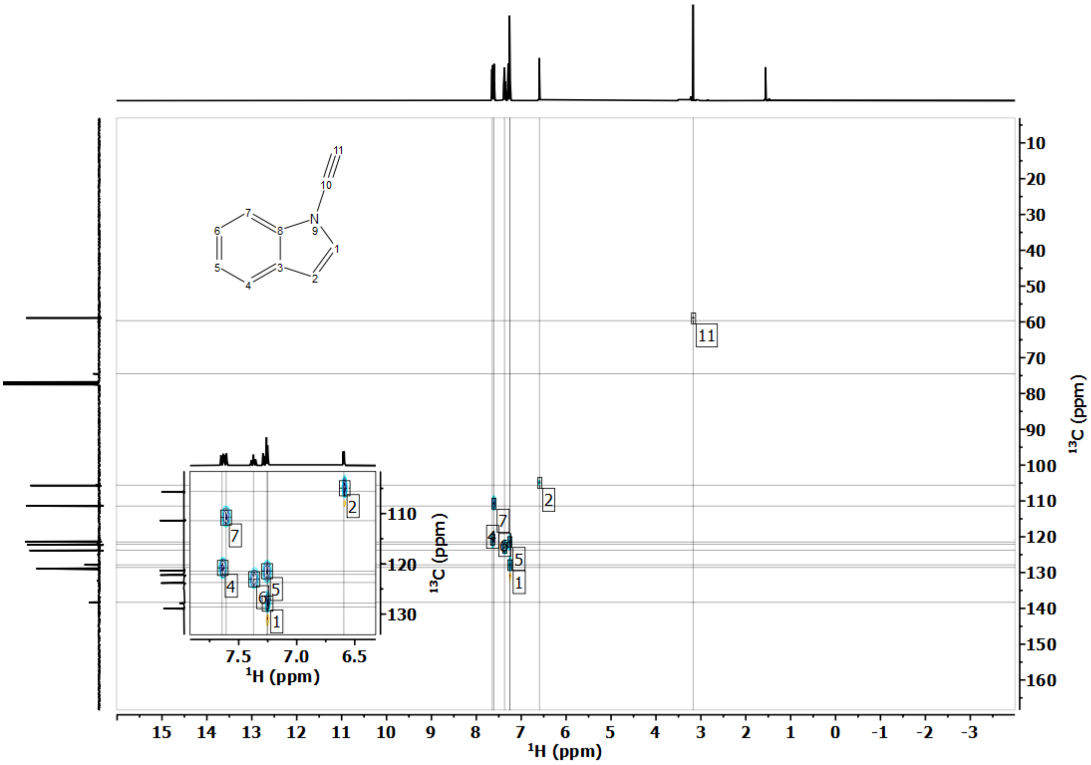

Figure S155. ^{1}H-^{13}C HSQC NMR spectrum of 1-ethynyl-1*H*-indole **8**.

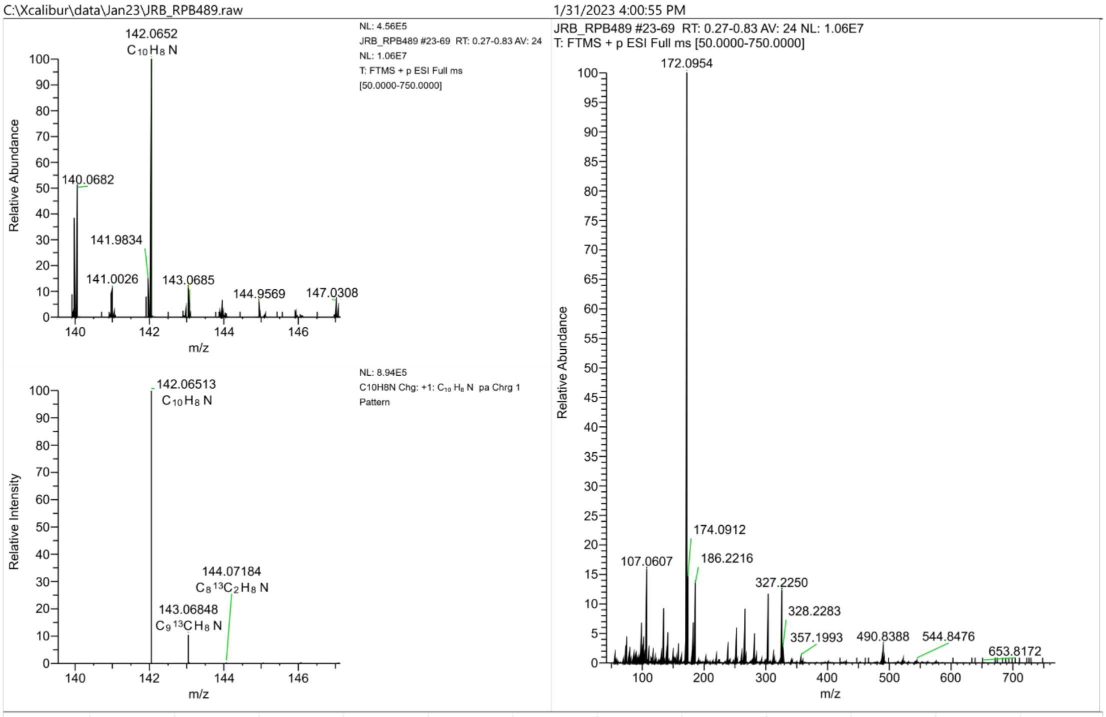

Peak Mass	Display For...	S Fit	RDB	Delta [ppm]	Theo. mass	Rank	Combined S...	# Matched I...	# Missed Iso.	MS Cov. [%]	Pattern Cov....	MSMS Matc...
142.0652	$C_{10}H_8N$	29.11212061 42962	7.50	0.43	142.06513	1	79.23	4	0	82.01	99.2	(Collection)

Figure S156. HRMS analysis of 1-ethynyl-1*H*-indole **8**.

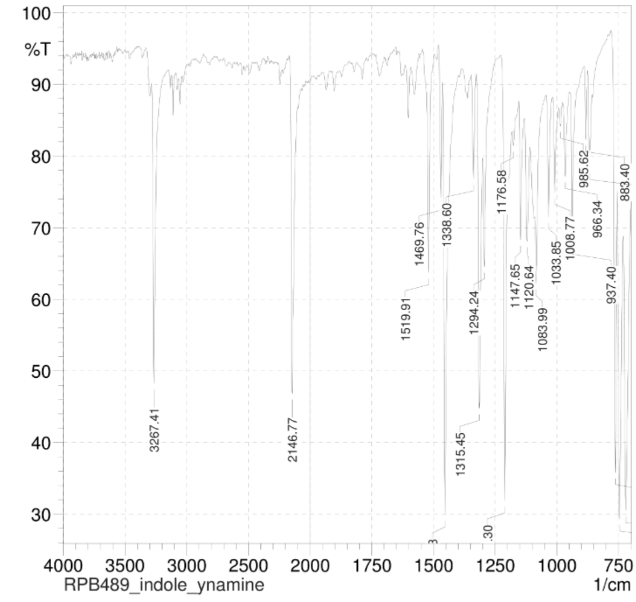

SHIMADZU

No.	Peak	Intensity	Corr. Inte	Base (H)	Base (L)	Area	Corr. Are
1	721.38	30.507	39.984	731.02	698.23	9.622	4.9
2	746.45	29.312	40.347	754.17	732.95	7.301	3.921
3	763.81	35.815	46.186	781.17	756.1	4.264	2.573
4	867.97	78.406	11.666	873.75	858.32	1.208	0.472
5	883.4	82.294	9.616	887.26	875.68	0.673	0.25
6	937.4	67.967	20.519	943.19	889.18	3.308	0.926
7	966.34	77.148	10.943	977.91	950.91	2.021	0.553
8	985.62	84.16	2.786	991.41	979.84	0.786	0.087
9	1008.77	74.913	12.714	1016.49	993.34	1.921	0.561
10	1033.85	71.505	16.896	1043.49	1018.41	2.055	0.708
11	1083.99	62.188	11.613	1089.78	1045.42	4.117	0.412
12	1120.64	68.108	14.758	1130.29	1111	2.466	0.867
13	1147.65	68.277	18.706	1153.43	1132.21	2.127	0.772
14	1176.58	81.429	2.925	1182.36	1155.36	1.958	0.145
15	1211.3	31.873	58.226	1226.73	1184.29	6.752	4.444
16	1294.24	64.557	16.623	1300.02	1244.09	3.766	0.675
17	1315.45	44.767	41.281	1325.1	1301.95	4.317	2.606
18	1338.6	76.815	14.454	1346.31	1327.03	1.38	0.621
19	1454.33	29.892	58.996	1462.04	1400.32	8.661	5.811
20	1469.76	74.121	18.145	1479.4	1463.97	1.16	0.641
21	1519.91	63.715	28.963	1544.98	1508.33	2.651	1.528
22	2146.77	46.82	44.638	2169.92	2104.34	8.98	6.045
23	3267.41	48.242	42.073	3284.77	3184.48	10.071	5.808

Comment;
RPB489_indole_ynamine

Date/Time; 30/01/2023 14:54:43
No. of Scans; 16
Resolution; 4 [1/cm]
Apodization; Happ-Genzel

Figure S157. FT-IR spectrum of 1-ethynyl-1*H*-indole **8**.

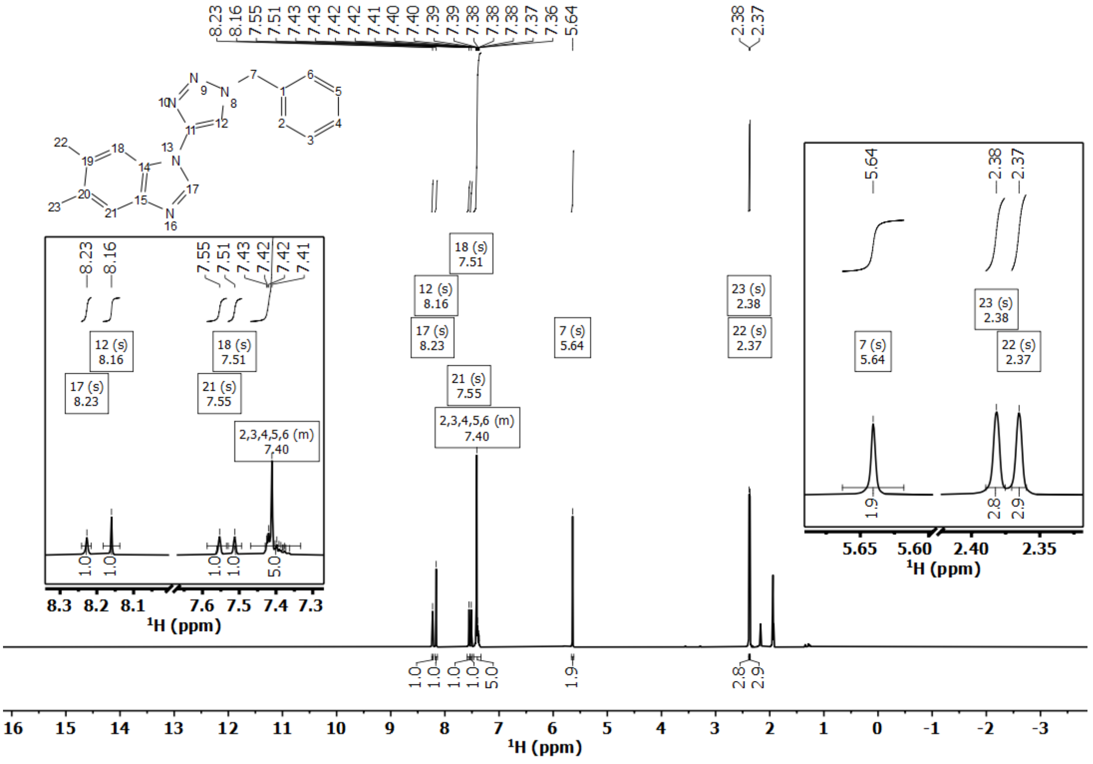

Figure S158. ^{1}H NMR spectrum of 1-(1-benzyl-1*H*-1,2,3-triazol-4-yl)-5,6-dimethyl-1*H*-benzo[*d*]imidazole **10**.

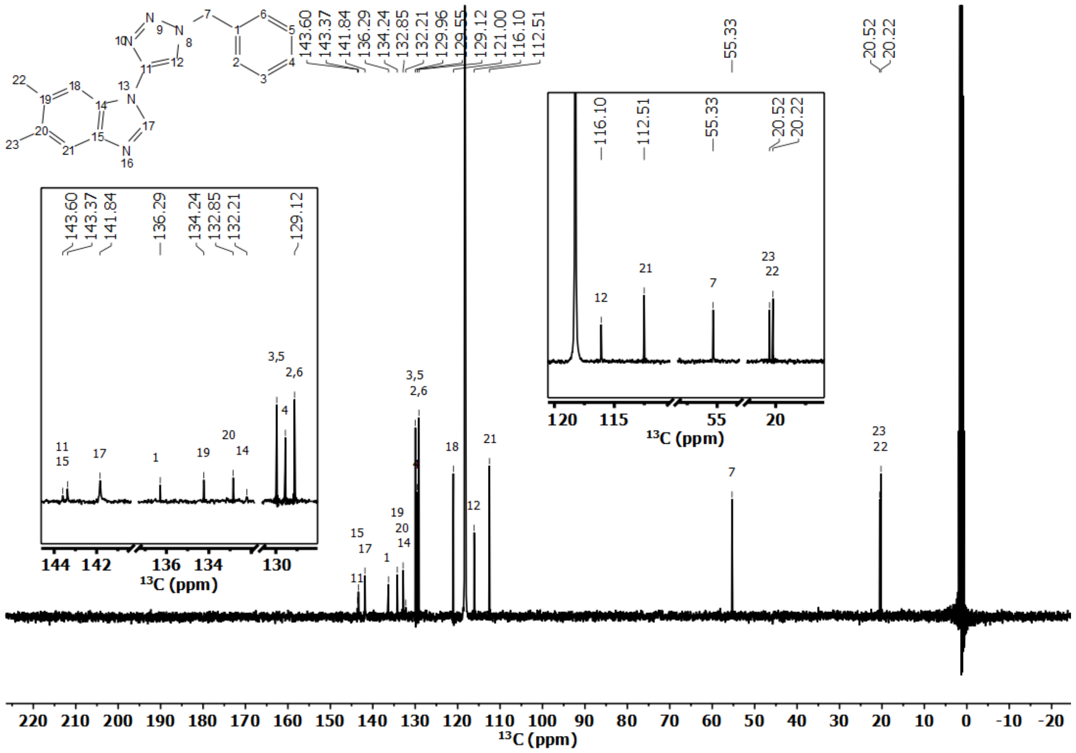

Figure S159. ^{13}C{^{1}H} NMR spectrum of 1-(1-benzyl-1*H*-1,2,3-triazol-4-yl)-5,6-dimethyl-1*H*-benzo[*d*]imidazole **10**.

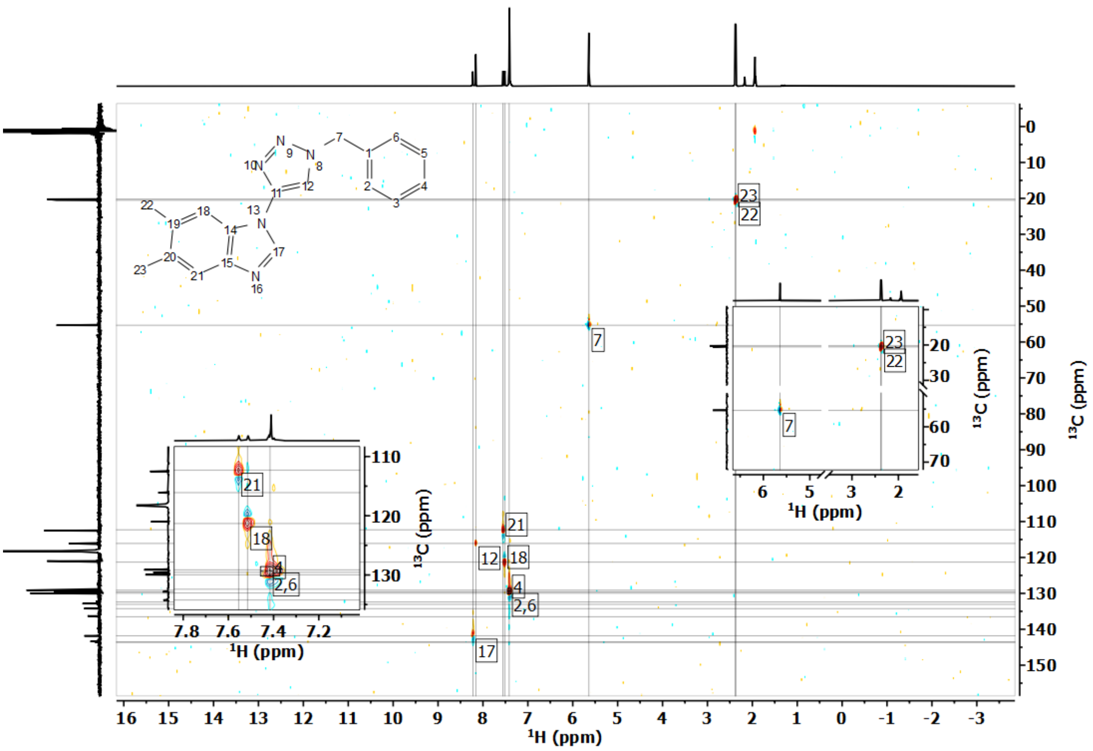

Figure S160. ^{1}H-^{13}C HSQC NMR spectrum of 1-(1-benzyl-1*H*-1,2,3-triazol-4-yl)-5,6-dimethyl-1*H*-benzo[*d*]imidazole **10**.

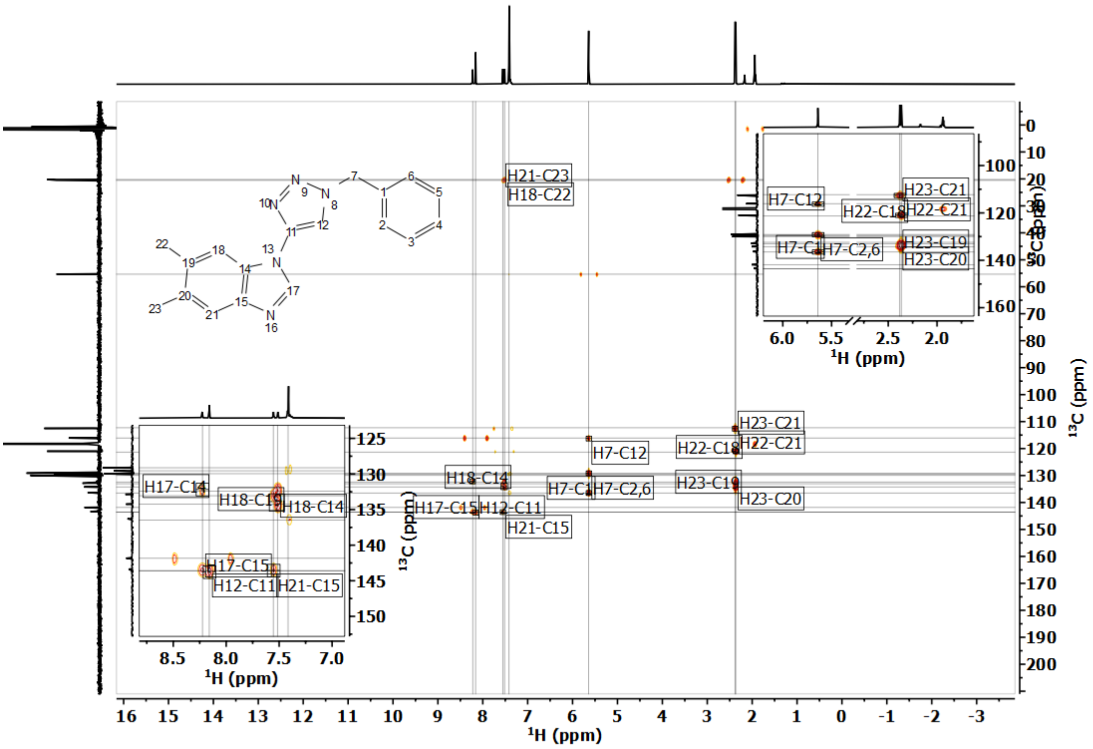

Figure S161. ^{1}H-^{13}C HMBC NMR spectrum of 1-(1-benzyl-1*H*-1,2,3-triazol-4-yl)-5,6-dimethyl-1*H*-benzo[*d*]imidazole **10**.

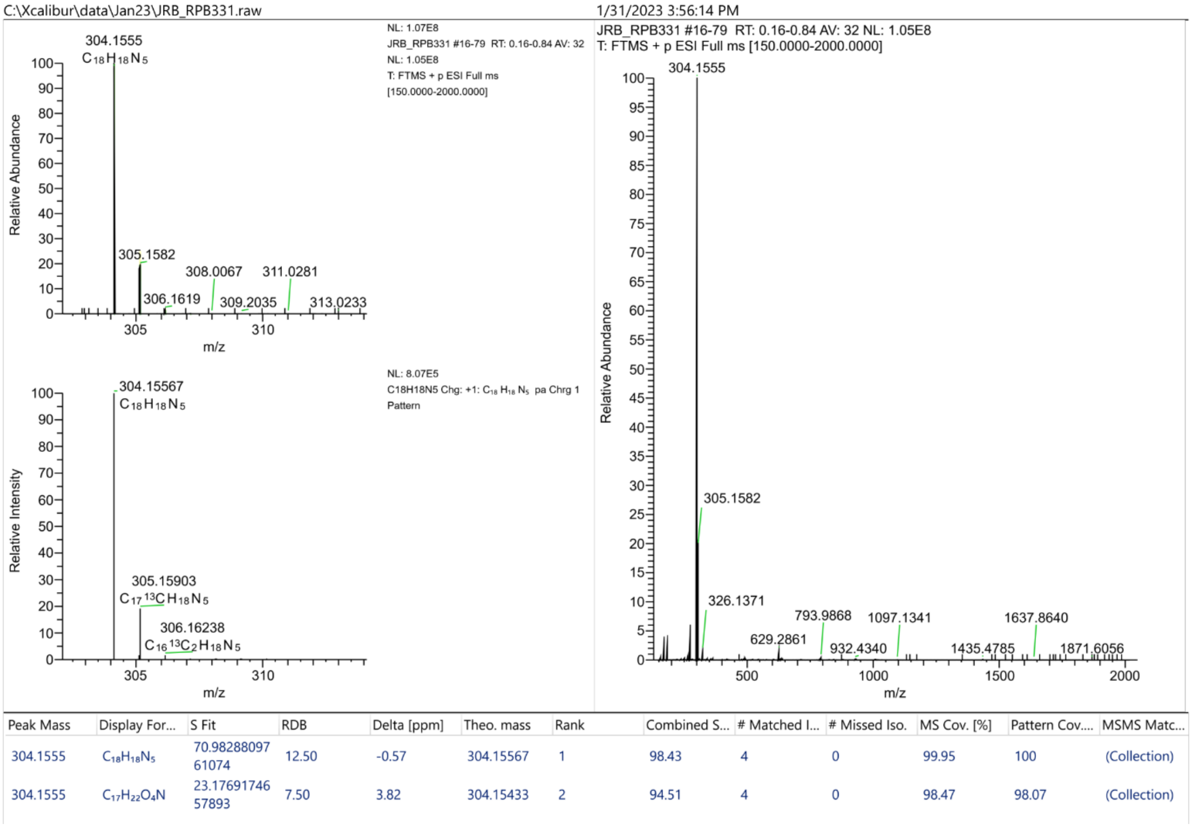

Peak Mass	Display For...	S Fit	RDB	Delta [ppm]	Theo. mass	Rank	Combined S...	# Matched I...	# Missed Iso.	MS Cov. [%]	Pattern Cov....	MSMS Matc...
304.1555	$C_{18}H_{18}N_5$	70.9828809761074	12.50	-0.57	304.15567	1	98.43	4	0	99.95	100	(Collection)
304.1555	$C_{17}H_{22}O_4N$	23.1769174657893	7.50	3.82	304.15433	2	94.51	4	0	98.47	98.07	(Collection)

Figure S162. HRMS analysis of 1-(1-benzyl-1*H*-1,2,3-triazol-4-yl)-5,6-dimethyl-1*H*-benzo[*d*]imidazole **10**.

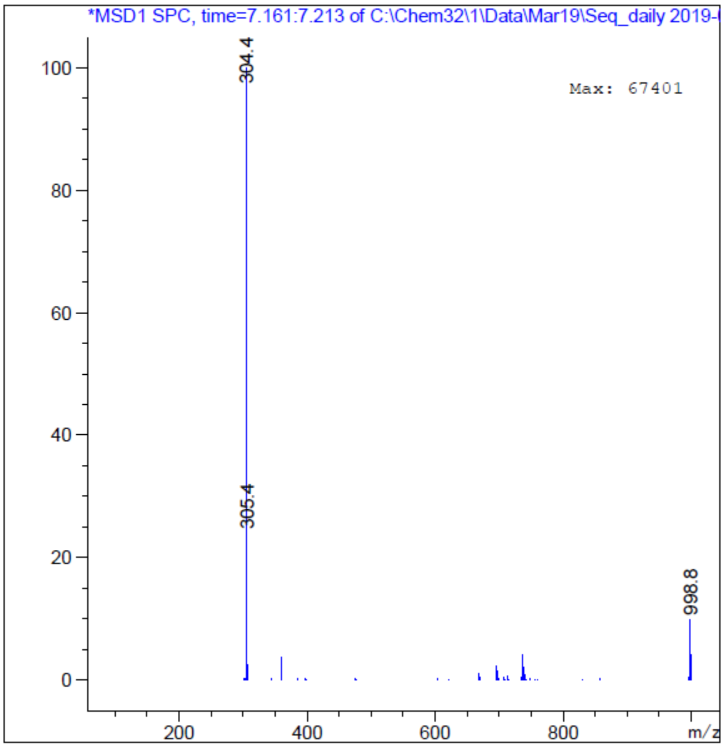

Figure S163. LC-MS analysis of 1-(1-benzyl-1*H*-1,2,3-triazol-4-yl)-5,6-dimethyl-1*H*-benzo[*d*]imidazole **10**.

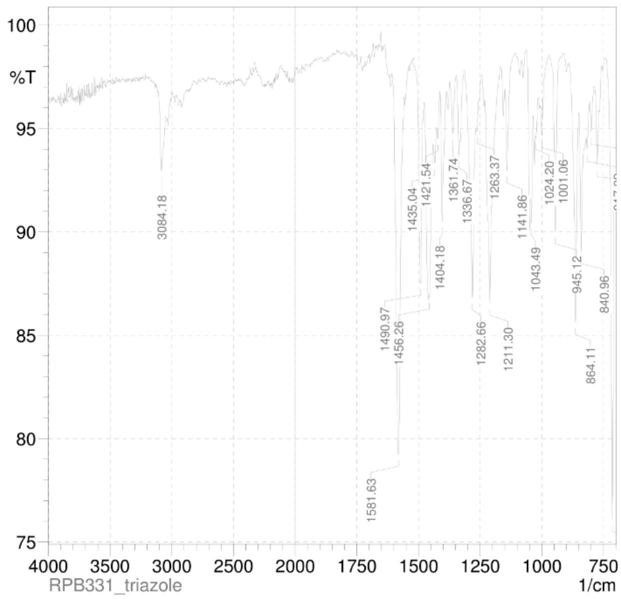

SHIMADZU

No.	Peak	Intensity	Corr. Inte	Base (H)	Base (L)	Area	Corr. Are
1	715.59	76.075	18.195	740.67	705.95	1.507	0.802
2	775.38	93.251	3.003	786.96	769.6	0.373	0.095
3	800.46	94.82	1.742	806.25	788.89	0.304	0.053
4	817.82	94.001	0.728	819.75	808.17	0.258	0.024
5	840.96	89.055	6.195	848.68	821.68	0.882	0.281
6	864.11	85.62	10.965	891.11	850.61	1.199	0.648
7	945.12	90.008	8.565	970.19	914.26	0.778	0.43
8	1001.06	94.661	1.541	1004.91	972.12	0.404	0.045
9	1024.2	94.588	0.41	1026.13	1016.49	0.196	0.013
10	1043.49	90.427	1.948	1045.42	1035.77	0.313	0.053
11	1141.86	92.945	4.298	1149.57	1109.07	0.559	0.181
12	1211.3	86.55	10.333	1230.58	1166.93	1.869	1.092
13	1263.37	94.858	0.709	1267.23	1257.59	0.195	0.016
14	1282.66	86.823	9.005	1313.52	1269.16	1.419	0.692
15	1336.67	93.688	3.928	1346.31	1315.45	0.562	0.247
16	1361.74	94.734	2.704	1373.32	1348.24	0.409	0.129
17	1404.18	90.504	6.114	1411.89	1388.75	0.64	0.293
18	1421.54	94.501	0.967	1425.4	1413.82	0.227	0.02
19	1435.04	93.311	1.284	1440.83	1427.32	0.363	0.044
20	1456.26	86.846	8.071	1475.54	1442.75	1.479	0.753
21	1490.97	87.517	9.398	1521.84	1477.47	1.121	0.591
22	1581.63	79.247	17.794	1606.7	1558.48	2.338	1.709
23	3084.18	92.94	3.33	3111.18	3047.53	1.507	0.423

Comment;
RPB331_triazole

Date/Time; 30/01/2023 14:19:14
No. of Scans; 16
Resolution; 4 [1/cm]
Apodization; Happ-Genzel

Figure S164. FT-IR spectrum of 1-(1-benzyl-1*H*-1,2,3-triazol-4-yl)-5,6-dimethyl-1*H*-benzo[*d*]imidazole **10**.

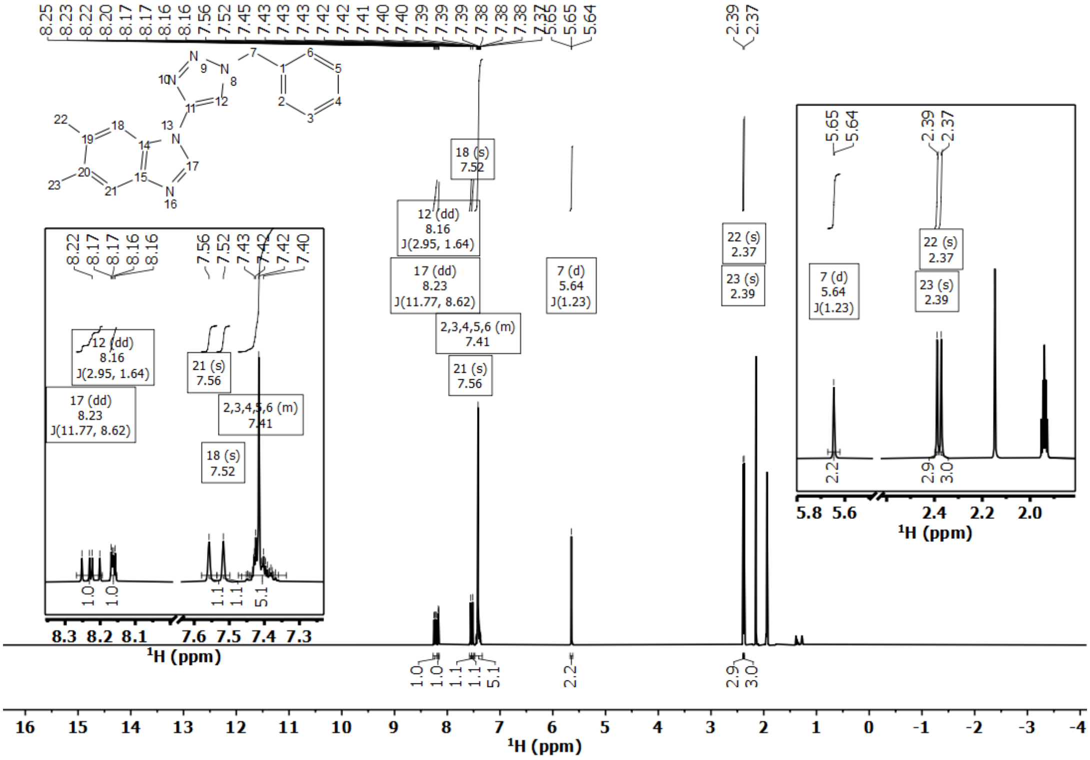

Figure S165. ^{1}H NMR spectrum of 1-(1-benzyl-1*H*-1,2,3-triazol-4-yl1/3-^{15}N)-5,6-dimethyl-1*H*-benzo[*d*]imidazole-1,3-$^{15}N_2$ **10-^{15}N$_4$.**

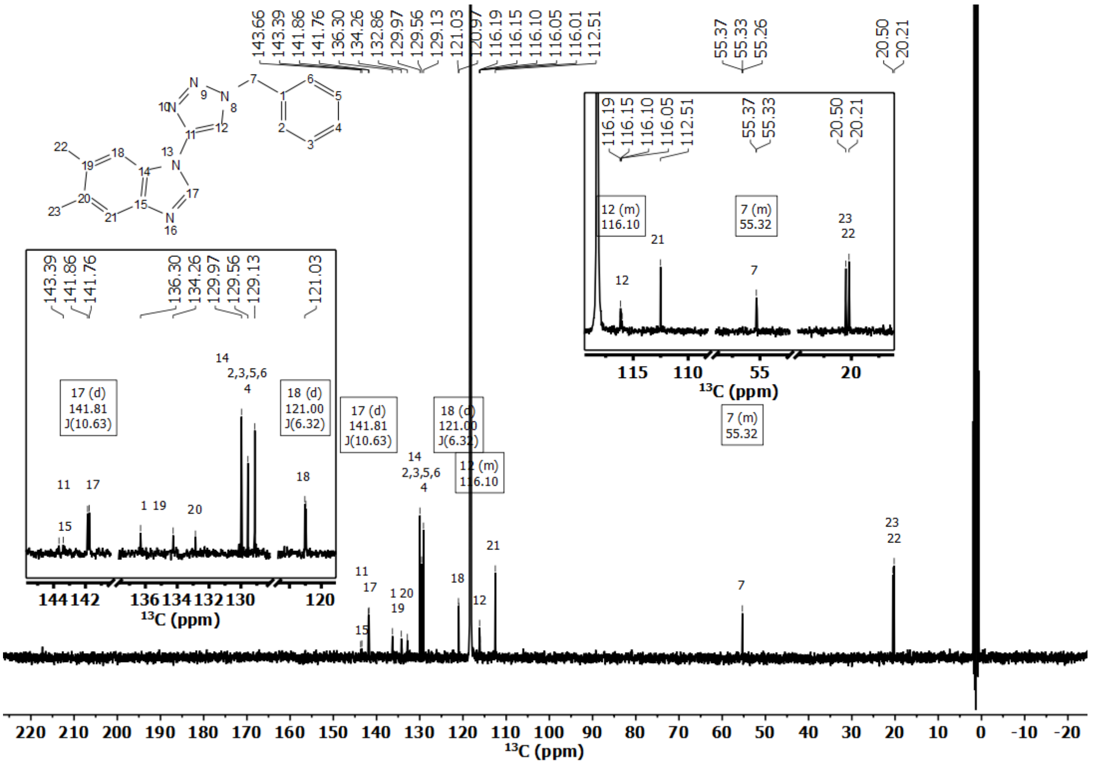

Figure S166. $^{13}C\{^{1}H\}$ NMR spectrum of 1-(1-benzyl-1*H*-1,2,3-triazol-4-yl1/3-^{15}N)-5,6-dimethyl-1*H*-benzo[*d*]imidazole-1,3-$^{15}N_2$ **10-^{15}N$_4$.**

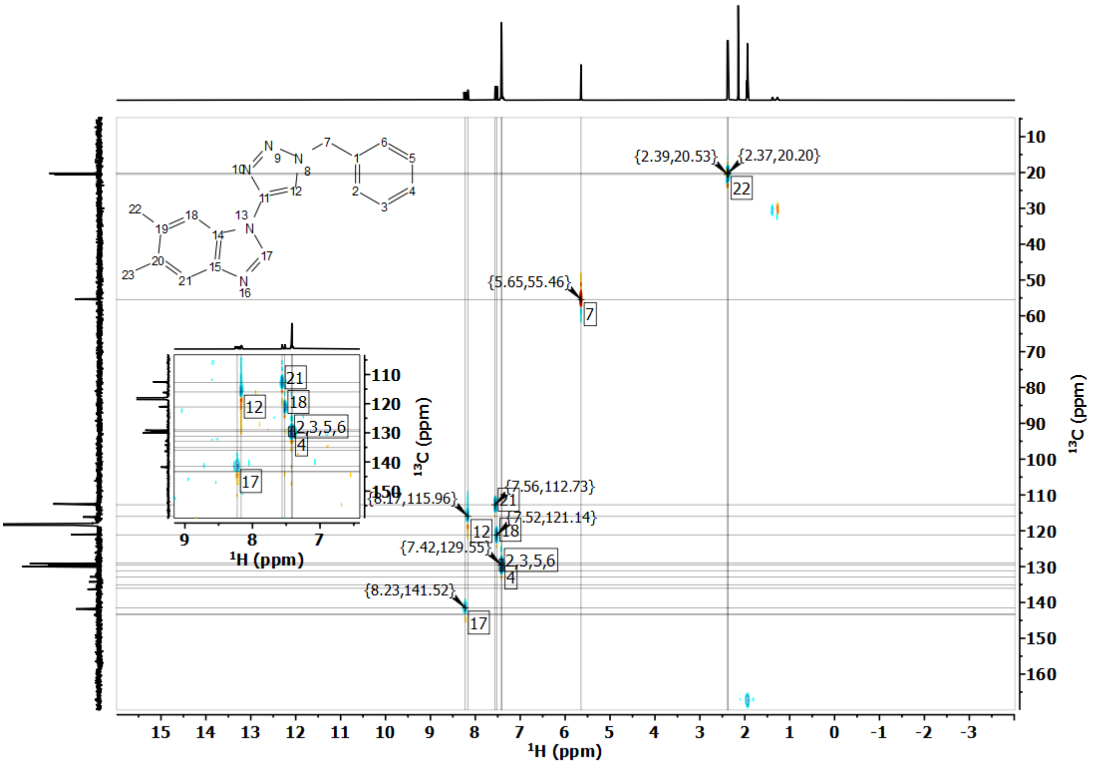

Figure S167. ^{1}H-^{13}C HSQC NMR spectrum of 1-(1-benzyl-1*H*-1,2,3-triazol-4-yl1/3-^{15}N)-5,6-dimethyl-1*H*-benzo[*d*]imidazole-1,3-$^{15}N_2$ **10-^{15}N$_4$.**

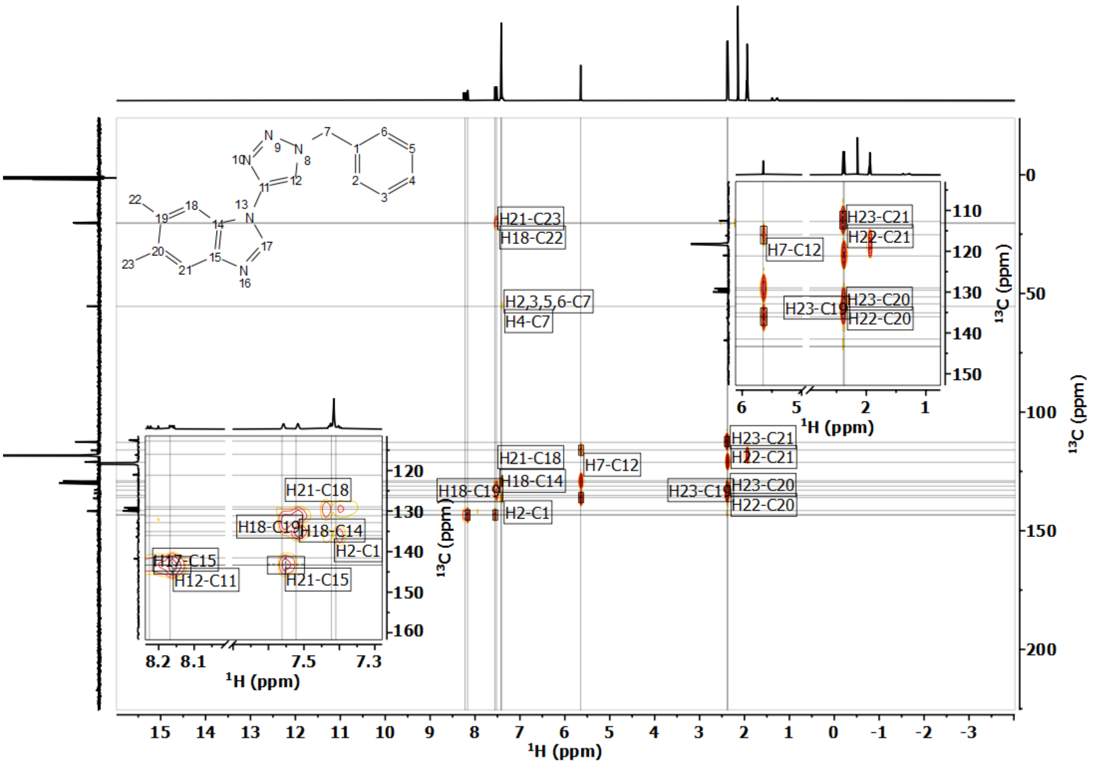

Figure S168. ^{1}H-^{13}C HMBC NMR spectrum of 1-(1-benzyl-1*H*-1,2,3-triazol-4-yl1/3-^{15}*N*)-5,6-dimethyl-1*H*-benzo[*d*]imidazole-1,3-^{15}*N*$_2$ **10-^{15}N$_4$.**

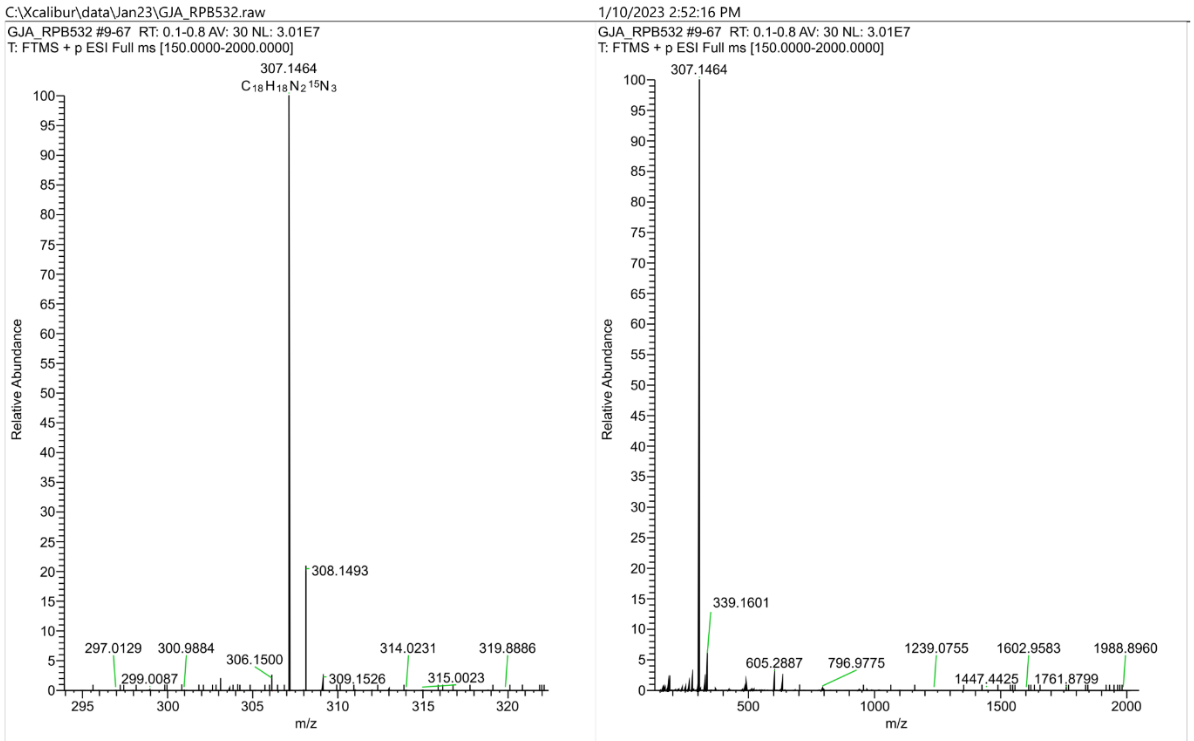

Peak Mass	Display For...	S Fit	RDB	Delta [ppm]	Theo. mass	Rank	Combined S...	# Matched I...	# Missed Iso.	MS Cov. [%]	Pattern Cov....	MSMS Matc...
307.1464	$C_{18}H_{18}N_2{}^{15}N_3$	11.17820921 32234	12.50	-1.21	307.14678	1	0.6	2	2	0.01	16.11	(Collection)

Figure S169. HRMS analysis of 1-(1-benzyl-1*H*-1,2,3-triazol-4-yl1/3-^{15}*N*)-5,6-dimethyl-1*H*-benzo[*d*]imidazole-1,3-^{15}*N*$_2$ **10-^{15}N$_4$.**

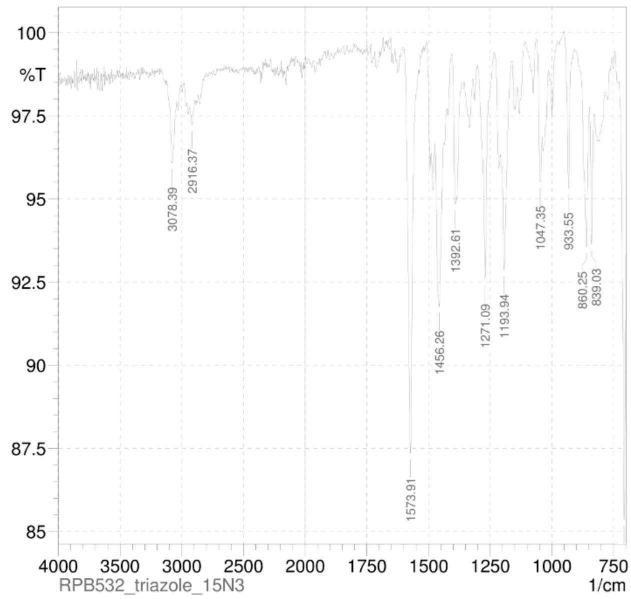

SHIMADZU

No.	Peak	Intensity	Corr. Inte	Base (H)	Base (L)	Area	Corr. Are
1	707.88	85.349	9.606	729.09	698.23	1.036	0.461
2	839.03	93.629	4.025	846.75	829.39	0.317	0.136
3	860.25	93.544	4.567	904.61	848.68	0.669	0.323
4	933.55	95.313	4.203	950.91	920.05	0.262	0.204
5	1047.35	95.516	2.339	1062.78	1039.63	0.274	0.105
6	1193.94	92.863	4.41	1209.37	1163.08	0.775	0.327
7	1271.09	92.568	5.82	1300.02	1255.66	0.715	0.425
8	1392.61	94.842	4.241	1404.18	1365.6	0.535	0.348
9	1456.26	91.765	4.897	1473.62	1438.9	0.934	0.422
10	1573.91	87.345	11.924	1600.92	1541.12	1.301	1.098
11	2916.37	97.227	0.647	2939.52	2895.15	0.475	0.06
12	3078.39	96.078	1.892	3105.39	3041.74	0.819	0.251

Comment;
RPB532_triazole_15N3

Date/Time; 30/01/2023 14:23:48
No. of Scans; 16
Resolution; 4 [1/cm]
Apodization; Happ-Genzel

Figure S170. FT-IR spectrum of 1-(1-benzyl-1*H*-1,2,3-triazol-4-yl1/3-^{15}N)-5,6-dimethyl-1*H*-benzo[*d*]imidazole-1,3-$^{15}N_2$ **10-^{15}N$_4$**.

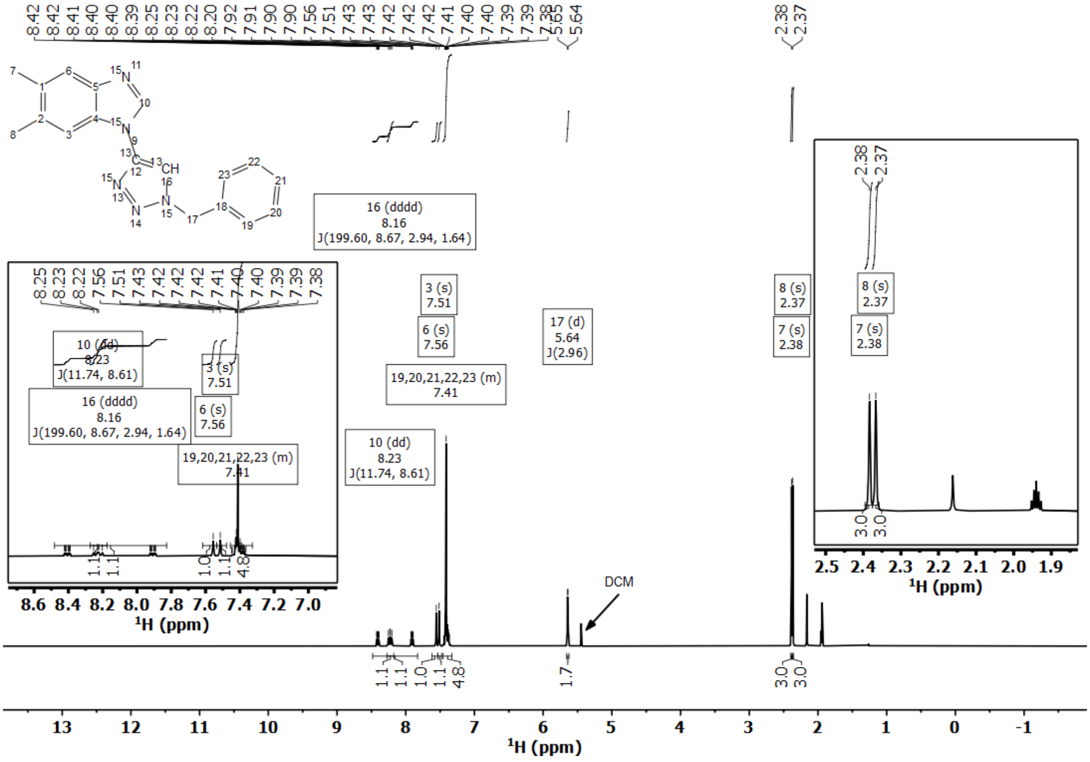

Figure S171. ^{1}H NMR spectrum of 1-(1-benzyl-1*H*-1,2,3-triazol-4-yl-4,5-$^{13}C_2$-1/3-^{15}N)-5,6-dimethyl-1*H*-benzo[*d*]imidazole-1,3-$^{15}N_2$ **10-^{13}C$_2$-^{15}N$_4$**.

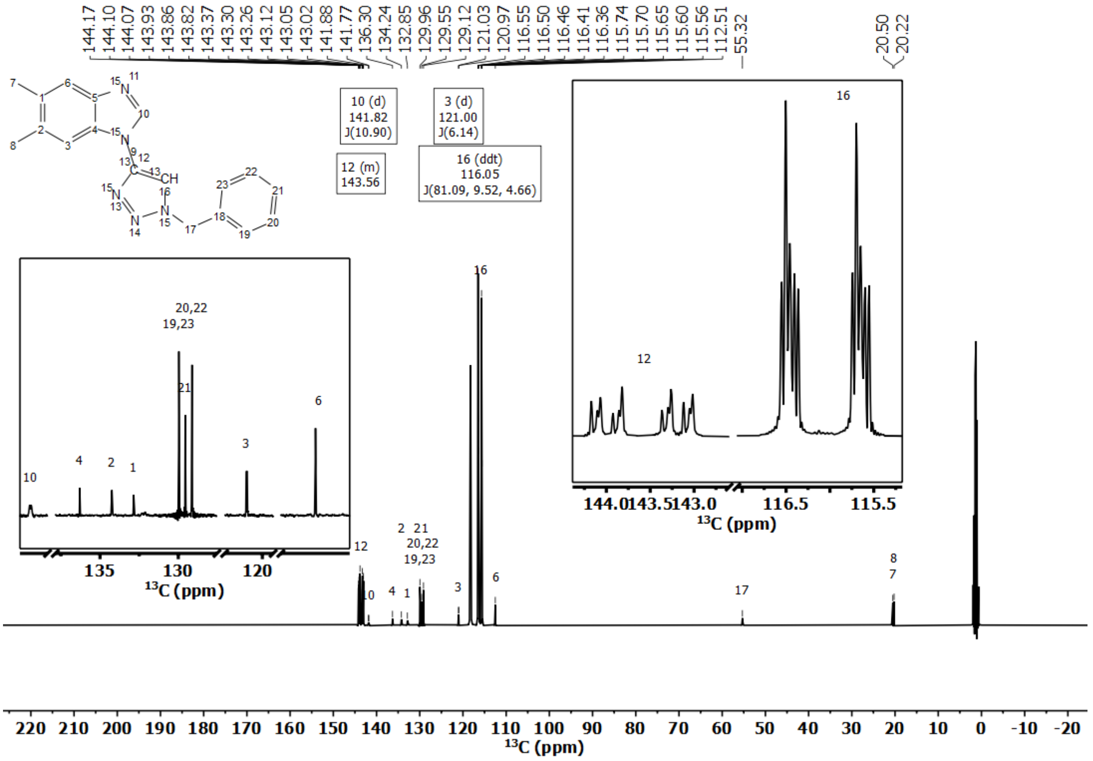

Figure S172. ^{13}C NMR spectrum of 1-(1-benzyl-1*H*-1,2,3-triazol-4-yl-4,5-$^{13}C_2$-1/3-^{15}N)-5,6-dimethyl-1*H*-benzo[*d*]imidazole-1,3-$^{15}N_2$ **10-$^{13}C_2$-$^{15}N_4$**.

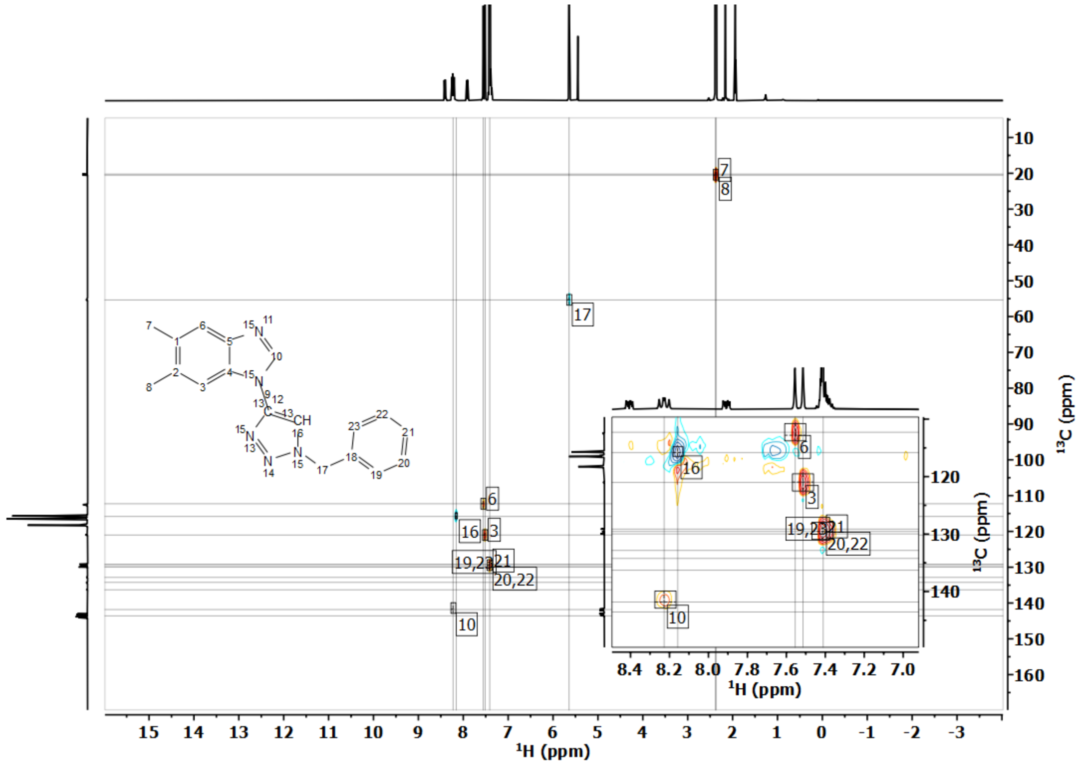

Figure S173. ^{1}H-^{13}C HSQC NMR spectrum of 1-(1-benzyl-1*H*-1,2,3-triazol-4-yl-4,5-$^{13}C_2$-1/3-^{15}N)-5,6-dimethyl-1*H*-benzo[*d*]imidazole-1,3-$^{15}N_2$ **10-$^{13}C_2$-$^{15}N_4$**.

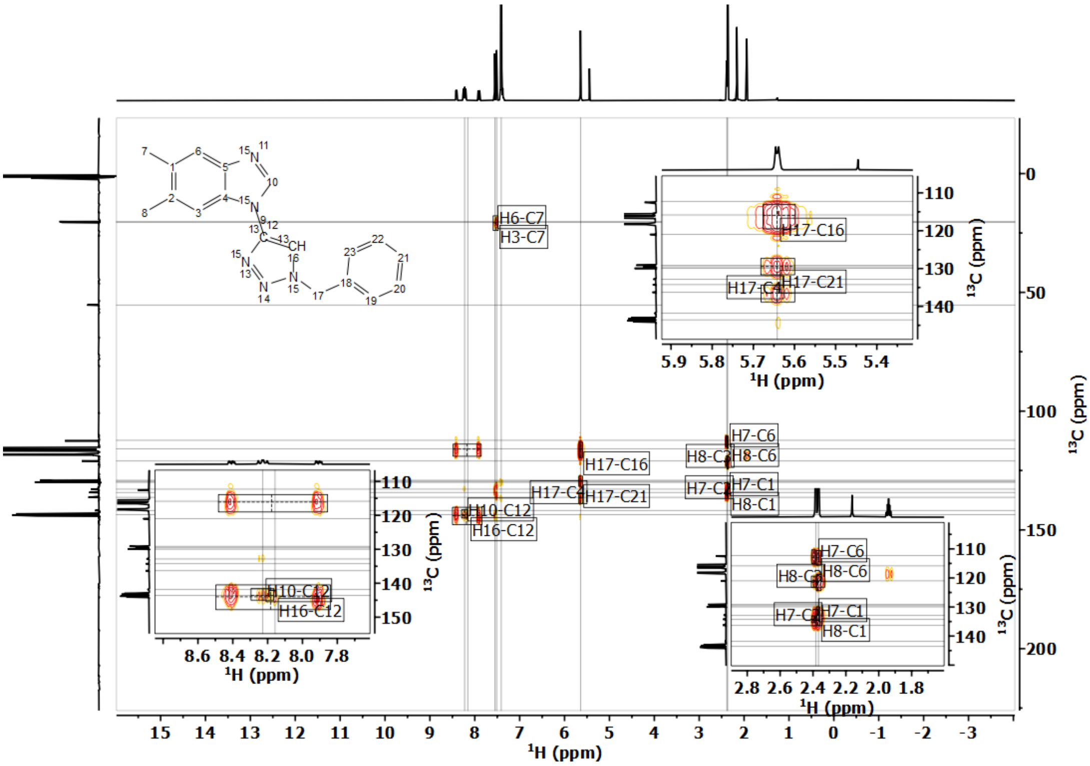

Figure S174. ^1H-^{13}C HMBC NMR spectrum of 1-(1-benzyl-1*H*-1,2,3-triazol-4-yl-4,5-$^{13}C_2$-1/3-^{15}N)-5,6-dimethyl-1*H*-benzo[*d*]imidazole-1,3-$^{15}N_2$ **10-^{13}C$_2$-^{15}N$_4$**.

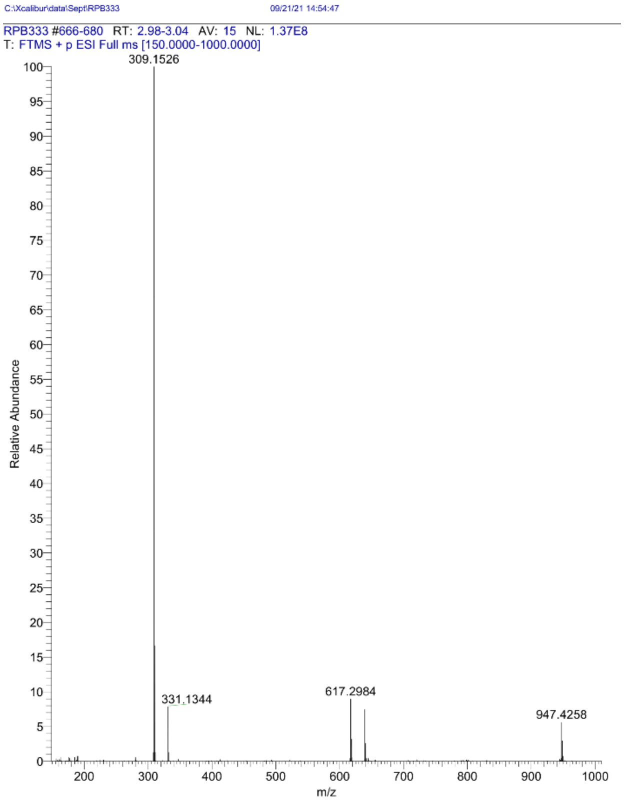

Figure S175. HRMS analysis of 1-(1-benzyl-1*H*-1,2,3-triazol-4-yl-4,5-$^{13}C_2$-1/3-^{15}N)-5,6-dimethyl-1*H*-benzo[*d*]imidazole-1,3-$^{15}N_2$ **10-^{13}C$_2$-^{15}N$_4$**.

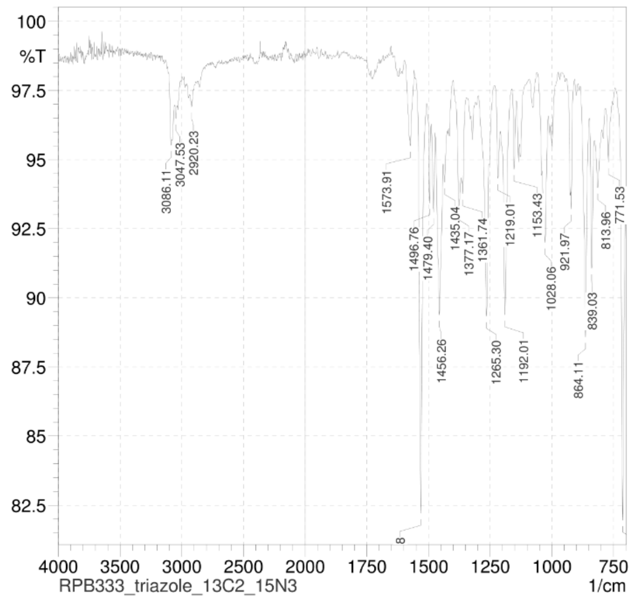

SHIMADZU

No.	Peak	Intensity	Corr. Inte	Base (H)	Base (L)	Area	Corr. Are
1	713.66	81.955	13.067	744.52	700.16	1.452	0.619
2	771.53	94.902	2.062	783.1	756.1	0.45	0.092
3	813.96	93.982	1.889	827.46	798.53	0.635	0.109
4	839.03	91.068	5.013	848.68	829.39	0.526	0.187
5	864.11	88.59	8.31	885.33	850.61	0.954	0.495
6	921.97	93.214	4.647	941.26	910.4	0.516	0.228
7	1028.06	91.995	2.256	1031.92	1016.49	0.412	0.079
8	1153.43	94.651	2.703	1161.15	1143.79	0.293	0.088
9	1192.01	89.385	7.209	1209.37	1163.08	1.127	0.476
10	1219.01	94.323	2.264	1230.58	1211.3	0.359	0.077
11	1265.3	89.329	8.17	1298.09	1232.51	1.464	0.745
12	1361.74	93.76	1.445	1367.53	1348.24	0.38	0.039
13	1377.17	93.255	2.17	1388.75	1369.46	0.473	0.092
14	1435.04	94.173	0.893	1438.9	1425.4	0.297	0.024
15	1456.26	89.38	5.918	1471.69	1440.83	1.081	0.431
16	1479.4	93.093	2.998	1489.05	1473.62	0.368	0.102
17	1496.76	93.43	3.082	1506.41	1490.97	0.311	0.077
18	1531.48	82.203	15.298	1552.7	1508.33	1.435	0.939
19	1573.91	95.502	2.878	1597.06	1558.48	0.5	0.232
20	2920.23	96.909	0.529	2933.73	2904.8	0.36	0.035
21	3047.53	96.488	0.46	3055.24	3037.89	0.252	0.018
22	3086.11	95.518	0.647	3115.04	3080.32	0.46	0.006

Comment;
RPB333_triazole_13C2_15N3

Date/Time; 30/01/2023 14:27:28
No. of Scans; 16
Resolution; 4 [1/cm]
Apodization; Happ-Genzel

Figure S176. FT-IR spectrum of 1-(1-benzyl-1*H*-1,2,3-triazol-4-yl-4,5-$^{13}C_2$-1/3-^{15}N)-5,6-dimethyl-1*H*-benzo[*d*]imidazole-1,3-$^{15}N_2$ **10-$^{13}C_2$-$^{15}N_4$**.

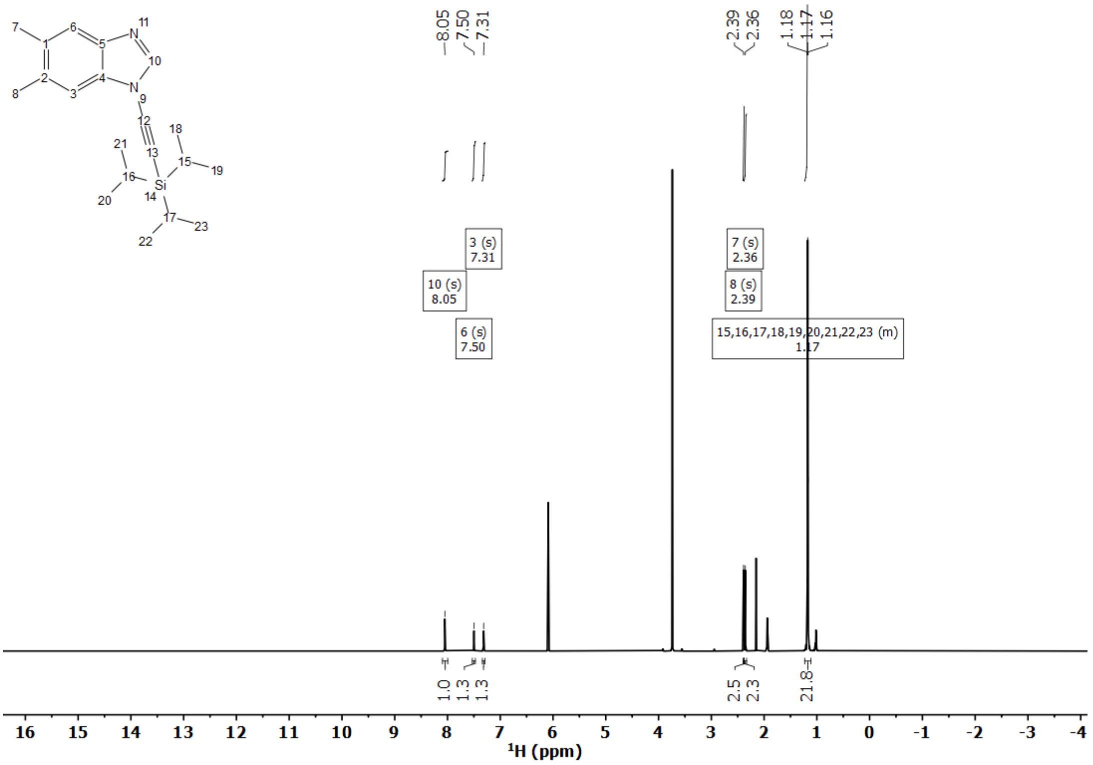

Figure S177. ^{1}H NMR spectrum of 5,6-dimethyl-1-((triisopropylsilyl)ethynyl)-1*H*-benzo[*d*]imidazole **14**.

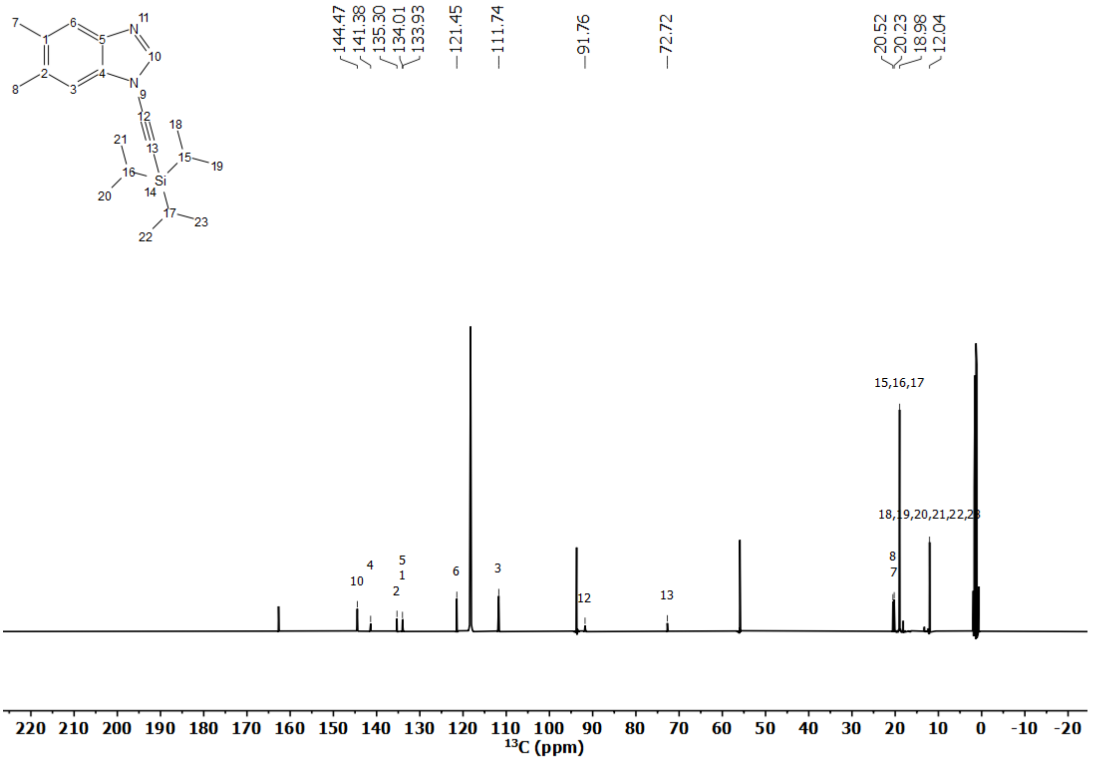

Figure S178. $^{13}C\{^{1}H\}$ NMR spectrum of 5,6-dimethyl-1-((triisopropylsilyl)ethynyl)-1*H*-benzo[*d*]imidazole **14**.

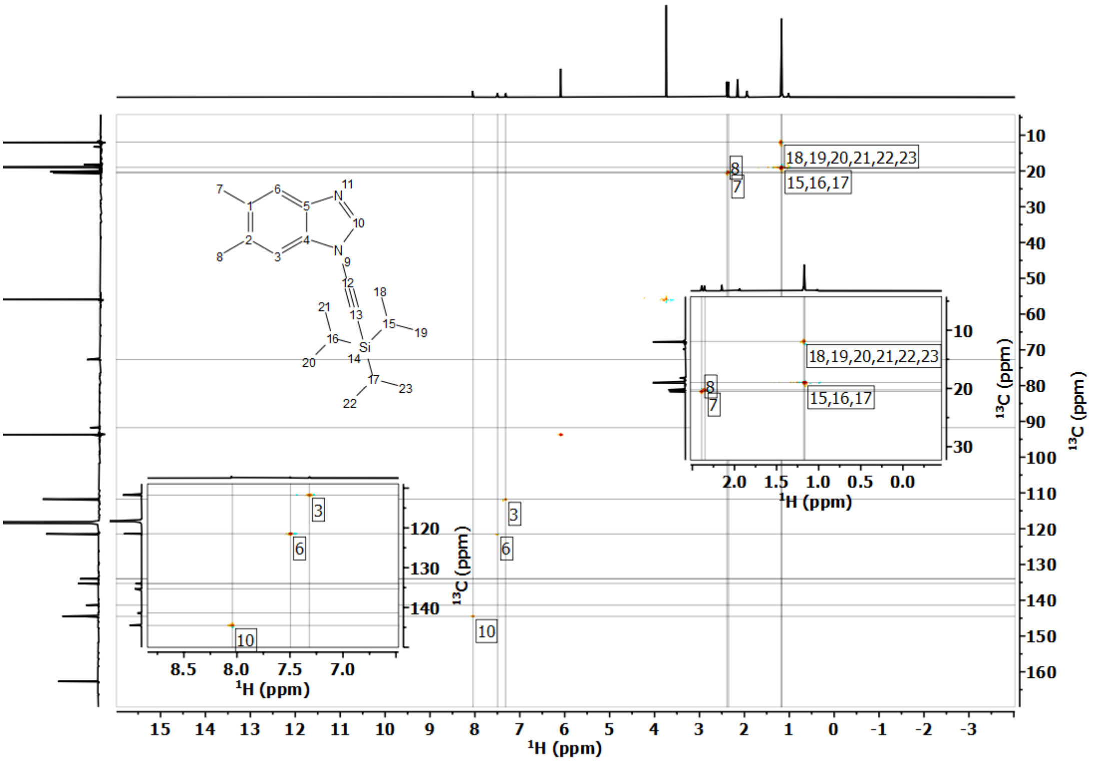

Figure S179. ^{1}H-^{13}C HSQC NMR spectrum of 5,6-dimethyl-1-((triisopropylsilyl)ethynyl)-1*H*-benzo[*d*]imidazole **14**.

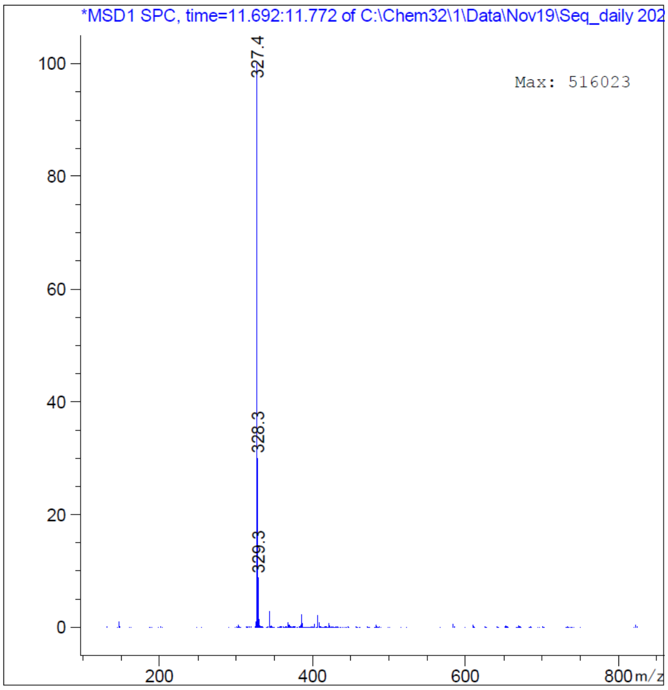

Figure S180. LC-MS analysis of 5,6-dimethyl-1-((triisopropylsilyl)ethynyl)-1*H*-benzo[*d*]imidazole **14**.

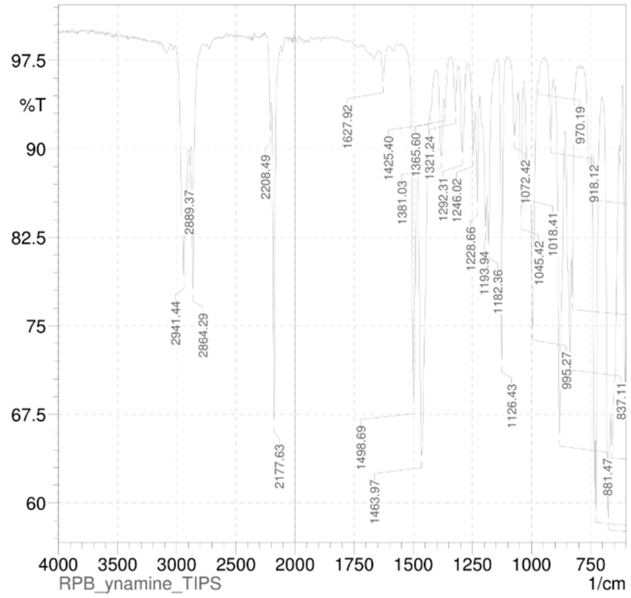

No.	Peak	Intensity	Corr. Inte	Base (H)	Base (L)	Area	Corr. Are
1	603.72	69.029	11.177	613.36	599.86	1.547	0.423
2	624.94	89.793	1.318	626.87	615.29	0.431	0.079
3	659.66	64.809	6.271	665.44	628.79	4.739	0.919
4	675.09	58.719	14.275	702.09	667.37	4.079	0.921
5	727.16	59.369	10.091	731.02	704.02	2.355	0.277
6	754.17	86.711	4.625	777.31	746.45	1.245	0.269
7	827.46	77.41	5.789	831.32	806.25	1.127	0.132
8	837.11	72.288	6.919	842.89	833.25	1.194	0.217
9	881.47	65.837	27.779	906.54	858.32	3.804	2.417
10	918.12	90.687	5.711	943.19	908.47	0.767	0.272
11	970.19	95.626	0.21	972.12	952.84	0.265	0.003
12	995.27	74.835	16.394	1008.77	974.05	2.247	1.016
13	1018.41	86.674	4.376	1031.92	1010.7	0.975	0.177
14	1045.42	84.237	10.979	1055.06	1033.85	0.9	0.456
15	1072.42	91.107	4.798	1099.43	1056.99	0.976	0.285
16	1126.43	72.121	25.546	1141.86	1101.35	1.57	1.16
17	1182.36	81.814	4.586	1186.22	1143.79	1.343	0.138
18	1193.94	83.402	4.585	1205.51	1188.15	1.073	0.164
19	1228.66	85.426	8.352	1238.3	1220.94	0.799	0.315
20	1246.02	89.541	4.491	1269.16	1240.23	0.775	0.164
21	1292.31	89.65	7.496	1307.74	1271.09	0.988	0.531
22	1321.24	93.177	4.159	1340.53	1309.67	0.556	0.203
23	1365.6	93.428	1.326	1369.46	1342.46	0.464	0.041
24	1381.03	89.354	5.67	1400.32	1371.39	0.942	0.345
25	1425.4	93.134	0.594	1429.25	1402.25	0.567	0.014
26	1463.97	64.016	27.727	1483.26	1429.25	5.729	3.801
27	1498.69	68.592	24.01	1543.05	1485.19	2.814	1.616
28	1627.92	95.768	2.743	1647.21	1608.63	0.416	0.165
29	2177.63	67.027	28.013	2196.92	2119.77	3.866	2.617
30	2208.49	91.526	3.852	2235.5	2198.85	0.742	0.194
31	2864.29	78.122	13.163	2877.79	2781.35	2.916	0.961
32	2889.37	88.552	1.507	2900.94	2879.72	1.04	0.075
33	2941.44	79.161	13.947	3008.95	2902.87	5.483	2.729

Comment;
RPB_ynamine_TIPS

Date/Time; 12/03/2021 12:59:35
No. of Scans; 16

Resolution; 4 [1/cm]
Apodization; Happ-Genzel

Figure S181. FT-IR spectrum of 5,6-dimethyl-1-((triisopropylsilyl)ethynyl)-1*H*-benzo[*d*]imidazole **14**.

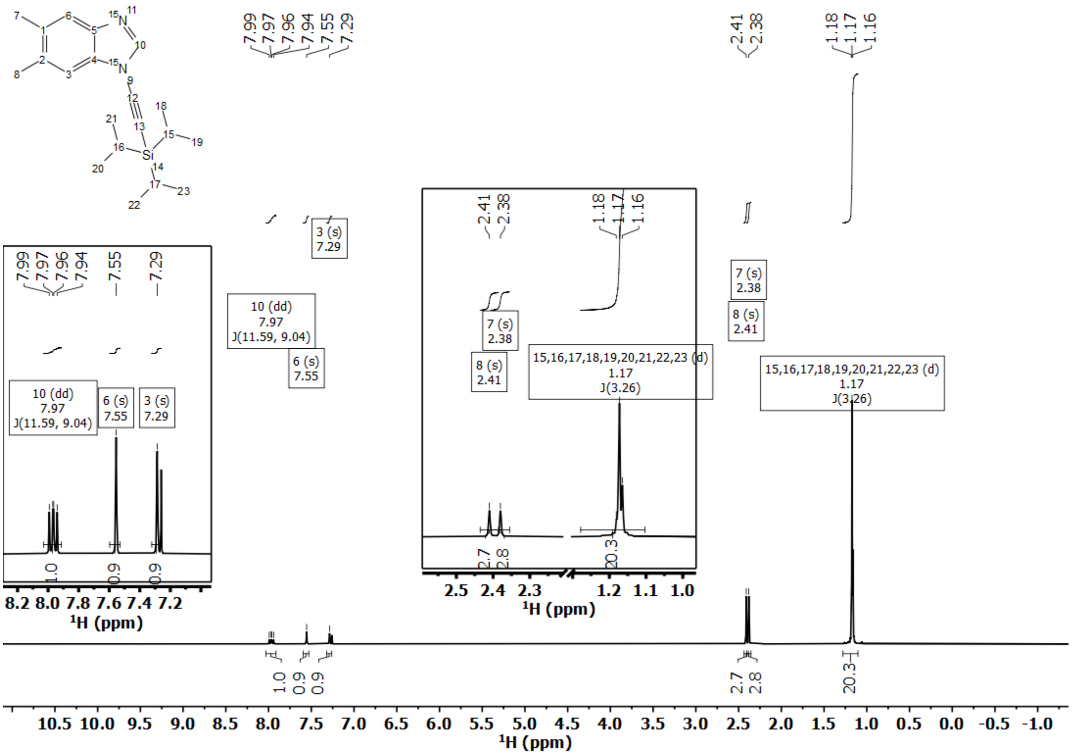

Figure S182. ^{1}H NMR spectrum of 5,6-dimethyl-1-((triisopropylsilyl)ethynyl)-1*H*-benzo[*d*]imidazole-1,3-$^{15}N_2$ **14-^{15}N$_2$**.

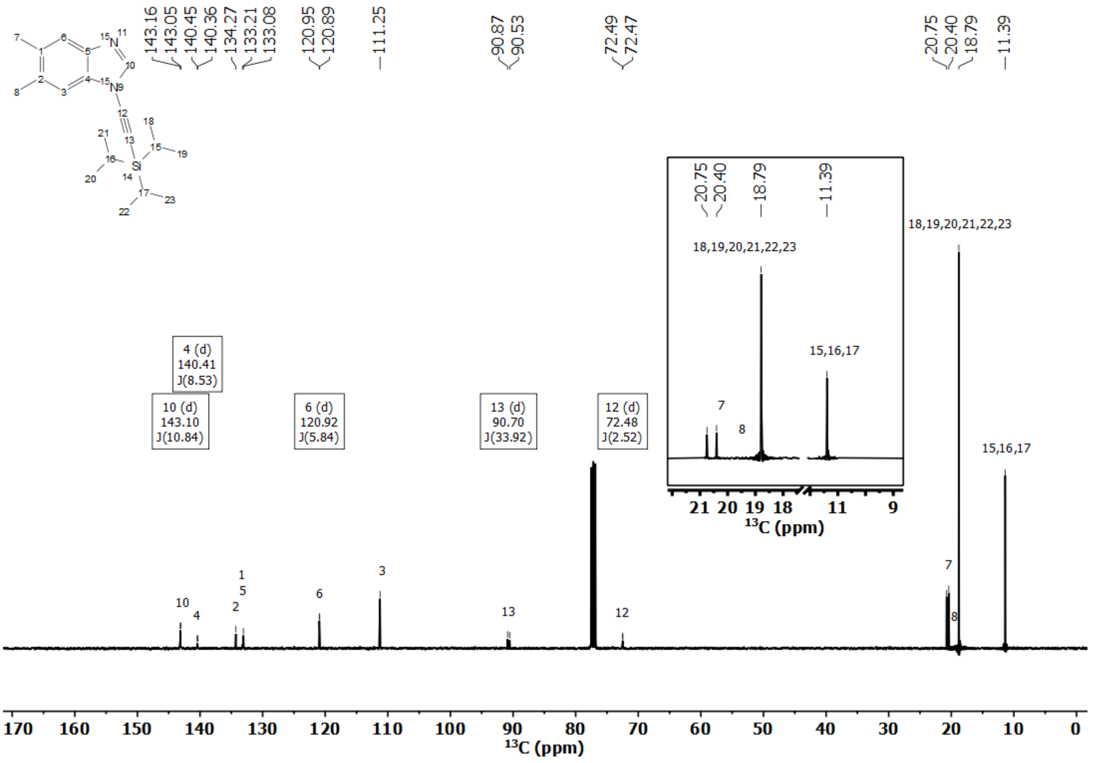

Figure S183. ^{13}C{^{1}H} NMR spectrum of 5,6-dimethyl-1-((triisopropylsilyl)ethynyl)-1*H*-benzo[*d*]imidazole-1,3-$^{15}N_2$ **14-^{15}N$_2$**.

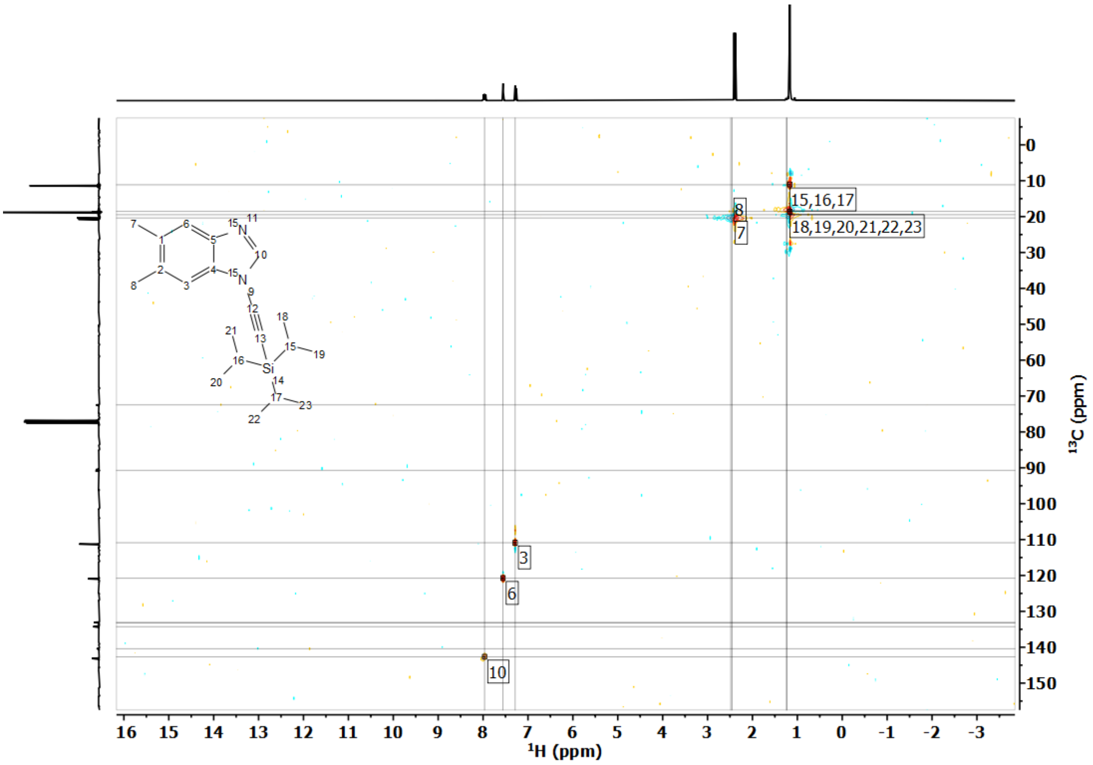

Figure S184. ^{1}H-^{13}C HSQC NMR spectrum of 5,6-dimethyl-1-((triisopropylsilyl)ethynyl)-1*H*-benzo[*d*]imidazole-1,3-^{15}*N*$_2$ **14-^{15}N$_2$**.

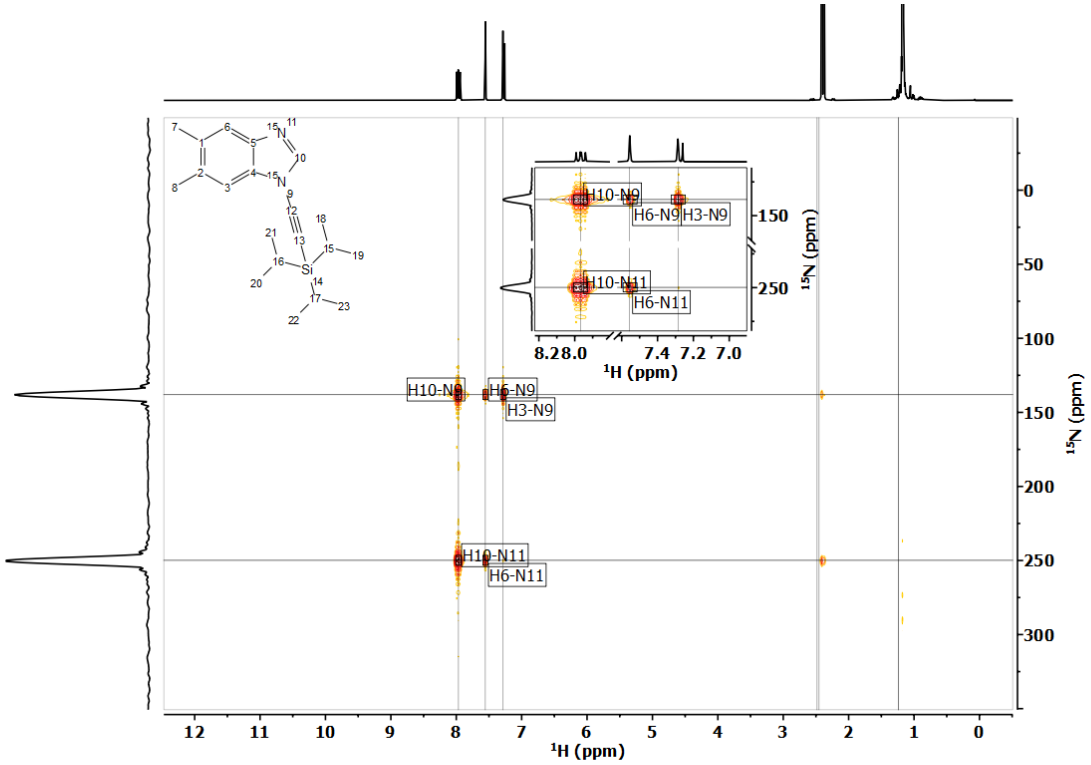

Figure S185. ^{1}H-^{15}N HMBC NMR spectrum of 5,6-dimethyl-1-((triisopropylsilyl)ethynyl)-1*H*-benzo[*d*]imidazole-1,3-^{15}*N*$_2$ **14-^{15}N$_2$**.

Display Report

Analysis Info

Analysis Name D:\Data\Alans Data May 2021\STRA_Emma Campbell R3 162.d
Method 010713alanstune_low.m
Sample Name Emma Campbell R3 162
Comment Emma Campbell R3 162

Acquisition Date 5/18/2021 4:48:03 PM
Operator Bruker UK
Instrument micrOTOF 8213750.10408

Acquisition Parameter

Source Type	ESI	Ion Polarity	Positive	Set Nebulizer	1.0 Bar
Focus	Active			Set Dry Heater	230 °C
Scan Begin	40 m/z	Set Capillary	4000 V	Set Dry Gas	6.0 l/min
Scan End	3000 m/z	Set End Plate Offset	-500 V	Set Divert Valve	Waste

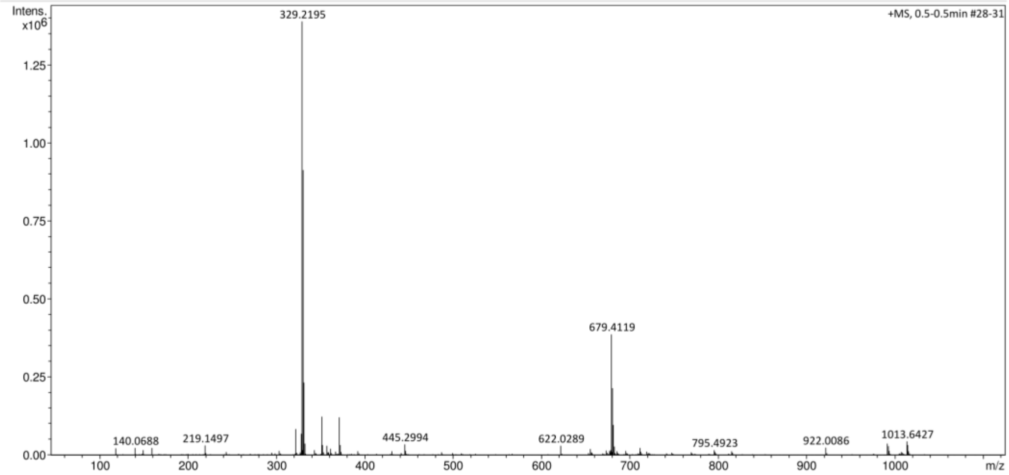

Bruker Compass DataAnalysis 4.1 printed: 5/18/2021 4:52:24 PM by: Bruker UK 1 of 1

Figure S186. HRMS analysis of 5,6-dimethyl-1-((triisopropylsilyl)ethynyl)-1*H*-benzo[*d*]imidazole-1,3-$^{15}N_2$ **14-^{15}N$_2$.**

SHIMADZU

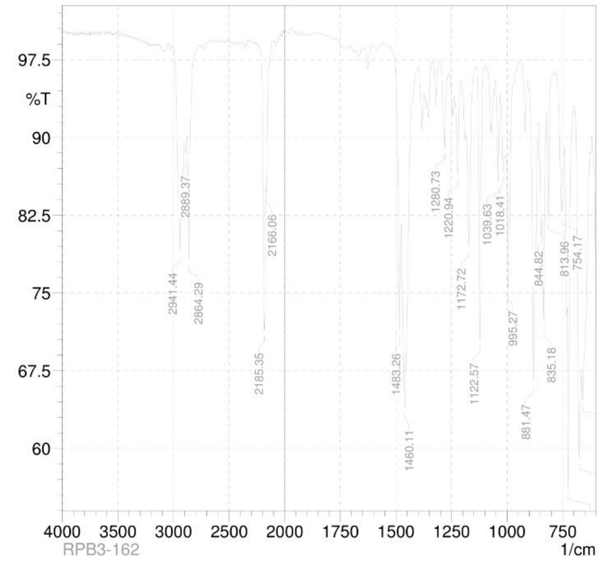

No.	Peak	Intensity	Corr. Inte	Base (H)	Base (L)	Area	Corr. Are
1	659.66	64.665	6.098	665.44	626.87	4.916	0.76
2	675.09	59.037	14.295	698.23	667.37	4.042	0.955
3	725.23	56.19	34.052	740.67	700.16	4.196	2.51
4	754.17	82.742	8.314	783.1	742.59	1.944	0.586
5	813.96	82.164	11.416	821.68	798.53	0.963	0.383
6	835.18	71.919	13.384	840.96	823.6	1.511	0.502
7	844.82	81.218	2.136	858.32	842.89	1.126	0.137
8	881.47	66.444	26.661	906.54	860.25	3.7	2.278
9	995.27	75.456	15.472	1008.77	974.05	2.24	0.951
10	1018.41	86.641	3.989	1029.99	1010.7	0.962	0.163
11	1039.63	85.781	8.843	1053.13	1031.92	0.88	0.383
12	1122.57	70.396	26.451	1138	1097.5	1.895	1.335
13	1172.72	79.539	12.302	1182.36	1143.79	1.85	0.762
14	1220.94	86.413	8.381	1236.37	1207.44	1.068	0.396
15	1280.73	88.951	7.783	1301.95	1265.3	1.012	0.494
16	1460.11	63.986	21.335	1475.54	1427.32	5.463	2.675
17	1483.26	71.342	12.485	1525.69	1477.47	2.127	0.469
18	2166.06	84.751	1.087	2167.99	2139.06	1.072	0.074
19	2185.35	71.39	16.834	2239.36	2169.92	3.437	1.314
20	2864.29	78.181	12.981	2877.79	2775.57	3.008	0.974
21	2889.37	88.465	1.309	2899.01	2879.72	0.965	0.065
22	2941.44	79.12	13.895	3007.02	2900.94	5.719	2.849

Comment;
RPB3-162

Date/Time; 12/03/2021 13:16:35
No. of Scans; 16
Resolution; 4 [1/cm]
Apodization; Happ-Genzel

Figure S187. FT-IR spectrum of 5,6-dimethyl-1-((triisopropylsilyl)ethynyl)-1*H*-benzo[*d*]imidazole-1,3-$^{15}N_2$ **14-^{15}N$_2$.**

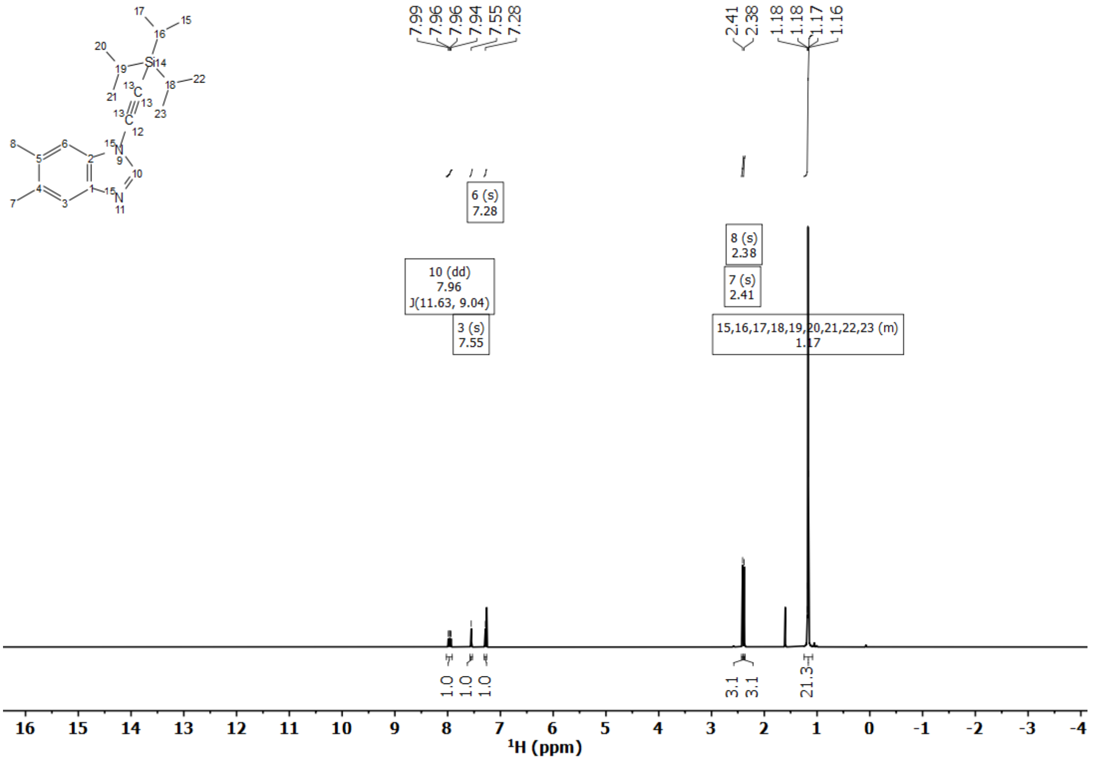

Figure S188. ^{1}H NMR spectrum of 5,6-dimethyl-1-((triisopropylsilyl)ethynyl-1,2-$^{13}C_2$)-1*H*-benzo[*d*]imidazole-1,3-$^{15}N_2$ **14-$^{13}C_2$-$^{15}N_2$**.

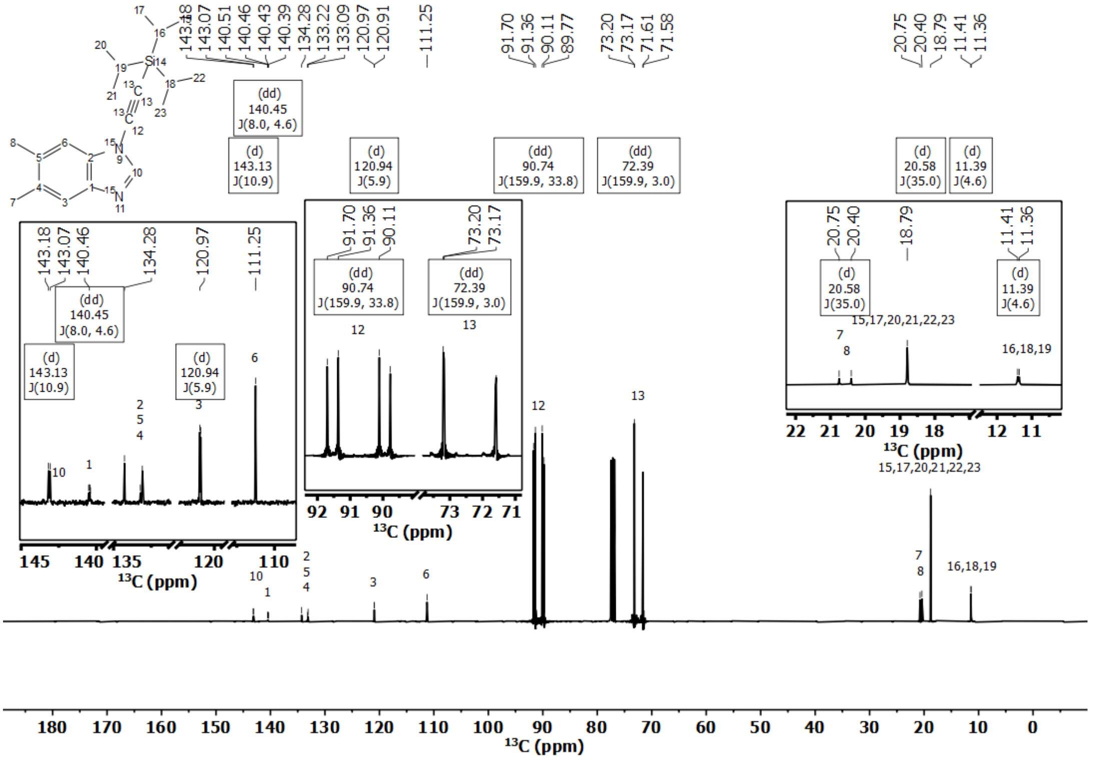

Figure S189. $^{13}C\{^{1}H\}$ NMR spectrum of 5,6-dimethyl-1-((triisopropylsilyl)ethynyl-1,2-$^{13}C_2$)-1*H*-benzo[*d*]imidazole-1,3-$^{15}N_2$ **14-$^{13}C_2$-$^{15}N_2$**.

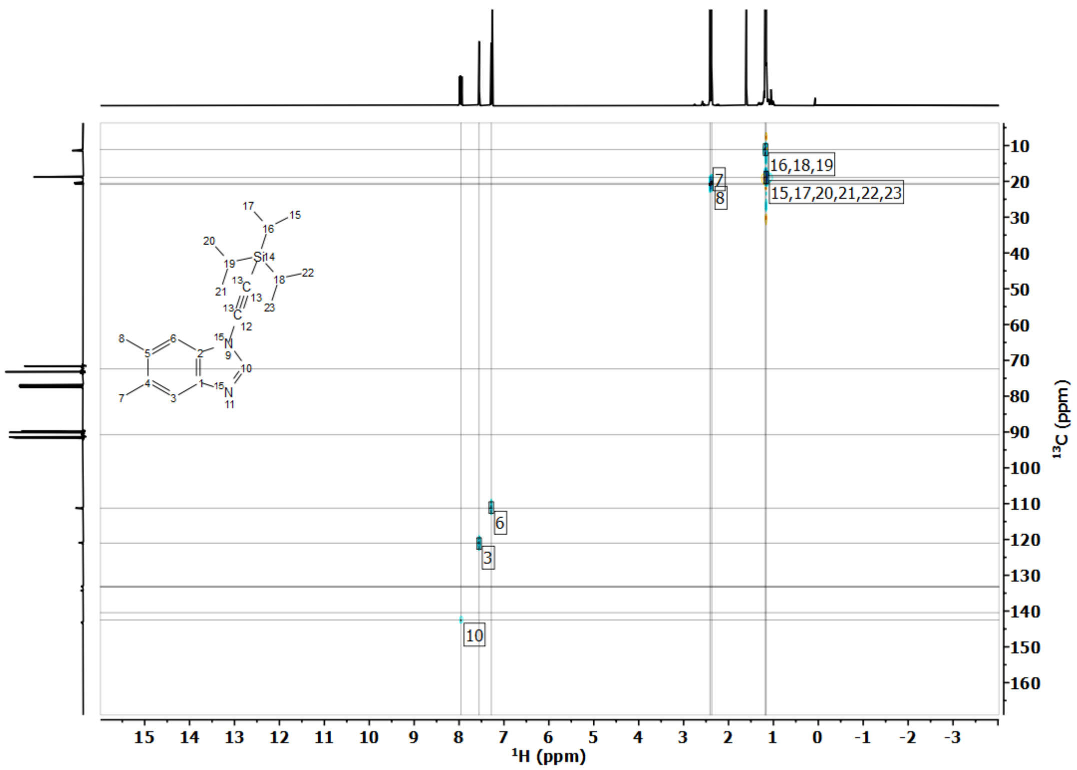

Figure S190. ^{1}H-^{13}C HSQC NMR spectrum of 5,6-dimethyl-1-((triisopropylsilyl)ethynyl-1,2-$^{13}C_2$)-1*H*-benzo[*d*]imidazole-1,3-$^{15}N_2$ **14-$^{13}C_2$-$^{15}N_2$.**

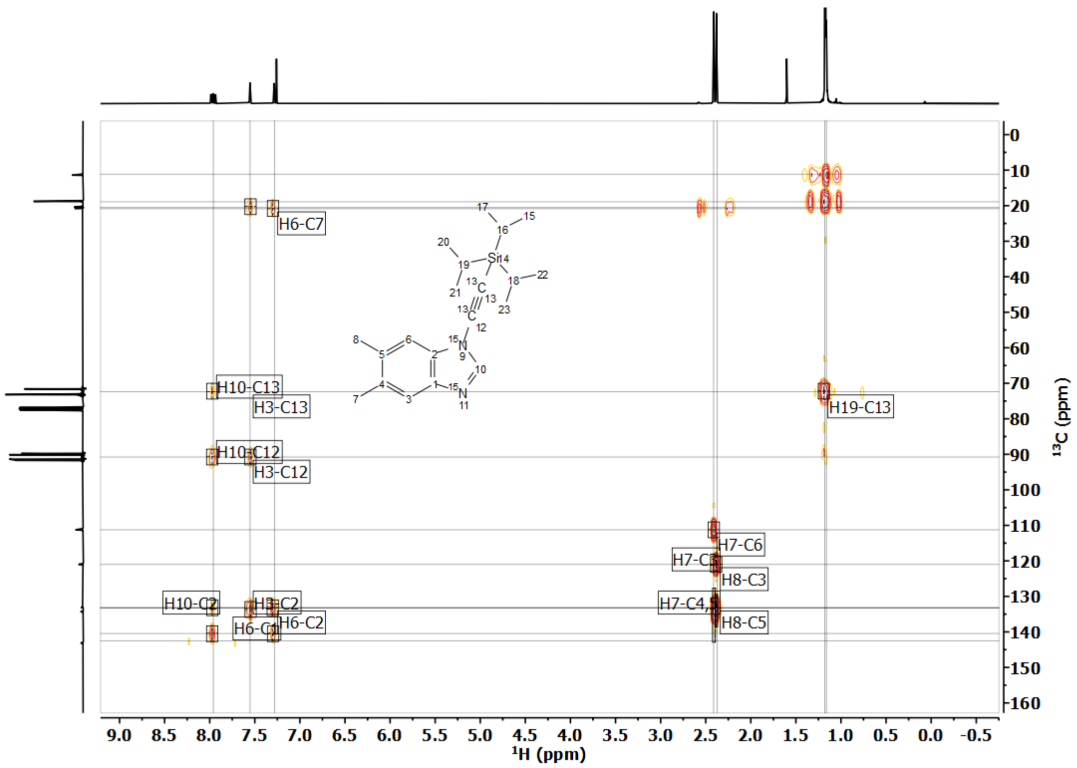

Figure S191. ^{1}H-^{13}C HMBC NMR spectrum of 5,6-dimethyl-1-((triisopropylsilyl)ethynyl-1,2-$^{13}C_2$)-1*H*-benzo[*d*]imidazole-1,3-$^{15}N_2$ **14-$^{13}C_2$-$^{15}N_2$.**

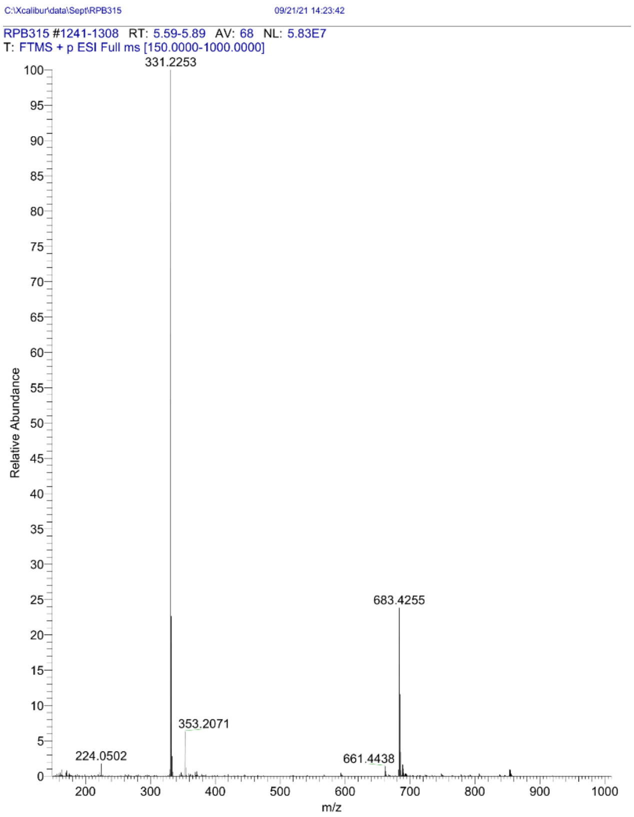

Figure S192. HRMS analysis of 5,6-dimethyl-1-((triisopropylsilyl)ethynyl-1,2-$^{13}C_2$)-1*H*-benzo[*d*]imidazole-1,3-$^{15}N_2$ **14-^{13}C$_2$-^{15}N$_2$.**

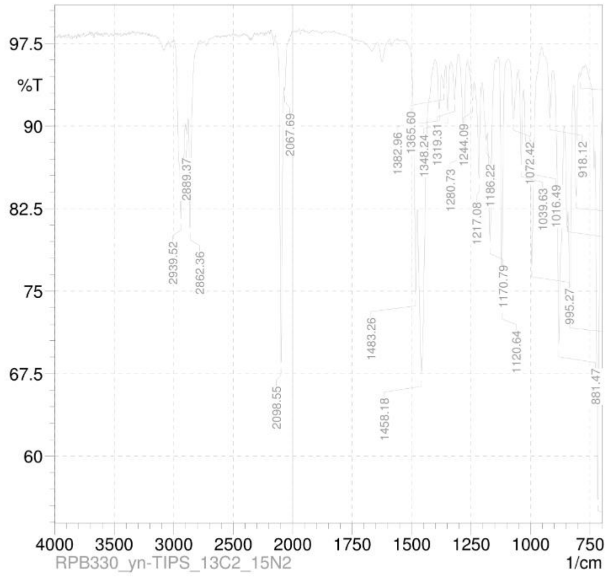

No.	Peak	Intensity	Corr. Inte	Base (H)	Base (L)	Area	Corr. Are
1	717.52	56.048	33	729.09	698.23	3.65	2.174
2	790.81	94.5	0.498	794.67	779.24	0.345	0.014
3	808.17	83.589	10.578	817.82	796.6	0.93	0.387
4	835.18	72.706	12.356	840.96	819.75	1.651	0.503
5	844.82	81.448	1.777	858.32	842.89	1.15	0.116
6	881.47	70.075	22.147	906.54	860.25	3.529	1.922
7	918.12	90.724	4.78	952.84	908.47	1.035	0.261
8	995.27	77.365	13.019	1008.77	954.76	2.521	0.797
9	1016.49	86.766	2.97	1028.06	1010.7	0.92	0.146
10	1039.63	86.47	7.635	1051.2	1029.99	0.853	0.293
11	1072.42	90.672	4.891	1093.64	1053.13	1.151	0.352
12	1120.64	73.517	22.664	1134.14	1095.57	1.739	1.082
13	1170.79	79.467	11.779	1180.44	1149.57	1.72	0.703
14	1186.22	88.638	1.57	1203.58	1182.36	0.905	0.082
15	1217.08	85.193	8.745	1236.37	1205.51	1.293	0.455
16	1244.09	92.321	2.08	1263.37	1238.3	0.671	0.097
17	1280.73	88.217	7.988	1301.95	1265.3	1.108	0.496
18	1319.31	92.382	3.804	1334.74	1303.88	0.723	0.205
19	1348.24	91.351	4.213	1355.96	1336.67	0.534	0.16
20	1365.6	93.45	1.65	1375.25	1357.89	0.438	0.059
21	1382.96	91.547	3.293	1400.32	1377.17	0.644	0.147
22	1458.18	67.387	18.267	1475.54	1402.25	5.633	2.133
23	1483.26	74.701	9.161	1529.55	1477.47	2.043	0.309
24	2067.69	93.26	0.823	2071.55	2040.69	0.587	0.038
25	2098.55	68.456	26.73	2135.2	2073.48	3.833	2.594
26	2862.36	80.708	11.335	2877.79	2806.43	2.757	0.899
27	2889.37	89.221	1.22	2899.01	2879.72	0.899	0.06
28	2939.52	81.622	11.704	2995.45	2900.94	4.873	2.278

Comment;
RPB330_yn-TIPS_13C2_15N2

Date/Time; 30/01/2023 13:55:12
No. of Scans; 16
Resolution; 4 [1/cm]
Apodization; Happ-Genzel

Figure S193. FT-IR spectrum of 5,6-dimethyl-1-((triisopropylsilyl)ethynyl-1,2-$^{13}C_2$)-1*H*-benzo[*d*]imidazole-1,3-$^{15}N_2$ **14-^{13}C$_2$-^{15}N$_2$.**

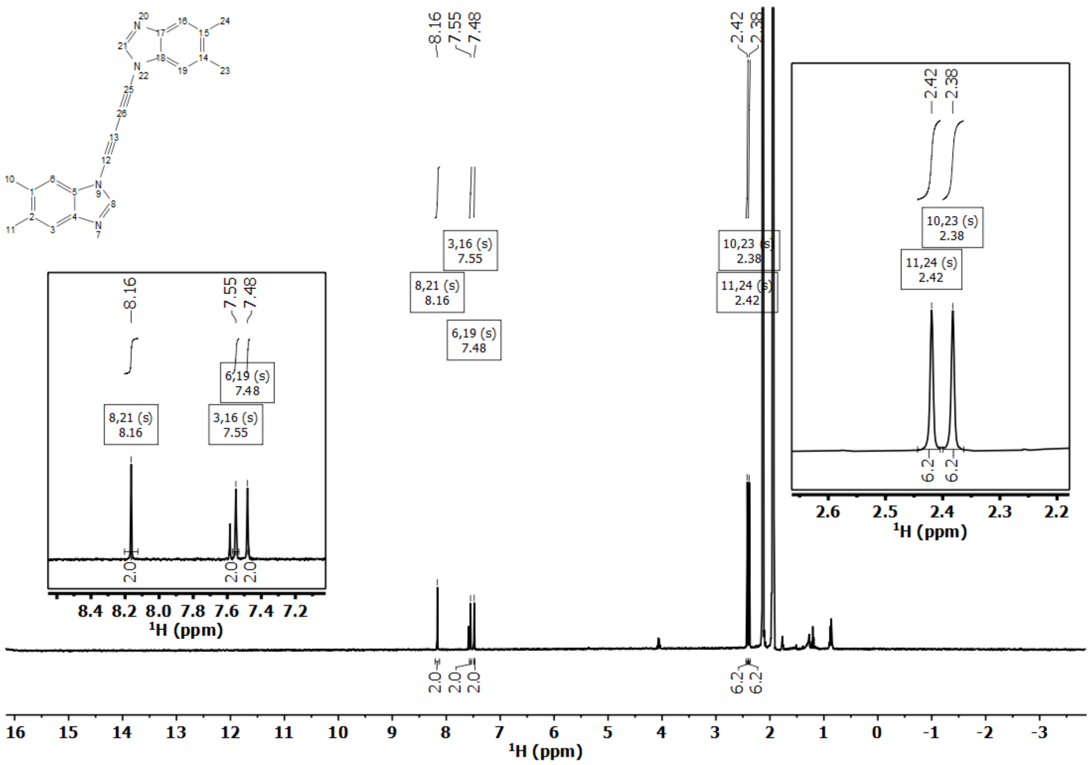

Figure S194. ^{1}H NMR spectrum of 1,4-bis(5,6-dimethyl-1*H*-benzo[*d*]imidazol-1-yl)buta-1,3-diyne **15**.

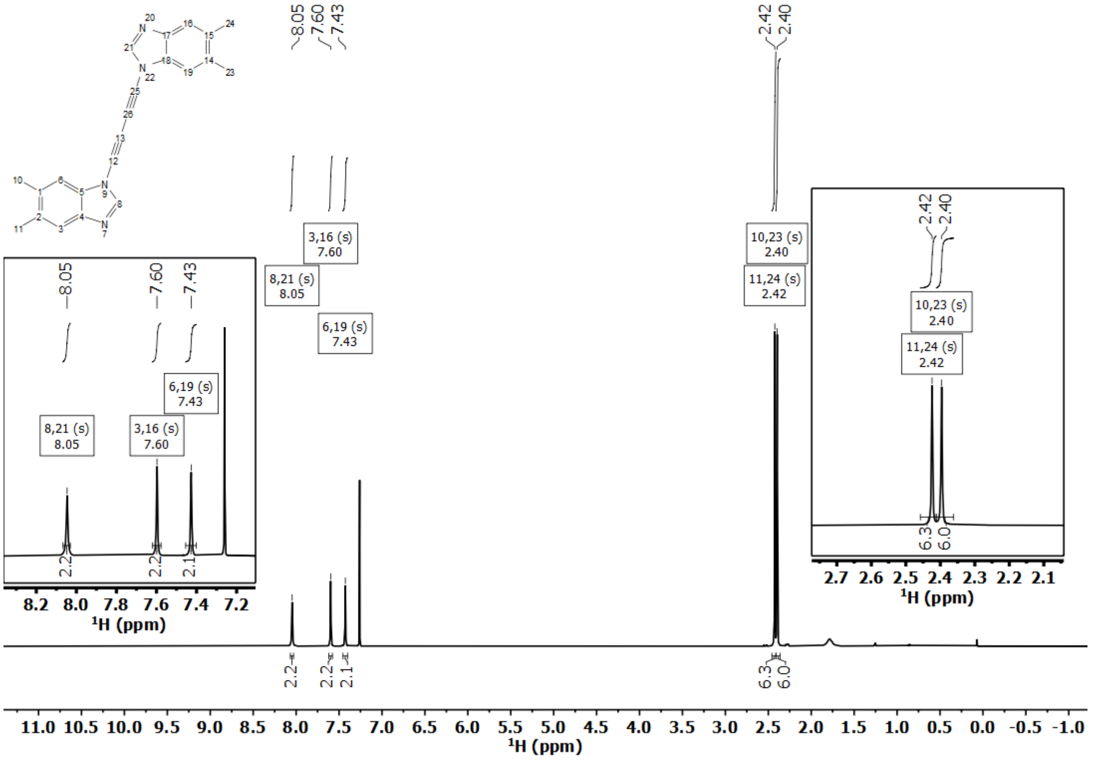

Figure S195. ^{1}H NMR spectrum of 1,4-bis(5,6-dimethyl-1*H*-benzo[*d*]imidazol-1-yl)buta-1,3-diyne **15**.

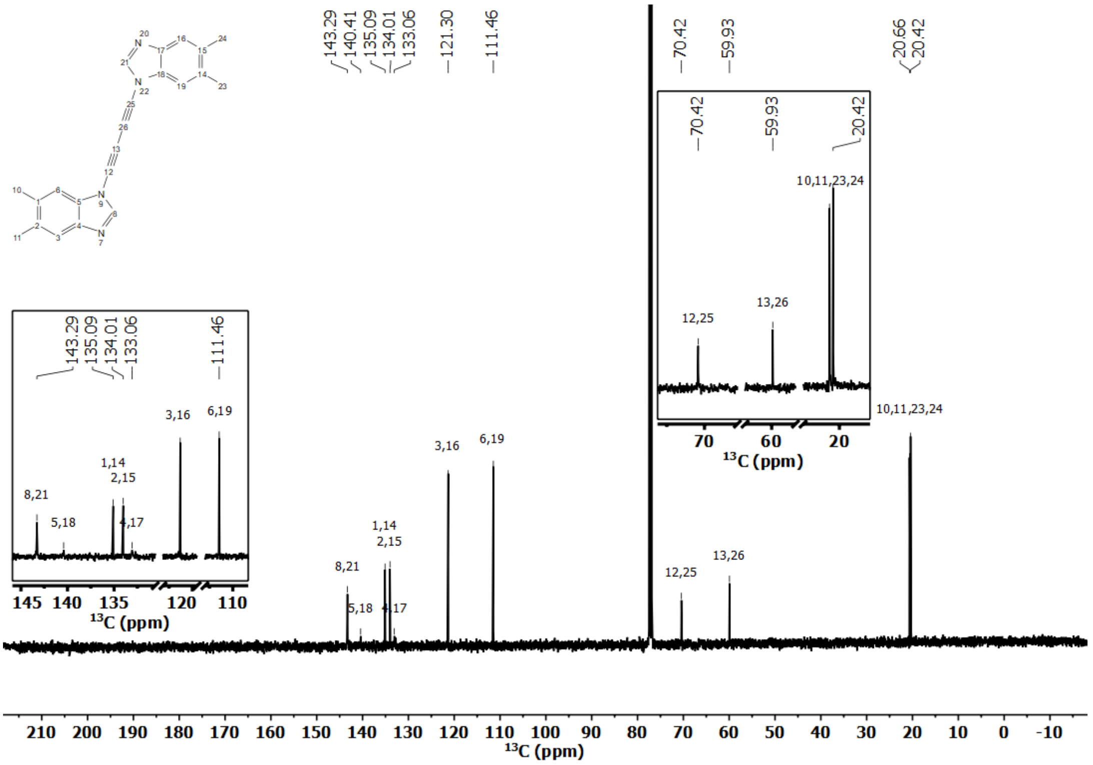

Figure S196. ^{13}C{^{1}H} NMR spectrum of 1,4-bis(5,6-dimethyl-1*H*-benzo[*d*]imidazol-1-yl)buta-1,3-diyne **15**.

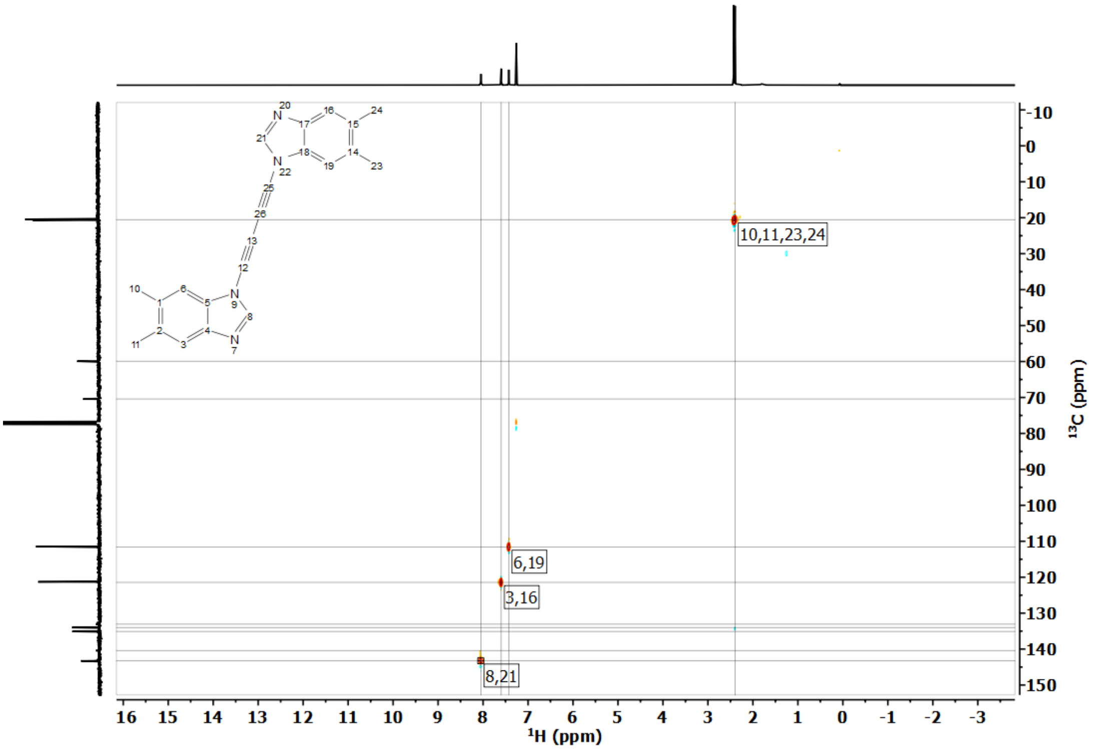

Figure S197. ^{1}H-^{13}C HSQC NMR spectrum of 1,4-bis(5,6-dimethyl-1*H*-benzo[*d*]imidazol-1-yl)buta-1,3-diyne **15**.

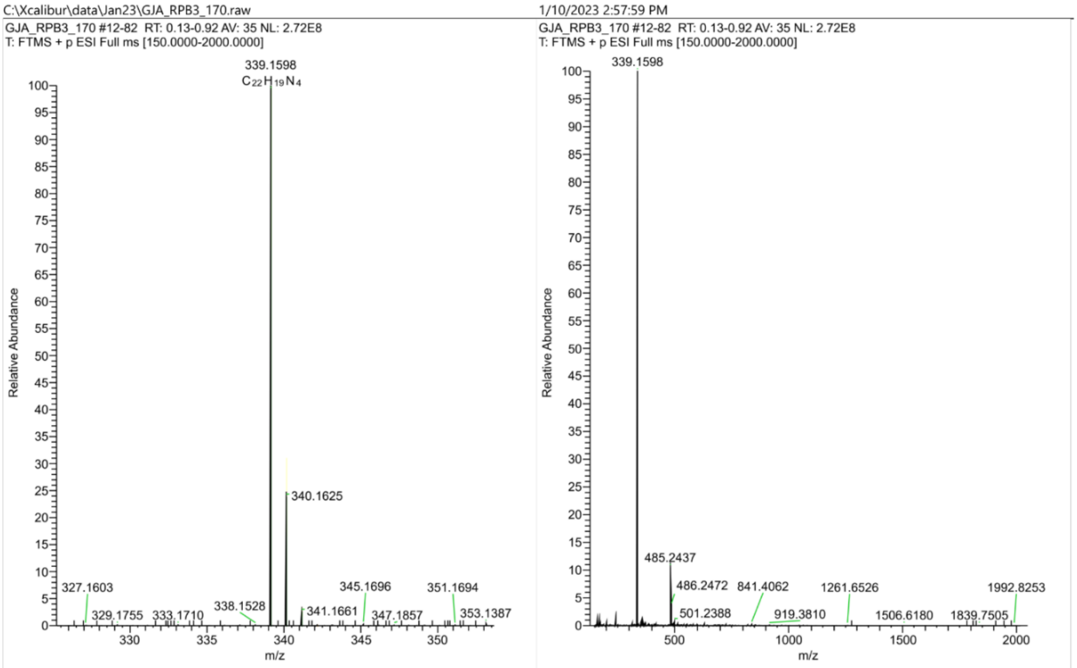

Peak Mass	Display For...	S Fit	RDB	Delta [ppm]	Theo. mass	Rank	Combined S...	# Matched I...	# Missed Iso.	MS Cov. [%]	Pattern Cov....	MSMS Matc...
339.1598	$C_{22}H_{19}N_4$	38.25226613 15241	15.50	-1.78	339.16042	1	96.62	4	0	99.86	99.85	(Collection)

Figure S198. HRMS analysis of 1,4-bis(5,6-dimethyl-1*H*-benzo[*d*]imidazol-1-yl)buta-1,3-diyne **15**.

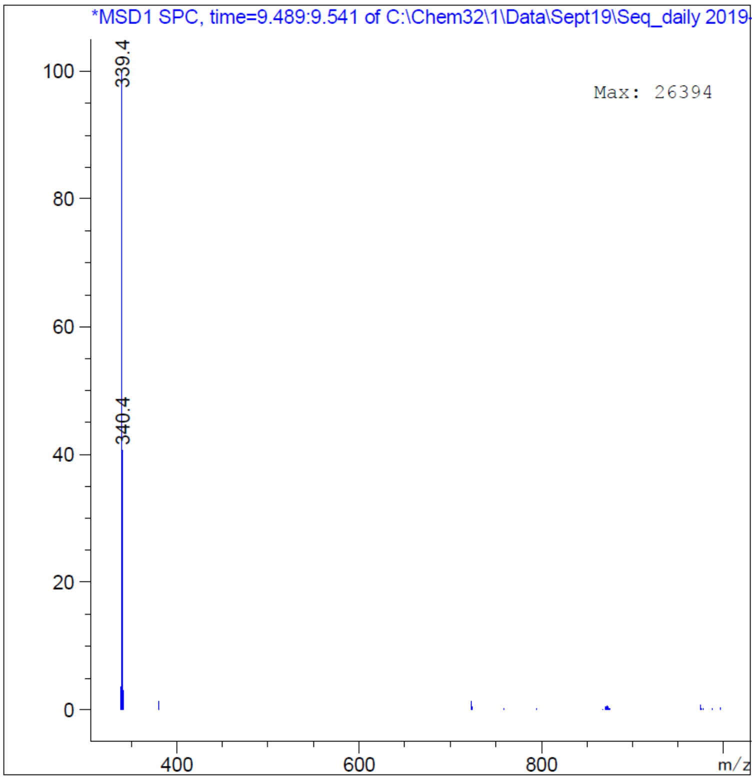

Figure S199. LC-MS analysis of 1,4-bis(5,6-dimethyl-1*H*-benzo[*d*]imidazol-1-yl)buta-1,3-diyne **15**.

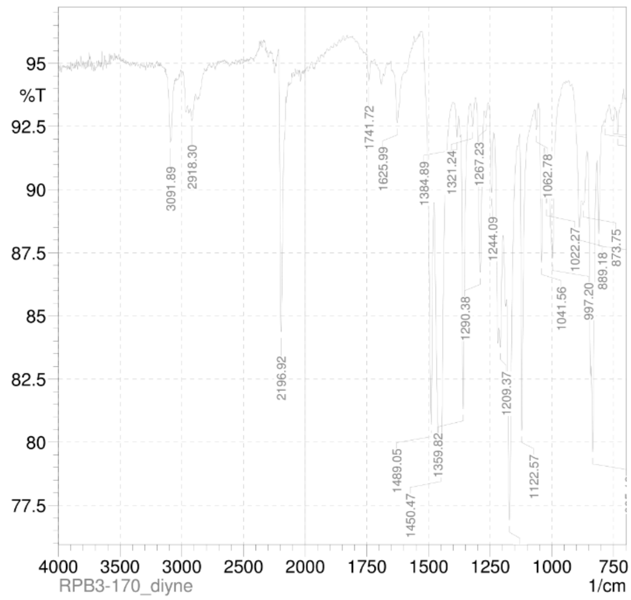

SHIMADZU

No.	Peak	Intensity	Corr. Inte	Base (H)	Base (L)	Area	Corr. Are
1	732.95	92.238	1.097	744.52	719.45	0.792	0.042
2	750.31	92.686	0.599	765.74	744.52	0.678	0.039
3	785.03	92.648	0.2	788.89	777.31	0.372	0.004
4	810.1	88.246	3.079	815.89	788.89	1.083	0.103
5	835.18	79.614	5.436	840.96	817.82	1.554	0.213
6	873.75	89.396	0.687	879.54	858.32	0.963	0.054
7	889.18	88.478	2.04	918.12	881.47	1.441	0.088
8	997.2	87.28	4.78	1012.63	950.91	2.323	0.355
9	1022.27	89.449	2.073	1029.99	1014.56	0.673	0.078
10	1041.56	87.116	5.315	1053.13	1031.92	0.952	0.227
11	1062.78	92.362	0.921	1068.56	1055.06	0.433	0.028
12	1122.57	80.46	11.866	1130.29	1070.49	2.671	0.703
13	1172.72	76.909	10.392	1184.29	1141.86	2.969	0.775
14	1209.37	83.756	1.683	1213.23	1199.72	0.913	0.059
15	1244.09	89.878	2.814	1257.59	1236.37	0.791	0.103
16	1267.23	92.833	0.404	1273.02	1259.52	0.42	0.012
17	1290.38	86.719	6.628	1309.67	1274.95	1.477	0.441
18	1321.24	92.501	1.015	1330.88	1311.59	0.602	0.041
19	1359.82	81.32	11.176	1369.46	1332.81	1.807	0.62
20	1384.89	92.066	1.01	1398.39	1379.1	0.624	0.032
21	1450.47	78.923	11.952	1477.47	1400.32	4.648	1.688
22	1489.05	80.692	10.165	1525.69	1479.4	2.245	0.714
23	1625.99	92.646	2.037	1645.28	1598.99	1.26	0.16
24	1741.72	94.284	0.94	1751.36	1734.01	0.402	0.034
25	2196.92	84.362	10.446	2216.21	2158.35	2.711	1.265
26	2918.3	92.729	0.63	2929.87	2889.37	1.241	0.059
27	3091.89	91.864	2.525	3115.04	3057.17	1.773	0.319

Comment;
RPB3-170_diyne

Date/Time; 30/01/2023 14:31:28
No. of Scans; 16
Resolution; 4 [1/cm]
Apodization; Happ-Genzel

Figure S200. FT-IR spectrum of 1,4-bis(5,6-dimethyl-1*H*-benzo[*d*]imidazol-1-yl)buta-1,3-diyne **15**.

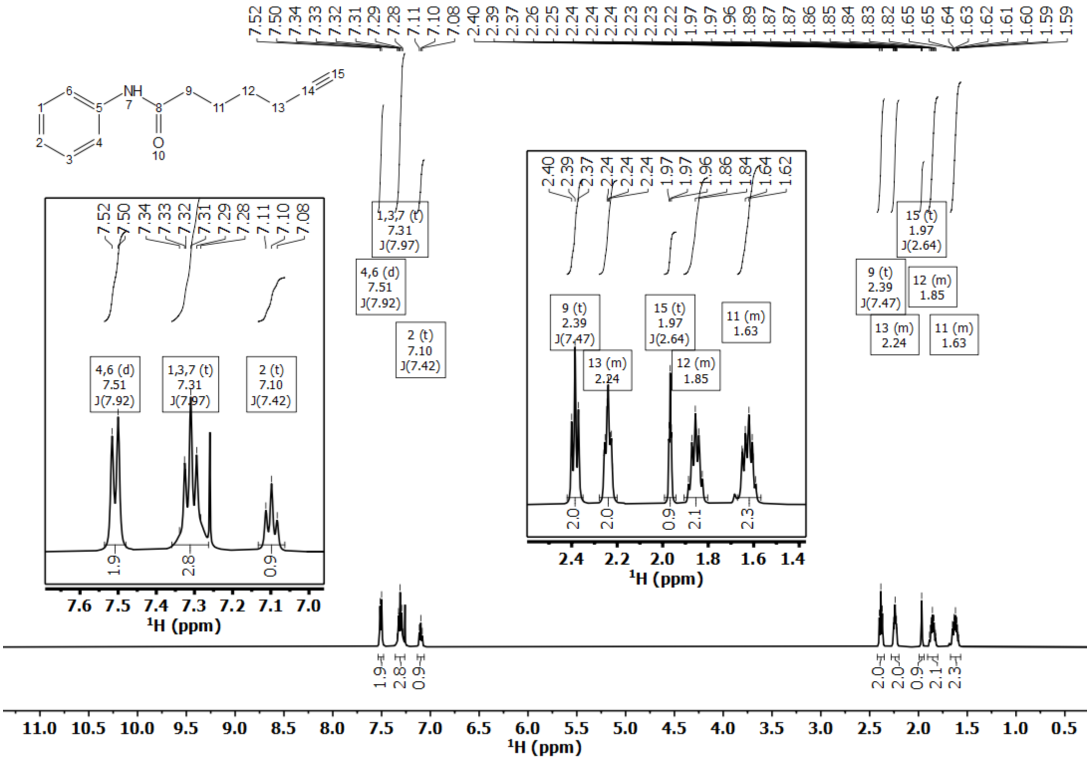

Figure S201. ^{1}H NMR spectrum of *N*-phenylhept-6-ynamide **S1**.

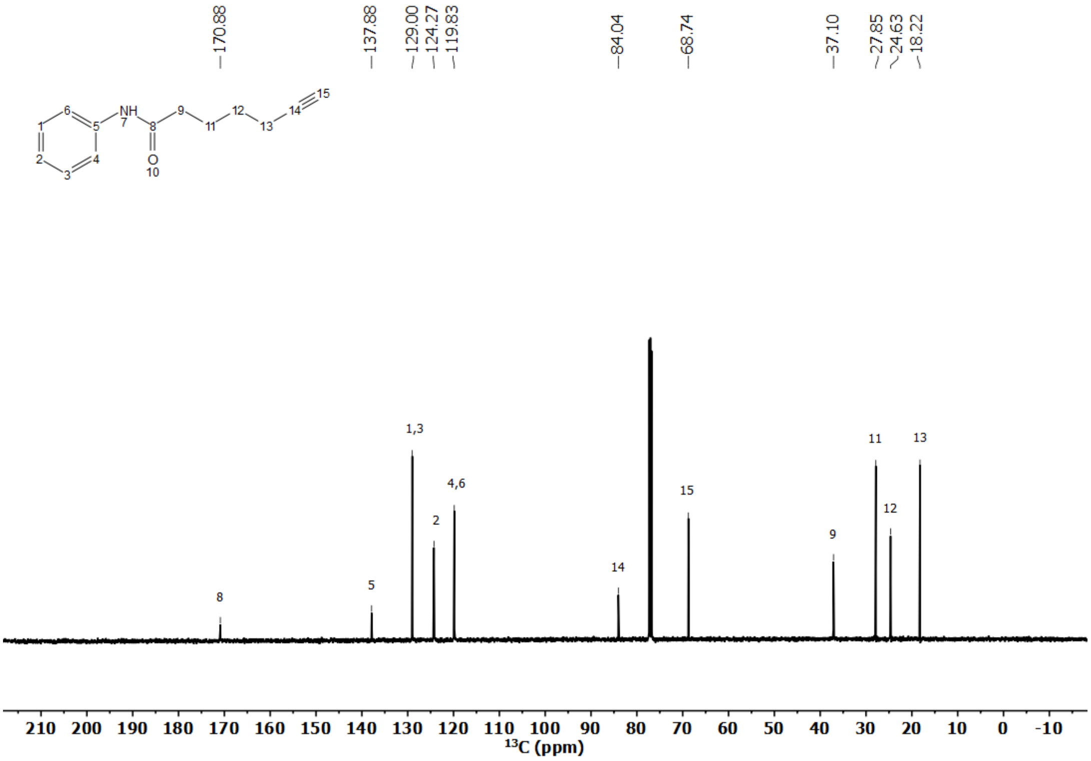

Figure S202. $^{13}C\{^{1}H\}$ NMR spectrum of *N*-phenylhept-6-ynamide **S1**.

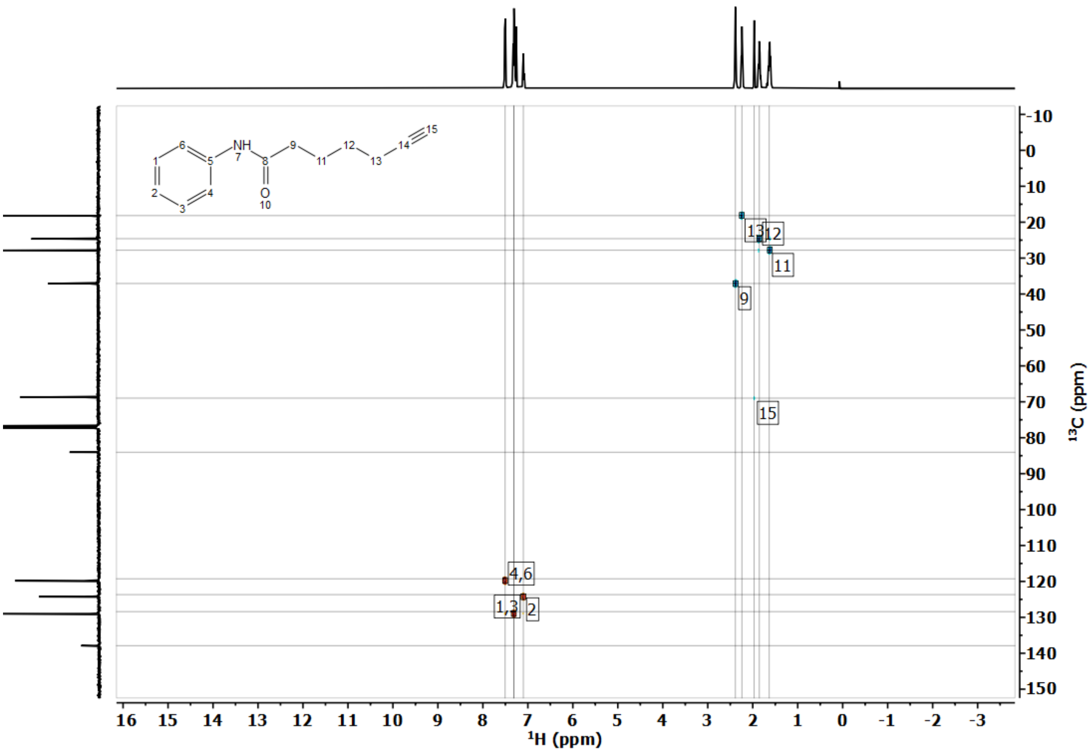

Figure S203. ^{1}H-^{13}C HSQC NMR spectrum of *N*-phenylhept-6-ynamide **S1**.

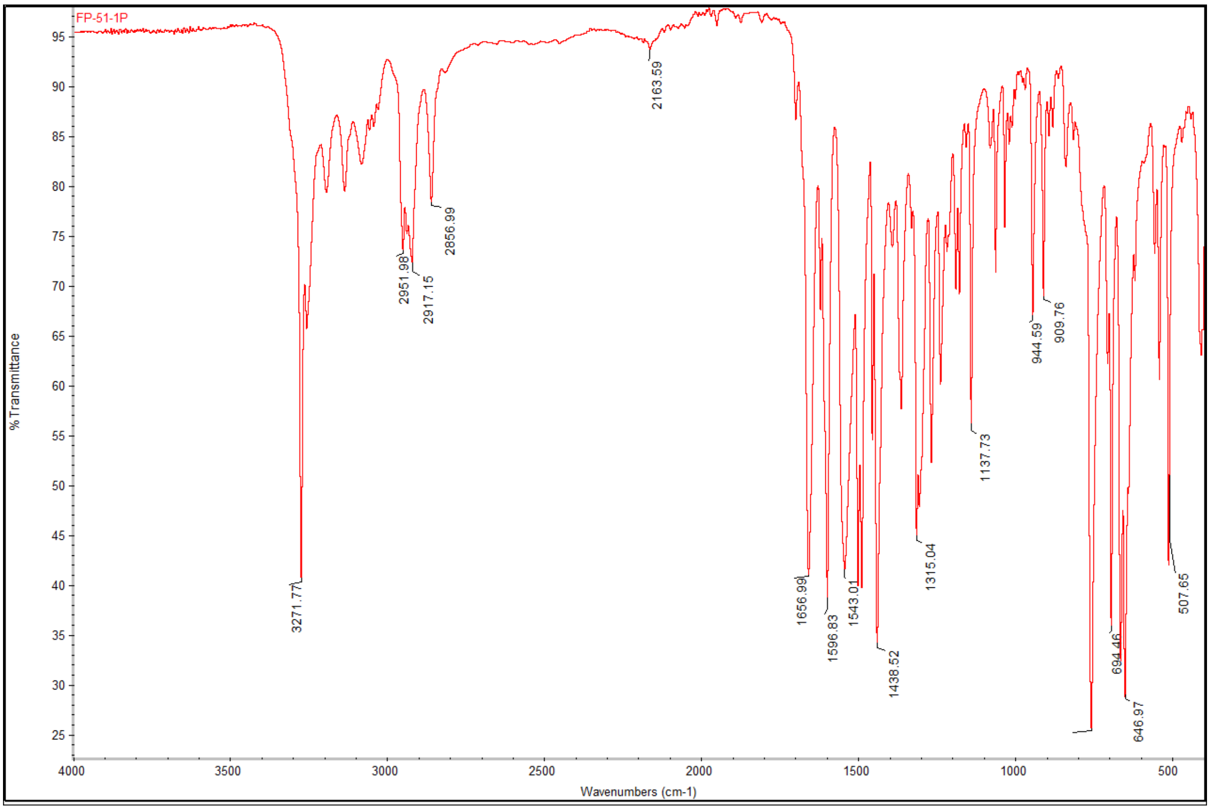

Figure S204. FT-IR spectrum of *N*-phenylhept-6-ynamide **S1**.

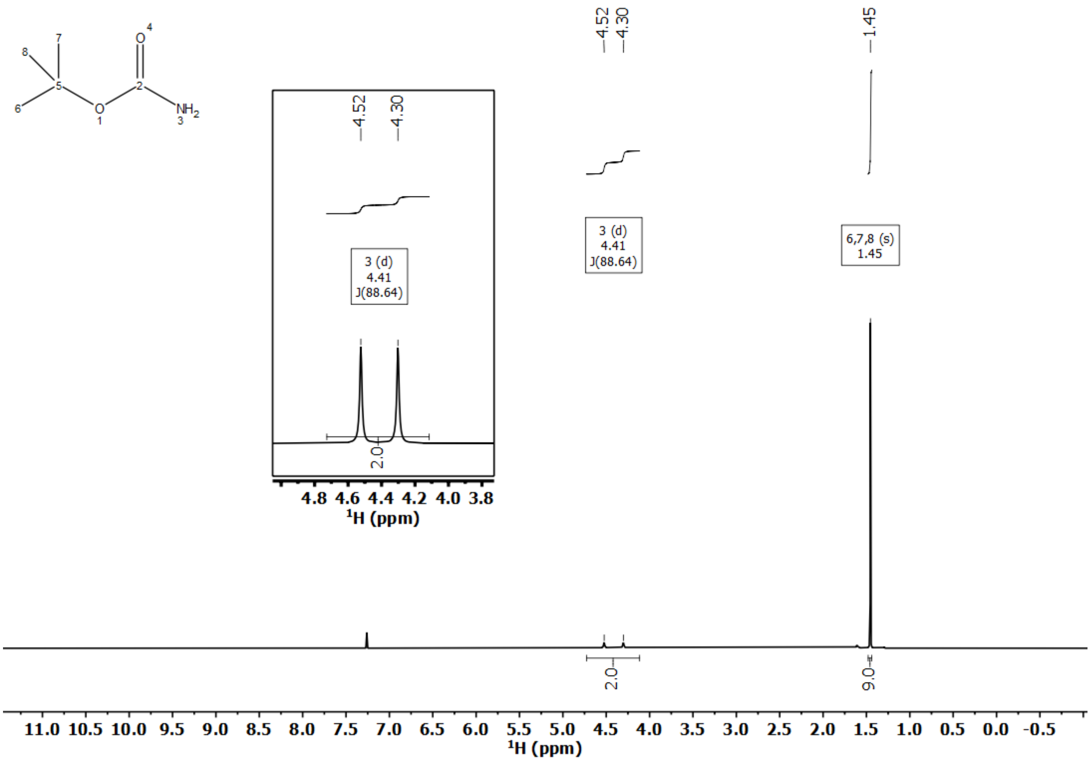

Figure S205. ^{1}H NMR spectrum of *tert*-Butyl [^{15}*N*] carbamate **S3**.

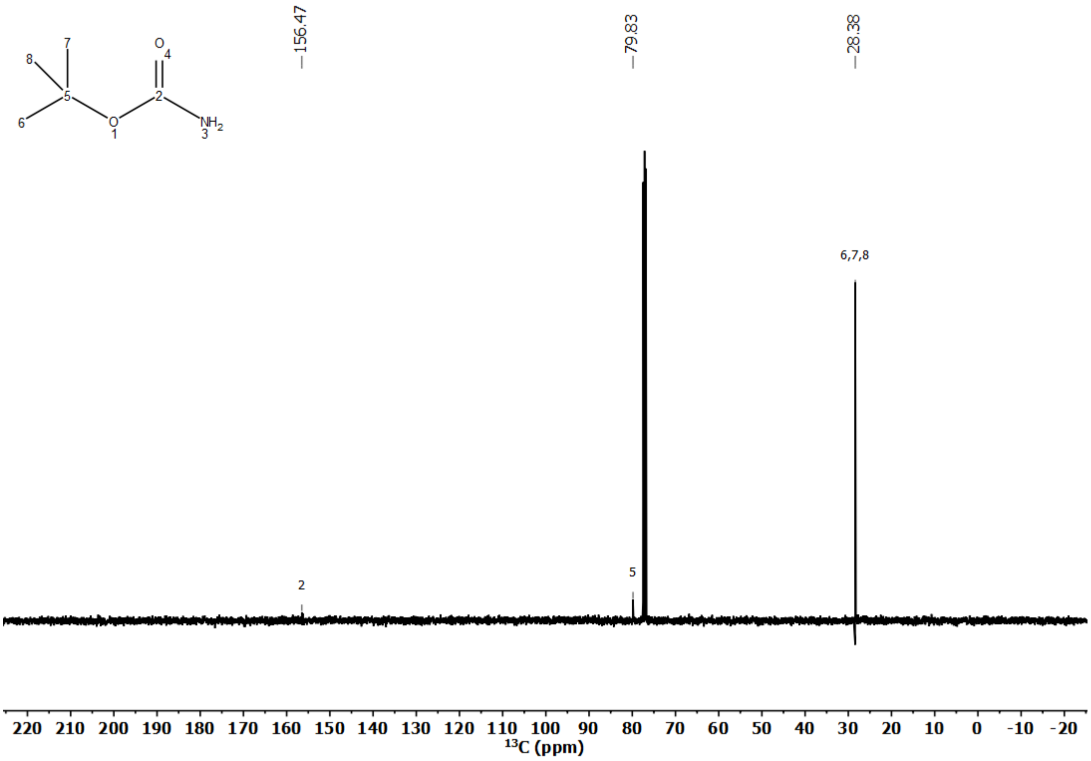

Figure S206. $^{13}C\{^{1}H\}$ NMR spectrum of *tert*-Butyl [^{15}*N*] carbamate **S3**.

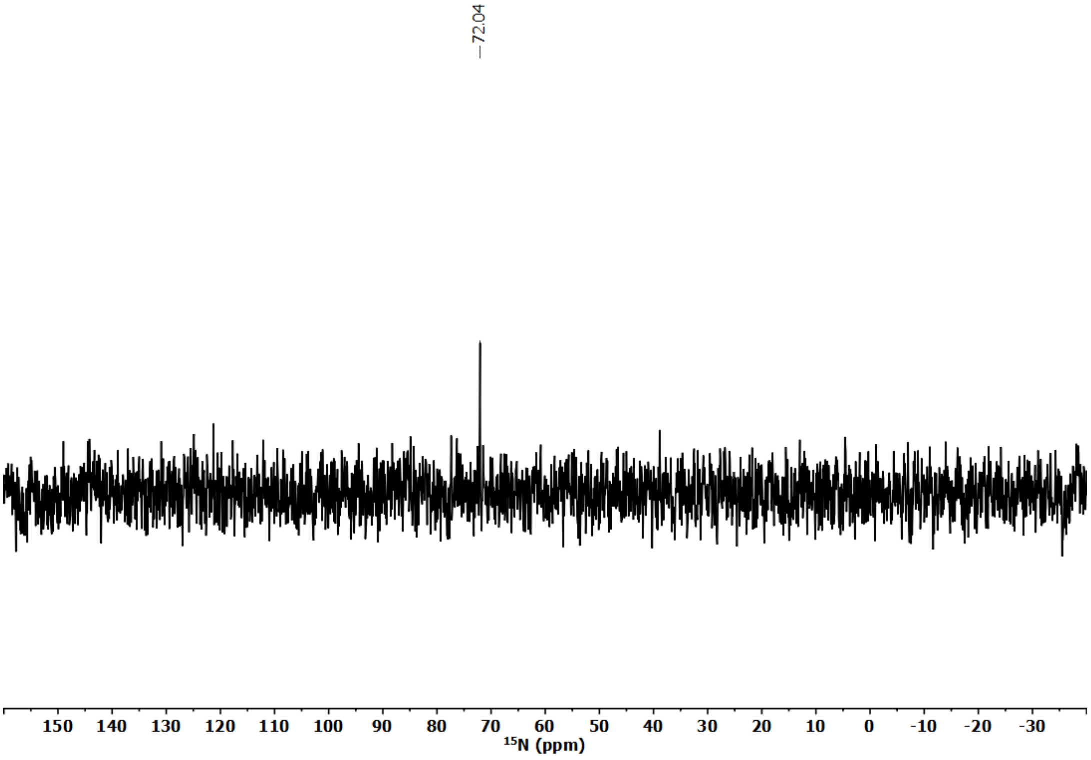

Figure S207. ^{15}N NMR spectrum of *tert*-Butyl [^{15}*N*] carbamate **S3**.

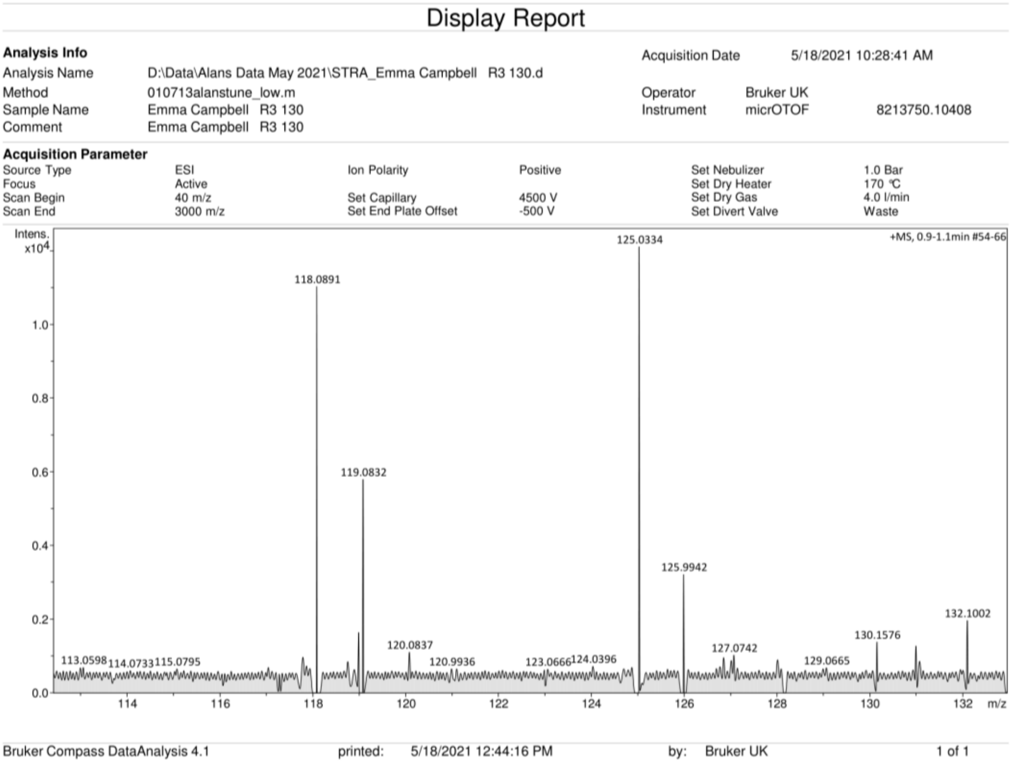

Figure S208. HRMS analysis of *tert*-Butyl [^{15}N] carbamate **S3**.

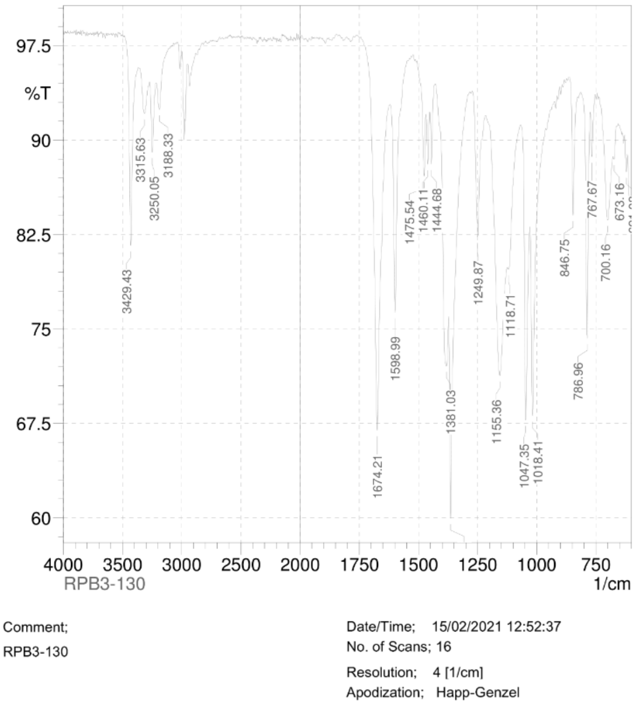

SHIMADZU

No.	Peak	Intensity	Corr. Inte	Base (H)	Base (L)	Area	Corr. Are
1	601.79	83.928	0.695	615.29	599.86	1.085	0.075
2	621.08	87.462	1.932	628.79	617.22	0.614	0.058
3	673.16	88.488	0.691	677.01	655.8	0.949	0.02
4	700.16	83.655	6.694	734.88	678.94	3.148	0.805
5	767.67	88.678	3.49	771.53	754.17	0.688	0.123
6	786.96	74.257	18.513	812.03	773.46	2.259	1.035
7	846.75	84.033	10.274	862.18	827.46	1.439	0.534
8	1018.41	68.097	16.081	1028.06	962.48	5.313	1.354
9	1047.35	67.782	19.165	1066.64	1029.99	3.591	1.369
10	1118.71	79.726	1.345	1124.5	1068.56	3.557	0.087
11	1155.36	71.29	12.503	1215.15	1126.43	8.19	2.281
12	1249.87	82.317	9.291	1271.09	1234.44	2.064	0.702
13	1363.67	59.96	16.892	1371.39	1282.66	7.098	1.173
14	1381.03	72.056	6.57	1427.32	1373.32	4.429	0.749
15	1444.68	88.084	4.736	1450.47	1429.25	0.762	0.143
16	1460.11	88.076	4.07	1467.83	1452.4	0.689	0.142
17	1475.54	87.138	5.333	1506.41	1469.76	1.229	0.227
18	1598.99	76.305	17.457	1618.28	1546.91	3.288	1.567
19	1674.21	66.945	28.25	1741.72	1620.21	7.194	4.717
20	3188.33	92.437	2.828	3213.41	3153.61	1.592	0.369
21	3250.05	89.032	5.55	3271.27	3219.19	1.869	0.608
22	3315.63	92.098	3.087	3354.21	3273.2	2.314	0.567
23	3429.43	81.636	15.4	3473.8	3369.64	3.717	2.265

Figure S209. FT-IR spectrum of *tert*-Butyl [^{15}N] carbamate **S3**.

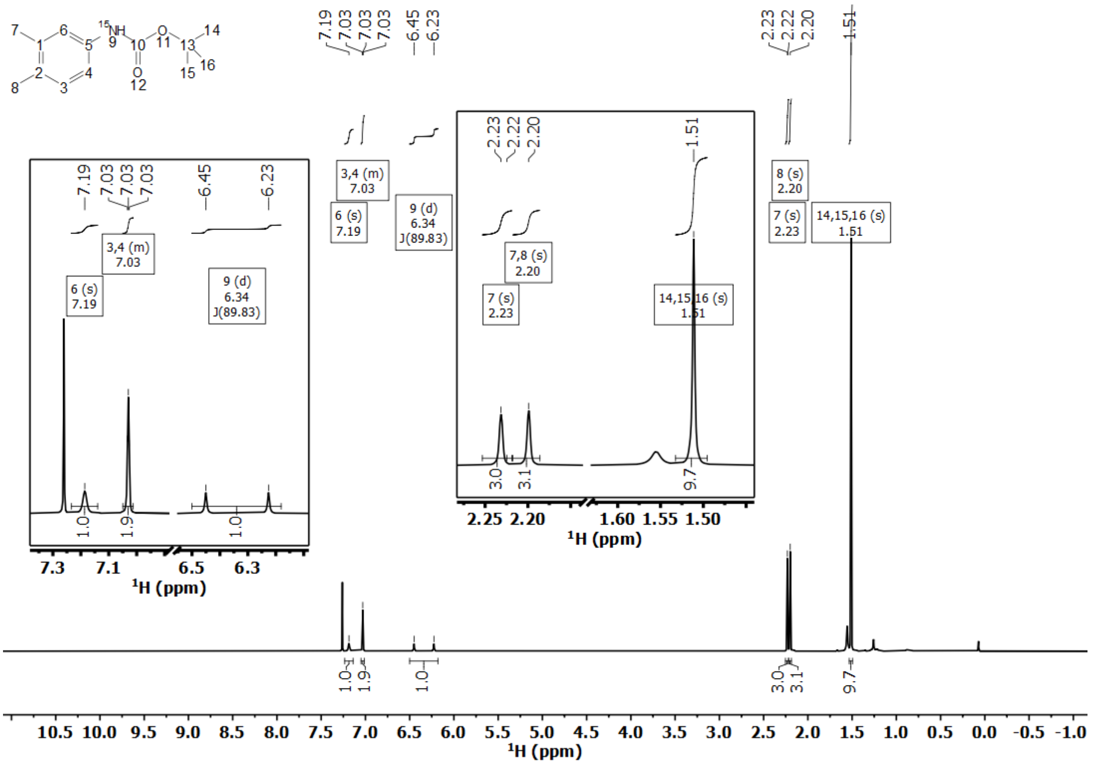

Figure S210. ^{1}H NMR spectrum of *tert*-Butyl (3,4-dimethylphenyl) [^{15}*N*] carbamate **S4**.

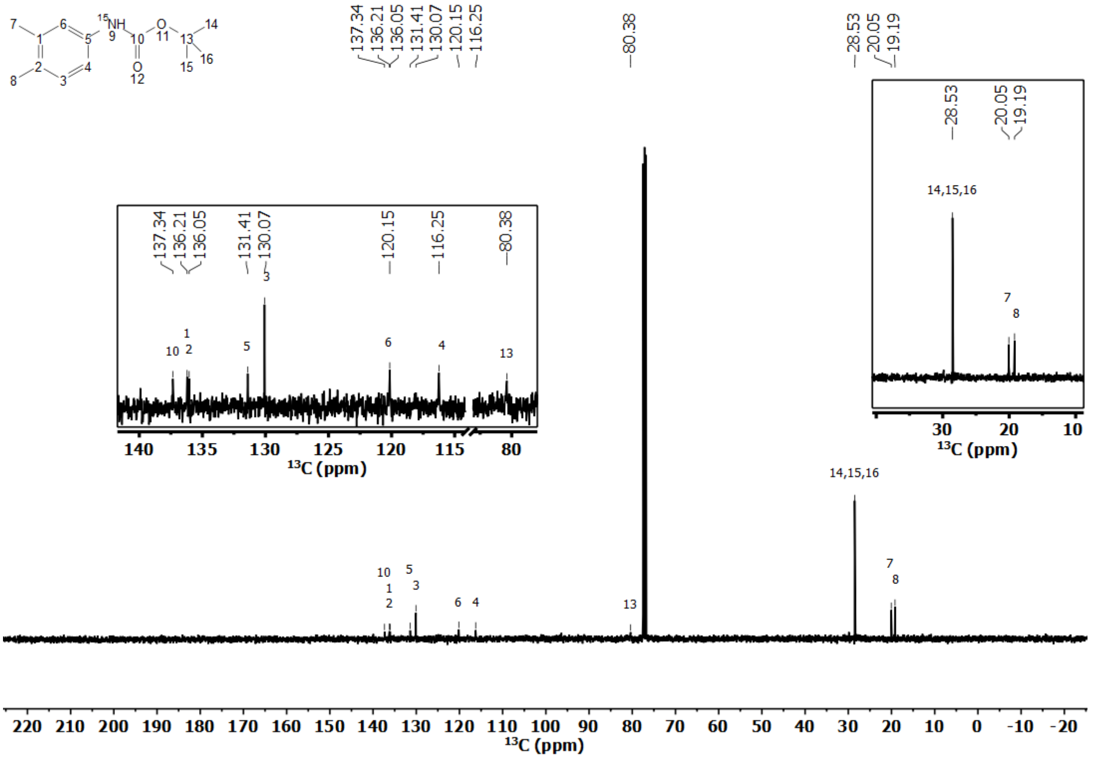

Figure S211. ^{13}C{^{1}H} NMR spectrum of *tert*-Butyl (3,4-dimethylphenyl) [^{15}*N*] carbamate **S4**.

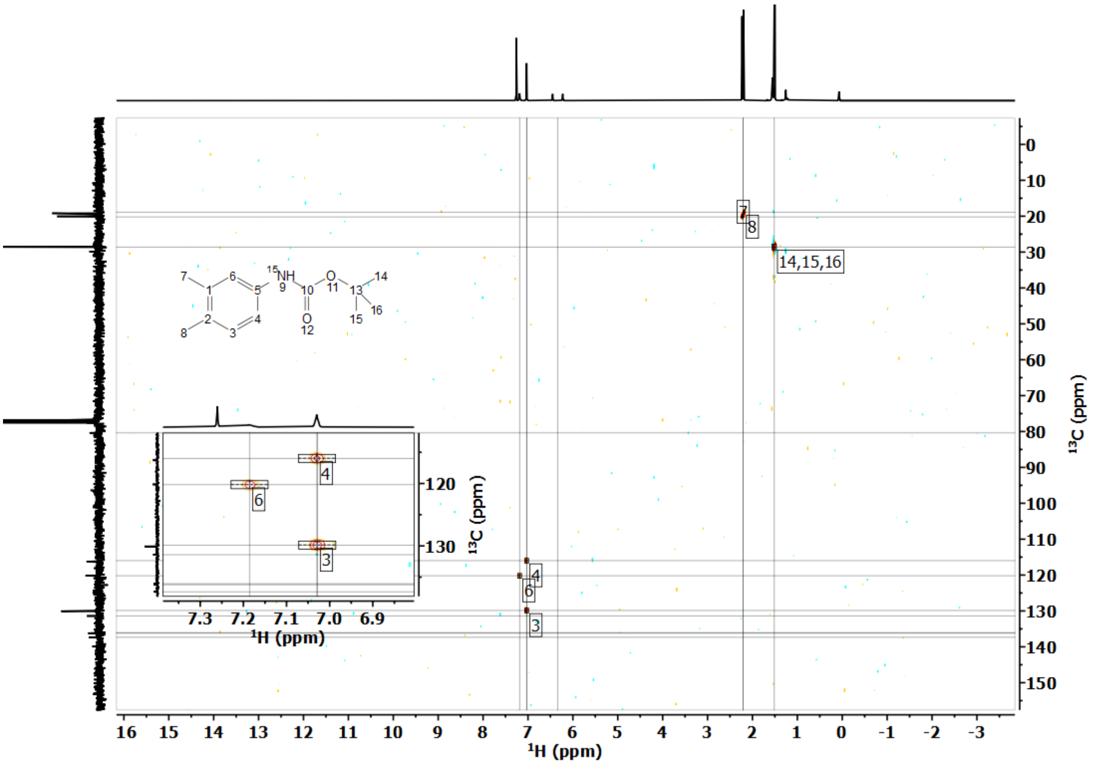

Figure S212. ^1H-^{13}C HSQC NMR spectrum of *tert*-Butyl (3,4-dimethylphenyl) [^{15}N] carbamate **S4**.

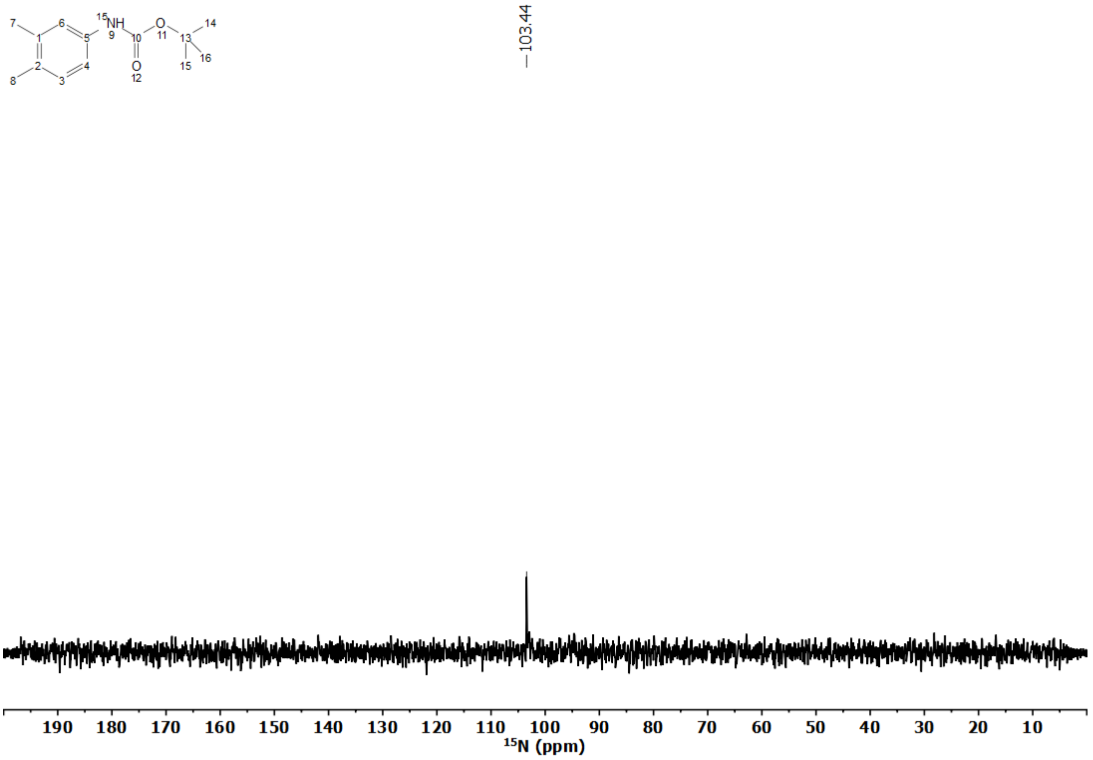

Figure S213. ^{15}N NMR spectrum *tert*-Butyl (3,4-dimethylphenyl) [^{15}N] carbamate **S4**.

Display Report

Analysis Info

Analysis Name D:\Data\Alans Data May 2021\STRA_Emma Campbell R252.d
Method 010713alanstune_low.m
Sample Name Emma Campbell R252
Comment Emma Campbell R252

Acquisition Date 5/18/2021 4:10:53 PM
Operator Bruker UK
Instrument micrOTOF 8213750.10408

Acquisition Parameter

Source Type	ESI	Ion Polarity	Positive	Set Nebulizer	1.0 Bar
Focus	Active			Set Dry Heater	230 °C
Scan Begin	40 m/z	Set Capillary	4000 V	Set Dry Gas	6.0 l/min
Scan End	3000 m/z	Set End Plate Offset	-500 V	Set Divert Valve	Waste

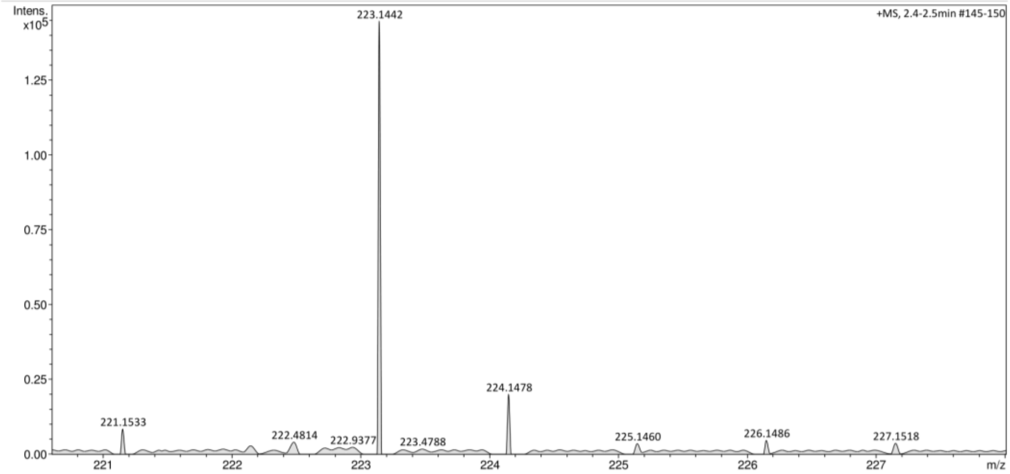

Bruker Compass DataAnalysis 4.1 printed: 5/18/2021 4:17:58 PM by: Bruker UK 1 of 1

Figure S214. HRMS analysis of *tert*-Butyl (3,4-dimethylphenyl) [^{15}N] carbamate **S4**.

SHIMADZU

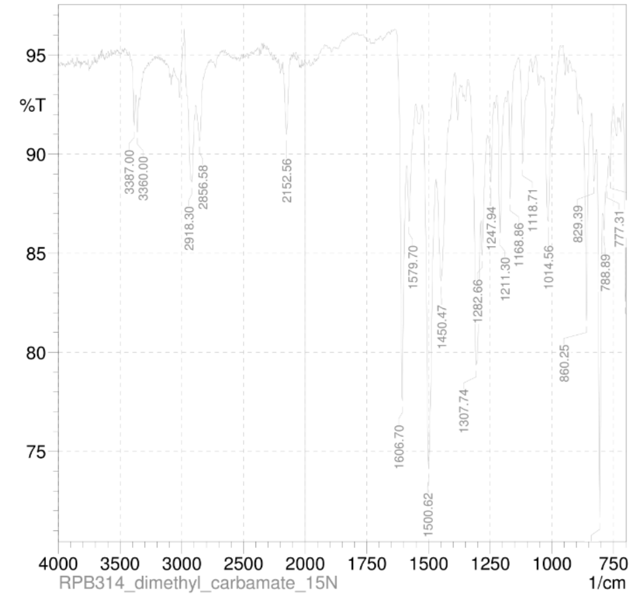

No.	Peak	Intensity	Corr. Inte	Base (H)	Base (L)	Area	Corr. Are
1	702.09	82.559	6.354	711.73	698.23	0.785	0.146
2	763.81	88.882	1.5	767.67	752.24	0.642	0.032
3	777.31	88.375	0.307	779.24	769.6	0.482	0.008
4	788.89	86.163	1.252	792.74	781.17	0.689	0.033
5	806.25	71.67	16.852	817.82	794.67	2.051	0.809
6	829.39	88.623	2.536	839.03	819.75	0.898	0.114
7	860.25	81.607	10.766	881.47	840.96	2.039	0.643
8	1014.56	86.602	5.693	1031.92	1002.98	1.39	0.404
9	1118.71	89.507	5.337	1134.14	1085.92	1.552	0.441
10	1168.86	87.742	5.673	1176.58	1136.07	1.295	0.217
11	1211.3	86.013	6.898	1222.87	1190.08	1.458	0.407
12	1247.94	88.582	3.1	1259.52	1236.37	1	0.134
13	1282.66	84.891	2.685	1288.45	1261.45	1.476	0.11
14	1307.74	79.357	9.46	1340.53	1290.38	3.131	0.811
15	1450.47	83.606	6.245	1469.76	1417.68	3.08	0.767
16	1500.62	74.124	16.585	1525.69	1471.69	4.282	1.936
17	1579.7	86.611	3.499	1587.42	1552.7	1.641	0.226
18	1606.7	77.544	14.67	1631.78	1589.34	2.428	1.022
19	2152.56	90.994	3.396	2173.78	2121.7	1.698	0.408
20	2856.58	90.663	2.498	2887.44	2823.79	2.291	0.338
21	2918.3	88.587	0.881	2926.01	2889.37	1.622	0.061
22	3360	91.107	1.815	3373.5	3344.57	1.025	0.102
23	3387	91.424	2.226	3410.15	3373.5	1.13	0.125

Comment;
RPB314_dimethyl_carbamate_15N

Date/Time; 30/01/2023 13:19:24
No. of Scans; 16
Resolution; 4 [1/cm]
Apodization; Happ-Genzel

Figure S215. FT-IR spectrum of *tert*-Butyl (3,4-dimethylphenyl) [^{15}N] carbamate **S4**.

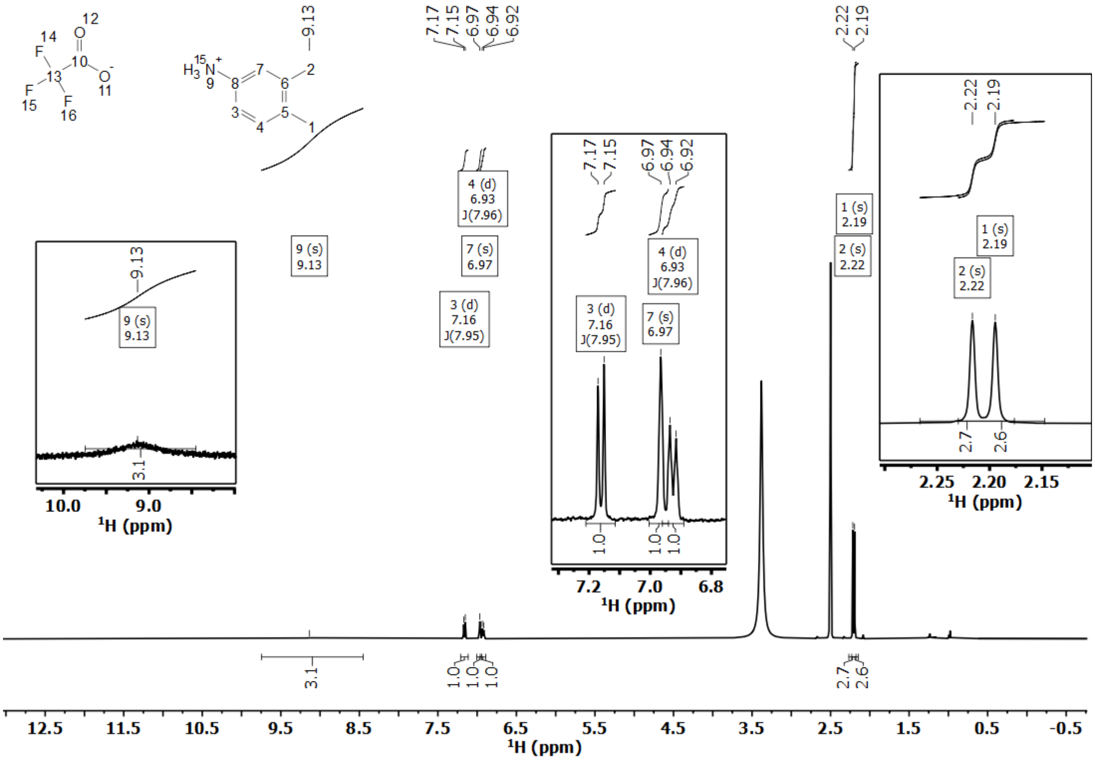

Figure S216. ^{1}H NMR spectrum of 3,4-dimethylbenzenaminium-^{15}*N* 2,2,2-trifluoroacetate **S5**.

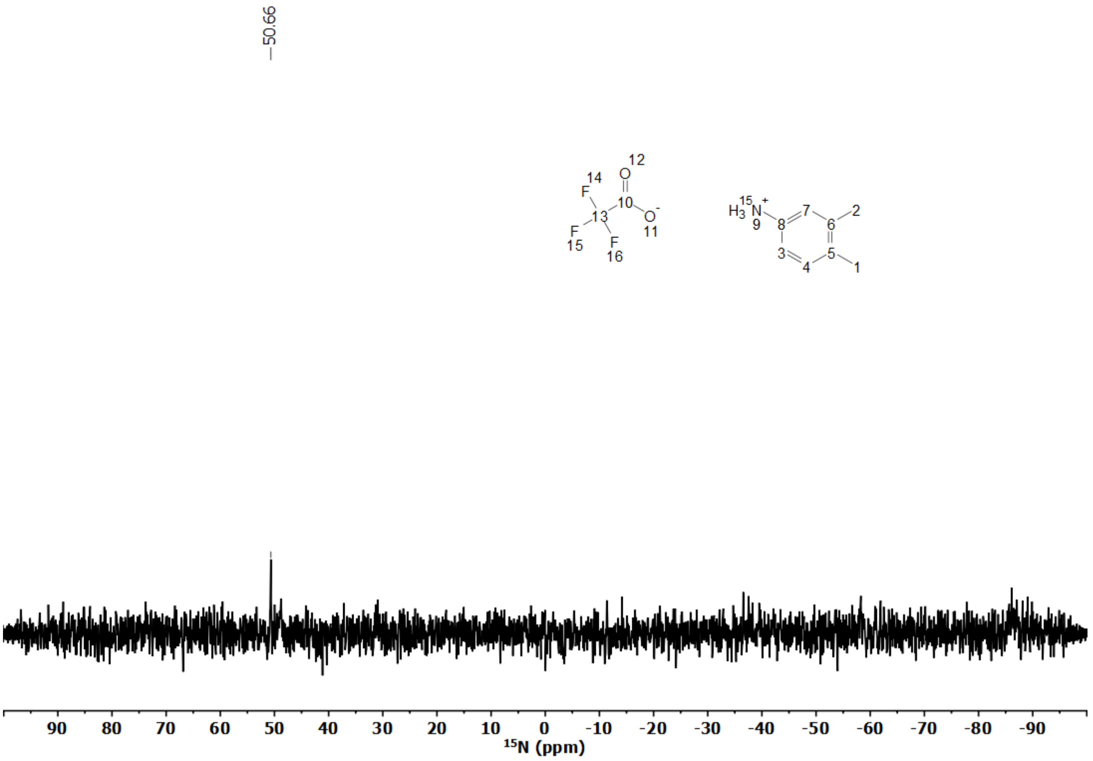

Figure S217. ^{15}N NMR spectrum of 3,4-dimethylbenzenaminium-^{15}*N* 2,2,2-trifluoroacetate **S5**.

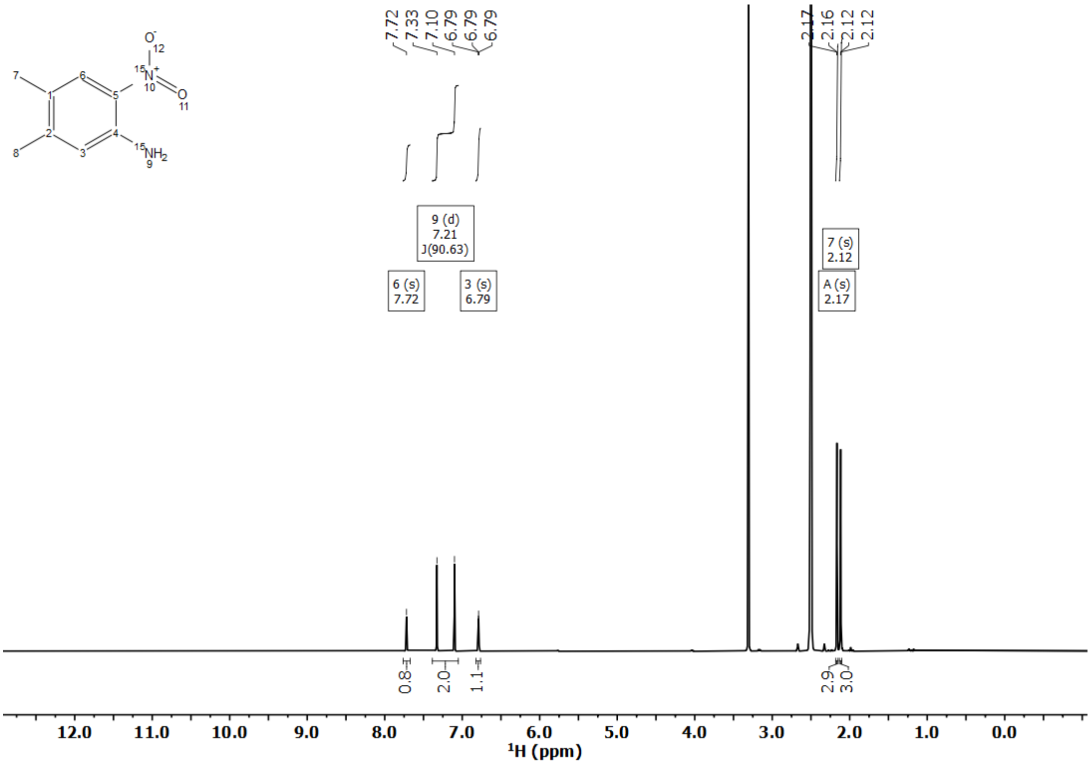

Figure S218. ^{1}H NMR spectrum of 4,5-dimethyl-2-(nitro)aniline-$^{15}N_2$ **S6**.

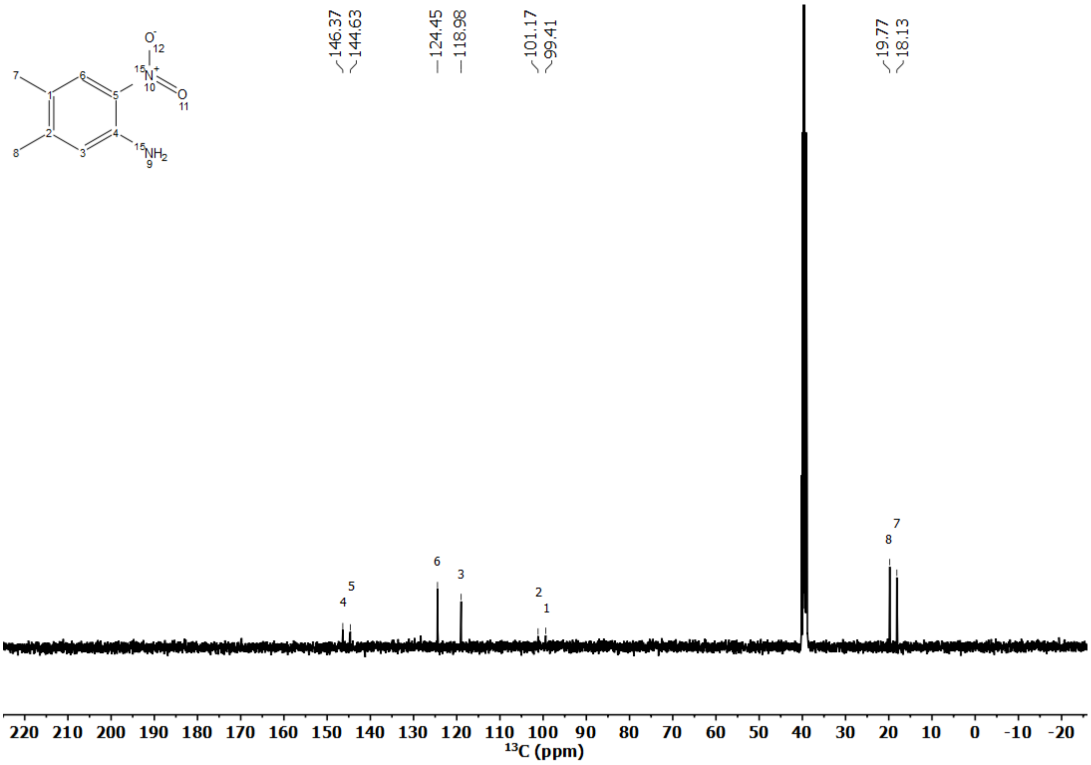

Figure S219. ^{13}C{^{1}H} NMR spectrum of 4,5-dimethyl-2-(nitro)aniline-$^{15}N_2$ **S6**.

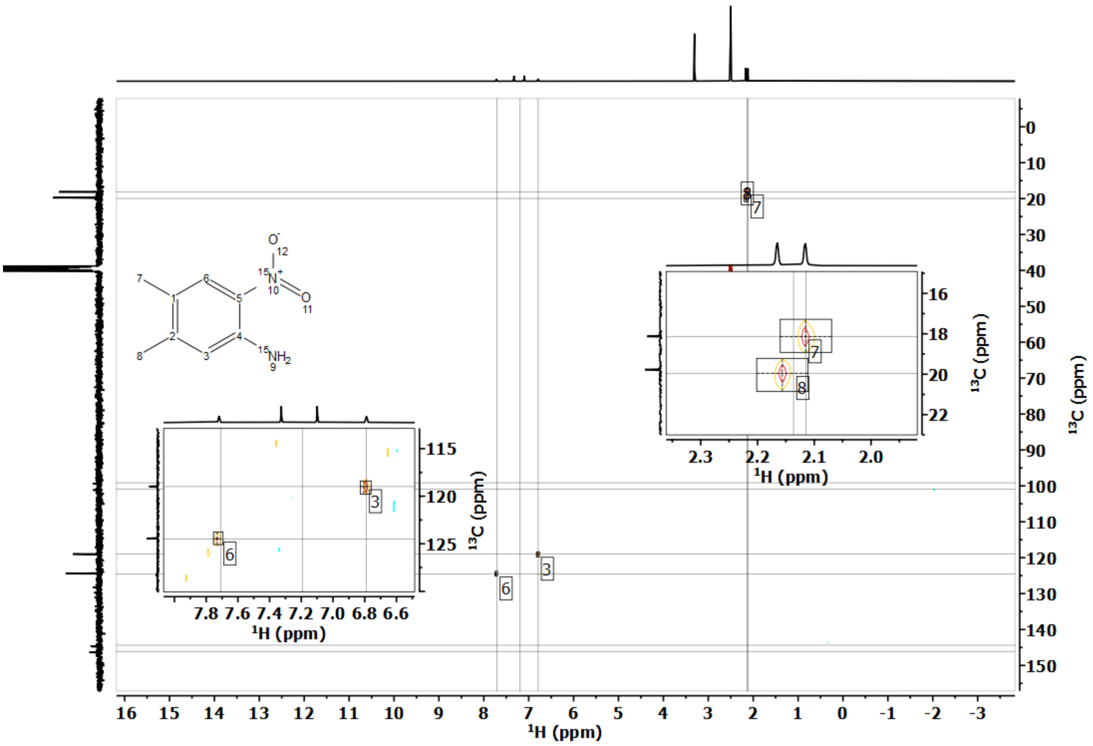

Figure S220. ^{1}H-^{13}C HSQC NMR spectrum of 4,5-dimethyl-2-(nitro)aniline-$^{15}N_2$ **S6**.

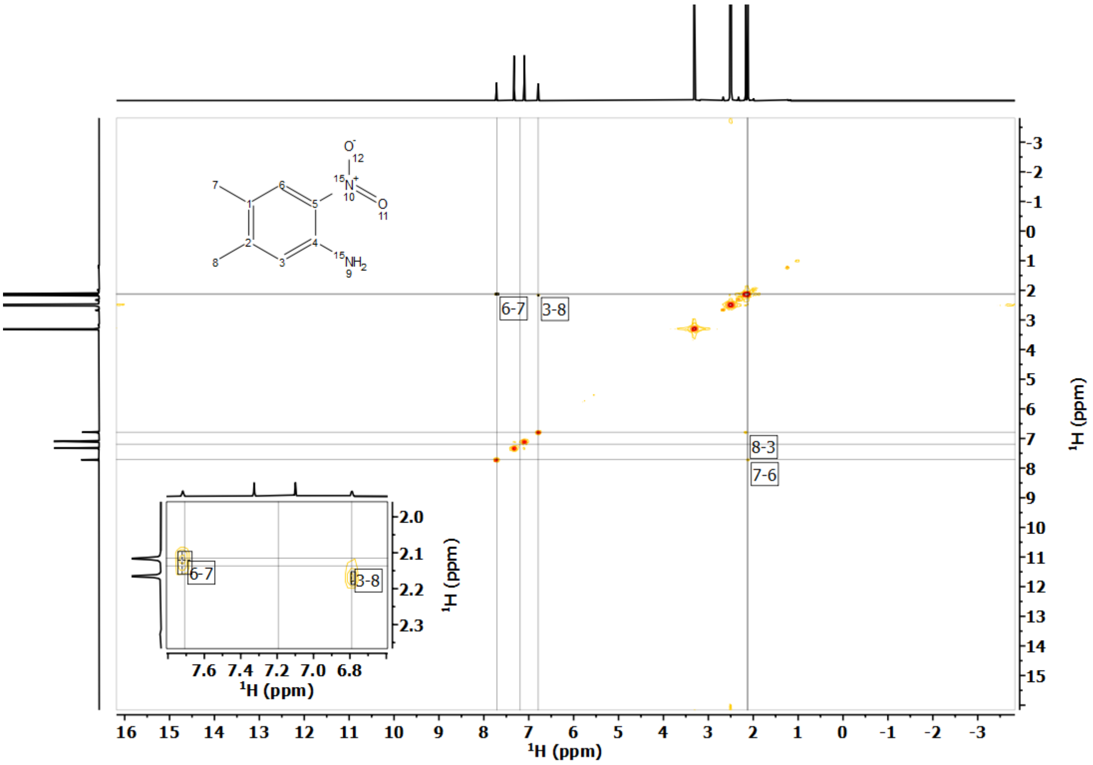

Figure S221. ^{1}H-^{1}H COSY NMR spectrum of 4,5-dimethyl-2-(nitro)aniline-$^{15}N_2$ **S6**.

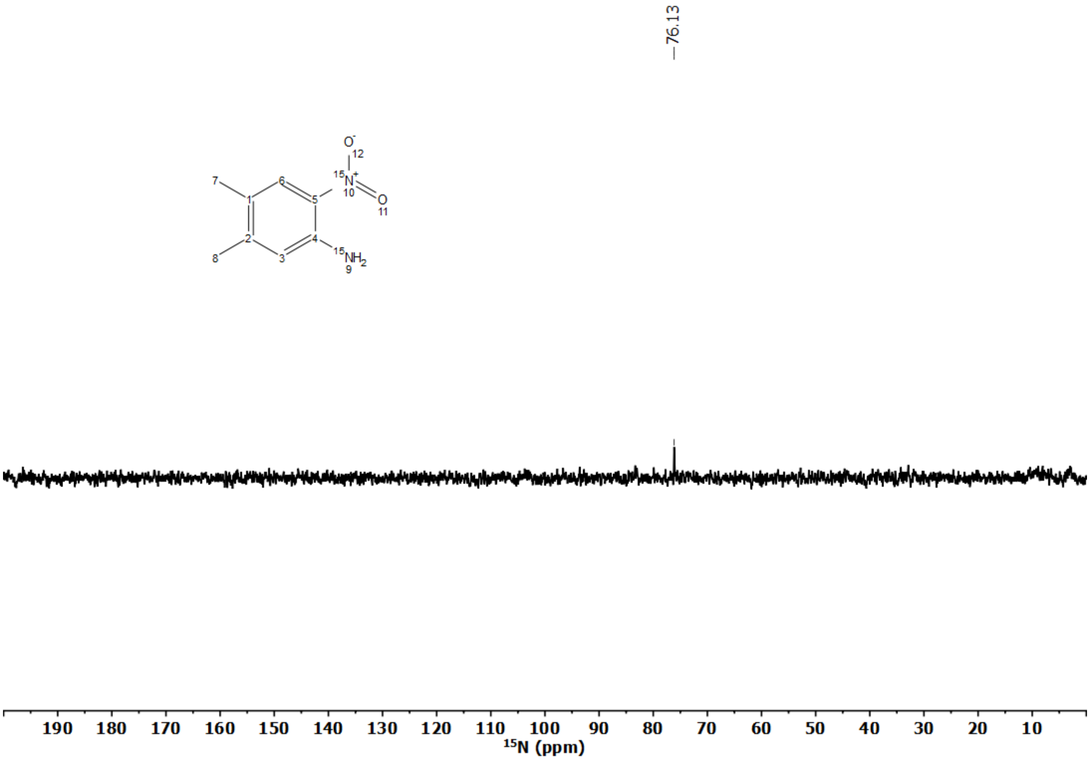

Figure S222. ^{15}N NMR spectrum of 4,5-dimethyl-2-(nitro)aniline-$^{15}N_2$ **S6**.

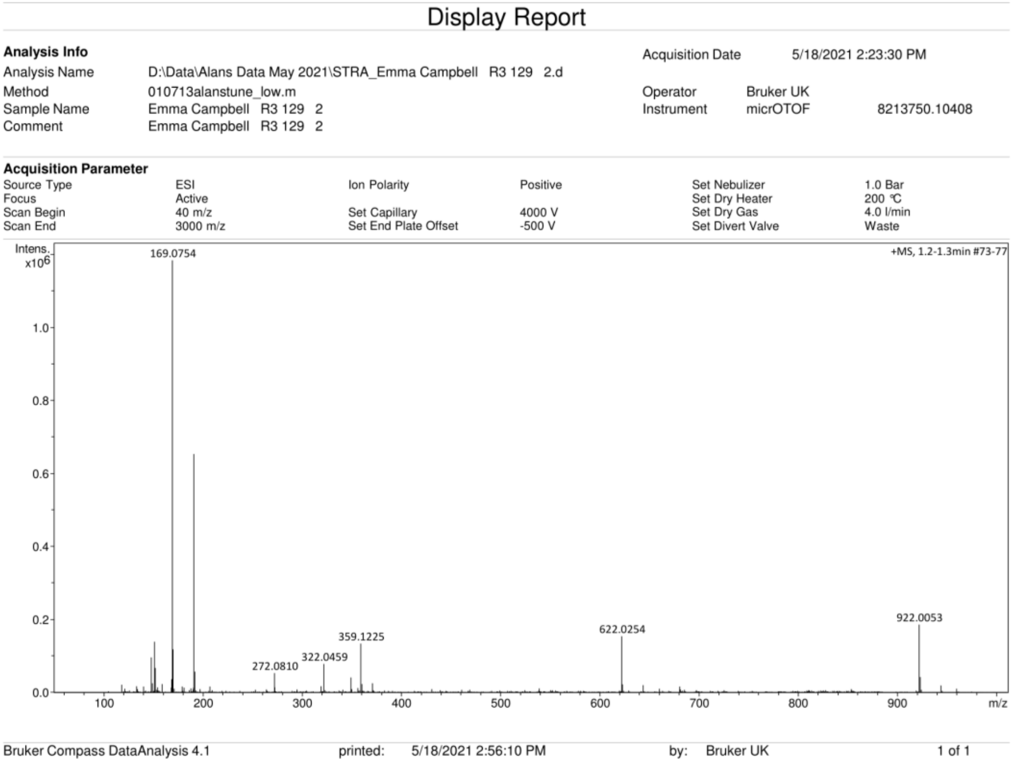

Figure S223. HRMS analysis of 4,5-dimethyl-2-(nitro)aniline-$^{15}N_2$ **S6**.

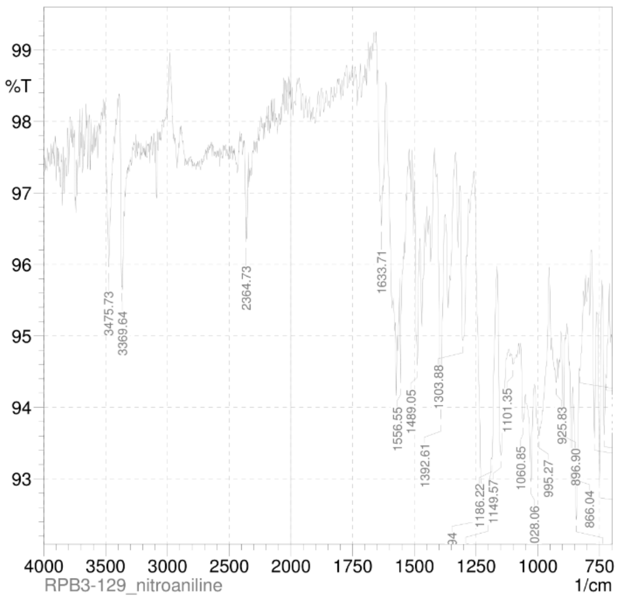

SHIMADZU

No.	Peak	Intensity	Corr. Inte	Base (H)	Base (L)	Area	Corr. Are
1	723.31	94.4101	0.2418	725.23	711.73	0.3051	0.0162
2	731.02	93.6176	1.3709	738.74	725.23	0.3432	0.0416
3	750.31	92.9023	2.5484	756.1	740.67	0.391	0.0859
4	771.53	93.5677	2.081	783.1	763.81	0.4538	0.0851
5	829.39	94.5105	0.1721	831.32	815.89	0.3459	0.0131
6	844.82	92.4272	1.9991	858.32	833.25	0.7444	0.1198
7	866.04	93.1728	1.1748	869.9	860.25	0.2767	0.0306
8	896.9	93.7661	1.2006	904.61	891.11	0.3411	0.0383
9	925.83	94.347	0.3888	943.19	921.97	0.5152	0.0242
10	995.27	93.6027	0.3547	999.13	954.76	1.1277	0.1205
11	1028.06	92.9608	1.2995	1051.2	1014.56	1.0206	0.0777
12	1060.85	93.793	0.6844	1068.56	1053.13	0.4116	0.0276
13	1101.35	94.604	0.1315	1105.21	1087.85	0.4138	0.0059
14	1149.57	93.3268	2.2614	1165	1118.71	1.1904	0.2368
15	1186.22	93.2696	0.2657	1188.15	1166.93	0.5068	0.0101
16	1201.65	92.4362	0.6313	1213.23	1190.08	0.7593	0.0389
17	1220.94	92.5878	0.8725	1259.52	1215.15	1.1286	0.14
18	1303.88	94.9315	1.6987	1315.45	1290.38	0.4982	0.116
19	1392.61	93.8356	3.1357	1408.04	1377.17	0.6162	0.2013
20	1489.05	94.5879	2.1177	1498.69	1479.4	0.3899	0.1058
21	1556.55	94.3418	1.5667	1560.41	1541.12	0.3755	0.041
22	1633.71	96.5394	0.4814	1637.56	1627.92	0.1337	0.0077
23	2364.73	96.3142	0.5647	2380.16	2360.87	0.245	0.007
24	3369.64	95.6808	0.2462	3390.86	3367.71	0.2955	-0.007
25	3475.73	95.9596	1.1681	3489.23	3450.65	0.5868	0.1043

Comment;
RPB3-129_nitroaniline

Date/Time; 30/01/2023 13:41:30
No. of Scans; 16
Resolution; 4 [1/cm]
Apodization; Happ-Genzel

Figure S224. FT-IR spectrum of 4,5-dimethyl-2-(nitro)aniline-$^{15}N_2$ **S6**.

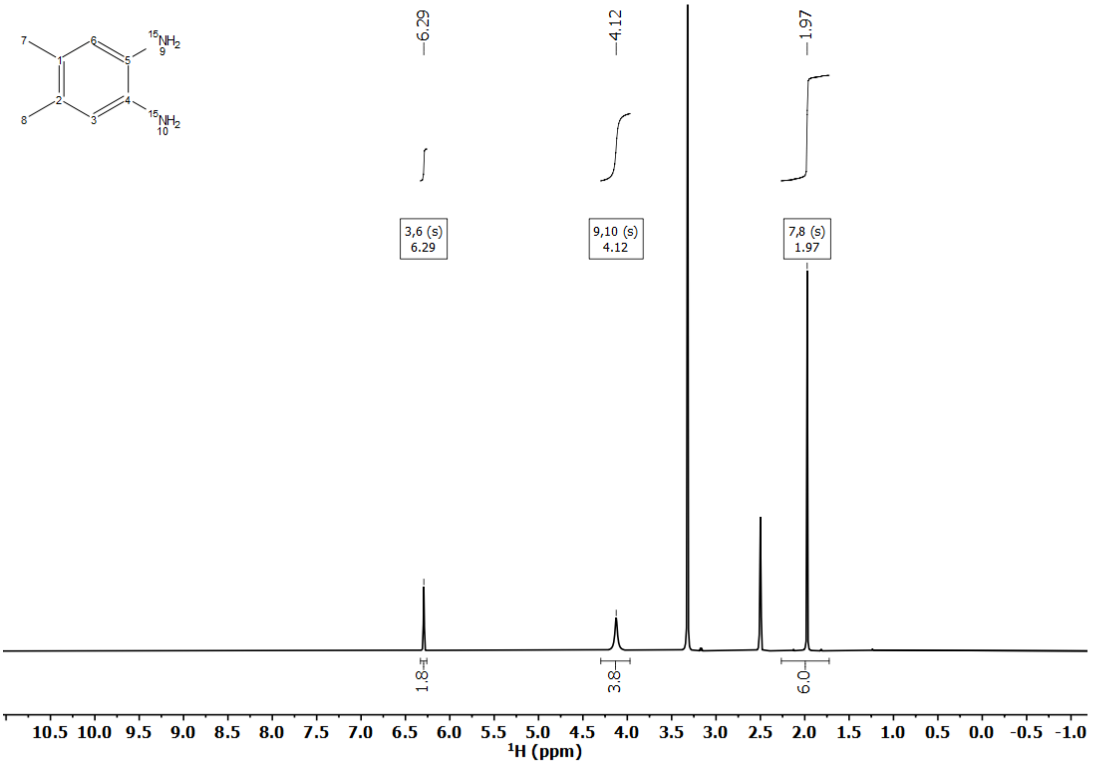

Figure S225. ^{1}H NMR spectrum of 4,5-dimethylbenzene-1,2-diamine-$^{15}N_2$ **S7**.

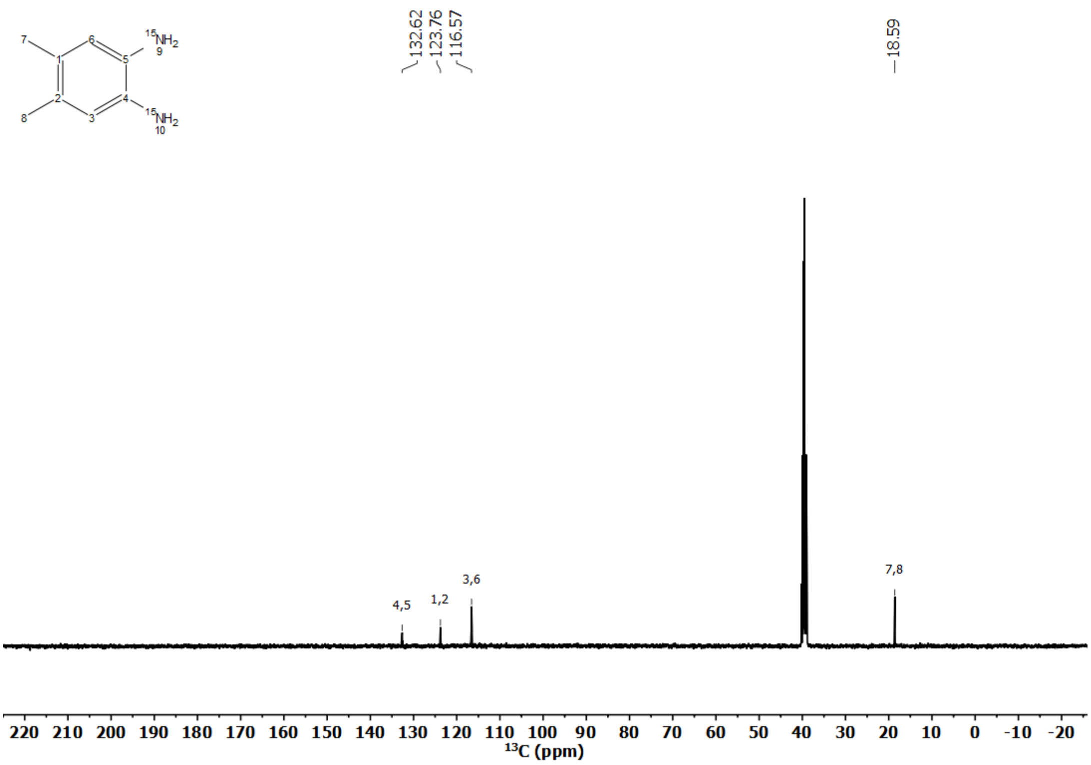

Figure S226. $^{13}C\{^{1}H\}$ NMR spectrum of 4,5-dimethylbenzene-1,2-diamine-$^{15}N_2$ **S7**.

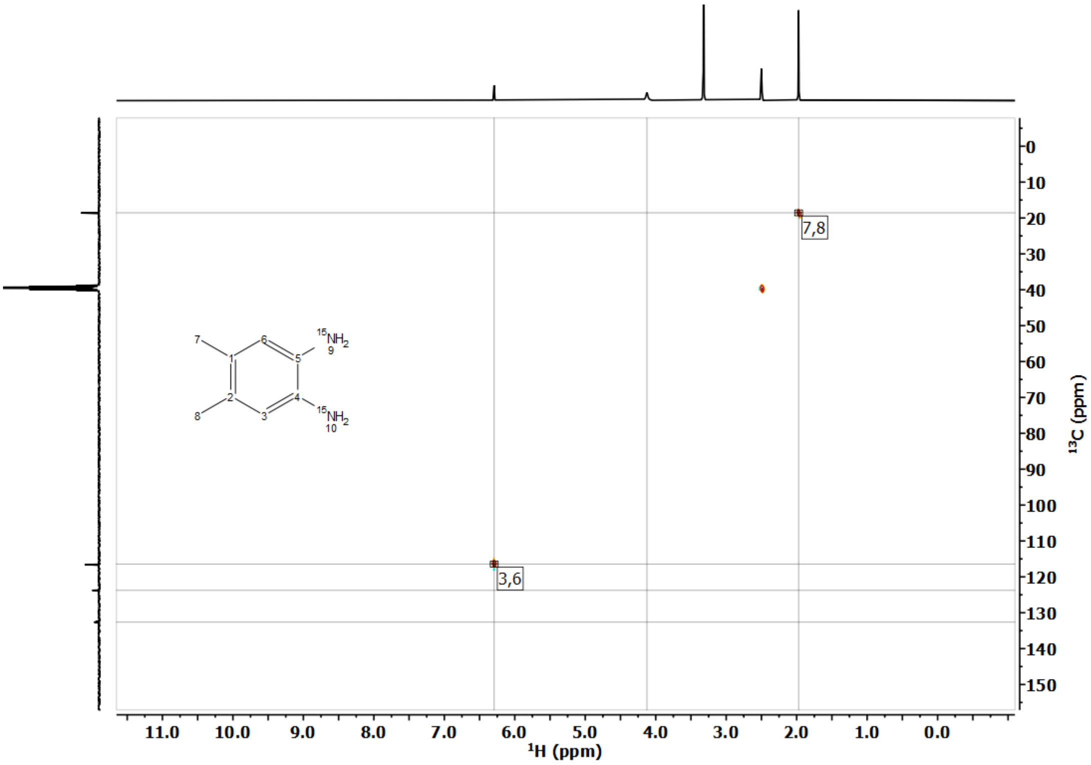

Figure S227. ^{1}H-^{13}C HSQC NMR spectrum of 4,5-dimethylbenzene-1,2-diamine-$^{15}N_2$ **S7**.

Display Report

Analysis Info

Analysis Name	D:\Data\Alans Data May 2021\STRA_Emma Campbell R3 131.d	Acquisition Date	5/18/2021 11:05:40 AM
Method	010713alanstune_low.m	Operator	Bruker UK
Sample Name	Emma Campbell R3 131	Instrument	micrOTOF 8213750.10408
Comment	Emma Campbell R3 131		

Acquisition Parameter

Source Type	ESI	Ion Polarity	Positive	Set Nebulizer	1.0 Bar
Focus	Active			Set Dry Heater	160 °C
Scan Begin	40 m/z	Set Capillary	3000 V	Set Dry Gas	4.0 l/min
Scan End	3000 m/z	Set End Plate Offset	-500 V	Set Divert Valve	Waste

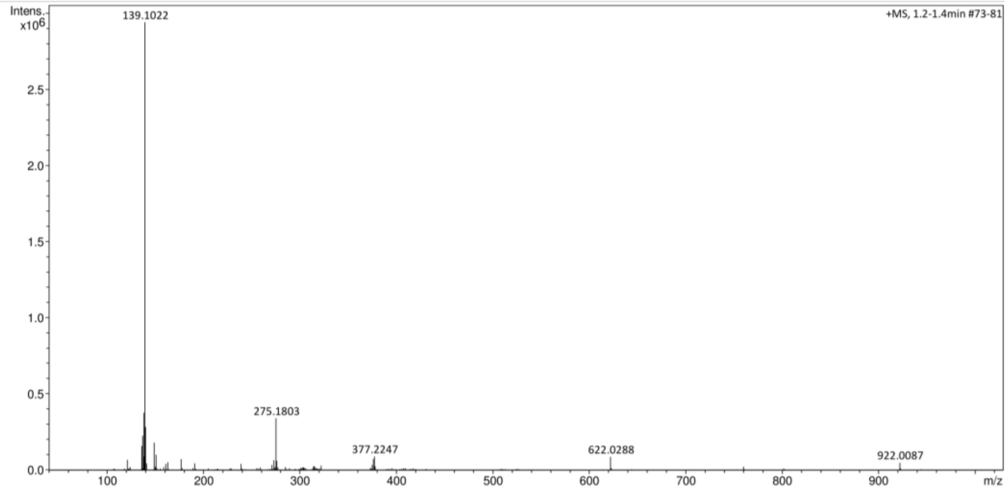

Bruker Compass DataAnalysis 4.1 printed: 5/18/2021 12:17:54 PM by: Bruker UK 1 of 1

Figure S228. HRMS analysis of 4,5-dimethylbenzene-1,2-diamine-$^{15}N_2$ **S7**.

SHIMADZU

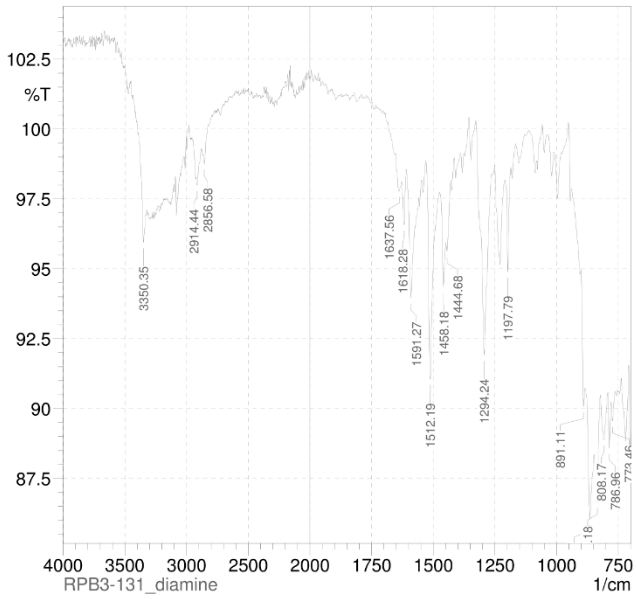

No.	Peak	Intensity	Corr. Inte	Base (H)	Base (L)	Area	Corr. Are
1	702.09	88.186	1.934	707.88	698.23	0.49	0.054
2	721.38	88.826	2.461	738.74	709.8	1.335	0.187
3	773.46	89.545	0.833	779.24	763.81	0.702	0.029
4	786.96	88.579	1.746	792.74	781.17	0.563	0.049
5	808.17	88.891	1.552	821.68	794.67	1.298	0.121
6	835.18	86.657	1.082	837.11	823.6	0.691	0.022
7	866.04	86.045	3.373	883.4	850.61	1.82	0.226
8	891.11	90.067	2.236	900.76	885.33	0.608	0.089
9	1197.79	94.885	3.526	1209.37	1186.22	0.278	0.119
10	1294.24	91.923	7.354	1327.03	1265.3	1.128	0.94
11	1444.68	95.625	0.758	1448.54	1429.25	0.285	0.034
12	1458.18	94.336	2.202	1473.62	1450.47	0.419	0.089
13	1512.19	91.05	7.455	1529.55	1475.54	1.074	0.668
14	1591.27	93.964	3.493	1608.63	1575.84	0.59	0.223
15	1618.28	96.576	1.785	1627.92	1610.56	0.195	0.068
16	1637.56	97.788	0.717	1653	1627.92	0.204	0.052
17	2856.58	98.758	0.58	2866.22	2823.79	0.107	0.038
18	2914.44	97.997	0.475	2922.16	2883.58	0.246	0.039
19	3350.35	95.946	1.821	3381.21	3334.92	0.531	0.163

Comment;
RPB3-131_diamine

Date/Time; 30/01/2023 13:46:17
No. of Scans; 16
Resolution; 4 [1/cm]
Apodization; Happ-Genzel

Figure S229. FT-IR spectrum of 4,5-dimethylbenzene-1,2-diamine-$^{15}N_2$ **S7**.

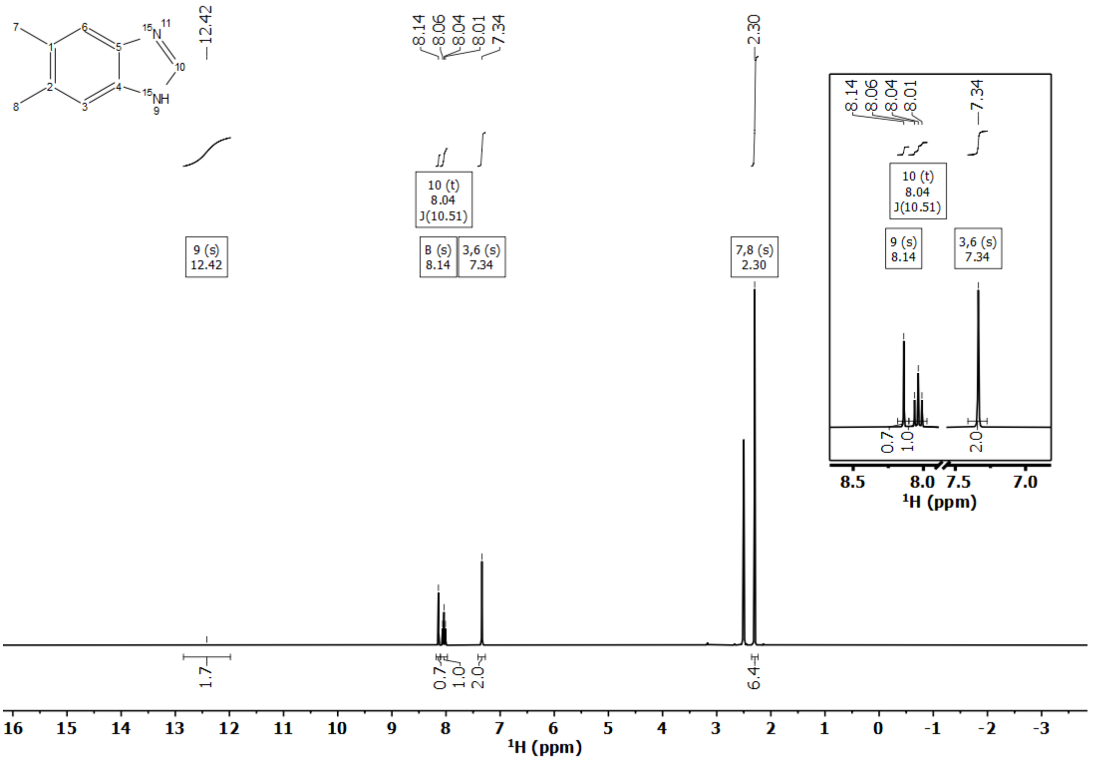

Figure S230. ^{1}H NMR spectrum of 5,6-dimethyl-1*H*-benzo[*d*]imidazole-1,3-$^{15}N_2$ **S8**.

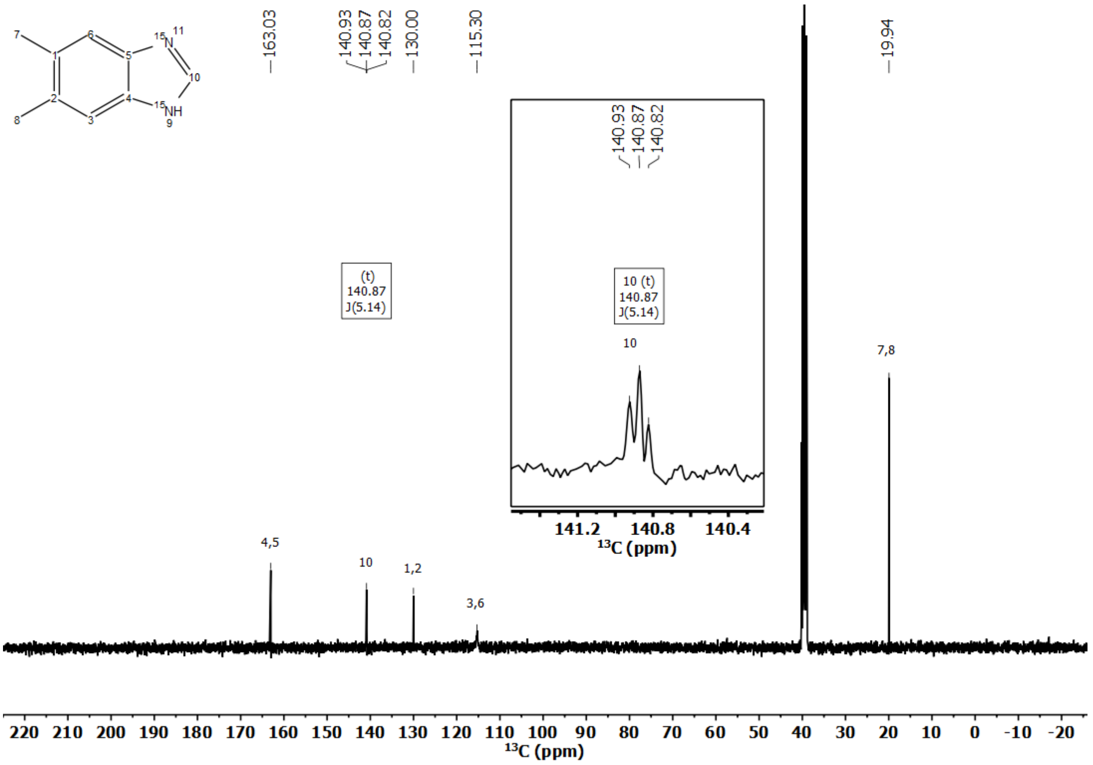

Figure S231. ^{13}C{^{1}H} NMR spectrum of 5,6-dimethyl-1*H*-benzo[*d*]imidazole-1,3-$^{15}N_2$ **S8**.

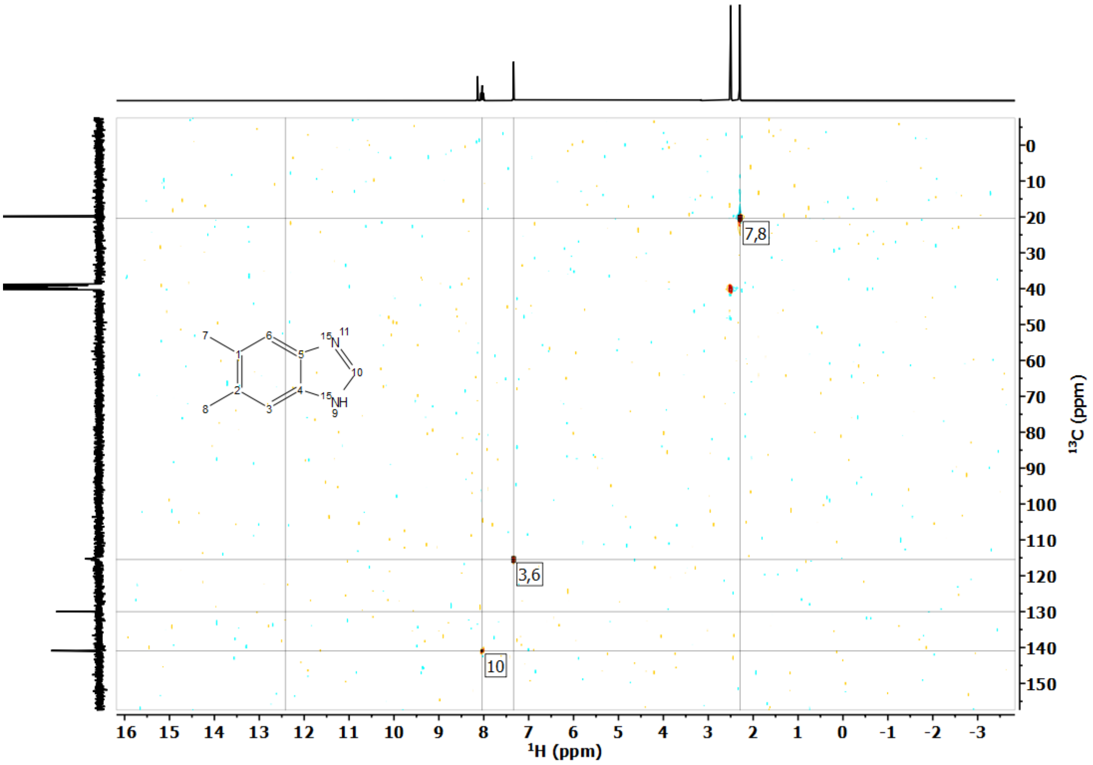

Figure S232. ^{1}H-^{13}C HSQC NMR spectrum of 5,6-dimethyl-1*H*-benzo[*d*]imidazole-1,3-$^{15}N_2$ **S8**.

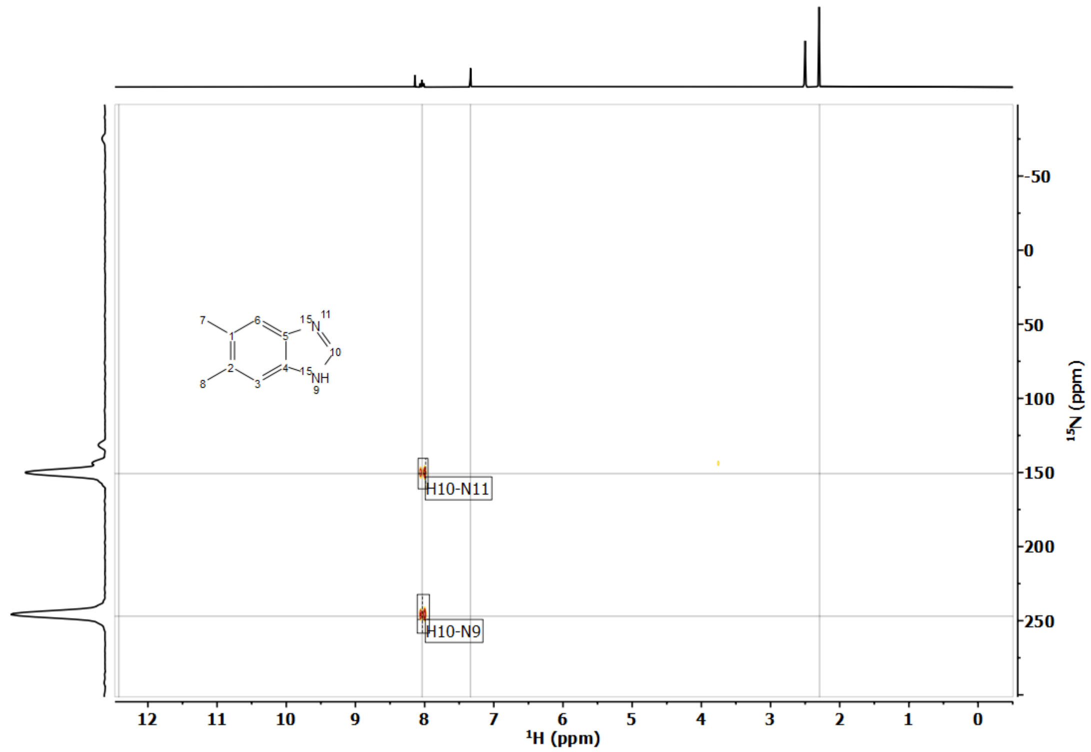

Figure S233. ^{1}H-^{15}N HMBC NMR spectrum of 5,6-dimethyl-1*H*-benzo[*d*]imidazole-1,3-$^{15}N_2$ **S8**.

Analysis Info

Analysis Name	D:\Data\Alans Data May 2021\STRA_Emma Campbell R3 133.d	Acquisition Date	5/18/2021 11:21:53 AM
Method	010713alanstune_low.m	Operator	Bruker UK
Sample Name	Emma Campbell R3 133	Instrument	microTOF 8213750.10408
Comment	Emma Campbell R3 133		

Acquisition Parameter

Source Type	ESI	Ion Polarity	Positive	Set Nebulizer	1.0 Bar
Focus	Active			Set Dry Heater	160 °C
Scan Begin	40 m/z	Set Capillary	3000 V	Set Dry Gas	4.0 l/min
Scan End	3000 m/z	Set End Plate Offset	-500 V	Set Divert Valve	Waste

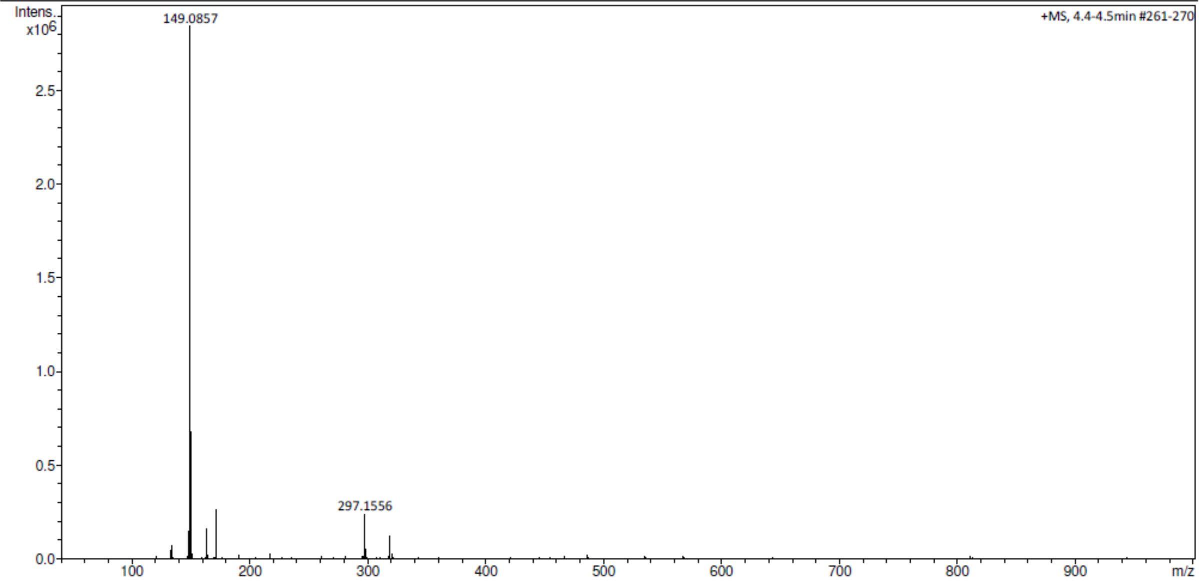

Figure S234. HRMS analysis of 5,6-dimethyl-1*H*-benzo[*d*]imidazole-1,3-$^{15}N_2$ **S8**.

SHIMADZU

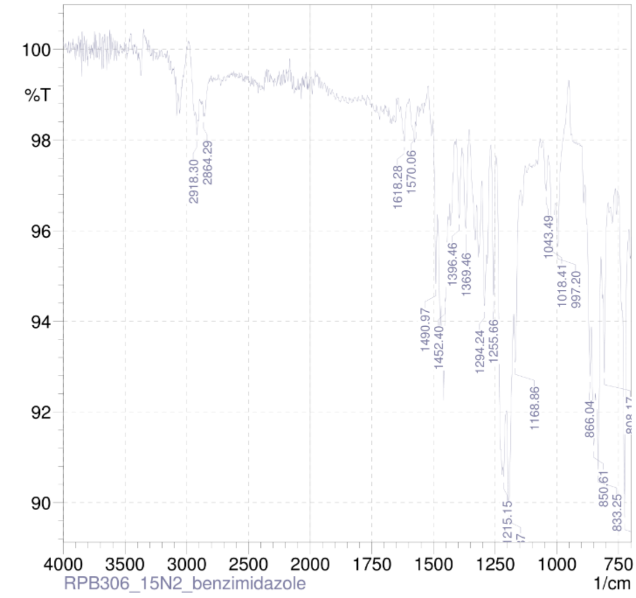

No.	Peak	Intensity	Corr. Inte	Base (H)	Base (L)	Area	Corr. Are
1	725.23	89.664	6.62	754.17	709.8	1.135	0.43
2	808.17	92.864	2.024	812.03	794.67	0.388	0.052
3	833.25	90.72	2.183	837.11	821.68	0.491	0.074
4	850.61	91.258	1.701	860.25	844.82	0.554	0.071
5	866.04	92.785	1.436	885.33	862.18	0.544	0.051
6	997.2	95.647	1.364	1004.91	952.84	0.549	0.082
7	1018.41	95.803	1.268	1035.77	1004.91	0.466	0.076
8	1043.49	96.87	0.904	1056.99	1035.77	0.248	0.045
9	1168.86	93.094	1.652	1174.65	1147.65	0.578	0.07
10	1195.87	89.847	2.775	1203.58	1176.58	1.022	0.188
11	1215.15	90.569	0.562	1219.01	1205.51	0.53	0.011
12	1255.66	94.574	3.172	1267.23	1247.94	0.321	0.131
13	1294.24	94.339	1.846	1303.88	1286.52	0.367	0.074
14	1369.46	96.042	2.004	1384.89	1355.96	0.367	0.117
15	1396.46	96.272	1.657	1408.04	1384.89	0.289	0.079
16	1452.4	94.433	0.346	1454.33	1440.83	0.277	0.012
17	1490.97	94.826	2.172	1502.55	1485.19	0.279	0.066
18	1570.06	98.306	0.236	1573.91	1564.27	0.063	0.003
19	1618.28	97.969	0.814	1633.71	1604.77	0.198	0.043
20	2864.29	98.516	0.193	2872.01	2858.51	0.08	0.005
21	2918.3	98.107	0.413	2931.8	2908.65	0.165	0.016

Comment;
RPB306_15N2_benzimidazole

Date/Time; 30/01/2023 13:50:45
No. of Scans; 16
Resolution; 4 [1/cm]
Apodization; Happ-Genzel

Figure S235. FT-IR spectrum of 5,6-dimethyl-1*H*-benzo[*d*]imidazole-1,3-$^{15}N_2$ **S8**.

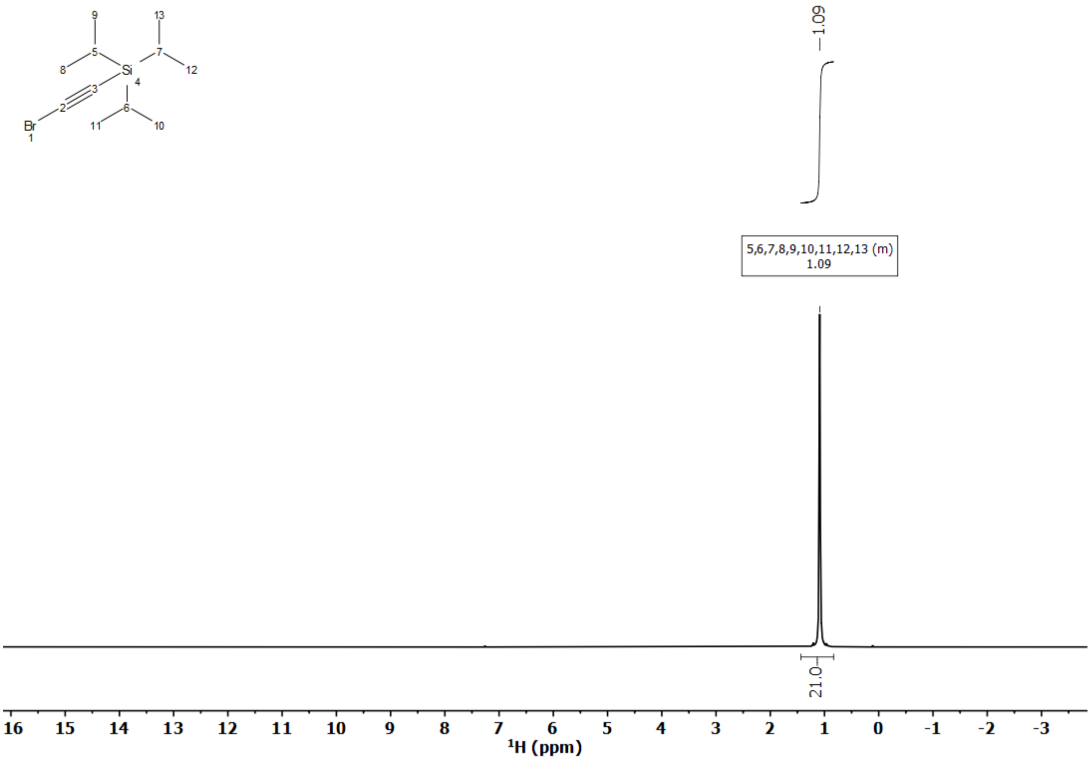

Figure S236. ^{1}H NMR spectrum of (bromoethynyl)Triisopropylsilane **S9**.

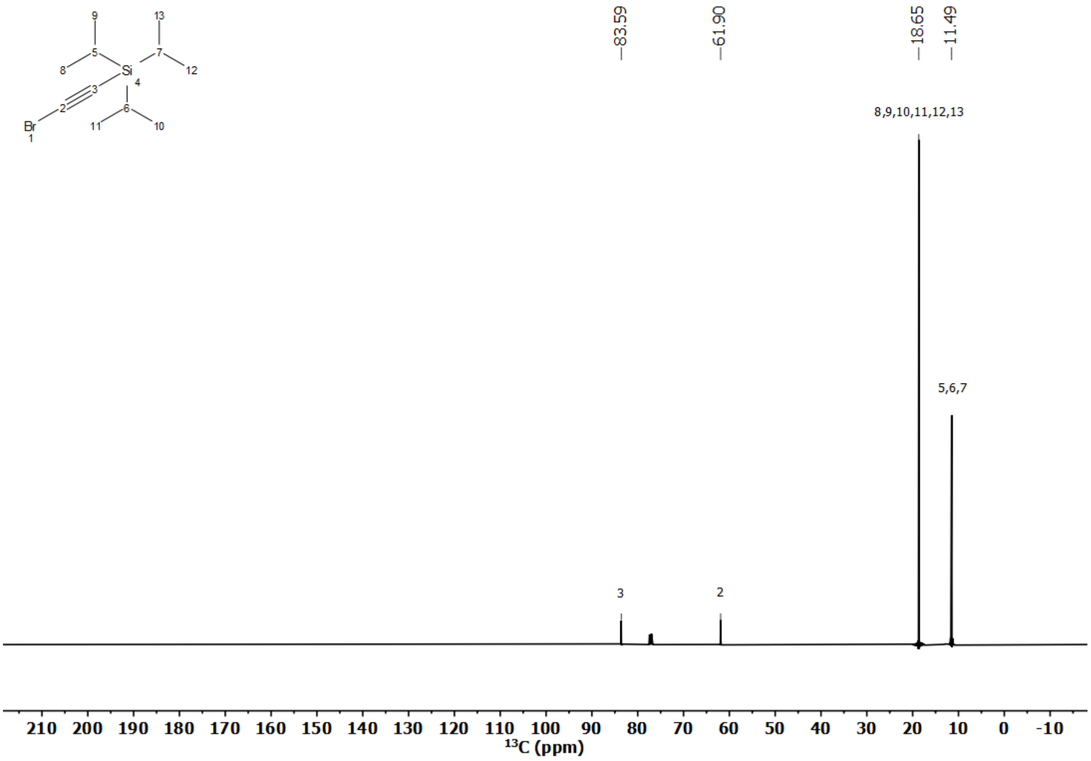

Figure S237. ^{13}C{^{1}H} NMR spectrum of {^{1}H} (bromoethynyl)Triisopropylsilane **S9**.

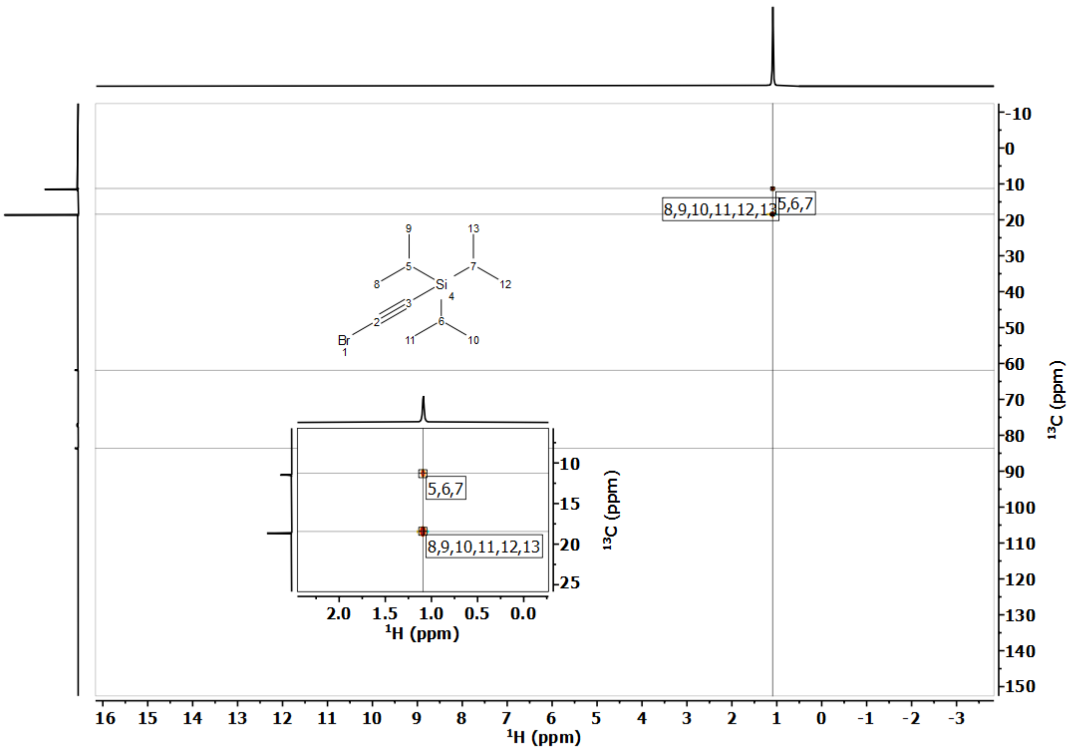

Figure S238. ^{1}H-^{13}C HSQC NMR spectrum of (bromoethynyl)Triisopropylsilane **S9**.

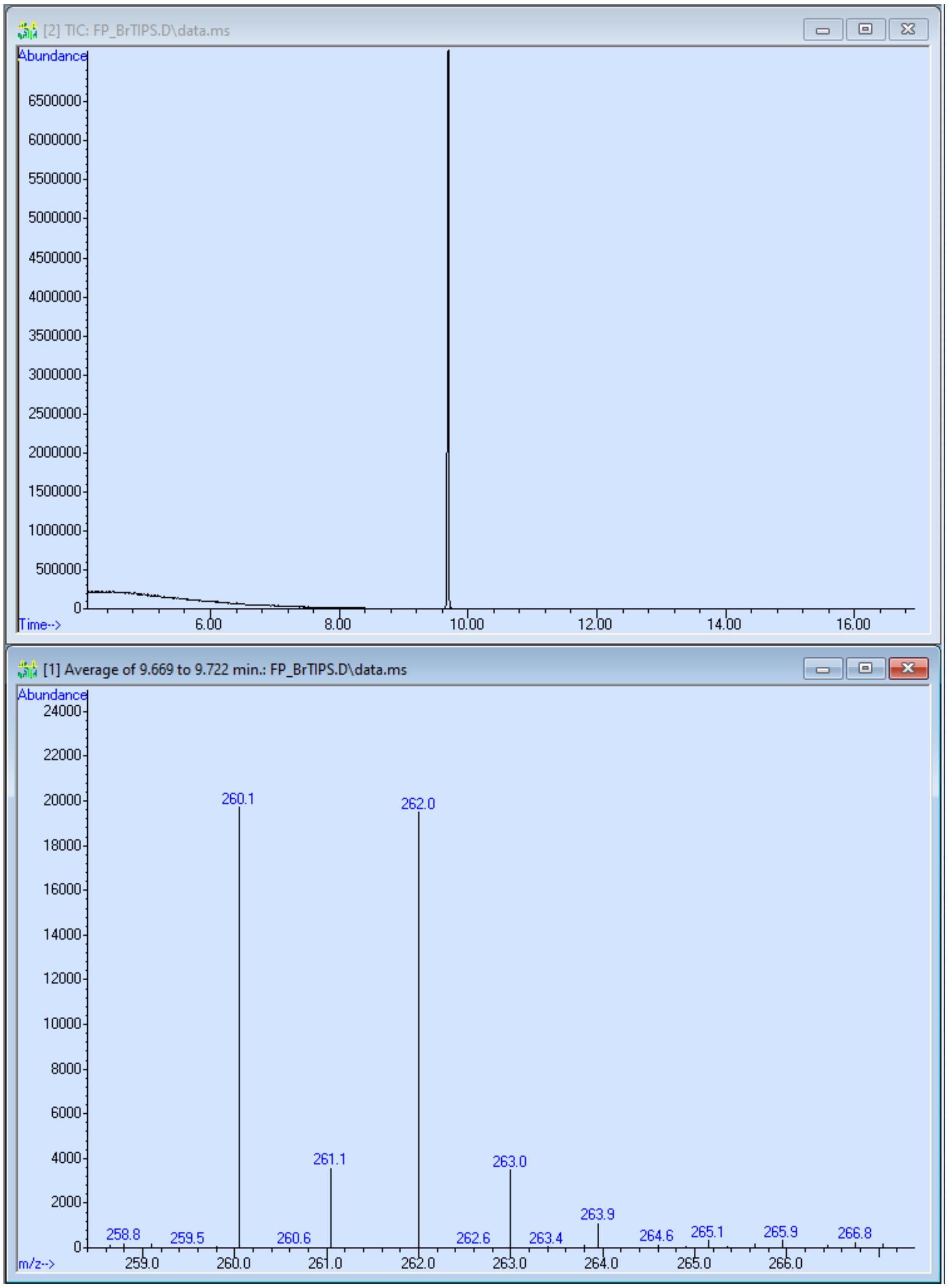

Figure S239. GC-MS analysis of (bromoethynyl)triisopropylsilane **S9**.

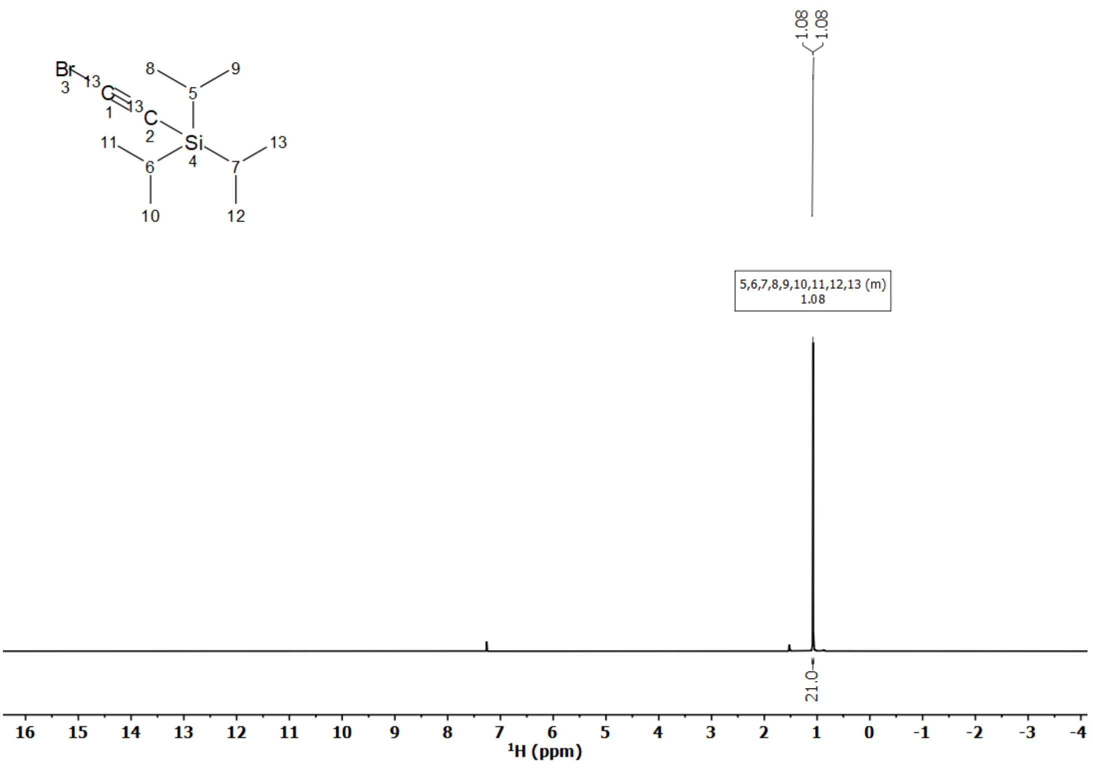

Figure S240. ^{1}H NMR spectrum of (bromoethynyl-1,2-^{13}*C*$_2$)triisopropylsilane **S9-^{13}C$_2$**.

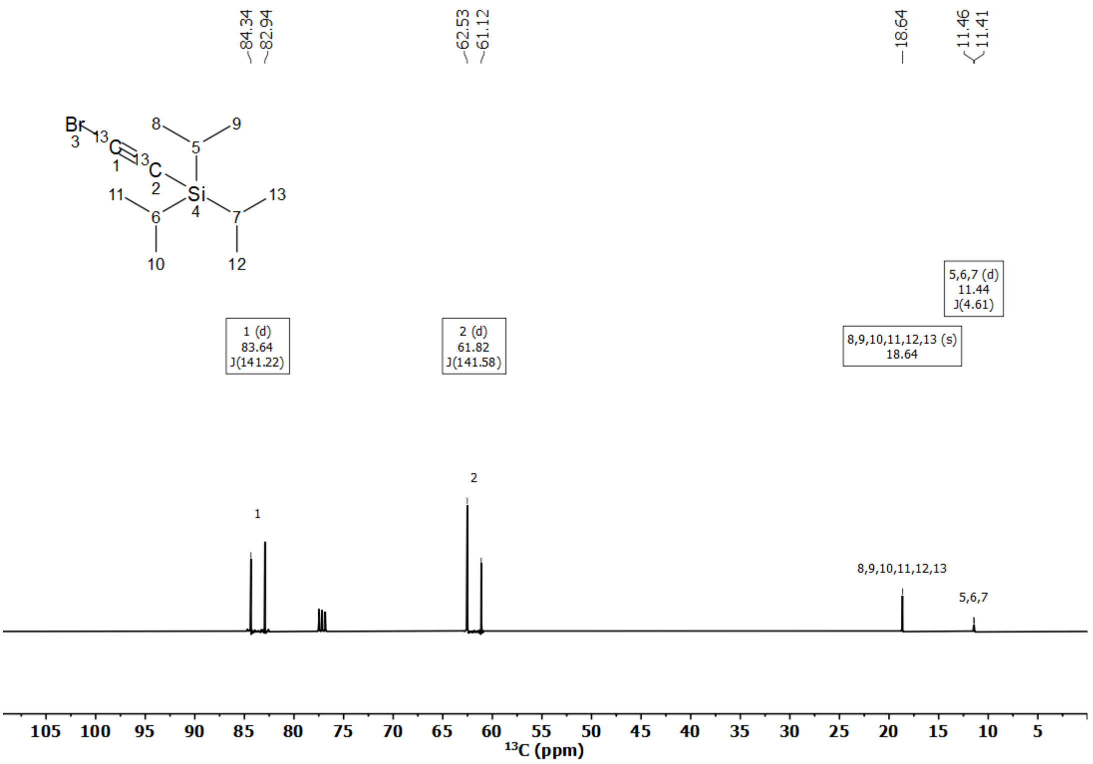

Figure S241. ^{13}C{^{1}H} NMR spectrum of (bromoethynyl-1,2-^{13}*C*$_2$)triisopropylsilane **S9-^{13}C$_2$**.

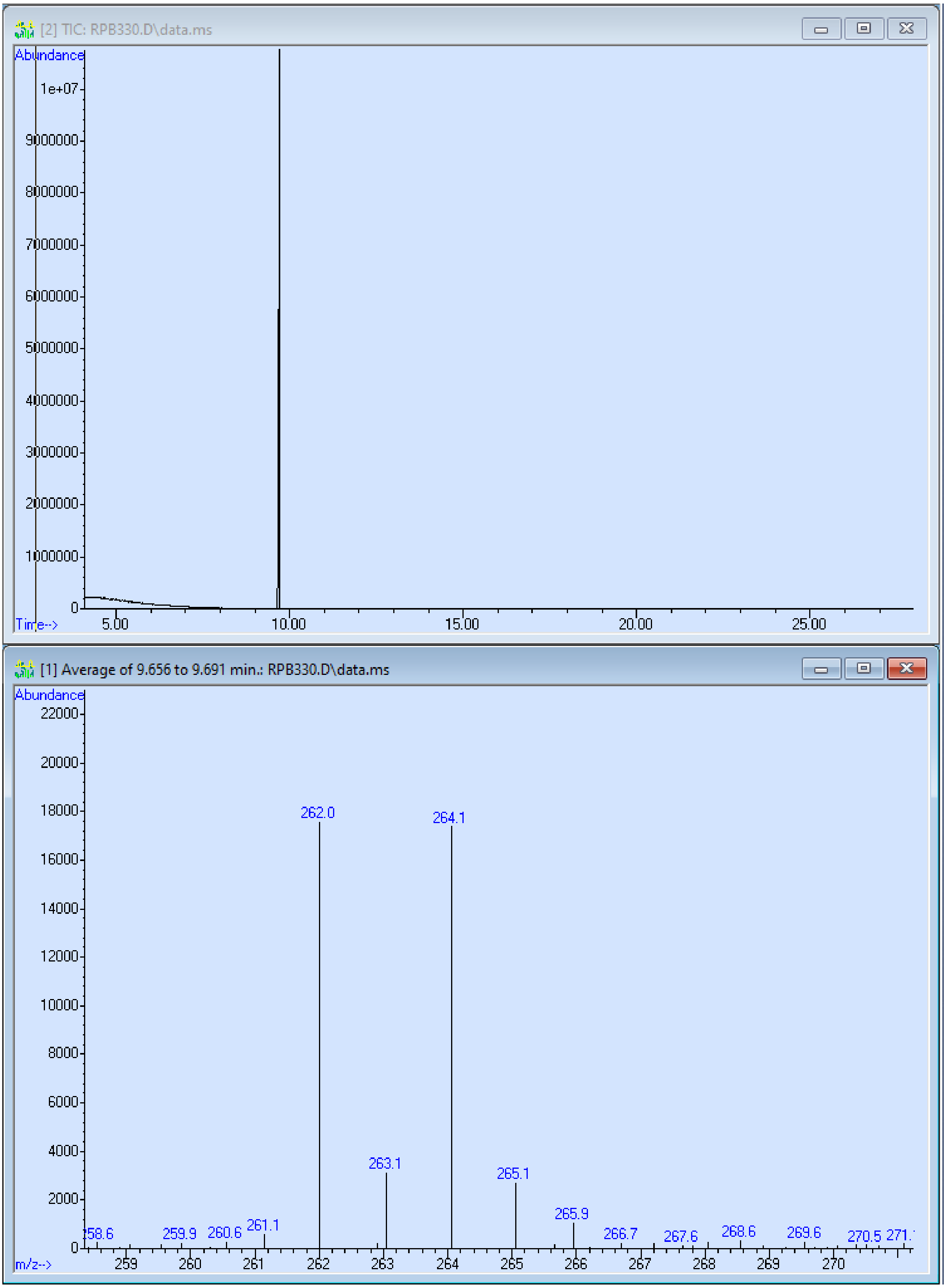

Figure S242. GC-MS analysis of (bromoethynyl-1,2-$^{13}C_2$)triisopropylsilane **S9-^{13}C$_2$**.

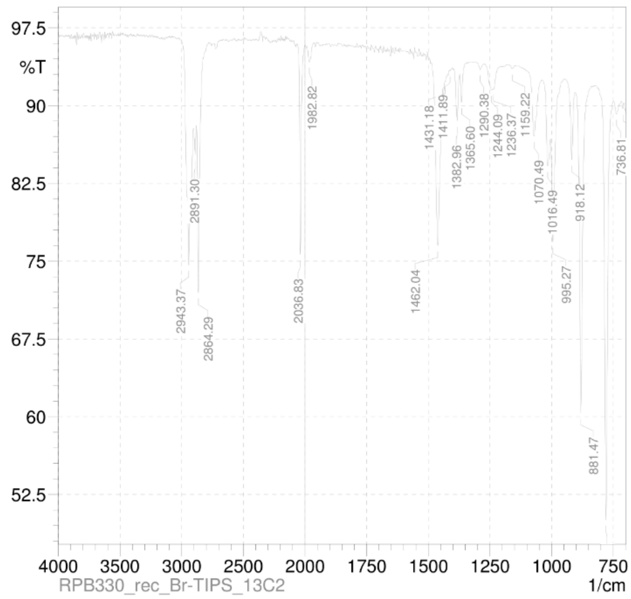

No.	Peak	Intensity	Corr. Inte	Base (H)	Base (L)	Area	Corr. Are
1	711.73	89.67	0.654	717.52	707.88	0.44	0.014
2	736.81	89.201	1.193	748.38	719.45	1.346	0.076
3	779.24	50.045	40.963	810.1	750.31	5.735	3.288
4	881.47	60.357	31.12	904.61	839.03	4.937	2.43
5	918.12	84.762	6.867	947.05	906.54	1.836	0.349
6	995.27	76.871	10.982	1004.91	952.84	3.017	0.645
7	1016.49	84.178	4.273	1037.7	1006.84	1.669	0.207
8	1070.49	87.043	5.897	1109.07	1039.63	2.771	0.577
9	1159.22	93.553	0.329	1168.86	1149.57	0.544	0.015
10	1236.37	91.6	0.146	1238.3	1209.37	0.916	0.007
11	1244.09	91.492	0.534	1271.09	1238.3	1.104	0.04
12	1290.38	93.486	0.588	1303.88	1276.88	0.748	0.031
13	1365.6	90.338	2.829	1371.39	1352.1	0.664	0.099
14	1382.96	88.521	4.737	1396.46	1373.32	0.915	0.215
15	1411.89	93.357	0.117	1415.75	1404.18	0.339	0.002
16	1431.18	92.295	0.132	1433.11	1423.47	0.318	0.001
17	1462.04	76.508	16.668	1487.12	1435.04	3.317	1.7
18	1982.82	94.223	1.59	1990.54	1965.46	0.549	0.092
19	2036.83	75.622	20.272	2059.98	2000.18	2.585	1.496
20	2864.29	71.961	17.738	2879.72	2812.21	3.916	1.44
21	2891.3	85.245	2.694	2902.87	2881.65	1.327	0.143
22	2943.37	74.619	7.871	2953.02	2904.8	4.395	0.883

Comment;
RPB330_rec_Br-TIPS_13C2

Date/Time; 30/01/2023 14:16:15
No. of Scans; 16
Resolution; 4 [1/cm]
Apodization; Happ-Genzel

Figure S243. FT-IR spectrum of (bromoethynyl-1,2-$^{13}C_2$)triisopropylsilane **S9-^{13}C$_2$**.

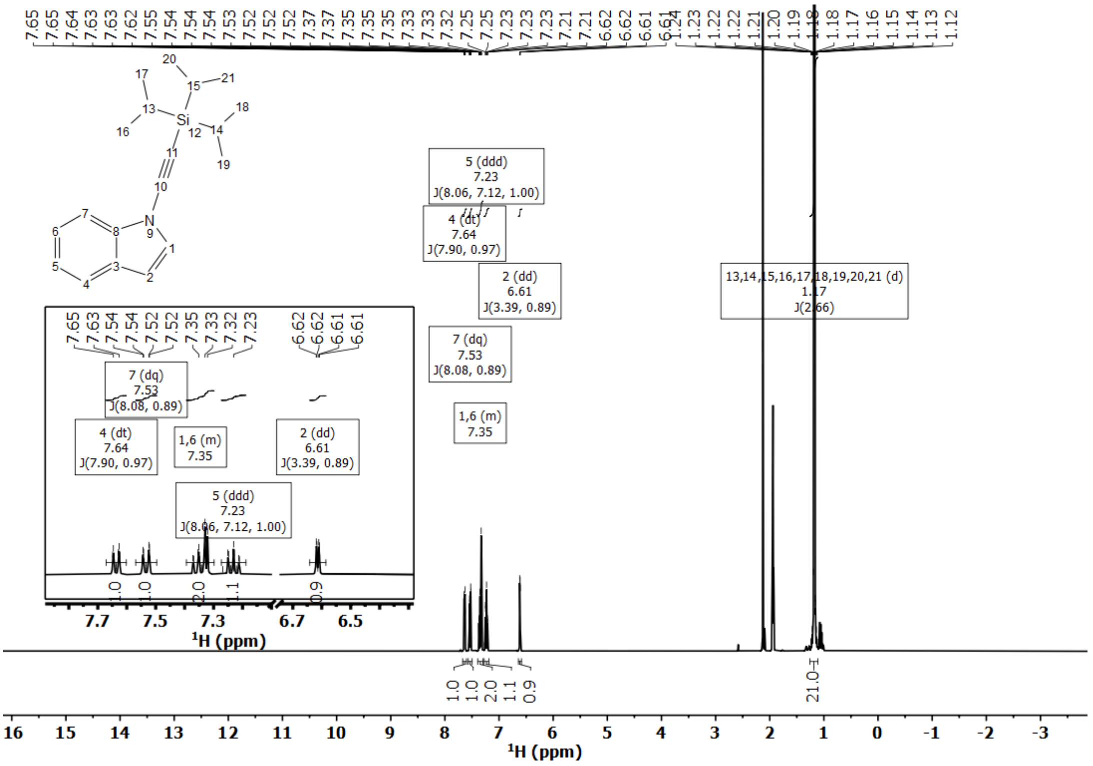

Figure S244. ^{1}H NMR spectrum of 1-((triisopropylsilyl)ethynyl)-1*H*-indole **S10**.

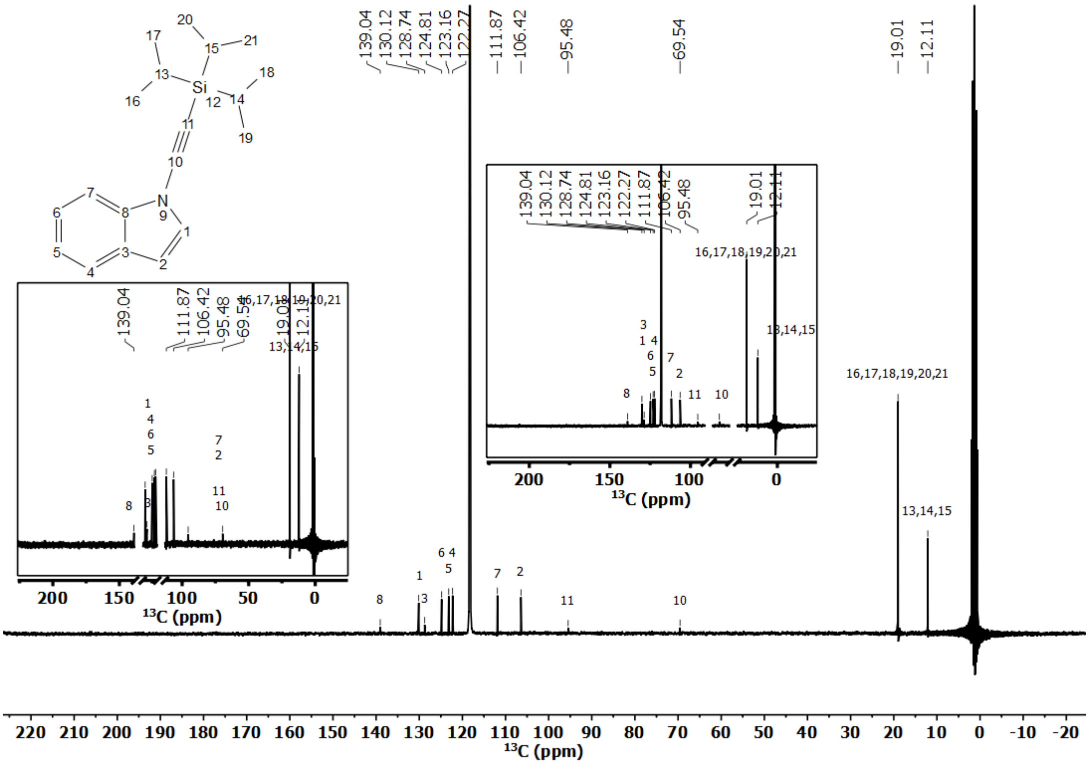

Figure S245. $^{13}C\{^{1}H\}$ NMR spectrum of 1-((triisopropylsilyl)ethynyl)-1*H*-indole **S10**.

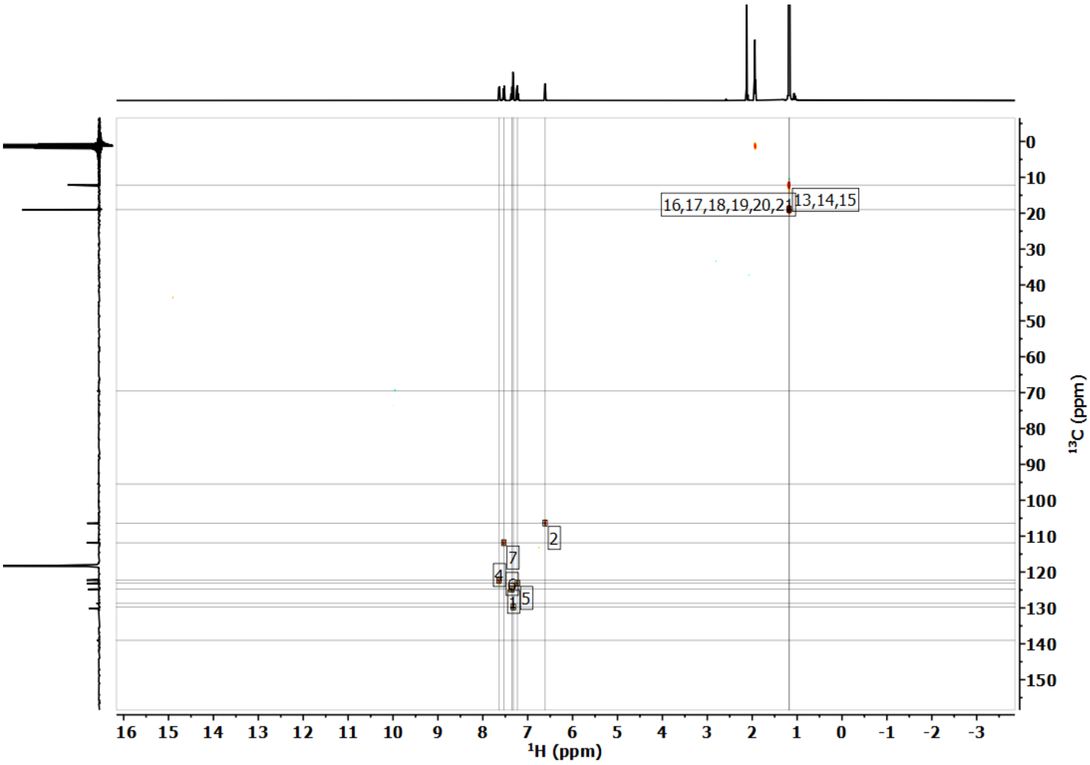

Figure S246. ^{1}H-^{13}C HSQC NMR spectrum of 1-((triisopropylsilyl)ethynyl)-1*H*-indole **S10**.

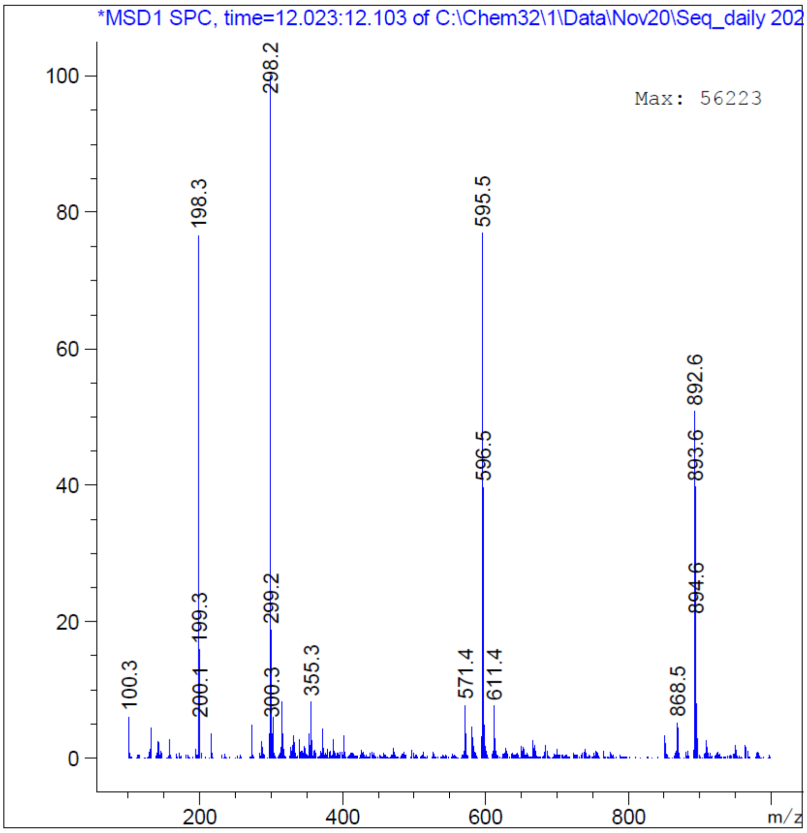

Figure S247. LC-MS analysis of 1-((triisopropylsilyl)ethynyl)-1*H*-indole **S10**.

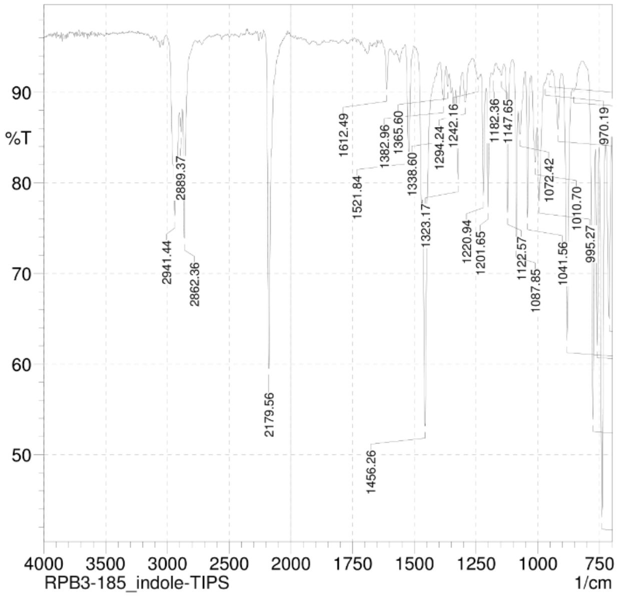

SHIMADZU

No.	Peak	Intensity	Corr. Inte	Base (H)	Base (L)	Area	Corr. Are
1	711.73	64.978	20.941	725.23	698.23	3.082	1.311
2	740.67	43.087	40.183	750.31	727.16	4.594	2.783
3	759.95	62.03	20.585	765.74	752.24	1.896	0.798
4	777.31	53.877	31.789	815.89	767.67	4.172	1.765
5	854.47	90.145	0.538	858.32	823.6	1.3	0.047
6	881.47	62.583	28.613	904.61	860.25	3.711	1.935
7	918.12	85.897	6.268	943.19	906.54	1.69	0.403
8	952.84	91.919	0.329	958.62	945.12	0.483	0.013
9	970.19	90.992	0.249	972.12	960.55	0.447	0.008
10	995.27	77.98	9.449	1002.98	974.05	2.085	0.582
11	1010.7	82.255	5.218	1026.13	1004.91	1.423	0.297
12	1041.56	76.164	15.937	1051.2	1028.06	1.407	0.574
13	1072.42	85.359	3.313	1078.21	1053.13	1.337	0.198
14	1087.85	73.145	16.833	1101.35	1080.14	1.412	0.508
15	1122.57	76.726	16.577	1136.07	1103.28	1.61	0.633
16	1147.65	91.859	0.917	1155.36	1138	0.594	0.034
17	1182.36	91.952	0.529	1188.15	1170.79	0.591	0.022
18	1201.65	77.317	13.802	1209.37	1190.08	1.194	0.438
19	1220.94	78.571	12.569	1230.58	1211.3	1.255	0.469
20	1242.16	91.377	1.112	1265.3	1232.51	1.129	0.069
21	1294.24	88.943	3.614	1303.88	1267.23	1.383	0.223
22	1323.17	80.334	10.139	1330.88	1305.81	1.436	0.414
23	1338.6	86.02	4.325	1346.31	1332.81	0.74	0.145
24	1365.6	90.619	1.81	1371.39	1359.82	0.449	0.049
25	1382.96	89.072	3.904	1396.46	1373.32	0.921	0.192
26	1456.26	53.144	27.482	1467.83	1398.39	5.912	1.762
27	1521.84	83.466	11.736	1537.27	1504.48	1.434	0.733
28	1612.49	90.314	4.846	1631.78	1598.99	0.91	0.211
29	2179.56	59.506	36.225	2220.07	2112.05	7.594	5.354
30	2862.36	73.898	15.3	2877.79	2777.5	4.584	1.211
31	2889.37	85.617	2.37	2902.87	2879.72	1.42	0.132
32	2941.44	76.334	14.344	3010.88	2904.8	6.494	2.669

Comment;
RPB3-185_indole-TIPS

Date/Time; 30/01/2023 14:50:26
No. of Scans; 16
Resolution; 4 [1/cm]
Apodization; Happ-Genzel

Figure S248. FT-IR spectrum of 1-((triisopropylsilyl)ethynyl)-1*H*-indole **S10**.
